# Peripheral Immune Modulation in Atopic Dermatitis During Dupilumab or Baricitinib Treatment Is Limited, as Assessed by Proteomic, Transcriptomic, and Torque Teno Virus Analyses

**DOI:** 10.3390/ijms27188056

**Published:** 2026-09-10

**Authors:** Toke Touborg, Anne Sofie Frølunde, Thomas Litman, Randi Berg, Pernille Koefoed-Nielsen, Mette Deleuran, Claus Johansen, Christian Vestergaard

**Affiliations:** 1 Department of Dermatology and Venereology, Aarhus University Hospital, 8200 Aarhus, Denmark; 2 Department of Clinical Medicine, Aarhus University, 8200 Aarhus, Denmark; 3 Department of Immunology and Microbiology, University of Copenhagen, 1165 Copenhagen, Denmark; 4 LEO Pharma, 2750 Ballerup, Denmark; 5 Department of Clinical Immunology, Aarhus University Hospital, 8200 Aarhus, Denmark

**Keywords:** atopic dermatitis, blood marker, transcriptomics, proteomics, torque teno virus, dupilumab, baricitinib

## Abstract

Atopic dermatitis is a common inflammatory skin disease affecting up to 10% of adults. Whether atopic dermatitis exhibits a strong systemic inflammatory signature remains debated. We characterized peripheral whole-blood alterations in adults with moderate-to-severe atopic dermatitis undergoing treatment with dupilumab or baricitinib therapy. Blood samples were collected at weeks 0, 4, and 16. Whole-blood proteins were measured using Olink, transcriptomic profiling was performed by RNA sequencing, and torque teno virus plasma levels were quantified by qPCR. During targeted atopic dermatitis therapy with dupilumab or baricitinib, clustering of samples based on gene expression levels showed no separation by time or treatment, with only a limited number of differentially expressed genes. Proteomic changes were similarly modest; however, treatment was consistently associated with decreased CCL17/TARC levels. Teno torque virus plasma load remained stable throughout therapy, indicating preserved immunocompetence. These findings suggest that the dominant inflammatory processes in atopic dermatitis may be largely tissue-restricted, supporting the use of peripheral biomarkers for pragmatic monitoring rather than mechanistic discovery.

## 1. Introduction

Atopic dermatitis (AD) is a chronic inflammatory skin disease primarily driven by type II inflammation, mainly mediated by Interleukin (IL)-4 and IL-13. It affects up to 20% of children and up to 10% of the adult population in affluent countries [1,2]. It is burdensome for those affected and patients have a significantly reduced quality of life [3]. Atopic dermatitis is linked to other type II inflammatory disorders such as asthma, rhino-conjunctivitis and food allergies, as well as psychiatric comorbidities. Emerging evidence also suggests associations with cardiovascular diseases, such as myocardial infarction and atherosclerosis, and with autoimmune disorders [1]. Atopic dermatitis may therefore be considered a systemic inflammatory disease [4]. Several studies have demonstrated systemic immune activation in AD, evidenced by increased levels of inflammatory markers in the blood [5,6]. One of the most reliable serum indicators of inflammatory activity in AD is CC chemokine ligand 17/thymic activation and regulated chemokine (CCL17/TARC), although others, including IL-4 and periostin, have also been investigated [5].

Torque teno virus (TTV) is a ubiquitous, clinically non-pathogenic virus, and its plasma levels have been proposed as an immune-status-monitoring biomarker in, among other conditions, solid organ and hematopoietic stem cell transplant recipients. Torque teno virus replication appears to increase in states of reduced immunocompetence, resulting in an inverse relationship between viral plasma levels and host immunocompetence. In solid organ transplant, a lower torque teno virus concentration is associated with acute graft rejection, and torque teno virus concentration is inversely correlated with CD4+ T cell count in human immunodeficiency virus (HIV) patients [7,8,9,10,11]. This dynamic relationship, along with a relatively high prevalence of torque teno virus in the general population [12], makes torque teno virus a reasonable candidate proxy when examining longitudinal immunocompetence across individuals and conditions.

Targeted systemic therapies, like dupilumab (anti-IL-4Rα, blocking IL-4/IL-13 signaling [13]) and the Janus kinase 1/2 (JAK1/2) inhibitor baricitinib [14], offer an opportunity to test whether effective treatment of AD yields measurable peripheral immunological changes, as well as the effect on immunocompetence. Of special interest are the immunological changes in the Th2-related axis, which is thought to be a main driver and contributor to the pathophysiology of several barrier-related diseases, including atopic diseases [15].

We report transcriptomic, proteomic, and torque teno virus load changes in whole-blood samples from AD patients undergoing dupilumab or baricitinib therapy.

## 2. Results

### 2.1. RNA Sequencing

A total of 81 sequential whole-blood samples from 31 patients across 3 study visits were analyzed. These included 71 samples collected during dupilumab treatment, 7 collected during baricitinib treatment, and two samples obtained during either dupilumab or baricitinib treatment.

**Figure 1:**
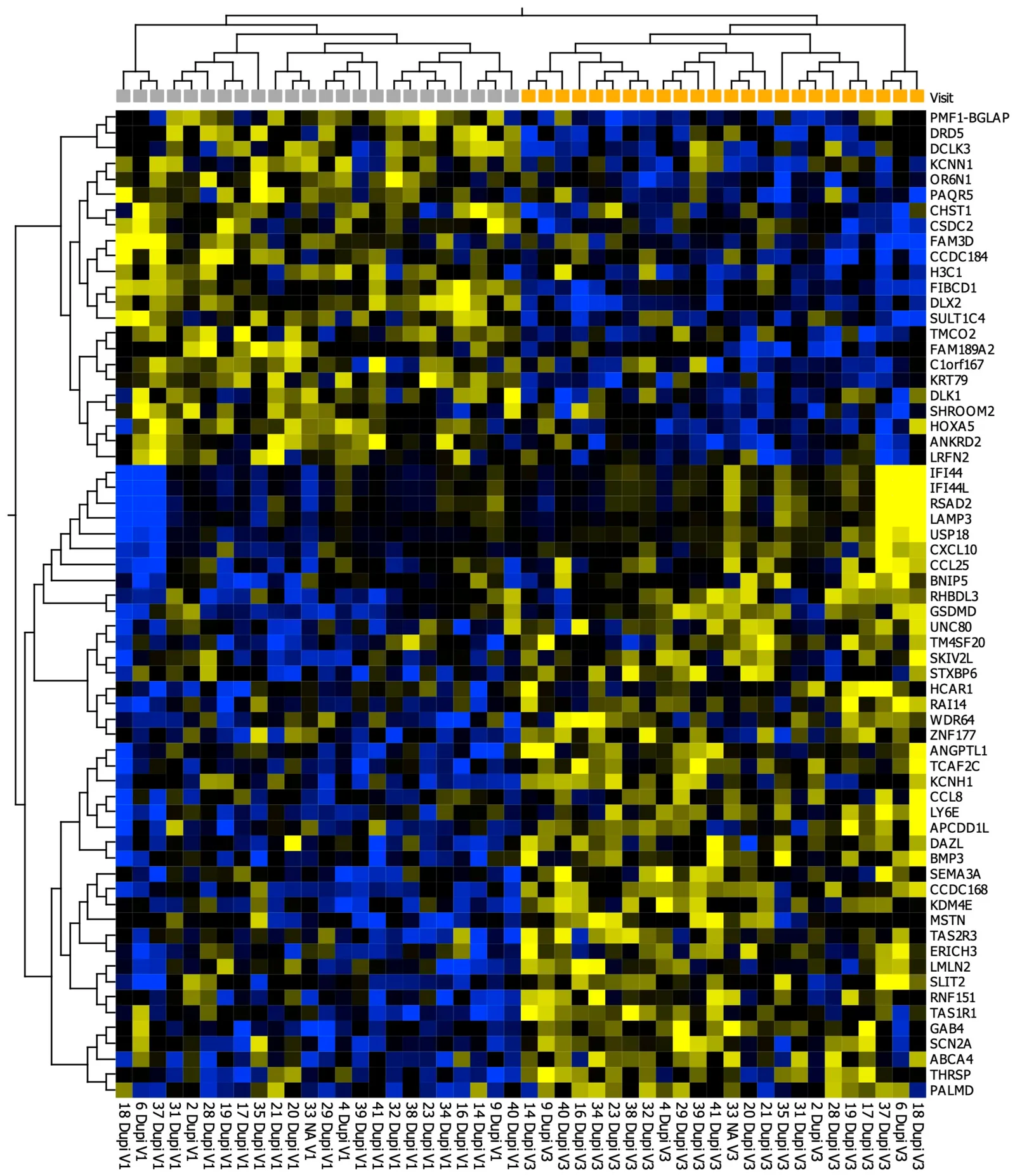
RNAseq results. Heatmap of differentially expressed genes (DEGs). Dupilumab, V3 (week 16) vs. V1 (week 0): 64 DEGs (Var > 0.1, *p* < 0.05, q_max_ = 0.57, >2-fold change, patient eliminated as factor, 23 pairs).

When comparing week 0 (V1) with week 16 (V3), 64 differentially expressed genes (DEGs) were identified using cutoffs of variance > 0.1, *p* < 0.05 and >2-fold change. The data are presented as a heatmap with two-way unsupervised hierarchical clustering (Figure 1).

It should be noted that, except for one gene, DLX2, the 64 genes did not meet a conventional FDR (q-value) threshold of 0.05. Thus, these should be regarded as nominally differentially expressed candidate genes. The DEG list, including *p*-, q-, and log2 fold change for the V3 vs. V1 contrast, is included as Supplementary Table S1.

We compared DEGs between responders and non-responders (EASI improvement) and found no correlations between these DEGs and EASI scores.

### 2.2. Olink Proteomics

**Figure 2:**
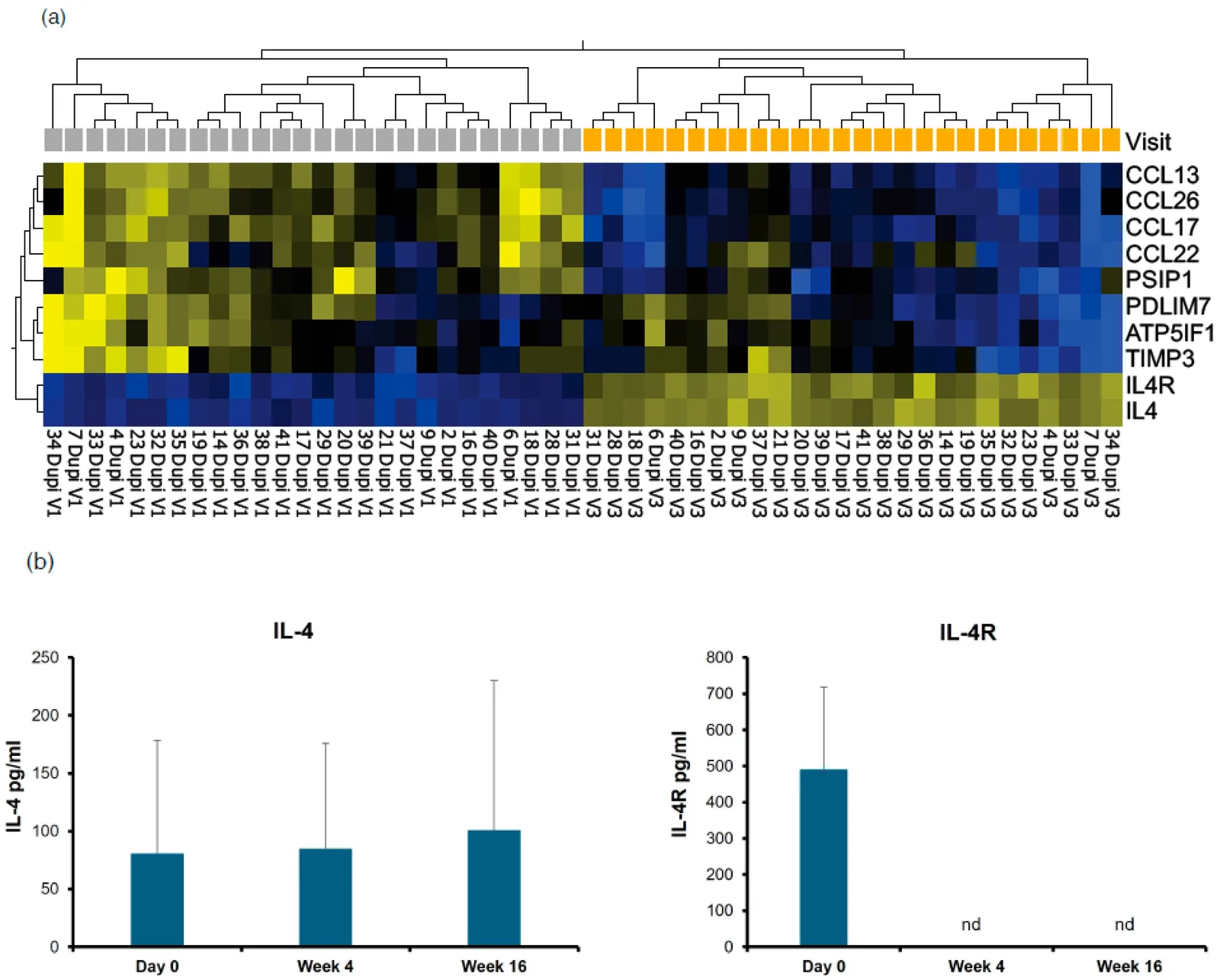
(**a**) Olink results. Heatmap of differentially expressed proteins (DEPs), Dupilumab, V3 vs. V1: 10 DEPs (Var > 0.1, *p* < 0.05, q_max_ = 0.29, log2 fold change > 1.5, patient eliminated as factor, 26 pairs). (**b**) ELISA results. *X*-axis = week, *Y*-axis = IL4/IL4R concentration (pg/mL), Interleukin-4 (IL-4), Interleukin-4 receptor (IL-4R), nd = not detectable.

In total, 88 sequential plasma samples from 31 patients were analyzed, including 73 samples obtained during dupilumab treatment and 7 during baricitinib treatment. Two-way hierarchical clustering based on 10 differentially expressed proteins (DEPs) suggested a treatment-associated shift, with several proteins showing lower abundance during dupilumab treatment (Figure 2a). Six of the 10 proteins met significance after multiple testing correction (q < 0.05), while 4 were only nominally significant (Supplementary Table S2).

When comparing V3 to V1, CCL17/TARC showed the largest decrease (−1.94 FC, *p* = 2.83 × 10^−7^, q = 3.14 × 10^−5^). CCL26 and CCL13 also decreased, along with other proteins (Supplementary Table S2), although to a lesser extent.

Surprisingly, IL-4 and IL-4R appeared to increase with treatment (Supplementary Table S2). To validate this finding, IL-4 and IL-4R were measured by ELISA using commercial kits (R&D Systems, Minneapolis, MN, USA and Abcam, Cambridge, United Kingdom). ELISA showed no significant differences in IL-4 concentration across samples or time points (Figure 2b). In contrast, IL-4R showed clear downregulation after treatment, with detectable levels at baseline and non-detectable (nd) levels after treatment (Figure 2b).

### 2.3. Torque Teno Virus

We analyzed torque teno virus plasma concentrations from week 0 (V1), 4 (V2) and 16 (V3). We excluded patients with 0 copies/mL in all samples from the longitudinal analysis, as this was considered a surrogate for absence of detectable torque teno virus infection (n = 12). Torque teno virus values were log2-transformed, and paired comparisons were performed for week 4 and week 16 versus baseline.

**Figure 3:**
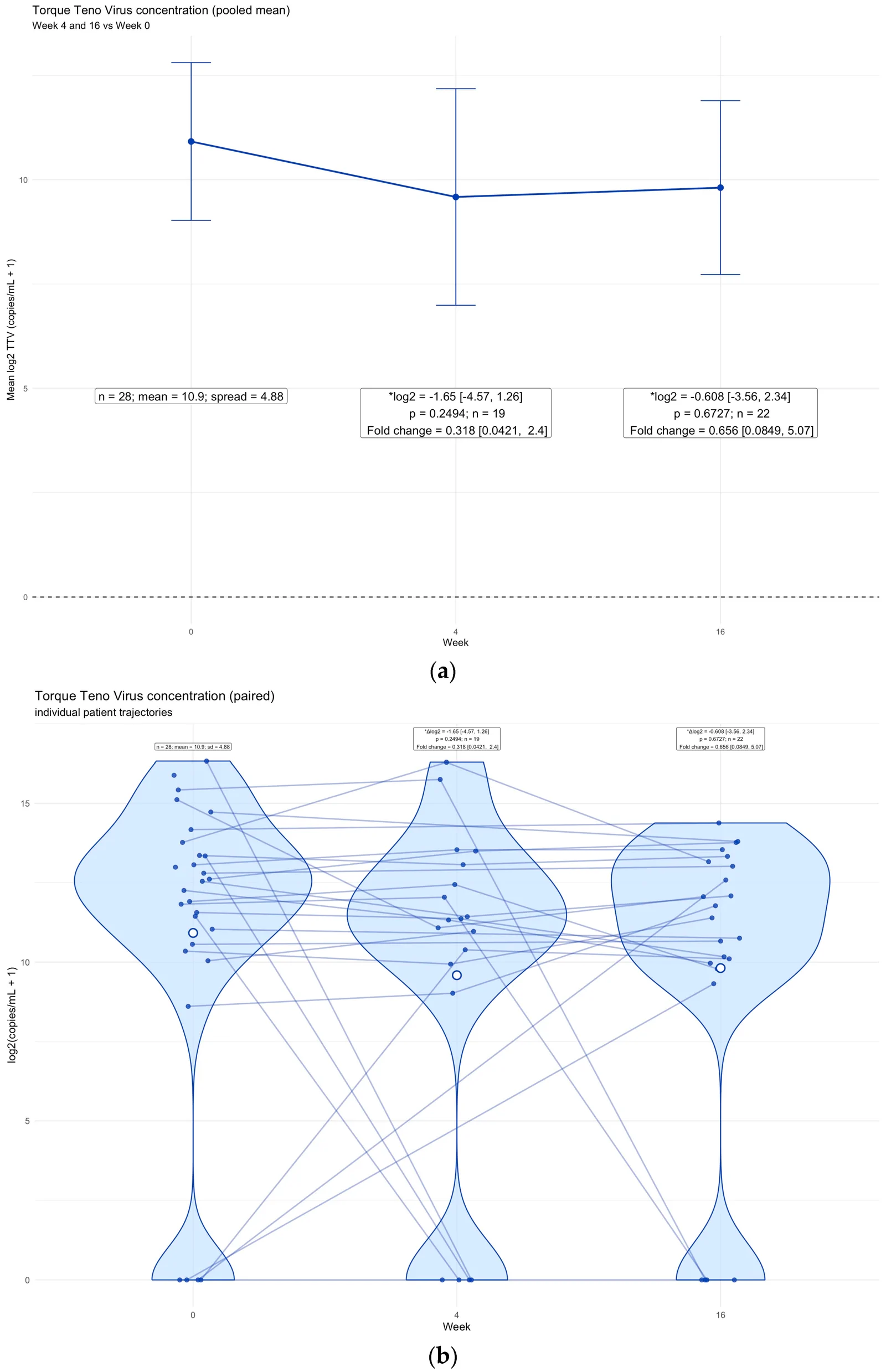
(**a**) *X*-axis = week, *Y*-axis = mean log2 torque teno virus (TTV) concentration (copies/mL + 1), * = Δ-value, FC = fold change. n = number of patients. (**b**) *X*-axis = week, *Y*-axis = log2 Torque teno virus (TTV) concentration (copies/mL + 1), blue dot = individual patient values, white dot = mean TTV concentration, lines = individual patient trajectories.

Mean torque teno virus levels were stable during treatment with no significant change in mean virus concentration across the cohort (Figure 3a), and individual patient trajectories likewise showed no substantial overall change (Figure 3b).

## 3. Discussion

In this study, we investigated peripheral blood transcriptomic, proteomic and torque teno virus changes in patients with atopic dermatitis during dupilumab and baricitinib treatment.

Unexpectedly, neither therapy resulted in marked alterations in the whole-blood transcriptome, and changes in circulating protein levels were modest. However, this systemic stability underscores a critical concept in precision medicine: the profound immunopathology of AD and the defensive response of the living tissue to targeted therapy may very likely be compartmentalized to the skin microenvironment and its local draining lymph nodes.

Torque teno virus levels remained stable throughout treatment, which is consistent with preserved immunocompetence during dupilumab and baricitinib therapy. This rationale is based on other studies examining the correlation between torque teno virus and immunocompetence [9,10]. This is, to our knowledge, the first study of torque teno virus concentration in a skin disease patient cohort. Torque teno virus and the proposed inverse relationship with immunocompetence hold the potential to monitor the immunocompetence effect in many aspects of medicine, including therapeutic implications [16]. Our study highlights the preserved immunocompetence measured by torque teno virus concentration within the limits of this proxy and adds to the evidence of the specificity and immunomodulating effects of dupilumab and baricitinib in AD treatment.

Atopic dermatitis is increasingly recognized for its systemic implications, including atherosclerosis, cardiovascular disease and systemic atopic diseases such as asthma and rhinoconjunctivitis [4]. A major translational challenge is determining whether these complications stem from a shared underlying cause of atopic dermatitis and the systemic complications or from atopic dermatitis and its systemic implications. Current theories on the pathophysiology of AD are centered around a dichotomous view: the outside-in hypothesis, which assumes an impaired barrier function (including filaggrin (*FLG*) mutations), resulting in increased transepidermal water loss and enhanced allergen exposure, that leads to subsequent inflammation; and the inside-out hypothesis, which assumes primary immune defects, leading to aberrant inflammation and secondary decreased barrier function, resulting in subsequent enhanced allergen penetration [1]. In both hypotheses, the pathophysiology creates a vicious inflammatory circle that is maintained by each of the contributing factors within itself. Biochemically, AD can be classified based on the dominant immune “endotype” that is present. These endotypes can be classified according to IgE levels: the classical (80%) “extrinsic” phenotype with high IgE serum levels, eosinophilia, family history of atopic diseases and higher prevalence of filaggrin mutations (*FLG*), which exhibit sustained barrier failures; and an “intrinsic” endotype that is characterized by low IgE serum levels, preserved barrier function, no background of atopic diseases and female predominance [17]. Interestingly, in line with this characterization, a German study found that whole-blood transcriptomics in this cohort generally identified distinct profiles only in the eosinophilic-high subpopulation, prompting the question whether all AD endotypes exhibit a dysregulated peripheral profile [18].

A recent American study showed a correlation between atherosclerosis-related products and Th2-related inflammatory markers in serum; both were modulated by dupilumab; however, no significant vascular inflammation assessed by PET-CT was found [19]. This study showcases the overlapping inflammatory, treatable Th2 markers in AD and atherosclerosis, but it does not address a highly specific shared disease mechanism.

One of the proteomic changes found in this study was a reduction in CCL17/TARC during dupilumab treatment, consistent with previous reports identifying CCL17/TARC as one of the most robust blood biomarkers of AD activity and severity [5,20]. This decrease has also been shown following other targeted (lebrikizumab, IL-13 inhibitor) and broad (cyclosporine) AD treatments [21,22]. CCL17/TARC cytokine is a member of the Th2 chemokine family [5] and signals through CCR4 in the skin [23]. CCL17/TARC, among other Th2-related products and Th2-type cells, is significantly upregulated across various AD cohorts [5]. A recent Danish study found an association between skin CCL17/TARC levels and the cumulative risk of developing AD within the first 2 years of life and the association was even stronger in moderate-to-severe AD [24], highlighting the role of skin Th2 perturbation in AD. While cyclosporine suppresses proteomics in both Th1, Th2, and Th17 inflammatory pathways peripherally [22], targeted therapies provide a more tailored Th2 immunomodulation [21,25]. By specifically neutralizing the Th2-derived chemokine gradients, and possibly IL-13 [26], that anchor localized dendritic cell (DC) [27] networks to the dermis, targeted therapies, in part, halt tissue-specific amplification of inflammatory circuits. This dampens the chronic inflammatory milieu in the skin and, theoretically, the systemic spillover that is hypothesized to be a driver of the comorbidities seen in AD, similar to the systemic involvement seen in other inflammatory skin diseases like psoriasis [28], without causing the massive peripheral disruption and broad severity marker (e.g., OX40) suppression seen with systemic immunosuppressants [22].

Our findings of a stable whole-blood transcriptome and a relatively discrete peripheral proteomic profile, as well as a preserved torque teno virus load, suggest that targeted therapies like dupilumab and baricitinib, to some extent, decouple localized skin inflammation from systemic spillover. However, while patients improve clinically during treatment, the peripheral reactomic shift remains limited. This leaves an evidential gap between the pathophysiology and the evident systemic correlations in AD that are yet to be elucidated.

Translating basic research into clinical practice requires recognizing the complex interplay between the skin barrier and inflammation. True clinical resolution likely necessitates active tissue repair, not just passive cytokine suppression. Stabilizing the pivotal actors in barrier integrity may well be fundamental to eradicating disease pathogenesis, yet these highly localized, protective shifts remain virtually invisible in peripheral whole-blood sampling.

The lack of peripheral transcriptomic volatility in AD becomes particularly striking when contrasted with other inflammatory skin diseases. For example, psoriasis driven heavily by Th1/Th17 and neutrophilic pathways frequently exhibits a prominent systemic blood transcriptome signature that directly overlaps with broader metabolic and cardiovascular disease pathways [29]. While AD shares some systemic burden, our findings suggest that its core pathophysiology is disproportionately anchored in the local barrier-immune crosstalk. This local entrapment may be perpetuated by the persistence of tissue-resident memory T cells (TRMs) [30,31]. TRMs that produce pro-inflammatory cytokines have been shown to persist in resolved AD lesions and are thought to harbor immunogenic residual memory and thus contribute to recurrent flares and inflammation [32]. Future advances in understanding the biology of TRMs and their role in dampening and/or exacerbating pro-inflammatory signaling and the downstream modulation of barrier integrity and inflammatory circuits could enhance disease control, decouple the skin-tropic inflammation and thereby reduce the systemic spillover of inflammatory skin diseases [33].

Lastly, an apparent compensatory increase in IL-4 and IL-4R detected by Olink analysis could not be confirmed by subsequent ELISA experiments. Thus, the Olink signal for these analytes should be interpreted with caution, as it may reflect a technical artifact.

The strengths of this study include the multi-omics approach. Including both transcriptomic and proteomic readouts in the same subjects and tissue ensures better translation of biochemical capture to biologic interpretation. Given that whole-blood samples do not capture the specificity of PBMCs, we compensated and sequenced to a median sequencing depth of 29.5 M reads per sample. Conversely, PBMCs’ biology relies on RNA transcription and subsequent protein expression, and given that cellular transcription is inherently stochastic [34], whole-blood sequencing in theory somewhat circumvents this cellular and biological behavior.

The study has several limitations, including a small sample size, limiting statistical power. The mixed treatment groups, although vastly dominated by dupilumab, could also impact the interpretation of the outcomes. Our cohort comprises mostly patients of European ancestry, possibly limiting the external validity of the study. EASI score dropouts due to missing data were statistically evaluated and found to be missing completely at random. Lastly, torque teno virus has multiple genotypes and tropisms in human biology, and the precise viral replication mechanism remains unclear, all of which could influence translation of torque teno virus measurements into clinical impact.

## 4. Materials and Methods

We collected plasma and whole blood (WB) from AD patients undergoing dupilumab (subcutaneous 600 mg/2 weeks loading dose, then 300 mg/2 weeks) or baricitinib (oral 4 mg/day) therapy at baseline (week 0, visit 1 (V1)), week 4 (V2) and week 16 (V3). The cohort comprised 41 patients (mean age 40 years (SD 17.9); 22 were males and 19 were females (Supplementary Table S5)). Thirty-four patients received dupilumab, 6 received baricitinib, and one received either dupilumab or baricitinib. All were included in downstream analysis.

Clinical treatment response was evaluated using the Eczema Area Severity Index (EASI), resulting in mean EASI: V1 = 14.46 (n = 32), V2 = 6.34 (n = 19) and V3 = 4.27 (n = 26). Using a linear mixed model, the mean EASI scores for V2 and V3 were compared to V1. V2 vs. V1: ΔEASI = −8.01 (SE 1.52), z = −5.26, *p* = <0.0005. V3 vs. V1: ΔEASI = −10.49 (SE 1.35), z = −7.74, *p* = <0.0005.

### 4.1. RNA Sequencing

Transcriptomic analysis was performed according to the protocols of Eurofins Genomics Europe Sequencing GmbH (Konstanz, Germany). RNA sequencing libraries were prepared using the NEBNext Ultra II Directional RNA Library Prep Kit (New England Biolabs, Ipswhich, MA, USA) and sequenced on an Illumina NovaSeq 6000 platform (San Diego, CA, USA), with a target depth of at least 20 million read pairs per sample. All sequenced samples passed QC (Supplementary Table S3). The median sequencing depth was 29.5 million read pairs per sample (range: 16.8 M–46.7 M). In total, 19.869 protein coding transcripts were mapped. The most highly expressed transcripts were hemoglobin, actin and mitochondrial genes. Hemoglobin (HBB, HBA2, HBA1) transcripts were excluded from downstream analysis. Expression values were floored at 0.01 and log2-transformed. Pearson correlation analysis was performed across all samples and revealed no obvious outliers.

### 4.2. Olink Proteomics

#### 4.2.1. Sample Preparation

Targeted proteomic profiling of 88 plasma samples was performed using Olink technology (Olink Proteomics, Uppsala, Sweden) targeting 368 proteins (laboratory provider BioXpedia, Aarhus, Denmark). The samples were randomized to minimize batch effects.

#### 4.2.2. Proximity Extension Assay and Incubation

We utilized the Olink Explore 384 Inflammation panel, which simultaneously measures 368 target proteins and 16 internal controls. The assay is based on Proximity Extension Assay (PEA) technology. Briefly, samples were incubated overnight at 4 °C with matching pairs of oligonucleotide-conjugated antibodies (probes) specific to the 368 target proteins. Concurrent binding of a probe pair to its target protein brings the attached oligonucleotides into close proximity.

#### 4.2.3. Extension, Amplification, and Sequencing

Following incubation, proximity-dependent DNA polymerization extended the hybridized oligonucleotides into unique, amplifiable double-stranded DNA barcodes. These barcodes underwent pre-amplification PCR, followed by a subsequent PCR step to incorporate sample-specific indices and sequencing adapters. The final prepared libraries were quantified and sequenced via Next-Generation Sequencing (NGS).

#### 4.2.4. Data Processing and Quality Control

Raw sequencing data were processed using version 3.9.0 of the Explore calculation module. Protein abundance was derived from the number of matched counts and reported as Normalized Protein eXpression (NPX) values on a log2 scale. The dataset underwent Intensity normalization; notably, data for the PNLIPRP2 assay were specifically plate-control normalized due to a natural bimodal distribution. Internal controls (Incubation, Extension, and Amplification) were utilized to monitor assay performance. All 88 samples (100%) passed quality control (Supplementary Table S4). Assays for BCL2L11, BID, and MGLL did not meet Olink’s batch release criteria and were excluded from the analysis. The assay demonstrated high precision, with average intra-assay and inter-assay coefficients of variance (%CV) both at 6%. Of the remaining evaluated proteins, 357 (98%) were successfully detected in >50% of the samples.

### 4.3. ELISA

ELISA was performed according to standard protocol using commercial kits from R&D Systems and Abcam.

### 4.4. PCR

Torque teno virus viral load was quantified using an in-house TaqMan real-time PCR assay targeting a conserved 63 bp sequence in the untranslated region of the torque teno virus genome (LightCycler 480 II, Roche, Basel, Switzerland). Results are reported as copies/mL and are log2-transformed.

### 4.5. Statistical Analysis

Statistical analysis for Torque Teno Virus data was performed in R (ver. 4.5.1) using the packages dplyr, tidyr, naniar and ggplot2. Paired *t* test was used to calculate statistical differences in torque teno virus concentrations. Little’s MCAR test [35] was applied to evaluate missing data in EASI scores. Heatmaps and clustering visualizations were generated in Qlucore Omics Explorer v. 3.11 (Qlucore, Lund, Sweden).

## 5. Conclusions

In conclusion, our findings support the interpretation that the predominant inflammatory processes in atopic dermatitis may largely be tissue-restricted and only modestly reflected in peripheral blood. Furthermore, the stable torque teno virus concentration observed during dupilumab and baricitinib therapy suggest a preserved immunocompetence, consistent with these targeted agents exerting immunomodulatory rather than immunocompromising effects in AD patients.

To advance precision medicine and develop curative therapeutic agents for AD, future advances and research in immunology and the underlying pathophysiology of AD could benefit from a focus on spatial multi-omics that directly target these compartmentalized skin-resident mechanisms.

## Data Availability

The raw data supporting the conclusions of this article will be made available by the authors on request.

## Supplementary Materials

The following supporting information can be downloaded at: https://www.mdpi.com/article/10.3390/ijms27188056/s1.

## Abbreviations

The following abbreviations are used in this manuscript:

AD	Atopic Dermatitis
TTV	Torque Teno Virus
IL	Interleukin
HIV	Human immunodeficiency virus
IL-4Rα	Interleukin 4 receptor subunit alpha
CCL17/TARC	CC chemokine ligand 17/thymic activation and regulated chemokine
CCL13	CC chemokine ligand 13
CCL26	CC chemokine ligand 26
JAK1/2	Janus kinase 1/2
WB	Whole blood
s.c.	Subcutaneous
NA	Not available
EASI	Eczema Area Severity Index
HBB, HBA2, HBA1	Hemoglobin
M	Million
DEG	Differential expressed gene
var	Variance
DLX2	Distal-less homeobox 2
DEP	Differential expressed protein
ELISA	Enzyme-linked immunosorbent assay
nd	Not Detectable
DC	Dendritic Cell
*FLG*	Filaggrin
CCR4	C-C motif chemokine receptor 4
TRM	Tissue resident memory T cell
PBMC	Peripheral blood mononuclear cell
PCR	Polymerase chain reaction
