# Supplementary material for: Peripheral Immune Modulation in Atopic Dermatitis During Dupilumab or Baricitinib Treatment Is Limited, as Assessed by Proteomic, Transcriptomic, and Torque Teno Virus Analyses
Source: Int J Mol Sci. 2026 Sep 10;27(18):8056. doi: 10.3390/ijms27188056 (PMC13607716; doi:10.3390/ijms27188056)
Supplement: Supplementary file 1 [file ijms-27-08056-s001.zip › ijms-4444078-supplementary.pdf]

V3 vs V1 Dupilumab RNASeq

Ensembl_ID	Gene	p-value	q-value	Difference
ENSG000001	DLX2	7.92E-06	0.06426362	-2.0401943
ENSG000001	FIBCD1	0.00079414	0.18856694	-1.5784553
ENSG000001	KRT79	0.00047799	0.18856694	-1.4229798
ENSG000001	FAM3D	0.01701382	0.27217086	-1.2244368
ENSG000001	TMCO2	0.01029466	0.24174018	-1.1948915
ENSG000001	SULT1C4	0.00712166	0.22673396	-1.1518511
ENSG000001	CCDC184	0.01664445	0.27031359	-1.1498932
ENSG000001	DRD5	0.00405978	0.21403539	-1.1457359
ENSG000001	SHROOM2	0.02908865	0.3136912	-1.1403583
ENSG000001	LRFN2	0.01939683	0.28170518	-1.1227245
ENSG000002	PMF1-BGLAP	0.00798199	0.23002982	-1.1088848
ENSG000001	HOXA5	0.00467398	0.21772133	-1.1050384
ENSG000001	OR6N1	0.00420734	0.21614597	-1.1036862
ENSG000001	CSDC2	0.01236264	0.25464329	-1.0837902
ENSG000001	FAM189A2	0.0050025	0.21833269	-1.0811061
ENSG000001	KCNN1	0.02284424	0.29500624	-1.0783967
ENSG000001	DLK1	0.03889631	0.33839374	-1.0639369
ENSG000001	ANKRD2	0.00310327	0.20994948	-1.0599952
ENSG000002	H3C1	0.04020494	0.34358292	-1.0508934
ENSG000001	DCLK3	0.04080116	0.34551949	-1.0487175
ENSG000001	CHST1	0.02523033	0.30100284	-1.044564
ENSG000002	C1orf167	0.02516749	0.30077106	-1.0401055
ENSG000001	PAQR5	0.03015142	0.31859418	-1.0207604
ENSG000001	TRIM50	0.04218561	0.34997262	-0.9949321
ENSG000002	C1orf210	0.01283866	0.25583328	-0.9947281
ENSG000001	C12orf56	0.04460629	0.35527495	-0.9869691
ENSG000001	AMPD1	0.01169732	0.24859874	-0.9815181
ENSG000001	SPP1	0.0001718	0.18011717	-0.971679
ENSG000002	N/A	0.0387445	0.33829709	-0.966984
ENSG000001	PTGIS	0.0241528	0.29927225	-0.9622295
ENSG000002	FAM72C	0.09237273	0.43743098	-0.9603088
ENSG000001	VHLL	0.02431251	0.29927225	-0.9567837
ENSG000001	ERICH5	0.04365667	0.35372007	-0.9516639
ENSG000001	PHF24	0.00140924	0.18910642	-0.9440973
ENSG000001	CCNP	0.03015823	0.31859418	-0.9424167
ENSG00000C	CPS1	0.0669504	0.39524277	-0.9357424
ENSG000001	NFATC4	0.02033741	0.28641584	-0.9311978
ENSG000001	SP6	0.07086716	0.40163003	-0.9271644
ENSG00000C	HHATL	0.02678982	0.30806392	-0.9215367
ENSG000002	HSFX4	0.05016214	0.36227834	-0.9214263
ENSG000001	CLDN10	0.10654914	0.45670098	-0.9093583

ENSG000001	ADCY1	0.04916436	0.360767	-0.9062293
ENSG00000C	CYP2W1	0.05051415	0.36271932	-0.8911223
ENSG000001	BEST3	0.05644681	0.3750908	-0.8909887
ENSG000001	N/A	0.09706967	0.44317188	-0.8896109
ENSG000001	ALOXE3	0.07498556	0.40825944	-0.8875206
ENSG000001	OLAH	0.03120361	0.32005877	-0.8812454
ENSG000001	CDH5	0.07239923	0.40374402	-0.8794491
ENSG000002	FBXO17	0.06170588	0.3855015	-0.8786761
ENSG000001	PCSK9	0.00320249	0.21134435	-0.8778979
ENSG000001	DKKL1	0.01949592	0.28172722	-0.8770047
ENSG000001	TMEM198	0.07674286	0.41097419	-0.8698344
ENSG000001	NAALADL2	0.01155546	0.24687632	-0.8636623
ENSG000001	KCNE2	0.07678799	0.41105	-0.861367
ENSG000001	C9orf131	0.02124177	0.28959078	-0.8611363
ENSG000001	KRT74	0.09652622	0.44251066	-0.8610353
ENSG000001	ANKRD31	0.07294117	0.40435477	-0.8549092
ENSG000002	CTAGE4	0.06093043	0.38464604	-0.8478885
ENSG00000C	MPP4	0.04742271	0.35865951	-0.8478151
ENSG000001	ECM2	0.06587965	0.39326762	-0.8458132
ENSG000001	NWD1	0.02246908	0.29398097	-0.8455129
ENSG000001	RSPH14	0.00058219	0.18856694	-0.8450028
ENSG000001	MYOZ3	0.02788957	0.31135087	-0.8367086
ENSG000002	FER1L5	0.01986822	0.28348004	-0.8349145
ENSG000001	RIIAD1	0.06960908	0.40004644	-0.8293009
ENSG000001	NOS1AP	0.07104318	0.40194888	-0.8233809
ENSG000001	CSMD2	0.00568535	0.21875144	-0.8218485
ENSG000001	CYP11A1	0.08258157	0.4220168	-0.8211061
ENSG000001	KCNJ5	0.01260018	0.25512421	-0.8200087
ENSG000002	N/A	0.04294011	0.35185871	-0.8181957
ENSG00000C	EPN3	0.03967137	0.34099737	-0.8174847
ENSG000001	CCDC27	0.0569819	0.37656295	-0.8173423
ENSG000001	ESRP1	0.04500867	0.35545023	-0.81496
ENSG000002	RHOXF2B	0.05143075	0.36502002	-0.8148726
ENSG000001	CILP2	0.05621537	0.37502119	-0.8125838
ENSG000001	EMX1	0.22073074	0.5880238	-0.8124726
ENSG000001	MAOA	0.07293166	0.40435477	-0.8119527
ENSG000001	PTPRD	0.10434037	0.45468989	-0.8068388
ENSG000002	PNMA6A	0.00024121	0.18011717	-0.8036702
ENSG000001	MEIS3	0.08311109	0.42250308	-0.8028855
ENSG000001	WFDC1	0.03321218	0.3238836	-0.8018225
ENSG000002	N/A	0.10989847	0.46255597	-0.7961707
ENSG000001	SFRP1	0.05283763	0.36963577	-0.7901632
ENSG000001	BMERB1	0.08782097	0.43050213	-0.7829434

ENSG000001	ARHGEF16	0.09503769	0.44064162	-0.7827291
ENSG000001	CDH2	0.00653566	0.22296216	-0.7784863
ENSG000001	PACRG	0.08046712	0.41715984	-0.7764258
ENSG00000(	TPSD1	0.10751452	0.45856726	-0.7741524
ENSG000001	KCNK17	0.01169286	0.24859874	-0.7735017
ENSG00000(	PTPN21	0.1294961	0.4871968	-0.7694138
ENSG000002	TBCEL-TECT/	0.11846275	0.47516823	-0.7675196
ENSG000002	N/A	0.14624129	0.50446567	-0.7651274
ENSG000001	SERPINF2	0.05171779	0.36546885	-0.7625309
ENSG000002	SCO2	0.17212359	0.53630279	-0.7617177
ENSG000002	TMDD1	0.07779485	0.41335649	-0.7615097
ENSG000002	GAGE10	0.17765022	0.5423686	-0.7585998
ENSG000001	SLC12A5	0.05915223	0.38128415	-0.7584742
ENSG000001	FNDC7	0.07020716	0.40066693	-0.758428
ENSG000001	PGPEP1L	0.0542494	0.37256839	-0.7576531
ENSG000002	TXNDC8	0.09632124	0.44215472	-0.7548971
ENSG000001	OVOL3	0.11139168	0.46472866	-0.7529297
ENSG000001	MAGED4B	0.17687338	0.54177807	-0.7519073
ENSG000001	CXCL12	0.02178982	0.29183808	-0.7489075
ENSG000001	HTR1B	0.13267362	0.49138265	-0.7484214
ENSG00000(	ELN	0.08063317	0.41748748	-0.7451988
ENSG000002	SPINK8	0.02178181	0.29183808	-0.7380592
ENSG000001	SNCG	0.10326464	0.45287405	-0.7302969
ENSG000002	PHGR1	0.10226466	0.45160695	-0.7297573
ENSG000002	PRR5-ARHG/	0.08735654	0.42952109	-0.7291082
ENSG000001	MIOX	0.09518426	0.44084511	-0.7281615
ENSG000001	TGM3	0.00185016	0.19438665	-0.7264838
ENSG000001	IGSF10	0.07751544	0.41275686	-0.722613
ENSG000001	TMPRSS4	0.14811775	0.50716742	-0.7225857
ENSG000001	ZNF385B	0.12427152	0.48162293	-0.7215346
ENSG000001	SHANK1	0.07056364	0.40156223	-0.7211579
ENSG000001	NDST3	0.05629514	0.37502119	-0.7210259
ENSG000002	ARHGEF33	0.07248698	0.40400469	-0.7199511
ENSG000001	PBK	0.10657388	0.45670098	-0.7174839
ENSG00000(	CDH17	0.11511113	0.47079584	-0.7173886
ENSG000001	CDH6	0.07971997	0.4152849	-0.7148508
ENSG000001	MAFA	0.01205841	0.25198499	-0.7130827
ENSG000001	MMP9	0.00360324	0.21208655	-0.7110346
ENSG000001	IRGC	0.1837963	0.54888735	-0.709976
ENSG000001	VARS2	0.29267562	0.64955906	-0.708514
ENSG000002	RGL3	0.00026965	0.18856694	-0.7070629
ENSG000001	MAP6	0.10069388	0.44879676	-0.7028306
ENSG00000(	PITX1	0.07071128	0.40156223	-0.7027158

ENSG000001	BCAM	0.02357338	0.29875884	-0.7006075
ENSG000001	GPR17	0.10186255	0.45128027	-0.7003342
ENSG00000C	OSBPL6	0.00100292	0.18856694	-0.6986192
ENSG000002	CNTNAP3C	0.06718987	0.39561184	-0.6983424
ENSG000001	FOXQ1	0.06077733	0.38464604	-0.6977872
ENSG000001	HSPA4L	0.06819211	0.39743754	-0.6968812
ENSG000001	TPSB2	0.00898435	0.23513527	-0.6964861
ENSG000001	RHOV	0.18994476	0.55472021	-0.6937692
ENSG000001	DEUP1	0.10367476	0.45355618	-0.6912438
ENSG000001	CRB1	0.19453911	0.56193152	-0.6909665
ENSG000001	CAPN13	0.11780567	0.4744792	-0.6880468
ENSG000001	HEY1	0.02858863	0.31345545	-0.6866342
ENSG000001	SLC28A1	0.10425955	0.45468989	-0.6855581
ENSG000001	AOX1	0.19882229	0.56598606	-0.6852548
ENSG000001	SHISA7	0.00708301	0.22670251	-0.6848996
ENSG000001	STARD13	0.04004406	0.34274931	-0.6829742
ENSG000001	GNB3	0.06602608	0.39343668	-0.6825085
ENSG000001	ARC	0.12620393	0.48388732	-0.679882
ENSG00000C	NOTCH3	0.07657366	0.41092575	-0.6788707
ENSG000001	CHRND	0.17675421	0.54166428	-0.6767496
ENSG000001	SRMS	0.1285847	0.48678709	-0.6757518
ENSG00000C	CFTR	0.02357988	0.29875884	-0.6755031
ENSG00000C	RHBDF1	0.2434883	0.60935874	-0.6739131
ENSG000001	GABRD	0.14338049	0.50145476	-0.6727126
ENSG000001	PLPPR4	0.1406624	0.50051652	-0.670619
ENSG000001	KRT13	0.0532905	0.36981204	-0.6705638
ENSG000001	SLC7A10	0.22207531	0.58938163	-0.6699573
ENSG000002	IFITM10	0.09083901	0.4347065	-0.667837
ENSG000001	PPARGC1A	0.18001688	0.54501079	-0.6663777
ENSG000001	FAM71F1	0.09032787	0.43397794	-0.6639482
ENSG000001	FREM1	0.20496396	0.56891466	-0.6639168
ENSG000001	MYRIP	0.20498726	0.56891466	-0.6608396
ENSG000001	MARCHF4	0.06055357	0.38436603	-0.6605048
ENSG000001	TNFSF9	0.10214426	0.45160695	-0.657936
ENSG000002	C13orf42	0.12103512	0.47724625	-0.6575785
ENSG000001	CACNA1B	0.23408065	0.60173104	-0.6555371
ENSG000001	KASH5	0.11788194	0.4744792	-0.6542501
ENSG000001	C20orf203	0.16581877	0.52885285	-0.654137
ENSG000002	KRTAP5-7	0.12557132	0.48358024	-0.6541232
ENSG000001	SMIM10L2B	0.1899417	0.55472021	-0.6532722
ENSG000001	GALNT14	0.01194818	0.25192551	-0.6530237
ENSG000002	SPATA31A1	0.15765493	0.52077869	-0.6521363
ENSG000001	THAP10	0.12142969	0.47774505	-0.6516099

ENSG000002	ACTN3	0.16272393	0.52590534	-0.6515073
ENSG000002	KRT81	0.15703217	0.5199629	-0.6505885
ENSG000001	CYP7A1	0.18218451	0.54662721	-0.6490379
ENSG000001	ITGB4	0.00830848	0.23282737	-0.648724
ENSG000001	SDC2	0.00884682	0.23464493	-0.6479613
ENSG000001	SVOPL	0.16052367	0.52348321	-0.6473444
ENSG000001	FAM171A2	0.09407079	0.43929464	-0.6462936
ENSG00000C	ENTPD2	0.01576802	0.26642326	-0.6423081
ENSG000002	RXRB	0.25000447	0.61588873	-0.6415651
ENSG000001	NRXN1	0.08904711	0.4331765	-0.6415469
ENSG000001	ABCA9	0.11423453	0.46971445	-0.6397581
ENSG00000C	INSRR	0.19143963	0.5568623	-0.6394238
ENSG000002	RASL10B	0.05269462	0.36895324	-0.6388457
ENSG00000C	BEST2	0.08282505	0.42228867	-0.6386766
ENSG000001	KRT77	0.02001366	0.28415764	-0.6385939
ENSG000002	DPEP2NB	0.1767251	0.54166428	-0.6384009
ENSG000001	TWIST1	0.20459103	0.5688754	-0.636911
ENSG000001	SOX11	0.14532539	0.50413578	-0.636326
ENSG00000C	FMO3	0.03160445	0.3219332	-0.6360134
ENSG000001	RHBG	0.11318251	0.46821512	-0.6353927
ENSG000001	MYBPC1	0.20167898	0.56773237	-0.635006
ENSG000001	RD3	0.04466015	0.35527495	-0.6346213
ENSG000001	REEP2	0.01584467	0.26687746	-0.6331195
ENSG000001	IGLL1	0.1517987	0.51292686	-0.6329484
ENSG000001	HOXB9	0.20147515	0.56773237	-0.6321223
ENSG000002	N/A	0.11460738	0.47026153	-0.6320764
ENSG000001	DLL4	0.087054	0.42907415	-0.6319649
ENSG000001	ODF3	0.06141443	0.38542193	-0.630566
ENSG000001	FAM149A	0.08996445	0.43377006	-0.6300706
ENSG000001	GPR173	0.15885276	0.52170151	-0.6299122
ENSG000001	NECAB2	0.00266378	0.2093801	-0.629582
ENSG000001	GHR	0.09710697	0.44317188	-0.6294686
ENSG000001	SLC2A4	0.11219312	0.46614115	-0.6293226
ENSG000001	COL7A1	4.85E-06	0.06426362	-0.6287647
ENSG000001	PCOLCE2	0.0969213	0.44311879	-0.6284648
ENSG000001	GALNT9	0.10593456	0.4561473	-0.6254092
ENSG00000C	TRPM3	0.14175966	0.50055578	-0.6253298
ENSG000002	FAM166B	0.11982926	0.47604098	-0.6249582
ENSG000001	DKK4	0.06850683	0.39796501	-0.624685
ENSG000001	ARFGEF3	0.08527897	0.42539927	-0.624678
ENSG000001	SHANK2	0.14534171	0.50413578	-0.6242811
ENSG00000C	EML1	0.06095496	0.38464604	-0.6227983
ENSG000001	EPCAM	0.09179946	0.43634305	-0.6222845

ENSG000001	PRR36	0.18578298	0.55052683	-0.6219583
ENSG000001	RIBC2	0.06651585	0.39445505	-0.621351
ENSG000001	CLSTN2	0.02784778	0.31135087	-0.6200432
ENSG000001	MOBP	0.11487033	0.47052227	-0.6197303
ENSG000001	CNGA4	0.03130266	0.32055029	-0.6152061
ENSG000002	CBY3	0.12179113	0.47854428	-0.6144981
ENSG000001	COX6B2	0.14173002	0.50055578	-0.6142207
ENSG000001	HPN	0.20437752	0.5688169	-0.6132825
ENSG000001	POU4F1	0.26682071	0.62929976	-0.6131949
ENSG000001	COL1A2	0.15477803	0.51610961	-0.6130134
ENSG00000C	CEACAM7	0.11815561	0.4748744	-0.6120843
ENSG000001	TMEM54	0.22466987	0.59200912	-0.6117489
ENSG00000C	TRPC5	0.23231101	0.5995921	-0.6101636
ENSG000001	IL17B	0.01132953	0.24556914	-0.6099339
ENSG000002	TTC23L	0.25340659	0.61912837	-0.6099314
ENSG000001	RGS7	0.1043711	0.4547018	-0.6094689
ENSG000001	PLAU	0.00188783	0.19438665	-0.6088501
ENSG000001	H2AC20	0.0349694	0.32915835	-0.606501
ENSG000001	WSCD1	0.2258678	0.59319591	-0.6059334
ENSG000002	CBSL	0.14232478	0.50068215	-0.6045776
ENSG00000C	ABCB11	0.12934116	0.4871968	-0.6043522
ENSG000001	PCLAF	0.07402861	0.40580777	-0.6030856
ENSG000001	ODAD2	0.15208397	0.51296214	-0.6022534
ENSG000001	CALHM3	0.19446909	0.56193152	-0.6017609
ENSG000001	ANGPT4	0.02987585	0.31859418	-0.6007628
ENSG000001	PLPPR3	0.20962798	0.5736945	-0.600635
ENSG000001	TGM4	0.20515805	0.56901717	-0.5997241
ENSG000002	KLHL41	0.23806936	0.60417821	-0.5991092
ENSG00000C	PGC	0.2206575	0.5880238	-0.5990893
ENSG000001	OR2AT4	0.08519286	0.42526981	-0.5982323
ENSG00000C	DKK3	0.07469707	0.40750684	-0.5971934
ENSG000002	TMEM253	0.14534638	0.50413578	-0.5971903
ENSG000001	RNF150	0.15903284	0.5217239	-0.5957252
ENSG000001	XKR3	0.05300169	0.36981204	-0.5921353
ENSG000001	PIH1D2	0.24368219	0.60937379	-0.5917665
ENSG000001	UNC5C	0.23194836	0.599371	-0.5912438
ENSG000001	SCGB1A1	0.2659469	0.62873905	-0.5908256
ENSG000001	FAM107A	0.15469244	0.51606937	-0.5901167
ENSG000001	TXNDC2	0.14374999	0.50194033	-0.5887673
ENSG000001	NCS1	0.01914814	0.28080056	-0.5883266
ENSG000002	DEFA3	0.00949447	0.23577638	-0.5882812
ENSG000001	GJB7	0.28276199	0.64078247	-0.5875888
ENSG000001	CDH22	0.16211535	0.52484247	-0.5874571

ENSG000001	TERT	0.16372362	0.52709285	-0.5871499
ENSG00000(	ZMYND12	0.12236308	0.47887061	-0.5859733
ENSG000002	PAGE2B	0.16365133	0.52709285	-0.5827363
ENSG000001	BMPER	0.20609139	0.56956674	-0.582369
ENSG000002	NPIPA7	0.1226952	0.4792403	-0.5806078
ENSG000001	N/A	0.29845642	0.65398608	-0.5805566
ENSG00000(	COL16A1	0.25771228	0.62185678	-0.5804688
ENSG000001	CTXN1	0.20032781	0.56713365	-0.5802803
ENSG000001	S100A5	0.00597525	0.22079879	-0.5792792
ENSG000001	COL5A1	0.14409239	0.50241838	-0.579165
ENSG000001	CALML6	0.08818087	0.43100324	-0.575156
ENSG000002	N/A	0.24136334	0.60721888	-0.5747978
ENSG000001	LPIN3	0.04904952	0.360767	-0.5738492
ENSG000001	DNAH10	0.1958728	0.5634849	-0.5736913
ENSG00000(	CDH3	0.29179213	0.64936748	-0.5731954
ENSG000001	NPTX2	0.20384447	0.5688169	-0.5721988
ENSG000001	INSC	0.08028114	0.41684053	-0.5709438
ENSG000001	DAAM2	0.02771518	0.3113392	-0.5704746
ENSG000002	ZNF492	0.15633952	0.5190114	-0.5698974
ENSG000001	ANKRD1	0.20047026	0.56724227	-0.5691209
ENSG000001	FAM181B	0.18853119	0.55295897	-0.5683947
ENSG000001	RSPO4	0.2365363	0.60364584	-0.567421
ENSG000001	N/A	0.13002672	0.48787839	-0.5671818
ENSG00000(	ERBB3	0.16986574	0.53437545	-0.5670035
ENSG000002	MMP28	0.18708899	0.55108473	-0.5661125
ENSG000001	UPK2	0.20060103	0.56730451	-0.5658512
ENSG00000(	CPXM1	0.01257345	0.25512421	-0.5648175
ENSG000001	TGM5	0.27977738	0.63864899	-0.5647683
ENSG000001	RGPD4	0.19236018	0.55820972	-0.5645168
ENSG000001	RND1	0.21725437	0.5839363	-0.5642943
ENSG000002	PGA3	0.24235005	0.60796025	-0.5634417
ENSG000001	SMTNL2	0.17505375	0.53975461	-0.5633103
ENSG000001	CBS	0.00309186	0.20994948	-0.5616317
ENSG000001	KIF18B	0.03199609	0.32288405	-0.5600272
ENSG000001	IL19	0.28638121	0.64387022	-0.5599652
ENSG000001	DMRTC2	0.00608655	0.22079879	-0.5579919
ENSG000002	POU5F1	0.14950819	0.50925123	-0.5579128
ENSG000002	PRRT4	0.00757714	0.22933195	-0.5565794
ENSG000001	PDE6H	0.19991868	0.56670385	-0.5560556
ENSG000001	DZIP1	0.34468504	0.68522517	-0.5554073
ENSG000002	N/A	0.02715422	0.30967858	-0.555176
ENSG000002	N/A	0.24420132	0.61002532	-0.5539277
ENSG000001	GOLT1A	0.23770352	0.60402516	-0.5538179

ENSG000001	ACTRT3	0.2894948	0.64727721	-0.5518758
ENSG00000(	ANO2	0.35519729	0.6919142	-0.5500319
ENSG000001	RADIL	0.10541926	0.45584355	-0.5499808
ENSG000001	LYPD3	0.0592551	0.38150633	-0.5498224
ENSG000001	HMOX2	0.21729101	0.58393813	-0.5495632
ENSG000001	VSTM2L	0.05626933	0.37502119	-0.5488805
ENSG000001	FA2H	0.18284694	0.54726006	-0.5484139
ENSG000002	TEX46	0.28287052	0.64084947	-0.5468238
ENSG000001	ARG1	0.01065014	0.24206404	-0.5462131
ENSG000001	INHBB	0.0069725	0.22577227	-0.5460943
ENSG000001	HBZ	0.06669271	0.39496052	-0.5458415
ENSG000001	RHBDL2	0.23086622	0.59855451	-0.5456393
ENSG000002	MLLT6	0.25386276	0.61921212	-0.5450759
ENSG000002	CD177	0.01812391	0.27631728	-0.5450367
ENSG000001	RPRML	0.29862122	0.65406944	-0.5448946
ENSG000001	SEMA6B	0.02329903	0.2981138	-0.5442507
ENSG000001	RASSF10	0.09556876	0.4413663	-0.5438552
ENSG000001	SCN11A	0.29464341	0.65098879	-0.5433462
ENSG000001	MLNR	0.08501812	0.42526981	-0.5424372
ENSG00000(	MOCOS	0.14104773	0.50055066	-0.5418273
ENSG000001	CCBE1	0.113528	0.46882009	-0.541424
ENSG000001	LEP	0.01730161	0.27265457	-0.5412756
ENSG000001	TMC7	0.08961458	0.43361062	-0.5412273
ENSG000001	SDC1	0.36480688	0.69785218	-0.5385569
ENSG00000(	DSG2	0.19559606	0.5632015	-0.5385491
ENSG000002	PRH1	0.28122862	0.63941315	-0.5369365
ENSG000001	FAM47E	0.25050718	0.616379	-0.5340807
ENSG000001	FAM86B2	0.17792097	0.54282279	-0.5339338
ENSG000001	CDC25A	0.01340286	0.25828987	-0.5332977
ENSG000002	CCL23	0.26013115	0.6235237	-0.5332388
ENSG000001	SLC25A2	0.07430427	0.40673667	-0.53272
ENSG000001	LRIT3	0.09151368	0.43611772	-0.5326017
ENSG000001	RBFOX3	0.10328106	0.45287405	-0.5321776
ENSG000002	HPR	0.16512633	0.5286578	-0.531604
ENSG00000(	PTPN3	0.09317867	0.43758758	-0.5305961
ENSG000001	GSG1	0.06111573	0.38477554	-0.5303394
ENSG000001	ZBTB7C	0.15697632	0.5199629	-0.5301099
ENSG000001	IRS2	0.00716727	0.22727528	-0.5280969
ENSG000001	UTF1	0.03782442	0.33525901	-0.5267828
ENSG000001	AK7	0.3280279	0.67237631	-0.5237087
ENSG00000(	PANX2	0.00112577	0.18856694	-0.5235137
ENSG000001	NKAIN4	0.18234992	0.54678908	-0.5234846
ENSG000001	GALNT5	0.13950536	0.49975817	-0.5229585

ENSG000001	BMP10	0.21846332	0.58528507	-0.5224509
ENSG000001	CCDC40	0.08740147	0.42952109	-0.5223764
ENSG000001	CHD5	0.07579324	0.41015711	-0.5223038
ENSG000002	DEFA1	0.19247121	0.55826279	-0.5220155
ENSG000001	DUSP8	0.20244769	0.56807486	-0.521569
ENSG000001	OR13D1	0.18817288	0.55220732	-0.5210767
ENSG000001	OR2L3	0.10205216	0.45141805	-0.5209473
ENSG000002	N/A	0.23263813	0.59995954	-0.5204917
ENSG00000(	PRSS3	0.28357706	0.64182335	-0.520322
ENSG000002	N/A	0.13784383	0.49881219	-0.5198717
ENSG000001	CGNL1	0.21347847	0.57911862	-0.519474
ENSG00000(	CA12	0.10781247	0.45898034	-0.5192783
ENSG000001	C10orf82	0.17303532	0.53759345	-0.5180884
ENSG000001	LPO	0.31489312	0.662749	-0.5175223
ENSG000001	UCHL1	0.37017399	0.70302695	-0.5171902
ENSG00000(	BAIAP3	0.0021004	0.19943931	-0.5171425
ENSG000001	EPO	0.23730081	0.60402516	-0.5164035
ENSG000002	ANKRD20A1	0.18703807	0.55108473	-0.5148703
ENSG000001	CA4	0.00552656	0.21833269	-0.5140969
ENSG000002	PTPN20	0.33329291	0.67569946	-0.5133941
ENSG000001	SFN	0.02203923	0.29231997	-0.5130298
ENSG000001	KCNH6	0.09908452	0.4460792	-0.5124174
ENSG000001	ALDH3B2	0.22341366	0.59081204	-0.5121216
ENSG000001	TICRR	0.04860264	0.360767	-0.5118943
ENSG000001	GALR2	0.30301252	0.65591444	-0.5118688
ENSG000001	WNK2	0.00191893	0.19438665	-0.511743
ENSG000001	BSND	0.1949623	0.56257381	-0.5116307
ENSG00000(	SYN1	0.01342047	0.25828987	-0.5112692
ENSG000001	MANSC1	0.001644	0.19292382	-0.5105419
ENSG000002	NR2E3	0.36912483	0.70197716	-0.5086094
ENSG000001	LMOD1	0.09299551	0.43758758	-0.5080841
ENSG000002	ADM5	0.10345489	0.45326416	-0.5075884
ENSG000001	SYT9	0.30949626	0.65916845	-0.5075533
ENSG000001	DAND5	0.20371751	0.56880022	-0.5073559
ENSG000001	HTR2A	0.29212152	0.64944907	-0.5051123
ENSG000001	TRIM63	0.05622749	0.37502119	-0.5050877
ENSG000001	ANPEP	0.00090623	0.18856694	-0.5030509
ENSG000001	SCUBE1	0.19969733	0.56647197	-0.5029026
ENSG000001	OR52N2	0.20380092	0.5688169	-0.5025755
ENSG000001	ITLN1	0.02429244	0.29927225	-0.5013564
ENSG000001	SYNGR3	0.01944827	0.28172722	-0.5011794
ENSG000001	GPR4	0.28102514	0.63941315	-0.5010874
ENSG000001	LMAN1L	0.1960395	0.56367864	-0.5010692

ENSG000001	TAC3	0.20178244	0.56773237	-0.5003401
ENSG000002	SOX18	0.28821024	0.64547167	-0.4998144
ENSG000001	KRT2	0.18365546	0.54871392	-0.4996383
ENSG000001	GDF15	0.00538666	0.21833269	-0.4994456
ENSG000001	ARSL	0.06356231	0.38981851	-0.4982071
ENSG000001	RSPH6A	0.20253651	0.56807486	-0.4979684
ENSG000001	PDZD3	0.00619579	0.22079879	-0.4969856
ENSG000002	H2BE1	0.06121939	0.38491421	-0.4967625
ENSG000001	KRT80	0.29114826	0.64879427	-0.496735
ENSG000001	PGF	0.12340388	0.47994951	-0.4964138
ENSG000001	EPHB3	0.09564323	0.4413663	-0.4963082
ENSG000001	CCDC87	0.12950022	0.4871968	-0.4956238
ENSG000001	GPNMB	0.02719664	0.30967858	-0.4947742
ENSG000002	TAS2R38	0.2341774	0.60173104	-0.4943765
ENSG000002	FOXD4L6	0.10368914	0.45355618	-0.493528
ENSG000001	RNF222	0.04322084	0.35247453	-0.4934799
ENSG000002	OR6K3	0.37522237	0.70720386	-0.4916298
ENSG000001	ELAPOR1	0.00174534	0.19438665	-0.4911371
ENSG000001	WFIKKN2	0.40460921	0.72672584	-0.4904668
ENSG000001	RAB3C	0.1848323	0.5498217	-0.4901571
ENSG000001	DQX1	0.29265046	0.64955906	-0.4899886
ENSG000001	YIPF7	0.2336266	0.60136256	-0.4899839
ENSG000001	PDC	0.26735305	0.62949703	-0.4898676
ENSG000001	BEND7	0.00920066	0.23551879	-0.4897668
ENSG000001	APOD	0.27525433	0.63499184	-0.4889219
ENSG000001	FAM227A	0.3297319	0.67344111	-0.4879105
ENSG000001	CHST6	0.2322633	0.59956425	-0.4876463
ENSG000002	OR52I2	0.26071058	0.62417543	-0.4875004
ENSG000001	DPEP3	0.00269713	0.2093801	-0.4874026
ENSG000002	OMP	0.29952173	0.6542516	-0.484534
ENSG000002	EHMT2	0.3624859	0.69636577	-0.4838414
ENSG000001	OR56B4	0.36600666	0.69846943	-0.4836251
ENSG000002	FDX2	0.23693622	0.60366129	-0.483448
ENSG000001	FAM78B	0.29749748	0.65276574	-0.483141
ENSG000001	RAB3IL1	0.02705229	0.3093296	-0.4813409
ENSG000001	TRIM54	0.22328521	0.59081204	-0.4813212
ENSG000001	CPNE9	0.17644809	0.54126676	-0.4809047
ENSG000001	UGT2B17	0.11031562	0.46284103	-0.4797304
ENSG000001	PIMREG	0.11374641	0.46915085	-0.4795575
ENSG000001	CTSE	0.00774453	0.23002982	-0.4789936
ENSG000001	ST8SIA5	0.40044385	0.72583241	-0.4788035
ENSG000001	FAM186A	0.13973653	0.49975817	-0.4781792
ENSG000001	SOAT2	0.10110973	0.44917064	-0.4774255

ENSG00000(	TP53INP2	0.00076851	0.18856694	-0.476486
ENSG000002	N/A	0.17060752	0.53457173	-0.4761633
ENSG000002	MALRD1	0.24893589	0.61478169	-0.4759743
ENSG000001	PROC	0.01151772	0.24639427	-0.4755171
ENSG000001	PGBD5	0.19922844	0.56600138	-0.47549
ENSG000001	GRK1	0.05757062	0.37707713	-0.4753309
ENSG000002	PGA5	0.25095047	0.61681543	-0.4752052
ENSG000001	ACER1	0.07367523	0.40523875	-0.4750997
ENSG000002	SYS1-DBNDI	0.30265223	0.65591444	-0.4750092
ENSG000002	TRNP1	0.03607245	0.33117113	-0.4749295
ENSG000002	DUXB	0.22242024	0.58995895	-0.4748574
ENSG000001	C1QTNF12	8.28E-05	0.18011717	-0.4741962
ENSG000001	C7orf57	0.27008914	0.63142427	-0.4740415
ENSG000001	ZNF730	0.3770651	0.7086118	-0.4737208
ENSG000001	HAP1	0.31956509	0.66652314	-0.4731013
ENSG000002	ANXA8L1	0.32684037	0.67217574	-0.4728605
ENSG000001	CPLX1	0.14183764	0.50055578	-0.47277
ENSG000001	GPC3	0.20571047	0.56950057	-0.4726148
ENSG000001	GTSF1L	0.23321738	0.60069012	-0.4724083
ENSG000002	IGSF23	0.32113902	0.66766831	-0.4722834
ENSG00000(	NPC1L1	0.29743477	0.65274757	-0.4698935
ENSG000002	CD200R1L	0.23983171	0.60656502	-0.4696527
ENSG000001	KLF1	0.01882503	0.27889987	-0.4688935
ENSG000001	KLHDC8A	0.0686286	0.39796501	-0.4679884
ENSG000001	RLN3	0.29132163	0.64903806	-0.4670406
ENSG000001	KCTD4	0.29758444	0.65286833	-0.4666542
ENSG00000(	RPH3A	0.04107413	0.34663236	-0.466511
ENSG000001	HTRA3	0.17014271	0.53437545	-0.4664418
ENSG000002	ARHGAP19-S	0.19343016	0.56004378	-0.4659688
ENSG000001	CMTM2	0.00056558	0.18856694	-0.4655594
ENSG000001	GREB1	0.28910595	0.6469423	-0.465556
ENSG000001	RAB36	0.00403173	0.21403539	-0.4655469
ENSG000001	FOXE3	0.13347509	0.49240713	-0.4651304
ENSG000001	NYAP1	0.04520678	0.35545023	-0.4644843
ENSG000001	NEIL3	0.27317932	0.6338147	-0.4641611
ENSG000002	N/A	0.19136433	0.55685897	-0.4639731
ENSG000001	MAS1	0.20139462	0.56773237	-0.4637396
ENSG000002	PCDHGB2	0.27631906	0.6360577	-0.4631526
ENSG000001	HUNK	0.29239729	0.64947398	-0.4630454
ENSG000001	DEFA4	0.12786223	0.48588488	-0.4630149
ENSG000001	NR4A3	0.19977745	0.56650131	-0.4629093
ENSG000001	SGIP1	0.06464516	0.39055819	-0.4611799
ENSG000001	DIAPH3	0.21154472	0.57620716	-0.4610261

ENSG000001	NPHS1	0.32619999	0.67170284	-0.4605133
ENSG000001	PADI4	0.00098651	0.18856694	-0.4603959
ENSG000001	CFAP91	0.35850084	0.69382497	-0.4601014
ENSG000001	SPATA46	0.3290968	0.67287758	-0.4598388
ENSG000001	CIDEB	0.16303008	0.52653704	-0.4597698
ENSG000002	N/A	0.22617361	0.59329927	-0.4596473
ENSG000001	AATK	0.00065764	0.18856694	-0.4596255
ENSG000002	RNF103-CHI	0.25341594	0.61912837	-0.4593472
ENSG000001	DYRK3	0.00942106	0.23551879	-0.4587846
ENSG000001	CHST5	0.21161072	0.57620716	-0.4586506
ENSG000002	ACKR1	0.05110722	0.36427913	-0.4576924
ENSG000001	COLEC12	0.10837518	0.45963571	-0.4576682
ENSG000001	BDH1	0.36056838	0.69535995	-0.456075
ENSG000001	KAZN	0.00689024	0.22420199	-0.4559128
ENSG000002	N/A	0.02930935	0.31537176	-0.4557121
ENSG000001	IQGAP3	0.16165293	0.52427832	-0.4556757
ENSG000002	SLC22A31	0.12818768	0.48619094	-0.4556191
ENSG000001	RHOD	0.35522955	0.6919142	-0.4555119
ENSG000001	CCN3	0.0026913	0.2093801	-0.4554268
ENSG000001	SH3RF3	0.00608733	0.22079879	-0.4548173
ENSG000002	MROH6	0.0030884	0.20994948	-0.454663
ENSG000002	SCX	0.02216735	0.29306353	-0.4523421
ENSG000001	SCGB3A1	0.23782324	0.60402516	-0.4522999
ENSG000001	TMEM125	0.30663392	0.65735608	-0.4517955
ENSG000001	FFAR4	0.06336106	0.38950527	-0.450575
ENSG000001	EPHB4	0.00298995	0.20994948	-0.450266
ENSG000001	OTOP2	0.38116219	0.71039147	-0.4500299
ENSG000001	FABP6	0.01343964	0.25828987	-0.4495502
ENSG000001	PLEK2	0.02377557	0.29875884	-0.4493288
ENSG000001	SFRP2	0.0610713	0.38464604	-0.4491265
ENSG000002	ANKRD18B	0.27457675	0.63464829	-0.4490087
ENSG000001	KCNG2	0.06987419	0.4002892	-0.448899
ENSG000001	MMRN2	0.16536999	0.52878404	-0.4485095
ENSG000001	LOXL4	0.14919798	0.5092072	-0.4476657
ENSG000001	ALPL	0.01365918	0.25910565	-0.4475686
ENSG000001	CLBA1	0.00057262	0.18856694	-0.4475159
ENSG000001	ABCB6	0.06484564	0.39068596	-0.4474229
ENSG000001	IL1R2	0.00130727	0.18856694	-0.4466901
ENSG000001	CMBL	0.04197514	0.34933386	-0.4450013
ENSG000001	ENPP1	0.15228065	0.51327042	-0.444696
ENSG000002	CCHCR1	0.1087992	0.46040635	-0.4440624
ENSG000001	PLN	0.38026882	0.70987972	-0.4440241
ENSG000001	SLC52A3	0.15313937	0.5142759	-0.4428581

ENSG000001	SYT8	0.13819731	0.49898885	-0.4421767
ENSG000001	SPC24	0.01956876	0.28172722	-0.4418028
ENSG000002	OOSP3	0.16487386	0.52822746	-0.4411832
ENSG000001	AICDA	0.07596482	0.41032627	-0.4406279
ENSG000001	DTL	0.04902746	0.360767	-0.4398347
ENSG000001	KIAA0319	0.05507337	0.37354464	-0.4398297
ENSG000002	GYPB	0.0719179	0.40315841	-0.4391731
ENSG000001	NTRK1	0.14214222	0.50068215	-0.4384522
ENSG000001	OPLAH	0.00306713	0.20994948	-0.4384431
ENSG000001	FXYD4	0.29518332	0.6512539	-0.4382967
ENSG000001	S100A2	0.2905297	0.648324	-0.4379402
ENSG000001	LRRC75B	0.01958921	0.28172722	-0.4372342
ENSG000001	PDZK1IP1	0.02074036	0.28641584	-0.4372151
ENSG000002	MRC1	0.03417537	0.32634691	-0.4372075
ENSG000002	UPK3B	0.03298315	0.32362351	-0.4362604
ENSG000002	PCDHGA1	0.33774109	0.67946062	-0.4353734
ENSG000002	TBC1D3L	0.0235487	0.29875884	-0.435322
ENSG000001	LIPI	0.3048457	0.65635638	-0.4349299
ENSG000001	S100P	0.00374793	0.21208655	-0.4341667
ENSG000002	TBC1D3C	0.30232883	0.65591444	-0.4339671
ENSG000001	PLIN4	0.0001443	0.18011717	-0.4335416
ENSG000001	LRTM2	0.13737121	0.49827463	-0.4333439
ENSG000001	SYDE1	0.444957	0.74940749	-0.4333089
ENSG00000(	F7	0.2360162	0.60336718	-0.4332368
ENSG000001	NR0B2	0.20626054	0.56956674	-0.4331983
ENSG000001	UPB1	0.03878993	0.33829709	-0.4331646
ENSG000001	SYT16	0.14032609	0.5002427	-0.4329053
ENSG000001	ARPP21	0.43305442	0.74263121	-0.4328698
ENSG000002	CLDN9	0.002799	0.20994948	-0.4323105
ENSG000002	MRPS18B	0.30220935	0.65591444	-0.4313939
ENSG000001	SPINK4	0.28313213	0.64117383	-0.4311081
ENSG00000(	DUSP13	0.11249852	0.46705151	-0.4310437
ENSG00000(	CPB2	0.42956282	0.74091639	-0.4306522
ENSG000001	ZNF560	0.25336119	0.61912837	-0.4305249
ENSG00000(	PTPRN	0.05232647	0.36780562	-0.4304284
ENSG000001	INHBE	0.24771492	0.61375236	-0.4302688
ENSG000001	RXFP1	0.34717749	0.68653332	-0.4299006
ENSG000001	OTX1	0.0119968	0.25192551	-0.4291763
ENSG000001	TIMP3	0.21832975	0.58528507	-0.4285096
ENSG000001	PTGES	0.04825488	0.360767	-0.4283736
ENSG000002	CFAP99	0.28090607	0.63927558	-0.428299
ENSG000001	C11orf53	0.35809238	0.69382497	-0.4270599
ENSG000001	NSUN7	0.01719944	0.27243096	-0.4267149

ENSG000001	HEYL	0.16191264	0.52453623	-0.4263063
ENSG000001	GUCY2D	0.09067928	0.43454471	-0.4262714
ENSG000002	SMIM38	0.0510483	0.36427913	-0.4262221
ENSG000001	ART3	0.11721612	0.47331831	-0.4262055
ENSG000001	OXT	0.30075106	0.65493729	-0.4256953
ENSG000001	PLEKHG7	0.31313061	0.6613866	-0.4252504
ENSG00000C	PRSS22	0.31071828	0.65949478	-0.4244983
ENSG000001	COL1A1	0.34571121	0.6853862	-0.4235919
ENSG000001	RHD	0.04167517	0.34831883	-0.4234879
ENSG000001	IGSF22	0.17382512	0.53862567	-0.4232027
ENSG000001	CXCL3	0.10456949	0.45514301	-0.4226838
ENSG000001	TRIM9	0.04477709	0.35545023	-0.4221318
ENSG000001	RNASE10	0.33142702	0.67444298	-0.4220106
ENSG000001	EEPD1	0.00520438	0.21833269	-0.422006
ENSG000001	CADPS	0.31240333	0.66090624	-0.4218088
ENSG000001	BICDL2	0.01271603	0.25521652	-0.4197617
ENSG000002	N/A	0.24718185	0.61321492	-0.4191374
ENSG000001	EPHX3	0.3641865	0.6973647	-0.4182264
ENSG000001	PLD5	0.29391227	0.65044158	-0.4181409
ENSG000001	DNAJB5	0.00748629	0.22933195	-0.4177143
ENSG000001	SFRP5	0.14517133	0.50398935	-0.417591
ENSG00000C	SLC7A4	0.3811406	0.71039147	-0.4175899
ENSG000001	CATSPERD	0.1428492	0.5010464	-0.4168254
ENSG000001	EMID1	0.01790149	0.27425066	-0.416637
ENSG000001	SCGB1C1	0.18613113	0.55069275	-0.4166206
ENSG000002	SOGA3	0.36558998	0.69837111	-0.4165965
ENSG000001	MYCN	0.39601565	0.72240267	-0.4158399
ENSG000001	TSPYL6	0.26311172	0.62659798	-0.4158267
ENSG000001	ADGRB3	0.14689664	0.5054701	-0.4157822
ENSG000001	CYP27A1	0.00339011	0.2114129	-0.4157322
ENSG000002	C11orf91	0.29321831	0.64988031	-0.4148228
ENSG000001	HTRA1	0.26350649	0.62687307	-0.4142195
ENSG000001	CDR2L	0.10092455	0.44917064	-0.4137859
ENSG000001	KAZALD1	0.39135614	0.71964297	-0.413588
ENSG000001	TMSB15A	0.11985449	0.47604098	-0.4132433
ENSG000001	REG4	0.26465374	0.6274921	-0.4123566
ENSG000001	CNN1	0.37159904	0.70378624	-0.4115342
ENSG000002	C16orf90	0.31595395	0.66358094	-0.4115056
ENSG000001	TP53I11	0.00181502	0.19438665	-0.4113846
ENSG000001	SHISA4	0.02157039	0.29113747	-0.411351
ENSG000001	SEPTIN5	0.06205712	0.38624952	-0.4108441
ENSG000001	SELENBP1	0.05992201	0.38277429	-0.410295
ENSG000001	ELF3	0.09558997	0.4413663	-0.4101416

ENSG000002	RPS4Y2	0.28120754	0.63941315	-0.4100884
ENSG000002	MICB	0.50875177	0.79124545	-0.4086253
ENSG000001	ZCCHC12	0.26365192	0.62687307	-0.4083283
ENSG000002	PABPN1L	0.0357351	0.33014896	-0.4071943
ENSG000002	N/A	0.37204785	0.70415443	-0.4071881
ENSG000002	PRRT1B	0.22064053	0.5880238	-0.4071476
ENSG000001	MSLN	0.39079147	0.71964297	-0.4071004
ENSG000002	N/A	0.36734735	0.69974413	-0.40607
ENSG000001	SH2D6	0.29918216	0.65423918	-0.4053341
ENSG000001	SV2C	0.24641211	0.61214306	-0.4052511
ENSG000001	NKX6-2	0.18684106	0.55108473	-0.4047298
ENSG000001	EPB42	0.04607456	0.35738124	-0.4037568
ENSG000002	C2CD4B	0.32379039	0.6699145	-0.4036993
ENSG000002	AMACR	0.1220352	0.47873532	-0.403162
ENSG000001	PPP1R27	0.28777673	0.64495772	-0.4026635
ENSG00000(	NMRK2	0.41953688	0.73452883	-0.4012502
ENSG000001	FAM83E	0.38066901	0.70987972	-0.4010748
ENSG000001	SLC6A9	0.05416118	0.37256839	-0.4009014
ENSG000001	CXCL6	0.04000908	0.34274931	-0.4005264
ENSG000001	PLB1	0.0075835	0.22933195	-0.4000572
ENSG000002	ORM1	0.0615055	0.38542193	-0.3998825
ENSG000001	TSPEAR	0.01287845	0.25583328	-0.3997003
ENSG000002	SULT1A4	0.32534059	0.67105629	-0.3996117
ENSG000001	KCNH3	0.00575095	0.21912363	-0.3995363
ENSG000001	PGM5	0.00206195	0.19943643	-0.3994602
ENSG000001	MATN3	0.30478358	0.65635638	-0.3991663
ENSG000001	GLIS3	0.06187035	0.38600832	-0.3984782
ENSG000002	LINC00514	0.03330289	0.32398208	-0.3984681
ENSG000002	TUBB3	0.36786863	0.7005727	-0.398301
ENSG000001	S100A16	0.3083007	0.6580621	-0.3981249
ENSG000001	C6orf132	0.29643582	0.65199276	-0.3979344
ENSG000001	SLC17A7	0.34717775	0.68653332	-0.397892
ENSG000001	NPW	0.40311666	0.72672584	-0.3978494
ENSG000001	AVPR1B	0.29022627	0.648324	-0.3978222
ENSG00000(	PGLYRP1	0.0191789	0.28080056	-0.3976065
ENSG000001	ARMC9	0.24816389	0.6144738	-0.3975537
ENSG000002	EFCAB8	0.00781588	0.23002982	-0.3974276
ENSG000001	GABRE	0.1592777	0.52203057	-0.3972737
ENSG000001	GAD1	0.3966833	0.72299866	-0.3970764
ENSG00000(	RPS6KA6	0.43722505	0.74497757	-0.3970279
ENSG00000(	PROM1	0.19467953	0.56205752	-0.3968777
ENSG000001	PI3	0.06911963	0.39896748	-0.3965667
ENSG000001	TMEM59L	0.48615081	0.77752459	-0.3962137

ENSG000001	BMP2	0.14508365	0.50391236	-0.3958766
ENSG000001	ZNF385C	0.07017595	0.40064939	-0.3954098
ENSG000001	SH2D4A	0.17409578	0.5392217	-0.3952965
ENSG000001	ROR2	0.34269809	0.68350189	-0.3952789
ENSG000001	TEX12	0.37259156	0.70460857	-0.3952385
ENSG000001	RHOXF2	0.36572854	0.69837111	-0.3950436
ENSG00000C	KIF4A	0.07532023	0.40943237	-0.3946185
ENSG000001	AOC3	0.00190825	0.19438665	-0.3943149
ENSG00000C	SLC4A1	0.06881727	0.39825823	-0.394141
ENSG000001	NECTIN1	0.0145773	0.26147992	-0.3939754
ENSG000002	ZNF878	0.29624084	0.65199276	-0.3937994
ENSG000001	CHIT1	0.00139938	0.18910642	-0.3935524
ENSG000001	CPAMD8	0.01406813	0.25910565	-0.3927978
ENSG00000C	CAMKK1	0.00145343	0.19195721	-0.3927291
ENSG000001	RAMP3	0.32039382	0.66729534	-0.3925847
ENSG000002	ANKRD63	0.45148031	0.75379803	-0.3922144
ENSG000001	TPST1	0.0323445	0.32362351	-0.3915777
ENSG000001	PHOSPHO1	0.0039586	0.21403539	-0.3913154
ENSG000001	VWA3A	0.13789506	0.49888639	-0.3912832
ENSG000001	MARCHF10	0.30580707	0.65705827	-0.3910195
ENSG000001	CCDC175	0.43573056	0.74462291	-0.3904724
ENSG000001	SPOCK1	0.0072093	0.22727528	-0.3902886
ENSG000001	PRL	0.44580528	0.75003008	-0.3902671
ENSG000001	ANKRD53	0.22663076	0.59418758	-0.3901101
ENSG000001	LRRC70	0.3452053	0.68522517	-0.3895607
ENSG000001	PDLIM7	0.00103256	0.18856694	-0.389524
ENSG000001	CENPA	0.35272615	0.69109369	-0.3892002
ENSG000002	N/A	0.40592783	0.72742568	-0.3890103
ENSG000002	PCDHGA10	0.41318518	0.73077485	-0.3889729
ENSG000001	COLEC11	0.28089182	0.63927558	-0.3889281
ENSG000001	CIT	0.05716646	0.37670936	-0.3886434
ENSG000001	WFDC3	0.37040159	0.70302695	-0.3885079
ENSG00000C	MCM10	0.11794039	0.47451798	-0.3881511
ENSG000001	BARHL1	0.27869499	0.63770725	-0.3881255
ENSG000002	OR2W3	0.07057353	0.40156223	-0.3874884
ENSG000001	NCCRP1	0.28664894	0.64421192	-0.3873268
ENSG000001	WFIKKN1	0.04872337	0.360767	-0.3864617
ENSG000001	CCNJL	0.00189848	0.19438665	-0.3860429
ENSG000001	UNC13A	0.4336532	0.74313742	-0.3859893
ENSG000001	PLVAP	0.02954935	0.31711354	-0.3853412
ENSG000002	N/A	0.34507547	0.68522517	-0.3852023
ENSG000002	N/A	0.09789995	0.44463533	-0.385134
ENSG000001	KIAA0895	0.13533148	0.49446444	-0.3851098

ENSG000001	VNN1	0.0433689	0.3530621	-0.3849401
ENSG000001	OR2A4	0.33450727	0.67664065	-0.3847806
ENSG00000(	FGF22	0.26185128	0.62561343	-0.3841771
ENSG000001	CYP2B6	0.14517203	0.50398935	-0.3840965
ENSG00000(	BARX2	0.11855805	0.47520101	-0.3839564
ENSG000001	CFAP69	0.43322192	0.74271189	-0.3836866
ENSG000001	H2BC13	0.42785943	0.73974265	-0.383127
ENSG000002	RNASE4	0.43715078	0.74497757	-0.3830328
ENSG000002	H4C2	0.30504696	0.65643145	-0.3827817
ENSG000001	FAM83F	0.16845008	0.53253292	-0.3816236
ENSG000001	CHRNA9	0.50635375	0.78902743	-0.38135
ENSG000001	CD248	0.02459545	0.29927225	-0.3813438
ENSG000001	RCN3	0.00021946	0.18011717	-0.3808766
ENSG000002	GOLGA6L7	0.28088419	0.63927558	-0.3801862
ENSG000001	CYP4F3	0.00375363	0.21208655	-0.3795976
ENSG000001	GLYATL2	0.44388322	0.74912503	-0.3793244
ENSG000001	NR2E1	0.39514926	0.72209218	-0.3793046
ENSG000001	NCR1	0.23036868	0.59855451	-0.37921
ENSG000001	LRP3	0.0199143	0.28363903	-0.3780757
ENSG000001	RPL3L	0.15581278	0.51800413	-0.3777862
ENSG00000(	CDH1	0.06149371	0.38542193	-0.3774988
ENSG000001	HPSE2	0.41034064	0.72965781	-0.3774125
ENSG000002	KRTAP10-4	0.2893053	0.64721	-0.3770558
ENSG000001	BEAN1	0.01038178	0.24174018	-0.3758462
ENSG000001	ASF1B	0.00083435	0.18856694	-0.3757818
ENSG000002	TMEM256-PL	0.26696875	0.62929976	-0.3756045
ENSG000001	TNNI3	0.37687771	0.70851939	-0.3747016
ENSG000001	ATP1A4	0.08650096	0.42833672	-0.3742276
ENSG000001	ALOX15B	0.03279141	0.32362351	-0.3739389
ENSG000001	CDA	0.00130829	0.18856694	-0.373448
ENSG000001	SLC24A2	0.52324912	0.79870477	-0.3730893
ENSG000002	N/A	0.29349497	0.6502221	-0.3721629
ENSG000002	CTAGE15	0.40484672	0.72672584	-0.3720617
ENSG000001	SEC14L4	0.09481005	0.43996306	-0.3719809
ENSG000001	TROAP	0.14706492	0.5054701	-0.3719469
ENSG000001	KCNS1	0.147279	0.50568178	-0.3717228
ENSG000001	TENM4	0.35896665	0.69428726	-0.3714772
ENSG000002	SIGLEC5	0.31906811	0.66632912	-0.3713504
ENSG000001	STOX1	0.09972046	0.44746644	-0.3711312
ENSG000002	PLIN5	0.00037089	0.18856694	-0.3711303
ENSG000001	PXDN	0.21354006	0.57911862	-0.3711087
ENSG000001	ALDH1L1	0.40956398	0.72909663	-0.3708313
ENSG000001	LCN2	0.03007553	0.31859418	-0.3707609

ENSG000001	DPEP2	0.00352528	0.21208655	-0.3706532
ENSG000001	PYY	0.38135972	0.71051496	-0.3704621
ENSG00000(	SLC9A3	0.02550145	0.30334587	-0.3703791
ENSG000002	TMEM150C	0.2531423	0.61912837	-0.3698321
ENSG000001	GPHA2	0.39925613	0.72496091	-0.369099
ENSG000001	INO80B	0.0225302	0.2943208	-0.3686001
ENSG000001	IMPA2	0.00068931	0.18856694	-0.3685947
ENSG000001	CAPN11	0.23458752	0.6022886	-0.3685288
ENSG000002	DYTN	0.17760352	0.5423686	-0.3680582
ENSG000002	TMED7-TICAI	0.48347305	0.77585394	-0.3678908
ENSG000001	CLEC2A	0.23863296	0.60492348	-0.3676331
ENSG000001	LIFR	0.40122192	0.72606468	-0.3675975
ENSG000001	OLFM4	0.15544365	0.51730654	-0.3667838
ENSG00000(	MMP25	0.00239737	0.2093801	-0.3667196
ENSG000001	HAS1	0.04280593	0.35185871	-0.3666139
ENSG00000(	RFX2	0.01882948	0.27889987	-0.3664387
ENSG00000(	SLC6A13	0.21405452	0.5797802	-0.3656525
ENSG000001	GLTPD2	0.24581613	0.61160228	-0.3655574
ENSG000001	FBXL22	0.21960981	0.58681553	-0.3645469
ENSG00000(	DGAT2	0.00233585	0.2093801	-0.3639611
ENSG000001	LRRC4B	0.09309074	0.43758758	-0.363875
ENSG000001	YAP1	0.3026052	0.65591444	-0.363836
ENSG00000(	HBQ1	0.03641923	0.33314876	-0.3637519
ENSG000001	MAP1LC3A	0.0004062	0.18856694	-0.3637426
ENSG000002	ADRA2B	0.3712506	0.70362349	-0.3634581
ENSG00000(	TRHDE	0.40302238	0.72672584	-0.3630434
ENSG000001	GPD1	0.09283719	0.43753271	-0.3630031
ENSG000001	ULK1	0.0006866	0.18856694	-0.3628794
ENSG000001	DOK5	0.29022791	0.648324	-0.362677
ENSG000001	KIT	0.0603997	0.38406301	-0.3622858
ENSG000002	BCKDHA	0.04931193	0.360767	-0.3621583
ENSG000001	CELF3	0.12014064	0.47648351	-0.3615372
ENSG000001	TPPP3	0.03095991	0.31953017	-0.361042
ENSG000001	TMEM132E	0.41931381	0.73438543	-0.3607736
ENSG000002	HBD	0.10865575	0.46040635	-0.35998
ENSG000001	IER5L	0.07710145	0.41179232	-0.3597847
ENSG000001	VWCE	0.06509191	0.39148468	-0.3596776
ENSG000001	KCNB1	0.00365429	0.21208655	-0.3589894
ENSG000001	FOXJ1	0.19138816	0.55685897	-0.3585659
ENSG000002	N/A	0.45295758	0.75444669	-0.3585624
ENSG000001	GLRA1	0.40419068	0.72672584	-0.3584794
ENSG000001	IHH	0.41629453	0.73241014	-0.3582842
ENSG000001	AGRP	0.00010704	0.18011717	-0.3581294

ENSG000001	OIT3	0.34156523	0.68283609	-0.357924
ENSG000001	HYAL1	0.01431327	0.2599395	-0.3576985
ENSG000001	KLC3	0.06420447	0.39043354	-0.3575785
ENSG000001	FGF18	0.39339002	0.72063539	-0.3575357
ENSG000001	HOXA7	0.39810487	0.72426093	-0.3574434
ENSG000001	GPR88	0.14174457	0.50055578	-0.356886
ENSG000002	C12orf71	0.42509539	0.73817463	-0.3567475
ENSG000001	SLC6A8	0.05331835	0.36981204	-0.3567212
ENSG000001	PCBP3	0.0141802	0.25910565	-0.3563696
ENSG000001	LRRC74B	0.32311142	0.66903175	-0.3562168
ENSG000001	MYBPH	0.05427131	0.37256839	-0.3561799
ENSG000001	FUT1	0.48016799	0.77383118	-0.3549751
ENSG000001	ENPP6	0.45692201	0.75721169	-0.3545502
ENSG00000C	IRAG1	0.00088995	0.18856694	-0.3544435
ENSG00000C	LTF	0.06479957	0.39055819	-0.3544371
ENSG000001	ATP4A	0.33650267	0.67836146	-0.3541498
ENSG000001	ETNK2	0.17378796	0.53861329	-0.3541445
ENSG000001	COL2A1	0.37640072	0.70798786	-0.3541022
ENSG000001	IQSEC3	0.29502734	0.65121791	-0.3540322
ENSG000001	SCN7A	0.47513617	0.76999017	-0.3539443
ENSG000001	PPM1H	0.01509215	0.26260224	-0.3539427
ENSG000001	CEACAM19	0.0374794	0.33471496	-0.3537678
ENSG000001	DCSTAMP	0.34292676	0.68359637	-0.3536581
ENSG000001	ESPN	0.01540875	0.26455656	-0.3534632
ENSG000001	MTTP	0.32493621	0.67056293	-0.3533789
ENSG000001	BCAN	0.35825746	0.69382497	-0.3533709
ENSG000001	TCERG1L	0.37153789	0.70378624	-0.3528721
ENSG000001	SYT2	0.01604732	0.2686189	-0.3526676
ENSG000002	HBM	0.03913582	0.33942602	-0.3526456
ENSG000001	RFLNB	0.00850884	0.23436355	-0.3524859
ENSG000002	H3Y2	0.51046059	0.79143652	-0.3523846
ENSG000001	CDC20	0.1247775	0.48220256	-0.352223
ENSG000001	DLGAP3	0.01791626	0.27425066	-0.3519726
ENSG000002	N/A	0.46153233	0.76122418	-0.3513166
ENSG00000C	MRC2	0.04510954	0.35545023	-0.3507774
ENSG00000C	MADCAM1	0.03550497	0.33014896	-0.350662
ENSG000001	KANK2	0.02880352	0.31345545	-0.3504027
ENSG000002	TTC34	0.42523546	0.73817463	-0.3503676
ENSG000001	OTOGL	0.30050281	0.65493729	-0.3500901
ENSG000001	ALK	0.19942232	0.56600138	-0.3496903
ENSG000002	RHEX	0.17627395	0.54121794	-0.3495493
ENSG000001	CCR10	0.05663627	0.37516454	-0.3490435
ENSG00000C	SMIM24	0.05196126	0.36634603	-0.3487391

ENSG000002	SENP3-EIF4A	0.51353753	0.79316402	-0.3484972
ENSG000001	THBS1	0.03278831	0.32362351	-0.3484395
ENSG00000C	FRMPD1	0.35821885	0.69382497	-0.3482831
ENSG000001	PEX5L	0.53747428	0.80547376	-0.3482393
ENSG000001	IL20	0.32111579	0.66766831	-0.3478085
ENSG000001	CYP2A7	0.43667454	0.74496191	-0.3468518
ENSG000001	GCNT3	0.33108886	0.67443203	-0.3468098
ENSG00000C	PRSS8	0.25895026	0.62261049	-0.3467573
ENSG000001	CEACAM8	0.06831358	0.39743754	-0.3467061
ENSG00000C	SLC25A39	0.04552966	0.3556589	-0.3465459
ENSG000001	SPINK2	0.32261696	0.66849561	-0.3461807
ENSG000001	GRIN3B	0.00715352	0.22727528	-0.3461159
ENSG000001	UPK1A	0.47507999	0.76999017	-0.3459594
ENSG00000C	MAPRE3	0.01344183	0.25828987	-0.345879
ENSG000001	ATCAY	0.36199908	0.69592458	-0.3457772
ENSG000001	UFSP1	0.1408894	0.50051652	-0.3457118
ENSG000002	PCDHGA5	0.43557272	0.74461931	-0.3455535
ENSG000001	APCDD1	0.12565424	0.48370029	-0.3454397
ENSG000001	ALAS2	0.11598098	0.47160917	-0.3453864
ENSG000001	BTNL3	0.0986707	0.44552729	-0.3452958
ENSG000001	GLS2	0.16455853	0.52786788	-0.3448291
ENSG000002	NPIPA9	0.42088523	0.7350227	-0.3444557
ENSG000002	MRM1	0.21474843	0.58027463	-0.344052
ENSG000001	UBTD1	0.00132018	0.18856694	-0.3440394
ENSG000002	N/A	0.49850257	0.78316099	-0.3439969
ENSG000002	TAF11L2	0.41225903	0.73053039	-0.3439865
ENSG000001	MMP21	0.47165859	0.76694135	-0.3432985
ENSG000001	SULT1A2	0.03152218	0.32149851	-0.3431563
ENSG000001	CITED4	0.01046454	0.24174018	-0.3430557
ENSG000002	CCDC153	0.00455346	0.21750928	-0.3429154
ENSG000001	DENND2B	0.07878812	0.41446832	-0.3427866
ENSG000001	IGF2BP1	0.45296795	0.75444669	-0.3427725
ENSG000001	ABTB1	0.00060338	0.18856694	-0.3426858
ENSG00000C	CRISP3	0.08427375	0.42405214	-0.3424447
ENSG000002	CASTOR2	0.03102984	0.31953017	-0.3422096
ENSG000001	PLPPR2	0.00068053	0.18856694	-0.3421195
ENSG000001	NPR3	0.32766342	0.67217574	-0.3411518
ENSG000001	FOXE1	0.45418428	0.7555818	-0.340791
ENSG000002	SHISA8	0.33285698	0.67523724	-0.3393798
ENSG000002	GPX3	0.01556869	0.26471158	-0.3391165
ENSG000001	AP1M2	0.00682599	0.22347774	-0.3390988
ENSG000001	NLRP12	0.00298236	0.20994948	-0.3390241
ENSG000002	C19orf81	0.44416273	0.7491529	-0.3389903

ENSG000001	MME	0.00956879	0.23648166	-0.338965
ENSG000002	CFAP45	0.0080748	0.23082837	-0.3385592
ENSG000001	PHLDA3	0.19577849	0.56346286	-0.3380898
ENSG000001	SH2D4B	0.21671496	0.58337291	-0.3379919
ENSG000001	BEST4	0.07994525	0.41591462	-0.337647
ENSG00000C	ITGA2B	0.06205991	0.38624952	-0.3369427
ENSG000001	TMEM91	0.004631	0.21772133	-0.3366239
ENSG000001	CNTNAP3	0.10650333	0.4566397	-0.3364064
ENSG000001	MEGF6	0.0161752	0.26909504	-0.3359515
ENSG000002	MANSC4	0.5322052	0.80280163	-0.3357793
ENSG000001	ITGAD	0.01930465	0.28112065	-0.3355119
ENSG000001	WNT1	0.28233422	0.64034932	-0.3351771
ENSG000001	NXPH4	0.19527776	0.56280385	-0.3350903
ENSG000001	GGT1	0.00261336	0.2093801	-0.3345991
ENSG000002	LY6G5B	0.16893828	0.53296205	-0.3345936
ENSG000001	OSBP2	0.05091926	0.36357784	-0.3343365
ENSG000001	SLC19A1	0.00152615	0.19292382	-0.3336237
ENSG000001	CRISPLD2	0.01215346	0.25234748	-0.3335165
ENSG000001	ADAMTS5	0.34856657	0.6878781	-0.3334345
ENSG000001	NFASC	0.3134397	0.66161883	-0.333311
ENSG000001	VWF	0.03706449	0.3336458	-0.3330348
ENSG000001	WNT6	0.14058379	0.50051652	-0.3328166
ENSG000001	ASB12	0.51215692	0.7921414	-0.3316925
ENSG000001	TIGD3	0.00042966	0.18856694	-0.3316046
ENSG00000C	ARX	0.46608509	0.76484555	-0.3313933
ENSG000001	FOXI2	0.5060262	0.78874871	-0.3310179
ENSG000001	KRT8	0.22117062	0.58861619	-0.330896
ENSG000002	NATD1	0.00162397	0.19292382	-0.3308545
ENSG000001	LRRC18	0.34427602	0.68479845	-0.3306654
ENSG000001	TMC1	0.46620744	0.76485906	-0.3305651
ENSG000001	ESPL1	0.08210634	0.42068814	-0.3305295
ENSG000002	BTBD19	0.001382	0.18856694	-0.3303987
ENSG000001	NR6A1	0.00459479	0.21750928	-0.3303241
ENSG00000C	ATP2B3	0.33034023	0.67392064	-0.3300845
ENSG000001	GABRB1	0.5165873	0.79535634	-0.3300181
ENSG000001	TBX3	0.30356021	0.6561378	-0.3298382
ENSG000001	PINK1	0.00285039	0.20994948	-0.3296141
ENSG000002	LRRC3C	0.21715617	0.583769	-0.3295892
ENSG000001	ACE2	0.44366952	0.74908353	-0.3290952
ENSG000001	DBN1	0.0010699	0.18856694	-0.3285636
ENSG000002	CYP2A6	0.10834539	0.45963571	-0.3284052
ENSG000002	N/A	0.46311403	0.76278986	-0.3277017
ENSG000001	ZNF219	0.01267073	0.25521652	-0.3276621

ENSG000001	B4GALNT4	0.12909429	0.4871968	-0.3272253
ENSG000001	FKBP8	0.03286984	0.32362351	-0.3271348
ENSG000001	TRPM6	0.01558498	0.26471158	-0.3270187
ENSG000001	SLC43A2	0.00720332	0.22727528	-0.326832
ENSG000001	EVPL	0.11406796	0.46962187	-0.3268114
ENSG000001	TDRD12	0.34401551	0.68472435	-0.3266216
ENSG000001	HNF1A	0.45497479	0.7559601	-0.3262452
ENSG000001	GYPC	0.02746835	0.31123767	-0.3258134
ENSG000001	ADAM20	0.42339884	0.73723614	-0.3256466
ENSG000001	BUB1B	0.14048833	0.50035294	-0.3252967
ENSG000002	FAM71F2	0.0011266	0.18856694	-0.3252949
ENSG000002	SRD5A2	0.43037981	0.7410474	-0.3251812
ENSG000002	BOLA2-SMG1	0.26844903	0.63073283	-0.3249746
ENSG000001	H1-2	0.00369192	0.21208655	-0.3249399
ENSG000000	TNS1	0.09535136	0.44121402	-0.3248541
ENSG000002	ATF6B	0.47146639	0.76694135	-0.3246125
ENSG000001	TUBB8B	0.21705029	0.58371881	-0.3244874
ENSG000000	YBX3	0.04567876	0.35605405	-0.3242581
ENSG000001	B3GNT8	0.00488086	0.21772133	-0.3241491
ENSG000001	MTHFS	0.0162508	0.26979984	-0.3241326
ENSG000001	SNX7	0.49253611	0.7804495	-0.3241025
ENSG000001	CCDC150	0.14941274	0.50925123	-0.3239762
ENSG000000	PLXNA2	0.00546183	0.21833269	-0.3237859
ENSG000001	CHI3L1	0.02265295	0.29448836	-0.3234881
ENSG000001	THBD	0.02909521	0.3136912	-0.3233245
ENSG000000	BIRC5	0.18836712	0.55257759	-0.3231999
ENSG000001	MGP	0.43372787	0.74318693	-0.3229342
ENSG000001	KISS1R	0.18761229	0.55167498	-0.3217856
ENSG000001	LDB3	0.3595734	0.69484359	-0.3216568
ENSG000002	ERFL	0.03377612	0.32489508	-0.3216015
ENSG000001	TLX2	0.42868499	0.74045488	-0.3214155
ENSG000001	TPSAB1	0.20208	0.56777015	-0.3211682
ENSG000001	S100A3	0.31407191	0.66224858	-0.3209892
ENSG000002	RTL8B	0.33244622	0.67523724	-0.3208974
ENSG000001	LRRC75A	0.0134207	0.25828987	-0.320761
ENSG000000	TNFRSF12A	0.00746865	0.22933195	-0.3207224
ENSG000001	PNMT	0.37604472	0.70771854	-0.3206958
ENSG000001	COL18A1	0.00106982	0.18856694	-0.3204199
ENSG000001	MYL9	0.06772438	0.39689346	-0.3201036
ENSG000001	NBPF4	0.3966986	0.72299866	-0.3200572
ENSG000000	GTSE1	0.14225911	0.50068215	-0.320018
ENSG000001	TST	0.00276502	0.20994948	-0.3199855
ENSG000001	AP3B2	0.20855001	0.57228774	-0.3196498

ENSG00000(	PHACTR3	0.13492613	0.49418805	-0.3193254
ENSG000001	RELL1	0.03282861	0.32362351	-0.3191678
ENSG000001	CLRN3	0.42720754	0.73942744	-0.3188915
ENSG000001	CLDN5	0.0462083	0.35738124	-0.3188584
ENSG00000(	LAMC3	0.14199848	0.50068215	-0.3185664
ENSG00000(	NRXN3	0.59201718	0.83377422	-0.3177773
ENSG000001	DSC3	0.39781649	0.72397964	-0.3176303
ENSG000002	SRXN1	0.04363318	0.35372007	-0.3173745
ENSG000001	RAB25	0.30761367	0.65762361	-0.3173145
ENSG000001	CLCN1	0.16377278	0.52709285	-0.3171144
ENSG000001	GP9	0.03183198	0.3227302	-0.3167823
ENSG000001	C1orf116	0.05598641	0.37502119	-0.3163568
ENSG000001	OR1R1P	0.28992537	0.64806142	-0.3162349
ENSG000001	BAIAP2	0.00659644	0.22296216	-0.3160823
ENSG000001	TK1	0.05181888	0.36581879	-0.314659
ENSG000001	HSPG2	0.10204431	0.45141805	-0.3146179
ENSG000001	TRIM58	0.1233293	0.47994951	-0.3141723
ENSG000001	VMO1	0.11096144	0.46411152	-0.3140951
ENSG000001	ATP23	0.6058778	0.83946052	-0.3138826
ENSG000001	TMEM251	0.40676961	0.7275473	-0.3138538
ENSG000002	ASPDH	0.05570696	0.37502119	-0.3138488
ENSG000001	MARVELD2	0.41471603	0.73184916	-0.3137884
ENSG000002	OR10G2	0.36362319	0.69694698	-0.3137346
ENSG000001	NKPD1	0.14986832	0.50942953	-0.3135625
ENSG000001	CIBAR2	0.40310428	0.72672584	-0.3134162
ENSG000001	KCND3	0.11552629	0.4710215	-0.3133627
ENSG000001	TAF7L	0.51497591	0.79423098	-0.3133311
ENSG000001	NEK2	0.21892963	0.58591803	-0.3132255
ENSG00000(	ME1	0.2513013	0.61695463	-0.3130391
ENSG000001	AJAP1	0.13961559	0.49975817	-0.3129056
ENSG000001	MKI67	0.11083766	0.46380827	-0.3126624
ENSG000001	PRRG1	0.41383207	0.73141372	-0.3126506
ENSG000001	FAM83D	0.01558565	0.26471158	-0.3124045
ENSG000002	AQP1	0.0555781	0.37486021	-0.3123912
ENSG000001	GREM2	0.21332754	0.57903699	-0.3121668
ENSG000001	ZNF746	0.00119724	0.18856694	-0.3121188
ENSG000001	ECRG4	0.30570544	0.65701379	-0.3120945
ENSG000001	QPCT	0.00537685	0.21833269	-0.3120809
ENSG000001	NAV2	0.34609293	0.68588759	-0.3118819
ENSG000001	SALL4	0.55153298	0.81323014	-0.3118678
ENSG000001	POTEE	0.43435872	0.7438413	-0.3118142
ENSG000001	KSR2	0.56067646	0.81728196	-0.3111981
ENSG000001	HOXA4	0.51820393	0.79572786	-0.3109877

ENSG000001	CTSF	0.09973401	0.44746644	-0.3109112
ENSG000001	KLHDC9	0.57348994	0.82246568	-0.3108613
ENSG000001	ADAMTS7	0.57495461	0.82344878	-0.3108191
ENSG00000C	TRO	0.08473163	0.42501928	-0.3106434
ENSG000001	NBL1	0.08677523	0.42885945	-0.3103896
ENSG000001	TSPAN7	0.25007948	0.61596135	-0.3103113
ENSG000001	NIPAL4	0.10009993	0.44762396	-0.3100077
ENSG000001	ISL2	0.02487971	0.30012694	-0.3096749
ENSG00000C	CDC6	0.06320516	0.38916273	-0.3096516
ENSG000001	KIAA2012	0.45814139	0.75759807	-0.3095453
ENSG000001	PEAR1	0.06926129	0.39935921	-0.3095322
ENSG000001	RHCE	0.04813982	0.36070431	-0.309508
ENSG00000C	FSCN1	0.02863795	0.31345545	-0.3094309
ENSG000001	FAXDC2	0.03356429	0.32398208	-0.3092906
ENSG000001	LRG1	0.00783771	0.23002982	-0.3091173
ENSG000002	LNP1	0.28750231	0.64495772	-0.3089018
ENSG000002	TLR9	0.02862358	0.31345545	-0.3088637
ENSG000001	WDR38	0.2773927	0.63709819	-0.3088262
ENSG000002	CEBPD	0.00599416	0.22079879	-0.3086572
ENSG000002	CAPN8	0.31723813	0.66464458	-0.3085802
ENSG000001	ABCG5	0.45483906	0.7559601	-0.3083981
ENSG000001	N/A	0.45743343	0.75742878	-0.3082606
ENSG000001	AHSP	0.15715946	0.5199629	-0.307836
ENSG00000C	BLVRB	0.02070961	0.28641584	-0.3076087
ENSG000001	TMEM89	0.57168233	0.82167	-0.3074834
ENSG000001	BMX	0.10817963	0.45961253	-0.3070148
ENSG000001	GOLGA6L9	0.43781136	0.74549116	-0.306477
ENSG000001	TRPV5	0.44813042	0.75114004	-0.3064446
ENSG000002	SCRT2	0.36996067	0.70295438	-0.3063725
ENSG000002	N/A	0.43452873	0.74400959	-0.3063355
ENSG000001	CYP3A4	0.57525199	0.82344878	-0.3061946
ENSG000001	BASP1	0.01805189	0.27573705	-0.3058395
ENSG000001	CTSG	0.19279149	0.55869274	-0.3058158
ENSG000001	LMNTD2	0.45466998	0.75585917	-0.3057509
ENSG000001	PRSS57	0.25450342	0.61954604	-0.3056581
ENSG000001	ADGRG3	0.01009156	0.24143268	-0.3055178
ENSG000001	CDT1	0.12658566	0.48433718	-0.3052733
ENSG000001	WLS	0.01550124	0.26471158	-0.3050134
ENSG000001	TMEM119	0.0699169	0.4002892	-0.3046454
ENSG000001	C4orf19	0.08348763	0.42260872	-0.304627
ENSG000001	BRICD5	0.01880456	0.27889987	-0.3044657
ENSG000001	GCAT	0.03027468	0.31901949	-0.3039641
ENSG00000C	COL23A1	0.22348727	0.59081204	-0.3038635

ENSG00000(	SLC27A5	0.05596592	0.37502119	-0.3036433
ENSG000001	SEC14L5	0.14536945	0.50413578	-0.3036339
ENSG000001	SYCE2	0.05833614	0.37994538	-0.3035703
ENSG000001	PALM3	0.31913656	0.66638636	-0.3032517
ENSG000002	PCDHGB3	0.58207373	0.82746456	-0.3030316
ENSG000002	N/A	0.17036921	0.53437545	-0.3029317
ENSG00000(	PHLDB1	0.18741696	0.5514841	-0.3025983
ENSG000002	CR1	0.0219853	0.29231997	-0.3024867
ENSG000001	CDH9	0.44100055	0.74750808	-0.3023943
ENSG00000(	FUZ	0.00150863	0.19292382	-0.3022636
ENSG00000(	ANK1	0.10311654	0.45287405	-0.302208
ENSG000001	KCNJ4	0.37548393	0.70743725	-0.3021596
ENSG000001	IER3	0.44542887	0.74955214	-0.3021447
ENSG000001	PACSIN3	0.49725625	0.78258696	-0.3021082
ENSG000001	RUBCNL	0.00949669	0.23577638	-0.3020673
ENSG000001	DMTN	0.06863074	0.39796501	-0.3018491
ENSG000001	RAMP2	0.46833958	0.76580361	-0.3016689
ENSG000001	LYVE1	0.13952202	0.49975817	-0.301636
ENSG000002	DIO1	0.16779583	0.53223305	-0.3015815
ENSG000001	FOXD4L1	0.3386255	0.68022544	-0.3014727
ENSG000001	HTRA4	0.43128406	0.74126857	-0.3013935
ENSG000001	ECHDC3	0.03224249	0.3235608	-0.301138
ENSG000001	ZNF385D	0.04230206	0.35009105	-0.3007517
ENSG000001	LAMB3	0.04538632	0.35545023	-0.3005003
ENSG00000(	SMOX	0.04579332	0.35661113	-0.3004437
ENSG00000(	TACC3	0.00113952	0.18856694	-0.3002913
ENSG000001	ZNF534	0.39369836	0.72076675	-0.3001258
ENSG000002	ACY1	0.35932274	0.69472772	-0.2998537
ENSG000001	DOK4	0.06332869	0.38950527	-0.2997573
ENSG000001	DLGAP5	0.25489782	0.6197628	-0.2992994
ENSG000002	SMIM1	0.03778689	0.33525901	-0.2991108
ENSG000001	TEX29	0.42790752	0.73974265	-0.2990563
ENSG000002	ALG1L2	0.11923035	0.4760126	-0.2989273
ENSG000001	TMEM190	0.42278356	0.73663877	-0.2987118
ENSG000001	HROB	0.09028067	0.43395124	-0.298557
ENSG000001	GALNT18	0.51125549	0.79171125	-0.2984602
ENSG00000(	DLL3	0.555475	0.8145753	-0.2983455
ENSG000002	LRRD1	0.53911663	0.80656378	-0.2983092
ENSG000001	ENPP7	0.38829617	0.71710248	-0.2982017
ENSG000001	KCP	0.30294112	0.65591444	-0.2981566
ENSG000001	SPINT1	0.0153298	0.26409578	-0.2981403
ENSG000001	ZSCAN10	0.30559893	0.65695881	-0.2981383
ENSG000001	CALY	0.33357431	0.67601673	-0.2980839

ENSG000001	NINJ2	0.00933651	0.23551879	-0.2980326
ENSG000001	CA1	0.21956411	0.58681553	-0.2979059
ENSG000001	CLMP	0.03356262	0.32398208	-0.297748
ENSG000001	BSG	0.01306554	0.25683445	-0.2977053
ENSG000001	GNMT	0.03194538	0.3227302	-0.2975981
ENSG000001	RGS2	0.01386349	0.25910565	-0.2975542
ENSG000001	RGS16	0.0555029	0.37471955	-0.2975178
ENSG000001	BPIFC	0.35371053	0.69146026	-0.29749
ENSG00000(	MLXIPL	0.52571022	0.79977109	-0.2969031
ENSG000001	RNASE8	0.3694252	0.70230148	-0.2967691
ENSG000001	WASF3	0.22836624	0.59653313	-0.2966415
ENSG00000(	SLC12A1	0.14483209	0.50345508	-0.2965291
ENSG000001	BFSP2	0.41763991	0.73321581	-0.2958668
ENSG000001	TCN1	0.01693757	0.27175434	-0.2957857
ENSG000001	TMOD1	0.14884332	0.50868638	-0.2953809
ENSG000002	LY6G6E	0.4579002	0.7575144	-0.2953147
ENSG000001	WFDC5	0.35600375	0.69235032	-0.2950942
ENSG000001	SGK1	0.04238847	0.35043867	-0.2950119
ENSG000001	KCNK9	0.44628375	0.750524	-0.2949203
ENSG000002	MPC1L	0.28286483	0.64084947	-0.2946768
ENSG000001	CFAP161	0.03023144	0.3189525	-0.2945799
ENSG000001	ENKD1	0.00611118	0.22079879	-0.2944705
ENSG000001	RAB3A	0.04070969	0.34551949	-0.2943187
ENSG000001	NTNG1	0.4869518	0.77767904	-0.2941294
ENSG000001	PLK2	0.01823941	0.27755705	-0.2940497
ENSG000001	RBM38	0.03517326	0.32931219	-0.293568
ENSG000001	CYP1A2	0.47918375	0.77317962	-0.2932815
ENSG000002	FAM236C	0.35162501	0.69054758	-0.2927823
ENSG00000(	SPTB	0.08266557	0.42208833	-0.2925205
ENSG000002	CATSPERZ	0.24133119	0.60721888	-0.2924216
ENSG000001	PHC2	0.00870072	0.23436355	-0.2924069
ENSG000001	KCNJ12	0.47865693	0.77255989	-0.2923414
ENSG000001	CPA3	0.26439456	0.6274921	-0.2919837
ENSG000001	SCNN1B	0.59627612	0.83586476	-0.2919466
ENSG000002	TMEM158	0.11163542	0.46525265	-0.2919245
ENSG000002	MEX3A	0.44891632	0.751914	-0.2914754
ENSG000001	C19orf18	0.32183552	0.66822528	-0.2914281
ENSG000001	COCH	0.16755634	0.53192406	-0.2910861
ENSG000001	ABCC3	0.02149688	0.2908707	-0.2910588
ENSG000001	FILIP1L	0.1190286	0.47567495	-0.290584
ENSG000001	HBG2	0.26864363	0.63079288	-0.2905119
ENSG000001	DAB2IP	0.20370597	0.56880022	-0.2905039
ENSG000001	EPGN	0.54840156	0.81148237	-0.2901151

ENSG000001	SPACA6	0.01097181	0.24311475	-0.29008
ENSG000001	MAGI1	0.56804833	0.82037292	-0.2898403
ENSG000001	MYADM	0.0095303	0.23624711	-0.2895312
ENSG000001	TUBB	0.55091531	0.81290547	-0.2894813
ENSG000001	NLRX1	0.00929327	0.23551879	-0.2894667
ENSG000001	SLC35D3	0.26618741	0.62894135	-0.2893732
ENSG000001	LAMA4	0.36682968	0.69892851	-0.2893126
ENSG000001	ARHGEF40	0.00316596	0.21033652	-0.2892162
ENSG00000C	MLN	0.48398395	0.77652642	-0.2891276
ENSG000001	SLC29A4	0.0458979	0.35708873	-0.2887994
ENSG000002	MFSD2B	0.04559082	0.3556589	-0.288772
ENSG000001	MAP7	0.03364438	0.32398208	-0.2887312
ENSG000001	KCNS3	0.46920636	0.7662622	-0.2882905
ENSG00000C	YBX2	0.55208126	0.81351696	-0.2878902
ENSG000001	AOC2	0.02186044	0.29231997	-0.2878332
ENSG000001	LETM2	0.00904992	0.23513527	-0.287776
ENSG000001	FCHO1	0.00304286	0.20994948	-0.2876035
ENSG000001	PDZRN3	0.49280745	0.7804495	-0.2872325
ENSG000001	TBXT	0.45184831	0.75386982	-0.2866919
ENSG000001	CGN	0.38730201	0.71665216	-0.2865614
ENSG000001	CNIH3	0.03560847	0.33014896	-0.286488
ENSG00000C	ACHE	0.2986691	0.65406944	-0.2862318
ENSG000001	EME1	0.41854825	0.73381176	-0.2861944
ENSG000001	SLC32A1	0.44206663	0.74830001	-0.2861258
ENSG000002	AGER	0.27683617	0.63632345	-0.2855532
ENSG000001	PYGL	0.01347583	0.25833181	-0.2852159
ENSG00000C	TPX2	0.08037985	0.41684053	-0.2851543
ENSG000001	ERICH6	0.52642979	0.80019103	-0.2850369
ENSG000002	ATAD3C	0.11062029	0.46340085	-0.2850273
ENSG000001	AGMO	0.1964283	0.56389367	-0.2849924
ENSG000001	ZSWIM4	0.02075759	0.28641584	-0.2849019
ENSG000002	VSIG8	0.59775799	0.83641817	-0.2848845
ENSG000001	EDA	0.05181446	0.36581879	-0.2845357
ENSG000001	OXER1	0.03693978	0.33341755	-0.2844504
ENSG000001	GJA1	0.51061966	0.79143652	-0.2842988
ENSG00000C	STRADB	0.11499568	0.47079584	-0.2841351
ENSG000001	ABHD14A-AC	0.50294951	0.7863641	-0.2841099
ENSG000001	TMPRSS5	0.24553008	0.61145565	-0.2838448
ENSG000001	NEFM	0.51349349	0.79316402	-0.2838241
ENSG000001	FOLR3	0.00646573	0.22296216	-0.2837889
ENSG000001	NTRK3	0.57234532	0.82182269	-0.2837324
ENSG000001	KCNE1	0.03684498	0.33341755	-0.2835031
ENSG000001	ROPN1L	0.02892414	0.31345545	-0.2832918

ENSG000001	C5AR2	0.00020856	0.18011717	-0.2832833
ENSG00000(	SLC38A5	0.05067015	0.36298244	-0.2830177
ENSG000001	EIF1AY	0.09522411	0.44084511	-0.2830071
ENSG000002	STARD10	0.00072742	0.18856694	-0.2829937
ENSG000001	OR5B2	0.35294981	0.69109369	-0.2829289
ENSG000001	SULF2	0.00040544	0.18856694	-0.2826127
ENSG000001	PCSK4	0.11541111	0.4710215	-0.2824776
ENSG000001	SULT1A1	0.0020758	0.19943643	-0.2823908
ENSG000001	ATP1A3	0.07452734	0.40721124	-0.2822129
ENSG000001	CRACR2B	0.06239653	0.38624952	-0.2820352
ENSG000001	C5AR1	0.00315959	0.21033652	-0.2820201
ENSG000001	IMPDH1	0.00116269	0.18856694	-0.2820112
ENSG000001	TMC5	0.0246799	0.29927225	-0.2819045
ENSG000001	ABCA13	0.05158974	0.36531299	-0.2818539
ENSG000001	LHFPL4	0.48644751	0.77752459	-0.2818376
ENSG000001	PXDNL	0.13810361	0.49898885	-0.2817099
ENSG000001	BTNL8	0.00247867	0.2093801	-0.2816131
ENSG000001	EYA1	0.50066475	0.7851753	-0.2814024
ENSG000001	FAM171B	0.50902361	0.79132674	-0.2807411
ENSG000001	INKA2	0.02785766	0.31135087	-0.2807049
ENSG000001	DEF8	0.00667443	0.22330866	-0.2806932
ENSG00000(	MAST3	0.00184764	0.19438665	-0.2806141
ENSG000001	TMEM139	0.40426044	0.72672584	-0.2803673
ENSG000002	PPP1R3G	0.41565078	0.73222542	-0.2800544
ENSG000001	CHRNG	0.26939259	0.63100512	-0.2795011
ENSG000001	ALOX5AP	0.00939633	0.23551879	-0.2794432
ENSG000002	MEI4	0.54004212	0.80698777	-0.2793469
ENSG000002	ODAD4	0.13919224	0.49975817	-0.2793266
ENSG000001	ASCC2	0.0364984	0.33341755	-0.2786655
ENSG000001	HLX	0.02095055	0.28755204	-0.2784247
ENSG000001	MED25	0.00466105	0.21772133	-0.2784154
ENSG000001	NKAPL	0.10511665	0.45525417	-0.2781212
ENSG000002	N/A	0.44975236	0.75279291	-0.2780133
ENSG000001	KCNJ11	0.53269599	0.80280163	-0.2777211
ENSG000001	CYP8B1	0.4799485	0.77364477	-0.2777036
ENSG00000(	P4HA2	0.00286354	0.20994948	-0.2774166
ENSG00000(	CLTCL1	0.00120196	0.18856694	-0.2772389
ENSG000001	CPXM2	0.52758766	0.80062313	-0.2772248
ENSG000001	SERPINA11	0.5208238	0.79735075	-0.2771728
ENSG000001	BCL2L1	0.09956514	0.44745066	-0.2770853
ENSG00000(	TESC	0.0558438	0.37502119	-0.2770393
ENSG000001	FBXO24	0.0539846	0.37208157	-0.2769289
ENSG000002	ZGLP1	0.01383425	0.25910565	-0.2767548

ENSG000001	BBC3	0.01274431	0.25546834	-0.2766042
ENSG000001	TMEM45B	0.0845281	0.42469888	-0.2763094
ENSG000001	KBTBD12	0.58447317	0.82887597	-0.2762294
ENSG000001	RBM24	0.24864077	0.61459845	-0.276171
ENSG000002	PBX2	0.29921662	0.65423918	-0.2759833
ENSG000002	PRSS1	0.517944	0.79566132	-0.2758877
ENSG000002	SLC48A1	0.00699233	0.22577227	-0.2757946
ENSG000001	TOX2	0.02371708	0.29875884	-0.2757882
ENSG000001	CFAP43	0.41320712	0.73077485	-0.2755798
ENSG000001	PKMYT1	0.10352064	0.45340382	-0.2753033
ENSG00000C	COL9A3	0.11384961	0.46939348	-0.2752905
ENSG000001	KRT1	0.20686449	0.57036149	-0.2752773
ENSG000001	SHC2	0.48629418	0.77752459	-0.2752692
ENSG000002	ZNF709	0.4661521	0.76484555	-0.2752109
ENSG000001	TUBB2A	0.10879742	0.46040635	-0.275019
ENSG000001	DRD3	0.62670869	0.85082517	-0.2749901
ENSG000001	MGAT5B	0.59251531	0.83404171	-0.2749731
ENSG000001	ANKK1	0.09630923	0.44215472	-0.2748852
ENSG000001	SYT5	0.48936699	0.77976956	-0.2747595
ENSG000001	F5	0.02489813	0.30012694	-0.2747443
ENSG000001	NRGN	0.06023651	0.38377569	-0.274708
ENSG000002	IFIT1B	0.13297032	0.49180844	-0.2746001
ENSG000002	TMEM225B	0.51951862	0.79650824	-0.2745538
ENSG00000C	CDC34	0.02773722	0.3113392	-0.2745043
ENSG000001	NCKAP5	0.58616934	0.82959975	-0.2741794
ENSG000001	BIRC7	0.51850073	0.79577989	-0.273986
ENSG000001	TNXB	0.53036362	0.8019663	-0.2739476
ENSG000001	NRIP3	0.05582607	0.37502119	-0.2739104
ENSG000001	ADGRE3	0.00531925	0.21833269	-0.2736505
ENSG000002	REELD1	0.06118968	0.38491421	-0.2731544
ENSG000001	TMEM120A	0.00079192	0.18856694	-0.2730526
ENSG000001	VPS37D	0.41403613	0.73141372	-0.2725329
ENSG000001	DYNLRB2	0.49224749	0.7804495	-0.2723067
ENSG000001	C1QTNF1	0.54794128	0.81141647	-0.2721175
ENSG000001	CACNA1I	0.10218399	0.45160695	-0.2720207
ENSG000001	S100A7	0.3239454	0.6699145	-0.2719555
ENSG00000C	TSPAN9	0.06965457	0.40004644	-0.2719017
ENSG000001	DOK3	0.00637432	0.22284683	-0.2717293
ENSG000001	RBP7	0.01935118	0.28154578	-0.271553
ENSG000001	GDF5	0.44250039	0.74855288	-0.2711516
ENSG000002	FXYD1	0.47172774	0.76694135	-0.2711385
ENSG000001	NFE2	0.00283806	0.20994948	-0.2710785
ENSG000001	ROM1	0.03099603	0.31953017	-0.2709944

ENSG000001	HEPHL1	0.52639925	0.80019103	-0.2709424
ENSG000001	RTN4RL1	0.14799492	0.50706204	-0.2706875
ENSG000001	ARAP3	0.00368211	0.21208655	-0.270644
ENSG000001	DHX34	0.0008982	0.18856694	-0.2705325
ENSG000001	CLN8	0.56996546	0.82058247	-0.270348
ENSG00000(	TRIP13	0.3102653	0.65949478	-0.2699511
ENSG00000(	KCNH2	0.06531208	0.39169239	-0.2699142
ENSG000001	EPOR	0.00128787	0.18856694	-0.2697446
ENSG000001	TMEM86B	0.01510956	0.26260224	-0.2697222
ENSG000001	STAB2	0.43242597	0.74197405	-0.2694886
ENSG00000(	RPGRIP1	0.02045534	0.28641584	-0.2692504
ENSG000001	GJC1	0.54852102	0.81157638	-0.2689312
ENSG000001	GYPE	0.12396701	0.48115857	-0.2688399
ENSG000001	TMEM88	0.05994459	0.38277429	-0.2688141
ENSG00000(	HAL	0.00898858	0.23513527	-0.2688094
ENSG000001	INSM2	0.44342963	0.74898844	-0.2687439
ENSG000001	KEL	0.53696568	0.80503473	-0.2685878
ENSG000002	TBC1D3E	0.26588587	0.62868632	-0.268456
ENSG000002	PECAM1	0.00262609	0.2093801	-0.2682597
ENSG000001	KDR	0.5433902	0.8084284	-0.2682596
ENSG000002	N/A	0.54677814	0.81063154	-0.2682565
ENSG000001	AWAT2	0.5281318	0.80062313	-0.2681457
ENSG000001	FAM210B	0.09093825	0.43488031	-0.2679968
ENSG000002	RNF208	0.12288095	0.4796197	-0.2679901
ENSG00000(	PLEKHH3	0.14153567	0.50055578	-0.2679032
ENSG000001	GCK	0.44641901	0.75067372	-0.2676859
ENSG000001	GLI1	0.00485116	0.21772133	-0.2672307
ENSG000001	LYL1	0.03134753	0.32055029	-0.2669558
ENSG000001	IGFBP5	0.61032758	0.84310779	-0.266937
ENSG00000(	NRCAM	0.30681956	0.65749363	-0.26664
ENSG000001	RAPSN	0.04626929	0.35738124	-0.2663957
ENSG000001	PEAK3	0.0427132	0.35185871	-0.2660682
ENSG000002	ANKUB1	0.18248753	0.54678908	-0.2659937
ENSG000001	CAMP	0.04739629	0.35865951	-0.2658539
ENSG000001	CHRNA2	0.15458503	0.51603559	-0.2658533
ENSG000001	SEMA6C	0.08683868	0.42885945	-0.2658492
ENSG000002	UQCRHL	0.02383278	0.29875884	-0.2657673
ENSG000001	FCGRT	0.00022053	0.18011717	-0.2657217
ENSG000001	PDE1A	0.52807037	0.80062313	-0.2654236
ENSG000001	KY	0.00328447	0.2114129	-0.2653928
ENSG000001	RFLNA	0.42112009	0.73521014	-0.2652107
ENSG00000(	ATP2C2	0.13825046	0.49898885	-0.2650123
ENSG000001	TANC2	0.02083249	0.28641584	-0.2648979

ENSG000002	TMEM269	0.10496134	0.45514301	-0.2646883
ENSG000001	BVES	0.55542683	0.8145753	-0.2644679
ENSG000001	HDC	0.33384926	0.67615222	-0.2643647
ENSG000001	EFEMP2	0.01039859	0.24174018	-0.26422
ENSG000001	C17orf64	0.57987596	0.82610777	-0.264201
ENSG000001	MAPK3	0.00261632	0.2093801	-0.2641956
ENSG000001	CLU	0.07666516	0.41092575	-0.2641411
ENSG000001	GPR143	0.21637818	0.58303866	-0.2639303
ENSG000001	CNDP1	0.61458173	0.84533626	-0.2636439
ENSG000001	XKRX	0.22583804	0.59319591	-0.2634608
ENSG000001	CBX2	0.12225144	0.47887061	-0.2634484
ENSG000001	PRAM1	0.00047426	0.18856694	-0.2634029
ENSG000001	TPTE2	0.56215947	0.81804833	-0.2632491
ENSG000001	CXCL1	0.0582594	0.37959785	-0.2632138
ENSG000001	ESAM	0.05263218	0.36895324	-0.2631939
ENSG000001	PTMS	0.02123175	0.28959078	-0.2631797
ENSG000001	HYAL3	0.00609614	0.22079879	-0.2631682
ENSG000001	ZC2HC1C	0.33639574	0.6782637	-0.2631376
ENSG000001	DNAJC28	0.4911057	0.78023514	-0.2630602
ENSG000001	PSMA8	0.56937367	0.82041807	-0.263016
ENSG000001	TUBA4A	0.00296058	0.20994948	-0.2629644
ENSG000001	CNRIP1	0.11657183	0.4718965	-0.2629398
ENSG000001	IL17REL	0.61347995	0.8451435	-0.2628929
ENSG000001	STK31	0.46480784	0.76408722	-0.2627759
ENSG000001	FBXO43	0.57333851	0.82244875	-0.2627013
ENSG00000C	PPP1R12B	0.02832114	0.31303636	-0.2626329
ENSG000001	GPT	0.30418982	0.65635638	-0.2625718
ENSG000001	SNAI1	0.39479292	0.72193673	-0.2623659
ENSG000001	PNPLA2	0.00794088	0.23002982	-0.2623336
ENSG000001	MYL4	0.08320568	0.42260872	-0.2617312
ENSG000001	CTSD	0.00156188	0.19292382	-0.2616602
ENSG000001	NPL	0.00578086	0.21912363	-0.2613836
ENSG00000C	ITGA8	0.63009599	0.85293934	-0.2612337
ENSG000001	KLHDC7A	0.5515203	0.81323014	-0.2612291
ENSG000002	N/A	0.42066764	0.7349809	-0.2612022
ENSG00000C	RAD54L	0.10981991	0.46255597	-0.2611752
ENSG000001	REPS2	0.00628021	0.22079879	-0.260928
ENSG000001	SAMD14	0.19044344	0.55565681	-0.2608687
ENSG000001	IFI27L2	0.6995568	0.88442761	-0.2606947
ENSG00000C	DAPK2	0.01435512	0.2599395	-0.260634
ENSG000001	KCNJ13	0.3499302	0.68928669	-0.2606179
ENSG000001	TDRD10	0.60999077	0.84290213	-0.2604505
ENSG000001	CDKL2	0.6161596	0.84569598	-0.2603145

ENSG000001	ARL3	0.02891508	0.31345545	-0.2602819
ENSG000001	PLK1	0.05461664	0.37354464	-0.2602539
ENSG000001	DPM2	0.04008672	0.34275311	-0.2602105
ENSG000001	SDK1	0.26612955	0.62892245	-0.2601757
ENSG000001	ZNF608	0.22096517	0.58835872	-0.2601574
ENSG000001	PROKR1	0.55466445	0.81429195	-0.2601371
ENSG000001	TMC3	0.6382486	0.8564206	-0.2600209
ENSG000001	LYPD2	0.26649307	0.62929726	-0.2596053
ENSG00000(	YPEL3	0.00503539	0.21833269	-0.2594284
ENSG000001	HASPIN	0.1796812	0.54477842	-0.259423
ENSG00000(	RAP1GAP	0.22032048	0.58779882	-0.2593461
ENSG00000(	ABHD5	0.00487933	0.21772133	-0.2590112
ENSG000001	SEC14L3	0.20902502	0.57301018	-0.2587996
ENSG000001	PER1	0.04780875	0.35895177	-0.2586985
ENSG00000(	KIF3C	0.02510792	0.30075912	-0.2585683
ENSG000001	AMN	0.15485237	0.51610961	-0.2584181
ENSG000001	NPRL3	0.07712388	0.41179232	-0.2581984
ENSG000001	ANO10	0.00154231	0.19292382	-0.2579122
ENSG000001	XCR1	0.40072587	0.72606468	-0.2577735
ENSG00000(	SLC11A1	0.01488447	0.26260224	-0.2577661
ENSG000002	SYCE1L	0.2720413	0.63323084	-0.2575848
ENSG000002	UBE2F-SCLY	0.65572174	0.86467033	-0.2575708
ENSG000001	ABLIM3	0.12233916	0.47887061	-0.2573254
ENSG000001	CARS1	0.11337724	0.46854321	-0.2572849
ENSG000002	LRRC37A2	0.37428973	0.70633918	-0.2570787
ENSG000002	C20orf202	0.61403224	0.8451435	-0.2566168
ENSG000002	NELFE	0.66357778	0.86832674	-0.256559
ENSG000001	GRAMD1C	0.24714949	0.61321492	-0.2564089
ENSG000001	ERN1	0.03415706	0.32634691	-0.2563972
ENSG000001	CAPN9	0.45850499	0.75804353	-0.2562422
ENSG000001	NLRP6	0.01052264	0.24200586	-0.2562349
ENSG00000(	CD82	0.00317377	0.21033652	-0.2561233
ENSG000001	S1PR4	0.00954475	0.23624711	-0.2558728
ENSG000002	N/A	0.59102619	0.83288424	-0.2558412
ENSG00000(	IL11	0.60349537	0.83883394	-0.2557859
ENSG000001	MAPK13	0.01042448	0.24174018	-0.2557603
ENSG000001	FAM9C	0.41906873	0.73426975	-0.255464
ENSG000001	UBXN6	0.0507241	0.36298244	-0.2551391
ENSG000001	ZYX	0.01241842	0.25464329	-0.2550968
ENSG000001	TMEM132D	0.19999585	0.56676738	-0.2548708
ENSG000002	TUBA4B	0.40012592	0.72574224	-0.2547524
ENSG000001	CAVIN3	0.30795023	0.65762361	-0.2546243
ENSG000001	ARHGEF37	0.22998408	0.59853365	-0.2544597

ENSG000002	TICAM2	0.4301494	0.74091639	-0.2544096
ENSG000002	C17orf107	0.27116763	0.63278941	-0.2543064
ENSG000001	TMCC2	0.07911657	0.41492758	-0.2542039
ENSG000001	SLC16A9	0.63003998	0.85293934	-0.2541355
ENSG000001	BMP6	0.03908658	0.33920759	-0.253896
ENSG00000C	PPEF1	0.13914076	0.49975817	-0.2538776
ENSG000001	RYR2	0.27422767	0.63454962	-0.2538728
ENSG000001	R3HDM4	0.02863784	0.31345545	-0.2537632
ENSG000001	CLVS1	0.52619738	0.80013737	-0.2537355
ENSG000001	CHRNA10	0.01135533	0.24556914	-0.253717
ENSG000001	ABAT	0.01049416	0.24174018	-0.2535518
ENSG000001	CYP1A1	0.56983469	0.82053972	-0.2535184
ENSG00000C	GUCY2C	0.03670996	0.33341755	-0.2534087
ENSG000001	ATP6V0C	0.22900729	0.59754644	-0.2533437
ENSG000001	ITGA5	0.00135429	0.18856694	-0.253296
ENSG00000C	CASS4	0.02464	0.29927225	-0.2532294
ENSG000001	UBE2C	0.04516626	0.35545023	-0.2530463
ENSG000001	IL4	0.46990182	0.7662622	-0.2530175
ENSG000001	CATIP	0.18407927	0.54950998	-0.2527235
ENSG000001	ERF	0.00365394	0.21208655	-0.2526312
ENSG000001	ABCC5	0.10429716	0.45468989	-0.2524789
ENSG000001	GABARAPL1	0.00193876	0.19438665	-0.2524185
ENSG000001	MAL	0.07158943	0.4024868	-0.2522055
ENSG000001	PNPLA1	0.01844971	0.2781279	-0.2521811
ENSG000001	NMNAT2	0.42558895	0.73817463	-0.2518415
ENSG000001	AMPD2	0.00621254	0.22079879	-0.2517733
ENSG000001	SNORC	0.00166804	0.19345748	-0.2515253
ENSG000002	WDR46	0.30182225	0.65561042	-0.2514914
ENSG000001	CYGB	0.46877103	0.76611043	-0.2514815
ENSG000001	C17orf99	0.27764637	0.63710347	-0.2514787
ENSG00000C	HOMER3	0.01908943	0.28068185	-0.2514123
ENSG000001	PAQR6	0.00138093	0.18856694	-0.2513248
ENSG000001	PIP5KL1	0.1151584	0.47087055	-0.2512925
ENSG000002	CCDC169	0.48472826	0.7769018	-0.2512517
ENSG000001	DIRAS1	0.20061972	0.56730451	-0.2512476
ENSG000001	APMAP	0.01789859	0.27425066	-0.2511169
ENSG000001	SLC16A5	0.00103457	0.18856694	-0.250792
ENSG000001	BNIPL	0.04438539	0.35466813	-0.2506106
ENSG00000C	ICAM3	0.0011592	0.18856694	-0.2504753
ENSG000001	IL1R1	0.01390341	0.25910565	-0.2504224
ENSG000001	SDR42E2	0.31388499	0.66197566	-0.2502841
ENSG000001	NCF4	0.0044987	0.21750928	-0.2499288
ENSG00000C	FKBP5	0.05520352	0.37354464	-0.2499003

ENSG000001	SPNS2	0.06822693	0.39743754	-0.2498693
ENSG000001	HOXB6	0.42699863	0.7392501	-0.2498542
ENSG000001	MTARC1	0.04557496	0.3556589	-0.2497616
ENSG000001	RUNDC3A	0.11668399	0.4721948	-0.2497381
ENSG000001	TGFB1I1	0.06687218	0.39524277	-0.2496492
ENSG000001	GATA2	0.34020537	0.68135285	-0.249462
ENSG000001	ADORA1	0.43877846	0.7460934	-0.2493681
ENSG000001	LMNA	0.03051888	0.3191802	-0.2492617
ENSG00000C	MAPK8IP2	0.24240287	0.60796025	-0.2491995
ENSG000001	AMH	0.41458192	0.73184916	-0.249193
ENSG000001	CCDC173	0.40623691	0.72748349	-0.2491227
ENSG000001	GJA4	0.51438342	0.79409791	-0.2490425
ENSG000001	FAM174A	0.01372697	0.25910565	-0.2489476
ENSG000001	ASIP	0.50607773	0.78874871	-0.2485601
ENSG000001	CYYR1	0.26038582	0.62385783	-0.2484412
ENSG000001	ABCA6	0.30315815	0.65593778	-0.2483868
ENSG000001	SCGB3A2	0.58395758	0.82875175	-0.2482983
ENSG000001	SESN2	0.00500253	0.21833269	-0.2482242
ENSG000001	ADAM19	0.02003822	0.28415764	-0.2481814
ENSG000001	ADRA2A	0.18728731	0.55140237	-0.248072
ENSG000002	MAGIX	0.00350204	0.21208655	-0.2478818
ENSG000001	MYH15	0.56640568	0.8197023	-0.2478676
ENSG000001	ANXA9	0.00446413	0.21750928	-0.2477483
ENSG000001	SHKBP1	0.00757393	0.22933195	-0.2477292
ENSG000001	CLEC9A	0.23235254	0.59960373	-0.2475724
ENSG000001	NKX3-1	0.02741106	0.3110643	-0.2475563
ENSG000001	RAB3D	0.00158143	0.19292382	-0.2472628
ENSG000001	ARHGEF17	0.13330992	0.49227955	-0.247197
ENSG00000C	SDK2	0.07252845	0.40400469	-0.2468644
ENSG000001	PTAFR	0.00348615	0.21208655	-0.2467854
ENSG000001	TBL1X	0.00457765	0.21750928	-0.2465226
ENSG000001	SPOCD1	0.09513499	0.44084098	-0.2463138
ENSG000001	RIBC1	0.25390074	0.61921212	-0.246227
ENSG000001	OLFML2A	0.08386978	0.42306421	-0.2456807
ENSG000001	CALD1	0.08980593	0.43377006	-0.2456264
ENSG000001	NCAPG	0.28171079	0.63946601	-0.2455886
ENSG000001	SAG	0.59371232	0.83456808	-0.2451469
ENSG000002	H4C15	0.60712944	0.84047751	-0.2451099
ENSG000002	MKRN2OS	0.20163631	0.56773237	-0.2451004
ENSG000001	CEP55	0.32433913	0.66992678	-0.2447281
ENSG00000C	NFIX	0.11481088	0.47052227	-0.2445888
ENSG000001	PAQR9	0.50277889	0.78624875	-0.2444533
ENSG000001	HOXA2	0.31954411	0.66652314	-0.2444452

ENSG000002	CCDC13	0.30915428	0.65901772	-0.244341
ENSG000001	RIPK4	0.50119515	0.7853582	-0.2442966
ENSG000001	TMEM184B	0.00528535	0.21833269	-0.2440957
ENSG000001	FAIM2	0.64490208	0.85991395	-0.2440408
ENSG000001	MPP1	0.02788192	0.31135087	-0.2439076
ENSG000001	MYO1F	0.00163229	0.19292382	-0.2438302
ENSG000001	EEF1D	0.41320334	0.73077485	-0.2437
ENSG000001	C4BPA	0.3446284	0.68522517	-0.2435099
ENSG000001	NPAS2	0.21625878	0.58303866	-0.2434619
ENSG000001	KCNK5	0.14441448	0.50298774	-0.2434139
ENSG00000(	LPAR2	0.00018499	0.18011717	-0.2434029
ENSG000001	CHRNE	0.07458806	0.4072247	-0.243026
ENSG000001	TTLL4	0.00281953	0.20994948	-0.2427601
ENSG000001	PRR7	0.01658624	0.27031359	-0.2427317
ENSG000001	GDPD3	0.0033985	0.2114129	-0.2426777
ENSG000002	ZNF488	0.48305263	0.77585394	-0.2426735
ENSG000002	EXOC3L2	0.18209962	0.54662721	-0.2424564
ENSG000001	MMEL1	0.6090592	0.84235896	-0.2423874
ENSG000002	LINGO3	0.03076389	0.31953017	-0.242286
ENSG000001	FAM86B1	0.44780045	0.7510408	-0.242278
ENSG000001	GIPC3	0.04273797	0.35185871	-0.2421174
ENSG000001	GMPR	0.1608284	0.52384569	-0.242055
ENSG000001	LGALS9C	0.17499562	0.53975461	-0.2420495
ENSG000002	SAMD13	0.64924419	0.86252724	-0.242
ENSG000001	SERP2	0.48603017	0.77752459	-0.2419276
ENSG000001	TRPM8	0.56281669	0.81833783	-0.2416392
ENSG000001	TAL1	0.05661719	0.37516454	-0.2414353
ENSG000001	ADIPOR1	0.08547489	0.42585327	-0.2413785
ENSG000001	SCAP	0.00458162	0.21750928	-0.241362
ENSG000002	MGAM	0.19632584	0.5638811	-0.2413347
ENSG000001	SLC8A3	0.18762949	0.55167498	-0.2409424
ENSG000001	CPNE2	0.01513906	0.26260224	-0.2404244
ENSG000001	CBR3	0.26310268	0.62659798	-0.2404128
ENSG000001	SELPLG	0.00563599	0.21875144	-0.2402327
ENSG000002	GPC2	0.05804232	0.37863925	-0.2399431
ENSG000002	TMEM213	0.58553362	0.82954508	-0.2396214
ENSG000001	OGDHL	0.52855007	0.80071625	-0.2395892
ENSG000001	B4GALNT3	0.04428452	0.35453012	-0.2395285
ENSG000001	GRM3	0.61388206	0.8451435	-0.2393604
ENSG000001	GLIS1	0.4262083	0.73856395	-0.2392748
ENSG000001	DDIT4	0.09786106	0.44463533	-0.2387582
ENSG000001	MICALL2	0.06392264	0.39019247	-0.2386651
ENSG000001	PTPRF	0.20757129	0.57126603	-0.2384135

ENSG000002	ECT2L	0.25347355	0.61917408	-0.2380529
ENSG000001	TLE3	0.0144022	0.2599395	-0.2380429
ENSG000001	FRAT1	0.00070836	0.18856694	-0.2379705
ENSG000001	LRRC4	0.03281567	0.32362351	-0.2378285
ENSG000001	MINDY1	0.00057059	0.18856694	-0.2377769
ENSG000001	TSPAN16	0.00124375	0.18856694	-0.2377201
ENSG000001	KREMEN1	0.27205878	0.63323084	-0.2376261
ENSG000001	CAP2	0.40137535	0.72606468	-0.2375493
ENSG000001	FLT3	0.13401431	0.4925946	-0.2375312
ENSG000002	LILRB3	0.01621016	0.26940057	-0.2375085
ENSG00000C	CALCR	0.46814333	0.76574496	-0.2374452
ENSG000001	UBALD1	0.00840474	0.23429041	-0.2374087
ENSG00000C	ASPM	0.24740078	0.61347686	-0.2373894
ENSG000001	PBX1	0.0787026	0.41446832	-0.2369465
ENSG000002	SCARF2	0.1128667	0.46777983	-0.2369214
ENSG000001	PPM1F	0.00843827	0.23429041	-0.2367648
ENSG000001	ZP3	0.01496195	0.26260224	-0.2367308
ENSG000001	GALR3	0.54125603	0.80757124	-0.2367012
ENSG000001	PLPP7	0.18096287	0.54594838	-0.2365782
ENSG000001	JDP2	0.00455566	0.21750928	-0.2364775
ENSG000001	F2	0.60205194	0.83845247	-0.2362856
ENSG000001	DYNC2H1	0.1824836	0.54678908	-0.2362216
ENSG000001	ZNF467	0.01258833	0.25512421	-0.2362216
ENSG000001	FUT7	0.00883011	0.23464493	-0.2361878
ENSG000001	MYRFL	0.42362779	0.73732623	-0.2361461
ENSG00000C	PRKCZ	0.01260995	0.25512421	-0.2361008
ENSG00000C	RRP12	0.01068669	0.24206404	-0.2359979
ENSG000001	CHST13	0.11617485	0.47160917	-0.2358641
ENSG000002	CORO7	0.23386403	0.60168758	-0.2358488
ENSG000001	CFAP74	0.48506126	0.77694975	-0.2358139
ENSG000002	SMIM22	0.52410286	0.79870477	-0.235503
ENSG000001	GUK1	0.05466018	0.37354464	-0.2354174
ENSG000001	MMP8	0.25245887	0.61827672	-0.2352592
ENSG000002	ANXA8	0.61912141	0.84711589	-0.2352261
ENSG000002	STK19	0.70140777	0.88518249	-0.2349589
ENSG000001	PLEKHG3	0.00135737	0.18856694	-0.2346118
ENSG000001	SDR16C5	0.63527901	0.85504577	-0.234413
ENSG000001	HJURP	0.32196975	0.66822528	-0.2344052
ENSG000001	C20orf144	0.62816615	0.8517356	-0.2343646
ENSG000001	SLPI	0.1349538	0.49418805	-0.2343138
ENSG000001	KIF27	0.1306921	0.48895106	-0.2342108
ENSG00000C	TYRO3	0.49630694	0.78193527	-0.2341742
ENSG000001	TSKS	0.25505996	0.61991532	-0.2341705

ENSG00000C	TMCC3	0.00378796	0.21208655	-0.2341054
ENSG000001	SNCA	0.2457706	0.61158271	-0.234062
ENSG000001	CRYBB3	0.55078807	0.81279159	-0.2338948
ENSG000001	KDM4B	0.0090995	0.23513527	-0.2338335
ENSG00000C	BCORL1	0.01969766	0.28263225	-0.2337862
ENSG000001	ERG	0.31930727	0.66648568	-0.2337251
ENSG000001	KCNK10	0.25881677	0.62248674	-0.2337165
ENSG000001	DTX4	0.02609882	0.30552739	-0.2337148
ENSG00000C	HEPH	0.55186452	0.81327139	-0.2335889
ENSG000001	TAGLN2	0.00642487	0.22290736	-0.2335712
ENSG000001	UCN	0.12305832	0.47985062	-0.2335474
ENSG000001	GDPD4	0.60009884	0.83754165	-0.2332715
ENSG000002	CCL4	0.37098833	0.70329686	-0.2330821
ENSG000001	ALDH3A1	0.60896	0.84233419	-0.2329779
ENSG00000C	NDST1	0.01135816	0.24556914	-0.2329149
ENSG000001	TSPAN5	0.09283623	0.43753271	-0.2328661
ENSG000001	EFNA1	0.30119034	0.65544978	-0.2328611
ENSG000001	GFOD2	0.00844921	0.23429041	-0.2327571
ENSG000001	LAMB2	0.01891111	0.27919309	-0.2326025
ENSG000001	GRK7	0.65658993	0.86503541	-0.2323292
ENSG000001	NACAD	0.50897467	0.79132674	-0.2322864
ENSG000001	SLC12A9	0.00389337	0.21403539	-0.2320015
ENSG000001	NLGN2	0.109621	0.46231587	-0.2319545
ENSG000001	PACS1	0.00334437	0.2114129	-0.231881
ENSG000001	SLC2A14	0.13937711	0.49975817	-0.2317798
ENSG000001	EPS15L1	0.00257595	0.2093801	-0.2316262
ENSG000001	LMOD2	0.61360494	0.8451435	-0.2315879
ENSG000001	TEK	0.36053939	0.69535995	-0.2314663
ENSG000001	CYTL1	0.3380522	0.67974658	-0.2314056
ENSG000001	FBXO7	0.09624082	0.44215472	-0.2311098
ENSG000001	FGF13	0.40456908	0.72672584	-0.231034
ENSG000001	SPARC	0.12752804	0.48584533	-0.2309689
ENSG000001	ARHGEF4	0.15966803	0.52219103	-0.2308652
ENSG000001	CD93	0.03449654	0.32831054	-0.2308389
ENSG000001	METTL27	0.34355234	0.68429197	-0.2308017
ENSG000001	ITGAX	0.00089665	0.18856694	-0.2307343
ENSG000001	C7orf61	0.09107476	0.43488031	-0.2305737
ENSG000001	CXCL8	0.41937008	0.73439517	-0.230427
ENSG000001	ITGB8	0.62593835	0.85056163	-0.2304133
ENSG000001	IKBIP	0.06371683	0.38981851	-0.2303037
ENSG00000C	MAP2K3	0.01696574	0.27193747	-0.2301037
ENSG000001	FTH1	0.0197149	0.28263225	-0.2300329
ENSG000001	MICAL2	0.0531215	0.36981204	-0.2297412

ENSG000001	SRPX	0.45073877	0.75341213	-0.2297323
ENSG00000C	SLC22A17	0.09696588	0.44311879	-0.2296935
ENSG000001	MELTF	0.2261047	0.59329927	-0.2295211
ENSG000001	AMBN	0.57226536	0.82182269	-0.2294872
ENSG000001	LRFN1	0.02756586	0.3113392	-0.2294569
ENSG000001	GAL3ST4	0.07743974	0.41266462	-0.2293915
ENSG000001	ATG2A	0.0322869	0.32360639	-0.22938
ENSG000001	RAB9B	0.43164959	0.74126857	-0.2293523
ENSG000001	KL	0.23238938	0.59960373	-0.2292111
ENSG000001	CDCA8	0.01044647	0.24174018	-0.2291639
ENSG000001	MYH9	0.05876439	0.38067583	-0.2290219
ENSG000001	AURKB	0.05839203	0.3800398	-0.2289961
ENSG000001	OSGIN1	0.02074393	0.28641584	-0.2288844
ENSG000001	MCHR1	0.4408124	0.74750808	-0.2287631
ENSG000001	ANGPT1	0.30567807	0.65701379	-0.2287599
ENSG000001	CARHSP1	0.01005808	0.24143268	-0.2287438
ENSG000001	N/A	0.01671212	0.2704224	-0.2286503
ENSG000001	MBOAT7	0.02505094	0.30062982	-0.2283223
ENSG000001	EGF	0.1408806	0.50051652	-0.228292
ENSG000001	SYTL4	0.1673647	0.53173243	-0.2281407
ENSG000002	ITGB3	0.16038937	0.52341736	-0.2281403
ENSG000001	CAGE1	0.64248294	0.85845915	-0.2281268
ENSG00000C	TLE2	0.17256843	0.53689236	-0.2280793
ENSG000001	CXCR1	0.01714852	0.27243096	-0.2280629
ENSG000001	SPATA6L	0.444978	0.74940749	-0.2280538
ENSG000001	ST20	0.07255496	0.40400469	-0.2279593
ENSG00000C	UPP2	0.60428929	0.83883394	-0.2278935
ENSG000001	ASB11	0.48330183	0.77585394	-0.2278619
ENSG000001	S100A11	0.01646953	0.27000801	-0.2278294
ENSG000001	ZFPM2	0.65000086	0.86293893	-0.2276866
ENSG000002	HBG1	0.53263258	0.80280163	-0.227676
ENSG000002	LTC4S	0.06527198	0.39169239	-0.2272611
ENSG000001	TMEM108	0.20135133	0.56773237	-0.2270688
ENSG000001	NFIC	0.00419083	0.21614597	-0.2269969
ENSG000001	TREML4	0.10108857	0.44917064	-0.226952
ENSG000001	IL20RB	0.18809308	0.5521164	-0.2269461
ENSG000001	CKAP2L	0.32410969	0.6699145	-0.2268868
ENSG000001	PDE2A	0.04613256	0.35738124	-0.2268838
ENSG000001	PCSK1N	0.16067382	0.52370945	-0.2266542
ENSG000001	TALDO1	0.00974404	0.23800279	-0.2264307
ENSG000001	CMTM5	0.10621993	0.4561473	-0.2264174
ENSG00000C	SMAP2	0.01518652	0.26260224	-0.2263175
ENSG000001	MASP1	0.56823092	0.82037292	-0.2260756

ENSG000001	MAEL	0.65048457	0.86318388	-0.2258241
ENSG00000(	CEACAM6	0.21483965	0.58042452	-0.225666
ENSG00000(	CHDH	0.17239554	0.53666665	-0.2256507
ENSG00000(	MECOM	0.58514027	0.82919554	-0.2256164
ENSG000001	PSMF1	0.04115735	0.34701527	-0.2254912
ENSG000001	AFF2	0.0407713	0.34551949	-0.2253139
ENSG00000(	SLC45A4	0.0031605	0.21033652	-0.2251795
ENSG000001	MGRN1	0.00237736	0.2093801	-0.225168
ENSG00000(	VSIG2	0.13152131	0.4894044	-0.2250429
ENSG00000(	CYP3A43	0.61515649	0.84533626	-0.2250255
ENSG000001	CABLES1	0.26543127	0.62834342	-0.2249206
ENSG000001	VIL1	0.17560746	0.54061531	-0.2247259
ENSG000002	CENPW	0.22941504	0.59783342	-0.2246554
ENSG000001	NKAIN2	0.57804078	0.82523365	-0.2246197
ENSG000002	TRMT9B	0.60485427	0.83913084	-0.2246076
ENSG000001	ALDOA	0.0286745	0.31345545	-0.2243869
ENSG000001	TREML1	0.11670368	0.4721948	-0.2243109
ENSG000002	C10orf105	0.0080629	0.23082837	-0.2243085
ENSG000001	CACHD1	0.16744152	0.53173243	-0.2242233
ENSG000001	C19orf67	0.59330577	0.83454291	-0.2242053
ENSG000001	SNAI3	0.00731727	0.22727528	-0.2241957
ENSG000001	PUSL1	0.00369832	0.21208655	-0.2241206
ENSG000001	ITPKA	0.59468543	0.83499718	-0.2240571
ENSG000001	PIEZO2	0.61085762	0.84334674	-0.2240418
ENSG000001	ODF3L2	0.51672483	0.79538014	-0.2239468
ENSG000001	MYO7B	0.01487031	0.26260224	-0.2238036
ENSG000001	IL1RL2	0.64454763	0.85980281	-0.2236805
ENSG000001	IL37	0.50206732	0.78558996	-0.2236654
ENSG000001	CXCR2	0.01316201	0.25748383	-0.2236526
ENSG000001	RGS14	0.01086995	0.24206404	-0.2232385
ENSG000001	KDM6B	0.03483996	0.32855052	-0.2231034
ENSG000001	GNA14	0.59823738	0.83653786	-0.2230127
ENSG000001	PIPOX	0.234468	0.60219185	-0.222806
ENSG00000(	GGT5	0.24095059	0.60721888	-0.2227694
ENSG000002	SMIM5	0.06673296	0.39496052	-0.2227291
ENSG000001	PLP1	0.58746862	0.83046863	-0.2226468
ENSG000001	IGFBP6	0.36012564	0.69520391	-0.2224182
ENSG000002	N/A	0.00524913	0.21833269	-0.2223347
ENSG000001	TKT	0.00169654	0.19438665	-0.2222436
ENSG000001	TESMIN	0.03827289	0.33650813	-0.222155
ENSG000002	TBC1D3F	0.48788507	0.7781719	-0.2220617
ENSG000001	TREM1	0.00782086	0.23002982	-0.2217155
ENSG000002	CENPS-COR	0.57737716	0.824709	-0.2216467

ENSG000001	RASL10A	0.30483315	0.65635638	-0.2214064
ENSG00000C	ST6GALNAC2	0.00940735	0.23551879	-0.2213787
ENSG000002	TBC1D3D	0.68774039	0.88051877	-0.2213354
ENSG000001	BPI	0.06197877	0.38624952	-0.2213343
ENSG000001	ADGRD1	0.25949628	0.6232076	-0.2211666
ENSG000002	HP	0.03908711	0.33920759	-0.2208746
ENSG000001	PODXL2	0.08955956	0.43356546	-0.2208616
ENSG000001	DOCK6	0.09902425	0.4460792	-0.2208024
ENSG000001	RTN3	0.00254842	0.2093801	-0.2207641
ENSG00000C	TBXA2R	0.06554276	0.39248431	-0.2206505
ENSG00000C	CABIN1	0.01591595	0.26742239	-0.2201888
ENSG00000C	EYA2	0.60200261	0.83845247	-0.2199026
ENSG000001	CRACDL	0.01399111	0.25910565	-0.219812
ENSG000001	BAIAP2L2	0.121601	0.47828106	-0.2197533
ENSG00000C	CTTNBP2	0.59556107	0.83572077	-0.2195054
ENSG000002	H2AC16	0.5737784	0.82271635	-0.2194011
ENSG000001	CPLX2	0.26240171	0.625825	-0.2193547
ENSG000001	ABCA10	0.49540847	0.78149688	-0.2193283
ENSG000001	DHRS7	0.00977692	0.23800279	-0.2192912
ENSG000001	GAS2L1	0.08570053	0.42602033	-0.2192759
ENSG000001	SLC25A10	0.30900705	0.65879037	-0.2190508
ENSG00000C	DGKG	0.05901888	0.38096018	-0.2190339
ENSG000001	ACOX1	0.00466954	0.21772133	-0.218935
ENSG000001	SERPINA9	0.59022323	0.83225833	-0.218847
ENSG000001	LRRC74A	0.66782169	0.87074767	-0.2187955
ENSG000001	SMIM10L2A	0.30002468	0.6544819	-0.2185323
ENSG000002	FOXO6	0.61506479	0.84533626	-0.2183886
ENSG000001	RRM2	0.37063917	0.7031833	-0.2183645
ENSG000001	SKI	0.04078758	0.34551949	-0.2181715
ENSG000001	EXTL1	0.61930284	0.84711589	-0.2181352
ENSG000001	ZNF516	0.03762173	0.33471496	-0.2181344
ENSG000001	CARM1	0.05572296	0.37502119	-0.2180346
ENSG000001	ZAR1L	0.53636205	0.80444134	-0.217954
ENSG000001	PRDM12	0.62031695	0.84781871	-0.2179428
ENSG000001	RASGRP4	0.0070621	0.22661511	-0.2177098
ENSG000001	LAMTOR4	0.00044319	0.18856694	-0.2176021
ENSG000001	CEP131	0.02872108	0.31345545	-0.2175227
ENSG000001	FPR1	0.01891436	0.27919309	-0.2174593
ENSG000001	ZER1	0.01671499	0.2704224	-0.2171477
ENSG000001	PBXIP1	0.00551848	0.21833269	-0.2171253
ENSG000001	HSD11B1L	0.16841625	0.53253292	-0.2170452
ENSG000001	COQ8A	0.00910677	0.23513527	-0.216985
ENSG000001	E2F1	0.17645452	0.54126676	-0.2169497

ENSG000001	CFAP94	0.13666045	0.49674408	-0.2169358
ENSG000001	SCN1B	0.05803485	0.37863925	-0.216913
ENSG000001	MSX1	0.51115319	0.79171125	-0.2166884
ENSG000001	PELI3	0.04485247	0.35545023	-0.216633
ENSG00000(	PLOD1	0.01237534	0.25464329	-0.2165103
ENSG000001	CCDC88B	0.00670461	0.22330866	-0.2164493
ENSG000001	LSP1	0.01440332	0.2599395	-0.21624
ENSG000001	MYO18B	0.52766723	0.80062313	-0.2161123
ENSG000001	FJX1	0.27834723	0.63759905	-0.2159512
ENSG000001	CDCA5	0.17486858	0.53975461	-0.2159499
ENSG000001	FCN2	0.60246112	0.83852406	-0.2156952
ENSG000001	IHO1	0.09828888	0.4448142	-0.2156471
ENSG000001	FRAT2	0.00206919	0.19943643	-0.2155994
ENSG000001	ZDHHC1	0.1986883	0.56598606	-0.2155945
ENSG000001	TMEM184A	0.53856667	0.80633537	-0.215589
ENSG000001	NR5A2	0.66620381	0.8698256	-0.2155326
ENSG00000(	RALBP1	0.00308602	0.20994948	-0.2155037
ENSG000001	CENPM	0.34077701	0.68167998	-0.2154601
ENSG000001	MIXL1	0.61126405	0.84339687	-0.2149242
ENSG000001	SPATA9	0.40276466	0.72672584	-0.21481
ENSG000001	PROSER2	0.08998681	0.43377006	-0.2146777
ENSG000001	ITGA7	0.09787114	0.44463533	-0.2141367
ENSG000001	SH3PXD2B	0.22194334	0.58912768	-0.2141275
ENSG000001	SHE	0.15731977	0.52024464	-0.2140895
ENSG00000(	ITGB5	0.1395708	0.49975817	-0.2139918
ENSG00000(	CREB3L3	0.69432881	0.8821445	-0.2139866
ENSG00000(	SUSD2	0.2426903	0.60820533	-0.2137788
ENSG000002	ASIC3	0.03947823	0.34078044	-0.2136757
ENSG000001	WNT4	0.16804175	0.53240547	-0.2136192
ENSG00000(	CLSPN	0.1113564	0.46472866	-0.2134569
ENSG000001	CHRDL1	0.58739802	0.83046863	-0.2132863
ENSG000002	N/A	0.66155126	0.86736018	-0.2132171
ENSG000002	BTBD18	0.23499575	0.60232491	-0.2127309
ENSG000001	AKR7A3	0.50392711	0.78683186	-0.2125561
ENSG000001	PLTP	0.03826631	0.33650813	-0.2124511
ENSG000001	SGCA	0.33797642	0.67967837	-0.2124379
ENSG000001	PLK3	0.01482393	0.26260224	-0.2123925
ENSG000001	GJC2	0.29065537	0.648324	-0.2123125
ENSG000001	SYPL2	0.64039198	0.85706063	-0.2122528
ENSG000001	TXNRD3	0.14564743	0.50423822	-0.2121256
ENSG000001	PADI2	0.07405814	0.40583259	-0.2120974
ENSG000001	QPRT	0.05478814	0.37354464	-0.2119926
ENSG000002	CASTOR1	0.30632748	0.65735608	-0.2119849

ENSG000000	MKNK2	0.00731135	0.22727528	-0.2119795
ENSG000001	SHROOM1	0.05614731	0.37502119	-0.2112245
ENSG000001	MYBPC3	0.00269209	0.2093801	-0.2111869
ENSG000001	LIPT2	0.27868266	0.63770725	-0.2111823
ENSG000001	H2BC11	0.06366308	0.38981851	-0.2111608
ENSG000001	ERGIC1	0.00453178	0.21750928	-0.2110558
ENSG000000	ZMYND10	0.07757171	0.41282593	-0.210824
ENSG000001	IGF2R	0.09837404	0.4448142	-0.2107908
ENSG000002	ORM2	0.03300286	0.32362351	-0.2107602
ENSG000001	ULBP3	0.58380831	0.82875175	-0.2106649
ENSG000001	DRICH1	0.23085261	0.59855451	-0.210549
ENSG000001	DNASE1L2	0.27420078	0.63454962	-0.2104742
ENSG000001	CKMT2	0.57125078	0.82159192	-0.2102779
ENSG000001	BBS1	0.09726854	0.44363742	-0.2100014
ENSG000001	SLC25A37	0.04321022	0.35247453	-0.2097422
ENSG000001	DUS2	0.01334232	0.25828987	-0.2095653
ENSG000001	TIMP1	0.0018524	0.19438665	-0.2095418
ENSG000001	DNM1	0.09661407	0.44276678	-0.2095001
ENSG000001	ZFR2	0.29524484	0.6512539	-0.2093501
ENSG000001	TMEM37	0.62407624	0.84973802	-0.2093386
ENSG000001	POR	0.00940699	0.23551879	-0.2092555
ENSG000001	UNC13C	0.63537574	0.85504577	-0.2091533
ENSG000001	NT5DC2	0.10948055	0.46193514	-0.2090901
ENSG000001	CTF1	0.28191823	0.63976328	-0.2090493
ENSG000001	RND2	0.5252271	0.79936321	-0.2088931
ENSG000001	PGLYRP4	0.61545823	0.84537475	-0.2088427
ENSG000002	PADI6	0.69013737	0.88074719	-0.2087556
ENSG000000	CTTN	0.19057267	0.55593398	-0.2086407
ENSG000001	SEMA4A	0.02439243	0.29927225	-0.2086388
ENSG000001	DYSF	0.23612101	0.60336718	-0.2084998
ENSG000002	MEIKIN	0.53007342	0.8016768	-0.2084233
ENSG000001	RILPL1	0.02347445	0.29875884	-0.2083602
ENSG000001	TPRG1L	0.00039085	0.18856694	-0.2083249
ENSG000001	TMCO3	0.04894386	0.360767	-0.2083107
ENSG000001	FRK	0.69354147	0.88183499	-0.2081623
ENSG000001	DUSP15	0.63133698	0.85334691	-0.2081597
ENSG000001	COL6A3	0.11201612	0.46600198	-0.2078889
ENSG000001	TMIGD3	0.08738849	0.42952109	-0.207884
ENSG000000	CYBRD1	0.15901985	0.5217239	-0.2077999
ENSG000001	RXRA	0.01045992	0.24174018	-0.2077323
ENSG000001	GBA	0.62646208	0.85068296	-0.2073897
ENSG000001	ADAP1	0.0164932	0.27000801	-0.2073603
ENSG000001	AVIL	0.01504164	0.26260224	-0.2073576

ENSG000001	GPR157	0.06384252	0.38992693	-0.2071875
ENSG000001	MAP6D1	0.4575773	0.75743527	-0.2070453
ENSG000001	QSOX1	0.0079707	0.23002982	-0.2068278
ENSG000001	SYT17	0.18765172	0.55167498	-0.2068253
ENSG000001	POLL	0.045063	0.35545023	-0.2067188
ENSG000002	TCEA3	0.1579686	0.52077869	-0.2067162
ENSG000001	KCNK7	0.13422757	0.49308892	-0.2065683
ENSG00000C	KCNH4	0.57044189	0.82090445	-0.2064884
ENSG000001	NINJ1	0.00259879	0.2093801	-0.2062373
ENSG000001	PCLO	0.58207798	0.82746456	-0.206108
ENSG000001	ESPNL	0.36042623	0.69534637	-0.2059616
ENSG000001	P2RX1	0.00024159	0.18011717	-0.2057963
ENSG000001	CD68	0.514717	0.79420539	-0.2057949
ENSG000001	TBX20	0.53540691	0.80413676	-0.2057233
ENSG000001	OPRD1	0.22316557	0.59081204	-0.2056803
ENSG000001	TOM1L2	0.019585	0.28172722	-0.2055361
ENSG000001	CENPF	0.18141878	0.54626754	-0.2054988
ENSG000001	TNNI2	0.0167337	0.2704224	-0.2054338
ENSG00000C	EXTL3	0.00638195	0.22284683	-0.2054132
ENSG000002	PCP4L1	0.2009643	0.56770499	-0.2051652
ENSG000001	MOCS1	0.29536452	0.6512539	-0.2051359
ENSG000002	B3GNT9	0.01976137	0.28270071	-0.2050623
ENSG000001	TNFRSF10C	0.0272104	0.30967858	-0.2048348
ENSG000001	MS4A3	0.37216108	0.70428665	-0.2047687
ENSG000001	SLC6A4	0.1292336	0.4871968	-0.2047111
ENSG00000C	NEXMIF	0.59628142	0.83586476	-0.2046105
ENSG000001	ABHD4	0.0028778	0.20994948	-0.2044099
ENSG000001	SRRM3	0.56909262	0.82037292	-0.2043919
ENSG000001	CLIP3	0.07091994	0.40178892	-0.2040641
ENSG000001	SH3BP5L	0.00470333	0.21772133	-0.2038796
ENSG000001	AQP7	0.38771822	0.71668724	-0.203821
ENSG000002	HMBS	0.0356913	0.33014896	-0.2037449
ENSG000001	RAB13	0.16738591	0.53173243	-0.2036843
ENSG000001	TFEB	0.00409959	0.21403539	-0.2035443
ENSG000001	FABP5	0.17694147	0.54177807	-0.2035201
ENSG00000C	RRAGD	0.0054206	0.21833269	-0.2034869
ENSG000001	SFXN5	0.03469662	0.32831054	-0.2033686
ENSG000001	SCNN1D	0.17160492	0.53577669	-0.2032095
ENSG000002	PPIAL4A	0.6240625	0.84973802	-0.2031629
ENSG00000C	SLC9A1	0.01553391	0.26471158	-0.2028499
ENSG000001	CNMD	0.65498213	0.86442301	-0.2028376
ENSG000001	HEPACAM2	0.50685703	0.7896601	-0.2027259
ENSG000001	PLCD1	0.02451	0.29927225	-0.2027182

ENSG000001	FOS	0.05670098	0.37516454	-0.2026624
ENSG000002	N/A	0.4951027	0.78124152	-0.2026095
ENSG000001	GNAZ	0.19943782	0.56600138	-0.2024979
ENSG000001	PLXNB1	0.1670948	0.53159417	-0.2024692
ENSG000001	DYRK1B	0.00680872	0.22347774	-0.2022873
ENSG000001	AKAP12	0.45336572	0.75477115	-0.2022021
ENSG00000(	DLEC1	0.19516638	0.56280385	-0.2021166
ENSG000001	MLLT1	0.01551533	0.26471158	-0.2020944
ENSG000001	NELL2	0.21897537	0.58594317	-0.2020403
ENSG000001	OVCH1	0.5011724	0.7853582	-0.2017229
ENSG000001	RNASE6	0.09453354	0.43981122	-0.2016711
ENSG000001	MID1IP1	0.01266315	0.25521652	-0.2016497
ENSG000001	ZFHX2	0.17931027	0.54453715	-0.2016483
ENSG000001	UTS2B	0.5108822	0.79160171	-0.2014318
ENSG000001	DUOX2	0.65870513	0.86560481	-0.2014296
ENSG000001	HPX	0.30610324	0.65735608	-0.2014146
ENSG000002	PVRIG	0.3247567	0.67036289	-0.2013764
ENSG000001	DGAT1	0.0037565	0.21208655	-0.2012535
ENSG00000(	NEDD4L	0.07054173	0.40156223	-0.2011991
ENSG000001	ZMYND15	0.09293181	0.43758758	-0.2011313
ENSG000001	VNN2	0.04955734	0.36137351	-0.2008592
ENSG000001	NQO2	0.01322274	0.25805013	-0.2008128
ENSG000001	ZWINT	0.22243818	0.58995895	-0.2008038
ENSG000001	IGF1R	0.12102837	0.47724625	-0.2007589
ENSG000001	DUSP1	0.02584716	0.30449397	-0.2007208
ENSG000001	CIDEC	0.63525938	0.85504577	-0.2007124
ENSG00000(	NAT14	0.06567057	0.39264465	-0.2006116
ENSG000001	CDH20	0.56284597	0.81833783	-0.2002757
ENSG00000(	ACTN1	0.03336997	0.32398208	-0.2002614
ENSG000001	KCNJ9	0.65528583	0.86446832	-0.2002413
ENSG000002	CORT	0.52715048	0.80038735	-0.2000987
ENSG00000(	CERS4	0.07547869	0.40974508	-0.199956
ENSG000001	PGAP6	0.01037723	0.24174018	-0.1999557
ENSG000001	CSF3R	0.00121283	0.18856694	-0.1999462
ENSG000001	GATA1	0.15396201	0.51533385	-0.1999344
ENSG000001	GJB6	0.5562814	0.81507187	-0.1998269
ENSG000001	PFKFB4	0.01708135	0.27224356	-0.1997833
ENSG000001	MPPE1	0.01330081	0.25828987	-0.1997807
ENSG000001	CLGN	0.70745161	0.88887192	-0.1997663
ENSG00000(	DTX2	0.02845163	0.31345545	-0.1995626
ENSG000002	TGFBR3L	0.56363765	0.81863326	-0.1995071
ENSG000001	F12	0.05638177	0.3750908	-0.1994762
ENSG000001	WDR87	0.5994597	0.83721204	-0.1993116

ENSG000001	HSD11B2	0.45710576	0.75734179	-0.199276
ENSG000002	DEFA1B	0.69490371	0.88222003	-0.1990593
ENSG000001	C21orf58	0.03262302	0.32362351	-0.1989581
ENSG000001	CAMK2G	0.01018597	0.24174018	-0.1989575
ENSG000001	PHKG1	0.0600181	0.38277429	-0.1987276
ENSG000001	IGSF11	0.65855933	0.86555188	-0.1986766
ENSG000001	S100A12	0.14235107	0.50068215	-0.19867
ENSG000001	CABP4	0.06682394	0.3951276	-0.1984133
ENSG000002	RING1	0.73723113	0.90247451	-0.1983905
ENSG000001	TSC22D3	0.01046837	0.24174018	-0.1982452
ENSG000002	KRTAP10-9	0.50131687	0.7853582	-0.1981831
ENSG00000C	QPCTL	0.08899753	0.43316932	-0.1980445
ENSG000001	VIPR1	0.10798523	0.45959534	-0.1979448
ENSG000001	PRDM7	0.65030282	0.86308377	-0.1978476
ENSG00000C	NME1-NME2	0.68742997	0.88047649	-0.1977469
ENSG000001	MFGE8	0.21312597	0.57868334	-0.1977266
ENSG000001	FAM178B	0.41263253	0.73063968	-0.1976691
ENSG000001	EFHC2	0.23686394	0.60364584	-0.1976485
ENSG000001	RASSF2	0.0055988	0.21875144	-0.1976136
ENSG000001	THRA	0.08506209	0.42526981	-0.1976083
ENSG000001	LEMD1	0.56561728	0.81931223	-0.197594
ENSG000001	TAF4	0.11913808	0.47578591	-0.1973408
ENSG000001	NXN	0.14452916	0.50315089	-0.1973028
ENSG000001	NOG	0.2566701	0.62074989	-0.1972702
ENSG000001	GINS2	0.33252482	0.67523724	-0.1972627
ENSG000001	PCOLCE	0.10810042	0.45960368	-0.1972553
ENSG000001	FGF17	0.59145066	0.83319316	-0.197246
ENSG000001	STYXL2	0.5629128	0.81833783	-0.1971668
ENSG000001	ABHD12B	0.04665509	0.35791197	-0.1971339
ENSG000001	N4BP3	0.21278861	0.5782114	-0.1971243
ENSG000001	SULT1B1	0.12426804	0.48162293	-0.1971185
ENSG000001	TEPP	0.39478449	0.72193673	-0.1968963
ENSG000001	RFX1	0.02131774	0.29038271	-0.1968125
ENSG000001	SHROOM4	0.33545961	0.67708232	-0.1967202
ENSG000001	EFCAB12	0.10049798	0.44829276	-0.1967
ENSG000001	DHRS13	0.10275069	0.45220348	-0.1966391
ENSG000001	SLC6A19	0.66092365	0.86725532	-0.196556
ENSG000001	DEPDC7	0.36578588	0.69837111	-0.1963839
ENSG000002	GPR162	0.07588616	0.41015711	-0.1963265
ENSG000002	SERPINB10	0.40181484	0.72645225	-0.1962995
ENSG000001	SLX1B	0.54665047	0.81063154	-0.1962046
ENSG000001	CCDC9B	0.0823808	0.42168296	-0.1961992
ENSG000001	FAM53C	0.00491118	0.21833269	-0.1961289

ENSG000001	AMDHD2	0.02254943	0.2943208	-0.1960378
ENSG000001	VSIR	0.00660496	0.22296216	-0.1960051
ENSG000002	H2AC8	0.23080531	0.59855451	-0.1959457
ENSG000000	ABCA7	0.00514717	0.21833269	-0.195871
ENSG000001	PRDM16	0.69007057	0.88074719	-0.1958272
ENSG000001	HRH2	0.0292843	0.31531118	-0.1955691
ENSG000001	CNPY3	0.00115875	0.18856694	-0.1953762
ENSG000001	ABCG2	0.40300471	0.72672584	-0.195115
ENSG000001	CFAP92	0.02659288	0.30699826	-0.1949271
ENSG000000	TMEM40	0.13391531	0.4925946	-0.1949204
ENSG000001	UTS2R	0.71433699	0.89175724	-0.1947408
ENSG000002	LTB4R	0.00145413	0.19195721	-0.194502
ENSG000001	PCSK6	0.15189955	0.51296214	-0.1943964
ENSG000001	RGL4	0.02468539	0.29927225	-0.1943859
ENSG000001	ITGAM	0.01030319	0.24174018	-0.1943662
ENSG000001	PPDPF	0.05754132	0.3770373	-0.1943338
ENSG000001	LTBP2	0.11482492	0.47052227	-0.1943268
ENSG000001	ST3GAL4	0.03489952	0.3286911	-0.194262
ENSG000001	CHST15	0.00372421	0.21208655	-0.1941967
ENSG000000	SBNO2	0.42985049	0.74091639	-0.1940923
ENSG000001	ELL	0.0057895	0.21912363	-0.1936443
ENSG000001	CCDC68	0.60467601	0.83901251	-0.1936058
ENSG000001	KRT19	0.51480428	0.79420539	-0.1935994
ENSG000001	GRIK5	0.26364952	0.62687307	-0.1935341
ENSG000002	CELA2B	0.49081823	0.7800506	-0.1935081
ENSG000002	ZNF865	0.02473512	0.29927285	-0.1934812
ENSG000000	VNN3	0.03532138	0.32934644	-0.1934575
ENSG000002	N/A	0.42982623	0.74091639	-0.1933823
ENSG000001	SLC25A29	0.12219861	0.47887061	-0.1933661
ENSG000001	NPIPA5	0.69828951	0.8835822	-0.193325
ENSG000002	CAPN14	0.54702093	0.81091746	-0.1933051
ENSG000001	PLAC9	0.07342897	0.40497004	-0.1932773
ENSG000001	COMTD1	0.11393032	0.46950297	-0.1932655
ENSG000001	TRIM8	0.00251577	0.2093801	-0.1931599
ENSG000000	IGF2BP2	0.21399942	0.5797802	-0.1931045
ENSG000002	DISP3	0.57557157	0.82346952	-0.1930856
ENSG000001	ZNF610	0.42960778	0.74091639	-0.193081
ENSG000000	UNC13D	0.00971149	0.23800279	-0.1929791
ENSG000001	TGFA	0.09354634	0.4384324	-0.1929072
ENSG000001	TMEM205	0.01900408	0.28000836	-0.1928947
ENSG000000	RTN4R	0.21490042	0.58049213	-0.1927711
ENSG000001	P3H3	0.20783472	0.57145912	-0.1924314
ENSG000001	MPZL3	0.05192861	0.36627491	-0.1923025

ENSG000002	MRPL23	0.1100167	0.46261866	-0.1921995
ENSG000001	HPCAL1	0.00036046	0.18856694	-0.1919975
ENSG000001	DYNLT4	0.2745401	0.63464829	-0.1918065
ENSG000001	ZBED3	0.03527521	0.32934644	-0.191681
ENSG000001	FCER1A	0.42194484	0.73601655	-0.1916572
ENSG000001	SHARPIN	0.03694046	0.33341755	-0.1915549
ENSG000001	DPYSL3	0.65734921	0.86522797	-0.1914438
ENSG000001	PRPH2	0.18539375	0.55033096	-0.1913476
ENSG000001	SARS2	0.22290364	0.5903895	-0.191208
ENSG000001	ARHGAP9	0.00209293	0.19943931	-0.1911684
ENSG000001	LYNX1	0.04321411	0.35247453	-0.1910381
ENSG000001	JUN	0.08331707	0.42260872	-0.1909696
ENSG000001	NAT8L	0.36408264	0.6973647	-0.190958
ENSG00000C	PRKACA	0.0062266	0.22079879	-0.1909304
ENSG00000C	SPI1	0.05708238	0.37665007	-0.1909243
ENSG000001	PTOV1	0.03469262	0.32831054	-0.1908848
ENSG000002	TMEM211	0.66112824	0.86736018	-0.1904846
ENSG000001	TMEM121	0.29953077	0.6542516	-0.1903946
ENSG000002	SMIM6	0.45757398	0.75743527	-0.190292
ENSG000001	AMBP	0.55166081	0.81323014	-0.1902211
ENSG000001	HDAC5	0.00669843	0.22330866	-0.1900267
ENSG00000C	MAOB	0.65837987	0.86550135	-0.1899957
ENSG000001	KIF15	0.37572813	0.70743725	-0.1899747
ENSG000001	C3orf62	0.00162261	0.19292382	-0.1899487
ENSG000002	FAM95C	0.25150089	0.6170914	-0.189901
ENSG000001	ERICH6B	0.50758596	0.79049235	-0.1898436
ENSG000001	DCAF12	0.19531931	0.56280385	-0.1898186
ENSG000001	DNASE1L3	0.34491204	0.68522517	-0.1898036
ENSG000002	NCR3	0.50152964	0.7853582	-0.1896729
ENSG000001	KCNMB4	0.29117225	0.64879427	-0.1894437
ENSG00000C	HAGH	0.10227005	0.45160695	-0.1894412
ENSG000001	RNPEPL1	0.02056278	0.28641584	-0.189311
ENSG000001	MAP1S	0.04692889	0.35811287	-0.1893104
ENSG000001	HPCAL4	0.30005317	0.6544819	-0.1892359
ENSG000001	PROK2	0.16870154	0.53281598	-0.1890523
ENSG000002	AKT1S1	0.02253403	0.2943208	-0.1890312
ENSG000001	FOXI1	0.68134456	0.87687561	-0.188996
ENSG00000C	MLF2	0.00533387	0.21833269	-0.1889842
ENSG000002	NT5M	0.15679367	0.51959295	-0.1889269
ENSG000001	NIBAN1	0.11613451	0.47160917	-0.1889104
ENSG000001	KRT72	0.26699311	0.62929976	-0.1887763
ENSG000001	ARID3A	0.0141626	0.25910565	-0.1885834
ENSG000001	SLC27A2	0.27397019	0.63422498	-0.1885203

ENSG000001	PREX1	0.0367746	0.33341755	-0.1884833
ENSG000001	CERS3	0.55170537	0.81323014	-0.1884177
ENSG000001	TREML2	0.03293176	0.32362351	-0.1881072
ENSG000001	LTBP3	0.13395757	0.4925946	-0.1880375
ENSG000001	UBB	0.10034435	0.4480999	-0.1879866
ENSG000001	NBEAL2	0.01836732	0.27758038	-0.1878289
ENSG000001	CXXC4	0.52697016	0.80038735	-0.1877976
ENSG000001	STON2	0.09394558	0.43929464	-0.1876967
ENSG00000C	MT3	0.64530392	0.8599145	-0.1876
ENSG000001	HPD	0.21011263	0.57424655	-0.187537
ENSG000001	NOMO3	0.63982737	0.85695596	-0.187432
ENSG000001	SIGLEC15	0.20320384	0.56867381	-0.1871955
ENSG000002	H2AC14	0.64769199	0.86142128	-0.1871604
ENSG00000C	MGLL	0.16689765	0.53125214	-0.1871345
ENSG000001	CLEC18A	0.16181076	0.52436829	-0.1870987
ENSG000001	OR6N2	0.70165459	0.88523111	-0.1868609
ENSG000001	CDH4	0.5557385	0.81461822	-0.1868321
ENSG000001	CABP7	0.20473471	0.56891466	-0.1867705
ENSG000001	MAP1A	0.15895225	0.5217239	-0.1867643
ENSG00000C	CDK11A	0.01839481	0.27758038	-0.1866541
ENSG000001	MPST	0.02593615	0.30450132	-0.1866387
ENSG000001	CCNB1	0.13894321	0.49945118	-0.1865461
ENSG000001	ADGRV1	0.30490799	0.65635638	-0.1865289
ENSG000001	REM2	0.00976996	0.23800279	-0.1864599
ENSG000001	KLF16	0.01057872	0.24206404	-0.1863483
ENSG000001	FADD	0.01827783	0.27758038	-0.1862317
ENSG00000C	RGS11	0.66133918	0.86736018	-0.1861957
ENSG000001	CLEC4E	0.06302941	0.38853777	-0.1860273
ENSG000001	SLC22A14	0.55235132	0.81369338	-0.1859971
ENSG000001	CFAP251	0.14299741	0.5010464	-0.1858711
ENSG000001	CTDSPL	0.24057735	0.60721888	-0.185848
ENSG000001	TNN	0.60293403	0.83860201	-0.1858384
ENSG000001	AQP10	0.20609373	0.56956674	-0.1857976
ENSG00000C	EHD3	0.05578663	0.37502119	-0.1857761
ENSG00000C	EVC	0.32727641	0.67217574	-0.1857223
ENSG000001	TMEM63B	0.04463521	0.35527495	-0.1856992
ENSG000001	MTUS2	0.53551534	0.80413676	-0.1856657
ENSG000001	HIP1	0.01836365	0.27758038	-0.1856321
ENSG000001	C5orf47	0.70117822	0.88513659	-0.1855943
ENSG000001	NMB	0.23714712	0.60391434	-0.1854826
ENSG000001	ELOF1	0.01367865	0.25910565	-0.1854507
ENSG000002	GPX1	0.09422756	0.43939485	-0.1853903
ENSG00000C	SLC2A3	0.06437092	0.39043354	-0.1853543

ENSG000001	E2F8	0.46460596	0.76392983	-0.1852827
ENSG000001	ASPH	0.16167159	0.52427832	-0.185274
ENSG000001	CDK5R1	0.14600843	0.50430399	-0.1852651
ENSG000001	DNAH12	0.35959075	0.69484359	-0.1851787
ENSG000001	GAA	0.0149336	0.26260224	-0.1849744
ENSG000001	PRDX6	0.03823201	0.33650813	-0.1847944
ENSG000001	THBS3	0.00567291	0.21875144	-0.184735
ENSG000001	ISM1	0.40961453	0.72910668	-0.1845406
ENSG000002	ZNF726	0.37699013	0.70855293	-0.1845368
ENSG000001	CASC3	0.00184755	0.19438665	-0.1845092
ENSG000001	RNF166	0.01022294	0.24174018	-0.1844297
ENSG000002	PISD	0.0015059	0.19292382	-0.1843688
ENSG000001	BAG1	0.125895	0.48382419	-0.1843374
ENSG000001	EVA1B	0.11623041	0.47160917	-0.1842848
ENSG000001	PRDX2	0.10203824	0.45141805	-0.1841572
ENSG000001	CHST8	0.60450188	0.83891427	-0.1841042
ENSG000001	RASIP1	0.66065745	0.86709953	-0.1839437
ENSG000001	RTN2	0.03326913	0.32398208	-0.1839372
ENSG000001	PHACTR1	0.06778257	0.39689346	-0.1839086
ENSG000001	DOC2A	0.59232622	0.83392318	-0.1838653
ENSG000001	FBXL13	0.15280011	0.51356145	-0.1837994
ENSG000001	FBLN5	0.29408694	0.65044158	-0.1837517
ENSG000001	B3GNTL1	0.02373152	0.29875884	-0.1837485
ENSG000001	ORAI3	0.00365133	0.21208655	-0.1835706
ENSG000001	IQCE	0.00305905	0.20994948	-0.1835394
ENSG00000C	ARHGAP33	0.08917464	0.43342334	-0.1835288
ENSG000001	CHST11	0.00671635	0.22330866	-0.1835239
ENSG000001	CCDC34	0.3227476	0.66859566	-0.1834803
ENSG000001	THEMIS	0.59927308	0.83716743	-0.1832841
ENSG000002	CROCC2	0.25562425	0.62030905	-0.1830231
ENSG000001	NME4	0.07156029	0.4024868	-0.1829096
ENSG000001	GPR27	0.12534637	0.48335545	-0.1828722
ENSG000001	NOXA1	0.16239423	0.52515336	-0.1827066
ENSG000001	ABCG1	0.11487037	0.47052227	-0.182602
ENSG000001	MATN2	0.37000009	0.70295438	-0.182549
ENSG000001	NEK10	0.51174255	0.79195232	-0.1825449
ENSG000001	C12orf40	0.70184368	0.88525098	-0.1825396
ENSG000001	ADAD2	0.69050084	0.88086598	-0.1825037
ENSG00000C	NAV3	0.34133444	0.68254277	-0.1824459
ENSG000001	WDR45	0.00538214	0.21833269	-0.1824278
ENSG000001	MYEOV	0.31385537	0.66197566	-0.1824275
ENSG000001	MPL	0.17184068	0.53586381	-0.1822909
ENSG000001	SIRPA	0.01287865	0.25583328	-0.1822136

ENSG000001	CPD	0.12417887	0.48156016	-0.1822006
ENSG000001	PLPP3	0.63097434	0.85326916	-0.1821632
ENSG000001	TNNT3	0.20452651	0.5688754	-0.1820536
ENSG000001	TUBB4A	0.32760164	0.67217574	-0.1820352
ENSG000001	ACSS3	0.23649637	0.60364584	-0.1819278
ENSG000001	UBE4B	0.00855889	0.23436355	-0.1819208
ENSG000001	LIN7B	0.17316091	0.53759345	-0.1815637
ENSG000001	UNC5B	0.55877556	0.81634324	-0.181534
ENSG000001	SLCO2B1	0.71152077	0.8906831	-0.1815226
ENSG000002	ZDHHC18	0.0104962	0.24174018	-0.181423
ENSG000001	ATP6V0B	0.00116582	0.18856694	-0.1812749
ENSG000001	VPS37B	0.01117384	0.2451811	-0.181185
ENSG000001	SLC16A11	0.4040813	0.72672584	-0.1811047
ENSG000001	PLGLB2	0.43779974	0.74549116	-0.1810359
ENSG000000	KCNQ1	0.01433827	0.2599395	-0.1810025
ENSG000001	SIRPB2	0.00099433	0.18856694	-0.1809979
ENSG000001	GPAT3	0.05319625	0.36981204	-0.1809941
ENSG000000	LRCH4	0.00552371	0.21833269	-0.1807557
ENSG000002	PRKDC	0.04082841	0.34551949	-0.180604
ENSG000002	H3C10	0.16556463	0.52885285	-0.1805143
ENSG000001	RAB17	0.65601485	0.86472748	-0.1805068
ENSG000002	FIGNL2	0.39110684	0.71964297	-0.1803028
ENSG000001	SPEF1	0.72558629	0.89686038	-0.1803001
ENSG000002	RGL2	0.70177341	0.88523111	-0.1801864
ENSG000002	FNDC10	0.16885647	0.53296205	-0.1801731
ENSG000001	ADAM8	0.03453154	0.32831054	-0.1801367
ENSG000001	SLC43A1	0.0517243	0.36546885	-0.1800942
ENSG000000	CNN2	0.0121124	0.25234748	-0.179986
ENSG000001	TNFSF14	0.0087533	0.23436355	-0.1797597
ENSG000001	TBC1D14	0.10493866	0.45514301	-0.1797554
ENSG000000	IDS	0.02475898	0.2993385	-0.17972
ENSG000001	SVIL	0.03664843	0.33341755	-0.1796287
ENSG000001	TLCD4	0.2280816	0.59645048	-0.1795808
ENSG000001	TPD52L2	0.00017252	0.18011717	-0.1795457
ENSG000001	SEC14L1	0.04527531	0.35545023	-0.1795006
ENSG000001	SLC26A11	0.18614108	0.55069275	-0.1794912
ENSG000001	MBD6	0.0047627	0.21772133	-0.1794769
ENSG000001	GRPR	0.59821388	0.83653786	-0.1794672
ENSG000001	CYP2S1	0.12508972	0.48290103	-0.1793877
ENSG000000	PYGM	0.01241412	0.25464329	-0.179336
ENSG000001	TYK2	0.00536811	0.21833269	-0.1792984
ENSG000001	MTX1	0.00982241	0.23839521	-0.1792131
ENSG000001	PRKN	0.43684698	0.74497757	-0.1790826

ENSG000001	PTGDS	0.2486635	0.61459845	-0.1790549
ENSG000001	STK40	0.00539543	0.21833269	-0.1790175
ENSG000001	FOXM1	0.2304623	0.59855451	-0.1790015
ENSG000001	TNIP1	0.0104678	0.24174018	-0.1788019
ENSG000002	CFAP97D2	0.40947989	0.72902686	-0.1787675
ENSG000001	SLC45A3	0.38379359	0.7123445	-0.1785366
ENSG000002	N/A	0.54997957	0.81218902	-0.1785153
ENSG000001	FGD3	0.0005648	0.18856694	-0.1784779
ENSG000002	N/A	0.49611436	0.78185081	-0.1784509
ENSG000001	DSG3	0.75361703	0.90943737	-0.1784113
ENSG000001	IFITM2	0.12394429	0.48115857	-0.1783693
ENSG000001	PTPRS	0.09580611	0.44171811	-0.1783662
ENSG000001	CAND2	0.48097117	0.77410996	-0.1782973
ENSG000002	INF2	0.09334945	0.43819457	-0.1780934
ENSG000001	TMX4	0.04910476	0.360767	-0.1780905
ENSG00000C	FTL	0.02073839	0.28641584	-0.1779511
ENSG000001	PSTPIP1	0.00800436	0.23002982	-0.1779211
ENSG00000C	RNASET2	0.00867107	0.23436355	-0.1778854
ENSG000001	RFNG	0.04556273	0.3556589	-0.1778671
ENSG000002	LAYN	0.43858484	0.74601932	-0.1778565
ENSG00000C	MYO9B	0.00732062	0.22727528	-0.1778199
ENSG000001	CBX7	0.02061569	0.28641584	-0.1777806
ENSG000001	MAPK10	0.53144701	0.80232066	-0.1777032
ENSG000001	PCSK1	0.65257968	0.86379349	-0.1776203
ENSG000001	ZNF185	0.017226	0.27243096	-0.1776031
ENSG000001	KRT18	0.3550896	0.6919142	-0.177523
ENSG000002	ZBTB8B	0.61462671	0.84533626	-0.1774916
ENSG000001	DUOXA2	0.50863117	0.79113366	-0.1773507
ENSG00000C	SNAP91	0.61639002	0.84579775	-0.177301
ENSG000001	ST6GALNAC4	0.15164178	0.51274628	-0.1770816
ENSG000002	AS3MT	0.51664941	0.79537655	-0.1769359
ENSG000001	FURIN	0.04489394	0.35545023	-0.1768132
ENSG000001	ZNF692	0.0487527	0.360767	-0.17678
ENSG000001	ESCO2	0.46910508	0.76620653	-0.1767417
ENSG000001	CBY2	0.71427681	0.89175724	-0.1767416
ENSG00000C	MARK4	0.01016849	0.24174018	-0.176709
ENSG000002	RFPL4AL1	0.53443967	0.80401157	-0.1766672
ENSG000001	YIPF3	0.00190751	0.19438665	-0.1766241
ENSG000001	GLRX5	0.19648134	0.56389367	-0.1766218
ENSG000001	SORL1	0.06217194	0.38624952	-0.1764218
ENSG000001	PCDHB5	0.58193674	0.82746456	-0.176411
ENSG000001	PLCB2	0.00408592	0.21403539	-0.1763913
ENSG000001	ZNF697	0.11786679	0.4744792	-0.1762668

ENSG000002	POC1B-GALN	0.55291716	0.81383402	-0.1760613
ENSG000001	MAP7D1	0.02388208	0.29875884	-0.1759972
ENSG000001	HCRT	0.60116514	0.83799671	-0.1759322
ENSG00000C	CST7	0.15401647	0.51533385	-0.175918
ENSG000001	JAM3	0.11173068	0.46538092	-0.1758628
ENSG000002	NPEPL1	0.02518961	0.30077106	-0.1758624
ENSG000001	ADSS1	0.19805897	0.56540555	-0.1757769
ENSG000001	TMEM163	0.40792447	0.72771726	-0.1757713
ENSG000001	HAMP	0.72890883	0.89755543	-0.175715
ENSG000001	TMPRSS9	0.1447928	0.50342628	-0.1754978
ENSG000001	MROH9	0.60898941	0.84233419	-0.1754873
ENSG000001	TDRD9	0.08793033	0.43050213	-0.1754472
ENSG000002	BGLAP	0.24320811	0.60875135	-0.1753869
ENSG000001	ARAP1	0.01221981	0.25275547	-0.1753593
ENSG000001	ISYNA1	0.10384401	0.45381195	-0.1753487
ENSG000001	KLHL21	0.00470662	0.21772133	-0.1752766
ENSG000001	H2BC12	0.05903285	0.38096018	-0.1752085
ENSG000001	ENHO	0.35169943	0.69054758	-0.1751572
ENSG000001	MAP3K3	0.00309326	0.20994948	-0.1751234
ENSG000001	ZFAND2B	0.02121518	0.28959078	-0.1751206
ENSG000001	FSIP2	0.76535762	0.91260342	-0.1747768
ENSG00000C	CRAT	0.058793	0.38067583	-0.1747432
ENSG000002	TWF2	0.01628233	0.27000801	-0.1747409
ENSG000002	ZNF90	0.44922868	0.75235957	-0.1747395
ENSG000002	FXYD7	0.27194212	0.63323084	-0.1745251
ENSG000001	GALR1	0.73485809	0.90133637	-0.1744314
ENSG000001	BEX3	0.17486731	0.53975461	-0.1743719
ENSG00000C	PIGQ	0.04401204	0.35392484	-0.1743643
ENSG000001	DNAJB2	0.04338213	0.3530621	-0.1740489
ENSG000001	DPY19L2	0.33083056	0.67424323	-0.1739742
ENSG000001	HSPA12B	0.55634918	0.81507187	-0.173753
ENSG000001	TOM1	0.00942829	0.23551879	-0.1737505
ENSG000001	PRKAR1B	0.15006099	0.50984313	-0.1737203
ENSG000001	ALPP	0.60619628	0.83983012	-0.1736568
ENSG000001	TRABD	0.03002942	0.31859418	-0.1736494
ENSG000001	PIGX	0.03665805	0.33341755	-0.1736463
ENSG00000C	STRN4	0.00399688	0.21403539	-0.1736312
ENSG000001	KDM4D	0.55729376	0.81587881	-0.1736173
ENSG000001	TPM2	0.13528364	0.49446444	-0.1736167
ENSG000001	FAM90A1	0.12037239	0.47705307	-0.1735831
ENSG000001	SLFNL1	0.38339449	0.71225009	-0.1732973
ENSG00000C	RNF10	0.12719809	0.48504355	-0.1731897
ENSG000001	NACC2	0.06887733	0.39825823	-0.1731691

ENSG000001	AQP9	0.07384039	0.40546039	-0.173152
ENSG000002	H3C14	0.61864949	0.847037	-0.173068
ENSG000001	BEGAIN	0.25620051	0.62056088	-0.1730503
ENSG00000C	FAM214B	0.00966162	0.23769052	-0.1730024
ENSG000002	TUBB8	0.30973462	0.65921628	-0.1729559
ENSG000001	GSTM5	0.59061131	0.83266712	-0.1729543
ENSG000001	ACP7	0.66090438	0.86725532	-0.1728723
ENSG000001	CRIP2	0.24716592	0.61321492	-0.172752
ENSG000001	ANKRD9	0.1613636	0.52426078	-0.1727434
ENSG000001	RNF123	0.02773408	0.3113392	-0.1726822
ENSG000001	G6PD	0.07328855	0.40494581	-0.1726087
ENSG000001	EMC3	0.03083548	0.31953017	-0.1725794
ENSG000001	MSANTD1	0.41588105	0.73235476	-0.1724853
ENSG000001	TEPSIN	0.01122055	0.2451811	-0.1724841
ENSG00000C	LZTS1	0.39913155	0.72496091	-0.1722906
ENSG000001	OSBPL1A	0.02860866	0.31345545	-0.1722793
ENSG000001	HSPBAP1	0.04595186	0.3571663	-0.1722648
ENSG00000C	NSFL1C	0.00429976	0.21614597	-0.1721907
ENSG000001	HDAC10	0.13084799	0.48934567	-0.1721751
ENSG000001	TBX6	0.12793894	0.48591919	-0.172161
ENSG000001	MXI1	0.2237331	0.59091342	-0.1720454
ENSG000002	TEX13D	0.46658979	0.76486809	-0.1719684
ENSG000002	ZNF853	0.17158056	0.53577669	-0.1719384
ENSG000002	GPSM3	0.17017399	0.53437545	-0.1718009
ENSG000001	GNB2	0.03572691	0.33014896	-0.1717303
ENSG000001	ADCY4	0.01865259	0.27889987	-0.1716471
ENSG000001	MRGPRE	0.55268789	0.81383402	-0.1715019
ENSG000001	EFCAB10	0.3262885	0.67170284	-0.1713286
ENSG000001	XPO6	0.01396555	0.25910565	-0.1712097
ENSG000001	PPFIA4	0.1988297	0.56598606	-0.1711851
ENSG000001	UBE2R2	0.0031255	0.21033652	-0.1711772
ENSG000002	UBA52	0.0645986	0.39055819	-0.1711752
ENSG00000C	BCAS1	0.21836764	0.58528507	-0.1711016
ENSG000001	PCYT1B	0.34409164	0.68472435	-0.1710953
ENSG000001	TMEM92	0.41680575	0.73289419	-0.1708926
ENSG000001	UBL7	0.02390454	0.29875884	-0.1708781
ENSG000001	F2RL3	0.2700464	0.63142427	-0.1708458
ENSG00000C	VIM	0.02313647	0.29720481	-0.1708348
ENSG000001	SLC27A1	0.07958192	0.4152849	-0.1708122
ENSG000002	HS3ST5	0.7237896	0.89594966	-0.1708004
ENSG000001	TLCD3A	0.28815347	0.64543356	-0.1704359
ENSG00000C	STXBP2	0.01886014	0.27889987	-0.1704228
ENSG000001	KIAA2013	0.00546256	0.21833269	-0.1703375

ENSG000001	LITAF	0.07322067	0.40493325	-0.1703085
ENSG000001	ANXA11	0.006752	0.22347774	-0.170284
ENSG000001	ATG16L2	0.00136178	0.18856694	-0.1698494
ENSG000001	TTC24	0.21110412	0.57528121	-0.1698218
ENSG000001	CFP	0.01118894	0.2451811	-0.1698097
ENSG000001	KMT5C	0.05042589	0.36244582	-0.1697961
ENSG000001	CCL24	0.55218461	0.81359542	-0.1697592
ENSG000001	SOX12	0.1584464	0.52129996	-0.1696687
ENSG000001	ACD	0.03408087	0.32634691	-0.169649
ENSG000001	SAMD1	0.16846026	0.53253292	-0.169639
ENSG000001	SPAG1	0.143642	0.50186843	-0.1696087
ENSG00000C	GAB2	0.02890584	0.31345545	-0.1696015
ENSG000001	MPZL1	0.03253578	0.32362351	-0.1695441
ENSG000001	JPT1	0.01296577	0.25583328	-0.1695384
ENSG000001	MOB3A	0.03357979	0.32398208	-0.1694449
ENSG000001	SPATA1	0.1198909	0.47604098	-0.169423
ENSG000001	BEST1	0.02576364	0.30449397	-0.1693945
ENSG000001	SOX4	0.13237012	0.49081838	-0.1692562
ENSG000001	HDGFL2	0.02099637	0.28769456	-0.1692495
ENSG000001	TPGS2	0.0811592	0.41848493	-0.1692191
ENSG000001	NMNAT3	0.14114423	0.50055066	-0.169211
ENSG000001	UBN1	0.0290278	0.31345545	-0.1691502
ENSG000001	FAM89B	0.08701379	0.42907415	-0.1691341
ENSG000001	SOD3	0.55146406	0.81323014	-0.1689452
ENSG000001	OAZ1	0.04655597	0.35774922	-0.1689229
ENSG000001	PRR15	0.69156576	0.88137846	-0.1688349
ENSG000002	CKLF-CMTM:	0.37881028	0.70962403	-0.1687792
ENSG000001	GLT1D1	0.01921509	0.28088168	-0.1685928
ENSG00000C	MAP4K4	0.02144146	0.2908707	-0.1685513
ENSG000001	AP2A1	0.0179208	0.27425066	-0.1685317
ENSG000001	HNF4A	0.61103023	0.84336092	-0.1685157
ENSG000001	WDR13	0.00750445	0.22933195	-0.1684615
ENSG000001	C16orf74	0.34537678	0.68522517	-0.1683402
ENSG000001	RPH3AL	0.4039496	0.72672584	-0.1680558
ENSG000002	LYPD8	0.61826063	0.8467902	-0.1680244
ENSG000001	CANT1	0.00474567	0.21772133	-0.1679531
ENSG000002	N/A	0.32954133	0.67330598	-0.16788
ENSG000001	MORN3	0.05739219	0.37700366	-0.1676803
ENSG000001	CKAP4	0.01649615	0.27000801	-0.1675616
ENSG000001	PF4	0.18709409	0.55108473	-0.1675474
ENSG000001	CA7	0.69981788	0.88452288	-0.1674641
ENSG000001	DACT1	0.27201753	0.63323084	-0.1673278
ENSG000002	N/A	0.62513347	0.85017942	-0.1672559

ENSG00000C	PILRA	0.03695056	0.33341755	-0.16725
ENSG000001	LPL	0.52201564	0.79776689	-0.1669904
ENSG000002	GOLGA8T	0.64953118	0.86257742	-0.1669864
ENSG00000C	LIMS2	0.21839715	0.58528507	-0.1669627
ENSG000001	PGD	0.03280949	0.32362351	-0.1669503
ENSG000001	GALNT13	0.58863685	0.83117632	-0.1667401
ENSG000001	SPACA3	0.71595425	0.89223808	-0.1667102
ENSG000001	ANKZF1	0.05983931	0.38277429	-0.1666974
ENSG000001	KPNA7	0.5725952	0.82203609	-0.166687
ENSG000001	RUNDC3B	0.66789045	0.87076741	-0.1665959
ENSG000001	ZNF628	0.0994856	0.44721699	-0.1665092
ENSG000001	SLC16A3	0.04847811	0.360767	-0.1660171
ENSG000001	PRR14	0.01016097	0.24174018	-0.1660163
ENSG000001	PRSS12	0.71620204	0.89233982	-0.1660142
ENSG000001	CELF6	0.66983627	0.87169443	-0.1659277
ENSG00000C	CIC	0.04864331	0.360767	-0.165901
ENSG000001	IGDCC3	0.62644349	0.85068296	-0.1658061
ENSG000001	PPP1R16A	0.12340092	0.47994951	-0.1657801
ENSG000001	NEUROD4	0.68809913	0.88051877	-0.1657652
ENSG000001	MT-ND1	0.11475855	0.47052227	-0.1656956
ENSG000001	BBOF1	0.16317881	0.52653704	-0.1656909
ENSG000001	PTGFRN	0.32544456	0.67118548	-0.1655175
ENSG00000C	TIMP2	0.00984174	0.238508	-0.1654984
ENSG000001	MMP24OS	0.01296735	0.25583328	-0.1653639
ENSG000001	PLEKHA4	0.27242563	0.63353982	-0.1653467
ENSG000001	ARSG	0.00875153	0.23436355	-0.1650902
ENSG000001	GPR146	0.21468835	0.58023513	-0.1650555
ENSG000001	PPM1M	0.02829404	0.31303636	-0.1649859
ENSG000001	GFPT2	0.63656467	0.85572098	-0.1648652
ENSG000001	CEACAM3	0.03281513	0.32362351	-0.1648228
ENSG000001	C16orf54	0.00057112	0.18856694	-0.1648164
ENSG00000C	REEP1	0.64707535	0.86129024	-0.1647407
ENSG000001	STMN1	0.31221573	0.66088329	-0.1647107
ENSG00000C	PXN	0.01641245	0.27000801	-0.1646711
ENSG000001	MSRB1	0.08964364	0.43361062	-0.1646677
ENSG000001	KIF14	0.4923794	0.7804495	-0.1645679
ENSG000001	FAM193B	0.02576872	0.30449397	-0.1644564
ENSG00000C	SCARB1	0.15741846	0.52046499	-0.1644532
ENSG000001	IRF2BPL	0.06602232	0.39343668	-0.1644428
ENSG000001	TXN	0.05358366	0.37086012	-0.164391
ENSG000001	TNRC18	0.07381021	0.40546039	-0.164356
ENSG000001	TECPR2	0.105033	0.45514301	-0.1643043
ENSG000001	IL6R	0.00770532	0.23002982	-0.1643023

ENSG000001	CD1C	0.30721048	0.65762361	-0.164277
ENSG000001	LAPTM4B	0.17730274	0.54223298	-0.1642629
ENSG000001	ANKRD6	0.11957726	0.47604098	-0.1641769
ENSG000001	PARP16	0.08521965	0.42526981	-0.1641704
ENSG000001	PI16	0.1646239	0.52786788	-0.1641334
ENSG000001	TP53TG5	0.3043717	0.65635638	-0.1641181
ENSG000001	SIGLEC7	0.01380175	0.25910565	-0.1639919
ENSG000002	C4A	0.64721888	0.86129024	-0.1639644
ENSG000001	ARSA	0.01468054	0.26167713	-0.1639154
ENSG000001	TUBB4B	0.01416305	0.25910565	-0.1637943
ENSG000001	SLC49A4	0.09367662	0.43871567	-0.1637746
ENSG000001	IP6K1	0.00682311	0.22347774	-0.1637385
ENSG000001	TRIOBP	0.0036449	0.21208655	-0.1637047
ENSG000001	MELK	0.58930883	0.83183583	-0.1636619
ENSG000001	TOP2A	0.32972346	0.67344111	-0.1636038
ENSG000001	DES	0.43607822	0.74469941	-0.1633261
ENSG000001	VASP	0.06998959	0.4002892	-0.1632978
ENSG00000(	MARCHF2	0.06829089	0.39743754	-0.1632672
ENSG000001	RHOG	0.05979501	0.38277429	-0.1629474
ENSG000001	ATG9A	0.02775258	0.3113392	-0.162925
ENSG000001	SPTA1	0.32403581	0.6699145	-0.1628991
ENSG000001	RRH	0.62761301	0.8511716	-0.1628643
ENSG000002	BLACAT1	0.72719884	0.89708846	-0.1628637
ENSG000001	TMEM121B	0.03334583	0.32398208	-0.1627953
ENSG000001	SPATA3	0.74345597	0.90602454	-0.1627886
ENSG000001	COL13A1	0.57174323	0.82168479	-0.1627265
ENSG00000(	PLXND1	0.13832979	0.49898885	-0.1626054
ENSG000001	CACNA1C	0.46582293	0.76466038	-0.1625886
ENSG000001	FRMD8	0.00981513	0.23839521	-0.1625158
ENSG000001	TRPV6	0.57900062	0.82561106	-0.1625071
ENSG000001	SEC14L2	0.30109072	0.65533646	-0.1624686
ENSG000001	PRRT3	0.10517562	0.45527501	-0.1624141
ENSG000001	ACAP3	0.07143853	0.40241675	-0.1623914
ENSG000001	FAM83A	0.6297069	0.85287112	-0.1623911
ENSG000001	NARF	0.00658954	0.22296216	-0.1623727
ENSG000001	SIRPB1	0.04085714	0.34551949	-0.1622787
ENSG000002	TMEM200B	0.72433634	0.89623486	-0.1622761
ENSG000001	BCKDK	0.0195805	0.28172722	-0.1622462
ENSG000001	ZNF777	0.07383772	0.40546039	-0.1621581
ENSG000001	TLCD3B	0.7634392	0.9121117	-0.1620858
ENSG000001	REEP6	0.27379241	0.63403633	-0.1619828
ENSG000001	WBP2	0.0379778	0.3358711	-0.1619678
ENSG000001	LRWD1	0.02638361	0.30699826	-0.1617906

ENSG00000(	ATP2A3	0.0495645	0.36137351	-0.1616403
ENSG000001	LLCFC1	0.7365456	0.90203016	-0.1615898
ENSG000001	METRNL	0.26232979	0.62579547	-0.1615763
ENSG000001	PLP2	0.0092272	0.23551879	-0.1615607
ENSG000001	STEAP4	0.22233363	0.58995895	-0.1615604
ENSG00000(	NLRP1	0.00872462	0.23436355	-0.1615058
ENSG000001	ARHGDIB	0.00787988	0.23002982	-0.1615009
ENSG000001	PSD4	0.00502148	0.21833269	-0.1613205
ENSG000001	PLCD3	0.23508108	0.60232491	-0.161212
ENSG000001	CD1E	0.50986629	0.79143652	-0.1612107
ENSG000001	SPTBN5	0.14153518	0.50055578	-0.1610921
ENSG00000(	MUSK	0.69059703	0.88091947	-0.1609201
ENSG000001	MAF1	0.04126002	0.3471186	-0.1608329
ENSG000002	ETV5	0.25252827	0.61835343	-0.1608065
ENSG000001	OR2T8	0.6637147	0.86832674	-0.1607362
ENSG000001	SLC35A2	0.00123259	0.18856694	-0.1606836
ENSG000001	ENOX1	0.71401673	0.89153258	-0.1606352
ENSG000001	CAPN5	0.19371515	0.56056904	-0.1604951
ENSG000001	TBC1D7	0.07143124	0.40241675	-0.1604718
ENSG000002	LST1	0.54482494	0.80950975	-0.1603275
ENSG000001	RAB40C	0.06571076	0.39269253	-0.1603249
ENSG000001	FAM234A	0.06204248	0.38624952	-0.1603093
ENSG000001	CLDN24	0.65137697	0.8634351	-0.1602479
ENSG000001	HMGN5	0.50202195	0.78558996	-0.1601951
ENSG000001	MAZ	0.07315287	0.40493325	-0.1601756
ENSG000001	CDKN2B	0.32565508	0.67124189	-0.1601657
ENSG00000(	RHOBTB1	0.20624404	0.56956674	-0.1600187
ENSG000002	C16orf95	0.68801732	0.88051877	-0.1598569
ENSG000001	AEBP1	0.18434721	0.54968745	-0.1598385
ENSG000001	SLC7A5	0.07969656	0.4152849	-0.1597933
ENSG00000(	BCL3	0.20422528	0.5688169	-0.1597932
ENSG000001	PIGZ	0.23801994	0.60414721	-0.1597326
ENSG000001	MYBL2	0.44766419	0.75102106	-0.15973
ENSG000002	FCGBP	0.27660303	0.6360577	-0.159656
ENSG000001	TSPO	0.08390523	0.42309602	-0.1596339
ENSG000001	FBRS	0.02462537	0.29927225	-0.1595905
ENSG000001	ZSCAN4	0.67077767	0.87216822	-0.1595806
ENSG000001	MROH7	0.46633338	0.76486809	-0.1595338
ENSG000001	FZD7	0.45025117	0.75290713	-0.1594469
ENSG000001	FOXH1	0.65325143	0.86393759	-0.1593924
ENSG000002	N/A	0.70768981	0.88890112	-0.1593373
ENSG000001	ANKRD34B	0.29059363	0.648324	-0.159034
ENSG000001	PODNL1	0.48744805	0.77793335	-0.1589877

ENSG000001	CPNE7	0.58200388	0.82746456	-0.1589589
ENSG000001	ZNF521	0.70703634	0.8886252	-0.1588958
ENSG000001	APOBR	0.12247551	0.4790737	-0.1588841
ENSG000001	PEBP4	0.74695035	0.90685156	-0.1588481
ENSG000001	ADPRHL1	0.20313723	0.56867381	-0.1588423
ENSG000001	RFX4	0.67169288	0.87278148	-0.1587347
ENSG000001	ITLN2	0.73567901	0.90150345	-0.1587258
ENSG000001	ANKRD35	0.35548119	0.69207883	-0.1587213
ENSG00000(	NDE1	0.7824693	0.9193471	-0.1586994
ENSG000002	AATF	0.60810327	0.84146691	-0.1585658
ENSG000001	NDRG2	0.27938815	0.63830383	-0.1584993
ENSG00000(	GRAMD1A	0.0335902	0.32398208	-0.1583795
ENSG000001	FAM120B	0.70001348	0.88452288	-0.1583165
ENSG00000(	SEC61A2	0.09617769	0.44215472	-0.1582706
ENSG000002	C14orf132	0.44417657	0.7491529	-0.1582433
ENSG000001	TMC2	0.56886508	0.82037292	-0.158135
ENSG000001	TBC1D17	0.01270705	0.25521652	-0.1580606
ENSG000001	DBP	0.18471841	0.5498217	-0.1580252
ENSG000001	GPR39	0.70159412	0.88523111	-0.1579881
ENSG000001	GPA33	0.23589603	0.60336718	-0.1579479
ENSG00000(	PLEKHB1	0.19247046	0.55826279	-0.1579217
ENSG000001	CDCA3	0.37617243	0.70783541	-0.1579214
ENSG000001	PTP4A3	0.76252389	0.91167139	-0.1578476
ENSG000001	FAH	0.04304209	0.35207781	-0.1578128
ENSG000002	MT-ATP8	0.02382544	0.29875884	-0.1576239
ENSG000002	N/A	0.57526086	0.82344878	-0.1575995
ENSG000002	NFAM1	0.05492296	0.37354464	-0.1575958
ENSG00000(	RNF215	0.11994093	0.47604098	-0.1575884
ENSG000001	MESP1	0.62591617	0.85056163	-0.1575772
ENSG000001	ADM2	0.16141154	0.52426078	-0.1575416
ENSG000001	FOXO4	0.08498065	0.42526981	-0.1574798
ENSG000001	ZNF787	0.03044803	0.3191802	-0.1574256
ENSG000001	HDGF	0.0393459	0.34025028	-0.1574086
ENSG000001	INSM1	0.70174359	0.88523111	-0.1573855
ENSG000001	RUNX2	0.12629954	0.48388732	-0.1573218
ENSG000001	ZNF552	0.00369297	0.21208655	-0.1572782
ENSG000001	RARA	0.07940605	0.4152849	-0.1572725
ENSG000001	SGSH	0.15276455	0.51354823	-0.1572118
ENSG000001	ABCA3	0.17347484	0.53805368	-0.157195
ENSG000001	RXFP2	0.41092811	0.72989984	-0.1571832
ENSG000001	TBC1D10C	0.07083046	0.40156223	-0.1571
ENSG000001	PICK1	0.04650501	0.35774922	-0.1569691
ENSG000001	ACRBP	0.11527352	0.47102068	-0.1569615

ENSG000001	STAT6	0.00116035	0.18856694	-0.156948
ENSG000001	USP10	0.01400051	0.25910565	-0.1569424
ENSG000001	NUF2	0.50663975	0.78939733	-0.1567761
ENSG000001	PLA2G5	0.70294536	0.88595232	-0.1567544
ENSG000001	NRG1	0.37021485	0.70302695	-0.1567231
ENSG000001	PMM1	0.06980039	0.4002892	-0.1566966
ENSG000001	MRPL41	0.1564055	0.51911584	-0.1566815
ENSG000001	MOSPD3	0.08025435	0.41684053	-0.1565103
ENSG00000C	EBF4	0.32802916	0.67237631	-0.1562947
ENSG00000C	OPHN1	0.38210925	0.71117698	-0.156088
ENSG000001	S100A6	0.00710389	0.22670251	-0.1560508
ENSG000001	SLC2A7	0.68484352	0.87924442	-0.1560064
ENSG000001	TMCC1	0.02859978	0.31345545	-0.1559982
ENSG000001	CCL19	0.70380057	0.88657108	-0.1559715
ENSG000002	ZNF316	0.03260023	0.32362351	-0.1559177
ENSG000001	KIF1A	0.69083231	0.88107274	-0.1558955
ENSG000001	ASAP1	0.09230445	0.43743098	-0.1558651
ENSG00000C	INPP5A	0.06997505	0.4002892	-0.1557964
ENSG000001	CA6	0.29290357	0.64967561	-0.1557684
ENSG000001	C9orf78	0.16997172	0.53437545	-0.1556248
ENSG000002	TMEM231	0.27761199	0.63710347	-0.1556204
ENSG00000C	HDAC7	0.02791937	0.31135087	-0.1556174
ENSG000001	RNF130	0.00554358	0.21847364	-0.1555721
ENSG000001	KIAA0232	0.06505209	0.39148468	-0.1554938
ENSG00000C	MCOLN1	0.062787	0.38794893	-0.1554447
ENSG000001	SIGLEC10	0.17429104	0.53937579	-0.1553708
ENSG000001	SIM2	0.48197774	0.77486788	-0.1553596
ENSG000001	BCL2L12	0.07348001	0.40498137	-0.1551673
ENSG000001	FHL2	0.13388613	0.4925946	-0.155156
ENSG000001	KHDC1	0.43011328	0.74091639	-0.1549858
ENSG000001	H2AW	0.26393219	0.62728666	-0.1549647
ENSG000001	EPHB1	0.0264181	0.30699826	-0.1549304
ENSG000001	HSF4	0.18794189	0.55211256	-0.1548725
ENSG000001	RAB6B	0.21468699	0.58023513	-0.1548322
ENSG000001	DGKZ	0.05932867	0.38166388	-0.1548138
ENSG000001	LRP10	0.06166898	0.3855015	-0.1545858
ENSG000001	PLA2G6	0.04471714	0.35539507	-0.1544234
ENSG000001	STARD5	0.16839177	0.53253292	-0.1543935
ENSG000001	SCYL1	0.00833277	0.23287295	-0.1543849
ENSG000001	NOCT	0.10276719	0.45220348	-0.1543488
ENSG00000C	MAP2K7	0.01289054	0.25583328	-0.1543418
ENSG000001	DTNA	0.66674033	0.87009558	-0.1543365
ENSG000001	ZFPL1	0.04937078	0.36077112	-0.154297

ENSG000001	MAN2A2	0.02454617	0.29927225	-0.1542572
ENSG000001	B9D2	0.03700509	0.33343596	-0.1542519
ENSG000002	GDI1	0.00762258	0.22933195	-0.1542219
ENSG000001	PTPRN2	0.07166522	0.40258447	-0.1540746
ENSG000001	ABCB9	0.34016196	0.68135285	-0.153961
ENSG000000	KCNAB2	0.04434092	0.35453012	-0.1539424
ENSG000001	MACROD1	0.14996606	0.50962725	-0.1538612
ENSG000000	GDE1	0.01509519	0.26260224	-0.1538035
ENSG000001	VPS37C	0.04118362	0.34701527	-0.153744
ENSG000001	CDC25C	0.5399742	0.80698777	-0.1536207
ENSG000000	RHBDD2	0.00621068	0.22079879	-0.1535017
ENSG000000	ICA1	0.1059766	0.4561473	-0.1534878
ENSG000001	CEBPB	0.09837046	0.4448142	-0.1534605
ENSG000001	C9orf16	0.06700827	0.39524277	-0.1534141
ENSG000002	CDK3	0.36624751	0.69855054	-0.1533387
ENSG000001	SH3BGRL3	0.03088045	0.31953017	-0.1533007
ENSG000001	SSH3	0.04284717	0.35185871	-0.1532855
ENSG000001	SLC35E3	0.07134914	0.40241675	-0.1532292
ENSG000001	GIGYF1	0.01556232	0.26471158	-0.1531598
ENSG000001	ARL6IP4	0.3379308	0.67967081	-0.1530988
ENSG000002	ASCL5	0.66950699	0.87133577	-0.152856
ENSG000001	CHMP2A	0.06759446	0.39679366	-0.1528327
ENSG000001	NOXRED1	0.14240941	0.50068215	-0.1527345
ENSG000001	TTLL12	0.04234361	0.35024612	-0.1525789
ENSG000001	NXNL2	0.5946066	0.83499718	-0.1524508
ENSG000001	LRRC25	0.01836414	0.27758038	-0.1523226
ENSG000001	LMTK3	0.24981875	0.61582699	-0.1522849
ENSG000002	C19orf73	0.6219626	0.84899595	-0.1522299
ENSG000001	NCLN	0.04507784	0.35545023	-0.1522078
ENSG000001	TPPP	0.29167196	0.64928401	-0.1521825
ENSG000001	CYHR1	0.00543136	0.21833269	-0.1521595
ENSG000001	KNDC1	0.34563009	0.68530904	-0.1521474
ENSG000000	SDCCAG8	0.67533421	0.87451032	-0.1521164
ENSG000000	SPPL2B	0.12632865	0.48388732	-0.1521152
ENSG000000	SPAG6	0.76733527	0.91352929	-0.1520479
ENSG000000	GNAS	0.02924548	0.31510211	-0.1519727
ENSG000001	TNFRSF8	0.07635123	0.41079896	-0.1518803
ENSG000001	PTCRA	0.19756201	0.56535325	-0.1518278
ENSG000001	ZNRF1	0.10006865	0.44762396	-0.1518205
ENSG000000	WAS	0.10473423	0.45514301	-0.1515678
ENSG000000	MYLK	0.17105695	0.53505139	-0.1512024
ENSG000001	ACOT12	0.75787876	0.91091885	-0.1511879
ENSG000002	MTFP1	0.54504755	0.80952117	-0.1511252

ENSG000001	BUB1	0.42057062	0.7349809	-0.1510999
ENSG000001	ATP13A4	0.51438097	0.79409791	-0.1509484
ENSG000002	LYPD4	0.48711583	0.77770893	-0.1509383
ENSG000001	ARRB2	0.01601066	0.2682819	-0.1508617
ENSG000001	VPS28	0.03119007	0.32005877	-0.1508508
ENSG000001	FMNL1	0.07490356	0.40812385	-0.1508487
ENSG000001	ZNF837	0.29425836	0.65044158	-0.1508392
ENSG000001	ZBTB47	0.03758399	0.33471496	-0.1506047
ENSG000001	RNF141	0.16792109	0.53240547	-0.1505845
ENSG000001	TDRP	0.4309483	0.74126857	-0.1505776
ENSG000001	TLN1	0.16856818	0.53253292	-0.1505039
ENSG000001	SLC25A44	0.00775065	0.23002982	-0.1504763
ENSG000001	CRABP2	0.20704521	0.57066596	-0.150351
ENSG000001	ALDOC	0.112333	0.46648363	-0.1503257
ENSG000001	PTTG1	0.31474416	0.66270362	-0.1503099
ENSG000002	FMN1	0.58077975	0.82662376	-0.1502988
ENSG000001	PRDX5	0.07454364	0.40721124	-0.1501321
ENSG000001	PRSS53	0.20593905	0.56956674	-0.150126
ENSG000001	CCT6B	0.44469678	0.74940749	-0.1500462
ENSG000000	AKAP8L	0.01097513	0.24311475	-0.1500051
ENSG000001	UNC119	0.01383285	0.25910565	-0.1499441
ENSG000001	EN2	0.71099119	0.89054277	-0.149788
ENSG000001	GPS2	0.16775401	0.53220434	-0.1497855
ENSG000001	ARHGAP45	0.00738006	0.22796473	-0.1496906
ENSG000001	RETREG2	0.00599214	0.22079879	-0.1496757
ENSG000001	DDX4	0.65224801	0.86379349	-0.1496357
ENSG000001	SYNE4	0.25220858	0.61794336	-0.1495382
ENSG000001	NPHP1	0.64483167	0.85991395	-0.1495167
ENSG000001	ITGB1BP2	0.56403284	0.81864675	-0.1495051
ENSG000001	CCDC17	0.05266692	0.36895324	-0.14941
ENSG000001	SLC22A1	0.11977284	0.47604098	-0.149166
ENSG000001	MFSD6L	0.25397412	0.61927883	-0.1489851
ENSG000001	USP43	0.60555399	0.83945694	-0.148632
ENSG000000	ATP6AP1	0.00937061	0.23551879	-0.1486266
ENSG000002	OR52B4	0.78066092	0.9189743	-0.1485436
ENSG000002	PSENEN	0.10489306	0.45514301	-0.1485375
ENSG000001	SLC39A4	0.19840124	0.56598606	-0.1484786
ENSG000001	SLC66A2	0.02046982	0.28641584	-0.1484646
ENSG000001	RANBP10	0.08514745	0.42526981	-0.1484266
ENSG000001	CPPED1	0.04995272	0.36170272	-0.1483684
ENSG000001	ALKBH7	0.14294042	0.5010464	-0.1482282
ENSG000001	OR10H5	0.5816357	0.82722887	-0.1480899
ENSG000001	SLC6A6	0.03101259	0.31953017	-0.1480457

ENSG000001	STX10	0.00253633	0.2093801	-0.1480201
ENSG000001	CLN6	0.11882197	0.47555373	-0.1479905
ENSG000001	TUBB1	0.21947269	0.58679039	-0.1479785
ENSG000001	PGAP2	0.06228542	0.38624952	-0.1479065
ENSG000001	FOSB	0.35471475	0.6919142	-0.1478647
ENSG000001	STAT5B	0.03466693	0.32831054	-0.1478173
ENSG000001	HPS1	0.05615452	0.37502119	-0.1477429
ENSG000001	NLGN3	0.01329783	0.25828987	-0.1477229
ENSG000001	PKIB	0.6944523	0.8821445	-0.1476858
ENSG000001	TCHH	0.72736155	0.89708846	-0.1476483
ENSG000002	TPTEP2-CSNI	0.6813783	0.87687561	-0.1476303
ENSG000001	CEP19	0.0911511	0.43488031	-0.1476029
ENSG000001	KLF2	0.06709954	0.39560468	-0.1473085
ENSG000001	MUC13	0.6546071	0.86439091	-0.147238
ENSG000001	CTHRC1	0.7262622	0.89706038	-0.147203
ENSG000001	NSG1	0.40725986	0.72768322	-0.1471758
ENSG000001	RBFOX2	0.73100861	0.89885549	-0.1471234
ENSG000001	AUNIP	0.33200176	0.67489153	-0.1471223
ENSG000001	KIF2C	0.32642172	0.67183539	-0.1471199
ENSG000001	LRP1	0.08677487	0.42885945	-0.1469871
ENSG000001	FKBP9	0.06376621	0.38991609	-0.146912
ENSG000001	DNER	0.71274115	0.89116324	-0.1469084
ENSG000002	RIMBP3B	0.3370364	0.67898382	-0.1468873
ENSG000001	ANKRD13D	0.01438789	0.2599395	-0.1468672
ENSG000001	PACSIN2	0.016648	0.27031359	-0.1468595
ENSG000002	CRPPA	0.36125043	0.69547347	-0.146808
ENSG000001	PELO	0.00865455	0.23436355	-0.1467043
ENSG000001	GADD45G	0.2605686	0.62402939	-0.1466247
ENSG000002	N/A	0.71458889	0.89175724	-0.1465798
ENSG000001	FAM53B	0.02432062	0.29927225	-0.1465574
ENSG000001	PPP1R3B	0.10523008	0.45538934	-0.146455
ENSG00000C	TBXAS1	0.01548374	0.26471158	-0.1464442
ENSG000001	CTNNBIP1	0.0514533	0.36502002	-0.1463095
ENSG000001	LRRN1	0.16094728	0.52386068	-0.1460785
ENSG00000C	LTK	0.24646424	0.61214306	-0.1460622
ENSG000002	MEF2B	0.29159162	0.64928401	-0.1459533
ENSG000002	MAP1LC3B2	0.04961734	0.36159635	-0.1457929
ENSG000001	PHF12	0.00958664	0.2365626	-0.1457412
ENSG000002	FAM229A	0.12787254	0.48588488	-0.1456955
ENSG000001	TRAPPC14	0.00292501	0.20994948	-0.1455785
ENSG000001	PGGHG	0.03994982	0.34274931	-0.1454728
ENSG000001	FLOT2	0.07427634	0.40673667	-0.1453482
ENSG000001	WFS1	0.25083631	0.61671278	-0.1452869

ENSG000001	FLII	0.02284054	0.29500624	-0.1452858
ENSG000002	N/A	0.07104967	0.40194888	-0.145264
ENSG000001	N/A	0.47888337	0.77284856	-0.1452607
ENSG000001	CORO1C	0.04003314	0.34274931	-0.1451757
ENSG000002	LAT	0.27221799	0.63335652	-0.1451425
ENSG00000C	UPF1	0.03649421	0.33341755	-0.1449005
ENSG000001	UBE2M	0.02467156	0.29927225	-0.1448679
ENSG000001	RNF157	0.15772828	0.52077869	-0.1448399
ENSG000001	AHCTF1	0.13229628	0.49081838	-0.1447065
ENSG000001	PLD2	0.03272385	0.32362351	-0.1446205
ENSG000001	CACNB4	0.4536412	0.75492182	-0.1445969
ENSG000001	RPS6KA1	0.00932771	0.23551879	-0.144593
ENSG00000C	RTEL1-TNFRS	0.19230457	0.55817827	-0.1445844
ENSG000001	ATP7B	0.18195976	0.54661139	-0.1444951
ENSG000001	PCDH18	0.72349018	0.89594062	-0.144471
ENSG000001	NOP10	0.00498869	0.21833269	-0.1444015
ENSG000001	CIB2	0.27340776	0.6339378	-0.1443806
ENSG000001	PIK3CD	0.02500297	0.30049829	-0.1443575
ENSG000002	PRAG1	0.16238305	0.52515336	-0.144293
ENSG000001	RAVER1	0.08667717	0.42881696	-0.1442606
ENSG000001	ARL17A	0.68240669	0.87750356	-0.1442349
ENSG000002	NYNRIN	0.50283739	0.78626452	-0.1441496
ENSG000001	C1R	0.18570287	0.55052683	-0.1441336
ENSG000002	C17orf113	0.34557069	0.68530904	-0.1440731
ENSG000001	ACSL1	0.26273157	0.62624377	-0.1438966
ENSG000001	UBXN10	0.44804201	0.75114004	-0.1438106
ENSG000001	ARID3B	0.0081945	0.23180164	-0.1438024
ENSG000001	MAP4K2	0.03782154	0.33525901	-0.1436502
ENSG000001	MTURN	0.07782415	0.41335649	-0.1436476
ENSG000001	EGFL7	0.26823132	0.63055915	-0.1435778
ENSG000001	LRFN3	0.16514948	0.5286578	-0.1434936
ENSG000001	WNT7A	0.45175617	0.75386982	-0.1434664
ENSG000001	TMEM81	0.100444	0.44829276	-0.1434588
ENSG000001	DNMBP	0.11754542	0.47406481	-0.1434176
ENSG000001	CPQ	0.0057845	0.21912363	-0.1432738
ENSG000001	PCNX1	0.0950717	0.44067346	-0.1431526
ENSG000002	LINC00672	0.24125923	0.60721888	-0.1430872
ENSG000001	SWSAP1	0.09182726	0.43634744	-0.1430867
ENSG000001	TMEM204	0.28303822	0.64105058	-0.1430831
ENSG000002	GLI4	0.10818742	0.45961253	-0.14308
ENSG00000C	APBB1IP	0.07659704	0.41092575	-0.1430696
ENSG000001	KCNE5	0.55350387	0.8139043	-0.143009
ENSG000001	CAMTA2	0.01308243	0.25685534	-0.1429596

ENSG00000(	DNM2	0.00937538	0.23551879	-0.1428206
ENSG000001	MYO5C	0.34921255	0.68858669	-0.142802
ENSG000001	SPTLC3	0.59394478	0.83456808	-0.1426839
ENSG000001	BCR	0.08140682	0.41884321	-0.1426553
ENSG000001	FOXO3	0.12871009	0.48692118	-0.1426086
ENSG000001	CELSR2	0.24574541	0.61158271	-0.142479
ENSG000001	MSRA	0.05080748	0.36325894	-0.1424682
ENSG000001	KIFC2	0.17880932	0.54395471	-0.1424564
ENSG000001	MFN2	0.01061528	0.24206404	-0.1424385
ENSG000002	ZBTB48	0.01500411	0.26260224	-0.1424182
ENSG000001	SIGLECL1	0.68818345	0.88051877	-0.1422653
ENSG000001	BCAR3	0.36354891	0.69694698	-0.1422644
ENSG000001	MYLPF	0.43156966	0.74126857	-0.1422515
ENSG000001	PTK7	0.13431771	0.49330843	-0.1422365
ENSG000002	SERF1B	0.70665001	0.88840748	-0.1422257
ENSG000001	PRKCSH	0.0357404	0.33014896	-0.1421961
ENSG000001	RIN3	0.04840565	0.360767	-0.1421687
ENSG000001	GRIN1	0.72674229	0.89706038	-0.1421281
ENSG000001	CXCL16	0.15790375	0.52077869	-0.1420425
ENSG00000(	DEF6	0.03970654	0.34111906	-0.1420263
ENSG000001	DNAL4	0.02591312	0.30450132	-0.1420115
ENSG000001	FES	0.05793064	0.37836675	-0.1418833
ENSG000001	MMP14	0.14407198	0.50241838	-0.1417465
ENSG000001	HHIP	0.65438291	0.86439091	-0.1417447
ENSG000001	SHOC1	0.3569088	0.69298434	-0.1417139
ENSG000002	PRR33	0.0284761	0.31345545	-0.1417118
ENSG000001	WDFY4	0.05015813	0.36227834	-0.141659
ENSG000001	GRK6	0.01145509	0.24590889	-0.1415206
ENSG000001	FCAR	0.70284506	0.88595232	-0.141328
ENSG000001	CMTM8	0.21935636	0.58667257	-0.1412927
ENSG000002	KCNE1B	0.39505126	0.72209218	-0.1412797
ENSG000001	TEF	0.04922778	0.360767	-0.1411954
ENSG000001	ELOB	0.08703502	0.42907415	-0.141173
ENSG00000(	ANOS1	0.63018413	0.85293934	-0.1411235
ENSG000001	SAXO2	0.6770588	0.87510133	-0.1411187
ENSG000001	HECW2	0.33148922	0.67448501	-0.1410905
ENSG000001	RAF1	0.00185849	0.19438665	-0.1410865
ENSG000001	RFPL2	0.49413088	0.78124152	-0.1410494
ENSG000001	METTL7B	0.69214165	0.88158966	-0.1410134
ENSG000001	LRRC8A	0.01884401	0.27889987	-0.1410031
ENSG000001	EZHIP	0.62549249	0.85038279	-0.1409496
ENSG000001	SCAF1	0.08610158	0.42701017	-0.1407384
ENSG000001	ABHD17C	0.23483495	0.60232491	-0.1407074

ENSG000001	NOL3	0.17614012	0.54121794	-0.1405526
ENSG00000C	GMIP	0.02578354	0.30449397	-0.140525
ENSG000002	SYT3	0.71030268	0.89018096	-0.1405019
ENSG000001	RASL11A	0.22742256	0.59536815	-0.1404935
ENSG000001	BANP	0.02165141	0.29174604	-0.1403755
ENSG000001	MT-ND3	0.09188642	0.43640386	-0.1402838
ENSG000001	FPR2	0.16641918	0.53014485	-0.1401529
ENSG000002	SH3D21	0.21097045	0.57523547	-0.140088
ENSG000002	PVALEF	0.68398091	0.87883184	-0.1400832
ENSG000002	RPS28	0.20430466	0.5688169	-0.1400757
ENSG000001	AGAP2	0.03317024	0.3238836	-0.1400535
ENSG000001	DNAH7	0.67348902	0.87357734	-0.1400311
ENSG000001	ARRDC1	0.03751672	0.33471496	-0.1400214
ENSG000001	SLC9A3R1	0.03463726	0.32831054	-0.1400131
ENSG000001	RBP1	0.7173715	0.89309227	-0.1400032
ENSG000002	ZSCAN31	0.55748448	0.81587881	-0.1399548
ENSG00000C	ARHGAP4	0.06371029	0.38981851	-0.139892
ENSG000001	MYT1L	0.72282029	0.89570645	-0.139878
ENSG000001	EZH1	0.02671589	0.30743159	-0.1398778
ENSG000001	SPOCK2	0.22060281	0.5880238	-0.1397957
ENSG000001	ALB	0.80455017	0.92881463	-0.1397693
ENSG00000C	LTBP1	0.41400183	0.73141372	-0.1396479
ENSG000001	ADAMTS4	0.41995138	0.73466853	-0.1396161
ENSG000001	ADORA2A	0.41266607	0.73063968	-0.1395309
ENSG000002	TMEM191B	0.55732607	0.81587881	-0.1395137
ENSG000001	MAPK8IP3	0.13359346	0.49240713	-0.1395017
ENSG000001	PPM1J	0.32316413	0.66903175	-0.1394907
ENSG000001	RABGEF1	0.09308007	0.43758758	-0.1393944
ENSG000001	NEK6	0.16607882	0.52926827	-0.1393867
ENSG000001	FN3K	0.25425862	0.619382	-0.1392905
ENSG00000C	MAEA	0.02972705	0.31817936	-0.1392739
ENSG000001	FBRSL1	0.07886677	0.41446832	-0.1392528
ENSG000002	PRSS41	0.56367062	0.81863326	-0.1392378
ENSG000001	MAMSTR	0.53964099	0.80698777	-0.1392027
ENSG000001	OAZ2	0.01459018	0.26147992	-0.1391381
ENSG000001	HSPB1	0.08665778	0.42881696	-0.1391239
ENSG000001	KIF20A	0.55982259	0.81699589	-0.1389401
ENSG000001	RAB11B	0.04372259	0.35372007	-0.1388988
ENSG000001	SH3BGRL2	0.27505707	0.63499184	-0.1388987
ENSG000001	AK5	0.37331826	0.70540773	-0.1388927
ENSG000001	KCNN4	0.15899066	0.5217239	-0.1387775
ENSG000001	ARMC3	0.44415294	0.7491529	-0.138685
ENSG000001	WNT5A	0.7271718	0.89708846	-0.1386732

ENSG000001	AP2M1	0.0404203	0.34469771	-0.1386682
ENSG000002	SPDYC	0.66285412	0.86788287	-0.1386649
ENSG00000C	LXN	0.26495571	0.6274921	-0.1386276
ENSG000002	N/A	0.4066874	0.7275473	-0.1385224
ENSG000001	FAM98C	0.11981009	0.47604098	-0.1384648
ENSG000001	APOLD1	0.34529842	0.68522517	-0.1384429
ENSG00000C	REX1BD	0.16849326	0.53253292	-0.1384163
ENSG000001	CD55	0.07667164	0.41092575	-0.1383984
ENSG00000C	IZUMO4	0.20115641	0.56773237	-0.1383972
ENSG000001	RAPGEFL1	0.06412545	0.39043354	-0.1382699
ENSG000002	PCDHGB5	0.76846933	0.91358163	-0.1382389
ENSG000001	SIGLEC9	0.02264188	0.29448836	-0.1381294
ENSG000001	SPACA9	0.37189791	0.70403479	-0.1381064
ENSG000001	TSC2	0.04883992	0.360767	-0.1380561
ENSG00000C	POLR2J	0.0244385	0.29927225	-0.1380366
ENSG000001	ITGB2	0.03318451	0.3238836	-0.1379754
ENSG000001	WIPF3	0.75755435	0.91082077	-0.1379026
ENSG000001	INPP5K	0.01494276	0.26260224	-0.1378834
ENSG000001	SLC15A3	0.07645349	0.41092575	-0.1377738
ENSG000001	TAMALIN	0.25033316	0.61627732	-0.1377573
ENSG00000C	EPN1	0.0628205	0.3879865	-0.1377551
ENSG000001	DEDD2	0.03692329	0.33341755	-0.1376669
ENSG000001	WIPI2	0.00614173	0.22079879	-0.1376194
ENSG00000C	PHGDH	0.22450035	0.59186495	-0.1375915
ENSG000002	RAD21L1	0.67328158	0.87354948	-0.1375552
ENSG000002	ARRDC5	0.28394323	0.64229398	-0.1375322
ENSG00000C	APLP2	0.00684045	0.22347774	-0.1374673
ENSG000001	TAC4	0.23282789	0.60019671	-0.1373685
ENSG000001	PITPNA	0.0065756	0.22296216	-0.137244
ENSG000001	LRRN3	0.41931929	0.73438543	-0.1372102
ENSG000001	CYB5R1	0.02999069	0.31859418	-0.137202
ENSG000001	ALPK3	0.20916375	0.57310021	-0.1371934
ENSG000002	CEBPA	0.11629953	0.47160917	-0.1371749
ENSG000001	SLC45A1	0.42327226	0.73709477	-0.1370499
ENSG000001	OR2T33	0.76351197	0.9121117	-0.1369214
ENSG000001	ZBTB16	0.42558368	0.73817463	-0.1369063
ENSG000001	HCLS1	0.00550499	0.21833269	-0.1367702
ENSG00000C	LMO3	0.77464913	0.91628579	-0.1367649
ENSG000001	APBB1	0.27358152	0.63403495	-0.1367322
ENSG000002	GGNBP2	0.736915	0.90222356	-0.1367056
ENSG000001	TSPAN2	0.12941319	0.4871968	-0.1366202
ENSG000001	MIDN	0.08567495	0.42602033	-0.1366167
ENSG000001	TBC1D10B	0.02941015	0.31615886	-0.1365543

ENSG000001	MAP2K2	0.02239821	0.29351345	-0.136402
ENSG00000(	FXYD5	0.09557569	0.4413663	-0.1363674
ENSG000001	FAM131A	0.04722456	0.35864605	-0.1363338
ENSG000001	CRTC2	0.00192934	0.19438665	-0.1362838
ENSG000001	RGCC	0.38026433	0.70987972	-0.1362503
ENSG000001	VEGFB	0.18567791	0.55052683	-0.1362386
ENSG000001	KLHL2	0.30958632	0.65916845	-0.1361552
ENSG000001	SYTL1	0.13485599	0.49418805	-0.1361011
ENSG000001	OSCAR	0.13045191	0.48854664	-0.1360705
ENSG000001	LIMD2	0.03293261	0.32362351	-0.13606
ENSG000001	SLC36A1	0.08141092	0.41884321	-0.1359651
ENSG000001	HGFAC	0.65163115	0.86352007	-0.1359356
ENSG000001	DGCR6	0.74839591	0.90731063	-0.1358622
ENSG000001	CARD19	0.00829105	0.23282737	-0.1357393
ENSG00000(	WDR62	0.25695362	0.62086214	-0.1357361
ENSG000001	LENEP	0.74563553	0.9064753	-0.1356846
ENSG00000(	ATP1A2	0.74077564	0.90510754	-0.1356805
ENSG00000(	STMN4	0.78714951	0.92141494	-0.1356733
ENSG00000(	TSPAN6	0.62349895	0.84952196	-0.1356086
ENSG00000(	FOXJ2	0.01094534	0.24311475	-0.1355168
ENSG000002	F8A3	0.27317034	0.6338147	-0.1354889
ENSG000001	F2RL1	0.2256383	0.59319591	-0.1354794
ENSG000001	SMPD2	0.10736894	0.45831177	-0.135474
ENSG000001	SLC2A5	0.28388	0.64229398	-0.1354173
ENSG000001	FDXR	0.19583955	0.5634849	-0.135318
ENSG000001	XKR8	0.04725778	0.35865951	-0.1352988
ENSG000001	SLCO4C1	0.29165807	0.64928401	-0.1352954
ENSG000001	KIAA0513	0.01087129	0.24206404	-0.135281
ENSG000001	MARVELD1	0.18704083	0.55108473	-0.1352717
ENSG00000(	FAM160A2	0.03500885	0.32931219	-0.1351897
ENSG000001	PPP4C	0.02820143	0.31256428	-0.1351832
ENSG000001	GSK3A	0.01282486	0.25583328	-0.1351733
ENSG000001	OR2B6	0.43134871	0.74126857	-0.1351689
ENSG000001	PDGFB	0.21083276	0.57523547	-0.1351257
ENSG000002	CRIP1	0.37967249	0.70965485	-0.1350822
ENSG000001	DENND3	0.01961573	0.28185889	-0.1349529
ENSG000001	DCHS1	0.35903371	0.69428738	-0.1348294
ENSG000001	ECSIT	0.07298029	0.40435477	-0.1346841
ENSG000001	PYCARD	0.08061798	0.41748748	-0.1346038
ENSG000001	PARD6A	0.32393794	0.6699145	-0.1344999
ENSG000001	BEND2	0.3350212	0.67700551	-0.1344038
ENSG000001	ADARB2	0.72335053	0.89589131	-0.134394
ENSG000001	SEMG1	0.70223564	0.88553899	-0.134319

ENSG000001	BRAT1	0.0549977	0.37354464	-0.1342883
ENSG000001	C1orf56	0.1313605	0.4894044	-0.1342616
ENSG000001	NDUFA11	0.13321075	0.49202523	-0.1342244
ENSG000002	SIPA1	0.06231869	0.38624952	-0.134219
ENSG000002	POU5F2	0.52632726	0.800185	-0.1341132
ENSG000001	HS1BP3	0.00880705	0.23442641	-0.1341074
ENSG000001	CCM2	0.00620914	0.22079879	-0.1339926
ENSG000001	ABHD2	0.13344721	0.49240713	-0.1339165
ENSG00000C	YBX1	0.06230422	0.38624952	-0.1339154
ENSG000001	RPGR	0.17271954	0.53714513	-0.1339108
ENSG00000C	CALCOCO1	0.00401222	0.21403539	-0.1339032
ENSG000001	ZNF396	0.25319853	0.61912837	-0.1338901
ENSG000001	ABHD8	0.09764966	0.44443627	-0.1338459
ENSG000002	PPT2-EGFL8	0.60361338	0.83883394	-0.1337491
ENSG000002	C6orf163	0.33730285	0.67926926	-0.1337014
ENSG000001	RAMP1	0.65412732	0.86439091	-0.1335118
ENSG000001	PC	0.28930069	0.64721	-0.1335018
ENSG000001	NFIA	0.22040594	0.58793023	-0.1333896
ENSG000001	MIB2	0.20894037	0.57297071	-0.1332728
ENSG000001	RPP40	0.39558347	0.72212079	-0.133202
ENSG00000C	TACR2	0.65509177	0.86442301	-0.1331424
ENSG000001	ORAI2	0.04485497	0.35545023	-0.1330164
ENSG000001	BST1	0.03110494	0.31965243	-0.1330087
ENSG000001	GNG4	0.73564127	0.90150345	-0.1329892
ENSG000002	N/A	0.48954501	0.77987726	-0.1329619
ENSG000002	N/A	0.26934229	0.63100512	-0.1328389
ENSG000001	PKP4	0.08836956	0.43153579	-0.1328384
ENSG000001	GLUL	0.14079085	0.50051652	-0.1328353
ENSG000001	GMFG	0.01767531	0.27418797	-0.1326697
ENSG000001	TPGS1	0.2132912	0.57903515	-0.1325864
ENSG000001	TANGO2	0.01653739	0.27013841	-0.1325602
ENSG000001	GP1BA	0.09422499	0.43939485	-0.132429
ENSG000001	ARHGEF12	0.24201189	0.60760172	-0.1324281
ENSG000001	TMEM71	0.14412844	0.50241838	-0.1324172
ENSG00000C	EIF2AK1	0.08603596	0.42681511	-0.1323436
ENSG000001	IFNL1	0.73199046	0.89917898	-0.1322826
ENSG000001	ZDHHC19	0.61424517	0.8451435	-0.1322165
ENSG000001	CYB5R2	0.69249803	0.88158966	-0.132208
ENSG000001	CDIPT	0.03516435	0.32931219	-0.1321851
ENSG000001	PROKR2	0.77239494	0.91518678	-0.1321265
ENSG000001	BRD4	0.03806389	0.33602196	-0.1320424
ENSG000001	ACTN4	0.11987813	0.47604098	-0.1320298
ENSG000001	CHP1	0.00617743	0.22079879	-0.1319756

ENSG000001	SHB	0.55340011	0.81385767	-0.1319444
ENSG000002	NHLRC4	0.29975817	0.6542516	-0.1319028
ENSG000001	GGH	0.43221392	0.74168858	-0.1318266
ENSG000001	TMEM127	0.00377689	0.21208655	-0.1317609
ENSG000001	AMPD3	0.17493508	0.53975461	-0.1317501
ENSG000001	SNAPC4	0.04711198	0.35851546	-0.1314986
ENSG000001	ST6GALNAC6	0.39097584	0.71964297	-0.1314642
ENSG000001	MLXIP	0.05053095	0.36271932	-0.1313874
ENSG000001	GCSAML	0.63503626	0.85504577	-0.1313752
ENSG000001	JUND	0.04078819	0.34551949	-0.1312673
ENSG000001	MEP1A	0.7877378	0.92183774	-0.1312213
ENSG000001	GLRA3	0.79902055	0.92603117	-0.1311176
ENSG000001	DKK2	0.74250655	0.90558693	-0.131115
ENSG000001	PLIN3	0.13798521	0.49898885	-0.1310495
ENSG000001	PRG2	0.75225351	0.908573	-0.1309823
ENSG000001	SLCO3A1	0.0052623	0.21833269	-0.130963
ENSG000001	ITIH3	0.51903213	0.79608361	-0.1309448
ENSG000001	ZNF524	0.09711188	0.44317188	-0.1308353
ENSG000001	FHOD1	0.04927756	0.360767	-0.1307985
ENSG000001	CRYBG2	0.23166209	0.599371	-0.1307701
ENSG000001	PMEL	0.53425846	0.80388793	-0.1307413
ENSG000001	RABEPK	0.15249555	0.5133091	-0.1306614
ENSG000002	CPTP	0.11291942	0.46784194	-0.1306395
ENSG000002	KMT2B	0.07955567	0.4152849	-0.1306105
ENSG000001	OSBPL2	0.0090615	0.23513527	-0.1305304
ENSG000001	SDCBP	0.0710967	0.40194888	-0.1305242
ENSG000001	FAAP24	0.08757156	0.4299506	-0.1304998
ENSG000001	C9orf153	0.79770285	0.9258334	-0.130425
ENSG000001	MYO7A	0.21841156	0.58528507	-0.1304101
ENSG000001	UBALD2	0.05085033	0.36340528	-0.1303123
ENSG000001	HGD	0.35466706	0.69190544	-0.1302732
ENSG000001	RPS6KL1	0.58322603	0.82845829	-0.1302069
ENSG000001	CNPPD1	0.08149196	0.41887936	-0.1301826
ENSG000001	MIEF2	0.17969668	0.54477842	-0.1301514
ENSG000001	WDTC1	0.04258046	0.35166779	-0.1301467
ENSG000001	ZNF653	0.15814589	0.52089921	-0.1301359
ENSG000000	ACADVL	0.0331816	0.3238836	-0.1300603
ENSG000001	JSRP1	0.41408186	0.73141372	-0.1299348
ENSG000001	FAM170B	0.79055135	0.92280247	-0.1299144
ENSG000001	PLXDC1	0.43988604	0.74656941	-0.1298778
ENSG000000	SIRT6	0.02445691	0.29927225	-0.1298681
ENSG000001	DCST1	0.58151145	0.82722887	-0.1298139
ENSG000001	XYLT1	0.23496185	0.60232491	-0.1298124

ENSG000001	ART4	0.75851812	0.91108587	-0.129676
ENSG000001	DNAJC4	0.08811833	0.43082726	-0.1295942
ENSG000001	ERCC2	0.16967924	0.53434481	-0.1295029
ENSG00000C	TMEM132A	0.45417176	0.7555818	-0.1294577
ENSG000001	TSC22D4	0.06555506	0.39248431	-0.1293925
ENSG00000C	SSH1	0.17980118	0.54485895	-0.1293393
ENSG000001	COL4A5	0.66295198	0.86788287	-0.1293015
ENSG000001	CCDC89	0.7797157	0.91827559	-0.12926
ENSG000001	FOXD4L4	0.78573287	0.92088527	-0.1292538
ENSG000001	VPS18	0.05228659	0.36780562	-0.1292019
ENSG000001	CD14	0.12962309	0.4871968	-0.1290961
ENSG000001	TUBA1A	0.09237857	0.43743098	-0.1290476
ENSG000001	PHKG2	0.05889503	0.38096018	-0.1287894
ENSG000002	GMCL2	0.69514637	0.88224216	-0.1287679
ENSG000001	SLC38A4	0.79722052	0.92579076	-0.1287553
ENSG000002	CYS1	0.76645331	0.91318627	-0.1287205
ENSG000001	RAB11FIP1	0.19097734	0.55661443	-0.1285701
ENSG000001	ABHD14B	0.1576757	0.52077869	-0.1284853
ENSG000001	OPRL1	0.20482893	0.56891466	-0.1284565
ENSG000001	CAMK1D	0.09399836	0.43929464	-0.1284219
ENSG000001	HEMGN	0.3839015	0.7123445	-0.1284118
ENSG000001	CBX8	0.18306892	0.5476216	-0.1283723
ENSG000001	PRSS36	0.393358	0.72063539	-0.1283236
ENSG000001	SYNPO2L	0.76167873	0.91151487	-0.1281404
ENSG000001	BICD2	0.08493972	0.42526981	-0.1281271
ENSG000001	KIF5C	0.32316003	0.66903175	-0.128014
ENSG000001	IFT122	0.1363165	0.49627151	-0.1279971
ENSG000001	RNF44	0.09806981	0.44466896	-0.1279416
ENSG000001	SLC44A2	0.01204004	0.25192551	-0.1278928
ENSG000001	GSPT1	0.23689298	0.60364584	-0.127783
ENSG000001	CARD14	0.35813574	0.69382497	-0.127717
ENSG000001	RHPN1	0.26443193	0.6274921	-0.1276668
ENSG000001	FERMT3	0.07390949	0.40570265	-0.1276231
ENSG000001	PLEKHA6	0.44268926	0.74861323	-0.127556
ENSG000001	PLCG2	0.01417036	0.25910565	-0.1275422
ENSG00000C	P2RX6	0.65763095	0.86540022	-0.1275273
ENSG000001	NAA10	0.08554811	0.42602033	-0.1274911
ENSG000001	JAML	0.0289638	0.31345545	-0.1274068
ENSG00000C	MID2	0.39523084	0.72209218	-0.1273671
ENSG000001	MXRA7	0.3805435	0.70987972	-0.1273669
ENSG000001	TAZ	0.10432676	0.45468989	-0.1273374
ENSG000001	TTLL9	0.73420645	0.90074122	-0.1273074
ENSG000001	RNF122	0.01980879	0.28312963	-0.1273046

ENSG000001	KATNB1	0.04148386	0.34755298	-0.127262
ENSG000001	CYSTM1	0.21448161	0.58023513	-0.1271939
ENSG00000C	TP63	0.49731948	0.78261062	-0.1271603
ENSG00000C	ARHGAP6	0.28947753	0.64727721	-0.127124
ENSG000001	TMIGD1	0.75382643	0.90944172	-0.1271068
ENSG000001	LAMP1	0.02900295	0.31345545	-0.1270676
ENSG000001	ENO3	0.3183121	0.66581089	-0.1270024
ENSG000001	DNAH14	0.37882744	0.70962403	-0.1269263
ENSG00000C	AHRR	0.48578202	0.7774422	-0.1269218
ENSG000001	SELP	0.22026733	0.58775361	-0.1268442
ENSG000001	C9orf24	0.73333709	0.89994666	-0.1267158
ENSG000001	RAB38	0.427323	0.73948387	-0.1266183
ENSG000001	SYN2	0.62993015	0.85293934	-0.1264884
ENSG000001	CNIH2	0.29611628	0.65199276	-0.1264246
ENSG000001	PPP1R37	0.17643251	0.54126676	-0.1263724
ENSG000001	GALNS	0.01036812	0.24174018	-0.1263326
ENSG000001	FBXL20	0.20757143	0.57126603	-0.1263027
ENSG000001	CCDC57	0.23261844	0.59995954	-0.1262574
ENSG000001	PAPSS2	0.28593932	0.6436707	-0.1261939
ENSG000001	NTSR1	0.3160838	0.66372668	-0.1259848
ENSG00000C	XRCC1	0.04434629	0.35453012	-0.1259627
ENSG000002	SMKR1	0.54521143	0.80952117	-0.1259227
ENSG000001	ATP6V1A	0.13014206	0.48813042	-0.1259093
ENSG000002	ZNF425	0.24300348	0.60856573	-0.1258733
ENSG000001	AKR1C4	0.75445111	0.90956509	-0.1257578
ENSG000001	MT-ND2	0.18014726	0.54501079	-0.1257526
ENSG000001	KNSTRN	0.15492327	0.51620955	-0.125706
ENSG000001	RNF187	0.10241545	0.45177448	-0.1256979
ENSG00000C	FKBP6	0.72710206	0.89708846	-0.1256785
ENSG000001	DALRD3	0.1378073	0.49881219	-0.1256736
ENSG000001	IL22RA1	0.6688859	0.87125589	-0.1256207
ENSG000001	IL17RA	0.08113085	0.41848493	-0.1255843
ENSG000001	DDIT3	0.09233051	0.43743098	-0.1255486
ENSG000001	DUSP2	0.40603618	0.72744229	-0.1254841
ENSG000001	AGPAT2	0.07864519	0.41446832	-0.1254319
ENSG00000C	CP	0.40721271	0.72768322	-0.1253367
ENSG000001	KAT8	0.03749827	0.33471496	-0.1252811
ENSG000001	OBSL1	0.47219233	0.76754298	-0.1252045
ENSG000001	GATA6	0.7627834	0.91167139	-0.1251845
ENSG000001	KDM5B	0.02351293	0.29875884	-0.1249866
ENSG000001	NMNAT1	0.08010996	0.41650511	-0.1249203
ENSG000001	RAI2	0.62211199	0.8490571	-0.124906
ENSG000001	CLPTM1	0.03690943	0.33341755	-0.1249057

ENSG00000C	MYH7B	0.34039918	0.68141884	-0.124777
ENSG000001	SSH2	0.08983915	0.43377006	-0.1247338
ENSG000001	ZNF414	0.18659168	0.55095275	-0.1246746
ENSG000001	ZNF821	0.22418116	0.59160178	-0.124672
ENSG000001	CIB3	0.33063017	0.67417332	-0.1246598
ENSG000001	PAK6	0.52013615	0.79709497	-0.1246503
ENSG000001	TTC9	0.24087013	0.60721888	-0.1246083
ENSG000002	TTC4	0.48047816	0.77383118	-0.1245277
ENSG000001	SPATA2L	0.15440038	0.51582229	-0.1245195
ENSG000001	ALKBH4	0.05216204	0.36728317	-0.1243697
ENSG000001	FAM81B	0.76001416	0.91136366	-0.1243478
ENSG00000C	STAG3	0.18654592	0.55095275	-0.124311
ENSG000001	PIK3R2	0.81273938	0.93170624	-0.1242719
ENSG000001	SHROOM3	0.76228364	0.91156278	-0.1242579
ENSG000001	MS4A2	0.68869765	0.88057199	-0.1242557
ENSG000001	PHLDB3	0.26192691	0.62561343	-0.1242457
ENSG000001	TPCN1	0.10780794	0.45898034	-0.1242278
ENSG000002	LINC02218	0.36103002	0.69535997	-0.1241718
ENSG000001	KLC4	0.05598879	0.37502119	-0.1241375
ENSG000001	CCDC24	0.28513487	0.64333814	-0.1241062
ENSG000001	NDEL1	0.04093394	0.34598876	-0.1240604
ENSG000001	WDR83	0.07859124	0.41446832	-0.1239498
ENSG000001	DHRS7B	0.02642186	0.30699826	-0.123912
ENSG000001	NEGR1	0.31802651	0.66560924	-0.1238872
ENSG000001	LGALS3	0.21036444	0.57453195	-0.123883
ENSG000001	ZNF835	0.34905825	0.6884911	-0.1238527
ENSG000001	ENO4	0.78234798	0.9193471	-0.1238421
ENSG000001	PLEKHH2	0.71905833	0.89386235	-0.1237726
ENSG000001	SLCO2A1	0.75317405	0.90916139	-0.1236849
ENSG000001	MCEMP1	0.23305667	0.60037143	-0.1236594
ENSG000001	NECAB3	0.20171714	0.56773237	-0.1236295
ENSG000002	MXD3	0.07330817	0.40494581	-0.1235502
ENSG000001	CHRM3	0.77881559	0.91810931	-0.1235467
ENSG000001	XPC	0.03418834	0.32634691	-0.1235344
ENSG000002	N/A	0.40308024	0.72672584	-0.1233403
ENSG000001	FPR3	0.48223215	0.77494096	-0.1232371
ENSG000001	CCDC157	0.22837862	0.59653313	-0.1232184
ENSG000002	N/A	0.78359343	0.91983554	-0.1231387
ENSG000002	LIPN	0.28690752	0.64432975	-0.1230881
ENSG000001	GBGT1	0.09073338	0.43458344	-0.1230819
ENSG000001	ATP6V0D1	0.06212789	0.38624952	-0.12305
ENSG000002	CCDC183	0.42658141	0.73902934	-0.1230361
ENSG000001	MT-CYB	0.19001377	0.55480195	-0.1230316

ENSG00000C	TNFRSF1A	0.08486797	0.42526981	-0.1230281
ENSG000001	CTDSP1	0.01878214	0.27889987	-0.1230145
ENSG000001	OR5AN1	0.80105351	0.92726213	-0.123007
ENSG000001	LSMEM1	0.26830123	0.63063217	-0.1230009
ENSG00000C	NTN4	0.69672695	0.88285035	-0.1229364
ENSG000001	LRP11	0.79536669	0.92462665	-0.1229285
ENSG000001	TCP11L2	0.17350988	0.53805957	-0.1228883
ENSG000001	GATAD2A	0.0395831	0.34093258	-0.1226838
ENSG000001	MTMR3	0.04597534	0.35717781	-0.1226636
ENSG000002	VAMP2	0.05431234	0.37256839	-0.1225859
ENSG000001	MRGPRD	0.74575965	0.90651247	-0.1225856
ENSG000001	IFNGR2	0.19656449	0.56398967	-0.1225486
ENSG00000C	CECR2	0.55945913	0.81696069	-0.1225148
ENSG00000C	GCKR	0.64620941	0.86053491	-0.1223871
ENSG000001	EEF2	0.1306057	0.48874043	-0.1223376
ENSG00000C	DGCR2	0.02767212	0.3113392	-0.1223358
ENSG000001	NUAK2	0.04404403	0.35392484	-0.1223298
ENSG000001	C1orf198	0.22918848	0.59761256	-0.1222911
ENSG00000C	MAP3K20	0.12313587	0.4799225	-0.12227
ENSG000001	MSL1	0.07380933	0.40546039	-0.1222498
ENSG000001	GAPDH	0.06737648	0.39585774	-0.1222126
ENSG000001	SYP	0.23136702	0.5991557	-0.1217499
ENSG000001	NUTM1	0.66197261	0.86736018	-0.1217462
ENSG00000C	TOLLIP	0.01698943	0.27204869	-0.121714
ENSG000001	CTNND2	0.76844026	0.91358163	-0.1216659
ENSG000001	CYP1B1	0.48432601	0.77665533	-0.1215316
ENSG000001	STMN3	0.28390624	0.64229398	-0.1215232
ENSG000001	SLC10A3	0.11086037	0.46380827	-0.1215189
ENSG000002	N/A	0.21851316	0.58528507	-0.121397
ENSG000001	SH3BP1	0.12959567	0.4871968	-0.1213542
ENSG000001	YIF1B	0.12681953	0.48465716	-0.1212708
ENSG000001	PLPP2	0.625186	0.85017966	-0.1212488
ENSG00000C	MPO	0.26600385	0.62878215	-0.1212398
ENSG000001	CADM1	0.60412732	0.83883394	-0.121202
ENSG000001	DNAJB12	0.00341136	0.2114129	-0.1211569
ENSG000002	ZNF487	0.163481	0.52709285	-0.1210834
ENSG000001	PGLS	0.04689981	0.35811287	-0.1210783
ENSG000001	SHLD1	0.19777526	0.56540555	-0.121073
ENSG000001	TCF20	0.65789725	0.86540022	-0.1210572
ENSG000001	TOR2A	0.08934854	0.43342334	-0.1210383
ENSG000001	SLCO4A1	0.54467182	0.8093563	-0.1209533
ENSG000001	EMD	0.07653243	0.41092575	-0.1209276
ENSG00000C	DPF1	0.63226267	0.85418109	-0.1209232

ENSG000001	CORO1A	0.06278283	0.38794893	-0.1209147
ENSG000001	CBFA2T3	0.06353725	0.38981851	-0.1209127
ENSG000002	C16orf96	0.54324825	0.8084284	-0.120738
ENSG000001	RAB42	0.44352395	0.7490637	-0.1205384
ENSG000001	PMF1	0.03942396	0.34049297	-0.1205343
ENSG000001	TMEM164	0.05034986	0.36227834	-0.1205315
ENSG000001	GRB14	0.75213954	0.908573	-0.1205206
ENSG000001	VEPH1	0.4531053	0.75457136	-0.1205087
ENSG000001	LDLRAP1	0.30758086	0.65762361	-0.1203989
ENSG000001	ADAMTS13	0.41043196	0.72968179	-0.1203745
ENSG000002	MBLAC1	0.46976693	0.7662622	-0.1203396
ENSG000001	RENBP	0.03318029	0.3238836	-0.1203189
ENSG000001	VENTX	0.23792154	0.60402516	-0.1203113
ENSG000001	C1QTNF7	0.80585094	0.92949365	-0.1202761
ENSG00000C	DSP	0.71461993	0.89175724	-0.1201087
ENSG00000C	FHL1	0.24005437	0.60689277	-0.1200694
ENSG00000C	KLHL22	0.13747463	0.49827463	-0.1199677
ENSG00000C	PITPNM2	0.20654728	0.57016461	-0.1199626
ENSG000002	N/A	0.81517993	0.93228896	-0.1198954
ENSG000001	ELMO3	0.16060105	0.52363038	-0.1198812
ENSG00000C	PGM1	0.05747998	0.3770373	-0.1198561
ENSG000001	ARHGEF11	0.02140617	0.2908707	-0.1198085
ENSG000001	SLC5A5	0.46701216	0.76514065	-0.1198065
ENSG000001	LHB	0.81535404	0.93228896	-0.119795
ENSG000001	APBB3	0.07941629	0.4152849	-0.1197908
ENSG000001	GFI1B	0.35043233	0.69002787	-0.1197688
ENSG000001	CAPS	0.19404971	0.5613371	-0.1196795
ENSG000002	TSPAN4	0.30717852	0.65762361	-0.1195982
ENSG000001	FBXO9	0.14845334	0.50794171	-0.1194968
ENSG000001	GCM1	0.39210574	0.71994497	-0.1194877
ENSG000002	ERVH48-1	0.54751021	0.81118038	-0.1194521
ENSG000001	MEST	0.41569903	0.73223098	-0.1193537
ENSG000002	ATP5MGL	0.82325495	0.93539963	-0.119287
ENSG000001	SF3A2	0.1304508	0.48854664	-0.1191641
ENSG000001	RPL36	0.30659704	0.65735608	-0.1190827
ENSG000001	CLPP	0.1431625	0.50140845	-0.1190249
ENSG000001	ADORA2B	0.38052538	0.70987972	-0.1190215
ENSG000001	TEX14	0.55993421	0.81699589	-0.1188799
ENSG000001	COL26A1	0.51982264	0.7967342	-0.1187652
ENSG000001	MED16	0.16182851	0.52436829	-0.1187357
ENSG000001	CLCN2	0.31211054	0.6608083	-0.118617
ENSG000001	CCDC125	0.20500054	0.56891466	-0.1185821
ENSG00000C	REXO5	0.42764703	0.73963622	-0.1185514

ENSG00000C	LASP1	0.0485064	0.360767	-0.11855
ENSG000001	PSMC3IP	0.24844041	0.61459845	-0.1185287
ENSG00000C	TBC1D10A	0.130232	0.48824219	-0.1184018
ENSG000001	MGAT4B	0.09458	0.43984453	-0.1183412
ENSG000001	FAM71E1	0.75503234	0.91006311	-0.1183036
ENSG000001	EML3	0.11697291	0.4728891	-0.1182944
ENSG000001	C1RL	0.03709119	0.3336458	-0.1181974
ENSG000001	HEY2	0.69160824	0.88137846	-0.1181362
ENSG000001	CTDSP2	0.01874933	0.27889987	-0.1181322
ENSG000001	AGO4	0.08707437	0.42907415	-0.1180746
ENSG00000C	TYROBP	0.07843121	0.41430635	-0.1180663
ENSG000001	MISP3	0.40032352	0.72583241	-0.1180028
ENSG000001	PRX	0.22374427	0.59091342	-0.1179699
ENSG000001	TMEM63C	0.14380898	0.50194033	-0.117927
ENSG00000C	ZBTB32	0.43902259	0.74609757	-0.1177673
ENSG000002	MYL5	0.07612159	0.41035399	-0.1177147
ENSG000002	PDXP	0.34099654	0.68203508	-0.1176739
ENSG000001	PF4V1	0.39146356	0.71964297	-0.1176502
ENSG000001	H2BC4	0.22265665	0.59012269	-0.1176002
ENSG000001	BTF3	0.22149081	0.58882965	-0.1175646
ENSG000001	PPP1R14D	0.72488587	0.89669145	-0.1174794
ENSG000001	DYNC2I1	0.19170972	0.55723719	-0.1174015
ENSG000001	GRINA	0.27741082	0.63709819	-0.1172343
ENSG000001	ADGRE5	0.21035958	0.57453195	-0.1172231
ENSG000001	RPLP2	0.24149714	0.60721888	-0.1172224
ENSG000001	PPARD	0.12665096	0.48443621	-0.1171694
ENSG000001	RECQL4	0.29178247	0.64936748	-0.1170885
ENSG000001	UPK1B	0.78443341	0.92028573	-0.1170407
ENSG000001	CCNF	0.33552253	0.67708232	-0.116971
ENSG000001	TONSL	0.27546471	0.63512113	-0.1169134
ENSG000001	ZNF768	0.12789338	0.48588488	-0.11682
ENSG000001	PPOX	0.30328243	0.65599134	-0.1168094
ENSG00000C	TFE3	0.04843209	0.360767	-0.1168001
ENSG000001	PLOD2	0.58460826	0.82887597	-0.1167771
ENSG000001	S100A4	0.1972975	0.56500135	-0.1167556
ENSG000001	PDZD9	0.69274311	0.88158966	-0.1167415
ENSG00000C	NLE1	0.25890839	0.62261049	-0.1167156
ENSG000001	CFAP100	0.53190244	0.80249953	-0.1166964
ENSG000001	CDC42BPA	0.40199809	0.72662172	-0.1165986
ENSG000001	TNS4	0.66912367	0.87125589	-0.116586
ENSG000001	GAMT	0.35861157	0.69382497	-0.1164916
ENSG000001	WWP2	0.10605223	0.4561473	-0.1164332
ENSG000001	CAMKK2	0.01722022	0.27243096	-0.1163624

ENSG000001	THEM5	0.35230551	0.69082603	-0.1163368
ENSG000001	MFSD10	0.1432122	0.50145476	-0.1163354
ENSG00000(	PKP2	0.4935387	0.78107975	-0.1163195
ENSG00000(	TFAP4	0.26966978	0.63105497	-0.1161449
ENSG00000(	RFFL	0.01338036	0.25828987	-0.1161402
ENSG000001	PLBD1	0.12726923	0.48508697	-0.1161345
ENSG000002	GNG14	0.72316127	0.89582373	-0.1160991
ENSG000001	AXIN2	0.40167204	0.72627493	-0.1160426
ENSG000002	SMIM17	0.7824934	0.9193471	-0.1160222
ENSG000001	POP7	0.17617538	0.54121794	-0.1159452
ENSG000001	APC2	0.11979293	0.47604098	-0.1158011
ENSG000001	RMND5A	0.14293077	0.5010464	-0.1157654
ENSG000001	RAB1B	0.09491838	0.44033991	-0.1157568
ENSG000001	TPCN2	0.02612292	0.30558921	-0.1157566
ENSG000002	NUTM2E	0.72689142	0.89706038	-0.1157277
ENSG000001	NR4A2	0.41518585	0.73212128	-0.1157121
ENSG000002	ZNF559-ZNF	0.74835969	0.90731063	-0.1156706
ENSG000001	MT-CO2	0.17232497	0.53663992	-0.1156386
ENSG000001	KIAA1614	0.24258014	0.60820533	-0.1156091
ENSG00000(	CD9	0.26550037	0.62841539	-0.1155114
ENSG000001	PITPNM1	0.12618127	0.48388732	-0.1154862
ENSG000001	GXYLT2	0.77880012	0.91810931	-0.1154515
ENSG000001	DTD1	0.12643287	0.48405814	-0.1154491
ENSG000001	ARID5A	0.12540862	0.48335545	-0.115391
ENSG000001	HSDL2	0.116125	0.47160917	-0.1153852
ENSG000001	CHTF18	0.25401472	0.61928338	-0.1153227
ENSG000001	CYP4F12	0.2581204	0.62198226	-0.115321
ENSG000001	HSPA6	0.12389291	0.48115857	-0.1153105
ENSG000001	CDC42BPG	0.3432313	0.68389331	-0.1152899
ENSG00000(	CDHR2	0.42090271	0.7350227	-0.1152797
ENSG000001	CRTC1	0.12232029	0.47887061	-0.1152615
ENSG000001	NUP62CL	0.725033	0.89675649	-0.1152475
ENSG000001	NUPR2	0.73515941	0.90147373	-0.1152202
ENSG000001	C1orf122	0.20961709	0.5736945	-0.1152128
ENSG000001	PIANP	0.29911225	0.65423918	-0.1151513
ENSG000002	NPTXR	0.5016468	0.7853582	-0.1151191
ENSG000001	NCOR2	0.13686384	0.49714946	-0.115067
ENSG000001	TCIRG1	0.08303096	0.42241031	-0.1150631
ENSG000001	CUEDC1	0.08069715	0.41756804	-0.1150576
ENSG000001	MAMDC2	0.79131574	0.92303115	-0.1150501
ENSG000001	RAD23A	0.09470582	0.43988973	-0.1149921
ENSG000002	KLLN	0.27344564	0.6339378	-0.1149639
ENSG000001	CHSY3	0.77597122	0.91686844	-0.114952

ENSG000001	GRK2	0.02983544	0.31849971	-0.11495
ENSG00000C	TBC1D25	0.08600213	0.42681511	-0.1148785
ENSG000001	SERF2	0.15717053	0.5199629	-0.1148761
ENSG000001	KLK1	0.52363745	0.79870477	-0.1148744
ENSG000001	SHH	0.75777115	0.91086246	-0.1148179
ENSG00000C	MARK2	0.04124136	0.3471186	-0.1147396
ENSG000002	H2AJ	0.12895018	0.4871454	-0.1146734
ENSG00000C	PREX2	0.74879137	0.90739051	-0.1146682
ENSG000001	SEMA4B	0.15785039	0.52077869	-0.1146677
ENSG000001	EME2	0.18962023	0.55424774	-0.1146525
ENSG000002	TEX22	0.40147334	0.72614031	-0.1146217
ENSG000001	IL13	0.81189948	0.93164748	-0.1145712
ENSG000001	MEGF9	0.24667469	0.61251827	-0.1145698
ENSG000001	GALK1	0.1071951	0.45803339	-0.1145686
ENSG000001	ALKAL2	0.72769473	0.8971681	-0.1144782
ENSG000001	ZNF775	0.2099018	0.57415357	-0.1144676
ENSG000001	SLC35G5	0.52349948	0.79870477	-0.1144391
ENSG000001	TFR2	0.22372158	0.59091342	-0.1144378
ENSG000001	SIGLEC5	0.32914118	0.67287758	-0.1143628
ENSG000002	ITGA1	0.30613905	0.65735608	-0.1142238
ENSG000001	ENDOG	0.41495978	0.73204063	-0.1142122
ENSG000002	H4C4	0.35431763	0.69167785	-0.1142119
ENSG000001	YY1AP1	0.05295661	0.36981204	-0.1141956
ENSG00000C	TEAD2	0.39915796	0.72496091	-0.1140935
ENSG000002	TRIM39	0.77082299	0.91453799	-0.1140856
ENSG000001	SNX21	0.26686678	0.62929976	-0.1140842
ENSG000001	WDR93	0.79407	0.92418569	-0.1140422
ENSG000001	ARHGAP1	0.0549276	0.37354464	-0.1139608
ENSG000001	TRIM3	0.11845626	0.47516823	-0.1138608
ENSG000001	GNAI2	0.04181698	0.34903589	-0.1138279
ENSG000001	PLXDC2	0.04855072	0.360767	-0.1138139
ENSG000001	OBSCN	0.3200914	0.66717895	-0.1137321
ENSG000002	ARHGAP19	0.10572775	0.4561473	-0.1135751
ENSG000001	CR1L	0.47310669	0.76859183	-0.1135528
ENSG00000C	XK	0.4976464	0.78278241	-0.1133758
ENSG000001	STK11	0.10941315	0.46193514	-0.113354
ENSG000001	NOS3	0.36580934	0.69837111	-0.1133406
ENSG000001	S100A9	0.19238416	0.55820972	-0.1133316
ENSG00000C	NCK2	0.06359879	0.38981851	-0.1133114
ENSG000001	HAAO	0.15308083	0.5142759	-0.1132177
ENSG000001	CYSRT1	0.7593936	0.91126109	-0.1131803
ENSG000001	KCNJ6	0.84730932	0.94527257	-0.1131654
ENSG000001	ALS2CL	0.37737306	0.7086984	-0.113163

ENSG000001	AGO2	0.24940432	0.61525038	-0.1130709
ENSG000001	PLXNC1	0.21645895	0.58303866	-0.113066
ENSG000001	NFKBID	0.09308041	0.43758758	-0.113037
ENSG000001	COQ10A	0.31695683	0.66422665	-0.1130032
ENSG000001	GCOM1	0.6516952	0.86352007	-0.1129417
ENSG000001	ATG4B	0.09343618	0.4384324	-0.1128131
ENSG00000C	TBC1D2	0.04069383	0.34551949	-0.1128033
ENSG000001	DCST2	0.37284905	0.70484923	-0.1127579
ENSG000001	ESRRB	0.83322776	0.93878551	-0.1127028
ENSG000001	CDK1	0.69129849	0.88131945	-0.1126427
ENSG000001	DRC7	0.37995067	0.70984455	-0.112627
ENSG00000C	RASGRP2	0.13106609	0.4894044	-0.1126183
ENSG000001	M1AP	0.43725049	0.74497757	-0.1124119
ENSG000001	ZNF215	0.8112932	0.93148672	-0.1123314
ENSG000001	RNF175	0.15113558	0.51190876	-0.112281
ENSG000001	LGR5	0.80998005	0.93062879	-0.1122544
ENSG000001	MICAL1	0.06199894	0.38624952	-0.1122508
ENSG000001	COL6A2	0.37802488	0.70909881	-0.1121884
ENSG000002	FADS3	0.30736427	0.65762361	-0.1121711
ENSG000001	KRBA1	0.20790153	0.57145912	-0.1121467
ENSG000001	PITPNC1	0.22546988	0.59296313	-0.1119791
ENSG000001	BRMS1	0.04698341	0.35811287	-0.1119463
ENSG000001	ALKBH2	0.30765185	0.65762361	-0.1119152
ENSG000001	NT5DC3	0.37054923	0.70312117	-0.1118618
ENSG000001	MYH14	0.72672218	0.89706038	-0.1118468
ENSG000002	PPP1R11	0.5583459	0.81600055	-0.1118061
ENSG000001	SLC22A7	0.8022297	0.92757225	-0.1117806
ENSG000001	ZMAT2	0.16166824	0.52427832	-0.1117058
ENSG000002	FIS1	0.1581475	0.52089921	-0.111688
ENSG000002	MTRNR2L12	0.47303176	0.76859183	-0.1116872
ENSG000001	SSNA1	0.12641669	0.48405814	-0.1116801
ENSG000001	TSHZ2	0.33704239	0.67898382	-0.1116761
ENSG000001	CERK	0.20324039	0.56867381	-0.1116714
ENSG00000C	NRP1	0.52855064	0.80071625	-0.1116588
ENSG000001	FGFBP1	0.81775719	0.9333228	-0.1116418
ENSG000001	PRC1	0.7218895	0.89512361	-0.1116339
ENSG000001	MRPL55	0.22468975	0.59200912	-0.1115085
ENSG000001	PCIF1	0.03867925	0.33829709	-0.1114945
ENSG000002	SLC26A6	0.18624834	0.55069275	-0.1114054
ENSG000001	EFCAB6	0.7923842	0.92314956	-0.1113399
ENSG000001	MKRN1	0.27513218	0.63499184	-0.1113333
ENSG000001	ING2	0.13276842	0.49139472	-0.111218
ENSG00000C	PPP1R15A	0.07271152	0.40432385	-0.1112015

ENSG000001	NOTCH1	0.20960704	0.5736945	-0.1111805
ENSG000002	BORCS8	0.02453045	0.29927225	-0.1111728
ENSG000001	GMEB2	0.05030616	0.36227834	-0.1111275
ENSG000001	MED11	0.13165216	0.4897219	-0.1110993
ENSG000001	NSUN5	0.10000977	0.44762396	-0.1110232
ENSG00000C	STK10	0.04223574	0.34997262	-0.1110121
ENSG000001	SLC25A34	0.30396094	0.65635638	-0.1109325
ENSG000001	DNTTIP1	0.00437828	0.21740126	-0.1109235
ENSG000001	BCL2L10	0.79210629	0.92314956	-0.1108727
ENSG000001	FAAP20	0.20401707	0.5688169	-0.1108578
ENSG000001	ANKRD11	0.0300525	0.31859418	-0.110742
ENSG000001	SLC2A1	0.18146309	0.54626754	-0.1107359
ENSG000001	GALE	0.12848149	0.48678709	-0.1107242
ENSG000001	ZFP41	0.25615443	0.62056088	-0.1105292
ENSG000001	SPATA2	0.00866972	0.23436355	-0.1105113
ENSG00000C	VMP1	0.07960086	0.4152849	-0.1104645
ENSG000002	C19orf38	0.03039071	0.3191802	-0.1104044
ENSG00000C	WWC3	0.02648163	0.30699826	-0.110346
ENSG000001	TBKBP1	0.15787023	0.52077869	-0.110261
ENSG000001	LDB1	0.03104769	0.31953017	-0.1102405
ENSG000001	PAPLN	0.15185314	0.51292686	-0.1101835
ENSG000001	TRIB1	0.1265263	0.48430163	-0.1101047
ENSG00000C	HMGB3	0.2997047	0.6542516	-0.1100337
ENSG00000C	GPC4	0.67296344	0.87354948	-0.1100298
ENSG000001	SEPTIN3	0.74987796	0.90798817	-0.1099422
ENSG000001	CASP9	0.01135246	0.24556914	-0.1097731
ENSG000001	PPP1R9B	0.0478175	0.35895177	-0.1097435
ENSG000001	USP21	0.09971107	0.44746644	-0.1097053
ENSG000001	SFI1	0.18626206	0.55069275	-0.109665
ENSG000001	APOBEC2	0.33141733	0.67444298	-0.1096213
ENSG000001	CD34	0.50116492	0.7853582	-0.1096197
ENSG000001	FAM102A	0.32013729	0.66718895	-0.1095941
ENSG000002	HLA-DMB	0.61039744	0.84313256	-0.1095128
ENSG000001	SAP30	0.29893248	0.65423918	-0.1094895
ENSG000001	MFNG	0.10132244	0.44962352	-0.1094554
ENSG00000C	BAD	0.19700055	0.56464218	-0.1094499
ENSG000001	C8G	0.62501723	0.85016376	-0.1094491
ENSG000001	PGP	0.20854085	0.57228774	-0.1094082
ENSG000002	INAFM2	0.07052003	0.40156223	-0.1093533
ENSG000001	SLC14A1	0.4301275	0.74091639	-0.109324
ENSG000001	HCFC1R1	0.24293875	0.60854621	-0.1092713
ENSG000001	PRR5	0.21645846	0.58303866	-0.1092548
ENSG000002	INAFM1	0.26482585	0.6274921	-0.1091832

ENSG000001	APP	0.13392416	0.4925946	-0.1091633
ENSG000001	PIK3IP1	0.28082344	0.63927558	-0.1091529
ENSG00000(	PTGS1	0.28433966	0.64253645	-0.1090866
ENSG000001	SIN3B	0.13859948	0.49898885	-0.1090856
ENSG00000(	FGFR1	0.31349844	0.66161883	-0.1090321
ENSG000001	SCAMP5	0.19898669	0.56598606	-0.1089963
ENSG00000(	ATP6V0A1	0.00914489	0.23551879	-0.108992
ENSG00000(	TSR3	0.19631158	0.5638811	-0.1089276
ENSG000001	EFNB2	0.61366497	0.8451435	-0.1089003
ENSG000001	ARL8A	0.03486436	0.32855052	-0.1088716
ENSG000001	NBEA	0.44312982	0.74887084	-0.1088709
ENSG000001	CD300LB	0.0242176	0.29927225	-0.1088578
ENSG000001	IL16	0.03574156	0.33014896	-0.1087579
ENSG000001	OSCP1	0.39969137	0.72527731	-0.1087574
ENSG000001	PHETA2	0.45736167	0.75742878	-0.1087328
ENSG000001	CCNI	0.02435754	0.29927225	-0.1086473
ENSG00000(	MNT	0.02817803	0.31251818	-0.1086117
ENSG000001	TLCD1	0.5439338	0.80884121	-0.1085395
ENSG000001	KATNBL1	0.40637555	0.7275473	-0.1084256
ENSG000001	EVA1C	0.38259237	0.71172172	-0.1084124
ENSG000001	CPSF7	0.04421833	0.35453012	-0.1083636
ENSG000001	POMT1	0.28867253	0.64641785	-0.1082669
ENSG000001	WASF2	0.03763362	0.33471496	-0.108251
ENSG000001	LPCAT4	0.27387317	0.63409078	-0.10824
ENSG000001	ARPC1B	0.11215673	0.46614115	-0.1082366
ENSG000001	PPP1R12C	0.05630976	0.37502119	-0.1081734
ENSG00000(	AGO1	0.04529937	0.35545023	-0.1081156
ENSG00000(	ACAP1	0.15984129	0.52241203	-0.1080868
ENSG000001	MAP3K11	0.12600657	0.48388732	-0.1080069
ENSG000001	CPM	0.18136284	0.54624161	-0.1079532
ENSG000001	UBAP1	0.03838936	0.33693407	-0.1079205
ENSG000001	KRTCAP3	0.46905993	0.76620653	-0.1078924
ENSG000001	HSD17B13	0.2368557	0.60364584	-0.1078674
ENSG000001	PTPRJ	0.18947629	0.55423532	-0.1078663
ENSG000002	N/A	0.73542401	0.90150345	-0.1078663
ENSG000001	SSBP3	0.21032897	0.57453195	-0.1078182
ENSG000001	TMPRSS11D	0.80428711	0.92881463	-0.1077881
ENSG00000(	ATP5F1D	0.2189299	0.58591803	-0.1077115
ENSG000001	SNPH	0.3543374	0.69167785	-0.1076825
ENSG000001	ZNF341	0.04950774	0.36128416	-0.10768
ENSG00000(	CRMP1	0.60271579	0.8385858	-0.1076338
ENSG000001	MXD4	0.24868583	0.61459845	-0.1074737
ENSG000001	RNPEP	0.04299364	0.35185871	-0.1073842

ENSG000001	TSACC	0.7063315	0.88808305	-0.1073736
ENSG000001	NDRG3	0.20660901	0.57023806	-0.1073445
ENSG000001	YIPF2	0.13489897	0.49418805	-0.1073035
ENSG000001	HECTD2	0.65266832	0.86379349	-0.107256
ENSG000001	MINK1	0.12050849	0.47724625	-0.1072087
ENSG000001	CCDC113	0.78945245	0.92258093	-0.1071513
ENSG00000(	LYPLA2	0.08304082	0.42241031	-0.1071265
ENSG000001	CCDC30	0.22191403	0.58912768	-0.1070843
ENSG000001	ZNF662	0.66690225	0.87009558	-0.1069123
ENSG000001	MSC	0.28342565	0.64165879	-0.1068503
ENSG000001	GPR137	0.08152843	0.4189168	-0.1068314
ENSG000001	CXCR4	0.06120183	0.38491421	-0.1067698
ENSG00000(	ROGDI	0.18702296	0.55108473	-0.1067338
ENSG000001	MEIS1	0.36540512	0.69833839	-0.1067167
ENSG000001	GRIK4	0.78655639	0.92119044	-0.1066944
ENSG000001	GABRR1	0.74485442	0.90628646	-0.1066932
ENSG000001	CYB5R3	0.14913273	0.50913963	-0.1066836
ENSG000001	NCF2	0.04737505	0.35865951	-0.1066241
ENSG000001	CCNE2	0.33353983	0.67601673	-0.1064302
ENSG000001	DAB1	0.68231924	0.87750056	-0.1063928
ENSG00000(	CCDC80	0.72162671	0.89490971	-0.1063715
ENSG000001	TADA2B	0.04844351	0.360767	-0.1063654
ENSG000001	RSPH4A	0.60270913	0.8385858	-0.1063442
ENSG000001	TIAM2	0.16455681	0.52786788	-0.1063437
ENSG000001	MYL6B	0.34674201	0.68642404	-0.1063416
ENSG000001	RAC2	0.0614241	0.38542193	-0.1063353
ENSG000001	IFT140	0.31191916	0.66057537	-0.1062434
ENSG000001	SIPA1L2	0.43938941	0.74621708	-0.1062356
ENSG00000(	PPP2R5B	0.15075993	0.51136179	-0.1062286
ENSG000001	AKT1	0.06460315	0.39055819	-0.1061715
ENSG000001	PAPPA	0.79266156	0.92320822	-0.1061462
ENSG000001	C16orf86	0.20128213	0.56773237	-0.1061308
ENSG000001	TRAPPC12	0.2242761	0.59169817	-0.106126
ENSG000001	MTFMT	0.14960451	0.50925123	-0.1061061
ENSG000001	SLC12A4	0.02729301	0.31011657	-0.1060652
ENSG00000(	CCDC124	0.15882861	0.52170151	-0.1060465
ENSG000002	N/A	0.71196909	0.89093319	-0.1059899
ENSG000001	DAPK3	0.09699757	0.44311879	-0.1059854
ENSG000001	KLF7	0.09026282	0.43395124	-0.1059288
ENSG000001	PCED1B	0.28681456	0.64421192	-0.1058977
ENSG000001	SLC38A10	0.10981163	0.46255597	-0.1058692
ENSG000002	SMIM3	0.11901355	0.47567495	-0.1058375
ENSG000001	GNG8	0.73053334	0.89847521	-0.1058297

ENSG000001	RPL13	0.36155921	0.69588937	-0.1058109
ENSG00000C	PTPN23	0.08744989	0.42962903	-0.1058038
ENSG000001	STKLD1	0.55512967	0.81438747	-0.1057848
ENSG000001	CIZ1	0.12297414	0.47983319	-0.1057757
ENSG000001	DDX39B	0.6395093	0.85691647	-0.1057251
ENSG00000C	ZFAT	0.02364898	0.29875884	-0.1057096
ENSG000001	DCAF15	0.08417214	0.42391533	-0.1056833
ENSG000002	NHSL2	0.0603589	0.38403111	-0.1056655
ENSG000001	MBP	0.07539984	0.40963494	-0.1056395
ENSG000001	NR1D1	0.32219869	0.66822584	-0.1055897
ENSG000001	GLDC	0.72836747	0.89755262	-0.1054799
ENSG000001	RNF167	0.01640549	0.27000801	-0.1053104
ENSG000001	PODXL	0.24361263	0.60937379	-0.1052972
ENSG000001	MT-ND5	0.33384938	0.67615222	-0.1052959
ENSG000001	RPS12	0.36435076	0.69739047	-0.1052949
ENSG000001	CD320	0.30408678	0.65635638	-0.1052143
ENSG000001	TFPT	0.75876698	0.91112183	-0.105209
ENSG00000C	NTHL1	0.45696301	0.75721169	-0.1051612
ENSG000001	RPL36AL	0.11750171	0.4740063	-0.1051464
ENSG000001	SYK	0.01428883	0.2599395	-0.1051362
ENSG000001	MCM5	0.10625661	0.45618418	-0.1051274
ENSG000001	KIF13A	0.17569914	0.54061531	-0.1050834
ENSG000001	TREX2	0.4009023	0.72606468	-0.1050167
ENSG000001	DSN1	0.20424121	0.5688169	-0.1049528
ENSG00000C	LAT2	0.01733352	0.27265457	-0.1049341
ENSG000001	DVL3	0.00947271	0.23577638	-0.1049297
ENSG000001	TPST2	0.17080484	0.53457173	-0.1048728
ENSG000001	JAM2	0.47011413	0.7662622	-0.1047622
ENSG000001	MED22	0.11159893	0.46521998	-0.104752
ENSG000001	CCR7	0.3537149	0.69146026	-0.1047508
ENSG000001	TRPV1	0.2646702	0.6274921	-0.1046592
ENSG000001	KBTBD7	0.3798932	0.70981886	-0.1045772
ENSG000001	LGI4	0.75985817	0.91136366	-0.1043745
ENSG000001	FGFR4	0.55571221	0.81461822	-0.1043108
ENSG000001	ZFAND3	0.07710575	0.41179232	-0.1042952
ENSG000001	SIGIRR	0.32625934	0.67170284	-0.104163
ENSG00000C	DNMT3B	0.56331511	0.81863326	-0.1041162
ENSG000001	ATP5F1E	0.2595797	0.6232076	-0.1040862
ENSG000001	OR56B2P	0.82276736	0.93539963	-0.1040673
ENSG00000C	ITPKC	0.06882802	0.39825823	-0.1040454
ENSG000001	EPHB6	0.36461406	0.69769659	-0.1039698
ENSG000001	MAPKAPK2	0.03462029	0.32831054	-0.103819
ENSG000001	RPS25	0.49952471	0.78410505	-0.1036584

ENSG000001	DEPTOR	0.6498318	0.86288182	-0.1036453
ENSG000001	WDR91	0.14775412	0.50645633	-0.1036122
ENSG00000C	MOK	0.3729737	0.70500278	-0.1035812
ENSG000001	SRF	0.04151135	0.34755298	-0.1035464
ENSG000001	VAV1	0.01723142	0.27243096	-0.1034885
ENSG000001	TUBB2B	0.82965982	0.93686532	-0.1033343
ENSG00000C	ZNF213	0.13840445	0.49898885	-0.1033337
ENSG000002	ZSWIM8	0.04969061	0.36170272	-0.1032705
ENSG000001	IGFBP3	0.52031184	0.79709497	-0.1032697
ENSG00000C	SULT2B1	0.5662484	0.8197023	-0.1032659
ENSG000001	PARL	0.29744899	0.65274757	-0.1032555
ENSG000001	GATM	0.52145173	0.79758937	-0.103251
ENSG000001	ZNRD2	0.20587306	0.56956674	-0.1032166
ENSG000001	GTDC1	0.14267408	0.50078343	-0.1031641
ENSG000001	BRI3	0.07680764	0.41105	-0.1031128
ENSG000001	CARNS1	0.43066616	0.74122605	-0.1030909
ENSG000002	PPAN-P2RY1	0.76359432	0.91212248	-0.1030501
ENSG000001	EPHA1	0.26229459	0.62579547	-0.1029945
ENSG000001	NFIB	0.66410325	0.86837979	-0.1029866
ENSG000001	LRP4	0.76409685	0.91243616	-0.1029101
ENSG000001	ELFN2	0.54787709	0.81141647	-0.1028601
ENSG000001	TRPM2	0.20081498	0.56765893	-0.1028372
ENSG000001	PPP4R1	0.11557944	0.4710215	-0.1027653
ENSG000002	TXNDC5	0.6258954	0.85056163	-0.1027372
ENSG00000C	MAGI3	0.41409851	0.73141372	-0.1026992
ENSG000001	ZNF592	0.02788226	0.31135087	-0.1026843
ENSG000001	SNRNP70	0.16359941	0.52709285	-0.1026557
ENSG00000C	SLC7A9	0.6330289	0.85440485	-0.1025476
ENSG000001	DNAI4	0.75578457	0.9104345	-0.1025405
ENSG000002	DENND1C	0.11504249	0.47079584	-0.1025377
ENSG000001	COQ8B	0.11759897	0.47416303	-0.1024762
ENSG000001	SAMD4B	0.02287448	0.29500624	-0.1024118
ENSG000001	SMPD1	0.17297387	0.53752663	-0.1024015
ENSG000001	ALDH1A3	0.82835367	0.93662954	-0.1023738
ENSG000001	WASHC1	0.10467671	0.45514301	-0.1023558
ENSG000001	SLC35E2B	0.16144016	0.52426078	-0.102351
ENSG000001	SPATA18	0.814266	0.93216232	-0.1023403
ENSG000001	CATSPERB	0.83769477	0.94141381	-0.1023144
ENSG000001	SH3TC2	0.40390489	0.72672584	-0.102295
ENSG000001	ST6GAL2	0.84013502	0.94245133	-0.1022806
ENSG00000C	MCOLN3	0.76735707	0.91352929	-0.1020743
ENSG000001	SERINC2	0.31635159	0.66372668	-0.1020573
ENSG000001	SURF2	0.31832789	0.66581089	-0.1020091

ENSG000001	CERS2	0.06986619	0.4002892	-0.1018925
ENSG000001	CCS	0.21810455	0.58515592	-0.1018328
ENSG000001	BCO2	0.53822364	0.80611911	-0.1017572
ENSG000001	KLHL8	0.17925337	0.54453451	-0.1017569
ENSG000001	GPR108	0.03520346	0.32931219	-0.1017527
ENSG000002	OST4	0.20251471	0.56807486	-0.1017266
ENSG000001	KLKB1	0.52246661	0.7979767	-0.1017118
ENSG000001	CDK19	0.21930108	0.58662135	-0.1016948
ENSG000001	ARHGAP25	0.05731506	0.37687699	-0.1016818
ENSG000001	GADD45A	0.29210345	0.64944907	-0.1016782
ENSG000001	ARMC7	0.08522682	0.42526981	-0.1016764
ENSG000001	HGS	0.08332564	0.42260872	-0.1016589
ENSG00000C	ZNF446	0.25669446	0.62074989	-0.1016334
ENSG000001	ELOVL7	0.49505987	0.78124152	-0.1015493
ENSG000002	MYO15B	0.17127319	0.53539947	-0.1015473
ENSG000001	TUFT1	0.16165354	0.52427832	-0.1014974
ENSG000001	LRSAM1	0.09005915	0.43377006	-0.1014395
ENSG000001	SHC3	0.81216175	0.93168506	-0.101391
ENSG00000C	SEZ6	0.30491447	0.65635638	-0.1013521
ENSG000001	EPB41	0.13814065	0.49898885	-0.1013182
ENSG000001	BMP8A	0.76059464	0.91141904	-0.1012818
ENSG000001	ISCU	0.11456297	0.47021209	-0.101241
ENSG000001	ITPKB	0.18889049	0.55371274	-0.1012317
ENSG000001	MT-ND4	0.29919295	0.65423918	-0.1012296
ENSG000001	CBX1	0.12924875	0.4871968	-0.1011997
ENSG000001	UBL4A	0.18266841	0.54696818	-0.1011892
ENSG000001	HCK	0.17503926	0.53975461	-0.1011588
ENSG000001	ZNF652	0.02348971	0.29875884	-0.1011552
ENSG000001	PBX4	0.40095087	0.72606468	-0.101037
ENSG000002	TP53TG3D	0.79544384	0.92462665	-0.1010183
ENSG00000C	KLHL13	0.58511498	0.82919554	-0.1009992
ENSG000001	EFHD2	0.11325681	0.46840315	-0.1009695
ENSG000001	LMLN	0.47953518	0.77351606	-0.1009423
ENSG000001	PI4K2A	0.16420571	0.52764855	-0.1009199
ENSG000001	NUSAP1	0.5502023	0.81237017	-0.1008395
ENSG000002	EPPK1	0.61134504	0.84343695	-0.1008183
ENSG000001	DHRS3	0.4198563	0.73466853	-0.1007636
ENSG000001	MAP7D2	0.74844664	0.90731063	-0.1007144
ENSG000001	PCBP1	0.11519889	0.47091753	-0.1006974
ENSG000001	TCAP	0.35319305	0.69126085	-0.1006452
ENSG000002	LIN37	0.13691957	0.49724068	-0.1005989
ENSG000001	AURKAIP1	0.09700012	0.44311879	-0.1005675
ENSG000002	N/A	0.59730369	0.83635909	-0.1004312

ENSG000002	PLXNA4	0.62591235	0.85056163	-0.1003852
ENSG000001	DIPK1B	0.35181352	0.6905543	-0.1003133
ENSG000001	KIF21B	0.10137856	0.4496268	-0.1002059
ENSG000001	AURKC	0.50491322	0.78768984	-0.1000804
ENSG000001	MT-CO3	0.18034978	0.54515554	-0.10003
ENSG000001	ASL	0.24923983	0.61514875	-0.0999237
ENSG000001	AFAP1L1	0.81364605	0.9318894	-0.099844
ENSG000001	KCNAB3	0.3163135	0.66372668	-0.0998233
ENSG00000C	ARHGEF1	0.08573568	0.42602033	-0.0997784
ENSG00000C	SF3A1	0.12120545	0.47724625	-0.0997055
ENSG000001	PPBP	0.45501917	0.7559601	-0.0996741
ENSG000001	HGSNAT	0.08782064	0.43050213	-0.0996289
ENSG00000C	RTF2	0.02774187	0.3113392	-0.0996129
ENSG000001	AQP3	0.40477514	0.72672584	-0.0996095
ENSG000001	PGPEP1	0.15173729	0.5128835	-0.0995767
ENSG000001	MCRIP2	0.31103438	0.65949478	-0.0995694
ENSG000001	USF2	0.08287224	0.42228867	-0.0995601
ENSG000001	CD27	0.41552467	0.73221244	-0.0995508
ENSG000001	LHPP	0.18526249	0.55032535	-0.0994934
ENSG00000C	ADD1	0.04581455	0.35661113	-0.0994599
ENSG000001	FEM1A	0.24553125	0.61145565	-0.099376
ENSG000001	ZNF771	0.66302442	0.86790774	-0.0993734
ENSG00000C	CTDP1	0.08720311	0.42932594	-0.0993594
ENSG000001	ADAMTS10	0.54308584	0.8084284	-0.0993494
ENSG000001	TMC8	0.27017552	0.63142427	-0.0993206
ENSG000001	OSER1	0.05862522	0.38045469	-0.0993
ENSG000001	TMEM11	0.06427415	0.39043354	-0.0992442
ENSG000001	EFNA5	0.69563045	0.88231682	-0.0992314
ENSG000001	PNKD	0.09966402	0.44746644	-0.0992304
ENSG000001	MEPCE	0.04790371	0.35943277	-0.0991747
ENSG00000C	PLD1	0.16580736	0.52885285	-0.0991721
ENSG000001	VCAM1	0.7929323	0.92345731	-0.09902
ENSG00000C	E2F2	0.1974793	0.56521618	-0.0989727
ENSG000002	GRIFIN	0.76752891	0.91352929	-0.098946
ENSG000001	RAB5B	0.00877582	0.23436355	-0.0989341
ENSG000001	ZNF205	0.48535247	0.7771862	-0.0988865
ENSG000001	CLMN	0.09445803	0.43981122	-0.0988844
ENSG000001	ZNF362	0.13150609	0.4894044	-0.0988776
ENSG000001	KHK	0.49477256	0.78124152	-0.0988539
ENSG000001	CLEC17A	0.26504135	0.62760338	-0.0988117
ENSG000001	CNNM3	0.22484421	0.59200912	-0.0987886
ENSG000001	PPP2R5D	0.16115201	0.52412971	-0.0987593
ENSG000001	SIX5	0.80886871	0.93001	-0.0987535

ENSG000001	ZBTB20	0.40767042	0.72771726	-0.0987233
ENSG000001	AZIN2	0.43589584	0.74462291	-0.0986083
ENSG000001	RIMS3	0.33277709	0.67523724	-0.098584
ENSG000001	SPTBN4	0.68459281	0.87906157	-0.0985448
ENSG00000C	OSBPL7	0.27204478	0.63323084	-0.0985139
ENSG000001	CCDC159	0.07229892	0.40373033	-0.098352
ENSG000001	MEF2D	0.06322666	0.38916273	-0.0983063
ENSG000001	C1orf87	0.75929667	0.91125749	-0.0983044
ENSG000001	NR2F6	0.4145502	0.73184916	-0.0982964
ENSG000001	PLEKHG5	0.56946089	0.82041807	-0.0982927
ENSG000001	PCDH8	0.71103014	0.89054277	-0.0982851
ENSG000001	SORBS3	0.31241581	0.66090624	-0.0982538
ENSG00000C	FAT1	0.82538418	0.93591669	-0.0982189
ENSG000001	CBLC	0.80271893	0.92789015	-0.0981276
ENSG000001	OR52W1	0.83307832	0.93878551	-0.0981223
ENSG000001	CCND3	0.10536865	0.45574608	-0.0980659
ENSG000002	FNTB	0.11424238	0.46971445	-0.0980446
ENSG000002	ZNF580	0.22268164	0.59012269	-0.0980067
ENSG000001	ZNF784	0.15174554	0.5128835	-0.0979687
ENSG000001	RAPGEF1	0.08775244	0.43050213	-0.097961
ENSG000001	MT-ATP6	0.25421677	0.619382	-0.0979211
ENSG000001	SLC2A4RG	0.32959605	0.67333304	-0.097749
ENSG000001	SIVA1	0.25140122	0.61698936	-0.0977051
ENSG00000C	FOSL2	0.2713544	0.63286144	-0.0976633
ENSG000001	CRHBP	0.60395149	0.83883394	-0.0976278
ENSG000001	RANGRF	0.4528738	0.75444669	-0.0975695
ENSG000001	LRPAP1	0.06535041	0.39169239	-0.0975629
ENSG00000C	TNFRSF1B	0.08346005	0.42260872	-0.0975363
ENSG000001	DPYSL4	0.59602287	0.83579095	-0.0974408
ENSG000001	UBC	0.02803698	0.31159655	-0.09744
ENSG000001	SAFB2	0.04490644	0.35545023	-0.0973881
ENSG00000C	IL4R	0.23614628	0.60336718	-0.0973848
ENSG000001	CETN2	0.11371005	0.46915085	-0.09737
ENSG00000C	PABPC4	0.25990661	0.62338681	-0.0973462
ENSG000001	OR2T34	0.84214591	0.9429376	-0.0973352
ENSG000001	SLC9A8	0.05311988	0.36981204	-0.0972826
ENSG000001	RFX8	0.70977363	0.88993007	-0.0972568
ENSG000001	FUT6	0.84172344	0.94283868	-0.097245
ENSG000001	NAMPT	0.38739721	0.71666498	-0.0972415
ENSG000001	ANP32B	0.25529476	0.62026352	-0.0972392
ENSG00000C	WIPI1	0.0456046	0.3556589	-0.0972356
ENSG000001	LRRC61	0.26682108	0.62929976	-0.0971462
ENSG000001	RGS10	0.29679712	0.65219851	-0.097125

ENSG000002	QTRT1	0.32719497	0.67217574	-0.0970979
ENSG000001	MTARC2	0.62304086	0.84930015	-0.0970506
ENSG000001	SIRT7	0.03682331	0.33341755	-0.0970395
ENSG000001	MAFG	0.10943122	0.46193514	-0.0970145
ENSG000001	SPRYD3	0.06454535	0.39055819	-0.0970129
ENSG000001	MT-ND6	0.39540051	0.72209218	-0.0969403
ENSG000001	TMEM43	0.0756058	0.41015711	-0.0969246
ENSG000001	MCM7	0.26343127	0.62687307	-0.0968852
ENSG000001	MYC	0.26699988	0.62929976	-0.0967553
ENSG000001	PPP1R14A	0.3789434	0.70965485	-0.0966426
ENSG000001	SQSTM1	0.07439621	0.40673667	-0.0966255
ENSG000001	IL22	0.79585156	0.9246397	-0.0965635
ENSG000001	ASTL	0.68150712	0.87697188	-0.0965042
ENSG000001	IFT172	0.39476531	0.72193673	-0.096426
ENSG000001	CLTB	0.11638467	0.47160917	-0.0964229
ENSG000001	UNC119B	0.23062531	0.59855451	-0.0964163
ENSG000001	ENPP3	0.68676568	0.88023892	-0.0964102
ENSG000001	TMUB2	0.02258434	0.29448836	-0.0964096
ENSG000001	NPRL2	0.26491099	0.6274921	-0.0962848
ENSG000001	ABCA2	0.24624823	0.61214306	-0.0962129
ENSG000001	FGGY	0.40816724	0.72796698	-0.0961688
ENSG000001	ZNF646	0.03218213	0.32322672	-0.0961418
ENSG000001	BCAT2	0.25828457	0.62209627	-0.0960962
ENSG000001	WDR90	0.40361556	0.72672584	-0.0960853
ENSG00000(	SRPK1	0.16903018	0.53299639	-0.0959996
ENSG000001	HEXIM2	0.22699956	0.59448256	-0.0959729
ENSG000001	SRPK2	0.16587439	0.52885285	-0.0959035
ENSG000001	GSTM3	0.56472289	0.81898782	-0.0958495
ENSG000002	YJEFN3	0.26891078	0.63079288	-0.0958479
ENSG000001	SLBP	0.19900317	0.56598606	-0.0957769
ENSG000001	PIGU	0.30400343	0.65635638	-0.0957483
ENSG000001	INTS1	0.18293828	0.54743253	-0.0957049
ENSG000002	CEACAM16	0.72866482	0.89755543	-0.0956798
ENSG000001	EHD1	0.24244709	0.60797119	-0.0956614
ENSG000001	RAB32	0.05842355	0.3800398	-0.0956101
ENSG000001	SNN	0.03007136	0.31859418	-0.0956024
ENSG000001	MED26	0.21227223	0.57742741	-0.0954664
ENSG000001	ELMO1	0.06791255	0.39726872	-0.0954604
ENSG000001	NUP98	0.08054418	0.41742606	-0.095423
ENSG000001	MARVELD3	0.82363076	0.93558785	-0.0953804
ENSG000001	NT5C3B	0.33844864	0.68012259	-0.0953546
ENSG000001	PARVB	0.13144863	0.4894044	-0.0953052
ENSG000001	SLC7A3	0.77681022	0.91723328	-0.0952649

ENSG00000C	AP1M1	0.08574456	0.42602033	-0.0952496
ENSG000001	CCNB2	0.6871996	0.88042583	-0.0952265
ENSG000001	NUMB	0.14755578	0.50620393	-0.0951941
ENSG00000C	DGKD	0.20613788	0.56956674	-0.0951744
ENSG000002	FNIP1	0.30554514	0.65695881	-0.0951588
ENSG000001	RPL38	0.45162061	0.75381609	-0.0950709
ENSG000001	COX6B1	0.11810298	0.4748744	-0.0950475
ENSG000001	PTCD1	0.15955206	0.52219103	-0.0949496
ENSG00000C	CD6	0.40342408	0.72672584	-0.0949307
ENSG000001	RPL37	0.49686805	0.78237677	-0.0946361
ENSG000001	SH2B2	0.30986262	0.65931585	-0.094585
ENSG000001	NDOR1	0.09065259	0.43454471	-0.0945557
ENSG000001	PFN1	0.12735917	0.48531585	-0.0945449
ENSG000001	FRG1	0.79843008	0.92603117	-0.0945146
ENSG000002	TECPR1	0.14760223	0.50621491	-0.0944633
ENSG000001	BRF2	0.16429341	0.52769983	-0.094425
ENSG000001	CRIP3	0.45516275	0.75598175	-0.0944108
ENSG000001	PPP1R32	0.33652778	0.67836146	-0.0943959
ENSG000001	SLC28A2	0.80806911	0.92978846	-0.0943267
ENSG000001	ZNF589	0.38665496	0.71586278	-0.0941719
ENSG000001	VPS51	0.24908751	0.61496823	-0.094156
ENSG000001	COPE	0.15230257	0.51327042	-0.0941444
ENSG000001	SH3GL1	0.13389554	0.4925946	-0.0941419
ENSG000001	SH3RF2	0.8125974	0.93170624	-0.0940482
ENSG000001	CTCFL	0.7795604	0.91827559	-0.0939918
ENSG00000C	HCN2	0.32103319	0.66766831	-0.0939333
ENSG000001	TYMS	0.55529699	0.81451209	-0.0938766
ENSG000001	FSTL1	0.47446998	0.76939669	-0.0938757
ENSG00000C	XYLT2	0.26540277	0.62834342	-0.0937751
ENSG000001	MSN	0.14854456	0.50798213	-0.0937663
ENSG000001	MYLK4	0.54084868	0.80750025	-0.0937257
ENSG000001	SLC12A6	0.11181042	0.46550406	-0.0937031
ENSG000002	N/A	0.33886674	0.68041384	-0.0936285
ENSG000001	PCTP	0.07290945	0.40435477	-0.0936275
ENSG000002	N/A	0.78820818	0.92204735	-0.0935496
ENSG000001	FAM110A	0.17346031	0.53805368	-0.0935445
ENSG000001	COL4A1	0.78119465	0.9189743	-0.0934975
ENSG000001	MYOT	0.60646368	0.84005722	-0.0934837
ENSG000002	N/A	0.78788696	0.92194585	-0.093474
ENSG00000C	TECR	0.29608943	0.65199276	-0.0934573
ENSG000002	BUB1B-PAK6	0.76905759	0.91375182	-0.0934176
ENSG000001	DNAJC5	0.06243887	0.38636432	-0.0934166
ENSG00000C	POLR2E	0.09277965	0.43753271	-0.0933643

ENSG000001	B4GALT5	0.33739647	0.67926926	-0.0933369
ENSG000001	MYG1	0.23050112	0.59855451	-0.0933361
ENSG000001	ZNF687	0.08445262	0.42466929	-0.0932717
ENSG000001	B4GAT1	0.50720612	0.79005235	-0.0932544
ENSG00000C	ACTB	0.18120149	0.54622749	-0.0932407
ENSG000002	NCOA4	0.21850781	0.58528507	-0.0932407
ENSG000001	CCDC28B	0.40460945	0.72672584	-0.093151
ENSG00000C	BOD1L1	0.13295587	0.49180844	-0.0930883
ENSG000001	APLP1	0.80806229	0.92978846	-0.0930701
ENSG000001	RPN1	0.02230045	0.29312398	-0.0930361
ENSG000001	NRBF2	0.22744772	0.59536815	-0.093025
ENSG000002	ALKBH6	0.33523985	0.67708232	-0.0929381
ENSG000001	PIP5K1C	0.18034787	0.54515554	-0.0928877
ENSG000001	SH3KBP1	0.0700254	0.40035295	-0.0928809
ENSG000001	ZNF282	0.23217281	0.59955779	-0.0928755
ENSG000001	EVI5L	0.22184444	0.58912768	-0.0927871
ENSG000002	N/A	0.60200725	0.83845247	-0.0927728
ENSG00000C	TEAD3	0.78527579	0.92061537	-0.0927003
ENSG000001	MYH11	0.83556906	0.94026854	-0.0926991
ENSG000001	CHD4	0.17902406	0.54414332	-0.0925433
ENSG000001	C19orf25	0.08748734	0.42968298	-0.0924746
ENSG00000C	CREBBP	0.28444442	0.64253645	-0.0924122
ENSG000001	PDXK	0.08490846	0.42526981	-0.0924088
ENSG00000C	MRPL28	0.16096206	0.52386068	-0.0923997
ENSG000001	ADGRE2	0.16852585	0.53253292	-0.0923118
ENSG000001	SNX18	0.3274246	0.67217574	-0.0922216
ENSG00000C	EFHC1	0.39324118	0.72063539	-0.0921966
ENSG00000C	STAB1	0.318308	0.66581089	-0.092192
ENSG000001	POLD4	0.13118609	0.4894044	-0.0921812
ENSG000001	CYTH2	0.03700395	0.33343596	-0.0920423
ENSG000001	PHF13	0.17874879	0.54395471	-0.0920309
ENSG000001	AKNA	0.05495205	0.37354464	-0.0919964
ENSG000001	SH3BP5	0.25998286	0.62338681	-0.0919734
ENSG000001	UGGT1	0.09110483	0.43488031	-0.091865
ENSG000001	LRP1B	0.81323037	0.93183372	-0.0918321
ENSG000001	RHOB	0.049348	0.360767	-0.0917466
ENSG000001	UROD	0.18415387	0.54950998	-0.0916685
ENSG000001	F11R	0.17328217	0.53786706	-0.0916361
ENSG000001	PDZD2	0.41037354	0.72965781	-0.0915272
ENSG000001	PLEKHG4	0.42697362	0.7392501	-0.0915119
ENSG000001	TSPAN11	0.81778853	0.9333228	-0.0913498
ENSG00000C	OTUD5	0.09594568	0.44194893	-0.0912686
ENSG000001	PPP3CA	0.2053494	0.56904198	-0.0912093

ENSG000001	PDLIM2	0.04217546	0.34997262	-0.0911746
ENSG000001	OLR1	0.69729612	0.88294449	-0.0910966
ENSG00000(	IFFO1	0.15265459	0.51346327	-0.0910853
ENSG000002	IGFL4	0.79657327	0.92526918	-0.0910647
ENSG000001	CD8B	0.53588585	0.80423066	-0.0910432
ENSG000001	ZMIZ1	0.27527915	0.63499184	-0.0910304
ENSG000001	SPDYE4	0.68092358	0.87677686	-0.0910295
ENSG000001	MEMO1	0.34482151	0.68522517	-0.0909395
ENSG000002	EFNA4	0.22952876	0.59783342	-0.0909378
ENSG00000(	SEMA3C	0.50404831	0.78690354	-0.0909351
ENSG000001	LZTS2	0.27776835	0.6371251	-0.0909276
ENSG000001	PRR12	0.35807931	0.69382497	-0.0909207
ENSG000001	GRB10	0.38039292	0.70987972	-0.0909196
ENSG000001	ADAMTS14	0.82851579	0.93664814	-0.0908433
ENSG000001	TMEM115	0.17440044	0.53940156	-0.0908244
ENSG000001	EMILIN1	0.41540713	0.73221244	-0.0908155
ENSG000001	SMYD3	0.39864648	0.72459676	-0.0907619
ENSG000002	SIGLEC14	0.5125339	0.79227085	-0.0907548
ENSG000002	MCRIP1	0.21284532	0.5782114	-0.0907048
ENSG000002	ZNF625	0.77598116	0.91686844	-0.0906857
ENSG000001	CDAN1	0.26174678	0.62545731	-0.0906778
ENSG00000(	TDRD1	0.81965179	0.93468999	-0.0906545
ENSG000001	CCDC189	0.64378864	0.85940463	-0.0906228
ENSG000001	RPL32	0.49702378	0.78237677	-0.0906147
ENSG00000(	RNF126	0.22366999	0.59091342	-0.0905921
ENSG000001	SLX1A	0.7808473	0.9189743	-0.0905308
ENSG000001	TMEM151A	0.76562311	0.9126184	-0.0904694
ENSG000001	ANKH	0.25712306	0.62086214	-0.0904514
ENSG000001	STARD3	0.02774486	0.3113392	-0.0904073
ENSG000001	XXYLT1	0.16979701	0.53437545	-0.0904037
ENSG000001	ATG9B	0.48157969	0.77466133	-0.0903867
ENSG000001	PCBP2	0.02830892	0.31303636	-0.0903319
ENSG00000(	DBNDD1	0.53299567	0.80294166	-0.0902827
ENSG00000(	IRAK3	0.40381066	0.72672584	-0.0901513
ENSG000001	RASSF3	0.15270371	0.51346327	-0.090138
ENSG000001	SLC25A23	0.42029379	0.73468557	-0.0901251
ENSG000001	KYAT1	0.40433226	0.72672584	-0.0900488
ENSG000001	HNRNPUL1	0.04752952	0.35865951	-0.0900423
ENSG00000(	PVR	0.28438061	0.64253645	-0.090021
ENSG000001	SGPL1	0.07483281	0.40801218	-0.0900136
ENSG000001	UBA1	0.18550955	0.55033096	-0.0899985
ENSG000001	CEP63	0.10086393	0.44917064	-0.0899605
ENSG000001	ALDH16A1	0.28260961	0.64061598	-0.0899557

ENSG000001	IQGAP1	0.31282882	0.66112215	-0.0899469
ENSG000001	F13A1	0.38014767	0.70987972	-0.0898891
ENSG000001	DHRS12	0.39581794	0.72222558	-0.0898504
ENSG000001	PROSER3	0.18034917	0.54515554	-0.0898094
ENSG000001	MOB2	0.19565063	0.56325872	-0.0897456
ENSG000001	FAM174B	0.36082944	0.69535997	-0.0897062
ENSG000001	XRCC3	0.1416982	0.50055578	-0.0896824
ENSG000001	WNT10B	0.61761244	0.8465451	-0.0896727
ENSG000002	NEFL	0.5352764	0.80413676	-0.0896015
ENSG000002	PRAF2	0.18053923	0.54527819	-0.0895077
ENSG00000C	AURKA	0.47040998	0.76633358	-0.0894613
ENSG00000C	WIZ	0.13918623	0.49975817	-0.0894514
ENSG000001	PEX6	0.21367861	0.57911862	-0.0894452
ENSG000001	POU6F1	0.45588307	0.75632853	-0.0894199
ENSG00000C	BORCS8-MEI	0.68411332	0.87891236	-0.0893238
ENSG000001	RAC1	0.07471502	0.40750684	-0.0893177
ENSG000001	CHPT1	0.31437946	0.66265276	-0.0893038
ENSG000001	MAN2B1	0.11608273	0.47160917	-0.0892958
ENSG000001	RORC	0.46612698	0.76484555	-0.0892678
ENSG000001	PRRG2	0.76547847	0.91260342	-0.0892638
ENSG000001	ZFP36L1	0.18016041	0.54501079	-0.0892173
ENSG000001	UBOX5	0.09103576	0.43488031	-0.0891942
ENSG000001	CCNE1	0.53949938	0.80691336	-0.0891291
ENSG000002	ARPC4	0.05853788	0.3800398	-0.0891222
ENSG000001	PPT1	0.09069853	0.43454471	-0.0891166
ENSG000001	FBN2	0.30640383	0.65735608	-0.08911
ENSG000001	HDLBP	0.07644563	0.41092575	-0.0890757
ENSG000001	TMEM8B	0.46513683	0.76426277	-0.0890406
ENSG000001	NHLH1	0.42115291	0.73521014	-0.0890228
ENSG00000C	TCF7	0.44466018	0.74940749	-0.0889983
ENSG000001	HSD17B12	0.29104559	0.64869008	-0.0889917
ENSG000001	CC2D1A	0.21684923	0.58352353	-0.0889829
ENSG000001	SETD1B	0.14116784	0.50055066	-0.0889452
ENSG000001	FBXO46	0.1098276	0.46255597	-0.0889386
ENSG000001	METTL21C	0.82306415	0.93539963	-0.0889338
ENSG000001	SLC2A9	0.17153492	0.53577669	-0.0889291
ENSG000001	NLGN1	0.85277598	0.94735383	-0.0889252
ENSG000001	B4GALT3	0.1653006	0.52878404	-0.0888745
ENSG00000C	CYBA	0.19670437	0.56409142	-0.0888577
ENSG000002	CPNE1	0.05144689	0.36502002	-0.0888436
ENSG000001	E2F3	0.39540073	0.72209218	-0.0888285
ENSG00000C	IGF1	0.85627016	0.9490718	-0.0888245
ENSG000001	TM6SF1	0.40650221	0.7275473	-0.0887977

ENSG000001	TEDC1	0.30448754	0.65635638	-0.0887544
ENSG000001	C11orf1	0.61572803	0.84553248	-0.08868
ENSG000000	ADGRA2	0.40209686	0.72669629	-0.088642
ENSG000000	SELENOO	0.22844386	0.59653313	-0.0886164
ENSG000000	ZNF839	0.20449791	0.5688754	-0.0885963
ENSG000001	AKR1E2	0.64656232	0.86080839	-0.0885851
ENSG000001	ZNF444	0.2708193	0.63206742	-0.0885719
ENSG000001	SLC26A8	0.57532046	0.82344878	-0.0885103
ENSG000001	MAP4K1	0.35512408	0.6919142	-0.0884841
ENSG000001	ZNF335	0.13020093	0.48823847	-0.0884458
ENSG000001	CFAP410	0.33175676	0.67477571	-0.0884184
ENSG000001	PALLD	0.55046342	0.81253404	-0.0882725
ENSG000001	HOMER2	0.43903625	0.74609757	-0.0882209
ENSG000001	MMACHC	0.32060408	0.66739083	-0.0882083
ENSG000001	RSPH10B2	0.85219441	0.94731122	-0.0881937
ENSG000001	RPS15	0.3313536	0.67444298	-0.0881727
ENSG000001	SMAD9	0.5952681	0.83552629	-0.0881591
ENSG000000	MYL6	0.30402319	0.65635638	-0.0881528
ENSG000001	BABAM1	0.23615244	0.60336718	-0.0881512
ENSG000001	TCEA2	0.35627289	0.69245905	-0.0881463
ENSG000001	PABPC3	0.39753378	0.72378963	-0.0880362
ENSG000000	USE1	0.3887939	0.71777675	-0.0878519
ENSG000001	RGS6	0.46334743	0.76294212	-0.0878276
ENSG000001	NUDT5	0.21092472	0.57523547	-0.0877502
ENSG000001	TCF25	0.11749992	0.4740063	-0.087746
ENSG000001	SWI5	0.32254325	0.66849561	-0.0877282
ENSG000000	CHERP	0.23746143	0.60402516	-0.0876994
ENSG000002	GSTM2	0.51954294	0.79650824	-0.0876609
ENSG000001	SLC37A3	0.41950406	0.73452883	-0.0876584
ENSG000002	CDK11B	0.03917499	0.33942602	-0.0876366
ENSG000000	HOOK2	0.25600742	0.62056088	-0.0876158
ENSG000001	SLC35E1	0.07770316	0.41335649	-0.0876127
ENSG000001	WBP1L	0.01329714	0.25828987	-0.0875975
ENSG000002	C1orf226	0.77752607	0.91769214	-0.0875192
ENSG000001	CHAF1B	0.45288373	0.75444669	-0.0874324
ENSG000001	KLHL25	0.42793636	0.73974265	-0.087421
ENSG000001	LAMTOR1	0.03751113	0.33471496	-0.0874189
ENSG000001	SRRD	0.27680167	0.63632345	-0.0874048
ENSG000001	CREB5	0.40411406	0.72672584	-0.0873814
ENSG000001	TMEM169	0.38395503	0.7123445	-0.0873616
ENSG000001	VAPA	0.12433346	0.48169947	-0.0873566
ENSG000001	FBXL15	0.41547555	0.73221244	-0.087345
ENSG000000	SREBF1	0.22290282	0.5903895	-0.0873057

ENSG000001	DNAJC3	0.22782282	0.5959657	-0.0871919
ENSG000001	COLGALT1	0.15154185	0.51262188	-0.0871737
ENSG000001	KIAA1841	0.4190093	0.73426975	-0.0871493
ENSG000001	TRIM28	0.18368346	0.54871392	-0.087149
ENSG000001	AP3S2	0.15056292	0.51090703	-0.0870716
ENSG000001	CEACAM5	0.82216098	0.93539963	-0.0870714
ENSG000001	ZHX3	0.38343154	0.71225009	-0.0870697
ENSG000001	POLR2F	0.25604166	0.62056088	-0.0870671
ENSG000001	RPL37A	0.48991326	0.77995113	-0.0870666
ENSG000001	PLEC	0.28290996	0.64084947	-0.0870644
ENSG000001	H1-4	0.83861832	0.94173685	-0.0870581
ENSG000001	CXCR3	0.49938657	0.78410505	-0.0870321
ENSG000001	CYRIA	0.08520097	0.42526981	-0.0870135
ENSG000001	PLD6	0.43184617	0.74137094	-0.0870042
ENSG000001	TRIP10	0.42605233	0.73856395	-0.0869185
ENSG000001	C20orf27	0.31562285	0.66324235	-0.0868677
ENSG000001	DNASE2	0.15905389	0.5217239	-0.0867893
ENSG000002	POM121C	0.18803858	0.55211256	-0.0866358
ENSG000001	COTL1	0.10545889	0.45589352	-0.0866313
ENSG000001	GABARAP	0.60976301	0.84290158	-0.0866279
ENSG000001	TRMU	0.26614068	0.62892245	-0.0866207
ENSG000001	WDR88	0.5434188	0.8084284	-0.0866065
ENSG000001	FBXO15	0.77944757	0.91827559	-0.0865801
ENSG000001	CSNK1D	0.0334856	0.32398208	-0.0865496
ENSG000001	WIPF2	0.06865184	0.39796501	-0.0864679
ENSG000001	USP4	0.0054337	0.21833269	-0.0864421
ENSG00000(	ELOVL1	0.03027709	0.31901949	-0.0864305
ENSG00000(	FLYWCH1	0.41590604	0.73235476	-0.0864092
ENSG000001	ZDHHC7	0.05014794	0.36227834	-0.0863811
ENSG00000(	SLC8B1	0.13103386	0.4894044	-0.0863716
ENSG000001	DNAJC30	0.34292073	0.68359637	-0.0863669
ENSG000001	SYNGR1	0.4421338	0.74830001	-0.0863385
ENSG000001	KIAA0753	0.32859307	0.67264721	-0.0862539
ENSG000001	MAP3K12	0.30580418	0.65705827	-0.0862041
ENSG000002	GSTA1	0.82352637	0.93558785	-0.086108
ENSG000001	RHBDL1	0.53480464	0.80406944	-0.0860785
ENSG000001	MFAP3L	0.49644673	0.78193527	-0.0860609
ENSG000002	MYMX	0.8489414	0.94594163	-0.0860388
ENSG000001	NOSIP	0.29498589	0.65121791	-0.0860307
ENSG000001	OLFM1	0.55122117	0.81320898	-0.0859992
ENSG000001	ARHGEF2	0.0298128	0.31849971	-0.0859936
ENSG00000(	CYP24A1	0.74805209	0.90731063	-0.0859576
ENSG000001	SUPT6H	0.09897029	0.4460792	-0.085928

ENSG000001	HPDL	0.7956049	0.9246397	-0.0859126
ENSG000001	NDUFA1	0.48732015	0.77793335	-0.0858885
ENSG000002	BRD3OS	0.42006188	0.73468557	-0.0858509
ENSG000001	HMGA1	0.33522304	0.67708232	-0.0857879
ENSG000001	DPP4	0.42137588	0.73536974	-0.0857657
ENSG000002	PPM1N	0.49114882	0.78023514	-0.0857601
ENSG00000C	AMPH	0.54035197	0.80714764	-0.0857471
ENSG00000C	CDHR5	0.85501979	0.94841893	-0.0856245
ENSG00000C	GRHL2	0.85389225	0.94801844	-0.0855998
ENSG000001	VPS8	0.06466214	0.39055819	-0.0855977
ENSG000001	VSIG1	0.53005455	0.8016768	-0.0855182
ENSG000001	POU6F2	0.75309262	0.90914237	-0.0855048
ENSG000001	CHD3	0.31880263	0.66618207	-0.0854805
ENSG000001	CDK5	0.33476903	0.67675278	-0.0854394
ENSG000001	TMEM222	0.10468513	0.45514301	-0.0854212
ENSG000001	POLR2A	0.35275291	0.69109369	-0.0853619
ENSG000001	AMOT	0.51607686	0.7950982	-0.085245
ENSG000001	EHBP1L1	0.1668613	0.53125145	-0.0852103
ENSG00000C	TSPAN32	0.31562934	0.66324235	-0.0851998
ENSG000001	STK16	0.12789752	0.48588488	-0.0851872
ENSG000001	RAC3	0.76268397	0.91167139	-0.0851792
ENSG000001	IQSEC1	0.20497189	0.56891466	-0.0851477
ENSG000001	FBLIM1	0.88667709	0.96095764	-0.0850948
ENSG000001	NUDT7	0.56245661	0.81818741	-0.0850514
ENSG000001	CYB5D2	0.35104589	0.69048239	-0.0850483
ENSG000001	LRRC37A3	0.43741082	0.74517254	-0.0850452
ENSG00000C	RPL18	0.39687241	0.72308765	-0.0850152
ENSG000002	XKR5	0.77110902	0.91463929	-0.0850023
ENSG000001	RPS4X	0.44102705	0.74750808	-0.0849856
ENSG000002	INSL3	0.40731269	0.72768322	-0.0849666
ENSG000002	CRLF2	0.81600019	0.93251163	-0.0849449
ENSG000001	FSD2	0.81507884	0.93228896	-0.084943
ENSG000001	LGI2	0.63974512	0.85691647	-0.0849348
ENSG000001	CHD7	0.14987638	0.50942953	-0.0849284
ENSG000001	RTF1	0.0408385	0.34551949	-0.0848971
ENSG000001	KCTD21	0.08144355	0.41887836	-0.0848646
ENSG000001	CENPT	0.36066163	0.69535997	-0.0848234
ENSG00000C	DGKA	0.37912821	0.70965485	-0.0847993
ENSG000001	SIT1	0.45573851	0.75632853	-0.0847813
ENSG000002	MUC19	0.8462769	0.94485306	-0.0847287
ENSG000001	CMTM4	0.48561403	0.7774422	-0.0846808
ENSG000001	EGLN3	0.36178067	0.69588937	-0.0846684
ENSG000001	S100B	0.69594981	0.88245905	-0.0846647

ENSG000001	ZNF574	0.26340418	0.62687307	-0.0845853
ENSG00000(	UTS2	0.76014868	0.91136366	-0.0845847
ENSG000001	CACNA2D3	0.50909646	0.79132674	-0.0845547
ENSG000001	CEACAM4	0.6142335	0.8451435	-0.0845194
ENSG000001	TIRAP	0.10639302	0.45652841	-0.0844882
ENSG000001	PSRC1	0.25084938	0.61671278	-0.0844704
ENSG000001	SERTAD2	0.38747036	0.71667898	-0.0844423
ENSG000001	CASP2	0.20023119	0.56699578	-0.0844289
ENSG000001	ANP32A	0.07160352	0.4024868	-0.0843918
ENSG000001	SLC22A18	0.32665516	0.67197514	-0.0843513
ENSG000001	GOLGA2	0.08508548	0.42526981	-0.0842725
ENSG000001	UBE3D	0.59303709	0.83444495	-0.0842158
ENSG000001	TCEAL2	0.81340015	0.93183372	-0.084207
ENSG000001	CXCL5	0.55746184	0.81587881	-0.0841953
ENSG000001	ZMAT5	0.07077831	0.40156223	-0.0841859
ENSG000001	USB1	0.14574037	0.50430399	-0.0841737
ENSG00000(	P3H2	0.56642535	0.8197023	-0.0841119
ENSG000001	ACSS2	0.14025006	0.5002427	-0.0841094
ENSG000001	CCNY	0.2151409	0.58090552	-0.0840585
ENSG000002	AP5Z1	0.1297131	0.48742228	-0.0839967
ENSG000001	NCKAP5L	0.23210172	0.59952843	-0.0839121
ENSG000001	RCOR2	0.85685428	0.94924076	-0.0839015
ENSG000001	SPIC	0.76549018	0.91260342	-0.0838252
ENSG000001	FAM9B	0.86937301	0.9539778	-0.0837857
ENSG00000(	ANO8	0.41471212	0.73184916	-0.0837746
ENSG000001	CALM3	0.11898949	0.47567495	-0.083728
ENSG00000(	FAM3A	0.33055715	0.67415584	-0.0836729
ENSG000001	RLBP1	0.84152737	0.94283868	-0.08367
ENSG00000(	SLC66A1	0.2211245	0.58858992	-0.0836334
ENSG000001	SERPINF1	0.41863019	0.73381176	-0.0835912
ENSG000001	PIM3	0.25949613	0.6232076	-0.0835733
ENSG000001	TSSC4	0.17184473	0.53586381	-0.0834911
ENSG000002	N/A	0.28907149	0.6469423	-0.0834885
ENSG000001	PKNOX1	0.21972624	0.58681553	-0.0834821
ENSG000001	KCNJ14	0.64728275	0.86129024	-0.0834507
ENSG000001	ARMC5	0.35974028	0.69484359	-0.0834275
ENSG000001	C11orf45	0.71913062	0.89386235	-0.0832674
ENSG000001	CXCR6	0.64043229	0.85706063	-0.0832417
ENSG000001	RAB33A	0.49067018	0.7800506	-0.083187
ENSG000001	USP9Y	0.5683431	0.82037292	-0.0831869
ENSG000001	ZGPAT	0.21939251	0.58667264	-0.0831777
ENSG000001	SRRM2	0.05651134	0.3751327	-0.0830567
ENSG000001	NACC1	0.1638084	0.52709285	-0.083045

ENSG000001	TSHB	0.87192186	0.95457921	-0.0829883
ENSG000001	CMTM7	0.15369689	0.51487033	-0.0829782
ENSG000001	MEAK7	0.2243273	0.59169817	-0.0829621
ENSG000002	NEURL4	0.25087072	0.61671278	-0.0828646
ENSG000001	PACS2	0.33727557	0.67926926	-0.0827749
ENSG000002	MUC12	0.78696658	0.92133364	-0.0827572
ENSG000001	MYPOP	0.2327683	0.60019671	-0.0827098
ENSG00000C	RIPOR3	0.4037591	0.72672584	-0.0826207
ENSG000001	ACMSD	0.72319132	0.89582373	-0.0825993
ENSG00000C	TMEM38A	0.46798793	0.76574496	-0.0825935
ENSG000001	OCM	0.5940152	0.83456808	-0.0825741
ENSG000001	TXNRD1	0.16353243	0.52709285	-0.08256
ENSG000001	ARTN	0.83442987	0.93963783	-0.0825561
ENSG000001	TLR2	0.21311823	0.57868334	-0.0825384
ENSG00000C	SIRT2	0.18455531	0.5498217	-0.0825372
ENSG000001	NUP214	0.02412733	0.29927225	-0.082521
ENSG000001	RHOT2	0.2476933	0.61375236	-0.0825171
ENSG000001	VCP	0.12770846	0.48588488	-0.0824018
ENSG000001	NUDT16L1	0.34491598	0.68522517	-0.0823543
ENSG000001	KNG1	0.85232923	0.94731122	-0.0823317
ENSG000001	CHMP4B	0.08589335	0.42662905	-0.0823254
ENSG00000C	NUBP2	0.32482894	0.67042678	-0.0822801
ENSG000001	MAL2	0.85930667	0.95038229	-0.0822767
ENSG000001	PPIF	0.09231206	0.43743098	-0.0822286
ENSG000001	B3GAT3	0.31029025	0.65949478	-0.0821762
ENSG000001	SERPINA1	0.33577725	0.67743727	-0.0821391
ENSG000001	MEGF11	0.67098933	0.87218472	-0.0821367
ENSG000001	RIC8A	0.04920392	0.360767	-0.082111
ENSG000001	CILP	0.67028887	0.87207375	-0.0821001
ENSG000001	LRRC19	0.75970915	0.91136366	-0.0819957
ENSG000001	VPS26B	0.16095579	0.52386068	-0.0819812
ENSG000001	DRC1	0.78151162	0.91907937	-0.0819636
ENSG000002	P2RY11	0.45377418	0.75506573	-0.0819335
ENSG000001	TPM1	0.3077528	0.65762361	-0.081895
ENSG000001	POLR2I	0.44746526	0.75099423	-0.0818748
ENSG00000C	GAS7	0.20327713	0.56867862	-0.08185
ENSG000001	RYR1	0.39832531	0.72429687	-0.0818172
ENSG000001	TUB	0.71449514	0.89175724	-0.0817656
ENSG000001	LCN12	0.68359949	0.87849752	-0.0817527
ENSG00000C	CNGB1	0.81553045	0.9323219	-0.0816967
ENSG000001	GNB1L	0.49505104	0.78124152	-0.0816872
ENSG000001	MAD2L1	0.59885527	0.83673325	-0.0816389
ENSG000001	DOK1	0.149541	0.50925123	-0.0816164

ENSG000001	MPZ	0.30480115	0.65635638	-0.0815912
ENSG000001	ZNF281	0.19882431	0.56598606	-0.0815601
ENSG000001	THOC5	0.06291253	0.38840714	-0.0815222
ENSG00000(	PRTFDC1	0.6955944	0.88231682	-0.0815217
ENSG000001	CD5	0.46008286	0.75987848	-0.0814989
ENSG00000(	VCL	0.26879673	0.63079288	-0.0814728
ENSG000001	PNPLA7	0.52524519	0.79936321	-0.0814564
ENSG000002	N/A	0.86660658	0.95264796	-0.0814043
ENSG000001	FGD5	0.80159523	0.92746876	-0.0813615
ENSG000001	MCAT	0.38415436	0.71245167	-0.0813332
ENSG000001	SHISA2	0.82006991	0.93468999	-0.081332
ENSG000001	SNX27	0.11003469	0.46261866	-0.0812418
ENSG000001	KDF1	0.71806109	0.89332947	-0.0812363
ENSG000001	SHCBP1	0.56907846	0.82037292	-0.0812038
ENSG000001	ZNF667	0.69035348	0.88074719	-0.0811619
ENSG000001	PFKFB3	0.39148109	0.71964297	-0.0810926
ENSG00000(	TNIP3	0.6774302	0.87510133	-0.0810837
ENSG000001	P3H4	0.59120744	0.83299508	-0.0810833
ENSG000001	PIWIL2	0.71822939	0.89332947	-0.0810804
ENSG000001	SEPTIN10	0.46213814	0.76172337	-0.0810493
ENSG000001	AK1	0.44840049	0.75136004	-0.080998
ENSG000001	ACTG1	0.09353096	0.4384324	-0.0809612
ENSG000001	LOXHD1	0.33302511	0.67532519	-0.0808736
ENSG000001	ZBTB7B	0.09892814	0.4460792	-0.0808681
ENSG000001	ZDHHC3	0.04141236	0.34750002	-0.0808537
ENSG000001	SUV39H1	0.27807838	0.63759905	-0.0808535
ENSG000001	STIM1	0.08702244	0.42907415	-0.0808528
ENSG000001	PLCL1	0.59283076	0.83431304	-0.0808423
ENSG000001	TMEM86A	0.47039261	0.76633358	-0.0807697
ENSG000001	LRRC6	0.501854	0.78548326	-0.0807119
ENSG00000(	ATP9A	0.48198922	0.77486788	-0.0806795
ENSG00000(	PNKP	0.20203567	0.56775306	-0.0806559
ENSG000001	MROH1	0.29380918	0.65039296	-0.080648
ENSG000001	KCNJ16	0.88671074	0.96095764	-0.0806164
ENSG000001	FLNA	0.33912576	0.68041384	-0.0805807
ENSG000001	FCHSD1	0.15618376	0.51865126	-0.0804729
ENSG000001	HID1	0.51038241	0.79143652	-0.0804697
ENSG00000(	MKNK1	0.10649243	0.4566397	-0.0804217
ENSG000001	ZDHHC12	0.23579594	0.60330268	-0.0803942
ENSG000001	CCDC102A	0.48816335	0.77853927	-0.0803287
ENSG000001	TUBGCP2	0.13946421	0.49975817	-0.0803038
ENSG000001	CDC42EP3	0.16836763	0.53253292	-0.0802862
ENSG000001	APBB2	0.59409659	0.83456808	-0.0802829

ENSG000001	NDUFB10	0.27074734	0.6319903	-0.0802752
ENSG000002	C4orf48	0.47587484	0.77036688	-0.0802727
ENSG000001	DMPK	0.52374782	0.79870477	-0.0802389
ENSG000001	HACD1	0.77854997	0.91810931	-0.0801476
ENSG000001	COL6A1	0.55389182	0.81396882	-0.0801219
ENSG000001	GBE1	0.14206387	0.50068215	-0.0801061
ENSG000001	E4F1	0.30134995	0.65546137	-0.0800814
ENSG000001	TTC8	0.52800142	0.80062313	-0.0800625
ENSG000001	POLR2J3	0.26438034	0.6274921	-0.0800609
ENSG000001	GET1	0.4850323	0.77694975	-0.080007
ENSG000001	RECQL5	0.18260352	0.54695002	-0.0798599
ENSG000001	LBR	0.41856233	0.73381176	-0.0797951
ENSG000001	PDZD8	0.47963657	0.77360276	-0.0797609
ENSG000001	H2AZ1	0.29402285	0.65044158	-0.0796919
ENSG000001	ITPRIP	0.37232131	0.70436683	-0.0795984
ENSG000001	PLLP	0.50365044	0.78673932	-0.0795212
ENSG000001	GSN	0.47844213	0.77232426	-0.0794567
ENSG000001	DAGLB	0.19089507	0.55647457	-0.0794549
ENSG000002	H4C9	0.3190215	0.66631747	-0.0794379
ENSG000001	FBXL18	0.26286286	0.6264304	-0.0794283
ENSG000001	RBM42	0.29141561	0.64906931	-0.0794274
ENSG000001	OR5B12	0.80745784	0.92973209	-0.0794046
ENSG000001	CATSPER1	0.56629974	0.8197023	-0.0793448
ENSG000001	MEIOC	0.78322492	0.91983554	-0.0792627
ENSG000001	C1QTNF6	0.41378392	0.73141372	-0.0792358
ENSG000001	RPLP1	0.46457544	0.76392983	-0.0792184
ENSG000001	THAP8	0.3657894	0.69837111	-0.079165
ENSG00000C	RFXANK	0.22189144	0.58912768	-0.0791514
ENSG000001	MUC5B	0.79223146	0.92314956	-0.0790895
ENSG000001	KCTD2	0.06061272	0.38444089	-0.0790623
ENSG000001	ATP2B2	0.86808852	0.95338532	-0.0790104
ENSG000002	LIME1	0.41146125	0.72999304	-0.078986
ENSG000001	SLIT1	0.36915643	0.70197716	-0.0789643
ENSG000001	MAK	0.40681173	0.7275473	-0.0789266
ENSG000001	ZNF668	0.32023362	0.66722134	-0.0789259
ENSG000001	C1orf194	0.78681904	0.92122734	-0.0789207
ENSG000001	MZT2A	0.48050255	0.77383118	-0.078835
ENSG000001	TSEN54	0.47542212	0.77011325	-0.0788046
ENSG000001	IMPDH2	0.42214468	0.73607851	-0.0787782
ENSG000002	OXLD1	0.40473212	0.72672584	-0.0787251
ENSG000001	POC1A	0.41741109	0.73321581	-0.0786022
ENSG000002	SULT1A3	0.47144325	0.76694135	-0.0785947
ENSG000001	THOP1	0.39362736	0.72076675	-0.0785724

ENSG000001	ATP6V1B2	0.18268205	0.54696818	-0.0785698
ENSG000001	PPP6R2	0.1195619	0.47604098	-0.0785342
ENSG00000C	MZF1	0.20860518	0.57234241	-0.0784278
ENSG000001	SLC22A4	0.36633523	0.69863579	-0.078402
ENSG000001	PTGFR	0.85336635	0.94777027	-0.0783976
ENSG000001	BRI3BP	0.44854413	0.75152317	-0.0783802
ENSG000001	TJP3	0.53282441	0.80291422	-0.0783247
ENSG000001	WASF1	0.5077771	0.79065693	-0.0783203
ENSG000001	PLCD4	0.2322334	0.59956425	-0.078283
ENSG000001	GUCA1B	0.45507644	0.7559601	-0.078283
ENSG00000C	TFPI	0.54207221	0.80808176	-0.0782439
ENSG000001	SLC39A5	0.83014784	0.93721091	-0.0782435
ENSG000001	GATD1	0.30450392	0.65635638	-0.0782146
ENSG00000C	TNFRSF9	0.49249896	0.7804495	-0.0782035
ENSG000001	VRK3	0.02545738	0.30326594	-0.0781388
ENSG000001	RNF24	0.3455532	0.68530904	-0.0780998
ENSG00000C	ARAF	0.10594195	0.4561473	-0.0780129
ENSG000001	SH3GLB2	0.25014776	0.61596135	-0.0779791
ENSG00000C	GNPTG	0.23394525	0.60173104	-0.0779407
ENSG000001	ASTN2	0.76811474	0.91357827	-0.0779314
ENSG000002	ATP6V1E2	0.32422101	0.66992678	-0.0779072
ENSG000001	LYG1	0.42697448	0.7392501	-0.0778533
ENSG000001	ZFYVE21	0.21002118	0.57424655	-0.077851
ENSG00000C	GBA2	0.03776044	0.33525901	-0.0778319
ENSG000001	COX5B	0.19431987	0.56191549	-0.0777524
ENSG000001	TP53I3	0.30939266	0.65916845	-0.0777348
ENSG000001	SPDEF	0.85074417	0.94684256	-0.0777227
ENSG00000C	TNC	0.79790121	0.92598971	-0.077635
ENSG00000C	HMG20B	0.22942068	0.59783342	-0.0776329
ENSG000001	GHRL	0.32844199	0.67259327	-0.0776282
ENSG00000C	DVL2	0.26687733	0.62929976	-0.0776142
ENSG000001	STXBP1	0.59369733	0.83456808	-0.077586
ENSG000001	RNF144B	0.28896354	0.64680191	-0.0775483
ENSG000001	CRYAB	0.76636414	0.91314702	-0.0774223
ENSG000001	FSCN2	0.77733606	0.91753458	-0.0774123
ENSG000001	PHLDA1	0.57986092	0.82610777	-0.0774013
ENSG000001	CHIC2	0.03226977	0.32360639	-0.0774012
ENSG000001	TSHZ3	0.37817432	0.70909881	-0.0773699
ENSG000001	PKN1	0.20752644	0.57126603	-0.0773624
ENSG000001	CCDC106	0.44783993	0.7510408	-0.0773327
ENSG000001	SPAG8	0.64042812	0.85706063	-0.0772839
ENSG000001	NTMT1	0.34248829	0.68338046	-0.0772813
ENSG000001	RASAL3	0.3073178	0.65762361	-0.0772757

ENSG000001	P3H1	0.31612318	0.66372668	-0.0772749
ENSG000001	DNAI2	0.65253329	0.86379349	-0.0772731
ENSG000001	INTS11	0.20691786	0.57041176	-0.077244
ENSG000001	OPTN	0.36239395	0.69636577	-0.0771536
ENSG000001	KDM3B	0.09639882	0.44215472	-0.0770926
ENSG00000C	ACAA1	0.13085785	0.48934567	-0.0770574
ENSG000001	MARCKSL1	0.35610395	0.69237934	-0.0770476
ENSG000001	VGF	0.8709673	0.95437279	-0.0770451
ENSG000001	PANK4	0.87436994	0.95579655	-0.0770192
ENSG00000C	ANLN	0.67914997	0.87595187	-0.077004
ENSG000001	CCDC54	0.82747638	0.93660278	-0.0769577
ENSG000001	JMJD8	0.2592458	0.62303431	-0.076918
ENSG000002	LIX1L	0.22819818	0.59645659	-0.0769127
ENSG000002	CDRT1	0.85217677	0.94731122	-0.0767947
ENSG000001	PDZD7	0.5633434	0.81863326	-0.0767417
ENSG000001	RPS23	0.56349991	0.81863326	-0.076704
ENSG000001	PPP6R1	0.20778777	0.57145912	-0.0766976
ENSG000001	FBXO32	0.4971921	0.78256186	-0.0766754
ENSG000001	OSTF1	0.0681303	0.39743754	-0.07663
ENSG000002	SCAMP4	0.2389282	0.60520581	-0.0766233
ENSG000002	SDHAF1	0.41774962	0.73327026	-0.076597
ENSG00000C	BRPF3	0.23662778	0.60364584	-0.0765522
ENSG000001	SLC37A4	0.37184656	0.70401966	-0.0764939
ENSG000001	VSIG4	0.58154154	0.82722887	-0.0764852
ENSG000001	WNT5B	0.71395001	0.89153258	-0.0764498
ENSG000001	GPR1	0.88422921	0.9606711	-0.0764326
ENSG000001	RIN1	0.36302216	0.69665415	-0.0764245
ENSG00000C	WDR1	0.07299009	0.40435477	-0.0764064
ENSG000001	ZFPM1	0.45087445	0.75348767	-0.0764012
ENSG00000C	PPP1R13B	0.41624932	0.73241014	-0.0763871
ENSG000001	ACTG2	0.87909563	0.95817324	-0.0763578
ENSG00000C	PHKA2	0.05106579	0.36427913	-0.076353
ENSG000001	RNF40	0.24602481	0.61202771	-0.0763481
ENSG000001	AFDN	0.49815444	0.78286233	-0.0763353
ENSG000001	FIZ1	0.26707224	0.62929976	-0.0763297
ENSG000001	DRC3	0.45770383	0.75749027	-0.0763149
ENSG000001	ZNF233	0.4936643	0.78107975	-0.0762859
ENSG000001	SERPINE1	0.61790933	0.84662697	-0.0762796
ENSG000001	UBTF	0.22956774	0.59783342	-0.0762201
ENSG000001	CARMIL2	0.47040649	0.76633358	-0.0762135
ENSG000001	KCNA5	0.80043118	0.92689709	-0.0759839
ENSG000001	CLIP2	0.29407082	0.65044158	-0.0759699
ENSG000001	NME3	0.42419974	0.73732623	-0.0758977

ENSG000001	FBXO40	0.67909324	0.87594827	-0.0758955
ENSG00000C	SLC4A11	0.80926286	0.93027167	-0.0758739
ENSG000001	ATP5IF1	0.42436337	0.7374131	-0.075862
ENSG000002	U2AF1L5	0.52433443	0.79878989	-0.0758288
ENSG000001	SMARCD2	0.11105624	0.46430702	-0.0758186
ENSG000001	RNF152	0.85221862	0.94731122	-0.0758161
ENSG000002	ATF7-NPFF	0.53114934	0.80232066	-0.0757666
ENSG000001	SLC4A3	0.61613556	0.84569598	-0.0757266
ENSG000001	GPSM2	0.40496426	0.72672584	-0.0757018
ENSG000002	TMEM250	0.19924385	0.56600138	-0.0756888
ENSG000001	PRUNE1	0.15701407	0.5199629	-0.0756586
ENSG000001	C1orf115	0.45741506	0.75742878	-0.0755834
ENSG00000C	ZC3H3	0.39014726	0.71927554	-0.0755621
ENSG000001	ZNF581	0.22685293	0.59438615	-0.0755277
ENSG000001	BTG1	0.23075815	0.59855451	-0.0755166
ENSG000001	CFL1	0.17647757	0.54126676	-0.075515
ENSG000001	IQCN	0.17529	0.54013466	-0.0755025
ENSG000001	HIC2	0.39294898	0.72063539	-0.0755004
ENSG000002	N/A	0.88635639	0.96095764	-0.0754981
ENSG00000C	ZXDC	0.26461201	0.6274921	-0.0754914
ENSG00000C	CD4	0.28708657	0.64446628	-0.0754914
ENSG000001	CD8A	0.57201964	0.82171839	-0.0754791
ENSG000001	H2AX	0.45627224	0.75682857	-0.0754763
ENSG000001	ESS2	0.11775956	0.47445705	-0.0754299
ENSG000001	LSR	0.53851012	0.80633537	-0.0753498
ENSG000001	DSTYK	0.26936358	0.63100512	-0.0753376
ENSG00000C	TMEM161A	0.46949011	0.7662622	-0.0753026
ENSG000001	RYBP	0.25045441	0.61634256	-0.0752823
ENSG00000C	FLT4	0.61096063	0.84334674	-0.0752692
ENSG000001	ENG	0.41302555	0.73077485	-0.0751911
ENSG000001	TPRA1	0.36368983	0.69694698	-0.0751845
ENSG000002	NME1	0.7266394	0.89706038	-0.0751804
ENSG000001	FAAH	0.41180048	0.73035548	-0.075154
ENSG000002	PLA2G4B	0.68985983	0.88074719	-0.0751435
ENSG000002	NME2	0.65147154	0.8634351	-0.0751424
ENSG00000C	RAB7A	0.07885413	0.41446832	-0.0751316
ENSG000002	CUX1	0.0469107	0.35811287	-0.0751248
ENSG000001	MAPK1	0.31076754	0.65949478	-0.075097
ENSG00000C	PAX6	0.85015096	0.94644505	-0.0750317
ENSG000001	ATXN1	0.30213207	0.65591444	-0.0749828
ENSG000001	ZNF598	0.3293017	0.67307046	-0.0746628
ENSG000001	PCP2	0.4861755	0.77752459	-0.074632
ENSG000001	CHST7	0.30416278	0.65635638	-0.0746287

ENSG000001	IRF2BP1	0.37266048	0.70465683	-0.0745806
ENSG000001	TDP2	0.25377193	0.61921212	-0.074561
ENSG000001	CNTROB	0.26756781	0.62982004	-0.0745191
ENSG000001	RNF227	0.49630541	0.78193527	-0.0745018
ENSG000001	TTF1	0.27369286	0.63403495	-0.0744643
ENSG000001	ZNF862	0.40723757	0.72768322	-0.0744483
ENSG000001	PLEKHM2	0.0674074	0.39585774	-0.0744169
ENSG00000(	LZTS3	0.56475822	0.81898782	-0.0744164
ENSG000001	FAM117A	0.22252069	0.5899614	-0.0744151
ENSG000001	ITGA10	0.43822241	0.74577269	-0.0743818
ENSG000001	FZR1	0.17620126	0.54121794	-0.0743797
ENSG000001	CSNK1G2	0.15204171	0.51296214	-0.0743603
ENSG000001	BRD8	0.22330413	0.59081204	-0.0743598
ENSG000001	NADSYN1	0.12805362	0.48599936	-0.0742986
ENSG000001	DLG4	0.31883358	0.66618207	-0.0742909
ENSG000001	HSF1	0.26412288	0.6274921	-0.0742391
ENSG000001	METTL26	0.46414778	0.76356307	-0.0742342
ENSG000001	DAP	0.08684088	0.42885945	-0.0741401
ENSG000001	SPTAN1	0.41750158	0.73321581	-0.0740394
ENSG000001	RAB35	0.02854708	0.31345545	-0.0740088
ENSG00000(	PABPC1	0.28424351	0.64253645	-0.0740052
ENSG000001	GPX4	0.34484254	0.68522517	-0.0739723
ENSG000001	FRMD6	0.68027201	0.87644445	-0.0739271
ENSG000001	CNGA1	0.82001188	0.93468999	-0.0738733
ENSG000001	MARCHF9	0.53660511	0.80465987	-0.0738391
ENSG00000(	GNA15	0.2516252	0.6172357	-0.0738037
ENSG00000(	SPEG	0.59770575	0.83641817	-0.0737247
ENSG000001	NDUFB3	0.568289	0.82037292	-0.0736885
ENSG000001	SLX4	0.19766961	0.56540555	-0.0736805
ENSG000001	ZNF497	0.64431212	0.85956564	-0.073669
ENSG000001	PNMA3	0.54858422	0.81157638	-0.0736597
ENSG000001	ACSBG1	0.51983754	0.7967342	-0.0735381
ENSG00000(	CA11	0.45298398	0.75444669	-0.0735352
ENSG000001	U2AF1L4	0.37735835	0.7086984	-0.0735265
ENSG000001	MTCH1	0.08387286	0.42306421	-0.0734572
ENSG000001	KLHDC3	0.27433139	0.63460874	-0.0734405
ENSG00000(	CAPZB	0.1352014	0.49446444	-0.0734263
ENSG00000(	AGPAT4	0.51023395	0.79143652	-0.0734141
ENSG000001	CDC25B	0.4624545	0.7620316	-0.0734138
ENSG00000(	RNF216	0.2422412	0.60792433	-0.0733818
ENSG000001	TSPAN33	0.41777934	0.73327026	-0.0733628
ENSG00000(	CAMSAP3	0.77972519	0.91827559	-0.0733588
ENSG00000(	MAN2B2	0.05379391	0.37194714	-0.0733559

ENSG000002	H4C5	0.64025241	0.85706063	-0.0733503
ENSG000001	SDCBP2	0.4672778	0.76533215	-0.0733385
ENSG000001	LAPTM5	0.00290027	0.20994948	-0.0732643
ENSG000001	PDGFRA	0.86243945	0.95132243	-0.0731764
ENSG000001	PLEKHM3	0.452048	0.75395007	-0.0731491
ENSG000001	VAT1	0.29049493	0.648324	-0.0731419
ENSG000001	FAM168B	0.32284774	0.66871778	-0.0731389
ENSG000001	LYSMD1	0.37800129	0.70909881	-0.073138
ENSG00000C	CHPF2	0.10906611	0.46105349	-0.0731266
ENSG000001	PSKH1	0.18649578	0.55095275	-0.0731265
ENSG000001	PEX16	0.27690468	0.63639084	-0.0731105
ENSG000001	CD7	0.46844971	0.76582944	-0.0730723
ENSG000001	TRAPPC1	0.2397419	0.60643234	-0.0730274
ENSG000001	SAMD10	0.59365264	0.83456808	-0.0729974
ENSG000002	MT-ND4L	0.41275681	0.73063968	-0.0729878
ENSG000001	THAP4	0.29472307	0.65098879	-0.0729867
ENSG000001	NPLOC4	0.06916234	0.39907212	-0.0729276
ENSG000001	FOXRED2	0.58329341	0.82845829	-0.0728607
ENSG00000C	NID2	0.6749289	0.87446425	-0.0727736
ENSG000001	PROS1	0.58504064	0.82919554	-0.0727509
ENSG000001	DPCD	0.543682	0.80862551	-0.0726931
ENSG000001	SEMA4D	0.08037375	0.41684053	-0.0726901
ENSG000001	LRRC46	0.35250729	0.69087104	-0.0726593
ENSG000001	SOX7	0.82308326	0.93539963	-0.0726319
ENSG000001	ENGASE	0.41132228	0.72999304	-0.0725978
ENSG000002	AP4M1	0.35313836	0.69124866	-0.0725699
ENSG000001	SMAGP	0.48351703	0.77585394	-0.0724895
ENSG000001	CDK5RAP3	0.2587659	0.62248674	-0.0724418
ENSG000002	CCNI2	0.62317928	0.84930015	-0.0724335
ENSG000002	SMIM35	0.65717679	0.86522797	-0.0724178
ENSG000001	AGTPBP1	0.45154982	0.75381609	-0.0723982
ENSG00000C	MRPS34	0.3451279	0.68522517	-0.0723088
ENSG000001	LEF1	0.54385302	0.80880578	-0.0723009
ENSG000001	C19orf53	0.4507127	0.75341213	-0.0722725
ENSG000001	RGS19	0.26954196	0.6310094	-0.0722291
ENSG000001	ZNF747	0.30156533	0.65549079	-0.0722178
ENSG000002	NOL12	0.37038449	0.70302695	-0.0722004
ENSG000001	TRAPPC5	0.45983536	0.75954697	-0.0721373
ENSG000001	CLSTN3	0.24289879	0.60853999	-0.0720657
ENSG000002	SPDYE6	0.57154892	0.82164253	-0.0719981
ENSG000001	PSMD4	0.1009618	0.44917064	-0.0719377
ENSG000001	TAOK2	0.06107147	0.38464604	-0.0717923
ENSG000001	ASB13	0.40137689	0.72606468	-0.0717632

ENSG000001	CRACD	0.84659141	0.94500099	-0.0717456
ENSG000001	TUBGCP6	0.37073123	0.70321998	-0.0717223
ENSG000001	ACTL9	0.86607005	0.95247761	-0.0716377
ENSG000001	LRRC20	0.55910264	0.8167476	-0.0716174
ENSG000001	CREG2	0.72966099	0.89828687	-0.071589
ENSG000001	ZFYVE1	0.04794617	0.35958518	-0.0715784
ENSG00000(	DLGAP4	0.10371772	0.45355901	-0.0715442
ENSG000001	FNDC5	0.74499615	0.90628646	-0.071534
ENSG000001	NDST2	0.55152136	0.81323014	-0.0714778
ENSG000001	RNF112	0.53245495	0.80280163	-0.0714427
ENSG000001	RIDA	0.68019136	0.87644445	-0.0713956
ENSG000001	NEDD9	0.36059191	0.69535995	-0.0713627
ENSG000001	RPS14	0.62928025	0.85253429	-0.0713622
ENSG000001	MRPL51	0.40736695	0.72768322	-0.071314
ENSG000001	GNG2	0.47352313	0.76895276	-0.0713126
ENSG000001	CCDC142	0.3916218	0.71964297	-0.0713068
ENSG000001	GPRASP1	0.61849807	0.84697252	-0.0712851
ENSG000002	ABHD14A	0.5211902	0.79758332	-0.07128
ENSG000001	TTC23	0.52892118	0.80100575	-0.0712212
ENSG000001	LRRTM2	0.90387105	0.96801489	-0.0712003
ENSG00000(	RPS20	0.65827078	0.86546032	-0.0711977
ENSG000001	ST3GAL2	0.30312006	0.65593778	-0.0711903
ENSG00000(	ZRANB1	0.29869587	0.65406944	-0.0711758
ENSG000001	MSRB3	0.3556675	0.69207883	-0.0711752
ENSG000001	TOP3A	0.12889558	0.4871454	-0.0710702
ENSG00000(	NISCH	0.26430361	0.6274921	-0.0708794
ENSG000001	CHRFAM7A	0.78171992	0.91916767	-0.0708629
ENSG000001	ATAD3B	0.39662788	0.72299866	-0.0708592
ENSG00000(	NFYC	0.04095757	0.34600833	-0.0708592
ENSG000002	TSTD1	0.41272095	0.73063968	-0.0708177
ENSG000001	PARVG	0.0782559	0.41427772	-0.0707205
ENSG00000(	IFRD1	0.47126598	0.76694135	-0.0706892
ENSG000001	TRAPPC3L	0.74806459	0.90731063	-0.0706881
ENSG000001	TMEM104	0.14255427	0.50068215	-0.0706164
ENSG000002	N/A	0.85642613	0.9490718	-0.070527
ENSG000001	R3HCC1	0.36539805	0.69833839	-0.0705218
ENSG000001	DMKN	0.77814434	0.91795445	-0.0704282
ENSG000001	HMGCR	0.44141339	0.74775474	-0.0704213
ENSG000001	B4GALT2	0.58456853	0.82887597	-0.0704184
ENSG000001	GNA12	0.32414774	0.6699145	-0.0703953
ENSG000001	FGFBP3	0.62632621	0.85066154	-0.0703345
ENSG000001	COLGALT2	0.6286376	0.85212646	-0.0703014
ENSG000001	PPP1R17	0.7401892	0.90464694	-0.0703004

ENSG000001	WNK3	0.71793098	0.89332947	-0.0702737
ENSG000001	TRADD	0.25012303	0.61596135	-0.070273
ENSG000001	ZNF429	0.42058297	0.7349809	-0.0702402
ENSG000002	H2BC8	0.5008489	0.78519683	-0.0702048
ENSG00000C	PHLPP1	0.49524062	0.78130801	-0.0701514
ENSG000001	NDUFAF1	0.31536044	0.66324235	-0.0700838
ENSG000001	SOWAHD	0.3514746	0.69054758	-0.0700744
ENSG000001	KIFC3	0.55504087	0.81433077	-0.0700637
ENSG00000C	SPEN	0.29416041	0.65044158	-0.0700157
ENSG000001	NR1H2	0.26698585	0.62929976	-0.0699981
ENSG000001	TMEM117	0.74506264	0.90628646	-0.0699478
ENSG000001	PALD1	0.5575166	0.81587881	-0.0699089
ENSG000002	PRCD	0.69310679	0.88183499	-0.0698987
ENSG00000C	AKR7A2	0.43434081	0.7438413	-0.0698013
ENSG000001	SUPT5H	0.18523792	0.55032535	-0.0697793
ENSG000001	NPEPPS	0.11280364	0.46772028	-0.0696182
ENSG000001	ANO9	0.53396902	0.80362357	-0.0694675
ENSG000001	TEX45	0.81135908	0.93148819	-0.069441
ENSG000001	TMEM177	0.5937754	0.83456808	-0.0693595
ENSG000002	PLEKHO2	0.38768254	0.71668724	-0.0693131
ENSG00000C	RFC2	0.08928563	0.43342334	-0.0693129
ENSG000001	CLDN15	0.26489607	0.6274921	-0.0692686
ENSG000001	TEX2	0.33701302	0.67898382	-0.069257
ENSG000001	INSR	0.2538846	0.61921212	-0.0692363
ENSG000001	RRBP1	0.35440739	0.69173131	-0.0690795
ENSG000001	TRAF7	0.12961948	0.4871968	-0.069048
ENSG000001	FGF9	0.84536892	0.94432529	-0.0689437
ENSG000001	ATP6V0E2	0.58385718	0.82875175	-0.0689086
ENSG00000C	CAPN1	0.31461382	0.66270362	-0.0688389
ENSG000001	TUSC2	0.17034478	0.53437545	-0.0688263
ENSG000001	ANTXR2	0.23192823	0.599371	-0.0687767
ENSG000001	PLXNB3	0.79808134	0.92603117	-0.0687621
ENSG000001	FBXO27	0.90037127	0.96608561	-0.0687192
ENSG000001	BTBD2	0.26305624	0.62659798	-0.0687144
ENSG000001	ADAMTSL2	0.82130965	0.93539963	-0.0685287
ENSG00000C	PITPNM3	0.63023897	0.85293934	-0.0684963
ENSG000001	EHF	0.898624	0.96576173	-0.0684871
ENSG000001	UBE2J2	0.10884021	0.46045817	-0.0684364
ENSG000001	RPS19	0.55329565	0.81385767	-0.0683984
ENSG000001	CSTB	0.2175733	0.58430991	-0.0683365
ENSG00000C	GRN	0.45111001	0.75364474	-0.0683273
ENSG000001	RAB37	0.06157446	0.38542193	-0.0683088
ENSG000001	PPP1CA	0.20796258	0.57145912	-0.0682967

ENSG000001	EIF1B	0.1651361	0.5286578	-0.0682411
ENSG000001	ANKS6	0.54583907	0.80990486	-0.0681931
ENSG000002	LCAT	0.31690202	0.66422665	-0.0680766
ENSG00000(	GABARAPL2	0.20589567	0.56956674	-0.0680579
ENSG000001	GAK	0.18931081	0.55414453	-0.0680383
ENSG000001	AKT2	0.14610212	0.50430701	-0.0679919
ENSG000001	ACOT1	0.78255197	0.9193471	-0.0679553
ENSG000002	ORAI1	0.32052958	0.66735114	-0.0679383
ENSG000002	CTAGE9	0.85014946	0.94644505	-0.0679359
ENSG000001	ACP5	0.25878345	0.62248674	-0.0678478
ENSG000001	ARHGAP30	0.05300026	0.36981204	-0.0678003
ENSG000001	STXBP5	0.56695924	0.8197023	-0.0677866
ENSG000001	ECE1	0.3830209	0.71198657	-0.0677849
ENSG000001	FCGR3B	0.52150631	0.79758937	-0.0677533
ENSG000001	ADAMTS17	0.6140017	0.8451435	-0.0677426
ENSG000002	MRPL38	0.50589225	0.78868674	-0.0677167
ENSG000001	ERICH1	0.15200283	0.51296214	-0.0676609
ENSG000001	RPP25L	0.39329242	0.72063539	-0.0676097
ENSG000001	SAC3D1	0.47707962	0.77162484	-0.0675863
ENSG000001	SBF1	0.3274696	0.67217574	-0.0675752
ENSG000001	CD1B	0.85252773	0.94733063	-0.0675066
ENSG000001	ACP6	0.45142493	0.75378336	-0.0674554
ENSG00000(	B4GALT1	0.03098778	0.31953017	-0.0674334
ENSG000001	TMEM134	0.43576596	0.74462291	-0.0673736
ENSG00000(	SLC9A3R2	0.59333395	0.83454291	-0.0673705
ENSG000001	ZNF358	0.46555536	0.76440716	-0.0673601
ENSG000001	ADTRP	0.65952579	0.86612102	-0.0673493
ENSG000001	FGF11	0.82891504	0.93682924	-0.0673203
ENSG000001	DBNL	0.04541924	0.35545023	-0.0673118
ENSG000001	AFAP1	0.54499025	0.80952117	-0.0673111
ENSG00000(	FAM50A	0.2667925	0.62929976	-0.0672955
ENSG000001	KCTD20	0.25067563	0.61651321	-0.0672908
ENSG000002	WBP1	0.40661975	0.7275473	-0.0672468
ENSG000001	RAB11FIP5	0.52299536	0.79855893	-0.0671991
ENSG000001	MOGS	0.30053104	0.65493729	-0.0671956
ENSG000001	HS6ST1	0.42618194	0.73856395	-0.0671886
ENSG000001	ZNF18	0.1963264	0.5638811	-0.067184
ENSG000002	FOXD1	0.88575218	0.96095764	-0.0671683
ENSG000001	SUSD3	0.47382402	0.76896358	-0.0671578
ENSG000001	DAZAP2	0.07584627	0.41015711	-0.0671463
ENSG000002	PPP3R1	0.09024275	0.43395124	-0.0671426
ENSG000001	MCF2L	0.55438344	0.81419157	-0.067092
ENSG00000(	HMMR	0.81156615	0.93159417	-0.067089

ENSG000001	RASGEF1A	0.26843569	0.63073283	-0.0670241
ENSG00000C	STPG1	0.50547908	0.78819398	-0.0669814
ENSG000001	SMG5	0.1231081	0.4799225	-0.0669335
ENSG000001	ZNF398	0.36599435	0.69846943	-0.0669173
ENSG000001	HPGDS	0.82407758	0.93583352	-0.0668702
ENSG000001	SEC16A	0.27897516	0.63787563	-0.066843
ENSG000001	AKR1C1	0.67041743	0.87207538	-0.066834
ENSG00000C	ALDH3B1	0.37688501	0.70851939	-0.0668252
ENSG000001	DTX3	0.56210834	0.81804833	-0.0668234
ENSG000001	UQCRH	0.61546489	0.84537475	-0.0668155
ENSG000001	CDC42SE1	0.0232708	0.29798743	-0.066789
ENSG000001	GGA3	0.05145416	0.36502002	-0.066785
ENSG000001	RAB40B	0.64916588	0.86252724	-0.0666403
ENSG000001	FLCN	0.12559327	0.48358024	-0.0666353
ENSG000001	TTI2	0.21426391	0.58004777	-0.0666192
ENSG000001	DPF2	0.04288886	0.35185871	-0.0666025
ENSG000002	ASPRV1	0.56884435	0.82037292	-0.0665854
ENSG000001	CAP1	0.04718529	0.35851546	-0.0665403
ENSG000002	RACK1	0.47002028	0.7662622	-0.0664519
ENSG00000C	MAP3K9	0.61351481	0.8451435	-0.0664281
ENSG000001	DOCK5	0.36510481	0.69801094	-0.0664263
ENSG000001	BIN3	0.09136425	0.43567732	-0.0663532
ENSG000001	ENDOV	0.49282187	0.7804495	-0.0663472
ENSG000001	GET3	0.18163911	0.54636426	-0.0663113
ENSG000001	ZNF319	0.37936963	0.70965485	-0.066309
ENSG000001	TP53INP1	0.54088642	0.80750025	-0.066266
ENSG000001	EPHX2	0.58421736	0.82875566	-0.0662607
ENSG000001	MMD	0.53587346	0.80423066	-0.0662013
ENSG000001	ATP8B3	0.51834782	0.79572786	-0.0661895
ENSG000001	SH2B1	0.39323138	0.72063539	-0.0661837
ENSG000001	NENF	0.51247927	0.79227085	-0.0661569
ENSG000001	CCNDBP1	0.13054143	0.48861254	-0.0660936
ENSG000001	C11orf68	0.33913834	0.68041384	-0.0660657
ENSG000001	SLC18A3	0.84175393	0.94283868	-0.0660515
ENSG000001	TOR4A	0.35285617	0.69109369	-0.0660231
ENSG000001	RBPMS2	0.74399691	0.90628646	-0.0660034
ENSG000001	TMEM216	0.46427981	0.76363273	-0.0660032
ENSG000001	DCAF6	0.30533969	0.65688103	-0.0660003
ENSG000001	STUB1	0.42541687	0.73817463	-0.0659222
ENSG000001	SGTA	0.2024247	0.56807486	-0.065882
ENSG000002	SLC23A3	0.67262403	0.87337134	-0.0658454
ENSG000001	GNAQ	0.38555236	0.7143101	-0.065822
ENSG000002	PRR13	0.16847963	0.53253292	-0.0658031

ENSG000001	EIF4EBP2	0.08680475	0.42885945	-0.0657586
ENSG000001	SLC18A2	0.72574743	0.89686038	-0.0657492
ENSG000001	HMOX1	0.35401376	0.69162813	-0.0657378
ENSG000002	UBXN2B	0.33552677	0.67708232	-0.0657087
ENSG000001	NPIPB13	0.54247005	0.80815796	-0.0656553
ENSG000001	IDUA	0.45422393	0.7555818	-0.0656532
ENSG000001	RABL6	0.39968351	0.72527731	-0.0656392
ENSG000001	RNF25	0.29960769	0.6542516	-0.0655712
ENSG000001	UBAC1	0.23522776	0.60245998	-0.0655241
ENSG00000C	UIMC1	0.05517617	0.37354464	-0.0655166
ENSG000001	H3-3A	0.2956318	0.65140093	-0.0654693
ENSG000001	ZSWIM1	0.24212151	0.60771789	-0.065444
ENSG000001	FOXP1	0.39982492	0.72527731	-0.0654207
ENSG000001	INO80E	0.35707481	0.69298434	-0.0654097
ENSG000001	ZC3H18	0.20222274	0.56797971	-0.0653961
ENSG00000C	CEBPE	0.68178304	0.8771879	-0.0653679
ENSG000001	ARF1	0.10742457	0.45841072	-0.0652523
ENSG000001	NUDT22	0.36897819	0.70186257	-0.0652411
ENSG000001	PPP3R2	0.88958296	0.96178976	-0.0652067
ENSG000001	EXOC3	0.14001526	0.4999841	-0.065195
ENSG00000C	ANKRD24	0.73785164	0.9028257	-0.065194
ENSG00000C	CIRBP	0.30864116	0.65826962	-0.0651674
ENSG000001	GNG7	0.32717636	0.67217574	-0.0651523
ENSG000002	CSNK1E	0.47939694	0.7733699	-0.0651264
ENSG000001	TCFL5	0.54819848	0.81141647	-0.0651185
ENSG000001	PERP	0.66174716	0.86736018	-0.0651067
ENSG000001	SLC29A2	0.61635459	0.84579775	-0.0650972
ENSG000001	GJB3	0.8935785	0.96386329	-0.0650807
ENSG000001	EIF3L	0.49664308	0.78215264	-0.0650684
ENSG000001	TUBGCP3	0.25970224	0.62332376	-0.0650652
ENSG000001	NUTM2B	0.67324622	0.87354948	-0.0650482
ENSG000001	ATG13	0.06067096	0.38451341	-0.0649827
ENSG000001	EMCN	0.85599452	0.94891671	-0.0649657
ENSG000001	INA	0.86663565	0.95264796	-0.0649406
ENSG000001	CUTC	0.503151	0.78652767	-0.0649303
ENSG00000C	CCDC28A	0.19183702	0.55723719	-0.064915
ENSG000001	TCTN2	0.53295149	0.80294166	-0.0649074
ENSG000001	USP20	0.37343866	0.70555312	-0.0648406
ENSG000001	TNFAIP8L2	0.19252217	0.55831085	-0.0648097
ENSG00000C	CYFIP2	0.30385289	0.65635638	-0.0648091
ENSG000001	RPS6KB2	0.31006387	0.65948451	-0.0647943
ENSG000002	TAX1BP3	0.59741866	0.83640463	-0.064787
ENSG000001	ZBTB17	0.16962783	0.53428653	-0.0647053

ENSG000001	GLB1L2	0.7122198	0.89093319	-0.064694
ENSG000001	CHMP1A	0.208271	0.5719087	-0.0646917
ENSG00000C	SPATA20	0.46953473	0.7662622	-0.0646777
ENSG000002	RPS18	0.9255268	0.97525983	-0.0646242
ENSG000001	ATP2A1	0.49030256	0.7800506	-0.0646116
ENSG00000C	MCM2	0.51260505	0.7923054	-0.0646009
ENSG00000C	MAMLD1	0.62500215	0.85016376	-0.0645938
ENSG000001	LMAN2L	0.2907229	0.64832683	-0.0645309
ENSG00000C	BAX	0.30406462	0.65635638	-0.0644488
ENSG000002	TMEM238	0.56631458	0.8197023	-0.0644427
ENSG00000C	NCAPH2	0.43899903	0.74609757	-0.0644205
ENSG00000C	NCOA1	0.33074141	0.6742308	-0.0644094
ENSG000002	ZNF486	0.48586201	0.77746541	-0.064316
ENSG000001	CDK9	0.19816529	0.56558444	-0.0642941
ENSG00000C	MAP2K4	0.31054354	0.65949478	-0.0642495
ENSG000001	TMEM74B	0.73199161	0.89917898	-0.0642477
ENSG000001	TEX11	0.88283082	0.96000425	-0.064219
ENSG000001	ARR3	0.71709697	0.89290672	-0.064142
ENSG000001	BAP1	0.0817642	0.41959714	-0.0641347
ENSG000001	TBC1D13	0.23771858	0.60402516	-0.064082
ENSG000001	GINS4	0.56366856	0.81863326	-0.0640619
ENSG000001	PRKCD	0.33985933	0.68127111	-0.0640597
ENSG000001	UPK3A	0.59812374	0.83653786	-0.0640596
ENSG000002	TTLL3	0.15609979	0.51859362	-0.0640254
ENSG000001	TMTC1	0.61302415	0.84505867	-0.0640141
ENSG000001	DOCK2	0.18593647	0.55069275	-0.0640139
ENSG00000C	BRCA1	0.41276365	0.73063968	-0.0639989
ENSG000002	NCKIPSD	0.35495213	0.6919142	-0.0639117
ENSG000001	EXOSC4	0.49099149	0.78013786	-0.0638952
ENSG000001	TMEM160	0.56898258	0.82037292	-0.0638866
ENSG000001	AGTRAP	0.27839538	0.63759905	-0.0638356
ENSG000001	AGFG2	0.51183598	0.79197129	-0.0638246
ENSG000001	SAP130	0.22630114	0.59351505	-0.0638224
ENSG000001	KLHDC2	0.51723957	0.79538014	-0.0637937
ENSG00000C	PGS1	0.31323908	0.6613866	-0.0637845
ENSG000001	D2HGDH	0.55439441	0.81419157	-0.0637633
ENSG000002	ZNF579	0.55180163	0.81325253	-0.0637582
ENSG00000C	MXD1	0.46331217	0.76294212	-0.0637448
ENSG000001	NCF1	0.66419049	0.86838401	-0.0637437
ENSG00000C	SIRPG	0.55470816	0.81429195	-0.063732
ENSG000001	FCGR2A	0.40136086	0.72606468	-0.0637196
ENSG000001	CRYBA1	0.7987291	0.92603117	-0.063681
ENSG000001	SLC46A2	0.60787158	0.84136143	-0.0636048

ENSG000001	BCAP31	0.18877798	0.55348285	-0.0635846
ENSG000001	SOX13	0.5271465	0.80038735	-0.0635532
ENSG000001	MAN1B1	0.37124113	0.70362349	-0.0635482
ENSG000001	PXK	0.48038755	0.77383118	-0.0634706
ENSG000001	PIDD1	0.52127446	0.79758332	-0.0634508
ENSG000002	GDF10	0.88148117	0.95922591	-0.0634306
ENSG000001	ETV4	0.83732353	0.94120179	-0.0634034
ENSG000001	TMEM258	0.46998286	0.7662622	-0.0633974
ENSG000001	PRELID2	0.79659653	0.92526918	-0.0633537
ENSG000001	KLHL35	0.79768404	0.9258334	-0.0633473
ENSG000001	ZHX2	0.24102282	0.60721888	-0.0633046
ENSG000001	FBXW5	0.35808981	0.69382497	-0.0633039
ENSG000001	ILVBL	0.4630404	0.76274596	-0.0632507
ENSG00000C	KCNK6	0.4036134	0.72672584	-0.0632037
ENSG000001	CD3E	0.55848838	0.81607054	-0.0631948
ENSG000001	RALY	0.2755181	0.63512113	-0.0631666
ENSG00000C	NFKB2	0.36833834	0.70105611	-0.0631653
ENSG000002	KRTAP10-3	0.88714344	0.96095764	-0.0631638
ENSG000001	CORO6	0.69030739	0.88074719	-0.063151
ENSG000001	ACTR3B	0.41267212	0.73063968	-0.0631348
ENSG000001	FAM104B	0.50150492	0.7853582	-0.0631127
ENSG000002	N/A	0.8627038	0.951353	-0.0631123
ENSG000001	SGSM2	0.43525086	0.744461	-0.0630507
ENSG000002	TNFRSF25	0.61605578	0.84569598	-0.0630389
ENSG000001	RBM47	0.55299814	0.81383402	-0.0630197
ENSG000001	FASN	0.52099384	0.79745258	-0.0629409
ENSG000001	PGAP3	0.46042746	0.76015269	-0.0629274
ENSG00000C	RANBP3	0.15211632	0.51296214	-0.0629274
ENSG000001	RABAC1	0.40542808	0.72703815	-0.0629121
ENSG000001	METRN	0.61429293	0.8451435	-0.0628938
ENSG00000C	ACTL6B	0.89483618	0.96412642	-0.0628821
ENSG00000C	CLCA4	0.85753181	0.94961808	-0.0627053
ENSG000001	GRB2	0.21207147	0.57697795	-0.0626843
ENSG000001	NPM2	0.87161543	0.95449478	-0.0626555
ENSG000001	CD151	0.39978906	0.72527731	-0.0626334
ENSG000001	TMEM187	0.24240529	0.60796025	-0.0625481
ENSG000001	NDUFAF3	0.40225639	0.72669629	-0.0624723
ENSG000001	SF1	0.09025688	0.43395124	-0.0623464
ENSG00000C	CPSF1	0.39951506	0.72527731	-0.062283
ENSG000001	MAGED1	0.47129903	0.76694135	-0.062268
ENSG000001	CAPN12	0.63676833	0.85580792	-0.0622471
ENSG000001	CYP4B1	0.81802024	0.93332827	-0.062216
ENSG000002	UQCC3	0.56241071	0.81818741	-0.0621714

ENSG000001	IGHMBP2	0.26351944	0.62687307	-0.0621411
ENSG000001	AXIN1	0.31028867	0.65949478	-0.0621145
ENSG000001	KMT5A	0.30801426	0.65762361	-0.0619961
ENSG000002	UHRF1	0.53340786	0.80312903	-0.0619563
ENSG000000	KATNIP	0.24004856	0.60689277	-0.0619348
ENSG000001	RPS27A	0.70885296	0.88947345	-0.0619224
ENSG000001	BHLHB9	0.66211558	0.86741192	-0.0619122
ENSG000001	CD109	0.79770951	0.9258334	-0.061879
ENSG000001	CIAO3	0.4514251	0.75378336	-0.0618728
ENSG000001	ANAPC15	0.51626345	0.79515933	-0.0618348
ENSG000000	RAB27A	0.26235079	0.62579547	-0.0618157
ENSG000001	ETV2	0.80416533	0.92881463	-0.0617952
ENSG000001	VKORC1	0.44494926	0.74940749	-0.0617918
ENSG000001	SFTPA1	0.87174041	0.95451137	-0.0617913
ENSG000001	FAM219A	0.27654125	0.6360577	-0.0617449
ENSG000001	LGALS12	0.5911593	0.83299508	-0.0617383
ENSG000000	AAAS	0.36185218	0.69588937	-0.0617307
ENSG000002	N/A	0.90458706	0.96810345	-0.061675
ENSG000002	PET100	0.44291415	0.74873472	-0.0616603
ENSG000001	MAML1	0.25534284	0.62028751	-0.0616008
ENSG000001	HLA-DRB5	0.64481019	0.85991395	-0.0615287
ENSG000001	STK11IP	0.23791291	0.60402516	-0.0615178
ENSG000001	SLC39A13	0.40091599	0.72606468	-0.0614371
ENSG000001	IRAK1	0.24980612	0.61582699	-0.061435
ENSG000002	IKBKG	0.37571182	0.70743725	-0.0614134
ENSG000002	N/A	0.7695399	0.91386073	-0.0614083
ENSG000001	ZNF221	0.87043346	0.95426978	-0.0613659
ENSG000001	POU2F3	0.87687585	0.9567976	-0.0613185
ENSG000001	MXRA8	0.57350228	0.82246568	-0.0613179
ENSG000000	SNX11	0.06408337	0.39043354	-0.061152
ENSG000001	TCTA	0.17502322	0.53975461	-0.0611452
ENSG000001	IQCD	0.77388746	0.91586084	-0.0611411
ENSG000001	PLEKHD1	0.86881102	0.95366611	-0.0611303
ENSG000001	RETN	0.60116399	0.83799671	-0.0611023
ENSG000001	CABP5	0.72190945	0.89512361	-0.0610958
ENSG000000	UFD1	0.39226193	0.72005693	-0.0610766
ENSG000002	PPP1R3E	0.53776627	0.80550839	-0.0610751
ENSG000001	BOLA2	0.90020801	0.96608561	-0.0610386
ENSG000001	ARHGAP26	0.37842043	0.7092687	-0.061017
ENSG000001	IL11RA	0.54742457	0.81118038	-0.0609928
ENSG000001	TMEM154	0.348578	0.6878781	-0.0609843
ENSG000001	GID8	0.1409595	0.50051652	-0.0609109
ENSG000001	CYB561D1	0.26307828	0.62659798	-0.0608865

ENSG000001	HORMAD2	0.84976982	0.94644505	-0.0608296
ENSG000001	NOVA1	0.88528484	0.96095764	-0.060775
ENSG000001	TTBK1	0.91119172	0.97086554	-0.0607647
ENSG00000(	TMEM176A	0.52655458	0.80019714	-0.0607362
ENSG000001	SLC5A6	0.34639855	0.68614821	-0.0607196
ENSG000001	RHOF	0.51251282	0.79227085	-0.0606574
ENSG00000(	TRIP6	0.35861978	0.69382497	-0.0606324
ENSG000002	EIF4EBP3	0.65329594	0.86393759	-0.0606205
ENSG000001	SPSB2	0.36896352	0.70186257	-0.060583
ENSG000001	PUS1	0.45259006	0.75425483	-0.0604833
ENSG000001	UBXN11	0.4731216	0.76859183	-0.0604712
ENSG00000(	LTBP4	0.57748829	0.82475832	-0.0604699
ENSG000001	MPG	0.37129064	0.70362349	-0.0604055
ENSG000001	CCDC130	0.37918986	0.70965485	-0.0603033
ENSG000001	SPNS3	0.7052621	0.88735689	-0.0602842
ENSG000001	MZT2B	0.52993898	0.8016768	-0.0602781
ENSG00000(	MUL1	0.08059375	0.41748748	-0.0602648
ENSG000001	TMIGD2	0.59595369	0.83576612	-0.0601294
ENSG000001	MBOAT2	0.50155136	0.7853582	-0.0601193
ENSG000001	FRMD4A	0.42415563	0.73732623	-0.0600761
ENSG000001	NPHP4	0.44620513	0.75046952	-0.0600673
ENSG000001	CACNA2D4	0.46486391	0.76408722	-0.0600648
ENSG000001	TMEM107	0.56773981	0.82037292	-0.0600314
ENSG000001	CAMK2N1	0.58640587	0.82959975	-0.0600249
ENSG000001	TRIM17	0.54282863	0.80839296	-0.0600234
ENSG000001	SYTL3	0.3720152	0.70415443	-0.0599222
ENSG000001	FAHD2B	0.64350882	0.85940463	-0.0598794
ENSG000001	PROZ	0.7744836	0.91617933	-0.0598779
ENSG000001	LMBR1L	0.28248707	0.64051704	-0.0598308
ENSG000002	ZNF688	0.39606686	0.72241491	-0.0596927
ENSG000001	IL13RA1	0.34613309	0.68588759	-0.0596896
ENSG000001	TRPT1	0.48316582	0.77585394	-0.0596867
ENSG000001	WDR24	0.46948753	0.7662622	-0.0596619
ENSG000001	PELP1	0.45087678	0.75348767	-0.0595964
ENSG00000(	GRAMD4	0.45471873	0.75586282	-0.059551
ENSG000001	CUL9	0.37506584	0.70720386	-0.0595124
ENSG000001	RHPN2	0.64391315	0.85940463	-0.0594478
ENSG000001	FANCE	0.56949757	0.82041807	-0.0594393
ENSG000001	NOP53	0.57530469	0.82344878	-0.0594233
ENSG000001	KCNE3	0.34301222	0.68362458	-0.0592719
ENSG00000(	LIPE	0.48895862	0.77927179	-0.059214
ENSG000001	PERM1	0.78984364	0.92277243	-0.0591822
ENSG000001	NAA60	0.27904005	0.63787563	-0.059176

ENSG000001	CDC42EP4	0.37793401	0.70909881	-0.0591543
ENSG000001	FCMR	0.57661455	0.8241744	-0.0591054
ENSG000001	MTG2	0.19829202	0.56584667	-0.0591053
ENSG000001	CYTIP	0.24486835	0.61092923	-0.0590883
ENSG000001	BIK	0.66947674	0.87133577	-0.0590871
ENSG000001	TLCD2	0.84981818	0.94644505	-0.0590589
ENSG000001	PRPF6	0.23720866	0.60397634	-0.0590463
ENSG000001	UAP1L1	0.43553346	0.74461931	-0.0588338
ENSG000001	HTRA2	0.39448233	0.72171376	-0.0588187
ENSG00000(	PHF20	0.27841393	0.63759905	-0.0588107
ENSG00000(	MGST1	0.61581836	0.84558501	-0.0588088
ENSG000001	RHBDD3	0.51747737	0.7955198	-0.058787
ENSG000001	ERP27	0.48629605	0.77752459	-0.0587629
ENSG000001	POLM	0.30634217	0.65735608	-0.0587026
ENSG000002	DCTN1	0.32109225	0.66766831	-0.0586957
ENSG000001	FBXO10	0.52918931	0.8011926	-0.0586256
ENSG000001	ZNF346	0.30959597	0.65916845	-0.0586254
ENSG000001	SCUBE3	0.64583155	0.86025245	-0.0585334
ENSG000001	TBL3	0.40851926	0.7282887	-0.0585177
ENSG000001	CORO2A	0.40385967	0.72672584	-0.0584955
ENSG000001	RPL21	0.76244946	0.91167139	-0.058487
ENSG000001	PNMA1	0.53891265	0.80646763	-0.0584745
ENSG000001	PPL	0.51419477	0.7940067	-0.0584465
ENSG000001	STK35	0.28019716	0.6389512	-0.0584197
ENSG00000(	LRRC23	0.61689719	0.84613614	-0.058415
ENSG000001	INPP5D	0.17589417	0.54090788	-0.0584033
ENSG000001	SSTR1	0.87690166	0.9567976	-0.0583882
ENSG000001	SCN4B	0.86443396	0.95224252	-0.0583644
ENSG000001	RCVRN	0.83110456	0.9379097	-0.0583518
ENSG00000(	MYO15A	0.60351353	0.83883394	-0.0583463
ENSG000001	LCK	0.61333998	0.8451435	-0.0583304
ENSG000001	NUMBL	0.52626243	0.80016136	-0.0583037
ENSG000001	PIM1	0.29978641	0.6542516	-0.0582304
ENSG00000(	NTN1	0.76485495	0.91255418	-0.0582238
ENSG000001	SLC24A3	0.57113658	0.82159192	-0.0582225
ENSG000001	LRRFIP1	0.18612397	0.55069275	-0.0582059
ENSG000001	SEMA4C	0.66046529	0.86700658	-0.0581801
ENSG000001	MRPS2	0.45094333	0.75352135	-0.0581486
ENSG00000(	B4GALT7	0.34745067	0.6869057	-0.0580976
ENSG000001	FLT1	0.71606394	0.89229516	-0.0580949
ENSG000001	PPM1A	0.44990468	0.75279291	-0.0580851
ENSG000001	TTLL1	0.5540423	0.8140426	-0.0580711
ENSG000001	TOP1MT	0.51047049	0.79143652	-0.0580706

ENSG000001	PSMD13	0.18650023	0.55095275	-0.0580519
ENSG000001	BRD3	0.280704	0.63927558	-0.0580147
ENSG000002	PTGES3L	0.81403906	0.93216232	-0.0579892
ENSG000001	PLCB3	0.44748747	0.75099423	-0.0579854
ENSG00000C	SDF4	0.25277831	0.61877905	-0.0579764
ENSG000001	ZSCAN18	0.49898489	0.78371229	-0.0579354
ENSG000001	ADRM1	0.35309944	0.69124866	-0.0579174
ENSG000001	GNAL	0.46752897	0.76548028	-0.0578854
ENSG000002	NAP1L4	0.73680655	0.90215881	-0.0578723
ENSG000001	SELENOK	0.48851117	0.77894097	-0.0578531
ENSG000001	FAM160B2	0.4169346	0.73289674	-0.057852
ENSG000001	SOX6	0.68594069	0.8799229	-0.0578199
ENSG000001	MTRES1	0.5694028	0.82041807	-0.0577833
ENSG000001	CENATAC	0.49403081	0.7812211	-0.0577493
ENSG000001	MIGA2	0.40428154	0.72672584	-0.0577448
ENSG000001	RASA4	0.73719978	0.90247451	-0.0577194
ENSG000001	TLR6	0.50265967	0.78619158	-0.057713
ENSG000001	SMPDL3B	0.67079544	0.87216822	-0.0576992
ENSG000001	GTF3C5	0.30860735	0.65826962	-0.057699
ENSG000001	FLYWCH2	0.57307158	0.82235115	-0.057666
ENSG000001	GAL3ST3	0.88419034	0.9606711	-0.0576416
ENSG000001	TRPC4AP	0.08655442	0.42847075	-0.0576256
ENSG000001	DUSP9	0.86936894	0.9539778	-0.0576
ENSG000001	DDX41	0.26792135	0.63017463	-0.0575812
ENSG00000C	ITGA3	0.64043337	0.85706063	-0.0575407
ENSG000001	EDAR	0.73181213	0.89916258	-0.0575327
ENSG00000C	TRMT2A	0.458225	0.75765778	-0.0575001
ENSG000001	RPS2	0.53830616	0.80616834	-0.0574648
ENSG000001	CKB	0.63085992	0.85326916	-0.0574579
ENSG000001	P2RX2	0.87944097	0.95817324	-0.0574543
ENSG000001	PNRC1	0.22002189	0.58729171	-0.057389
ENSG000002	H2BC15	0.66310676	0.86792185	-0.0573597
ENSG000001	SMARCA4	0.38362815	0.7123445	-0.0573544
ENSG000001	UNC45A	0.35327502	0.69126085	-0.0573452
ENSG000001	PIP4P2	0.463898	0.7634274	-0.057321
ENSG000001	KCNC3	0.46986986	0.7662622	-0.0572356
ENSG000001	PPP2R1A	0.26057272	0.62402939	-0.0571779
ENSG000001	MGAT5	0.41213885	0.73047686	-0.0570729
ENSG000001	TBPL2	0.8889337	0.96164016	-0.057053
ENSG000001	PELI2	0.41319693	0.73077485	-0.0570439
ENSG000001	STPG3	0.83946648	0.94217303	-0.0568686
ENSG00000C	ENO1	0.31643211	0.66372668	-0.0568275
ENSG000001	SPINT2	0.32189564	0.66822528	-0.0567705

ENSG000002	C10orf143	0.64003507	0.85706063	-0.0567572
ENSG000001	GAREM1	0.86736803	0.9528412	-0.0567338
ENSG000001	PPFIBP2	0.25695142	0.62086214	-0.0567074
ENSG000001	ZNRF3	0.75933447	0.91125749	-0.0566785
ENSG000001	LDHAL6A	0.72784154	0.89720339	-0.0566423
ENSG000001	RNF149	0.40421558	0.72672584	-0.0566351
ENSG000001	C7orf26	0.27254177	0.63371913	-0.0566184
ENSG000001	NSD1	0.25982949	0.62338681	-0.0564861
ENSG000001	GPCPD1	0.59652377	0.83600051	-0.0564685
ENSG000001	HDAC11	0.47679525	0.77130883	-0.0564338
ENSG000001	TGFBI	0.4483639	0.75136004	-0.0563747
ENSG000001	ZFYVE27	0.17835105	0.54332516	-0.0563269
ENSG000001	TFIP11	0.0733905	0.40497004	-0.0563252
ENSG000001	RARG	0.52482404	0.79902184	-0.0562975
ENSG00000C	SH2D3C	0.42293147	0.73681741	-0.0562717
ENSG000001	SUGP1	0.12936243	0.4871968	-0.0562365
ENSG000001	MMS19	0.43093918	0.74126857	-0.0562315
ENSG00000C	RIPOR1	0.32698786	0.67217574	-0.0562263
ENSG000001	COPS3	0.44709493	0.75099423	-0.0562158
ENSG000001	DOP1B	0.26958873	0.6310094	-0.056203
ENSG000001	CLK3	0.06872723	0.39825823	-0.0561451
ENSG000001	FBXW4	0.31635285	0.66372668	-0.0561438
ENSG000001	ARFGAP1	0.28341231	0.64165879	-0.0561434
ENSG000001	TFDP1	0.43954056	0.74621708	-0.0561385
ENSG000001	CDC123	0.29468872	0.65098879	-0.0561282
ENSG000001	TOMM40	0.35356625	0.69141939	-0.0561006
ENSG000001	ZNF554	0.61239319	0.84445251	-0.0560975
ENSG000001	OS9	0.22618233	0.59329927	-0.0560425
ENSG000002	CCPG1	0.5422047	0.80815796	-0.0559797
ENSG000001	TRIM65	0.54397648	0.80884121	-0.0559677
ENSG000001	RUFY1	0.29264995	0.64955906	-0.0559561
ENSG000001	ZNF284	0.56932453	0.82041807	-0.0559413
ENSG000001	ARHGEF18	0.34240986	0.68336859	-0.0559184
ENSG00000C	PIGS	0.17629499	0.54121794	-0.0559118
ENSG000001	P2RY4	0.90298045	0.96763453	-0.0558869
ENSG00000C	EPN2	0.60193756	0.83845247	-0.0558444
ENSG000001	FBF1	0.57312779	0.82235115	-0.0558228
ENSG000001	H2AC13	0.76958395	0.91386073	-0.0557853
ENSG000001	FAM209A	0.55950008	0.81696069	-0.055773
ENSG000001	SPTBN2	0.76101799	0.91141904	-0.0557544
ENSG000002	NAIP	0.77940625	0.91827559	-0.0556963
ENSG000001	TMEM259	0.43943885	0.74621708	-0.05569
ENSG00000C	FBXO42	0.15958659	0.52219103	-0.055687

ENSG000001	CTRC	0.74085914	0.90513447	-0.0556767
ENSG00000C	RHOA	0.11274475	0.46759554	-0.055614
ENSG000001	PSMB7	0.29089734	0.64862677	-0.0555991
ENSG000001	ARRDC4	0.66924656	0.87127337	-0.0555904
ENSG00000C	GLG1	0.34780425	0.68740599	-0.0555806
ENSG000001	FKBP2	0.46996333	0.7662622	-0.0555709
ENSG000002	POLR2J3	0.57740297	0.824709	-0.0555626
ENSG000001	EXD3	0.58150213	0.82722887	-0.0555206
ENSG000001	NTN3	0.87240867	0.95459934	-0.0555197
ENSG000001	ADCK5	0.56991946	0.82058247	-0.0554486
ENSG000002	CTXN2	0.82784952	0.93660278	-0.0554474
ENSG000001	ATAD3A	0.48331967	0.77585394	-0.0554121
ENSG000001	NCOA6	0.41066834	0.72975628	-0.0553835
ENSG000001	WDR73	0.53988803	0.80698777	-0.0553119
ENSG00000C	CDK14	0.49999123	0.78433741	-0.0553114
ENSG000001	FASTK	0.51355135	0.79316402	-0.0552964
ENSG00000C	CXCL2	0.88656946	0.96095764	-0.0552652
ENSG000001	ACTR1A	0.20538909	0.56904198	-0.055237
ENSG000001	LHFPL6	0.79106233	0.92297405	-0.0551685
ENSG000001	ZNF599	0.59952713	0.83723419	-0.0551265
ENSG000001	SDHAF2	0.27179491	0.63323084	-0.0549851
ENSG000002	H3C15	0.86834091	0.95343718	-0.0549664
ENSG00000C	FNDC8	0.90272994	0.96756393	-0.0549631
ENSG000001	CLN3	0.24695161	0.61292467	-0.0548745
ENSG00000C	DHPS	0.57884846	0.82557375	-0.0548698
ENSG000001	CBX4	0.30301037	0.65591444	-0.0548591
ENSG000002	CLIC1	0.91411768	0.97149685	-0.0548185
ENSG000001	SLC25A45	0.48051819	0.77383118	-0.0548115
ENSG000001	TAF6L	0.50163112	0.7853582	-0.0547656
ENSG000001	IFT52	0.50978378	0.79143652	-0.054744
ENSG000001	CDH23	0.24836305	0.61459845	-0.054737
ENSG00000C	NGEF	0.90727112	0.96868131	-0.0547251
ENSG000001	ACBD4	0.55665701	0.81537572	-0.0546861
ENSG000001	FAHD1	0.52033148	0.79709497	-0.0546705
ENSG000001	MGAT3	0.69622	0.88256705	-0.054646
ENSG000001	SLC25A11	0.31951805	0.66652314	-0.0546295
ENSG00000C	TBC1D22A	0.24505805	0.6111208	-0.0546293
ENSG000001	METTL9	0.42888711	0.74051893	-0.0545745
ENSG000001	ZNF69	0.72666018	0.89706038	-0.0545316
ENSG000001	FHL3	0.54959908	0.81217374	-0.0545293
ENSG000001	AREL1	0.20988025	0.57415357	-0.0544644
ENSG000001	MORN4	0.79230947	0.92314956	-0.0544452
ENSG000001	CSF1R	0.50781113	0.79065693	-0.0544025

ENSG000001	OAZ3	0.79039278	0.92280247	-0.0543507
ENSG000001	LARGE2	0.65137603	0.8634351	-0.0543332
ENSG00000(	WNK1	0.51642146	0.79520352	-0.0543016
ENSG000001	CHRAC1	0.0955599	0.4413663	-0.0542541
ENSG000001	TRIM66	0.47984247	0.77364477	-0.0542298
ENSG000001	KPNA1	0.38055006	0.70987972	-0.0542075
ENSG000001	PRCC	0.15943788	0.52219103	-0.0542041
ENSG000001	WRAP73	0.32811543	0.67242335	-0.0541987
ENSG000001	EVI2B	0.27342584	0.6339378	-0.0541902
ENSG000001	JAK1	0.2929282	0.64967561	-0.0541862
ENSG000001	TSPAN18	0.60309403	0.83873753	-0.0541365
ENSG00000(	SIRT4	0.73047044	0.89847521	-0.0540884
ENSG000001	TUBB6	0.6380826	0.8564206	-0.0540226
ENSG000001	JUNB	0.50445338	0.78712373	-0.0539998
ENSG00000(	PEBP1	0.54660302	0.81063154	-0.0539682
ENSG00000(	PSD	0.61012679	0.84290213	-0.0539625
ENSG000001	CTDNEP1	0.30291228	0.65591444	-0.0539618
ENSG00000(	TCF3	0.50162749	0.7853582	-0.0538331
ENSG000001	ASB3	0.67401651	0.87391249	-0.0538236
ENSG000002	H2AC19	0.81351858	0.93185899	-0.0538086
ENSG000001	SOS2	0.48299459	0.77585394	-0.0537522
ENSG00000(	PKM	0.34826895	0.68768877	-0.0537519
ENSG000001	CLEC4A	0.49930614	0.78408977	-0.053723
ENSG000001	CBL	0.41069996	0.72975628	-0.0537203
ENSG00000(	KLF6	0.11914427	0.47578591	-0.0537151
ENSG000001	DNAH17	0.53347124	0.80314998	-0.0536742
ENSG000001	C15orf39	0.63469452	0.85504577	-0.0536644
ENSG000001	RUSC2	0.51693004	0.79538014	-0.0536252
ENSG000001	ZNF460	0.73524768	0.90147373	-0.0536077
ENSG000001	ETS2	0.40743357	0.72768322	-0.0534948
ENSG000001	SLC25A52	0.91027985	0.97033967	-0.0534896
ENSG000001	HMCES	0.46816224	0.76574496	-0.0534626
ENSG000001	NT5C	0.46649726	0.76486809	-0.0534578
ENSG000002	GGTA1	0.68017389	0.87644445	-0.0534288
ENSG000001	TNK1	0.65123567	0.8634351	-0.0533658
ENSG000001	EDIL3	0.87984273	0.95827424	-0.0533385
ENSG000001	ZNF575	0.66557586	0.86935526	-0.0533309
ENSG000001	PA2G4	0.42819853	0.73996341	-0.0533024
ENSG000002	NUDT18	0.54337085	0.8084284	-0.0532774
ENSG000001	RIMKLA	0.86145875	0.95081668	-0.0532335
ENSG000001	SLC1A5	0.5231516	0.79870477	-0.0532292
ENSG000001	PLEKHJ1	0.44518648	0.74940749	-0.0531469
ENSG000001	BHLHE41	0.65377172	0.86420392	-0.0531411

ENSG000001	QARS1	0.4679729	0.76574496	-0.0531358
ENSG000002	PCDHGB7	0.89899107	0.96576173	-0.053123
ENSG000001	HAGHL	0.71689451	0.89272307	-0.0530406
ENSG00000C	ARF5	0.29402791	0.65044158	-0.0530053
ENSG000001	HES1	0.70090896	0.88498263	-0.05298
ENSG000001	SH2D3A	0.65268315	0.86379349	-0.0529576
ENSG000001	BEX5	0.80050982	0.92689709	-0.0527134
ENSG000001	PCYT1A	0.05256345	0.36883004	-0.052693
ENSG000001	ARHGAP24	0.29944816	0.6542516	-0.0526865
ENSG00000C	CTSA	0.50331803	0.78656158	-0.0526251
ENSG00000C	ST6GALNAC1	0.69273034	0.88158966	-0.0526163
ENSG000001	LRRC45	0.61949359	0.84711589	-0.0526148
ENSG000001	AGAP3	0.47994099	0.77364477	-0.0525861
ENSG000001	RACGAP1	0.58000091	0.82610777	-0.052511
ENSG000001	MERTK	0.74247693	0.90558693	-0.0525073
ENSG000001	PDGFC	0.76170404	0.91151487	-0.0525072
ENSG000002	REPIN1	0.40108372	0.72606468	-0.0524992
ENSG000001	BRF1	0.49180211	0.7804495	-0.0524575
ENSG000001	PIK3R5	0.03287982	0.32362351	-0.0524193
ENSG000001	LDHB	0.74649835	0.90664176	-0.0523702
ENSG000001	ACOXL	0.77934196	0.91827559	-0.0523342
ENSG000001	ZNF763	0.56393456	0.81864675	-0.0522895
ENSG000001	CACTIN	0.43829489	0.74581788	-0.0522766
ENSG00000C	ZDHHC8	0.50454434	0.78719001	-0.052233
ENSG000002	RNF212B	0.86399075	0.95219018	-0.0522233
ENSG00000C	COL5A3	0.71767595	0.89321665	-0.0522186
ENSG00000C	GTF3C1	0.41159891	0.73015749	-0.0522026
ENSG00000C	SLC35C2	0.31209679	0.6608083	-0.0521948
ENSG000001	PTPN6	0.47803065	0.77199385	-0.0521562
ENSG000001	EEF1AKMT1	0.7572407	0.91082077	-0.0521422
ENSG000001	CDKN2D	0.35797969	0.69382497	-0.0521
ENSG000001	TRIM47	0.67469577	0.8743925	-0.0520451
ENSG00000C	AKR1B1	0.60144587	0.83818355	-0.0520321
ENSG000001	IRF3	0.39929331	0.72496091	-0.0520084
ENSG000001	VDR	0.47566849	0.77034004	-0.0519854
ENSG000001	HTR7	0.82661227	0.93615843	-0.051982
ENSG000001	CAB39	0.50945395	0.79136944	-0.0519754
ENSG000001	TTLL7	0.70079911	0.88498263	-0.051973
ENSG000001	CHPF	0.71269843	0.89116324	-0.0519315
ENSG000001	IDI2	0.90852254	0.96935737	-0.0519303
ENSG000001	BCAS4	0.65518438	0.86446832	-0.051786
ENSG00000C	PAF1	0.39516346	0.72209218	-0.0517854
ENSG00000C	NEO1	0.63738216	0.8560819	-0.0517585

ENSG000001	ZNF48	0.60097407	0.83799671	-0.0517096
ENSG000001	TAF6	0.43673383	0.74496191	-0.0516911
ENSG000001	ATN1	0.54879007	0.81161901	-0.0516747
ENSG000001	MT-CO1	0.55438919	0.81419157	-0.0516358
ENSG000001	DEAF1	0.4639441	0.7634274	-0.0516318
ENSG000002	PI4KA	0.29317151	0.64988031	-0.0516065
ENSG000001	SLA2	0.47785882	0.77199385	-0.0515899
ENSG000001	SEPTIN9	0.45450752	0.75582124	-0.0515701
ENSG000001	CFAP54	0.75681491	0.91082077	-0.0515455
ENSG000001	OTUB1	0.35974467	0.69484359	-0.0515289
ENSG000001	NCOR1	0.39690074	0.72308765	-0.0514847
ENSG000001	PDLIM1	0.62103757	0.84844654	-0.0514525
ENSG000001	TPRN	0.5635596	0.81863326	-0.0514289
ENSG000001	MSRB2	0.62263389	0.84922092	-0.0513996
ENSG00000C	WDR54	0.71992841	0.8945116	-0.0513597
ENSG000001	RPL10	0.63836794	0.8564206	-0.0513126
ENSG000001	ZNF511	0.61899424	0.84711589	-0.0512863
ENSG000001	RAB15	0.4635231	0.76315398	-0.0512841
ENSG000001	FAM219B	0.57820772	0.82527766	-0.0512619
ENSG000001	PPP1R35	0.59585589	0.83576612	-0.0512099
ENSG000001	PLD3	0.43495803	0.74433605	-0.0511951
ENSG000001	SCAND1	0.53883743	0.8064433	-0.05118
ENSG000001	SSU72	0.19934637	0.56600138	-0.0511605
ENSG000001	STBD1	0.6323139	0.85418109	-0.0511452
ENSG000001	HIGD2A	0.50943354	0.79136944	-0.0510981
ENSG000001	GYS1	0.35864217	0.69382497	-0.0510947
ENSG000001	SDF2	0.27592412	0.63548653	-0.0510429
ENSG000001	VILL	0.54067593	0.80733448	-0.0510217
ENSG00000C	ZNF324	0.40841546	0.72824971	-0.0510031
ENSG000001	NTAN1	0.91826342	0.97272242	-0.0509736
ENSG00000C	U2AF2	0.24367879	0.60937379	-0.0508942
ENSG000001	RABL2A	0.59564148	0.83572573	-0.05086
ENSG000001	PKN3	0.68235032	0.87750056	-0.0507587
ENSG000001	AMIGO1	0.70244931	0.88567086	-0.0507479
ENSG000001	ZNF395	0.54861504	0.81157638	-0.0507272
ENSG000001	YIPF4	0.40088362	0.72606468	-0.0506938
ENSG000001	SAP30BP	0.13246731	0.49106659	-0.0506699
ENSG000001	TADA3	0.29012026	0.648324	-0.0506447
ENSG000001	C19orf47	0.41318967	0.73077485	-0.0505968
ENSG000001	TNFSF4	0.53554481	0.80413676	-0.0505729
ENSG000001	GP5	0.65694359	0.86514195	-0.0505645
ENSG000001	NELFA	0.42518025	0.73817463	-0.0505501
ENSG000001	EIF1	0.18821719	0.55223754	-0.0505309

ENSG000001	ZNF74	0.56900178	0.82037292	-0.0505207
ENSG000001	MFSD3	0.65941348	0.86611363	-0.0505063
ENSG000001	MYO1C	0.49352916	0.78107975	-0.050499
ENSG000001	PABPN1	0.35839583	0.69382497	-0.0504604
ENSG000001	NIPSNAP1	0.54979347	0.81217374	-0.0504382
ENSG000001	QRICH2	0.60681607	0.84023871	-0.0503661
ENSG000001	GPR132	0.3642514	0.6973647	-0.0503258
ENSG000001	TM7SF2	0.6194397	0.84711589	-0.0503048
ENSG000001	POLD2	0.58055385	0.82651932	-0.0502802
ENSG000001	GGT7	0.63544691	0.85504577	-0.0502748
ENSG000001	SYNPO	0.59744273	0.83640463	-0.0502514
ENSG000002	ATXN2	0.31554984	0.66324235	-0.0501983
ENSG000001	CTSB	0.31568	0.66326296	-0.0501366
ENSG000001	CYTH4	0.38414442	0.71245167	-0.0500984
ENSG00000C	SF3B2	0.33184669	0.67487409	-0.0500883
ENSG000001	OCIAD2	0.73874616	0.90351171	-0.050088
ENSG000001	PCNX2	0.65009199	0.86293893	-0.049994
ENSG00000C	ANKS1A	0.36078711	0.69535997	-0.049968
ENSG000001	H3-5	0.63321719	0.85444991	-0.0499528
ENSG000001	STX5	0.32304894	0.66903175	-0.0498285
ENSG000001	ZNF563	0.77671859	0.9172058	-0.0498202
ENSG000001	GRAMD2A	0.89712549	0.96518862	-0.0498196
ENSG000001	RGPD8	0.71532686	0.89186533	-0.0498133
ENSG000001	H4C3	0.91539686	0.97217816	-0.0498022
ENSG000001	SP2	0.39028267	0.71927554	-0.0497793
ENSG000001	AGMAT	0.68227522	0.87750056	-0.0497611
ENSG000001	POMGNT2	0.62387428	0.84972866	-0.0497563
ENSG000001	G0S2	0.80521692	0.92897486	-0.0497527
ENSG000001	ATP1B2	0.82245647	0.93539963	-0.0497062
ENSG000001	SOD2	0.69725824	0.88294449	-0.0496961
ENSG00000C	PPP2R5A	0.27365626	0.63403495	-0.0496712
ENSG000001	SLC16A1	0.60131778	0.83807698	-0.0496556
ENSG000001	RPS21	0.79803199	0.92603117	-0.0496306
ENSG000001	GLB1L	0.64093446	0.85737789	-0.0496285
ENSG000001	TXNRD2	0.51812292	0.79572786	-0.0494785
ENSG000001	FAM161B	0.5746573	0.82341424	-0.0494412
ENSG00000C	TRAPPC6A	0.60339088	0.83883394	-0.0493837
ENSG000001	NACA	0.58806059	0.83079611	-0.0493581
ENSG000001	ALG12	0.41979704	0.73466853	-0.0493509
ENSG000001	C20orf204	0.70780333	0.88890112	-0.0493191
ENSG000001	BIN2	0.07103941	0.40194888	-0.0493093
ENSG00000C	CASP8	0.30261601	0.65591444	-0.0492944
ENSG000001	SPG7	0.49140174	0.78031845	-0.0492922

ENSG000001	CCDC96	0.63294461	0.85440485	-0.0492901
ENSG000001	SLC47A2	0.88536133	0.96095764	-0.0492854
ENSG000001	MAPK14	0.51045575	0.79143652	-0.0492842
ENSG000001	EPHA4	0.70695986	0.88859787	-0.0492754
ENSG000001	TMBIM6	0.04468014	0.35527495	-0.0492585
ENSG000001	ZNF512B	0.55782979	0.81587881	-0.0492403
ENSG000001	NUP210L	0.790387	0.92280247	-0.0492248
ENSG000001	IL1RAP	0.6777465	0.8751149	-0.0492127
ENSG000001	SCN9A	0.68624801	0.87998223	-0.0491901
ENSG00000C	JMJD4	0.6157209	0.84553248	-0.0491736
ENSG000002	N/A	0.74300342	0.90596283	-0.0491639
ENSG00000C	NRDC	0.15194332	0.51296214	-0.0490591
ENSG000001	TRPM4	0.68972065	0.88074719	-0.0490457
ENSG000001	HABP4	0.67825475	0.87556228	-0.0490445
ENSG000001	MEI1	0.60069138	0.83785409	-0.0490407
ENSG000001	BET1L	0.3536257	0.69145236	-0.0490184
ENSG000002	VPS16	0.2765073	0.6360577	-0.0489976
ENSG00000C	OVGP1	0.76260468	0.91167139	-0.048967
ENSG000001	C1orf189	0.79901562	0.92603117	-0.0489652
ENSG000001	NAV1	0.68206302	0.87740907	-0.048965
ENSG00000C	XAB2	0.41691167	0.73289674	-0.0488967
ENSG000001	CLEC10A	0.55454048	0.81426804	-0.0488854
ENSG000001	FAM207A	0.54984448	0.81217374	-0.0488736
ENSG000001	SERINC3	0.29712258	0.65264233	-0.048796
ENSG00000C	PICALM	0.40221333	0.72669629	-0.048782
ENSG000001	FAM43A	0.55287609	0.81383402	-0.0487772
ENSG000001	ARL13A	0.70077155	0.88498263	-0.0487699
ENSG000001	ZC3H10	0.25930506	0.62303431	-0.0487369
ENSG000001	VPS39	0.05641785	0.3750908	-0.0487157
ENSG000001	CGREF1	0.91383073	0.97135384	-0.0487156
ENSG000001	TMEM129	0.46605273	0.76484555	-0.0487001
ENSG000001	GPAA1	0.54538314	0.80952117	-0.0486629
ENSG000001	MFAP5	0.91037754	0.9703801	-0.0486511
ENSG000001	HDGFL3	0.70498886	0.8872197	-0.0486423
ENSG000001	ROR1	0.92292318	0.97421357	-0.0486407
ENSG000001	STIL	0.72542247	0.89686038	-0.0486386
ENSG000001	RILPL2	0.25861333	0.6223087	-0.048612
ENSG000001	APPL2	0.49786253	0.78278241	-0.0485939
ENSG000001	MFSD5	0.43057911	0.74119757	-0.0485899
ENSG000001	NEK11	0.75071828	0.90816665	-0.0485044
ENSG000001	CAPNS1	0.40785563	0.72771726	-0.0484734
ENSG000001	SIRT3	0.51761173	0.79565102	-0.0484624
ENSG000001	TMEM39B	0.42572565	0.73827912	-0.0484526

ENSG000001	NMU	0.89619433	0.9647318	-0.0484433
ENSG000001	CCR8	0.82183693	0.93539963	-0.0484232
ENSG000001	MDM2	0.47796262	0.77199385	-0.0483585
ENSG000001	TRIM41	0.28550395	0.64333814	-0.0483518
ENSG000001	FOXO1	0.65791096	0.86540022	-0.0483035
ENSG000001	CES4A	0.58139599	0.82722887	-0.0482967
ENSG000001	SMIM43	0.92874244	0.97652064	-0.0482553
ENSG000001	ALDH8A1	0.73316798	0.89987515	-0.0481664
ENSG00000C	FRY	0.51153496	0.79176683	-0.0481545
ENSG000001	MOSPD2	0.59885922	0.83673325	-0.0481355
ENSG000001	H2AC11	0.6672529	0.8703555	-0.0481339
ENSG000001	GUCD1	0.22342533	0.59081204	-0.0480357
ENSG000001	TRIR	0.41565039	0.73222542	-0.0480301
ENSG000001	THYN1	0.54118077	0.80757124	-0.0480271
ENSG00000C	ATP2B4	0.6307637	0.85326916	-0.0480256
ENSG00000C	PDIA5	0.66802629	0.87080466	-0.0480157
ENSG000001	TMBIM1	0.13938708	0.49975817	-0.0479536
ENSG000001	HDDC3	0.62631206	0.85066154	-0.0479125
ENSG000001	FAM171A1	0.66911798	0.87125589	-0.0478958
ENSG000001	TMEM175	0.3934936	0.72063563	-0.0478956
ENSG000001	GLIPR1	0.65539276	0.8644689	-0.0478923
ENSG000001	NFKBIA	0.30945862	0.65916845	-0.0478492
ENSG000001	FOLR1	0.9139233	0.97135384	-0.0478269
ENSG00000C	IFNGR1	0.62075658	0.84820538	-0.0478076
ENSG000001	ABHD17A	0.61004236	0.84290213	-0.0478047
ENSG000001	AK8	0.8919947	0.96298656	-0.0477992
ENSG00000C	SETD1A	0.53006218	0.8016768	-0.0477417
ENSG000001	SMARCC2	0.32127194	0.66766831	-0.0477351
ENSG000001	MVD	0.60434067	0.83883394	-0.0476136
ENSG00000C	REXO1	0.45814224	0.75759807	-0.0475865
ENSG000001	ATP10B	0.92314671	0.97433263	-0.0475865
ENSG00000C	FBXL19	0.4670388	0.76514065	-0.0475852
ENSG000001	P4HTM	0.61063058	0.84331111	-0.0475838
ENSG000001	CTSK	0.66925928	0.87127337	-0.0475382
ENSG000001	MRTFA	0.5080476	0.79067965	-0.047532
ENSG000001	PEX5	0.47983049	0.77364477	-0.047514
ENSG000001	CITED1	0.86720515	0.9528412	-0.0474585
ENSG000001	AIP	0.52350657	0.79870477	-0.0474487
ENSG00000C	COL9A2	0.6593074	0.86611363	-0.0474449
ENSG000001	ATP5MC2	0.53026115	0.80188603	-0.0474204
ENSG000001	DPM3	0.63437399	0.85503542	-0.0474137
ENSG000001	CD2BP2	0.22642432	0.5937422	-0.0473851
ENSG00000C	CUL7	0.44134197	0.74775474	-0.0473654

ENSG00000(	HYAL2	0.53425106	0.80388793	-0.0472835
ENSG000001	H2AZ2	0.4656574	0.76449739	-0.0472813
ENSG000001	INPPL1	0.31159521	0.66022122	-0.0472806
ENSG00000(	PALM	0.84775877	0.94527257	-0.04726
ENSG00000(	KHSRP	0.37933779	0.70965485	-0.0472579
ENSG000001	MED12	0.45001558	0.75285025	-0.0471644
ENSG00000(	BICRA	0.52132486	0.79758332	-0.0471344
ENSG000001	TMC6	0.57069911	0.82112905	-0.0470954
ENSG000001	IFNAR1	0.45554312	0.75630405	-0.0470791
ENSG000002	TBC1D3	0.93460302	0.97859224	-0.047047
ENSG000001	RPL12	0.66266304	0.86788287	-0.0470273
ENSG000001	NPIPB15	0.78804845	0.92204735	-0.0470113
ENSG000001	TNIP2	0.2565801	0.62074989	-0.0470111
ENSG000001	CNTNAP1	0.52822293	0.80062313	-0.0470107
ENSG000001	PRXL2B	0.91892524	0.97272242	-0.0470077
ENSG000001	POLR3H	0.51206226	0.7921414	-0.046967
ENSG000001	RPIA	0.54524949	0.80952117	-0.0469641
ENSG000001	DVL1	0.55331858	0.81385767	-0.0469242
ENSG000001	EPHX1	0.56170742	0.81801019	-0.046919
ENSG000002	PHB2	0.53881824	0.8064433	-0.0469128
ENSG000001	SCAMP2	0.27656286	0.6360577	-0.0468933
ENSG000001	CSAD	0.4411421	0.74752915	-0.0468865
ENSG000001	S100A10	0.54832385	0.81144134	-0.0468841
ENSG000001	ADCY6	0.79871632	0.92603117	-0.04688
ENSG000001	TMCO6	0.41074353	0.72975628	-0.0468587
ENSG000001	USP32	0.45794304	0.7575144	-0.0468517
ENSG000001	ANKRD54	0.59667477	0.83600051	-0.0468117
ENSG000001	MANBA	0.32362567	0.66973108	-0.0467691
ENSG000001	TMEM234	0.37919903	0.70965485	-0.0467251
ENSG000002	STRADA	0.51069152	0.79143652	-0.046724
ENSG000002	ADAT3	0.6818497	0.87720415	-0.0467215
ENSG000001	RPL13A	0.73812797	0.90295968	-0.0466873
ENSG00000(	CYTH3	0.5688009	0.82037292	-0.0466701
ENSG000001	NR3C2	0.7120826	0.89093319	-0.0466691
ENSG000001	TUBA1C	0.46993565	0.7662622	-0.0466572
ENSG000001	TLN2	0.82007721	0.93468999	-0.0466496
ENSG000001	ANXA7	0.22699158	0.59448256	-0.0466055
ENSG00000(	OCEL1	0.54544856	0.80952117	-0.0465989
ENSG000001	TMUB1	0.55555872	0.8145753	-0.0465565
ENSG00000(	TNRC6C	0.6189546	0.84711589	-0.0465326
ENSG000001	GGA1	0.4185898	0.73381176	-0.0465201
ENSG000001	RPL7A	0.65307448	0.86386724	-0.046419
ENSG000001	CCND1	0.75019583	0.9080858	-0.0463617

ENSG00000(	CRKL	0.29996132	0.65445739	-0.0462956
ENSG000001	CLEC18B	0.73154974	0.89911234	-0.0462819
ENSG000001	BHLHE40	0.51832452	0.79572786	-0.0462778
ENSG000001	PAK2	0.45641839	0.75682857	-0.0462709
ENSG000001	RTP5	0.906078	0.96827466	-0.0462525
ENSG000001	BABAM2	0.44610995	0.75038716	-0.0462478
ENSG000001	MGMT	0.54754801	0.81118038	-0.0462413
ENSG000001	NDUFV1	0.56685952	0.8197023	-0.0462096
ENSG000001	PCNX3	0.41049021	0.72970545	-0.0461918
ENSG000001	CRYL1	0.5801809	0.82620569	-0.0461613
ENSG000001	PRPF40B	0.74942962	0.90782518	-0.0460771
ENSG000001	ERO1A	0.32805106	0.67237631	-0.0460556
ENSG000001	CMIP	0.54986919	0.81217374	-0.046039
ENSG000001	GHDC	0.49400532	0.7812211	-0.0460054
ENSG000001	RAPGEF2	0.58410368	0.82875175	-0.0459833
ENSG000001	PGM2	0.59464018	0.83499718	-0.0459739
ENSG000001	RAB5IF	0.63300922	0.85440485	-0.0459689
ENSG000001	PSME3	0.05712418	0.37665007	-0.045964
ENSG000001	POFUT2	0.37653179	0.70810131	-0.0459405
ENSG000001	LDHD	0.6979374	0.88341203	-0.0459247
ENSG000001	GUSB	0.42160316	0.73568731	-0.045917
ENSG000001	RPL23A	0.64788978	0.86149962	-0.045912
ENSG000001	RPL10A	0.71826176	0.89332947	-0.0458877
ENSG000001	SNRPE	0.74923616	0.90770972	-0.045847
ENSG00000(	LAMP2	0.4192791	0.73438543	-0.0458163
ENSG000001	CLNK	0.88493736	0.96095764	-0.0458044
ENSG000001	FXR2	0.3700284	0.70295438	-0.0457653
ENSG000001	ASPSCR1	0.54541465	0.80952117	-0.0457333
ENSG000001	LMNB2	0.53931519	0.80671218	-0.0457265
ENSG000001	HMGB2	0.724029	0.89610937	-0.0455873
ENSG000001	PTEN	0.50161038	0.7853582	-0.0455496
ENSG00000(	TMED1	0.53309287	0.80294166	-0.0455034
ENSG000001	CACUL1	0.41105937	0.72995333	-0.0454014
ENSG000001	RAB20	0.70722083	0.88878828	-0.0453985
ENSG000001	RPS16	0.72512361	0.89678056	-0.0453696
ENSG000001	SYVN1	0.46648411	0.76486809	-0.0453657
ENSG000001	H1-10	0.64054487	0.85713919	-0.0453649
ENSG000001	DIAPH1	0.56844896	0.82037292	-0.0453195
ENSG000001	PPIL6	0.80571729	0.92941402	-0.0453175
ENSG000001	PSAP	0.41140697	0.72999304	-0.0452848
ENSG000002	TAPBP	0.76287979	0.91167139	-0.045256
ENSG000001	RBM10	0.30658411	0.65735608	-0.0452153
ENSG000002	SRA1	0.31314817	0.6613866	-0.0451981

ENSG000001	DUS1L	0.58430806	0.82881191	-0.0451786
ENSG000001	FAM83G	0.65260005	0.86379349	-0.0451698
ENSG000001	NELFB	0.49337224	0.78107975	-0.0451477
ENSG000001	CD3D	0.7732718	0.91571183	-0.0451173
ENSG000001	CDK10	0.60661299	0.84018575	-0.0450535
ENSG000001	CHST14	0.54128212	0.80757124	-0.0450209
ENSG000001	USP22	0.36280583	0.69656832	-0.0450088
ENSG000001	LMAN2	0.31222737	0.66088329	-0.0449912
ENSG000001	SCRN2	0.62490098	0.85016376	-0.0449516
ENSG000001	PTPN7	0.61884169	0.84711589	-0.0449276
ENSG000002	NUDT3	0.66069812	0.86709953	-0.04491
ENSG000001	ULK3	0.63369874	0.85461553	-0.0448587
ENSG00000C	JARID2	0.36351311	0.69694698	-0.0448513
ENSG000001	CHKB	0.63639645	0.85560729	-0.0448492
ENSG000001	PHF2	0.41726756	0.73321077	-0.0448412
ENSG000001	ZBTB7A	0.38390308	0.7123445	-0.0448184
ENSG000001	CTCF	0.31693869	0.66422665	-0.0448144
ENSG000001	GPS1	0.46168777	0.76122418	-0.0447907
ENSG000001	MNDA	0.50269391	0.78619158	-0.0447336
ENSG000001	ZNF629	0.47796398	0.77199385	-0.0446909
ENSG000001	UCK1	0.25670846	0.62074989	-0.044678
ENSG000001	YIF1A	0.57929036	0.82595167	-0.0446442
ENSG000001	CD37	0.33628016	0.67811486	-0.044614
ENSG000001	NOTCH2	0.53472464	0.80406779	-0.0446
ENSG000001	LSM14B	0.363879	0.69706268	-0.044555
ENSG00000C	ZZEF1	0.4127707	0.73063968	-0.0445314
ENSG000001	RITA1	0.55480777	0.81433077	-0.0445055
ENSG000002	CFAP57	0.8997491	0.96608561	-0.0444999
ENSG000001	CRTAP	0.56484562	0.81898782	-0.0444935
ENSG000001	TMEM97	0.73934366	0.9039701	-0.0444807
ENSG000001	ACCS	0.61711925	0.84630481	-0.0444772
ENSG000001	PSMC1	0.29702931	0.65262041	-0.0444751
ENSG000001	H2BU1	0.83050022	0.93748833	-0.0444407
ENSG00000C	ACACB	0.62864164	0.85212646	-0.044431
ENSG000001	CLASRP	0.37808051	0.70909881	-0.044423
ENSG00000C	EVI5	0.48126821	0.77439848	-0.0444055
ENSG000001	PGAM1	0.23067471	0.59855451	-0.0443966
ENSG000001	RS1	0.90392571	0.96801489	-0.0443637
ENSG000001	IRAG2	0.5021806	0.78569151	-0.0443278
ENSG00000C	MCM6	0.66176708	0.86736018	-0.044311
ENSG000001	C2orf68	0.2801031	0.6389512	-0.0442877
ENSG000001	NAA38	0.63941257	0.85691647	-0.0442669
ENSG00000C	IL32	0.69498042	0.88222003	-0.0442452

ENSG000001	RPL18A	0.67565016	0.87451032	-0.0442098
ENSG000002	MARCKS	0.57284343	0.82223748	-0.0441788
ENSG000000	PCBP4	0.7029374	0.88595232	-0.044144
ENSG000001	STX4	0.18080054	0.54567047	-0.0441156
ENSG000000	WDR18	0.59825569	0.83653786	-0.0441101
ENSG000001	ZNF705A	0.92282622	0.97418434	-0.0440299
ENSG000002	GPR52	0.91972493	0.97312633	-0.044021
ENSG000001	SAFB	0.29522957	0.6512539	-0.0440181
ENSG000001	UFC1	0.58535077	0.82942145	-0.0439311
ENSG000001	TMEM201	0.59237975	0.83392318	-0.0438819
ENSG000001	IMMP2L	0.78866633	0.92204735	-0.0438308
ENSG000001	BCL6	0.6872353	0.88042583	-0.0437691
ENSG000001	SLA	0.44318416	0.74887084	-0.0437456
ENSG000001	ALDH9A1	0.36022542	0.69531385	-0.0437178
ENSG000001	PCED1A	0.6309788	0.85326916	-0.0436953
ENSG000001	CDKN2C	0.59786075	0.83648988	-0.0436924
ENSG000001	RSPH3	0.40739017	0.72768322	-0.0436679
ENSG000001	LGALS4	0.85417556	0.94814417	-0.0436558
ENSG000002	N/A	0.93034762	0.97723213	-0.043646
ENSG000001	ANAPC2	0.52245628	0.7979767	-0.0436303
ENSG000001	UNC5A	0.79052876	0.92280247	-0.0436033
ENSG000001	TRAPPC9	0.51602982	0.7950982	-0.0435549
ENSG000001	TSPAN14	0.24755024	0.61366004	-0.0434705
ENSG000001	MZB1	0.84009103	0.94245133	-0.0434582
ENSG000000	GSTP1	0.5360944	0.80424764	-0.0434193
ENSG000001	NDUFS2	0.29322062	0.64988031	-0.0433985
ENSG000001	DCTN2	0.33473205	0.67675278	-0.0433778
ENSG000001	ATOH8	0.78250403	0.9193471	-0.0433517
ENSG000001	NSMCE1	0.61391792	0.8451435	-0.0433507
ENSG000001	ARPC3	0.38899732	0.71798902	-0.0433193
ENSG000001	LDLRAD4	0.71762224	0.89321665	-0.0433096
ENSG000000	MCAM	0.80136329	0.92735626	-0.0432907
ENSG000001	DUSP23	0.54187285	0.80808176	-0.0432509
ENSG000001	FBXW9	0.76113776	0.91141904	-0.0432493
ENSG000000	ST3GAL1	0.198935	0.56598606	-0.0432473
ENSG000000	ITGAE	0.65622233	0.86486055	-0.0432317
ENSG000001	DUSP18	0.33080774	0.67424323	-0.0432108
ENSG000001	P2RY13	0.47839058	0.77232426	-0.0432014
ENSG000001	NET1	0.68897617	0.88065072	-0.0431497
ENSG000001	MLST8	0.60683248	0.84023871	-0.043058
ENSG000001	HECTD3	0.46839556	0.76581802	-0.0430415
ENSG000001	MTA1	0.62620267	0.85066154	-0.0429671
ENSG000001	LGALS1	0.67119465	0.87234352	-0.0429215

ENSG000001	PRR15L	0.8888748	0.96164016	-0.042908
ENSG000001	STK32C	0.56155689	0.81801019	-0.0429028
ENSG000001	RHAG	0.85012118	0.94644505	-0.0428982
ENSG000001	DDX28	0.52198097	0.79776689	-0.0428537
ENSG000001	CUEDC2	0.42419789	0.73732623	-0.0428185
ENSG000001	LRRC57	0.53463696	0.80406779	-0.0428011
ENSG000001	MCRS1	0.41614439	0.73237984	-0.0427339
ENSG000001	LMF2	0.5013569	0.7853582	-0.0426814
ENSG000001	TUFM	0.49296627	0.78060205	-0.0426465
ENSG000001	NRBP1	0.23972954	0.60643234	-0.0426433
ENSG000002	N/A	0.85176846	0.94720831	-0.0426364
ENSG000001	UQCC2	0.65240006	0.86379349	-0.0426364
ENSG000001	YPEL4	0.68366661	0.87849752	-0.0426241
ENSG000001	HOXA3	0.81341398	0.93183372	-0.0425938
ENSG000001	ERGIC3	0.54256899	0.80822869	-0.0425721
ENSG000001	EOLA2	0.54802923	0.81141647	-0.0425697
ENSG000001	PFAS	0.59668719	0.83600051	-0.0425133
ENSG00000C	NDUFB7	0.57781665	0.82508213	-0.04247
ENSG000001	BAG3	0.6756645	0.87451032	-0.0424688
ENSG000001	ZNF106	0.44322527	0.74887084	-0.0424496
ENSG000001	CA14	0.76913108	0.91376172	-0.0424217
ENSG000002	GATD3B	0.66818335	0.87090078	-0.0424128
ENSG000001	ARPC2	0.34419916	0.68479845	-0.0424037
ENSG000001	SPRED2	0.6581668	0.8654563	-0.0423853
ENSG000001	CLTC	0.40935038	0.72902686	-0.0423628
ENSG00000C	MTA3	0.66755632	0.8706114	-0.042359
ENSG000002	STX16-NPEPL	0.64550215	0.8600572	-0.0423447
ENSG000001	RAD23B	0.32192031	0.66822528	-0.0423238
ENSG00000C	CFAP20	0.46483465	0.76408722	-0.0423177
ENSG000001	VAMP1	0.62283832	0.84927918	-0.0422986
ENSG000001	GIT1	0.48479829	0.7769018	-0.0422694
ENSG00000C	PPP1R3F	0.69378678	0.88200877	-0.0420646
ENSG000001	CYBC1	0.35610179	0.69237934	-0.0420514
ENSG000001	MAPKBP1	0.54993234	0.81218902	-0.0419971
ENSG000001	LRTOMT	0.62764535	0.8511716	-0.0419356
ENSG000001	CSK	0.48704163	0.77767904	-0.041883
ENSG000001	SNRNP35	0.36916813	0.70197716	-0.0418715
ENSG000001	GRIN2A	0.91554419	0.97224705	-0.0418468
ENSG00000C	MAD1L1	0.61523267	0.84533626	-0.0417915
ENSG00000C	PAFAH1B3	0.67634797	0.87463061	-0.0417673
ENSG000001	H2AC6	0.51008758	0.79143652	-0.0417128
ENSG000001	C2orf88	0.69079473	0.88107274	-0.0416837
ENSG000001	FOXRED1	0.58416356	0.82875175	-0.0416733

ENSG000001	ADAM15	0.56678372	0.8197023	-0.0416629
ENSG000001	IGSF3	0.93509406	0.97860923	-0.0416443
ENSG000001	PLBD2	0.49195912	0.7804495	-0.0416192
ENSG000001	THEMIS2	0.54899136	0.81177567	-0.0415962
ENSG00000C	IFT88	0.6991818	0.8842289	-0.0415175
ENSG000002	CRCP	0.36229103	0.69632095	-0.0414906
ENSG000001	DDIT4L	0.91806901	0.97272242	-0.0414369
ENSG000002	CDPF1	0.58923067	0.8317978	-0.0414087
ENSG00000C	RELT	0.40601293	0.72744229	-0.0413775
ENSG000001	LTBR	0.44369319	0.74908353	-0.0413746
ENSG000001	REEP4	0.52410422	0.79870477	-0.041373
ENSG000001	SHISAL2A	0.75161385	0.908573	-0.0413282
ENSG000001	SF3B4	0.51706415	0.79538014	-0.0412451
ENSG000002	CCDC163	0.74366401	0.906159	-0.041115
ENSG000001	MECP2	0.44695497	0.75099423	-0.041112
ENSG000002	C2orf74	0.87013296	0.95423131	-0.0410777
ENSG00000C	RETSAT	0.5256678	0.79977109	-0.0410415
ENSG00000C	MAP3K14	0.41545833	0.73221244	-0.0410322
ENSG00000C	VCAN	0.59820352	0.83653786	-0.0410283
ENSG000001	NOP2	0.74162734	0.90543757	-0.0410193
ENSG000001	C17orf97	0.79593687	0.9246397	-0.041017
ENSG000001	JOSD2	0.54669575	0.81063154	-0.0409973
ENSG000001	SYF2	0.31467268	0.66270362	-0.0409693
ENSG000001	PPFIA1	0.47356188	0.76895276	-0.0409175
ENSG000001	EIF3F	0.60933344	0.84246798	-0.0409111
ENSG000001	MYORG	0.70542746	0.88749618	-0.0408651
ENSG000001	LMF1	0.67666473	0.87490088	-0.0408267
ENSG000001	PRKAR2A	0.52399694	0.79870477	-0.0408204
ENSG000001	POLR2L	0.58159943	0.82722887	-0.0407855
ENSG000001	TK2	0.3331789	0.67555268	-0.0407216
ENSG000001	CHKA	0.56962223	0.82045207	-0.0406931
ENSG000001	RPS13	0.77026474	0.91423988	-0.0406374
ENSG000002	N/A	0.72487456	0.89669145	-0.0406127
ENSG000001	UBE2H	0.31096453	0.65949478	-0.0406107
ENSG000001	UBQLN4	0.57036036	0.82085989	-0.0405966
ENSG000002	ZNF783	0.60391665	0.83883394	-0.0405909
ENSG00000C	CD44	0.43782741	0.74549116	-0.0405546
ENSG000001	ARFRP1	0.57363711	0.8225864	-0.040512
ENSG000001	HPS6	0.46978027	0.7662622	-0.0405062
ENSG000001	UCP2	0.53535054	0.80413676	-0.0404194
ENSG000001	ABCF3	0.4340677	0.74353384	-0.0404072
ENSG000001	UNC13B	0.74090917	0.90513447	-0.0403613
ENSG00000C	EPDR1	0.73052788	0.89847521	-0.0403611

ENSG00000(	NCAPD2	0.4367825	0.74496191	-0.0403568
ENSG000001	ZBTB46	0.72768119	0.8971681	-0.0403161
ENSG00000(	ALKBH5	0.45499438	0.7559601	-0.0403076
ENSG000001	SLC31A1	0.42693242	0.7392501	-0.0403042
ENSG000001	C1orf74	0.68480889	0.87924442	-0.0402906
ENSG000001	SIGMAR1	0.58449461	0.82887597	-0.0402875
ENSG000002	KRT222	0.90563994	0.96827466	-0.0402548
ENSG00000(	PHF23	0.30673956	0.65749363	-0.0402114
ENSG00000(	POLRMT	0.64376813	0.85940463	-0.0401897
ENSG000001	CHID1	0.56976341	0.8205285	-0.0401678
ENSG000001	GLIPR2	0.55362058	0.8139043	-0.040145
ENSG000001	COA3	0.62309415	0.84930015	-0.0401398
ENSG000002	ARL16	0.57812606	0.82523365	-0.0401351
ENSG000001	EIF4EBP1	0.57477225	0.82341424	-0.0400942
ENSG000001	NPM3	0.742849	0.90586851	-0.0400884
ENSG000001	DAD1	0.51696414	0.79538014	-0.0400741
ENSG000001	BMP1	0.66392573	0.86837979	-0.0400509
ENSG000001	PRRT2	0.65480231	0.86439228	-0.0399824
ENSG000001	GPR153	0.75833344	0.9110527	-0.0399252
ENSG000002	HYPK	0.70392772	0.88657108	-0.0399145
ENSG000001	MCM4	0.72160534	0.89490971	-0.0398665
ENSG000001	SLC3A1	0.90312549	0.96763453	-0.0398081
ENSG000002	TCTN1	0.67586823	0.87451032	-0.0397529
ENSG00000(	SLC25A3	0.4923201	0.7804495	-0.039746
ENSG000001	C19orf71	0.65498131	0.86442301	-0.0397206
ENSG000001	UBE2B	0.46687086	0.7650199	-0.0396811
ENSG000002	TNFSF12	0.52457435	0.79886642	-0.0396568
ENSG000001	RTL8C	0.59774149	0.83641817	-0.0396435
ENSG00000(	TG	0.74037734	0.90475705	-0.0396174
ENSG000001	NEIL2	0.66201852	0.86736018	-0.039617
ENSG000001	IFT81	0.82599165	0.93591669	-0.0396095
ENSG000001	MEDAG	0.9243532	0.97507159	-0.0396057
ENSG000001	MGAT1	0.37156084	0.70378624	-0.0395213
ENSG00000(	TCOF1	0.5648731	0.81898782	-0.0394682
ENSG000001	CA2	0.72564014	0.89686038	-0.0394537
ENSG000001	PI4KB	0.16431917	0.52769983	-0.0394305
ENSG000001	KCNS2	0.93810931	0.97949205	-0.0393496
ENSG000001	PHETA1	0.65066252	0.86327891	-0.0393375
ENSG000001	GRK5	0.38262111	0.71172172	-0.0393101
ENSG000002	N/A	0.93501756	0.97859224	-0.0392866
ENSG000001	ITGA9	0.85143828	0.94709895	-0.0392616
ENSG000001	HIP1R	0.65667466	0.86503541	-0.0392592
ENSG000001	CHMP6	0.51034895	0.79143652	-0.0392487

ENSG000001	ALOX12	0.43144941	0.74126857	-0.0392188
ENSG00000C	ERCC1	0.55064148	0.81264913	-0.0392086
ENSG000001	CA8	0.86852801	0.95343718	-0.0392069
ENSG000001	FAM184A	0.81150004	0.93158415	-0.0392006
ENSG00000C	ALAS1	0.39120355	0.71964297	-0.0391996
ENSG000001	EIF3B	0.43197225	0.7414306	-0.0391965
ENSG00000C	EDC4	0.49144155	0.78031845	-0.0391755
ENSG000001	SMG7	0.19933147	0.56600138	-0.0391315
ENSG000001	OLFML2B	0.78599247	0.92105345	-0.0391064
ENSG000001	USP19	0.42250551	0.73639137	-0.0390936
ENSG00000C	RCOR1	0.546289	0.8104984	-0.0390835
ENSG000001	LRRC14	0.58618341	0.82959975	-0.0390707
ENSG000001	SLC40A1	0.59533571	0.83554896	-0.0390524
ENSG00000C	ACOX3	0.39695621	0.72308765	-0.0390502
ENSG000001	ANAPC11	0.56203926	0.81804833	-0.0390327
ENSG000001	MAFK	0.49825755	0.7829486	-0.0390065
ENSG000001	PASK	0.76517234	0.91260342	-0.0389871
ENSG000001	NFRKB	0.45536494	0.75616287	-0.0389834
ENSG000001	TMEM116	0.75709103	0.91082077	-0.0389549
ENSG000001	KMT2D	0.57535422	0.82344878	-0.0389513
ENSG000001	CTC1	0.63844584	0.85644788	-0.0389342
ENSG000001	MAU2	0.26228296	0.62579547	-0.038918
ENSG000001	WDFY3	0.72719662	0.89708846	-0.0388678
ENSG000001	NIT1	0.29976314	0.6542516	-0.0388526
ENSG000001	PPM1G	0.44982263	0.75279291	-0.0388481
ENSG000001	DHX40	0.55372148	0.8139043	-0.0388328
ENSG00000C	YIPF1	0.36105863	0.69535997	-0.0388255
ENSG000001	TNFSF8	0.57440629	0.82330481	-0.0387959
ENSG000001	MECR	0.74478684	0.90628646	-0.0387439
ENSG00000C	GINM1	0.48127456	0.77439848	-0.0387282
ENSG000001	RELA	0.45246219	0.75419655	-0.0386894
ENSG000001	BICDL1	0.73244166	0.89954428	-0.0386505
ENSG000001	LMBRD1	0.65787617	0.86540022	-0.038563
ENSG000001	INPP5E	0.69999447	0.88452288	-0.0385024
ENSG000001	BTBD11	0.56847609	0.82037292	-0.0384382
ENSG000001	NDUFA2	0.52600264	0.80004441	-0.0384303
ENSG000001	DYNC2I2	0.74225432	0.90555138	-0.0383443
ENSG000001	PRCP	0.33998386	0.68135285	-0.0383248
ENSG000001	FHOD3	0.92514752	0.97521605	-0.0383042
ENSG000001	DMGDH	0.92798179	0.97652064	-0.0382991
ENSG000001	ALAD	0.51801046	0.79566132	-0.0382953
ENSG000001	GAB3	0.39129548	0.71964297	-0.0382915
ENSG000001	RAP1GAP2	0.29234568	0.64947398	-0.038287

ENSG000001	TMEM266	0.72530874	0.89686038	-0.038254
ENSG000001	DDX27	0.40500968	0.72672584	-0.0382212
ENSG000001	CORIN	0.83253729	0.93841777	-0.0382127
ENSG000001	SGSM3	0.73578606	0.90152115	-0.0381823
ENSG000001	HTATIP2	0.35701762	0.69298434	-0.0381777
ENSG000001	HK3	0.55161628	0.81323014	-0.0381621
ENSG000001	ZNF174	0.58957236	0.83191276	-0.0381602
ENSG000001	GLB1	0.34601183	0.6858147	-0.0381497
ENSG000001	MARS1	0.17647126	0.54126676	-0.0381101
ENSG000001	FKRP	0.4405924	0.74745573	-0.0380919
ENSG000001	MTERF2	0.773863	0.91586084	-0.0380918
ENSG000001	WRAP53	0.59311013	0.83444495	-0.0380895
ENSG000002	PINLYP	0.86710873	0.9528412	-0.0380625
ENSG000001	MYO1D	0.67320454	0.87354948	-0.0380294
ENSG000001	STX3	0.61791278	0.84662697	-0.0380218
ENSG000001	GTPBP3	0.70740861	0.88887192	-0.0379865
ENSG000001	ZNF517	0.67225877	0.87323725	-0.0379814
ENSG000001	CCDC85B	0.6990249	0.88418481	-0.0379608
ENSG00000(	NLRC4	0.54432965	0.80906998	-0.0379475
ENSG000001	ZC3H13	0.44964345	0.75274366	-0.0379339
ENSG000001	ECI1	0.64811282	0.86158867	-0.0379217
ENSG00000(	FLT3LG	0.78158822	0.91907937	-0.0379206
ENSG000001	DENND4B	0.25526028	0.62026352	-0.0379086
ENSG000001	IK	0.20551671	0.56915826	-0.0379034
ENSG00000(	GNB1	0.23495528	0.60232491	-0.0378788
ENSG000001	DPH7	0.65695167	0.86514195	-0.0378635
ENSG000001	ETFB	0.5527694	0.81383402	-0.0378556
ENSG000001	ESRRA	0.44311889	0.74887084	-0.0378436
ENSG000001	ERAL1	0.51440055	0.79409791	-0.0377982
ENSG000001	DENND5A	0.49057977	0.7800506	-0.0377895
ENSG000001	EMC4	0.55320455	0.81385767	-0.0377444
ENSG000002	CEP43	0.73277937	0.89967021	-0.0377245
ENSG000001	PORCN	0.71611124	0.89229516	-0.0377126
ENSG000001	GLMP	0.58585452	0.82959975	-0.0376668
ENSG000002	ACBD6	0.66279022	0.86788287	-0.0375807
ENSG000001	SEMA4F	0.76063804	0.91141904	-0.0375797
ENSG000001	LRRC3	0.7134944	0.89149739	-0.0375438
ENSG00000(	APBA3	0.54726208	0.81118038	-0.0374933
ENSG000001	CCDC69	0.24991843	0.61582699	-0.0374842
ENSG000001	C19orf54	0.51220623	0.7921414	-0.0374813
ENSG000001	RPL29	0.72053366	0.89471589	-0.0374338
ENSG000001	RALGAPA2	0.42926258	0.74085209	-0.0374266
ENSG000001	SORCS3	0.88176358	0.95932126	-0.0374094

ENSG00000(	C3orf18	0.76626203	0.91309236	-0.0374042
ENSG00000(	AOC1	0.87611677	0.95676199	-0.0373668
ENSG00000(	HSD3B7	0.82270201	0.93539963	-0.0373251
ENSG000001	ZNF641	0.34534906	0.68522517	-0.0373227
ENSG00000(	WWTR1	0.84760856	0.94527257	-0.0372948
ENSG000001	POLR2C	0.37517306	0.70720386	-0.0372005
ENSG000001	KDM5C	0.36144545	0.69576643	-0.0371916
ENSG000001	PPM1E	0.91829201	0.97272242	-0.0371238
ENSG000001	CCDC61	0.64938429	0.86252724	-0.0370675
ENSG00000(	RPS5	0.75973111	0.91136366	-0.0369966
ENSG000001	SLC39A3	0.65949235	0.86612102	-0.0369919
ENSG000002	COG8	0.53895303	0.80646763	-0.0369321
ENSG000001	FBXL16	0.77384023	0.91586084	-0.036917
ENSG000001	VPS11	0.43105166	0.74126857	-0.0369145
ENSG000002	AJM1	0.77229251	0.91517395	-0.036913
ENSG000001	LCP1	0.51043492	0.79143652	-0.0369045
ENSG000001	GLA	0.45163041	0.75381609	-0.0368968
ENSG00000(	CFLAR	0.48762409	0.77796645	-0.0368947
ENSG000001	ZNF16	0.68565203	0.87981649	-0.0368439
ENSG000001	ROMO1	0.77277153	0.91554114	-0.0368306
ENSG000001	FBXO31	0.63358759	0.85459721	-0.0367961
ENSG00000(	SPG21	0.29541769	0.65128269	-0.0367532
ENSG000001	THEM6	0.6729716	0.87354948	-0.0367321
ENSG000001	TRIM11	0.4307881	0.74126857	-0.036719
ENSG000001	BEND5	0.745254	0.90628646	-0.0366977
ENSG000001	SERGEF	0.68106945	0.87679749	-0.0366855
ENSG000001	ZNF423	0.91330679	0.97135384	-0.036661
ENSG000001	DCTN3	0.51185236	0.79197129	-0.0366606
ENSG00000(	IGSF9B	0.80634724	0.92964486	-0.0366572
ENSG000001	ST14	0.64185875	0.85790751	-0.036633
ENSG00000(	KIZ	0.66193347	0.86736018	-0.0366107
ENSG000001	FZD8	0.92390595	0.9747538	-0.0366038
ENSG000001	ECM1	0.82301512	0.93539963	-0.0365922
ENSG000001	BCAS3	0.40213495	0.72669629	-0.0365719
ENSG00000(	FKBP1A	0.39421918	0.72144863	-0.0365647
ENSG000001	NHLRC1	0.83081303	0.93764588	-0.0365146
ENSG000002	FAM209B	0.76035693	0.91141904	-0.0364648
ENSG000001	PIP4K2A	0.493749	0.78107975	-0.0364369
ENSG000002	C8orf58	0.59590973	0.83576612	-0.036333
ENSG00000(	MAST2	0.5423038	0.80815796	-0.0363199
ENSG000001	FCSK	0.632811	0.85440485	-0.0362973
ENSG000001	PRPSAP1	0.65352547	0.8639711	-0.0362836
ENSG000001	ZMYND19	0.72105382	0.89490971	-0.036258

ENSG000001	PRKRIP1	0.54018055	0.80711435	-0.036102
ENSG000002	PPP1R2B	0.90366172	0.96790491	-0.0360684
ENSG000001	PDAP1	0.34370311	0.68437426	-0.0360645
ENSG000002	ZNF844	0.69491197	0.88222003	-0.036003
ENSG000001	MAP3K2	0.74160801	0.90543757	-0.0359977
ENSG00000(	TSG101	0.29995165	0.65445739	-0.0359548
ENSG000001	IFT43	0.73759879	0.90258434	-0.0358548
ENSG00000(	ZNF586	0.52169839	0.79764995	-0.0358288
ENSG000002	TMSB4X	0.56775006	0.82037292	-0.0357942
ENSG000002	C6orf226	0.75299609	0.90909343	-0.0357406
ENSG000001	ASB6	0.47759327	0.77191738	-0.0357339
ENSG000001	SLC13A5	0.88627737	0.96095764	-0.035676
ENSG000001	PLCG1	0.70514765	0.88728165	-0.035669
ENSG000002	COA8	0.62287065	0.84927918	-0.035659
ENSG000001	MID1	0.93544085	0.97876865	-0.0356435
ENSG00000(	AP3M2	0.70560868	0.88765539	-0.0356349
ENSG00000(	MGST2	0.68651658	0.88020923	-0.0355961
ENSG00000(	TBC1D1	0.54247174	0.80815796	-0.035578
ENSG000001	PGLYRP2	0.86525539	0.95237042	-0.0355747
ENSG000002	PJVK	0.81635275	0.93267095	-0.0355723
ENSG000001	ASB16	0.70481785	0.88716768	-0.0355622
ENSG000001	CTSV	0.93877244	0.97961749	-0.0355547
ENSG000001	MYOM1	0.63717374	0.85602759	-0.0354704
ENSG000002	ARPC1A	0.49988486	0.78433741	-0.0354663
ENSG000001	NAIF1	0.46397127	0.7634274	-0.0354604
ENSG000001	UBR4	0.50938718	0.79136944	-0.0354203
ENSG000001	NUMA1	0.59524396	0.83552629	-0.0353633
ENSG000001	DIRAS3	0.93548751	0.97876865	-0.0353039
ENSG000001	OXA1L	0.55676926	0.81546657	-0.0352993
ENSG000001	RIT1	0.61477966	0.84533626	-0.0352772
ENSG00000(	CLEC16A	0.40981792	0.72922889	-0.0352462
ENSG000001	TGFB1	0.6437133	0.85940463	-0.0352295
ENSG000002	N/A	0.79041879	0.92280247	-0.0351973
ENSG000002	HNRNPA1P4ٕ	0.78609101	0.92105345	-0.0351604
ENSG000001	OIP5	0.85551709	0.9486844	-0.0350674
ENSG000001	NSDHL	0.58006126	0.82610777	-0.0350374
ENSG000001	COPS6	0.53142614	0.80232066	-0.0350238
ENSG000001	SPECC1L	0.58477769	0.82904352	-0.0350068
ENSG000001	IL24	0.77430622	0.9161561	-0.0350031
ENSG000001	TKFC	0.63311817	0.85444991	-0.0349838
ENSG000001	MORC1	0.9461183	0.98315624	-0.0349551
ENSG000001	ZNF622	0.45460727	0.75583231	-0.0349112
ENSG000002	MC1R	0.70753768	0.88890112	-0.0349073

ENSG00000C	CROCC	0.70875417	0.88947345	-0.0348652
ENSG000001	TRUB2	0.64811504	0.86158867	-0.0348629
ENSG000001	TMEM214	0.54064461	0.80733448	-0.034813
ENSG000001	MYO5A	0.65403226	0.86439091	-0.0348016
ENSG00000C	HEXB	0.57223607	0.82182269	-0.0347952
ENSG000001	CINP	0.3778611	0.70909881	-0.0347501
ENSG000001	TMEM120B	0.68276268	0.87781049	-0.0347321
ENSG00000C	TTC22	0.67587793	0.87451032	-0.0347277
ENSG000001	ASAH1	0.5862356	0.82959975	-0.0347087
ENSG000001	RBM23	0.44536648	0.74952482	-0.0347009
ENSG000001	GOLGA7	0.53597216	0.80423066	-0.0346929
ENSG000001	HCST	0.66216486	0.86741192	-0.0346589
ENSG000002	MRPL12	0.74411466	0.90628646	-0.0346266
ENSG000001	ARMCX1	0.70407004	0.88665789	-0.0346207
ENSG000001	STX6	0.43512374	0.74433605	-0.0345171
ENSG000001	ARHGDIA	0.59476476	0.83503635	-0.0344803
ENSG000001	FITM1	0.78364753	0.91983554	-0.0344776
ENSG000001	RPS26	0.76758274	0.91352929	-0.0344738
ENSG000001	DHTKD1	0.43354875	0.74303684	-0.0344244
ENSG000001	SIPA1L1	0.78157227	0.91907937	-0.0343759
ENSG00000C	H6PD	0.4736478	0.76895276	-0.0343527
ENSG000001	USP35	0.5876242	0.83046863	-0.0343422
ENSG000001	TSC1	0.64111289	0.8575293	-0.0343033
ENSG000001	UCKL1	0.64601548	0.86041779	-0.0342943
ENSG000001	RAD9B	0.79887669	0.92603117	-0.0342926
ENSG000001	TTLL11	0.69020881	0.88074719	-0.0342651
ENSG00000C	RPUSD1	0.65804963	0.8654563	-0.0342386
ENSG000002	RTL10	0.67731382	0.87510133	-0.0342322
ENSG000001	IDNK	0.71395076	0.89153258	-0.0341865
ENSG00000C	PTPN18	0.47008029	0.7662622	-0.0340468
ENSG000001	PCYOX1	0.7338383	0.90042672	-0.0339545
ENSG000001	RPL3	0.80095528	0.92721452	-0.0338816
ENSG000001	POU2F1	0.5000472	0.78433741	-0.0338792
ENSG000002	RBM15B	0.50357732	0.78673932	-0.0338558
ENSG000001	MARK1	0.94159531	0.98086117	-0.0338532
ENSG000001	FBP1	0.64918586	0.86252724	-0.0338501
ENSG000001	BBS5	0.84293642	0.94328484	-0.0338303
ENSG000001	ARF3	0.51136997	0.79175206	-0.033797
ENSG000001	DDX51	0.66842731	0.8710625	-0.0337916
ENSG00000C	CWF19L1	0.51462724	0.79420539	-0.0337887
ENSG000001	PPP1R7	0.5482071	0.81141647	-0.0337762
ENSG000001	HDHD3	0.66931727	0.87127337	-0.0337085
ENSG00000C	PIK3CB	0.47379449	0.76896358	-0.033704

ENSG000001	NPPA	0.85474465	0.9482433	-0.0336716
ENSG00000(	EIF4B	0.74618781	0.90653584	-0.0336648
ENSG000001	NEFH	0.68580058	0.87991655	-0.0336632
ENSG000001	TIMM17B	0.57571495	0.82352953	-0.0336202
ENSG000001	TSSK6	0.70769782	0.88890112	-0.0336057
ENSG000001	PSTK	0.81335856	0.93183372	-0.0336033
ENSG000001	TRMO	0.68543428	0.87972633	-0.0335874
ENSG000001	DPH1	0.75714794	0.91082077	-0.0335223
ENSG000001	GPX7	0.78667406	0.92119044	-0.0334994
ENSG000002	CCL5	0.84759921	0.94527257	-0.0334994
ENSG000001	SHF	0.82596559	0.93591669	-0.0334609
ENSG000001	RER1	0.36569302	0.69837111	-0.0334544
ENSG000001	UBE2G1	0.45883877	0.75828652	-0.0333881
ENSG000001	ENKUR	0.77763292	0.91771115	-0.0333233
ENSG000001	SLAMF9	0.95117332	0.98403516	-0.0333001
ENSG000002	NAA80	0.75279396	0.90891698	-0.0332752
ENSG000001	CD63	0.54964425	0.81217374	-0.0332418
ENSG000001	KLF3	0.69021997	0.88074719	-0.0332373
ENSG000001	TMEM236	0.8647413	0.95224252	-0.0332081
ENSG000001	ABHD11	0.63038475	0.8529631	-0.0331649
ENSG000001	KMT5B	0.56088892	0.81751826	-0.0331346
ENSG000001	RPL19	0.75168862	0.908573	-0.0331117
ENSG000001	PHYHD1	0.87324844	0.95510037	-0.0331077
ENSG000001	COX6A1	0.69516816	0.88224216	-0.0330644
ENSG000001	IL18RAP	0.75458996	0.90966495	-0.032985
ENSG000001	RAB5C	0.50411527	0.78690354	-0.0329532
ENSG000001	ATP6V0E1	0.47673428	0.77129678	-0.0329363
ENSG000001	MYLK3	0.90782414	0.96899425	-0.0328809
ENSG000001	ZSCAN21	0.66268833	0.86788287	-0.0328344
ENSG000002	STIMATE-MUS	0.75221331	0.908573	-0.0327283
ENSG000002	AP1G2	0.60425719	0.83883394	-0.0327019
ENSG00000(	ARHGAP15	0.64304305	0.85899548	-0.0326956
ENSG000001	MCTP2	0.69522008	0.88224216	-0.0326791
ENSG000001	RRP36	0.54120688	0.80757124	-0.0326201
ENSG000001	FBXW2	0.58912645	0.83172299	-0.0326032
ENSG000001	ACAD8	0.60281925	0.83858603	-0.032568
ENSG000001	RPS3	0.80876382	0.93001	-0.0325121
ENSG00000(	LMCD1	0.91711372	0.97245317	-0.032493
ENSG000001	SUSD6	0.67854826	0.8756627	-0.0324735
ENSG000001	DUS3L	0.67603208	0.87463061	-0.0324709
ENSG000001	ZNF71	0.69130904	0.88131945	-0.0324631
ENSG000001	SRPX2	0.91889154	0.97272242	-0.0324488
ENSG000001	GRAP2	0.69007259	0.88074719	-0.0324413

ENSG000001	ZNF212	0.50084509	0.78519683	-0.0323548
ENSG000001	BCL9L	0.74245304	0.90558693	-0.0323057
ENSG000001	POM121	0.57840554	0.82533908	-0.0322593
ENSG000001	CD101	0.75738044	0.91082077	-0.0321982
ENSG000001	MAPKAPK3	0.35564291	0.69207883	-0.0321976
ENSG00000C	MASP2	0.81482315	0.93228896	-0.03214
ENSG000001	CLUH	0.69388772	0.88206401	-0.0320784
ENSG000001	TMEM80	0.52663177	0.80019714	-0.0320096
ENSG000001	SGPP2	0.77311778	0.91571183	-0.0320053
ENSG000001	APRT	0.67569143	0.87451032	-0.0320028
ENSG000002	H2BC6	0.81995951	0.93468999	-0.0319995
ENSG000001	SALL2	0.79560868	0.9246397	-0.0319922
ENSG000001	THAP11	0.64516055	0.85991395	-0.0319554
ENSG000001	RALGDS	0.70974506	0.88993007	-0.0318919
ENSG000001	MEA1	0.65345656	0.86395123	-0.0318848
ENSG000001	BRSK2	0.82908273	0.93682924	-0.0318843
ENSG000001	NDRG1	0.48763894	0.77796645	-0.0318693
ENSG000001	MRNIP	0.72224409	0.89532737	-0.031834
ENSG00000C	PSEN1	0.32940544	0.67311302	-0.0317863
ENSG000001	RPS11	0.77012014	0.91423988	-0.0317764
ENSG000001	BRPF1	0.54052144	0.80732652	-0.0317678
ENSG000001	MRPS26	0.69432076	0.8821445	-0.0317629
ENSG000001	PTTG1IP	0.24041057	0.60721888	-0.0317401
ENSG000001	UBE2I	0.60216616	0.83852406	-0.0317307
ENSG000001	STAR	0.91260591	0.97123107	-0.0317029
ENSG000001	FAU	0.77820491	0.91795445	-0.031659
ENSG000001	PLXNA1	0.79100188	0.92297405	-0.0316502
ENSG000001	ITM2B	0.55554241	0.8145753	-0.0316443
ENSG000001	LPCAT3	0.49232354	0.7804495	-0.0315621
ENSG00000C	CLUL1	0.94488342	0.98252902	-0.0315573
ENSG000001	CXXC1	0.63484947	0.85504577	-0.0315263
ENSG000001	SLC44A5	0.91823988	0.97272242	-0.0314762
ENSG000001	MICOS13	0.7466467	0.90675411	-0.0314125
ENSG000001	PLGLB1	0.82526457	0.93591669	-0.0313686
ENSG000001	KRI1	0.63583558	0.85527813	-0.0313611
ENSG000001	SV2A	0.76537629	0.91260342	-0.0312932
ENSG000001	TAF1C	0.64954943	0.86257742	-0.0312845
ENSG000002	CHMP1B	0.66876724	0.87125589	-0.0312755
ENSG000001	HEXIM1	0.52568664	0.79977109	-0.0312582
ENSG000001	TMEM25	0.7783201	0.91796204	-0.0312525
ENSG000002	LEFTY1	0.86460898	0.95224252	-0.031231
ENSG00000C	ATP2B1	0.68848615	0.88057199	-0.0312297
ENSG000001	ZNF526	0.66226102	0.86746791	-0.0311612

ENSG000001	SPPL3	0.24110254	0.60721888	-0.0311319
ENSG000001	DPP7	0.72035108	0.8946285	-0.0310946
ENSG00000C	SRRT	0.53541536	0.80413676	-0.0310348
ENSG000001	CYB561D2	0.67626477	0.87463061	-0.0310202
ENSG00000C	FGR	0.55295914	0.81383402	-0.0310193
ENSG000001	MN1	0.83205899	0.93831391	-0.0310077
ENSG000001	C16orf72	0.63948407	0.85691647	-0.0310011
ENSG000002	RTL8A	0.64131171	0.8575293	-0.0309504
ENSG000001	RPL8	0.74333978	0.90602454	-0.0309417
ENSG00000C	PKD1	0.76713148	0.91352929	-0.0309169
ENSG000001	ZFHX3	0.72335623	0.89589131	-0.0308854
ENSG00000C	ARHGEF5	0.78082436	0.9189743	-0.0308698
ENSG000001	HARS2	0.3846007	0.71295371	-0.0307968
ENSG000001	RIC3	0.8316165	0.93804129	-0.0307524
ENSG000001	OLFM2	0.76985994	0.91402572	-0.0307143
ENSG000001	COG1	0.56320379	0.81861427	-0.0306179
ENSG000002	ANKRD34A	0.82549612	0.93591669	-0.0305998
ENSG000001	TESK1	0.66402202	0.86837979	-0.0305977
ENSG000001	TAF4B	0.79526643	0.92462665	-0.030523
ENSG000001	SFXN3	0.62260603	0.84922092	-0.0304967
ENSG000001	APEH	0.51862677	0.79577989	-0.0304846
ENSG000001	NAP1L3	0.88656606	0.96095764	-0.0304308
ENSG000001	TRIM74	0.84118025	0.94272653	-0.0303942
ENSG000001	ZNF408	0.63809075	0.8564206	-0.0303544
ENSG00000C	NME8	0.79566872	0.9246397	-0.0303347
ENSG000001	SYNGAP1	0.92176392	0.97393309	-0.0302777
ENSG00000C	AP3D1	0.52922507	0.8011926	-0.0302655
ENSG000001	DCLRE1B	0.67619522	0.87463061	-0.0301851
ENSG000001	DNAJB6	0.40775586	0.72771726	-0.0301726
ENSG000001	DAPK1	0.69222906	0.88158966	-0.0301544
ENSG000001	TAF12	0.53240656	0.80280163	-0.0301501
ENSG000001	COL14A1	0.93280729	0.97819097	-0.0301332
ENSG000002	ARL2	0.66749445	0.87060064	-0.0301012
ENSG000001	CADM4	0.74210746	0.90555138	-0.0300953
ENSG000001	ST6GALNAC3	0.82869753	0.9367559	-0.0299141
ENSG000001	ACADS	0.71808849	0.89332947	-0.0298895
ENSG000002	EGLN2	0.63193544	0.85385169	-0.0297661
ENSG00000C	TOMM34	0.63714872	0.85602759	-0.0297607
ENSG000001	SUMF1	0.61798594	0.84662823	-0.0297333
ENSG000002	STMP1	0.64233761	0.85833559	-0.0297256
ENSG000001	ANKRD55	0.73738197	0.90250162	-0.0296513
ENSG00000C	GMCL1	0.74555632	0.9064753	-0.029641
ENSG000001	UBE2O	0.74108162	0.90520893	-0.0296388

ENSG000001	MEGF8	0.73571609	0.90150345	-0.0296011
ENSG000002	H2AC12	0.94990417	0.98403516	-0.02953
ENSG000001	CRY2	0.57707932	0.82458032	-0.0295244
ENSG000001	YDJC	0.74303794	0.90596283	-0.0294861
ENSG000001	USP9X	0.6168694	0.84613614	-0.0294468
ENSG000001	ARMH1	0.71817172	0.89332947	-0.0294409
ENSG000001	SCRG1	0.91597234	0.9722662	-0.029372
ENSG000001	FAAP100	0.72240463	0.89532737	-0.0293666
ENSG000001	NT5DC4	0.82797485	0.93660278	-0.0293647
ENSG000001	NDUFS8	0.72835925	0.89755262	-0.0293416
ENSG000001	SURF1	0.5180018	0.79566132	-0.0293116
ENSG000001	PSMD2	0.50806657	0.79067965	-0.0292949
ENSG000001	ZYG11B	0.709963	0.88993477	-0.0292668
ENSG000001	MEX3B	0.88114765	0.9590558	-0.0292629
ENSG00000C	RASSF1	0.72571031	0.89686038	-0.0292342
ENSG000001	ASCC1	0.65134248	0.8634351	-0.0291893
ENSG000001	SUMF2	0.72764345	0.8971681	-0.0291525
ENSG000001	TGFBR2	0.56523621	0.81917305	-0.0291029
ENSG000001	IQCC	0.80647953	0.92967903	-0.0290987
ENSG000001	NUBPL	0.82357984	0.93558785	-0.0290606
ENSG000001	PURG	0.9394397	0.97998953	-0.029025
ENSG000001	TRIM46	0.84013153	0.94245133	-0.0290193
ENSG000001	HIRA	0.69184276	0.88153896	-0.0289998
ENSG000001	BIN1	0.7780269	0.91788294	-0.0289571
ENSG000001	LRGUK	0.92585722	0.97536168	-0.0289174
ENSG00000C	KEAP1	0.58213511	0.82746456	-0.0288895
ENSG000001	TENT5C	0.79699902	0.92560426	-0.0288485
ENSG000001	ZNF683	0.89472471	0.96412642	-0.0287839
ENSG000002	E2F4	0.37912268	0.70965485	-0.0287831
ENSG000001	AP1B1	0.58077994	0.82662376	-0.0287247
ENSG000001	PIGC	0.65465306	0.86439091	-0.0286995
ENSG000001	SLC25A1	0.72660093	0.89706038	-0.0286742
ENSG000001	EIF3K	0.65735371	0.86522797	-0.0286627
ENSG000001	PAK1	0.67101876	0.87218472	-0.0286223
ENSG00000C	MSANTD3	0.79244063	0.92314956	-0.0285669
ENSG000001	NRBP2	0.75132013	0.908573	-0.0285634
ENSG000001	ERMN	0.77473522	0.91628579	-0.028511
ENSG000001	ACKR2	0.87825668	0.95778789	-0.0285022
ENSG00000C	DEPDC1	0.95649846	0.98578332	-0.0284741
ENSG00000C	CRLF1	0.89747935	0.96522986	-0.0284303
ENSG000001	MIDEAS	0.72232342	0.89532737	-0.0284118
ENSG000001	MTMR14	0.42551705	0.73817463	-0.0283887
ENSG000001	PIAS4	0.61093883	0.84334674	-0.0283851

ENSG000001	MBD4	0.6016362	0.83830636	-0.028368
ENSG000001	FAM131B	0.8548286	0.94827164	-0.0283142
ENSG000001	ADHFE1	0.80133012	0.92735626	-0.0283058
ENSG000001	THOC6	0.66176705	0.86736018	-0.0282974
ENSG000002	RNASEK	0.70259371	0.88578415	-0.0282687
ENSG000001	FAM193A	0.5433668	0.8084284	-0.0282681
ENSG000001	CNPY4	0.80419471	0.92881463	-0.0282638
ENSG000001	MPP2	0.87935732	0.95817324	-0.0282562
ENSG000001	PSMB5	0.73854443	0.90333303	-0.0282505
ENSG000001	ADAMTSL4	0.77790604	0.91780703	-0.0282092
ENSG000001	AFMID	0.77825712	0.91795445	-0.0282064
ENSG000001	RRP1	0.63693895	0.85591142	-0.0281172
ENSG000001	RIPOR2	0.54194574	0.80808176	-0.0281021
ENSG000002	MRPL53	0.75768037	0.91082077	-0.0280955
ENSG000001	SLC17A3	0.94968606	0.98403516	-0.0280768
ENSG000001	ZDHHC11	0.8380292	0.94146888	-0.0280765
ENSG00000C	L2HGDH	0.83473604	0.93977114	-0.0280691
ENSG000001	DUSP28	0.78121588	0.9189743	-0.0280454
ENSG000001	MAN1C1	0.78654665	0.92119044	-0.0280113
ENSG000001	MET	0.95723975	0.98578332	-0.0279283
ENSG000001	FHIT	0.82705955	0.93644411	-0.0279236
ENSG00000C	ADGRL1	0.73193582	0.89917898	-0.0279027
ENSG000001	RPS9	0.90069619	0.96608561	-0.0279019
ENSG000002	ZNF814	0.7277611	0.89717235	-0.0278839
ENSG000001	IL27RA	0.72041325	0.8946348	-0.0278782
ENSG000001	SIAE	0.69724828	0.88294449	-0.0278741
ENSG000001	RELB	0.75721476	0.91082077	-0.027826
ENSG00000C	MARK3	0.37356963	0.70563635	-0.0278009
ENSG000001	EIF4A3	0.57143991	0.82164253	-0.0276624
ENSG000001	NCAPH	0.87827506	0.95778789	-0.0276507
ENSG000001	SRM	0.75050897	0.90811642	-0.0276176
ENSG00000C	NFATC3	0.69725607	0.88294449	-0.0275867
ENSG000001	ZMIZ2	0.60550683	0.83945694	-0.0275388
ENSG000001	ARRDC2	0.62265043	0.84922092	-0.0275337
ENSG000001	RBKS	0.85269675	0.94733063	-0.027526
ENSG00000C	TGM1	0.74686258	0.90685156	-0.0275202
ENSG000001	DYNLRB1	0.63749761	0.85609534	-0.0275153
ENSG00000C	IDH3G	0.63726749	0.85602759	-0.0275103
ENSG000001	WDR6	0.69218549	0.88158966	-0.027502
ENSG000001	GPAT4	0.64531984	0.8599145	-0.0274983
ENSG000001	GSR	0.49836279	0.78303818	-0.0274854
ENSG000001	ACSM6	0.94678141	0.98329857	-0.0274651
ENSG000001	ZNF500	0.72151672	0.89490971	-0.0274173

ENSG000001	GBF1	0.60585481	0.83946052	-0.027408
ENSG00000C	KIF22	0.76158475	0.91151487	-0.0273412
ENSG000001	EDF1	0.70759919	0.88890112	-0.0273391
ENSG000001	NDUFB11	0.74792115	0.90731063	-0.027295
ENSG000001	NAGLU	0.74205343	0.90555138	-0.0272803
ENSG000001	AUP1	0.60561388	0.83945694	-0.0272555
ENSG000002	HNRNPUL2	0.53081554	0.80212192	-0.0272343
ENSG000002	N/A	0.82561484	0.93591669	-0.0272237
ENSG000001	PAOX	0.75021753	0.9080858	-0.0272121
ENSG00000C	POLDIP2	0.54648125	0.81061139	-0.0271683
ENSG000001	RINL	0.5873567	0.83046863	-0.0271578
ENSG000001	RPL14	0.81449397	0.93225283	-0.0271367
ENSG000001	PAK4	0.68980125	0.88074719	-0.0271175
ENSG000001	ACOT8	0.61653623	0.84592689	-0.0270998
ENSG000001	RETREG3	0.62450219	0.84989037	-0.0270347
ENSG000001	TCEAL3	0.68778301	0.88051877	-0.0270138
ENSG00000C	PDK2	0.72598929	0.89696303	-0.0269886
ENSG000001	DUOX1	0.83530281	0.9401644	-0.0269856
ENSG000001	GPR107	0.31110468	0.65949478	-0.0269307
ENSG000001	NAB2	0.75748711	0.91082077	-0.0268979
ENSG000001	SURF6	0.64505004	0.85991395	-0.0268707
ENSG000001	MAP1LC3B	0.4846752	0.7769018	-0.0268063
ENSG000001	GSDME	0.85925528	0.95038229	-0.0268017
ENSG000002	NACA2	0.89836631	0.96566178	-0.0268012
ENSG000001	FAM177A1	0.64063733	0.85719228	-0.0268003
ENSG000001	ABRA	0.94025988	0.98028763	-0.0267858
ENSG000001	ZFYVE19	0.69944022	0.88434908	-0.0267674
ENSG000001	EDC3	0.66016814	0.86682436	-0.0267532
ENSG000001	LRRC42	0.52157997	0.79759785	-0.026742
ENSG000001	NCSTN	0.56063541	0.81728196	-0.0267085
ENSG000001	NTN5	0.90039237	0.96608561	-0.0266865
ENSG000002	ZNF727	0.94418017	0.98229342	-0.026683
ENSG000001	ECHDC2	0.81813025	0.93333067	-0.0266733
ENSG000001	IGSF6	0.71655594	0.89257528	-0.0266724
ENSG000001	SLC7A11	0.85424654	0.94814417	-0.0266564
ENSG00000C	SH2D2A	0.76218743	0.91151487	-0.0266537
ENSG000001	KXD1	0.40479417	0.72672584	-0.0266436
ENSG000001	ARFGAP2	0.48647236	0.77752459	-0.0266025
ENSG000001	ZNF93	0.7806191	0.9189743	-0.0265957
ENSG000002	GTF2H5	0.80522216	0.92897486	-0.0265911
ENSG000001	DHCR7	0.74346241	0.90602454	-0.0265637
ENSG000001	ELOVL3	0.85871125	0.95004733	-0.0265603
ENSG000001	SPOP	0.466846	0.7650199	-0.0265491

ENSG000001	CDS2	0.35698119	0.69298434	-0.0265451
ENSG000001	SNRPB	0.69011363	0.88074719	-0.0265017
ENSG000001	PHF21A	0.62124393	0.84851427	-0.0264885
ENSG000001	NAGPA	0.65696867	0.86514195	-0.0264591
ENSG000001	NUDT1	0.75218288	0.908573	-0.0264496
ENSG000001	SGCB	0.83707102	0.94119112	-0.026441
ENSG000001	GIPC1	0.79169478	0.92314169	-0.0264404
ENSG000002	MRPS6	0.78878676	0.92204735	-0.0264203
ENSG000000	MED15	0.59071827	0.83266712	-0.0264158
ENSG000001	CAV2	0.88898123	0.96164016	-0.026408
ENSG000000	CDC45	0.90269642	0.96756393	-0.0263891
ENSG000001	JOSD1	0.67933909	0.87598276	-0.0263633
ENSG000000	METTL22	0.67341373	0.87354948	-0.0263551
ENSG000002	MACO1	0.66886179	0.87125589	-0.0263348
ENSG000001	ZC3H4	0.63822422	0.8564206	-0.0262928
ENSG000001	GEMIN4	0.67263094	0.87337134	-0.0261887
ENSG000001	BNIP3L	0.74861369	0.90731063	-0.0261421
ENSG000001	ODAPH	0.95058784	0.98403516	-0.0261347
ENSG000001	TGOLN2	0.39123962	0.71964297	-0.0261279
ENSG000001	JCAD	0.95113842	0.98403516	-0.0261252
ENSG000001	YKT6	0.27184748	0.63323084	-0.02611
ENSG000001	NT5E	0.82698227	0.93644411	-0.0260562
ENSG000001	SLC15A4	0.58980718	0.83191276	-0.0260321
ENSG000001	SNRPN	0.81243639	0.93170624	-0.0260029
ENSG000001	NDUFS7	0.79354257	0.92392642	-0.0259859
ENSG000001	EFNA3	0.90479593	0.96811674	-0.0259701
ENSG000001	ANKMY1	0.74469762	0.90628646	-0.0259591
ENSG000001	STK36	0.74398699	0.90628646	-0.0259244
ENSG000001	SENP8	0.88220641	0.95956494	-0.0259094
ENSG000001	ZNF785	0.79251064	0.92316488	-0.0259052
ENSG000001	FICD	0.48945099	0.77982687	-0.0258353
ENSG000001	ZNHIT1	0.60752475	0.84095306	-0.0258339
ENSG000001	EAPP	0.75187751	0.908573	-0.0257656
ENSG000001	GOLGB1	0.55631246	0.81507187	-0.0256664
ENSG000000	GNA11	0.74518239	0.90628646	-0.0256549
ENSG000001	SYAP1	0.53589971	0.80423066	-0.0256522
ENSG000001	CHMP3	0.49246763	0.7804495	-0.0255983
ENSG000002	N/A	0.76152268	0.91151487	-0.0255925
ENSG000001	TMC4	0.95521756	0.98521399	-0.025565
ENSG000001	PRXL2A	0.79027608	0.92280247	-0.0255539
ENSG000001	TRIB3	0.72684722	0.89706038	-0.0255334
ENSG000001	ITM2C	0.81365992	0.9318894	-0.0255309
ENSG000002	DCDC2B	0.84104091	0.942702	-0.0255237

ENSG000001	CDH26	0.87626706	0.95679296	-0.0254841
ENSG000001	PDE6B	0.79926385	0.92618093	-0.0254742
ENSG000001	TJAP1	0.58329276	0.82845829	-0.0254605
ENSG000001	FCRLB	0.82317813	0.93539963	-0.0254193
ENSG000001	ATG4D	0.71100282	0.89054277	-0.0254
ENSG000001	UBXN1	0.63996443	0.85706063	-0.0253974
ENSG000001	CMSS1	0.86101253	0.95080722	-0.0253857
ENSG00000C	TENM1	0.87230975	0.95459934	-0.0253823
ENSG000001	TMLHE	0.66873467	0.87125589	-0.0253487
ENSG000001	DDX49	0.6282671	0.85180135	-0.0253433
ENSG000001	MRPL34	0.78482475	0.92041622	-0.0253397
ENSG000001	RSU1	0.58856735	0.83116738	-0.0252919
ENSG000001	ARRB1	0.54531883	0.80952117	-0.0252859
ENSG000001	DUSP16	0.73142453	0.8990265	-0.0252609
ENSG000001	BTD	0.68450927	0.87902381	-0.0252522
ENSG000001	ZFP36	0.71481438	0.89175724	-0.02524
ENSG000001	CD70	0.9233168	0.97437154	-0.0252392
ENSG000001	TRIM62	0.62804369	0.85164067	-0.0252174
ENSG00000C	CACNA2D2	0.82827436	0.93662954	-0.025209
ENSG000001	CCDC9	0.80116966	0.92733047	-0.0251799
ENSG00000C	EIF2B3	0.84721587	0.94527257	-0.025157
ENSG000001	BORCS6	0.7033404	0.88628755	-0.0251484
ENSG000001	LRRK1	0.61746882	0.84653095	-0.0251412
ENSG000001	MDH2	0.64769262	0.86142128	-0.0251
ENSG000001	SDSL	0.84801035	0.94542324	-0.025082
ENSG000001	ZNF829	0.87601648	0.95676199	-0.0250669
ENSG000001	CES2	0.60997495	0.84290213	-0.0250484
ENSG000001	TNFRSF21	0.85757765	0.94961808	-0.0250334
ENSG000001	ESRP2	0.8402854	0.94245133	-0.0249716
ENSG000001	CLEC1A	0.74874504	0.90739051	-0.0249542
ENSG000001	PILRB	0.71896023	0.89385584	-0.0249452
ENSG000001	P4HB	0.61518163	0.84533626	-0.0249412
ENSG00000C	DNASE1L1	0.63520949	0.85504577	-0.0249018
ENSG000001	HIRIP3	0.75095275	0.90838261	-0.0248608
ENSG000001	PTCH2	0.82934695	0.93682924	-0.0248111
ENSG00000C	GPKOW	0.58982446	0.83191276	-0.0247987
ENSG000001	SCNN1A	0.84101141	0.942702	-0.024751
ENSG000001	TTLL5	0.73254215	0.89954428	-0.02474
ENSG000001	OPA3	0.59758997	0.83641817	-0.0247195
ENSG000001	RPS19BP1	0.66040815	0.86700658	-0.0247153
ENSG000001	GPR19	0.86234247	0.95132243	-0.0246957
ENSG000001	C3orf33	0.89322934	0.96361469	-0.0246335
ENSG000002	RPS29	0.90867906	0.96946066	-0.0246207

ENSG000001	RBM44	0.9035093	0.96782428	-0.0245967
ENSG000001	LIPC	0.79840517	0.92603117	-0.0245758
ENSG000001	COPG1	0.58500548	0.82919554	-0.0245715
ENSG000001	BCL9	0.77447361	0.91617933	-0.0245572
ENSG000001	TTC16	0.86107796	0.95080722	-0.0245134
ENSG000001	KDELR1	0.63611968	0.85549278	-0.024496
ENSG000001	CCDC71	0.57710974	0.82458032	-0.024411
ENSG00000(	ALPK1	0.80427907	0.92881463	-0.0243689
ENSG00000(	AP2S1	0.69323039	0.88183499	-0.0243472
ENSG000001	SRPRA	0.40903016	0.72872927	-0.0243078
ENSG000001	GLYCTK	0.77148855	0.9150003	-0.0242687
ENSG000001	RAB31	0.71042842	0.89026983	-0.0242486
ENSG000001	MLX	0.41729388	0.73321077	-0.0242399
ENSG000001	USP7	0.50815121	0.79067965	-0.0242398
ENSG000001	PSMB6	0.74328788	0.90602454	-0.0242124
ENSG000001	ZNF513	0.70337509	0.88628755	-0.0242116
ENSG000001	ATP9B	0.72504891	0.89675649	-0.0241888
ENSG000001	CHEK1	0.88216451	0.95956494	-0.0241588
ENSG000001	UTP4	0.79086112	0.92297405	-0.0241526
ENSG000001	TEX264	0.76283737	0.91167139	-0.0241197
ENSG000001	RPL22	0.82646301	0.93615843	-0.0240799
ENSG00000(	GPATCH1	0.69245149	0.88158966	-0.0240795
ENSG000002	TNFRSF13B	0.88360988	0.96044809	-0.024044
ENSG000001	LAGE3	0.79951495	0.92633968	-0.0240363
ENSG000001	ENDOU	0.92576733	0.97536168	-0.0240361
ENSG000001	PRKCA	0.77983014	0.91827559	-0.0240187
ENSG000001	ADM	0.85214414	0.94731122	-0.0240107
ENSG000001	HTR2B	0.95724283	0.98578332	-0.0240032
ENSG000001	KCTD13	0.66293167	0.86788287	-0.0239977
ENSG000001	TRAPPC4	0.7459997	0.90653584	-0.023961
ENSG000001	NRF1	0.60561671	0.83945694	-0.0239266
ENSG000001	DNMT3A	0.67734538	0.87510133	-0.0239105
ENSG000001	SUN2	0.68695692	0.88028723	-0.0239099
ENSG000001	ZNF491	0.88664576	0.96095764	-0.023907
ENSG000001	SMIM29	0.63745411	0.85609534	-0.0238739
ENSG000001	ZSCAN2	0.81462517	0.93225797	-0.0238532
ENSG000002	NOX5	0.94139851	0.98082846	-0.023825
ENSG000001	HEATR5A	0.82514098	0.93591669	-0.0238177
ENSG000001	USP3	0.43036355	0.7410474	-0.0238154
ENSG000001	BMF	0.6494054	0.86252724	-0.0237264
ENSG000001	EHMT1	0.59235642	0.83392318	-0.0236868
ENSG00000(	BID	0.58616263	0.82959975	-0.0236412
ENSG000001	FUCA1	0.72991443	0.89828814	-0.0236348

ENSG000001	DYNC1LI1	0.68512255	0.87953312	-0.0236058
ENSG000001	KLK2	0.94109189	0.98071427	-0.0235834
ENSG00000C	CAD	0.77312754	0.91571183	-0.0235722
ENSG000001	EGLN1	0.76037945	0.91141904	-0.023565
ENSG000001	ZNHIT2	0.7977052	0.9258334	-0.023503
ENSG000001	SFR1	0.82527916	0.93591669	-0.0234699
ENSG000001	CIR1	0.75837829	0.9110527	-0.0234624
ENSG00000C	MPND	0.71892786	0.89385584	-0.0234456
ENSG000001	CSTF1	0.73565116	0.90150345	-0.0234404
ENSG000001	IGFBP2	0.89985136	0.96608561	-0.0234392
ENSG000001	IVNS1ABP	0.77596533	0.91686844	-0.0234207
ENSG000001	ETHE1	0.7564502	0.91082077	-0.0234115
ENSG000001	IL2RG	0.7303262	0.89842461	-0.0234075
ENSG00000C	HDAC4	0.73246989	0.89954428	-0.0234061
ENSG000001	HERC1	0.73570222	0.90150345	-0.0233442
ENSG000001	TAOK3	0.55942442	0.81696069	-0.0232855
ENSG000001	ANKRD23	0.8841789	0.9606711	-0.0232682
ENSG000001	LIN7A	0.82776618	0.93660278	-0.023263
ENSG000001	ERI3	0.75422065	0.90944172	-0.0232508
ENSG000001	CEP89	0.79743311	0.9258334	-0.023241
ENSG000001	CCDC120	0.77980337	0.91827559	-0.0232275
ENSG000001	LEMD2	0.58304756	0.82845829	-0.0232027
ENSG000001	CPT1C	0.94652839	0.98320732	-0.0231094
ENSG000001	ANAPC7	0.67958921	0.87610083	-0.0230895
ENSG000001	GZMM	0.84119672	0.94272653	-0.0230755
ENSG000001	CCDC171	0.89228538	0.96313198	-0.023062
ENSG000001	C6orf89	0.39214099	0.71994497	-0.023002
ENSG000001	FKBP1C	0.72566904	0.89686038	-0.0229898
ENSG000001	S100A8	0.86675591	0.95271565	-0.0229564
ENSG000001	NRG4	0.95263561	0.9844851	-0.0229368
ENSG000001	VPS4A	0.67084491	0.87216822	-0.0229323
ENSG000001	RPL27A	0.85960639	0.95051954	-0.0229172
ENSG000001	DDA1	0.65233623	0.86379349	-0.0228845
ENSG000001	TBCB	0.73748851	0.90251741	-0.0228681
ENSG000001	SPATA6	0.8466422	0.94500099	-0.022863
ENSG000001	CACNA1E	0.91431118	0.9716023	-0.0228292
ENSG000001	PLXNA3	0.80683445	0.92967903	-0.0226994
ENSG000001	ZNF182	0.79850388	0.92603117	-0.0226883
ENSG000002	NEURL1B	0.92077642	0.97360588	-0.0226587
ENSG00000C	SEMA3B	0.85981899	0.95062414	-0.0226442
ENSG000002	MINDY4B	0.95319295	0.9845509	-0.0226439
ENSG000001	NFATC1	0.72842971	0.89755262	-0.0226328
ENSG000001	PTGER4	0.798118	0.92603117	-0.0226273

ENSG000001	OR10AD1	0.93921977	0.97986875	-0.0226066
ENSG000001	TSNARE1	0.81412416	0.93216232	-0.0225626
ENSG000001	LPGAT1	0.76099215	0.91141904	-0.0225467
ENSG000001	PDE1B	0.76898717	0.91375182	-0.0225371
ENSG000001	AKTIP	0.82519151	0.93591669	-0.0225157
ENSG000001	OGFRL1	0.84080708	0.94263513	-0.0224868
ENSG00000(	FDFT1	0.54104809	0.80757124	-0.0224509
ENSG000001	C3	0.84155256	0.94283868	-0.0224297
ENSG000001	LRRC36	0.94640217	0.98315624	-0.0224138
ENSG000001	ANKRD52	0.69231915	0.88158966	-0.0224051
ENSG000001	RBM4B	0.73516304	0.90147373	-0.0223818
ENSG000001	DDB1	0.67246886	0.87337134	-0.0223751
ENSG00000(	PNPLA4	0.76452499	0.91243616	-0.0222591
ENSG000001	STAT3	0.8072587	0.92967903	-0.0222303
ENSG000001	MYBBP1A	0.77719568	0.91750227	-0.0222117
ENSG000001	TMEM255B	0.87529652	0.95634141	-0.0221291
ENSG000002	CCDC188	0.87078189	0.95437279	-0.0220862
ENSG00000(	CELSR3	0.82854451	0.93664814	-0.0220737
ENSG000001	S100A13	0.84444721	0.94402232	-0.0220515
ENSG000001	APH1B	0.64431689	0.85956564	-0.0220465
ENSG000001	SPATA33	0.79201617	0.92314956	-0.0220423
ENSG000001	PYCR2	0.72287012	0.89570645	-0.0220263
ENSG000001	SDC4	0.82320754	0.93539963	-0.0220118
ENSG000001	KHNYN	0.40904345	0.72872927	-0.0220111
ENSG000001	PLD4	0.84945978	0.94625949	-0.0219341
ENSG000001	DSTN	0.7936366	0.92392642	-0.0219295
ENSG000001	RFT1	0.73327903	0.89994343	-0.0218944
ENSG000001	TTPAL	0.67021812	0.87205157	-0.021886
ENSG000001	PSMD9	0.76693755	0.91352929	-0.0218854
ENSG000001	BCL2L11	0.63837276	0.8564206	-0.0217928
ENSG00000(	DCBLD2	0.84036162	0.94246109	-0.0217788
ENSG000001	ALDH4A1	0.84337005	0.94348901	-0.0217601
ENSG000001	PSD2	0.95087826	0.98403516	-0.0217269
ENSG00000(	SKAP2	0.76307218	0.91183419	-0.021718
ENSG000001	ANKS1B	0.93094759	0.97748293	-0.0216503
ENSG000001	MBOAT4	0.88997072	0.96183364	-0.0216468
ENSG000001	IFT22	0.90155899	0.96669547	-0.0216464
ENSG000001	EMX2	0.9391885	0.97986875	-0.0215591
ENSG00000(	TDP1	0.78240239	0.9193471	-0.0215495
ENSG000002	CWC25	0.96747778	0.98864217	-0.0215278
ENSG000001	SLC16A13	0.77771173	0.91771115	-0.0214944
ENSG000001	LCA5	0.95972853	0.98631483	-0.021447
ENSG00000(	UBR2	0.7404813	0.90481599	-0.0213685

ENSG00000(	PITHD1	0.74783062	0.90731063	-0.0213484
ENSG000001	MVK	0.81343913	0.93183372	-0.0213397
ENSG000001	CNTD1	0.70498913	0.8872197	-0.0213329
ENSG000001	BRD1	0.80399671	0.92881463	-0.0212358
ENSG000001	PIGP	0.85689583	0.94924076	-0.0212202
ENSG00000(	FSTL3	0.78458099	0.92028573	-0.0212151
ENSG000001	OR14L1P	0.96289676	0.98702997	-0.0211858
ENSG000001	XPO7	0.65014054	0.86293893	-0.0211555
ENSG000002	N/A	0.95639943	0.98578332	-0.0211442
ENSG000001	MACROH2A1	0.71507764	0.89175724	-0.0209687
ENSG000001	HNRNPA0	0.73168352	0.89916258	-0.0209556
ENSG000001	GLTP	0.5328696	0.80291422	-0.0209132
ENSG000001	CDH24	0.85525785	0.9485534	-0.0209075
ENSG000001	SLC25A15	0.86221308	0.9512641	-0.0208833
ENSG000001	FNBP1	0.53079069	0.80212192	-0.0208714
ENSG000001	ST13	0.73256599	0.89954428	-0.0208377
ENSG000001	MPHOSPH8	0.73737419	0.90250162	-0.0207922
ENSG000001	METAP1D	0.87585126	0.95675437	-0.0207226
ENSG000002	ZSWIM7	0.82992717	0.93703689	-0.0206849
ENSG00000(	SFSWAP	0.61503576	0.84533626	-0.0206787
ENSG000001	IST1	0.24504497	0.6111208	-0.0206741
ENSG000001	UBE2D2	0.68813135	0.88051877	-0.0206572
ENSG000001	SOD1	0.85461922	0.94818753	-0.0206374
ENSG000002	HSPB9	0.93385191	0.97838131	-0.0205567
ENSG000001	FAM104A	0.73123835	0.89893376	-0.0204273
ENSG000001	MORC2	0.78193514	0.91932519	-0.0204159
ENSG000001	CPSF4	0.77085465	0.91453799	-0.0203621
ENSG000001	NLRP9	0.96884621	0.98906985	-0.0203559
ENSG000001	GCNA	0.81515255	0.93228896	-0.0203313
ENSG000001	PEMT	0.85838673	0.9497652	-0.0203278
ENSG000001	VWA2	0.96071314	0.98640251	-0.0203243
ENSG000001	GPR37L1	0.92105091	0.97363955	-0.0203023
ENSG000001	ATP6V1D	0.76739859	0.91352929	-0.0202937
ENSG000001	COG4	0.71287625	0.89118991	-0.02029
ENSG000001	CHCHD5	0.80061071	0.92694781	-0.0202709
ENSG00000(	CDKL5	0.77323477	0.91571183	-0.0202654
ENSG00000(	PIGB	0.7581436	0.91104039	-0.0202534
ENSG000001	ZNF208	0.95886643	0.98631483	-0.0202302
ENSG000002	TCP10L	0.95002064	0.98403516	-0.020226
ENSG000001	GZF1	0.70240312	0.88567086	-0.0201992
ENSG000001	DSC2	0.85420387	0.94814417	-0.0201729
ENSG000001	SLC25A14	0.80420774	0.92881463	-0.0201315
ENSG000001	ADI1	0.85692991	0.94924076	-0.0201108

ENSG000002	GIGYF2	0.68761055	0.88051877	-0.0201009
ENSG000002	TMEM191C	0.9191903	0.97282387	-0.0200678
ENSG000001	ARHGAP22	0.86786485	0.95327639	-0.0200485
ENSG000001	ADK	0.84504875	0.94421922	-0.0200411
ENSG000001	TTC1	0.7094679	0.88968414	-0.020037
ENSG000001	INTS5	0.72884253	0.89755543	-0.019994
ENSG000001	TPM4	0.56215909	0.81804833	-0.0199803
ENSG000001	LRRC17	0.93226063	0.97787927	-0.0199788
ENSG000000	AAMDC	0.85806495	0.94972056	-0.0199473
ENSG000001	ABCD4	0.78327421	0.91983554	-0.0199423
ENSG000000	FMO1	0.96749689	0.98864217	-0.0199407
ENSG000001	WBP2NL	0.85794909	0.94972056	-0.0198923
ENSG000001	SLC41A3	0.81043502	0.93101733	-0.0198225
ENSG000001	ZBTB45	0.74733681	0.90718509	-0.0197278
ENSG000000	BCS1L	0.8245567	0.93591669	-0.0197224
ENSG000001	HDAC3	0.68449782	0.87902381	-0.019708
ENSG000001	NUP153	0.7598569	0.91136366	-0.0196548
ENSG000001	PABPC1L	0.73243883	0.89954428	-0.0196208
ENSG000001	MPI	0.83644378	0.94092682	-0.0195999
ENSG000000	TRAF3IP2	0.74989969	0.90798817	-0.0195945
ENSG000001	PGK1	0.51126571	0.79171125	-0.0195583
ENSG000001	C12orf42	0.89417057	0.96404358	-0.0194767
ENSG000000	TRAPPC3	0.67583524	0.87451032	-0.0194673
ENSG000001	POLR1D	0.77671497	0.9172058	-0.0194167
ENSG000001	CCNB1IP1	0.88662419	0.96095764	-0.0193662
ENSG000001	OTUD1	0.81269476	0.93170624	-0.0193516
ENSG000001	C12orf60	0.9207792	0.97360588	-0.0193172
ENSG000001	USP11	0.77317271	0.91571183	-0.0192786
ENSG000001	NDUFV3	0.7598843	0.91136366	-0.0192668
ENSG000001	ANKLE2	0.76800522	0.91357827	-0.0192495
ENSG000001	MAML3	0.81741414	0.93316132	-0.0192283
ENSG000001	ATP6V1E1	0.6615361	0.86736018	-0.0191416
ENSG000001	SUOX	0.65997798	0.86664477	-0.0191229
ENSG000001	TUT1	0.83668203	0.94106443	-0.0191063
ENSG000001	RNF113A	0.78401801	0.92007086	-0.0190843
ENSG000001	TRIM24	0.72033868	0.8946285	-0.0190734
ENSG000001	CHTF8	0.62432852	0.84979805	-0.0190368
ENSG000000	SMARCD1	0.69845871	0.88371351	-0.0190313
ENSG000000	XRN2	0.59404611	0.83456808	-0.0189787
ENSG000001	CD99L2	0.70825105	0.88911955	-0.0189327
ENSG000002	N/A	0.92946352	0.976809	-0.0188347
ENSG000001	ZKSCAN4	0.74986256	0.90798817	-0.0187561
ENSG000000	SNX29	0.79867273	0.92603117	-0.0187126

ENSG000001	IRGQ	0.754216	0.90944172	-0.0186641
ENSG000001	SPTBN1	0.83848395	0.9416561	-0.0186406
ENSG000001	CHCHD6	0.88708504	0.96095764	-0.0186153
ENSG000001	OSGIN2	0.81968946	0.93468999	-0.0185764
ENSG00000(	CYP26B1	0.94629478	0.98315624	-0.0185724
ENSG000001	FAM50B	0.81267227	0.93170624	-0.0185448
ENSG000001	STOML2	0.75691054	0.91082077	-0.0185079
ENSG000001	GPATCH3	0.74315134	0.90601403	-0.0184873
ENSG000001	FAM151A	0.95995742	0.98631483	-0.0184816
ENSG000001	KCNK1	0.9513695	0.98403516	-0.0184762
ENSG000001	ZFP92	0.78121237	0.9189743	-0.0184561
ENSG000001	TSPYL2	0.86118561	0.95080722	-0.0184503
ENSG000001	TRABD2A	0.90060756	0.96608561	-0.0184211
ENSG000001	RAD9A	0.79232995	0.92314956	-0.018413
ENSG000001	N/A	0.87494125	0.95615483	-0.0183972
ENSG000001	ANKRD13B	0.91289944	0.97123107	-0.0183627
ENSG000001	H2BC21	0.84501929	0.94421922	-0.0183587
ENSG000001	UROS	0.83221803	0.93838362	-0.0183014
ENSG000001	CASQ1	0.93363747	0.97838131	-0.0182912
ENSG000001	CFAP65	0.93803592	0.9794784	-0.0182162
ENSG000001	GALNT2	0.70858102	0.88938447	-0.018207
ENSG000001	KDM3A	0.79187774	0.92314956	-0.0181985
ENSG000001	MAP3K15	0.94434388	0.98233785	-0.0181972
ENSG000001	PMPCA	0.76502154	0.91260342	-0.0181598
ENSG00000(	KAT6A	0.77241607	0.91518678	-0.018139
ENSG000001	DDX56	0.78616293	0.9210569	-0.0180578
ENSG000001	RNF26	0.79937567	0.9262444	-0.0180443
ENSG000001	C2CD2L	0.67773263	0.8751149	-0.0178887
ENSG000001	BOLA3	0.90631679	0.96827647	-0.0178747
ENSG000001	PXT1	0.96565999	0.98812783	-0.0178373
ENSG00000(	VASH1	0.85010696	0.94644505	-0.0178199
ENSG00000(	BAMBI	0.92517566	0.97521605	-0.0178003
ENSG000001	SLC24A4	0.78336711	0.91983554	-0.0177969
ENSG000001	RAB43	0.86115651	0.95080722	-0.017776
ENSG000002	CLEC2L	0.93486662	0.97859224	-0.0177705
ENSG000001	CDK2AP2	0.79897616	0.92603117	-0.017767
ENSG000001	IFT20	0.83413718	0.93944074	-0.0177656
ENSG000001	YY1	0.68851043	0.88057199	-0.01776
ENSG000001	ANGPTL2	0.96297442	0.98704726	-0.0177464
ENSG000001	NUDT8	0.9233636	0.97437154	-0.0177455
ENSG000001	N4BP1	0.82451125	0.93591669	-0.0177239
ENSG000001	KIF11	0.89243091	0.9631373	-0.0176662
ENSG000001	ZNF217	0.85096814	0.94684256	-0.017657

ENSG000002	FCGR3A	0.80788687	0.92978846	-0.0176405
ENSG000002	N/A	0.90969007	0.9700931	-0.0175953
ENSG00000C	DDX3Y	0.84893971	0.94594163	-0.0175838
ENSG000001	RP9	0.85435968	0.94818753	-0.0175775
ENSG000001	CLCNKA	0.9660369	0.98812783	-0.01757
ENSG000001	TPP1	0.75423676	0.90944172	-0.0175661
ENSG000001	NDUFB8	0.86649956	0.95264796	-0.0175216
ENSG000001	TSHR	0.95067839	0.98403516	-0.0175207
ENSG000001	EEFSEC	0.82277383	0.93539963	-0.0174872
ENSG000001	ANKS3	0.84539708	0.94432529	-0.0174723
ENSG000001	DAG1	0.77043186	0.91430362	-0.017445
ENSG000001	PTPRB	0.946633	0.98320732	-0.0173991
ENSG000001	PPP2R2D	0.70930852	0.88962169	-0.0173925
ENSG000001	WDFY2	0.76847407	0.91358163	-0.0173715
ENSG000001	ATP1A1	0.75166692	0.908573	-0.0173545
ENSG000001	TMEM94	0.7696084	0.91386073	-0.0173466
ENSG000001	UHRF1BP1L	0.8485469	0.94589153	-0.0173372
ENSG000001	SENP3	0.78454972	0.92028573	-0.0173306
ENSG000001	MMP15	0.93200583	0.97784797	-0.0172743
ENSG000001	G6PC3	0.81793247	0.9333228	-0.0172706
ENSG000002	COX19	0.8429159	0.94328484	-0.0172133
ENSG000001	RPS6KA5	0.78361438	0.91983554	-0.0171038
ENSG000001	MPPED1	0.97112464	0.99011676	-0.0170096
ENSG000001	CHST10	0.89237067	0.9631363	-0.0170063
ENSG000001	TMEM9	0.80681248	0.92967903	-0.0170032
ENSG000001	SRGAP1	0.90405434	0.96802495	-0.0169664
ENSG000001	H2BC5	0.87007743	0.95423131	-0.0169414
ENSG000001	SH3TC1	0.80015748	0.92688571	-0.0169075
ENSG00000C	FAM184B	0.87083649	0.95437279	-0.0169075
ENSG000001	SUFU	0.7460338	0.90653584	-0.0169024
ENSG000001	NUDT10	0.9616371	0.9865484	-0.0168586
ENSG000001	NUP210	0.81774276	0.9333228	-0.0167864
ENSG000001	BMP8B	0.88576991	0.96095764	-0.0167832
ENSG00000C	KIF1B	0.82554465	0.93591669	-0.0167796
ENSG000001	ZNF445	0.83918863	0.94205654	-0.016778
ENSG000001	CMTM3	0.63829399	0.8564206	-0.0167703
ENSG000001	RPL5	0.91792589	0.97272242	-0.0167532
ENSG000001	WDR59	0.81031374	0.93094631	-0.0167521
ENSG00000C	SUGP2	0.82881947	0.93682852	-0.016662
ENSG00000C	CADPS2	0.95975485	0.98631483	-0.0166319
ENSG000001	PRMT1	0.82225229	0.93539963	-0.016604
ENSG000001	TXNDC17	0.8544462	0.94818753	-0.0165657
ENSG000001	TAB1	0.80336551	0.92841607	-0.0165617

ENSG000001	CAPN10	0.83556404	0.94026854	-0.0165546
ENSG000002	NDUFA7	0.90520025	0.96827466	-0.0164847
ENSG00000C	NADK	0.85658742	0.94912037	-0.0164747
ENSG000001	SNRNP200	0.69623707	0.88256705	-0.0164729
ENSG000001	TRAP1	0.8426323	0.94324858	-0.0163237
ENSG00000C	DRD4	0.97164691	0.99023671	-0.0162876
ENSG000001	CAMKMT	0.88514968	0.96095764	-0.016236
ENSG00000C	THRAP3	0.57938551	0.8260148	-0.0162272
ENSG000001	NME6	0.7737508	0.91586084	-0.0162149
ENSG000001	NDUFS5	0.90880842	0.96953498	-0.0161878
ENSG000001	RAE1	0.75029888	0.9080858	-0.0161825
ENSG00000C	DEPDC1B	0.94792118	0.98373753	-0.0161624
ENSG000001	HINFP	0.76212504	0.91151487	-0.0161338
ENSG000001	PTGES2	0.85795046	0.94972056	-0.0161061
ENSG00000C	JADE1	0.82130907	0.93539963	-0.0160536
ENSG000001	FN3KRP	0.84022766	0.94245133	-0.0160493
ENSG000001	PCYOX1L	0.85266805	0.94733063	-0.016043
ENSG000002	CCDC71L	0.83074724	0.93764588	-0.016037
ENSG000001	FANCD2	0.82284925	0.93539963	-0.0159924
ENSG000001	SOCS3	0.90601592	0.96827466	-0.0159704
ENSG00000C	ATXN7L3	0.58949692	0.831894	-0.0159555
ENSG000001	TESPA1	0.87633036	0.95679296	-0.0159354
ENSG000001	ELP5	0.81186572	0.93164748	-0.0158648
ENSG000002	TEN1-CDK3	0.89845088	0.96566178	-0.0158642
ENSG000001	DGUOK	0.75933028	0.91125749	-0.0158174
ENSG00000C	ITGAL	0.77884077	0.91810931	-0.0158049
ENSG000001	ELK1	0.77488679	0.91637558	-0.0158047
ENSG000001	CLIC6	0.97758831	0.99280436	-0.0158026
ENSG000001	ZSWIM9	0.81113507	0.931484	-0.0157484
ENSG000001	FAM153A	0.92165837	0.97390127	-0.0157189
ENSG000002	PTX4	0.95148615	0.98403516	-0.0157063
ENSG000001	SDHB	0.67672164	0.87490479	-0.0156871
ENSG000001	AMDHD1	0.91790263	0.97272242	-0.0156411
ENSG00000C	GANAB	0.74858255	0.90731063	-0.0156114
ENSG000001	OR2C3	0.96592284	0.98812783	-0.0156057
ENSG000001	MMP23B	0.94477522	0.98252902	-0.0155992
ENSG000001	LIMK2	0.89049005	0.96200179	-0.0155898
ENSG000001	CPB1	0.97060201	0.98995829	-0.0155318
ENSG00000C	MAVS	0.73287957	0.8997252	-0.0154999
ENSG00000C	TRAF1	0.89388903	0.9639421	-0.0154978
ENSG000001	AHDC1	0.8462364	0.94485306	-0.0154477
ENSG000002	POLR2J2	0.88004175	0.95827424	-0.0154357
ENSG000001	ANAPC13	0.79121656	0.92298177	-0.0154148

ENSG000001	VAC14	0.77110774	0.91463929	-0.0153936
ENSG000002	MRPL33	0.88229254	0.95959434	-0.0153715
ENSG000001	CDC37	0.76660695	0.9132353	-0.0153461
ENSG000001	CERS5	0.61008861	0.84290213	-0.0153202
ENSG000001	PAQR4	0.89422224	0.96404358	-0.0153045
ENSG000001	ELANE	0.97819081	0.99280436	-0.0152919
ENSG00000C	NOA1	0.82965566	0.93686532	-0.0152793
ENSG000001	SERINC5	0.85820387	0.94974484	-0.015276
ENSG000001	SGSM1	0.88813434	0.96164016	-0.0151529
ENSG000001	KLC1	0.81488537	0.93228896	-0.0151203
ENSG000001	DUSP7	0.81616868	0.93252627	-0.0151027
ENSG000002	AP5B1	0.90435158	0.9680581	-0.0150711
ENSG000001	KIF5A	0.94501175	0.98252902	-0.0150529
ENSG000001	CCDC78	0.91688199	0.97239796	-0.0150009
ENSG000001	ZMYND8	0.80597035	0.92950781	-0.0149533
ENSG000001	GPR152	0.96108551	0.98648116	-0.0149517
ENSG00000C	DDX11	0.85516357	0.94851362	-0.014951
ENSG000001	CACNB3	0.89610233	0.96472706	-0.0149448
ENSG00000C	UNKL	0.82149793	0.93539963	-0.0149349
ENSG000001	TAS1R3	0.90596257	0.96827466	-0.0149032
ENSG000001	IPO13	0.82457354	0.93591669	-0.014902
ENSG000002	MYBPHL	0.97461327	0.99140483	-0.0148401
ENSG000001	VWA1	0.97242572	0.99060647	-0.0148109
ENSG000001	UBAP2	0.77617852	0.91690146	-0.0147974
ENSG000001	KLHDC8B	0.8575949	0.94961808	-0.0147947
ENSG000001	GEMIN7	0.82973484	0.93688488	-0.0147921
ENSG000001	TP53I13	0.87258603	0.95472907	-0.014739
ENSG000001	HIPK1	0.8472367	0.94527257	-0.014725
ENSG000001	MRGBP	0.86987136	0.95423131	-0.0147215
ENSG000001	TRMT12	0.87846971	0.95778789	-0.0147077
ENSG000002	H2BC9	0.90716186	0.96868131	-0.0146625
ENSG000001	FAHD2A	0.87240233	0.95459934	-0.0146107
ENSG000001	SYNE3	0.85228086	0.94731122	-0.0145788
ENSG000001	MACIR	0.88621782	0.96095764	-0.0145394
ENSG000002	RBM14	0.81689724	0.93280078	-0.0145345
ENSG000002	KLRC2	0.96359422	0.98725092	-0.0145325
ENSG000001	ENO2	0.90032073	0.96608561	-0.0145304
ENSG000001	SETD9	0.92148015	0.97390127	-0.0145135
ENSG000001	SLC51A	0.81304094	0.93170624	-0.0145134
ENSG000001	DUSP22	0.75043003	0.90811642	-0.0144962
ENSG000001	MFSD2A	0.83489553	0.93977114	-0.0144744
ENSG000001	CNNM2	0.75893381	0.91112428	-0.0144507
ENSG000001	ING1	0.72731616	0.89708846	-0.014423

ENSG000001	KIAA0825	0.91300479	0.97123107	-0.0144136
ENSG000001	GTF3C2	0.76105058	0.91141904	-0.0144033
ENSG000001	MST1	0.90747565	0.96881334	-0.0143955
ENSG000001	TMPPE	0.88385685	0.96050431	-0.014393
ENSG000002	TMEM240	0.92922579	0.97674883	-0.0143701
ENSG00000C	ZNF76	0.84476043	0.94419874	-0.0143484
ENSG000001	RAB11FIP4	0.71275227	0.89116324	-0.0143452
ENSG000001	CD40LG	0.89665706	0.96490939	-0.0142998
ENSG000001	SART1	0.84194219	0.94289495	-0.0142981
ENSG000001	LY9	0.90529055	0.96827466	-0.0142925
ENSG00000C	SPHK2	0.8325254	0.93841777	-0.0142868
ENSG000001	PFKL	0.81493104	0.93228896	-0.0142448
ENSG000002	TRAPPC2B	0.95094068	0.98403516	-0.0142354
ENSG000001	DNAAF5	0.85060112	0.94684256	-0.0142322
ENSG000001	C22orf23	0.91056987	0.9705214	-0.0142169
ENSG000001	ALDH7A1	0.95930976	0.98631483	-0.0142096
ENSG000001	GLYR1	0.56809939	0.82037292	-0.0141985
ENSG00000C	AGBL5	0.84224601	0.9429376	-0.0141018
ENSG000001	CDKN1C	0.94780175	0.98373753	-0.0140139
ENSG00000C	COASY	0.75229115	0.908573	-0.0139856
ENSG000001	CSF2RB	0.89071939	0.96218124	-0.0139242
ENSG000001	PIGT	0.71912632	0.89386235	-0.0139102
ENSG00000C	JMJD6	0.74209362	0.90555138	-0.0139073
ENSG000001	ZNF764	0.84985964	0.94644505	-0.0138462
ENSG000001	GID4	0.81392631	0.93212875	-0.0138274
ENSG000001	ATG7	0.71152617	0.8906831	-0.0137656
ENSG000001	PARN	0.97933995	0.99292419	-0.0137499
ENSG000001	RCC1	0.8799793	0.95827424	-0.0137372
ENSG000001	CDC42EP1	0.8814634	0.95922591	-0.013714
ENSG000001	ING5	0.86553564	0.95237042	-0.013711
ENSG000001	TNFAIP2	0.86083035	0.95080722	-0.0136825
ENSG000002	PAGR1	0.88079539	0.95876913	-0.013662
ENSG000002	N/A	0.89228898	0.96313198	-0.0136101
ENSG000001	BLK	0.86486648	0.95228442	-0.0136008
ENSG000001	GOT2	0.83690056	0.94117983	-0.0135764
ENSG000001	DNAH3	0.937857	0.97941755	-0.013558
ENSG000001	CCDC115	0.82185332	0.93539963	-0.0135575
ENSG000001	ZNF385A	0.84229642	0.9429376	-0.0135555
ENSG000002	XKR7	0.89077468	0.96218124	-0.0135521
ENSG000001	ZFAND2A	0.84123697	0.94272653	-0.0135422
ENSG000001	RNF181	0.82121419	0.93539963	-0.0135161
ENSG000001	MEGF10	0.96800327	0.98871211	-0.013493
ENSG000001	PPAN	0.90017206	0.96608561	-0.0133812

ENSG000001	MYO10	0.92225448	0.97410521	-0.0133452
ENSG000001	RNF19B	0.87096451	0.95437279	-0.0133295
ENSG000001	LRRC41	0.8181373	0.93333067	-0.013311
ENSG000002	LTB4R2	0.88514043	0.96095764	-0.0133108
ENSG000001	SEPTIN12	0.95478161	0.98499328	-0.0132865
ENSG000001	SEPTIN1	0.894938	0.96412642	-0.0132333
ENSG000001	SLC29A3	0.84602047	0.94474488	-0.0132332
ENSG000000	MVP	0.88186421	0.95932126	-0.0131979
ENSG000001	VAPB	0.8390287	0.94195206	-0.0131949
ENSG000000	HEBP2	0.86818718	0.95338532	-0.0131727
ENSG000001	UPF2	0.71999762	0.89452914	-0.0131513
ENSG000001	TSPAN10	0.95092652	0.98403516	-0.0131175
ENSG000001	WDR25	0.81987225	0.93468999	-0.0131091
ENSG000001	BMP2K	0.85803133	0.94972056	-0.0130814
ENSG000001	CD163	0.86383798	0.95211331	-0.0130809
ENSG000001	HNRNPK	0.65146612	0.8634351	-0.0130775
ENSG000001	C1QTNF4	0.9667037	0.98843703	-0.0130595
ENSG000001	ISG20L2	0.63560194	0.85510554	-0.0130304
ENSG000001	STARD9	0.92749478	0.97641005	-0.0130111
ENSG000001	MEIG1	0.97799301	0.99280436	-0.013007
ENSG000001	TRMT112	0.85389972	0.94801844	-0.0129877
ENSG000001	WDR82	0.79318274	0.92368272	-0.012962
ENSG000001	SRSF9	0.80033941	0.92689709	-0.0129352
ENSG000001	ARID1A	0.79168465	0.92314169	-0.0129094
ENSG000001	H3-3B	0.82109766	0.93539963	-0.0128934
ENSG000002	HGH1	0.87433912	0.95579655	-0.0128845
ENSG000001	LKAAEAR1	0.95283107	0.9844851	-0.0128801
ENSG000001	BTBD6	0.85338433	0.94777027	-0.0128562
ENSG000001	SUSD4	0.8990938	0.96577044	-0.0128258
ENSG000001	SEC24C	0.74694144	0.90685156	-0.0128214
ENSG000001	CIB1	0.83180333	0.93817662	-0.0128113
ENSG000001	MAP2K5	0.84436553	0.94401729	-0.0127389
ENSG000001	DEDD	0.70023054	0.88463644	-0.0126804
ENSG000001	PSCA	0.97384997	0.99120425	-0.0126475
ENSG000001	CENPH	0.9203238	0.97350473	-0.0125911
ENSG000000	LUC7L	0.89149669	0.96270496	-0.0125789
ENSG000001	PTGDR2	0.93228897	0.97787927	-0.0125281
ENSG000001	PHF19	0.8778813	0.95753437	-0.012522
ENSG000000	TNPO3	0.63912096	0.85691647	-0.0124649
ENSG000001	ZNF609	0.82903311	0.93682924	-0.012451
ENSG000001	GOLGA7B	0.91791673	0.97272242	-0.012423
ENSG000001	IL34	0.94797775	0.98373753	-0.0124099
ENSG000002	ALG3	0.84743272	0.94527257	-0.0124041

ENSG000001	CCDC167	0.93427876	0.97850748	-0.0124004
ENSG000001	ARFIP2	0.86565237	0.95237042	-0.0123791
ENSG000001	TSEN2	0.92598317	0.9754242	-0.0123482
ENSG000001	VPS72	0.82213323	0.93539963	-0.0123437
ENSG000001	ACAD10	0.82582646	0.93591669	-0.0123208
ENSG000001	THAP7	0.88623377	0.96095764	-0.0123011
ENSG000001	LRRC14B	0.97628381	0.99235759	-0.0122394
ENSG000001	CLP1	0.73888199	0.90360977	-0.0121711
ENSG000001	ZNF442	0.96042854	0.98631483	-0.0121415
ENSG000001	NDUFS6	0.87950972	0.95817324	-0.0121332
ENSG000001	FOCAD	0.84645281	0.94497018	-0.0121295
ENSG000001	COX4I1	0.88508422	0.96095764	-0.0120141
ENSG000001	GPR55	0.90609622	0.96827466	-0.012009
ENSG000001	GRTP1	0.92763898	0.97641005	-0.0120013
ENSG000001	AKAP1	0.88297675	0.96008126	-0.0119527
ENSG000001	PEX10	0.88316815	0.96016078	-0.0119418
ENSG000001	UQCR11	0.87655634	0.95679296	-0.0118723
ENSG000001	NEDD8	0.82939708	0.93682924	-0.0118534
ENSG000002	ZNF229	0.97087032	0.99007797	-0.0118429
ENSG000001	ULK4	0.89560934	0.9644521	-0.0118264
ENSG00000C	CNOT3	0.90202856	0.96713139	-0.0118161
ENSG000002	ZNF564	0.94099115	0.98067223	-0.0117958
ENSG000001	CLIP1	0.83640693	0.94092682	-0.0117647
ENSG000001	ROBO3	0.93961391	0.98005467	-0.0117538
ENSG000001	CNIH1	0.92503137	0.97521605	-0.0117373
ENSG000001	LOXL3	0.8688674	0.95366611	-0.0117336
ENSG000001	NIBAN2	0.87673965	0.9567976	-0.0117296
ENSG000001	CLYBL	0.92819534	0.97652064	-0.0117215
ENSG000001	MMP24	0.88524678	0.96095764	-0.0117074
ENSG000001	CSGALNACT:	0.91657815	0.97226625	-0.0116505
ENSG00000C	PRICKLE3	0.85893066	0.95016058	-0.0116365
ENSG000001	HES6	0.93001233	0.97715121	-0.0116152
ENSG000001	TBCC	0.82519152	0.93591669	-0.0115857
ENSG000002	CD8B2	0.95533269	0.98521399	-0.0115808
ENSG000001	APAF1	0.9090151	0.96962805	-0.0115686
ENSG000001	GTF2F1	0.75877252	0.91112183	-0.0115686
ENSG000001	CDNF	0.96093828	0.98647152	-0.0115615
ENSG000001	DGCR8	0.86454481	0.95224252	-0.0115545
ENSG000001	URGCP	0.88673897	0.96095764	-0.0115463
ENSG000001	PIN1	0.86700892	0.95280026	-0.011537
ENSG000001	FBXO36	0.94632027	0.98315624	-0.0115197
ENSG00000C	SPAG7	0.88442804	0.96075855	-0.0115028
ENSG000001	PPIP5K1	0.89743915	0.96522986	-0.0114526

ENSG00000(	SH3BP2	0.78459785	0.92028573	-0.0114387
ENSG000002	NBPF19	0.86558936	0.95237042	-0.0114257
ENSG000001	POLG	0.81530035	0.93228896	-0.0113994
ENSG000001	RIPK3	0.86244172	0.95132243	-0.0113405
ENSG000001	ZNF541	0.95820555	0.98595586	-0.0113203
ENSG000001	SNTA1	0.92186255	0.97393309	-0.0113116
ENSG000001	TBCD	0.87166647	0.95449478	-0.0113058
ENSG00000(	UQCRC1	0.8432328	0.94340047	-0.0112956
ENSG000001	ACTR1B	0.88452149	0.96079579	-0.0112866
ENSG000001	HK1	0.8464982	0.94497018	-0.0112435
ENSG00000(	TMEM14A	0.94349209	0.9821999	-0.0112283
ENSG000001	TRMT61A	0.90045867	0.96608561	-0.0112122
ENSG00000(	HADHA	0.7915738	0.92313321	-0.0111982
ENSG000001	DMAP1	0.86850837	0.95343718	-0.0111497
ENSG00000(	ZCWPW1	0.78662102	0.92119044	-0.011066
ENSG000001	LRRN2	0.91148304	0.97090829	-0.011045
ENSG000001	RBPJ	0.8098565	0.93055269	-0.0110119
ENSG000001	ACVR1B	0.8515429	0.94715044	-0.0110065
ENSG000001	PDE1C	0.9835448	0.99392119	-0.0109181
ENSG000001	IFFO2	0.91248507	0.97123107	-0.0108919
ENSG000001	JAK3	0.84761018	0.94527257	-0.0108897
ENSG00000(	CDC14B	0.90556647	0.96827466	-0.0108759
ENSG000001	ARSJ	0.97870252	0.99291627	-0.0108467
ENSG000001	RCL1	0.89382769	0.9639421	-0.0108028
ENSG000001	RPUSD3	0.8828468	0.96000425	-0.0107735
ENSG000001	TOB1	0.90761114	0.96883059	-0.0107542
ENSG00000(	PDK3	0.84321047	0.94340047	-0.0107523
ENSG000001	GCC1	0.81118333	0.931484	-0.0106972
ENSG000001	FCHO2	0.94130078	0.98080606	-0.010696
ENSG000001	HENMT1	0.93166114	0.97763745	-0.0106864
ENSG000001	NAA50	0.90446024	0.96807652	-0.0106825
ENSG000001	BPGM	0.94004257	0.98022609	-0.010677
ENSG000002	AWAT1	0.98118081	0.99323148	-0.0105299
ENSG000001	NAT9	0.8801345	0.95827424	-0.0105294
ENSG00000(	KDM4A	0.74129727	0.90533613	-0.0105263
ENSG000001	DDRGK1	0.8749491	0.95615483	-0.010526
ENSG000001	TLE5	0.90267827	0.96756393	-0.0105016
ENSG000001	TBC1D16	0.90818419	0.96925113	-0.010437
ENSG000001	CCDC174	0.68123441	0.87687561	-0.0103785
ENSG000001	KCNJ15	0.95106788	0.98403516	-0.010373
ENSG00000(	RNF4	0.74611312	0.90653584	-0.010372
ENSG000001	FTCD	0.98070057	0.99311682	-0.0103563
ENSG000001	UXT	0.9079933	0.9691111	-0.0103493

ENSG000001	MEN1	0.88664881	0.96095764	-0.0103214
ENSG000001	APOO	0.90015022	0.96608561	-0.0101783
ENSG000001	NSRP1	0.84493835	0.94421922	-0.0101639
ENSG000001	SSR2	0.88798294	0.96159657	-0.0101476
ENSG000001	COPG2	0.83977128	0.94238484	-0.0101388
ENSG000001	ILDR1	0.96457508	0.98781492	-0.0101135
ENSG000001	MED31	0.924541	0.975107	-0.0100904
ENSG000001	FHDC1	0.94398934	0.98221647	-0.0100532
ENSG000002	CD302	0.9223405	0.97410521	-0.0100245
ENSG000001	PQBP1	0.86813436	0.95338532	-0.0099919
ENSG000001	MUS81	0.88831921	0.96164016	-0.0099858
ENSG000001	RASA3	0.89866945	0.96576173	-0.009942
ENSG000001	SAPCD2	0.93166478	0.97763745	-0.0098831
ENSG000001	CNR1	0.96037556	0.98631483	-0.0098793
ENSG000001	CACNG6	0.9481866	0.98373753	-0.0098522
ENSG000001	H1-0	0.90944466	0.96995881	-0.0098075
ENSG000001	VOPP1	0.8222755	0.93539963	-0.009743
ENSG000001	CEP250	0.89894986	0.96576173	-0.0097386
ENSG000002	ZNF587B	0.91280888	0.97123107	-0.0097117
ENSG000001	LCN10	0.96708011	0.98843703	-0.0097023
ENSG000001	ATPAF2	0.89834998	0.96566178	-0.0096586
ENSG000002	NPIPA2	0.96939059	0.98933227	-0.0096526
ENSG000001	RGP1	0.81665006	0.93280078	-0.0096423
ENSG000001	GCNT7	0.94222107	0.98113535	-0.0096404
ENSG000001	DCUN1D2	0.91882292	0.97272242	-0.0096276
ENSG00000C	ADIPOR2	0.71595553	0.89223808	-0.0096099
ENSG000001	ARMC2	0.92743954	0.97641005	-0.0095939
ENSG000001	TNFSF13	0.87304804	0.95504496	-0.0095828
ENSG000001	SURF4	0.82738338	0.93657445	-0.0095666
ENSG000001	CHAC1	0.9809036	0.99314408	-0.0095335
ENSG000001	NFIL3	0.92860178	0.97652064	-0.0095314
ENSG000001	ENPP2	0.91529073	0.97217084	-0.0095157
ENSG000001	DCDC2	0.9834136	0.99392119	-0.0094999
ENSG000001	ADCY10	0.96043751	0.98631483	-0.0094594
ENSG000001	PYGO2	0.87659371	0.95679296	-0.0094355
ENSG000001	WDR74	0.88864923	0.96164016	-0.0094177
ENSG000001	FAM3B	0.97868071	0.99291627	-0.0094051
ENSG000001	STYXL1	0.88082522	0.95876913	-0.0094017
ENSG000002	HAUS7	0.94634824	0.98315624	-0.009386
ENSG000001	CDK2AP1	0.9061739	0.96827466	-0.0093625
ENSG000001	DCAF8	0.89258381	0.96323829	-0.0093394
ENSG000001	MFHAS1	0.91868167	0.97272242	-0.0093097
ENSG000001	ATRNL1	0.98107788	0.9931892	-0.0093048

ENSG000001	ZNF384	0.7016352	0.88523111	-0.0091521
ENSG000001	CTBP1	0.84775217	0.94527257	-0.0091419
ENSG00000C	ASB1	0.90839436	0.96934801	-0.0091238
ENSG000001	PRKCB	0.7463534	0.90662373	-0.0091232
ENSG000001	SEC13	0.84227943	0.9429376	-0.0091049
ENSG000001	EGR1	0.93555414	0.9787753	-0.0090535
ENSG000001	PNLDC1	0.97499452	0.99154366	-0.0090443
ENSG00000C	CAPG	0.88499362	0.96095764	-0.0089921
ENSG000001	CUX2	0.95536085	0.98521399	-0.0089731
ENSG000001	CAVIN2	0.93864332	0.97960866	-0.0089668
ENSG000002	HOXA10	0.97791146	0.99280436	-0.0089161
ENSG000001	PDE7B	0.95883654	0.98631483	-0.0089101
ENSG000001	RNF220	0.87398757	0.95568296	-0.0089098
ENSG000001	FBLN7	0.94576396	0.98299638	-0.0088646
ENSG00000C	GSTO2	0.93381945	0.97838131	-0.0088539
ENSG000001	LZTFL1	0.94144309	0.98082846	-0.0088485
ENSG000001	DDX46	0.86460149	0.95224252	-0.0088145
ENSG000001	CCDC191	0.9424596	0.9813208	-0.0088126
ENSG000001	TERF2	0.84709297	0.94527257	-0.0087814
ENSG000001	UBE2Q1	0.75763597	0.91082077	-0.0087409
ENSG000001	CEL	0.97224148	0.99054307	-0.0087395
ENSG00000C	RBM6	0.87850804	0.95778789	-0.0087248
ENSG000001	WDR26	0.88389822	0.96050431	-0.0087222
ENSG000001	ZNF561	0.92814744	0.97652064	-0.0087215
ENSG000001	RRNAD1	0.81737675	0.93316132	-0.0087028
ENSG000001	FANCD2OS	0.98361798	0.99392119	-0.008677
ENSG00000C	PNPLA6	0.91256134	0.97123107	-0.0086577
ENSG000001	OMG	0.93476287	0.97859224	-0.0086422
ENSG000002	TMEM185A	0.90106388	0.96635233	-0.0086306
ENSG000001	MIEN1	0.9109498	0.97086261	-0.0085413
ENSG000001	TLR8	0.91920083	0.97282387	-0.0085107
ENSG000001	SLC22A13	0.98487447	0.99462139	-0.0085089
ENSG000001	LYST	0.90849658	0.96935737	-0.0084484
ENSG000001	CCDC127	0.92876814	0.97652064	-0.0084128
ENSG000001	PYROXD2	0.92720934	0.9763358	-0.0084093
ENSG000001	SDHAF4	0.93585761	0.97895209	-0.0083633
ENSG000001	SRGN	0.93317019	0.97829811	-0.0083558
ENSG000001	ZNF433	0.95471406	0.98499328	-0.0083395
ENSG000001	DCXR	0.91354061	0.97135384	-0.0083368
ENSG000001	ZNF394	0.80048184	0.92689709	-0.0083263
ENSG00000C	MBD3	0.92547194	0.97525983	-0.0083224
ENSG000001	IFT46	0.93587075	0.97895209	-0.0083122
ENSG000001	ATXN7L2	0.92009617	0.97332735	-0.0083031

ENSG00000C	NR1H3	0.90300221	0.96763453	-0.0082915
ENSG000001	CALML4	0.95424811	0.98494225	-0.00827
ENSG000001	ING4	0.89726807	0.96522986	-0.0082553
ENSG00000C	EXOSC5	0.92402734	0.97481852	-0.0082074
ENSG000001	VEGFA	0.91847217	0.97272242	-0.0082036
ENSG000001	WDR81	0.92577096	0.97536168	-0.0080788
ENSG000001	COPA	0.8281006	0.93660278	-0.0080778
ENSG000001	MRPL4	0.92221328	0.97410521	-0.008054
ENSG000001	TNPO2	0.88355839	0.96044809	-0.0079682
ENSG000001	OLIG1	0.95168513	0.98403516	-0.0079361
ENSG000001	MYOG	0.98601151	0.99508166	-0.0079066
ENSG000002	NAXD	0.90890412	0.96957336	-0.0078996
ENSG000001	EOLA1	0.86891276	0.95366611	-0.007859
ENSG000001	STRIP1	0.81537895	0.93228896	-0.0078495
ENSG000001	SPNS1	0.93772557	0.97941755	-0.0078426
ENSG000001	TBC1D24	0.93969389	0.98007513	-0.0076999
ENSG000001	ORMDL3	0.90996617	0.97015546	-0.0076913
ENSG00000C	PRKAR2B	0.94307494	0.98183559	-0.0076887
ENSG000001	ARPC5	0.827195	0.93649179	-0.0076782
ENSG000001	RCE1	0.91334007	0.97135384	-0.007647
ENSG000001	NOMO1	0.9832895	0.99392119	-0.0076181
ENSG000001	NANOG	0.98753136	0.99544434	-0.0075353
ENSG000001	CHD8	0.8626089	0.951353	-0.0074793
ENSG000001	MADD	0.89492313	0.96412642	-0.0074337
ENSG000001	GAREM2	0.95024546	0.98403516	-0.0074
ENSG000001	C18orf32	0.9216542	0.97390127	-0.0073213
ENSG000001	MUTYH	0.93600118	0.97899067	-0.0072721
ENSG000001	CPNE5	0.92473651	0.97521605	-0.0072701
ENSG00000C	EFNB1	0.93667136	0.97918399	-0.0072365
ENSG00000C	KPNA6	0.86050203	0.95080722	-0.0072319
ENSG000001	MFAP2	0.98483255	0.99462139	-0.0072312
ENSG000001	LILRA1	0.98953184	0.99620755	-0.0071975
ENSG000001	SLC9A5	0.95996109	0.98631483	-0.0071949
ENSG000001	PAQR7	0.93009004	0.97715121	-0.0071353
ENSG000001	MAP3K6	0.94166914	0.98087515	-0.0071344
ENSG000001	EXOC7	0.83486669	0.93977114	-0.0070056
ENSG000002	SMIM26	0.95384417	0.98471311	-0.0069908
ENSG000001	MIA2	0.87639872	0.95679296	-0.0069387
ENSG000001	FIGN	0.98730365	0.99536186	-0.0069359
ENSG000001	PIP4P1	0.86357854	0.95205898	-0.0069103
ENSG000001	DOHH	0.93925461	0.97986875	-0.006901
ENSG000002	RANBP17	0.98614108	0.99508873	-0.0068869
ENSG000001	UBL5	0.94038922	0.98035954	-0.0068806

ENSG000001	SCCPDH	0.93334828	0.97829811	-0.0068569
ENSG000001	CEP295NL	0.95835551	0.98604768	-0.0068188
ENSG000001	GRHPR	0.91677885	0.97237856	-0.0068187
ENSG000001	VPREB1	0.98725958	0.99536186	-0.0067733
ENSG000001	FBXL8	0.9480121	0.98373753	-0.0067416
ENSG000001	DHX30	0.9113894	0.97090539	-0.0067035
ENSG000001	RPL11	0.96602912	0.98812783	-0.0065815
ENSG00000C	RBM5	0.89823222	0.96566178	-0.0065733
ENSG000001	ATXN2L	0.88385624	0.96050431	-0.0065282
ENSG000001	CXADR	0.98581582	0.99500786	-0.0064926
ENSG000001	CACNG8	0.96866981	0.98900043	-0.0064823
ENSG000001	C12orf57	0.96496206	0.98797129	-0.0064265
ENSG000002	RNF103	0.94366807	0.98221647	-0.0064209
ENSG000002	NFS1	0.91949403	0.97307009	-0.0063415
ENSG000001	CARD9	0.93468599	0.97859224	-0.0063346
ENSG000001	SUCLG1	0.91885533	0.97272242	-0.0063256
ENSG000001	EEF1AKMT3	0.95121844	0.98403516	-0.0062946
ENSG000001	HAUS8	0.92851961	0.97652064	-0.0062754
ENSG000001	SAP30L	0.85165875	0.94720831	-0.0062734
ENSG000001	CCDC22	0.92586374	0.97536168	-0.0062527
ENSG000002	CCNL2	0.93111659	0.97753395	-0.006251
ENSG000001	JAGN1	0.91469308	0.97179032	-0.0062466
ENSG00000C	BCL7C	0.94801511	0.98373753	-0.0062399
ENSG000001	HIF1AN	0.86346595	0.9519995	-0.006207
ENSG000001	NHS	0.96521454	0.98797129	-0.0061639
ENSG000001	CHD2	0.87925873	0.95817324	-0.0061386
ENSG000001	ZNF710	0.91440463	0.9716023	-0.0061207
ENSG000001	TARS2	0.92263139	0.97416868	-0.0060958
ENSG000001	ZNF3	0.93454181	0.97859224	-0.0060673
ENSG000002	C22orf39	0.93341491	0.97830221	-0.0060457
ENSG00000C	POMGNT1	0.94211626	0.98113535	-0.0060317
ENSG000001	GPI	0.93482254	0.97859224	-0.0059779
ENSG000001	TACC1	0.907564	0.96883059	-0.005896
ENSG00000C	MBNL3	0.95191168	0.98411089	-0.0058927
ENSG000001	LIG1	0.94772974	0.98373753	-0.0058824
ENSG000001	HSD17B11	0.91309382	0.97123514	-0.0058729
ENSG000002	DENND6B	0.94491717	0.98252902	-0.0058544
ENSG000001	SCNM1	0.90621799	0.96827466	-0.0058116
ENSG00000C	METTL2A	0.95155202	0.98403516	-0.0058101
ENSG000001	LRMDA	0.95555416	0.98528818	-0.0057962
ENSG00000C	ARG2	0.96412457	0.98766502	-0.0057698
ENSG000001	MRPL2	0.93384592	0.97838131	-0.0057683
ENSG000001	C1orf35	0.95192742	0.98411089	-0.0057589

ENSG00000(	GNAO1	0.96672584	0.98843703	-0.0057364
ENSG00000(	SOAT1	0.91978628	0.97312633	-0.0057364
ENSG000001	EBP	0.95336948	0.9845509	-0.0057237
ENSG000001	EIF3D	0.9187129	0.97272242	-0.0056957
ENSG000001	GMEB1	0.8593984	0.950419	-0.0056473
ENSG000001	SNU13	0.93700145	0.97919489	-0.005635
ENSG00000(	CEP170B	0.96432479	0.98777932	-0.0056344
ENSG000001	RPS8	0.96825912	0.98884354	-0.0056285
ENSG000001	QRICH1	0.89820314	0.96566178	-0.0055845
ENSG000001	KLHDC4	0.94794788	0.98373753	-0.0055814
ENSG000001	APEX1	0.95289572	0.9844851	-0.0055713
ENSG000001	C16orf70	0.90016605	0.96608561	-0.0055481
ENSG00000(	PSMC5	0.93526471	0.97866167	-0.0054736
ENSG000001	TMEM143	0.95123228	0.98403516	-0.0054721
ENSG000001	GTF2IRD2B	0.95717287	0.98578332	-0.0054694
ENSG00000(	THAP3	0.93743839	0.97929531	-0.0054569
ENSG000001	TAF10	0.92629378	0.97562867	-0.005449
ENSG00000(	COMT	0.93821347	0.97953782	-0.005379
ENSG000002	N/A	0.9737963	0.99120425	-0.0053186
ENSG000001	HMGN2	0.91623409	0.9722662	-0.0052664
ENSG000001	BEX2	0.97158278	0.99023671	-0.0052496
ENSG000001	GCA	0.95527448	0.98521399	-0.0052179
ENSG000001	EFCAB11	0.95955299	0.98631483	-0.0052102
ENSG000001	FITM2	0.9614313	0.98649866	-0.0051631
ENSG00000(	TP73	0.98353038	0.99392119	-0.0051502
ENSG000001	NFKBIB	0.9305902	0.97729727	-0.0051282
ENSG000001	PYCR1	0.98026574	0.99292419	-0.0051187
ENSG000001	MCL1	0.93658326	0.97918399	-0.0051103
ENSG000001	SESN3	0.96853455	0.98900043	-0.0050874
ENSG000001	UXS1	0.95170635	0.98403516	-0.0050781
ENSG000002	SEC14L6	0.9912676	0.99710148	-0.0050365
ENSG000001	PDE9A	0.97029502	0.9899265	-0.0050173
ENSG000001	ATF4	0.9333261	0.97829811	-0.0049922
ENSG000001	FAM126B	0.95811245	0.98595586	-0.0049715
ENSG000001	LAMTOR2	0.93274845	0.97819097	-0.0049698
ENSG000001	TPI1	0.91855739	0.97272242	-0.0049644
ENSG00000(	SMARCD3	0.91373167	0.97135384	-0.0049397
ENSG000001	GSTM4	0.96076588	0.98640251	-0.0049205
ENSG000001	POLR2G	0.93639613	0.97918399	-0.004883
ENSG000001	RPRD2	0.93074454	0.97733293	-0.0048174
ENSG000001	RAVER2	0.96782372	0.98864761	-0.0048088
ENSG000001	ADGRB1	0.97300561	0.99088636	-0.0047739
ENSG000001	TMEM150A	0.94004935	0.98022609	-0.0047382

ENSG000001	SETMAR	0.96920844	0.98927535	-0.0047298
ENSG000001	CSRP1	0.93520031	0.97865735	-0.0047253
ENSG00000C	UNG	0.95625205	0.98578332	-0.0046859
ENSG00000C	HAUS4	0.93987138	0.98019729	-0.0046706
ENSG000001	SHMT1	0.95351196	0.9845509	-0.0046546
ENSG000001	ESYT1	0.95738932	0.98578332	-0.0045805
ENSG00000C	SPAG9	0.95440751	0.98498155	-0.0045246
ENSG000001	KCNT1	0.98610415	0.99508873	-0.0045056
ENSG000002	IFRD2	0.95345544	0.9845509	-0.0045017
ENSG000001	CYP2D6	0.98660713	0.9951261	-0.0044826
ENSG000001	CCAR2	0.91757632	0.97272242	-0.0044554
ENSG000001	TIMM29	0.93949111	0.97998953	-0.0044499
ENSG000001	MIIP	0.95028838	0.98403516	-0.0044249
ENSG000001	ART1	0.99228091	0.99727564	-0.0043615
ENSG000001	TCP10L2	0.99197258	0.99722988	-0.0043545
ENSG000001	RPL24	0.97832605	0.99287956	-0.0042872
ENSG000001	SH3D19	0.98005521	0.99292419	-0.004245
ENSG000001	YJU2	0.94985297	0.98403516	-0.0042211
ENSG00000C	CTNS	0.9374164	0.97929531	-0.0042138
ENSG000001	SYNGR2	0.9300644	0.97715121	-0.0041919
ENSG000002	PCDHGC3	0.98555169	0.99486495	-0.0041742
ENSG000001	RFTN2	0.9698828	0.9896925	-0.0041318
ENSG000001	CLEC4C	0.96095461	0.98647152	-0.0040676
ENSG000001	MCU	0.92770079	0.97641005	-0.0040165
ENSG000001	LYG2	0.98521832	0.99469772	-0.0039733
ENSG000001	BTBD9	0.93220341	0.97787927	-0.0039576
ENSG000001	CRYBB1	0.99301759	0.99744056	-0.0039425
ENSG000001	RCSD1	0.92090671	0.97363955	-0.0039061
ENSG000001	PDCD11	0.95276616	0.9844851	-0.0038915
ENSG000001	ACSL6	0.98145395	0.99328526	-0.0038397
ENSG00000C	ADAMTS2	0.99271182	0.99738023	-0.0038345
ENSG00000C	NOP16	0.96772494	0.98864761	-0.0037342
ENSG000001	CTIF	0.94980123	0.98403516	-0.0037039
ENSG000001	TBRG4	0.95356577	0.9845509	-0.0036609
ENSG000001	PSME3IP1	0.91592933	0.9722662	-0.0036606
ENSG000001	SIN3A	0.94458396	0.98246171	-0.0036154
ENSG000001	NRDE2	0.94753048	0.98373753	-0.0035992
ENSG000002	ANKHD1-EIF4	0.96481959	0.98797129	-0.0034562
ENSG00000C	VDAC3	0.96562406	0.98812783	-0.0034447
ENSG000001	CLPX	0.94970496	0.98403516	-0.0034263
ENSG000001	GPSM1	0.96709511	0.98843703	-0.0034222
ENSG000001	SERPINE2	0.97444436	0.99135671	-0.0034056
ENSG000002	C8orf82	0.96687264	0.98843703	-0.0033925

ENSG000001	TRAPPC2L	0.97216677	0.99052911	-0.0033325
ENSG000001	WDR55	0.93701237	0.97919489	-0.0033015
ENSG000001	TMED4	0.95672382	0.98578332	-0.0032961
ENSG000001	TOPORS	0.97332271	0.99108497	-0.0032571
ENSG000001	PKDREJ	0.98717589	0.99536186	-0.0032517
ENSG000001	CST3	0.96284964	0.98702997	-0.003193
ENSG000001	FUNDC2	0.97398479	0.99126182	-0.0031806
ENSG000001	TLR4	0.97252231	0.9906427	-0.0031734
ENSG000002	USP51	0.98507984	0.99469772	-0.0031014
ENSG000001	RPRD1B	0.9523158	0.98432437	-0.003079
ENSG000001	CRTC3	0.96105904	0.98648116	-0.003053
ENSG000001	GPR12	0.99207603	0.99723216	-0.0030436
ENSG000001	ZFYVE28	0.97341351	0.99111527	-0.0030161
ENSG000001	KLF13	0.95780125	0.98585223	-0.0030108
ENSG000001	CENPB	0.96136704	0.98649866	-0.0029557
ENSG000001	SLC25A4	0.98301288	0.99392119	-0.0029437
ENSG000001	CENPE	0.98600797	0.99508166	-0.0029411
ENSG000002	TMEM185B	0.96435781	0.98777932	-0.0029232
ENSG00000C	DHX8	0.95456367	0.98499328	-0.0029178
ENSG000002	MTRNR2L3	0.99446418	0.9977832	-0.0028999
ENSG000001	MT1F	0.97891259	0.99291627	-0.0028552
ENSG000001	SELENON	0.95963073	0.98631483	-0.0028329
ENSG000001	R3HDM2	0.93672581	0.97918399	-0.0028153
ENSG000002	SPINK13	0.99540442	0.99826429	-0.0027641
ENSG000001	ZNF165	0.99242001	0.99727564	-0.0027445
ENSG000001	GIT2	0.93033734	0.97723213	-0.0027418
ENSG000001	PRSS16	0.99077502	0.99694194	-0.0027332
ENSG000001	RUNDC1	0.92099843	0.97363955	-0.0027045
ENSG00000C	GIPR	0.98640943	0.9950966	-0.0026795
ENSG000001	EBPL	0.97780821	0.99280436	-0.0025873
ENSG00000C	HDHD5	0.97238111	0.99060647	-0.0025514
ENSG000001	FUT2	0.98779105	0.99544434	-0.0025451
ENSG00000C	TIMM13	0.9769614	0.99278181	-0.0024976
ENSG000001	ETS1	0.98166554	0.99335058	-0.0024853
ENSG000002	N/A	0.99585652	0.99826429	-0.0024247
ENSG000001	EIF4A1	0.9731037	0.9909241	-0.0024221
ENSG000002	SYS1	0.95478286	0.98499328	-0.0024128
ENSG000001	CTBP2	0.96021519	0.98631483	-0.0024
ENSG00000C	ADD2	0.97792896	0.99280436	-0.002374
ENSG000001	CHAF1A	0.96671428	0.98843703	-0.0023125
ENSG000001	TSFM	0.97650333	0.99241714	-0.0022953
ENSG00000C	POU2F2	0.97423671	0.99126968	-0.0022382
ENSG000001	EIF4ENIF1	0.95985786	0.98631483	-0.0022252

ENSG000001	LIX1	0.99189275	0.99722988	-0.0021401
ENSG00000C	ALG1	0.98006914	0.99292419	-0.0021391
ENSG000001	PNPLA3	0.99601579	0.99826429	-0.0021264
ENSG000001	BUD13	0.95087986	0.98403516	-0.0021065
ENSG000001	PARP6	0.97260488	0.99066467	-0.0021016
ENSG000001	DNAJB1	0.96311241	0.98706408	-0.0020939
ENSG000001	ZNF133	0.97850744	0.99291627	-0.00209
ENSG000001	RAB9A	0.97115228	0.99011676	-0.0020629
ENSG000001	MRPL37	0.97420925	0.99126968	-0.0020578
ENSG000001	TRMT1	0.98088901	0.99314408	-0.0020504
ENSG000001	NPM1	0.98805346	0.99553361	-0.0020465
ENSG000001	DPY19L3	0.98768444	0.99544434	-0.0020458
ENSG000001	KCNIP3	0.99566633	0.99826429	-0.0020089
ENSG000001	TMEM248	0.95432664	0.98496069	-0.0019988
ENSG000002	N/A	0.98264804	0.99382718	-0.0019861
ENSG000001	NOC2L	0.97413626	0.99126968	-0.0019692
ENSG000001	RDH5	0.97992074	0.99292419	-0.0019661
ENSG00000C	SNAP23	0.97150193	0.99022454	-0.0019063
ENSG000001	PRMT5	0.96512713	0.98797129	-0.0019041
ENSG000001	B3GALT6	0.98015598	0.99292419	-0.0018479
ENSG000001	B3GALNT1	0.99284573	0.99744056	-0.0018381
ENSG000001	SF3B5	0.97983143	0.99292419	-0.001802
ENSG000001	SLC25A41	0.99639263	0.99848344	-0.0017899
ENSG00000C	NFE2L1	0.96520274	0.98797129	-0.0017703
ENSG000001	ODC1	0.98102508	0.9931892	-0.0017298
ENSG000001	NR1I3	0.99409613	0.9977832	-0.0016761
ENSG000001	PRRC2C	0.97040646	0.98993922	-0.0016613
ENSG000001	CCR3	0.98666843	0.99512612	-0.0016498
ENSG00000C	PTPRH	0.99547035	0.99826429	-0.0016443
ENSG000002	SPDYE3	0.98323015	0.99392119	-0.0016386
ENSG000001	FAM167B	0.99442503	0.9977832	-0.0015937
ENSG000001	KRT73	0.99310941	0.99747108	-0.0015871
ENSG000001	SPINDOC	0.98462057	0.99458953	-0.0015327
ENSG000001	FBXO33	0.98034376	0.99294128	-0.0015197
ENSG000001	KCMF1	0.95274595	0.9844851	-0.001506
ENSG000001	ZNF544	0.99051442	0.99677612	-0.0015039
ENSG000001	VCPKMT	0.98647449	0.9950966	-0.0015035
ENSG000001	TGFB3	0.98922571	0.99620755	-0.0014309
ENSG000001	HYLS1	0.98978292	0.99634874	-0.0013905
ENSG000001	INTS6	0.97727121	0.99280436	-0.0013858
ENSG000001	FBXL14	0.98637905	0.9950966	-0.0013705
ENSG000001	NDUFC1	0.9865166	0.9950966	-0.0013455
ENSG000001	FLNB	0.98773422	0.99544434	-0.0013107

ENSG00000C	FOXN3	0.97482297	0.9914934	-0.001282
ENSG000001	VPS25	0.9832574	0.99392119	-0.0012803
ENSG000001	PSMA7	0.98263492	0.99382718	-0.0012603
ENSG000001	ARHGAP35	0.98449183	0.99453704	-0.001172
ENSG000002	AGPAT1	0.99464303	0.9977832	-0.001167
ENSG000001	STK4	0.97936808	0.99292419	-0.0011608
ENSG000001	DNHD1	0.99121335	0.99710148	-0.0011441
ENSG000001	ENTPD3	0.99793949	0.99917023	-0.0009997
ENSG000001	SLC49A3	0.99256437	0.99729379	-0.0009863
ENSG000001	FAM13A	0.9895813	0.99620755	-0.0009607
ENSG000001	TPO	0.99858161	0.99952354	-0.00095
ENSG000001	SMTN	0.99299819	0.99744056	-0.0009235
ENSG000001	SLTM	0.98799042	0.99553186	-0.0008799
ENSG000001	TBCA	0.99430627	0.9977832	-0.0008763
ENSG00000C	THOC3	0.99192351	0.99722988	-0.0008091
ENSG000001	SMS	0.98856633	0.99586497	-0.0008039
ENSG000001	NUDT14	0.99253808	0.99729379	-0.0007911
ENSG00000C	USP48	0.99181289	0.99722988	-0.0007822
ENSG000001	SMARCC1	0.9852635	0.99469772	-0.0007769
ENSG000001	WDCP	0.99192754	0.99722988	-0.0007575
ENSG000001	ZDHHC5	0.98137268	0.99328526	-0.0007402
ENSG000001	HTR3B	0.99837878	0.99948679	-0.0007401
ENSG000001	LFNG	0.99234297	0.99727564	-0.0006909
ENSG000001	FADS2	0.99447466	0.9977832	-0.0006877
ENSG000001	LRP2BP	0.99676821	0.99855167	-0.0006786
ENSG000001	FBXO44	0.99459017	0.9977832	-0.0006443
ENSG00000C	CUL3	0.98936006	0.99620755	-0.0006186
ENSG000001	TIGD6	0.99597143	0.99826429	-0.0006181
ENSG000001	PPCDC	0.99320561	0.99749572	-0.0005174
ENSG000001	ASGR1	0.99498504	0.99799679	-0.0004974
ENSG00000C	TTC7A	0.99182186	0.99722988	-0.000489
ENSG00000C	BUD23	0.99448159	0.9977832	-0.0004857
ENSG000001	PRADC1	0.99577676	0.99826429	-0.0004814
ENSG000001	SELENOM	0.99734249	0.99875724	-0.0003925
ENSG000001	PSPN	0.99654239	0.99849904	-0.0003585
ENSG00000C	SARS1	0.99575474	0.99826429	-0.0002613
ENSG000001	GHITM	0.99424962	0.9977832	-0.000254
ENSG000001	PPP1R3D	0.99662973	0.99849904	-0.0002515
ENSG000001	PDE3B	0.99894907	0.99965328	-0.0001563
ENSG000001	ZBTB4	0.99847549	0.99952199	-0.0001539
ENSG000001	KAT5	0.99665419	0.99849904	-0.0001486
ENSG000001	TMEM183A	0.99810773	0.99927705	-0.000123
ENSG000001	THADA	0.99877803	0.99961372	-7.16E-05

ENSG000001	ATP5PD	0.9993308	0.99970021	-7.10E-05
ENSG000001	SZT2	0.99904881	0.99965328	-5.92E-05
ENSG000002	TDRD15	0.99997326	0.99997326	1.07E-05
ENSG000001	CACFD1	0.99987027	0.99993185	1.31E-05
ENSG000001	PFKM	0.9998361	0.99993185	1.41E-05
ENSG000001	MAN2C1	0.99980454	0.99993185	1.63E-05
ENSG000001	DNAH1	0.99978088	0.99993185	1.85E-05
ENSG000001	ARMC10	0.99978203	0.99993185	2.86E-05
ENSG000001	KIF1C	0.99881339	0.99961372	7.53E-05
ENSG000001	IL17RC	0.99924347	0.99967445	0.00010501
ENSG000001	MAFF	0.99909918	0.99965328	0.00010837
ENSG000001	PSMD1	0.99784102	0.99913324	0.00011252
ENSG000001	RSBN1L	0.99685756	0.99857957	0.00018292
ENSG000001	ARL4D	0.99860017	0.99952354	0.00019542
ENSG000001	ENTR1	0.99780527	0.99913324	0.00020802
ENSG000001	BRAF	0.99649274	0.99849904	0.00026657
ENSG000001	ZNF274	0.99464919	0.9977832	0.00030079
ENSG000001	ZNF329	0.9972757	0.99875724	0.0003142
ENSG000001	ZSCAN5B	0.99916656	0.99965909	0.00041843
ENSG000001	SSR4	0.99570963	0.99826429	0.00044371
ENSG000001	BRDT	0.99909361	0.99965328	0.00044601
ENSG000001	COPRS	0.99605098	0.99826429	0.00048214
ENSG000001	GDF11	0.99636149	0.99848344	0.00050636
ENSG000001	UBXN7	0.99391887	0.9977832	0.00051202
ENSG000001	PAM	0.99200943	0.99722988	0.00057322
ENSG000001	GMPR2	0.98626034	0.9950966	0.00062281
ENSG000001	MSS51	0.99359085	0.99770777	0.00066231
ENSG000001	SPECC1	0.98874407	0.99598223	0.00067928
ENSG000001	HLF	0.99733038	0.99875724	0.00068168
ENSG000001	SFXN2	0.99586495	0.99826429	0.00069802
ENSG000002	CCNQ	0.99375158	0.99774576	0.00070395
ENSG000001	KDELR2	0.98856617	0.99586497	0.00070771
ENSG000001	DHRS4L2	0.99332451	0.99750201	0.00073875
ENSG000001	HSPBP1	0.99213453	0.99723216	0.00078542
ENSG000001	KLHL12	0.98562447	0.99487657	0.00081979
ENSG000002	HSBP1L1	0.99325681	0.99749572	0.00084534
ENSG000001	GSS	0.98997854	0.99648388	0.00090358
ENSG000001	RPL4	0.99475717	0.99782987	0.00092057
ENSG000001	ZFP36L2	0.98950311	0.99620755	0.00094791
ENSG000002	DYNLL2	0.98091098	0.99314408	0.00106404
ENSG00000(	DOCK3	0.99409222	0.9977832	0.00106513
ENSG000001	DLST	0.98196543	0.99341071	0.00109503
ENSG000001	HS2ST1	0.99113533	0.99710148	0.00109571

ENSG000001	PPP1R13L	0.99242346	0.99727564	0.00113329
ENSG000001	ARL11	0.98362789	0.99392119	0.00115707
ENSG000001	RPL28	0.98553774	0.99486495	0.00132482
ENSG000001	ASB8	0.95965779	0.98631483	0.00133823
ENSG000001	POLR1E	0.99012805	0.9965726	0.0013809
ENSG000001	RMND5B	0.97906042	0.99292419	0.00138383
ENSG00000C	TPR	0.97968405	0.99292419	0.00140225
ENSG000001	ABT1	0.9781427	0.99280436	0.00141708
ENSG000001	TRIP4	0.98229571	0.99367862	0.00142871
ENSG000001	LSM10	0.97377648	0.99120425	0.00143792
ENSG000002	SLC35F6	0.97009955	0.98985147	0.00144684
ENSG000001	CABP1	0.99693107	0.9985916	0.00150593
ENSG000001	ATOH7	0.99720483	0.99875724	0.00152737
ENSG000001	PIH1D1	0.98377973	0.99400321	0.00155204
ENSG000001	AHNAK	0.98733129	0.99536186	0.00155421
ENSG00000C	OSGEP	0.98021037	0.99292419	0.00161509
ENSG000001	TRIM7	0.98920805	0.99620755	0.00167951
ENSG00000C	EDN1	0.99297745	0.99744056	0.00168476
ENSG000001	EP400	0.98163963	0.99335058	0.00170247
ENSG000001	SLC35D2	0.98044415	0.99298101	0.00173295
ENSG000001	TTC28	0.98686553	0.99526308	0.00178715
ENSG000001	TNFAIP1	0.95100407	0.98403516	0.00178774
ENSG00000C	FECH	0.9888636	0.99604083	0.001826
ENSG000001	STING1	0.97844169	0.99291627	0.0018616
ENSG00000C	ZFY	0.98270426	0.99382718	0.00187775
ENSG000002	LYRM9	0.98363735	0.99392119	0.00189811
ENSG000001	URAD	0.99590651	0.99826429	0.00189909
ENSG000001	NFKB1	0.96713057	0.98843703	0.00196523
ENSG000001	WBP4	0.98147864	0.99328526	0.00199609
ENSG000001	PRDM2	0.97269445	0.99069375	0.00201542
ENSG000001	DOT1L	0.97779608	0.99280436	0.00201764
ENSG000001	CUBN	0.98949244	0.99620755	0.0020593
ENSG000001	FBL	0.9846663	0.99458953	0.00205984
ENSG000001	TOB2	0.96953464	0.98939941	0.00206072
ENSG000002	FAM174C	0.98013433	0.99292419	0.00208493
ENSG000001	ANKLE1	0.97890465	0.99291627	0.00208834
ENSG000001	P4HA1	0.98334892	0.99392119	0.0021271
ENSG000001	ADCY9	0.98006119	0.99292419	0.00214014
ENSG000001	TBC1D20	0.93676879	0.97918399	0.00215252
ENSG00000C	HDAC6	0.96054696	0.98636485	0.00219505
ENSG000001	YPEL2	0.97814983	0.99280436	0.00229873
ENSG000001	DNM3	0.98274819	0.99382718	0.00231559
ENSG000001	CCDC47	0.96923029	0.98927535	0.00233635

ENSG000001	LONP1	0.97103015	0.99011676	0.0023435
ENSG000001	EIF3G	0.97505891	0.99154704	0.00234968
ENSG000001	PRPF19	0.9687173	0.98900043	0.0023578
ENSG000001	AHCYL1	0.91914334	0.97282387	0.00236163
ENSG000001	HNRNPU	0.94858287	0.98384797	0.00242356
ENSG000001	RC3H1	0.97386718	0.99120425	0.00244275
ENSG000001	TNFAIP8L1	0.97640657	0.99241714	0.00244677
ENSG000001	RPA1	0.96252492	0.98683571	0.00259125
ENSG000001	BCL11B	0.98189143	0.99341071	0.00268703
ENSG000001	ZNF33A	0.97986938	0.99292419	0.00274911
ENSG000001	HDAC1	0.95913808	0.98631483	0.00275748
ENSG000001	MFSD12	0.9623439	0.98677473	0.00276665
ENSG00000C	ABLIM1	0.97957373	0.99292419	0.0027765
ENSG000002	SMIM7	0.97063788	0.98995829	0.00277849
ENSG000001	PDCD5	0.977864	0.99280436	0.00277868
ENSG000002	N/A	0.99049696	0.99677612	0.00279599
ENSG000001	IQCH	0.99366307	0.99771858	0.00284297
ENSG000001	PECR	0.97909208	0.99292419	0.00284624
ENSG000002	N/A	0.99581594	0.99826429	0.0028834
ENSG000001	PRRG4	0.98314124	0.99392119	0.00288569
ENSG000001	YLPM1	0.95596787	0.98558958	0.00289707
ENSG000001	ATP13A2	0.96567764	0.98812783	0.00296818
ENSG000001	ZNF565	0.97967798	0.99292419	0.00301298
ENSG000001	CACNA1H	0.98488161	0.99462139	0.00304496
ENSG00000C	LZTR1	0.96940792	0.98933227	0.00304669
ENSG000001	EMC10	0.96513003	0.98797129	0.00305326
ENSG000001	PIP4K2C	0.93048677	0.97729727	0.00306572
ENSG000001	RUVBL1	0.97183145	0.99027388	0.00311305
ENSG000001	CCDC15	0.97774923	0.99280436	0.00314355
ENSG000001	CEP72	0.98057589	0.9930525	0.00314636
ENSG00000C	HOXA9	0.98947354	0.99620755	0.00315347
ENSG000001	TWNK	0.96598961	0.98812783	0.00315572
ENSG000001	TAGLN	0.95746703	0.98578332	0.00319527
ENSG000002	TRIM27	0.99391387	0.9977832	0.00322613
ENSG000001	ASCL3	0.99119944	0.99710148	0.00322909
ENSG000001	FBXL6	0.96654384	0.98843703	0.00324875
ENSG000001	PXDC1	0.97185529	0.99027388	0.00326239
ENSG000001	SLC3A2	0.95748809	0.98578332	0.00326808
ENSG000002	STUM	0.99429896	0.9977832	0.003295
ENSG000001	NFXL1	0.97416502	0.99126968	0.00331063
ENSG000001	EMP3	0.95843718	0.98606923	0.00331744
ENSG00000C	CAPN3	0.96666853	0.98843703	0.00339727
ENSG000001	TCTN3	0.96318656	0.98707777	0.00343115

ENSG000001	BLOC1S1	0.95945869	0.98631483	0.00346048
ENSG000001	OLFML3	0.99174078	0.99722988	0.0034775
ENSG000001	CC2D1B	0.93785668	0.97941755	0.00347895
ENSG000002	IL10RB	0.95794524	0.98593793	0.00352355
ENSG000001	SRR	0.97169684	0.99023671	0.00353437
ENSG000001	PRSS33	0.98627703	0.9950966	0.00353437
ENSG000001	MICU1	0.95749146	0.98578332	0.0035439
ENSG000001	PRDM8	0.96218206	0.98675362	0.00357516
ENSG000001	MMP17	0.96800865	0.98871211	0.00360835
ENSG000001	SPATA25	0.98408763	0.99425243	0.00361109
ENSG000001	XRCC2	0.990802	0.99694194	0.00362029
ENSG000001	LAMTOR5	0.9546848	0.98499328	0.00362224
ENSG000002	ZNF407	0.93801308	0.9794784	0.00364105
ENSG00000C	SLC22A16	0.97700686	0.99278181	0.00372748
ENSG000001	FIP1L1	0.93667925	0.97918399	0.00373895
ENSG00000C	CMTM1	0.98008566	0.99292419	0.00375911
ENSG000001	ASXL1	0.95367495	0.98460101	0.00377906
ENSG000001	SEZ6L	0.97735334	0.99280436	0.00380301
ENSG000001	GCDH	0.95720876	0.98578332	0.00383249
ENSG000001	COMMD5	0.90577367	0.96827466	0.00386302
ENSG000001	ALPK2	0.98936914	0.99620755	0.0038788
ENSG000001	SLC13A4	0.9614671	0.98649866	0.00389499
ENSG000001	TARBP2	0.96340761	0.98717969	0.00393565
ENSG000001	XPR1	0.942199	0.98113535	0.0039358
ENSG000001	SMG9	0.95097582	0.98403516	0.00395803
ENSG00000C	HNRNPC	0.94966424	0.98403516	0.00396879
ENSG000001	DPP9	0.92824062	0.97652064	0.00399433
ENSG00000C	POLD3	0.88185297	0.95932126	0.0040711
ENSG000001	MTG1	0.96916903	0.98927535	0.00409723
ENSG000002	SMIM33	0.99195642	0.99722988	0.00410423
ENSG00000C	LETMD1	0.96127164	0.98649866	0.0041293
ENSG000001	ARMCX4	0.97054794	0.98995829	0.00419
ENSG000002	STPG4	0.98522905	0.99469772	0.00419634
ENSG000001	EIF2B4	0.95087607	0.98403516	0.0042076
ENSG000001	PHYH	0.96442484	0.98778567	0.00430246
ENSG000001	NAPSA	0.97527059	0.99170018	0.00432515
ENSG000001	CCNA2	0.9793684	0.99292419	0.00435067
ENSG00000C	CACNB1	0.96274454	0.98699855	0.00437613
ENSG000001	YPEL5	0.95450165	0.98499328	0.00449332
ENSG000001	PLIN2	0.94824164	0.98373753	0.0045904
ENSG000001	TMEM79	0.93496338	0.97859224	0.00459952
ENSG000001	KRTCAP2	0.96143561	0.98649866	0.00461196
ENSG000001	SPCS1	0.94539936	0.98278631	0.00467815

ENSG00000(	WBP11	0.89126062	0.9626008	0.00469158
ENSG000001	ALDH1A2	0.97347467	0.99111539	0.00470561
ENSG000001	FAM217B	0.95113126	0.98403516	0.00472834
ENSG000001	C11orf49	0.95156059	0.98403516	0.00474398
ENSG000002	DHFR	0.96001918	0.98631483	0.00475327
ENSG000001	SNX33	0.95109838	0.98403516	0.00475756
ENSG000002	LYN	0.95818234	0.98595586	0.00476846
ENSG000001	RAI1	0.95311696	0.9845509	0.00477549
ENSG000002	ISY1	0.91758551	0.97272242	0.00478086
ENSG000001	FBXW12	0.98770095	0.99544434	0.00479553
ENSG000001	RPS6KA4	0.94496404	0.98252902	0.00486869
ENSG000002	EIF6	0.93693686	0.97919489	0.0048706
ENSG000001	C9orf40	0.96194358	0.9866758	0.00488298
ENSG000002	KRTAP10-10	0.99039596	0.99677612	0.00488538
ENSG000001	RTN4	0.91231915	0.97123107	0.00490704
ENSG000001	MYL12B	0.94552501	0.98281094	0.0049694
ENSG000001	JAG2	0.96252282	0.98683571	0.005005
ENSG000001	GPR35	0.94302043	0.98183559	0.00500921
ENSG000001	ABLIM2	0.97890089	0.99291627	0.00502509
ENSG000001	PID1	0.95530303	0.98521399	0.00502582
ENSG00000(	ATG2B	0.95680836	0.98578332	0.00503043
ENSG000001	THEM4	0.97042943	0.98993922	0.00504319
ENSG000002	TRIM49D2	0.99091584	0.9969947	0.00505122
ENSG000001	SMPDL3A	0.95159346	0.98403516	0.00505395
ENSG00000(	BRAP	0.85448081	0.94818753	0.00507912
ENSG000001	BLMH	0.9484158	0.98373753	0.0050923
ENSG000001	CHMP7	0.95081903	0.98403516	0.00512459
ENSG000001	CLSTN1	0.94118571	0.9807491	0.00517843
ENSG000001	PER2	0.91622583	0.9722662	0.00523665
ENSG00000(	UBE2D1	0.94919983	0.98403516	0.00524476
ENSG000001	HAS3	0.95686688	0.98578332	0.00525867
ENSG000001	RAPGEF6	0.95212553	0.98419036	0.00526693
ENSG000001	ESD	0.94544079	0.98278631	0.00531914
ENSG000001	ACIN1	0.85987663	0.95062414	0.00534451
ENSG000001	DCAF11	0.91642657	0.9722662	0.00534656
ENSG000001	UBASH3A	0.96179643	0.98664952	0.00538195
ENSG000001	TRERF1	0.94014095	0.98022659	0.00539283
ENSG00000(	CHFR	0.84367997	0.94370569	0.00543523
ENSG000001	PDPK1	0.94097503	0.98067223	0.00544496
ENSG00000(	TOX4	0.76882223	0.91372907	0.00547467
ENSG000001	TBC1D9	0.95243724	0.98438724	0.00551624
ENSG00000(	LRRC7	0.97934243	0.99292419	0.00552692
ENSG000001	KCND1	0.95163277	0.98403516	0.00557234

ENSG000001	FPGS	0.9474914	0.98373753	0.00557346
ENSG000002	N/A	0.99135157	0.99712417	0.00564649
ENSG000001	SH3BP4	0.95743051	0.98578332	0.00566236
ENSG000001	AMFR	0.886261	0.96095764	0.00566612
ENSG00000C	ZNF263	0.92678146	0.97601184	0.00567507
ENSG000001	UBAP2L	0.85572654	0.9486844	0.00569519
ENSG000001	RUVBL2	0.9343179	0.97850748	0.00572808
ENSG000001	ANKRD50	0.96064577	0.98640251	0.00574157
ENSG000001	DST	0.94824223	0.98373753	0.00575029
ENSG000001	BLOC1S4	0.93741215	0.97929531	0.00575396
ENSG000002	PRSS51	0.976177	0.99235759	0.00575534
ENSG000001	CDK4	0.94185361	0.98094144	0.00576176
ENSG00000C	MRPL43	0.935904	0.97895209	0.00579302
ENSG000001	STK25	0.93166946	0.97763745	0.0058549
ENSG000001	DOLK	0.94388877	0.98221647	0.00588713
ENSG000001	MAP2K1	0.88701315	0.96095764	0.00592689
ENSG00000C	PPP2R3C	0.93147904	0.97763745	0.00597156
ENSG000001	NHP2	0.9406925	0.9805498	0.00606924
ENSG00000C	MKRN2	0.92753892	0.97641005	0.00608567
ENSG000001	BSDC1	0.86839252	0.95343718	0.00611242
ENSG00000C	TBC1D22B	0.91202843	0.97118348	0.00611778
ENSG000001	ELAVL3	0.98729094	0.99536186	0.0061184
ENSG000001	AIFM1	0.90318296	0.96763453	0.00612104
ENSG000001	PLOD3	0.91285413	0.97123107	0.00613436
ENSG000002	SIAH3	0.98507445	0.99469772	0.00614877
ENSG000001	MICOS10	0.9439575	0.98221647	0.00618395
ENSG000001	CRELD1	0.92958719	0.97687575	0.00619065
ENSG000001	VIPAS39	0.884747	0.96095764	0.00619292
ENSG000001	NOC4L	0.93375781	0.97838131	0.00619395
ENSG000001	TM9SF2	0.90053126	0.96608561	0.00622757
ENSG000001	ANO7	0.98726233	0.99536186	0.00623426
ENSG000001	NRM	0.97720589	0.99280436	0.00624542
ENSG000001	ADCK2	0.92629744	0.97562867	0.00627033
ENSG000001	NUTM2G	0.95771453	0.98585223	0.00627844
ENSG000001	ZNF576	0.93900247	0.97979455	0.00629535
ENSG000002	FANCG	0.94354586	0.9821999	0.00630025
ENSG000001	IL7R	0.96866008	0.98900043	0.00633956
ENSG000001	PTCH1	0.96677346	0.98843703	0.00635913
ENSG000001	LLGL1	0.92077774	0.97360588	0.00636502
ENSG000001	PCNT	0.93300042	0.97819271	0.00640411
ENSG000001	SLC28A3	0.97879161	0.99291627	0.00640457
ENSG000001	ZNF786	0.93839686	0.97954033	0.00640972
ENSG000001	PCCA	0.93675464	0.97918399	0.00642886

ENSG00000(	ANKRD13A	0.90596534	0.96827466	0.00645583
ENSG000001	DGKQ	0.93207816	0.97784797	0.00646806
ENSG00000(	LRP6	0.97121551	0.99011906	0.00649977
ENSG000001	DCAF4	0.93717048	0.97920445	0.00650699
ENSG000001	LRRC37A	0.97461378	0.99140483	0.00653844
ENSG000001	BOK	0.96336447	0.98717969	0.00653893
ENSG000001	BLCAP	0.89145821	0.96270496	0.00654606
ENSG000001	TMEM59	0.90321297	0.96763453	0.00655686
ENSG000001	INO80C	0.89349677	0.96383916	0.00657374
ENSG000001	RAP1A	0.91434763	0.9716023	0.00658095
ENSG000002	N/A	0.98784225	0.99544434	0.00658548
ENSG000001	YWHAB	0.85463599	0.94818753	0.00660035
ENSG000001	TAF8	0.88923017	0.96166446	0.0066319
ENSG000001	ATF7IP	0.93057758	0.97729727	0.00665008
ENSG000001	AGFG1	0.88797794	0.96159657	0.00666003
ENSG000001	ARRDC3	0.94710201	0.98350571	0.00668482
ENSG000002	RCC1L	0.92186843	0.97393309	0.00668584
ENSG000001	RBBP5	0.85252091	0.94733063	0.00680834
ENSG000001	SBK1	0.94153351	0.98085972	0.00684163
ENSG000001	ALDH1B1	0.93407745	0.97846361	0.00686325
ENSG000001	SMARCA1	0.97763364	0.99280436	0.00688195
ENSG000001	MON1B	0.79534476	0.92462665	0.00689565
ENSG000001	TACSTD2	0.95975145	0.98631483	0.00693435
ENSG000001	ATRAID	0.92160676	0.97390127	0.00694081
ENSG00000(	LAS1L	0.905336	0.96827466	0.00695486
ENSG00000(	MED29	0.86069634	0.95080722	0.00695869
ENSG000002	PEDS1	0.91637753	0.9722662	0.00702293
ENSG000001	TNFAIP8L3	0.98846688	0.99586497	0.00704155
ENSG000001	COPS7A	0.84159415	0.94283868	0.00704843
ENSG000001	HNRNPL	0.87657747	0.95679296	0.0070562
ENSG000001	SLC25A38	0.92239131	0.97410521	0.00708662
ENSG000001	GEMIN6	0.95155596	0.98403516	0.00710544
ENSG000001	SKAP1	0.95587206	0.98555338	0.00712084
ENSG000001	RSPH1	0.98140767	0.99328526	0.0071456
ENSG000001	BAZ2B	0.90680342	0.96841405	0.007161
ENSG000001	PGRMC1	0.92868067	0.97652064	0.00716992
ENSG000001	ELAPOR2	0.96035202	0.98631483	0.00718626
ENSG00000(	ANGEL1	0.92444738	0.97507159	0.00722576
ENSG00000(	SNRPA	0.91113423	0.97086554	0.00723463
ENSG000001	FAM241B	0.96359873	0.98725092	0.00727988
ENSG000002	KCTD11	0.91651822	0.9722662	0.00731577
ENSG000001	ARL2BP	0.94637813	0.98315624	0.00733483
ENSG000001	SPIN2B	0.9481823	0.98373753	0.00734841

ENSG000001	GTF2A2	0.91975867	0.97312633	0.00735122
ENSG000001	HARS1	0.90570325	0.96827466	0.00735681
ENSG000001	RPSA	0.95706388	0.98578332	0.00737803
ENSG000001	MALSU1	0.89973353	0.96608561	0.00740657
ENSG000001	MYO1G	0.9043834	0.9680581	0.00744794
ENSG000002	PINX1	0.93704072	0.97919489	0.00754924
ENSG000001	RSPH10B	0.96155697	0.98652853	0.00755767
ENSG000001	CLEC14A	0.98196969	0.99341071	0.00761926
ENSG000001	ASPN	0.98274861	0.99382718	0.00761949
ENSG000001	CYB561A3	0.86791186	0.95327639	0.00762743
ENSG000001	CLEC1B	0.92117616	0.97370857	0.00765712
ENSG00000C	CIAPIN1	0.90998754	0.97015546	0.00766938
ENSG000001	FBXO2	0.95287334	0.9844851	0.0077284
ENSG000001	BTBD8	0.96449618	0.98779642	0.00783428
ENSG00000C	PLAUR	0.9443354	0.98233785	0.00783696
ENSG000002	POU5F1B	0.95704458	0.98578332	0.00787642
ENSG000002	PTTG2	0.96379768	0.98739245	0.00792032
ENSG000001	DDX6	0.90003555	0.96608561	0.00792189
ENSG000001	TMEM243	0.94662202	0.98320732	0.00797163
ENSG000001	TMEM63A	0.913724	0.97135384	0.00798131
ENSG000001	DAB2	0.92933564	0.97675413	0.00800981
ENSG000001	XRRA1	0.89293655	0.96342686	0.00802634
ENSG000001	FAM155B	0.98766849	0.99544434	0.00804015
ENSG00000C	ASIC4	0.98433335	0.99443882	0.00804197
ENSG000001	ABITRAM	0.90435633	0.9680581	0.0080493
ENSG000001	MTA2	0.87668153	0.9567976	0.00807965
ENSG000001	PXMP2	0.96538087	0.98801697	0.00809028
ENSG000001	RRP9	0.91770524	0.97272242	0.00815164
ENSG000001	COPS9	0.94818036	0.98373753	0.00817881
ENSG00000C	HERPUD1	0.78610326	0.92105345	0.0082229
ENSG000001	SPOUT1	0.90830925	0.96932088	0.00822804
ENSG000001	IDH3B	0.88866364	0.96164016	0.00823651
ENSG000001	SNF8	0.86446166	0.95224252	0.00826893
ENSG000001	BLOC1S3	0.87443864	0.95579655	0.00827543
ENSG00000C	PTBP1	0.83781195	0.94142232	0.00829759
ENSG000001	ASB7	0.89553343	0.96443433	0.00830666
ENSG000001	FRMD5	0.97475782	0.99148924	0.00834115
ENSG000001	FXN	0.94008011	0.98022609	0.0083765
ENSG000001	MTMR12	0.91140851	0.97090539	0.00839049
ENSG000001	RPA2	0.8597622	0.95062414	0.00839442
ENSG000001	NUTF2	0.90573228	0.96827466	0.00840965
ENSG000001	KCNRG	0.94615375	0.98315624	0.00845533
ENSG000001	TAFA1	0.97494961	0.99154366	0.00845813

ENSG00000C	NUDC	0.87046189	0.95426978	0.00848685
ENSG000001	GSTM1	0.93433392	0.97850748	0.00848719
ENSG000001	MRPS12	0.92913397	0.97671555	0.00851262
ENSG000002	ZNF616	0.92069919	0.97360588	0.00856309
ENSG000001	CNBP	0.87155343	0.95449478	0.00859789
ENSG00000C	ITIH4	0.94973293	0.98403516	0.00862015
ENSG000001	RNF41	0.71208246	0.89093319	0.00863946
ENSG000001	DHRS1	0.88173699	0.95932126	0.00871933
ENSG000001	MRPL57	0.91864041	0.97272242	0.00874019
ENSG00000C	DELE1	0.87232252	0.95459934	0.00877281
ENSG000001	FDPS	0.86096801	0.95080722	0.00883048
ENSG000001	TOE1	0.89128167	0.9626008	0.00883251
ENSG000001	PRPSAP2	0.90981572	0.97015546	0.00887376
ENSG000002	LBH	0.93292533	0.97819271	0.00891478
ENSG000002	BOP1	0.91597524	0.9722662	0.00893401
ENSG000001	SLC31A2	0.94448035	0.98241688	0.0089375
ENSG000002	MYCBP	0.87525351	0.95634141	0.00895797
ENSG000001	NECTIN2	0.94377271	0.98221647	0.0089902
ENSG000001	NCAPG2	0.93335074	0.97829811	0.0089977
ENSG00000C	KMT2E	0.80218891	0.92757225	0.00902359
ENSG000001	DGLUCY	0.86442066	0.95224252	0.00906531
ENSG000002	ATP5MF	0.9150626	0.97199212	0.00906992
ENSG00000C	TIPIN	0.94684738	0.98330418	0.00907175
ENSG000001	DYM	0.87005996	0.95423131	0.00909895
ENSG00000C	POLR3B	0.8887532	0.96164016	0.00913877
ENSG000002	FER1L6	0.97288636	0.99082706	0.00914291
ENSG000001	VPREB3	0.93300921	0.97819271	0.00915378
ENSG000001	TTC31	0.8649339	0.95228442	0.00916463
ENSG000001	BORCS5	0.90234306	0.96736749	0.0091709
ENSG00000C	TAB2	0.89019609	0.96183364	0.00920756
ENSG000001	GON4L	0.81290682	0.93170624	0.00920801
ENSG000001	IBA57	0.89746194	0.96522986	0.00924418
ENSG00000C	KIF9	0.92275377	0.97417119	0.00937453
ENSG000001	CBX6	0.83934931	0.94217178	0.00945303
ENSG000001	TTC7B	0.92497782	0.97521605	0.00946151
ENSG000002	N/A	0.97627113	0.99235759	0.00950045
ENSG000001	RBL2	0.91108102	0.97086554	0.00951425
ENSG000002	RAB44	0.92844578	0.97652064	0.00953038
ENSG000001	PCYT2	0.91352897	0.97135384	0.00953496
ENSG000001	SDHAF3	0.92662393	0.97590924	0.00953515
ENSG000001	OGFOD2	0.94722551	0.98357105	0.00955956
ENSG000001	PSMD3	0.82762736	0.93660278	0.00956697
ENSG000001	GGN	0.96528163	0.98797768	0.00956701

ENSG000001	EYA3	0.81608755	0.93251163	0.00956888
ENSG00000C	RTRAF	0.91868327	0.97272242	0.00958218
ENSG000001	KLHL26	0.88976204	0.96183364	0.00958674
ENSG000001	IGSF8	0.91651792	0.9722662	0.0096055
ENSG000001	GNL3L	0.80489246	0.92897486	0.00964106
ENSG000001	AP2A2	0.9569903	0.98578332	0.00964487
ENSG000001	ZCCHC24	0.87792167	0.95753437	0.00968508
ENSG000001	AKIRIN2	0.86472768	0.95224252	0.00968896
ENSG000001	RCAN2	0.97385523	0.99120425	0.00969483
ENSG00000C	OAT	0.89012517	0.96183364	0.00972691
ENSG00000C	SNX24	0.92768641	0.97641005	0.00975599
ENSG000001	STK38	0.70605287	0.88780148	0.0097675
ENSG000001	CCR9	0.95091903	0.98403516	0.00978323
ENSG000001	UTP11	0.88268785	0.95995999	0.00981527
ENSG000001	EPHA2	0.95927298	0.98631483	0.00983911
ENSG000001	CKAP5	0.86386237	0.95211331	0.00985141
ENSG000001	CALM1	0.84418037	0.94396799	0.00990204
ENSG000001	DHDDS	0.83903755	0.94195206	0.0099175
ENSG00000C	SESN1	0.93856281	0.97958761	0.00993422
ENSG00000C	CELF2	0.83774639	0.94141381	0.0099425
ENSG000001	PDP2	0.93130451	0.97763745	0.00999416
ENSG000001	SLC37A2	0.87197874	0.95457921	0.01003806
ENSG00000C	PRSS21	0.94465559	0.98247328	0.01004225
ENSG000001	PSMG3	0.91010615	0.97021821	0.01010093
ENSG00000C	CTPS2	0.91259827	0.97123107	0.01010874
ENSG000001	GMPPA	0.87108311	0.95443528	0.01015092
ENSG000001	FLRT1	0.970692	0.98995829	0.01020958
ENSG000001	NUP85	0.86019138	0.95064852	0.0102111
ENSG000001	SGK2	0.96861717	0.98900043	0.01023972
ENSG000002	RASSF5	0.7789337	0.91815219	0.01024477
ENSG000001	CTBS	0.89005619	0.96183364	0.01025271
ENSG00000C	GSK3B	0.85092584	0.94684256	0.01029391
ENSG000001	CD53	0.79916568	0.92613327	0.01030486
ENSG000001	LRRC28	0.87966184	0.95827369	0.01031672
ENSG00000C	HSPA5	0.84060842	0.94254257	0.01031728
ENSG000001	TGIF1	0.90469513	0.96810345	0.01033453
ENSG000001	AP5S1	0.86559716	0.95237042	0.01038501
ENSG000001	MRPL10	0.86422588	0.95224252	0.01040974
ENSG000001	BAHD1	0.86113797	0.95080722	0.01042842
ENSG000002	TSTD3	0.97603635	0.99229242	0.01043028
ENSG000001	PMPCB	0.88921173	0.96166446	0.01047467
ENSG000001	CADM3	0.97112017	0.99011676	0.01048982
ENSG00000C	MAST4	0.92441626	0.97507159	0.01050071

ENSG000001	JTB	0.87434188	0.95579655	0.01056062
ENSG000001	POLR1G	0.93359915	0.97838131	0.01058486
ENSG00000(	PSMD8	0.85116939	0.9469296	0.01058665
ENSG000001	MVB12A	0.87096449	0.95437279	0.01059378
ENSG000001	HR	0.98024389	0.99292419	0.01061584
ENSG000001	ANXA6	0.88359487	0.96044809	0.01069953
ENSG000001	CCDC149	0.86080153	0.95080722	0.01070463
ENSG000001	SLC25A18	0.96220176	0.98675362	0.01071491
ENSG000001	PTPRE	0.81085967	0.93130993	0.0107183
ENSG000001	DMAC2	0.8884073	0.96164016	0.01077414
ENSG00000(	CCN5	0.97871115	0.99291627	0.01077652
ENSG000001	POLDIP3	0.7268968	0.89706038	0.01079091
ENSG000001	P4HA3	0.9828267	0.99384425	0.01082795
ENSG000001	BPTF	0.84468449	0.94417885	0.01085926
ENSG00000(	RBM22	0.60516802	0.83940826	0.01086333
ENSG000001	SAE1	0.88038604	0.95848385	0.01088154
ENSG000001	SLC25A20	0.89593385	0.9646736	0.01088173
ENSG000001	TXN2	0.85070945	0.94684256	0.01089669
ENSG000001	KRT10	0.90729232	0.96868131	0.01096675
ENSG000001	C7orf25	0.95315813	0.9845509	0.01117091
ENSG000001	PEF1	0.80261597	0.92788006	0.01117352
ENSG000001	SNX8	0.8429128	0.94328484	0.01121527
ENSG000001	RETREG1	0.93491637	0.97859224	0.01121652
ENSG000002	TMEM249	0.97951481	0.99292419	0.01121716
ENSG000001	GDPGP1	0.91484067	0.97188353	0.0112479
ENSG00000(	WHRN	0.88715618	0.96095764	0.01125261
ENSG000001	CUZD1	0.93101876	0.97749444	0.01129875
ENSG000001	WDR97	0.92150172	0.97390127	0.01135319
ENSG000001	VSIG10	0.86017308	0.95064852	0.01139892
ENSG000001	ZNF143	0.78729354	0.9215171	0.0114166
ENSG000001	TRAF2	0.87037374	0.95426978	0.0114243
ENSG000001	EIF4G1	0.84142993	0.94283868	0.01142832
ENSG000001	ARL15	0.84029487	0.94245133	0.01145363
ENSG000001	PEX14	0.87934177	0.95817324	0.01146094
ENSG00000(	HNRNPM	0.80926818	0.93027167	0.01149207
ENSG000001	KAT6B	0.88682346	0.96095764	0.01150498
ENSG000001	ENC1	0.86642791	0.95264796	0.01151644
ENSG000001	ALDH5A1	0.8997247	0.96608561	0.01152632
ENSG000001	GKAP1	0.94968924	0.98403516	0.0115285
ENSG000001	CDR2	0.91870957	0.97272242	0.01153374
ENSG00000(	PSMC4	0.83708449	0.94119112	0.01156029
ENSG000001	ZNF875	0.89965069	0.96608561	0.01162501
ENSG000001	TUBD1	0.90067891	0.96608561	0.01163915

ENSG00000(	SLC23A2	0.89412517	0.96404358	0.01169558
ENSG000001	RRAGA	0.693531	0.88183499	0.01187624
ENSG000001	MARCHF3	0.86728301	0.9528412	0.01188608
ENSG000001	OXSR1	0.77513226	0.9163989	0.01190727
ENSG000001	TMEM203	0.84008628	0.94245133	0.01192478
ENSG000001	CYTH1	0.7483825	0.90731063	0.01196966
ENSG000001	GAS8	0.91383409	0.97135384	0.01206736
ENSG000001	POLE	0.85093937	0.94684256	0.01211574
ENSG000001	NEUROG3	0.96762605	0.98864761	0.01213405
ENSG000001	DTX1	0.90464628	0.96810345	0.01216312
ENSG00000(	RANBP1	0.88696942	0.96095764	0.01216531
ENSG000001	KANK3	0.94097503	0.98067223	0.01217539
ENSG00000(	EIPR1	0.84305761	0.94333447	0.0121876
ENSG000002	TMEM199	0.6892403	0.88074719	0.01219274
ENSG000001	DDX42	0.82297772	0.93539963	0.0122009
ENSG000001	COQ7	0.8003076	0.92689709	0.01223725
ENSG000001	CD3G	0.92997154	0.97715121	0.01227001
ENSG000001	FOXK2	0.83377608	0.93922729	0.01227622
ENSG000001	MON1A	0.89476261	0.96412642	0.01230581
ENSG000001	DNAJC17	0.87353226	0.95524941	0.01230839
ENSG000001	MARCHF8	0.91610672	0.9722662	0.01235143
ENSG000002	LILRA6	0.96577692	0.98812783	0.01238926
ENSG000001	EIF2AK4	0.81585781	0.93251163	0.01245224
ENSG00000(	RPLP0	0.9227358	0.97417119	0.01246878
ENSG000001	PPP6C	0.79496856	0.92462665	0.01252853
ENSG000001	OSBPL9	0.77210424	0.91511173	0.0125289
ENSG000001	KBTBD4	0.76658875	0.9132353	0.01252948
ENSG000001	RPL30	0.92874014	0.97652064	0.01257886
ENSG000002	C2orf92	0.93678907	0.97918399	0.01266558
ENSG000001	CDKN1A	0.90156245	0.96669547	0.0126882
ENSG000001	ANTKMT	0.89769816	0.96535697	0.01270038
ENSG000001	TTI1	0.72010948	0.89454101	0.01270641
ENSG000001	MGST3	0.89881435	0.96576173	0.01273319
ENSG000001	MTFR2	0.9136809	0.97135384	0.0127424
ENSG000001	SIAH2	0.86689428	0.95276749	0.01281996
ENSG000001	SF3A3	0.81884896	0.93394567	0.01283761
ENSG000001	CFAP298	0.88438277	0.96075855	0.01286648
ENSG00000(	CTNNA2	0.97816331	0.99280436	0.01290062
ENSG00000(	TMEM101	0.84486175	0.94421922	0.01292281
ENSG000001	RAB4B	0.83457442	0.93973543	0.01294404
ENSG000001	AFTPH	0.70603073	0.88780148	0.01297681
ENSG000001	FRS3	0.89549174	0.96443433	0.01297985
ENSG000001	SLC9C1	0.97431299	0.99128517	0.01298716

ENSG000001	CCDC154	0.87318728	0.95510037	0.01299503
ENSG000001	GNPDA1	0.7885259	0.92204735	0.01300872
ENSG000001	CENPX	0.88890775	0.96164016	0.01303069
ENSG000001	SAMM50	0.864956	0.95228442	0.01303739
ENSG00000C	SEC62	0.87996936	0.95827424	0.01303969
ENSG000002	NBPF9	0.89633438	0.96478497	0.01306506
ENSG000001	XG	0.97550084	0.99187219	0.0130767
ENSG000001	SEM1	0.9123175	0.97123107	0.01309993
ENSG000001	B3GNT5	0.92052584	0.97359159	0.01312835
ENSG000001	BNIP3	0.89893862	0.96576173	0.01313404
ENSG00000C	STX1B	0.92875853	0.97652064	0.01313684
ENSG000001	HECTD4	0.86962121	0.95412119	0.01318804
ENSG000001	GRAP	0.90955813	0.97001611	0.01320407
ENSG000001	KIF6	0.98185431	0.99341071	0.01325185
ENSG000001	EMILIN2	0.84428363	0.94399073	0.01325439
ENSG000002	MLLT11	0.86438794	0.95224252	0.01328391
ENSG000002	FAM229B	0.97811558	0.99280436	0.01329178
ENSG000001	NOB1	0.86613877	0.95248867	0.0133372
ENSG000001	PAFAH1B2	0.76464364	0.91243616	0.0133745
ENSG000001	C3orf49	0.97028106	0.9899265	0.0133867
ENSG000002	KLHL23	0.95410278	0.98485485	0.01339062
ENSG000001	STT3A	0.81701282	0.93283434	0.01346286
ENSG000001	C20orf96	0.91116868	0.97086554	0.01347224
ENSG000001	PHF14	0.86984351	0.95423131	0.01347914
ENSG000001	CAV1	0.96504405	0.98797129	0.01349884
ENSG000001	TUSC3	0.97784	0.99280436	0.01350067
ENSG000001	DDX23	0.63816349	0.8564206	0.01354938
ENSG000001	KSR1	0.80495828	0.92897486	0.01356251
ENSG000001	AHCY	0.84374145	0.94370944	0.01357375
ENSG000001	BTBD10	0.8524653	0.94733063	0.01359972
ENSG000001	RAB2B	0.83019674	0.93721091	0.01366991
ENSG000002	DACH1	0.90012063	0.96608561	0.01373538
ENSG000001	ERMAP	0.82645012	0.93615843	0.01373667
ENSG00000C	TRIT1	0.88695883	0.96095764	0.01377101
ENSG000001	LPXN	0.82194433	0.93539963	0.01383078
ENSG000001	ABCB8	0.84043139	0.94247424	0.01383821
ENSG000001	TRAIP	0.92096992	0.97363955	0.01387039
ENSG000001	RPS6	0.93780923	0.97941755	0.01394001
ENSG000001	NID1	0.8994156	0.96605219	0.01394128
ENSG000001	TAGAP	0.82709522	0.93644411	0.01395307
ENSG000001	ARF4	0.72361676	0.89594062	0.01397282
ENSG000001	RCCD1	0.90412559	0.9680374	0.01399454
ENSG000002	TRIM16	0.91855513	0.97272242	0.01401782

ENSG000001	MTLN	0.91646379	0.9722662	0.01403407
ENSG000001	SYMPK	0.78809668	0.92204735	0.01406659
ENSG00000(	MRI1	0.90311648	0.96763453	0.01407693
ENSG000001	DTNB	0.84516336	0.94421922	0.01408986
ENSG000001	ZNF836	0.88658768	0.96095764	0.01411916
ENSG000001	RCBTB2	0.81672947	0.93280078	0.01421164
ENSG000001	DHX38	0.62940637	0.85263401	0.01425764
ENSG000001	SARAF	0.83262088	0.93844684	0.01427616
ENSG000001	TMEM69	0.76746411	0.91352929	0.01428511
ENSG000001	SETD6	0.91281059	0.97123107	0.01431382
ENSG000001	FAM162A	0.89603664	0.96472031	0.01431807
ENSG000001	ADAM9	0.8655236	0.95237042	0.01433057
ENSG000001	CNNM4	0.81942667	0.93453894	0.01437882
ENSG000001	SEMA5A	0.96580663	0.98812783	0.01438551
ENSG000001	PRG4	0.87521172	0.95634141	0.01448228
ENSG000001	ASGR2	0.85918134	0.95037315	0.0144901
ENSG00000(	CYP51A1	0.94914317	0.98403516	0.01449247
ENSG000001	USP39	0.65176752	0.86353259	0.01449582
ENSG000001	TTYH3	0.83867185	0.94173685	0.01452453
ENSG000001	ZNF142	0.81564283	0.93238473	0.01456444
ENSG000001	ZBTB18	0.86157051	0.95081668	0.01456812
ENSG000001	DLG2	0.96700066	0.98843703	0.01458711
ENSG000001	NUDT16	0.78640878	0.92119044	0.01460241
ENSG000001	SHPK	0.91607095	0.9722662	0.01460538
ENSG000001	IQCK	0.95105903	0.98403516	0.01460715
ENSG000001	MAGEA8	0.96781939	0.98864761	0.01461188
ENSG000001	MAN1A1	0.87701045	0.9567976	0.01462055
ENSG000001	CD300LF	0.71489725	0.89175724	0.0146697
ENSG00000(	WDR37	0.76563946	0.9126184	0.01467425
ENSG000002	NBPF10	0.77952335	0.91827559	0.01471941
ENSG000001	HEATR4	0.91174385	0.9710451	0.01472859
ENSG000001	RBMS3	0.96019762	0.98631483	0.01474078
ENSG000001	MRFAP1	0.63902509	0.85691647	0.01479084
ENSG000001	UVSSA	0.80075115	0.92704431	0.01479905
ENSG000001	SNX32	0.89804559	0.96560269	0.01486093
ENSG000001	TLCD5	0.97652575	0.99241714	0.01489387
ENSG000001	NLRP11	0.96519692	0.98797129	0.01490646
ENSG000001	BDH2	0.93139888	0.97763745	0.01491517
ENSG000001	DNPH1	0.90100098	0.9663487	0.01493278
ENSG000002	NBPF15	0.90641282	0.96831534	0.01497452
ENSG000001	TNFRSF13C	0.87935976	0.95817324	0.01497796
ENSG000001	RALB	0.86129763	0.95080722	0.01505872
ENSG000001	GPR42	0.95989903	0.98631483	0.01509794

ENSG00000(	AACS	0.78959851	0.92261881	0.0151031
ENSG000001	RBX1	0.89021592	0.96183364	0.01515985
ENSG000001	CDCA2	0.9604208	0.98631483	0.01517108
ENSG000001	DAP3	0.84011912	0.94245133	0.01520739
ENSG00000(	ATRN	0.82923052	0.93682924	0.01520922
ENSG000001	CXXC5	0.84925976	0.94621791	0.01522312
ENSG000001	RABIF	0.76127982	0.91150138	0.01523468
ENSG000001	FARS2	0.87281269	0.95491272	0.01523661
ENSG000001	ZNF354A	0.89567766	0.96446171	0.01523911
ENSG000001	NMRAL1	0.97140499	0.99018788	0.01528505
ENSG000001	MAGED2	0.8175208	0.93321747	0.01532315
ENSG000001	MLEC	0.74759914	0.90727819	0.01536007
ENSG000001	NBR1	0.71094145	0.89054277	0.01539119
ENSG000001	REC114	0.96751395	0.98864217	0.01540976
ENSG000001	CRNKL1	0.64930814	0.86252724	0.01543189
ENSG000001	PEX19	0.69213769	0.88158966	0.01544419
ENSG000001	SRGAP3	0.90436175	0.9680581	0.01546407
ENSG000001	OGDH	0.77927845	0.91827559	0.01547165
ENSG000001	MED30	0.83688208	0.94117983	0.01547911
ENSG00000(	USP40	0.82199721	0.93539963	0.01549562
ENSG000001	LEFTY2	0.96134098	0.98649866	0.01550354
ENSG000001	CYP2E1	0.95649568	0.98578332	0.01552669
ENSG000001	TMEM102	0.83338629	0.93885334	0.01552747
ENSG000001	SLC25A27	0.95991114	0.98631483	0.01553607
ENSG000001	SP1	0.72895105	0.89755543	0.01556997
ENSG000001	RBM4	0.84683701	0.94515346	0.01559635
ENSG000001	ARNT	0.65345723	0.86395123	0.01559874
ENSG000001	ZCCHC18	0.91277701	0.97123107	0.0156006
ENSG00000(	LLGL2	0.88642127	0.96095764	0.01562046
ENSG000002	SLFN14	0.92297391	0.97421357	0.01564709
ENSG000001	GJB4	0.96815487	0.98879926	0.01567638
ENSG000001	ZAP70	0.88912836	0.96166446	0.01569264
ENSG000001	CDHR1	0.91581984	0.9722662	0.01571927
ENSG000001	CPT2	0.71070808	0.89043535	0.01575512
ENSG000001	SLC29A1	0.91445626	0.9716023	0.01583031
ENSG000001	PARD3	0.91839653	0.97272242	0.01583785
ENSG000001	PES1	0.76446455	0.91243616	0.01589466
ENSG000001	NIT2	0.89010565	0.96183364	0.01591354
ENSG000001	HOOK3	0.79448548	0.92447045	0.0159157
ENSG00000(	MYBPC2	0.94524932	0.9827131	0.01594969
ENSG00000(	PAFAH1B1	0.56710484	0.81973482	0.01596369
ENSG000001	TMEM179B	0.80780256	0.92977176	0.01602174
ENSG000001	FAM118A	0.83814329	0.94146888	0.01602577

ENSG000001	KDM8	0.85183222	0.9472127	0.016068
ENSG000001	HIBADH	0.87601913	0.95676199	0.01606853
ENSG000001	ECI2	0.87060637	0.95436374	0.01608326
ENSG000001	IFT27	0.85798986	0.94972056	0.01613962
ENSG000001	LGMN	0.79839757	0.92603117	0.01614843
ENSG00000(	COL17A1	0.95660019	0.98578332	0.01617909
ENSG000001	SLC5A11	0.97135621	0.99018788	0.01623464
ENSG000001	COX14	0.88995348	0.96183364	0.01624666
ENSG000001	DPY30	0.88887413	0.96164016	0.01636338
ENSG000001	CD2	0.89179117	0.96289488	0.01636365
ENSG000002	CDKN2AIPNL	0.86104877	0.95080722	0.01636637
ENSG000001	EP300	0.85011044	0.94644505	0.01641197
ENSG000001	SCAF4	0.66412758	0.86837979	0.01642113
ENSG00000(	IGBP1	0.86603439	0.95247761	0.01644555
ENSG000001	CAMSAP1	0.80168899	0.92746876	0.01650869
ENSG000001	PCDH12	0.88828688	0.96164016	0.01651973
ENSG000001	CAMLG	0.8612411	0.95080722	0.01652786
ENSG000001	YWHAH	0.71346012	0.89149739	0.01654294
ENSG000001	SLC35F3	0.92253079	0.9741258	0.01657357
ENSG000001	FKBP1B	0.90614382	0.96827466	0.01657991
ENSG000001	GLB1L3	0.90933864	0.96990945	0.01659026
ENSG000001	RNFT2	0.89269321	0.96329233	0.01664322
ENSG000001	SPATA17	0.96012647	0.98631483	0.0166601
ENSG00000(	NUDCD3	0.75369359	0.90944172	0.0166608
ENSG000001	NELFCD	0.73137784	0.8990265	0.0166794
ENSG000001	MRPS9	0.85839775	0.9497652	0.01672358
ENSG000001	MRPS11	0.83423255	0.93948078	0.01672867
ENSG000002	TBC1D3G	0.97597351	0.99229066	0.01674195
ENSG000002	PRRC2A	0.9532592	0.9845509	0.01674468
ENSG00000(	IP6K2	0.62392458	0.84972866	0.01674855
ENSG000001	OR52N4	0.95173297	0.98403516	0.01677629
ENSG000001	BACH2	0.88010648	0.95827424	0.01679945
ENSG000001	SCFD2	0.80478419	0.9289316	0.0168624
ENSG000001	MED8	0.62968237	0.85287112	0.01686679
ENSG000001	CDYL	0.75994024	0.91136366	0.01687289
ENSG000001	FARSA	0.81168939	0.93160392	0.01697489
ENSG000001	SLC25A19	0.82169981	0.93539963	0.01703341
ENSG000001	CDKN1B	0.74849511	0.90731063	0.01704905
ENSG000001	ATP6V1C1	0.72875955	0.89755543	0.01708216
ENSG000001	XPNPEP3	0.81049024	0.93101733	0.0172143
ENSG000001	BSPRY	0.91612391	0.9722662	0.01722185
ENSG000001	POC1B	0.81606215	0.93251163	0.01725365
ENSG000001	CCDC122	0.91754389	0.97272242	0.01725819

ENSG000001	PDE4DIP	0.84823198	0.9456054	0.01725852
ENSG000001	ZNF570	0.89495064	0.96412642	0.01727131
ENSG000001	ZC4H2	0.83594008	0.94062086	0.01732242
ENSG000001	NPHP3	0.86191751	0.95106725	0.01732425
ENSG000001	NKIRAS2	0.63492308	0.85504577	0.01732738
ENSG00000C	TESK2	0.75824969	0.9110527	0.01736103
ENSG00000C	PHPT1	0.82092069	0.93538871	0.01736244
ENSG000001	SIK3	0.74525782	0.90628646	0.01739656
ENSG000001	PPIB	0.78154988	0.91907937	0.01744151
ENSG00000C	RPS6KA2	0.84867736	0.94591943	0.01744588
ENSG000001	RGS3	0.76827125	0.91358163	0.01748603
ENSG000002	PGAM5	0.80345188	0.9284498	0.01751512
ENSG000001	MOCS3	0.8045262	0.92881463	0.01754666
ENSG000001	CLPB	0.696672	0.88285035	0.01760523
ENSG000001	SSBP2	0.82835501	0.93662954	0.01761761
ENSG00000C	LY75	0.81537949	0.93228896	0.01766671
ENSG000001	RAB22A	0.77337805	0.91571183	0.01767015
ENSG000002	N/A	0.96710416	0.98843703	0.0176881
ENSG000001	ASB9	0.96209259	0.98675362	0.01771459
ENSG000001	CBY1	0.89668768	0.96490939	0.01773602
ENSG000001	PHTF1	0.81419038	0.93216232	0.01774797
ENSG00000C	HUWE1	0.76852938	0.91358163	0.01779563
ENSG000001	FLAD1	0.76829788	0.91358163	0.01782596
ENSG000001	MBD1	0.5987592	0.83673325	0.0178416
ENSG00000C	COQ9	0.79496437	0.92462665	0.01787869
ENSG000001	LIN54	0.82231568	0.93539963	0.01788457
ENSG000001	PHYKPL	0.69339459	0.88183499	0.0179074
ENSG000001	ATF6	0.70120785	0.88513659	0.0179188
ENSG000001	SKP1	0.79259472	0.92319659	0.01794697
ENSG000001	SOX10	0.93074423	0.97733293	0.01795007
ENSG00000C	SMG6	0.78514314	0.92052634	0.0179675
ENSG000001	BICRAL	0.73849042	0.90333303	0.018039
ENSG00000C	DERL2	0.73459456	0.9010812	0.01804647
ENSG000001	HM13	0.70135066	0.88517922	0.018061
ENSG000001	PRPF4	0.67125325	0.87234986	0.01812907
ENSG000001	METTL16	0.80773348	0.92977176	0.01813597
ENSG000001	ZNF250	0.83822	0.94148991	0.01814057
ENSG000001	CCDC137	0.76811294	0.91357827	0.01817524
ENSG000001	MORF4L1	0.65811469	0.8654563	0.01825884
ENSG000001	ZNF211	0.80826226	0.92983947	0.01827922
ENSG000001	GNG11	0.89376034	0.9639421	0.01827945
ENSG000001	ETFBKMT	0.82807261	0.93660278	0.01828735
ENSG000001	SEPHS2	0.65188185	0.86355598	0.01835913

ENSG000001	PCNA	0.8565169	0.94910701	0.01836405
ENSG000001	TNNC1	0.93169686	0.97763745	0.01836533
ENSG000001	STAU1	0.65620847	0.86486055	0.0183792
ENSG000001	GATA3	0.85136362	0.94708079	0.01839183
ENSG000002	PDF	0.91368127	0.97135384	0.01841406
ENSG000001	RPP25	0.82296955	0.93539963	0.01842042
ENSG000001	SETD2	0.67359243	0.87364168	0.01842426
ENSG000001	MLYCD	0.7856309	0.92083223	0.01849081
ENSG000001	ZNF77	0.86062041	0.95080722	0.01849845
ENSG000001	NBAS	0.75832414	0.9110527	0.01850366
ENSG00000C	USP36	0.7959407	0.9246397	0.01850607
ENSG000001	PACSIN1	0.82215485	0.93539963	0.01854267
ENSG000001	ZNF428	0.80228061	0.92757225	0.01855512
ENSG000002	FAM200B	0.85571404	0.9486844	0.01856458
ENSG000001	NDUFB9	0.82329224	0.93539963	0.01856654
ENSG000001	BOLA1	0.85072435	0.94684256	0.01856998
ENSG000001	SLC50A1	0.75887638	0.91112428	0.01859296
ENSG00000C	C1QTNF3	0.90661746	0.96840672	0.01860693
ENSG000001	RASGRF2	0.88625593	0.96095764	0.01861925
ENSG000001	PARP2	0.87217643	0.95459934	0.01865431
ENSG000001	DRG2	0.79383809	0.92398201	0.01875449
ENSG00000C	POLD1	0.82588332	0.93591669	0.01877581
ENSG000001	TFAP2E	0.87848917	0.95778789	0.0187845
ENSG000001	METTL2B	0.80726394	0.92967903	0.01879098
ENSG000001	COPS7B	0.79235524	0.92314956	0.0188156
ENSG000001	PRMT7	0.80001397	0.92678559	0.01882285
ENSG000001	LTA4H	0.73559985	0.90150345	0.01882936
ENSG000001	POLR1C	0.84222985	0.9429376	0.01883896
ENSG000001	ENTPD1	0.69437857	0.8821445	0.01888507
ENSG000002	GOLGA8H	0.95930311	0.98631483	0.01891189
ENSG000001	SNRPF	0.86582335	0.95239982	0.01895185
ENSG000001	DET1	0.88566402	0.96095764	0.01908357
ENSG000001	TLR5	0.79899767	0.92603117	0.01908828
ENSG00000C	ARID4A	0.71329515	0.89145423	0.01909039
ENSG000001	CACNA1F	0.86736557	0.9528412	0.01914524
ENSG000001	WDR33	0.59065481	0.83266712	0.01915723
ENSG00000C	WDR47	0.79461116	0.92455044	0.01918248
ENSG000001	BACE1	0.83738263	0.94120179	0.01920495
ENSG000002	CSKMT	0.89230735	0.96313198	0.0192439
ENSG000001	MIF4GD	0.74140183	0.90539572	0.01925143
ENSG000001	UBE2L3	0.63953168	0.85691647	0.01925271
ENSG000001	JAZF1	0.75217344	0.908573	0.01925308
ENSG000001	COP1	0.68793634	0.88051877	0.01925776

ENSG000001	CYP4F11	0.96228274	0.98677433	0.01926114
ENSG000001	TMSB4Y	0.82690172	0.93642093	0.01933131
ENSG000002	BRK1	0.80620275	0.92964486	0.01937442
ENSG000001	PPIL2	0.69032967	0.88074719	0.01938968
ENSG000001	SYNDIG1L	0.96764391	0.98864761	0.0194157
ENSG000001	UGT2B28	0.95997147	0.98631483	0.01942527
ENSG000001	ILF3	0.77377943	0.91586084	0.01942634
ENSG000001	KCNC4	0.89198224	0.96298656	0.01942923
ENSG000001	ID3	0.8700162	0.95423131	0.0194352
ENSG000001	BUD31	0.65733245	0.86522797	0.01945333
ENSG00000C	NUP37	0.84715296	0.94527257	0.01950587
ENSG000001	GPN1	0.82588869	0.93591669	0.01951517
ENSG000001	TMEM14C	0.87145483	0.95449478	0.01951976
ENSG000001	NUDT13	0.80636335	0.92964486	0.01953412
ENSG000001	C14orf119	0.72105559	0.89490971	0.01954466
ENSG000001	DGCR6L	0.80433016	0.92881463	0.01959744
ENSG000001	DICER1	0.82956643	0.93686532	0.01961215
ENSG000001	CCDC65	0.8074818	0.92973209	0.01962273
ENSG000001	RBMX2	0.80686714	0.92967903	0.01972349
ENSG000001	RNF185	0.67395252	0.87391249	0.01974048
ENSG000001	YTHDF1	0.57545675	0.82346952	0.01976598
ENSG000001	OR2B11	0.89891276	0.96576173	0.01977871
ENSG000001	ARHGEF9	0.84881086	0.94594163	0.01981075
ENSG000001	C1orf50	0.82262253	0.93539963	0.01982352
ENSG000001	SMYD5	0.79744319	0.9258334	0.01985624
ENSG000001	TTYH2	0.71382955	0.89153258	0.01986079
ENSG000001	SNTB2	0.79488974	0.92462665	0.01987477
ENSG000001	TM9SF4	0.5215253	0.79758937	0.01993987
ENSG000001	KDM2A	0.61921313	0.84711589	0.0199442
ENSG000001	TNFRSF18	0.87132087	0.95449478	0.0200255
ENSG000001	UQCR10	0.80318405	0.92827243	0.02004674
ENSG000001	KAT2A	0.82429605	0.93591669	0.02006907
ENSG000001	ZNF366	0.90404434	0.96802495	0.02009214
ENSG000001	KAT7	0.56977636	0.8205285	0.02010501
ENSG000002	MRPL20	0.75974888	0.91136366	0.02014811
ENSG000001	NXT1	0.83162557	0.93804129	0.02020145
ENSG00000C	HIPK2	0.76758515	0.91352929	0.02020325
ENSG000001	SENP2	0.55831617	0.81600055	0.02024432
ENSG000001	APEX2	0.70410586	0.88665789	0.02025553
ENSG00000C	FCN1	0.74715309	0.90702989	0.02025661
ENSG000001	MFSD13A	0.69785501	0.88337661	0.02031868
ENSG000001	MTHFSD	0.69998424	0.88452288	0.02035639
ENSG000001	ZDHHC14	0.8284419	0.93664814	0.02036626

ENSG000001	ZNF546	0.89279688	0.96334017	0.02037184
ENSG000001	TUBG1	0.84596391	0.94474488	0.0203974
ENSG000001	ECH1	0.7889138	0.92208417	0.02043098
ENSG000001	PHF20L1	0.78507709	0.92051536	0.02044371
ENSG000001	TMEM50A	0.53245373	0.80280163	0.02044951
ENSG000001	UPP1	0.69451286	0.8821445	0.02046293
ENSG000001	DDX25	0.96308779	0.98706408	0.02046317
ENSG000001	RALGPS1	0.83209842	0.93831391	0.02047259
ENSG000001	RASD1	0.91694772	0.97240416	0.02048851
ENSG000001	PRELID1	0.76587386	0.91276379	0.0205519
ENSG000001	NAA16	0.86505045	0.95229913	0.02057264
ENSG000001	PRKAG1	0.6507456	0.8633186	0.02064655
ENSG000001	TXNL4A	0.72669876	0.89706038	0.02068383
ENSG000001	PCSK7	0.82203105	0.93539963	0.02068396
ENSG000001	MOSPD1	0.80453871	0.92881463	0.02073585
ENSG000001	EXO1	0.92212805	0.97410521	0.02075215
ENSG000001	TNKS1BP1	0.81528803	0.93228896	0.02076894
ENSG000001	DCAKD	0.81256666	0.93170624	0.02082006
ENSG000001	ZMAT3	0.81301309	0.93170624	0.02084342
ENSG000001	TEX261	0.44400671	0.7491529	0.0208475
ENSG000001	PFDN5	0.87409786	0.95573919	0.0208704
ENSG000001	FRMPD3	0.90663254	0.96840672	0.02087162
ENSG000001	MMAB	0.8187522	0.93390091	0.02088797
ENSG000001	ISCA1	0.81602727	0.93251163	0.02089958
ENSG000002	NCBP2AS2	0.82377861	0.9356656	0.02099383
ENSG000002	JPT2	0.79047665	0.92280247	0.02102381
ENSG000001	CDK16	0.63815949	0.8564206	0.02102625
ENSG000001	DEPDC5	0.65006223	0.86293893	0.02104023
ENSG000001	ALDH2	0.71937718	0.89410037	0.0210569
ENSG000001	VAV2	0.69758438	0.883235	0.02106656
ENSG000001	CARS2	0.70444429	0.88694649	0.02109736
ENSG000001	PTBP3	0.73120435	0.89893376	0.02110364
ENSG00000C	P2RX5	0.78586221	0.92097039	0.02111693
ENSG000001	ZNF266	0.82051218	0.93512012	0.02116287
ENSG000001	KLC2	0.84180158	0.94283868	0.02118176
ENSG00000C	LIG3	0.6392263	0.85691647	0.02118368
ENSG000001	TMEM221	0.89742308	0.96522986	0.02119315
ENSG00000C	ZNF416	0.83246604	0.93841777	0.0212115
ENSG000001	PHF8	0.54034907	0.80714764	0.02122644
ENSG000001	KLHL32	0.91491407	0.97189792	0.0212683
ENSG000001	GNRH1	0.82204294	0.93539963	0.02130361
ENSG000001	UCK2	0.78261781	0.91935794	0.02133453
ENSG000001	VWA8	0.81435387	0.93216232	0.02137687

ENSG000001	IER2	0.75195568	0.908573	0.02141354
ENSG000001	IKBKB	0.63543475	0.85504577	0.0214299
ENSG000002	NDUFAF8	0.86053727	0.95080722	0.0215143
ENSG000001	SLC26A1	0.87568493	0.95663704	0.02158182
ENSG000001	CFAP70	0.89502463	0.96414217	0.02158427
ENSG000002	MRPS21	0.84412886	0.94396799	0.02162377
ENSG000002	EEF1AKMT4	0.84767325	0.94527257	0.02162751
ENSG000001	SSBP4	0.80946621	0.93030173	0.02169517
ENSG000001	CPT1A	0.82482628	0.93591669	0.02170656
ENSG000001	GKN2	0.95772471	0.98585223	0.0217099
ENSG000001	USP5	0.70669896	0.88840748	0.02173298
ENSG000001	SNAP47	0.75996708	0.91136366	0.02175374
ENSG000001	DIPK1A	0.86471261	0.95224252	0.02175889
ENSG000001	PLCL2	0.4359919	0.74463032	0.02177335
ENSG000001	SPTY2D1	0.74347915	0.90602454	0.02186046
ENSG000001	RERE	0.81116503	0.931484	0.02190664
ENSG000001	PAICS	0.80273899	0.92789015	0.0219109
ENSG000001	CDC26	0.77043142	0.91430362	0.0219515
ENSG000001	SETD5	0.59286222	0.83431304	0.02198694
ENSG000001	CCNA1	0.94173488	0.9808807	0.02198762
ENSG000001	SMIM12	0.59709404	0.83621548	0.02200551
ENSG000001	ITPA	0.78652714	0.92119044	0.02201289
ENSG00000C	TUBA3D	0.87039903	0.95426978	0.02201425
ENSG000001	MED18	0.52238877	0.7979767	0.02204208
ENSG000001	ANAPC16	0.79505024	0.92462665	0.02205678
ENSG00000C	GNAI3	0.6843608	0.87902381	0.02206364
ENSG000001	MYCT1	0.88379844	0.96050431	0.02207255
ENSG000001	FCER1G	0.7611098	0.91141904	0.02211006
ENSG000001	CORO1B	0.69772634	0.88328259	0.02215214
ENSG000001	NAA40	0.81266722	0.93170624	0.02216545
ENSG000001	FAM32A	0.52453174	0.79886642	0.0221894
ENSG000001	IQSEC2	0.76198632	0.91151487	0.02219177
ENSG00000C	GOLGA3	0.70913907	0.88947787	0.02219601
ENSG000001	NIM1K	0.93834472	0.97954033	0.02220481
ENSG000001	HADHB	0.33536843	0.67708232	0.02220704
ENSG00000C	SLCO1A2	0.94839746	0.98373753	0.02224747
ENSG000001	WDR53	0.81671281	0.93280078	0.02225664
ENSG000001	CDC20B	0.95338826	0.9845509	0.02230589
ENSG000001	KLHL30	0.96188372	0.9866758	0.02232733
ENSG000001	ACO2	0.68280757	0.87781049	0.02233063
ENSG000001	CSPP1	0.80810339	0.92978846	0.02233909
ENSG00000C	SH3GLB1	0.63031793	0.85294376	0.0223997
ENSG000001	REXO4	0.76422601	0.91243616	0.02241468

ENSG000001	BECN1	0.24540968	0.61141177	0.02241597
ENSG000001	IVD	0.74926591	0.90770972	0.02244487
ENSG000001	CTU1	0.80819293	0.92982559	0.02244993
ENSG000001	CHCHD3	0.70775437	0.88890112	0.02245343
ENSG000001	MYO18A	0.68300716	0.87799757	0.02246349
ENSG000001	NIPBL	0.70907386	0.88947345	0.02246426
ENSG000001	GTF2IRD2	0.80719208	0.92967903	0.02250123
ENSG000001	FCER2	0.7680507	0.91357827	0.0225217
ENSG00000C	MTIF2	0.80026526	0.92689709	0.02258317
ENSG00000C	SRCAP	0.75009724	0.9080858	0.02259761
ENSG000001	SPAG16	0.90144095	0.96669287	0.02261666
ENSG000001	CCDC74A	0.96639704	0.98843394	0.02263139
ENSG000001	GOLGA6A	0.95068543	0.98403516	0.02265056
ENSG000001	FBXO38	0.62921734	0.85252019	0.02268252
ENSG000001	DLC1	0.88868683	0.96164016	0.02270938
ENSG00000C	BCAT1	0.853161	0.94771671	0.02273368
ENSG000001	BFAR	0.93607171	0.97900138	0.02276196
ENSG000002	ADORA3	0.88642923	0.96095764	0.02279747
ENSG000001	CREB3L4	0.81795796	0.9333228	0.02285401
ENSG000001	KLHL18	0.56173022	0.81801019	0.02289209
ENSG000001	SPICE1	0.915602	0.97224705	0.02291136
ENSG000001	CYRIB	0.6565417	0.86503541	0.0229262
ENSG000001	ZNF333	0.72106124	0.89490971	0.02295146
ENSG000001	MTIF3	0.80676846	0.92967903	0.02295944
ENSG000001	UACA	0.84577882	0.94462172	0.02296363
ENSG000001	DNAJB13	0.88602645	0.96095764	0.02298279
ENSG000001	TCAIM	0.80456844	0.92881463	0.02301068
ENSG00000C	HFE	0.66611613	0.86978107	0.02302491
ENSG000001	JMJD7-PLA2C	0.81302601	0.93170624	0.02305613
ENSG000001	RAMAC	0.69850214	0.88371351	0.02306991
ENSG000001	KDELR3	0.87872762	0.95796297	0.02307169
ENSG000001	SEC61B	0.71821569	0.89332947	0.02318922
ENSG000001	GOLGA1	0.61316168	0.8450816	0.02320663
ENSG000001	RABEP2	0.72138813	0.89490971	0.02321058
ENSG000001	VTI1B	0.62910444	0.85243835	0.02330872
ENSG000001	DENND10	0.65871172	0.86560481	0.02333108
ENSG000001	TCF7L1	0.77339256	0.91571183	0.02333628
ENSG00000C	CMTM6	0.74017366	0.90464694	0.0233427
ENSG000001	EIF3H	0.79511167	0.92462665	0.02336833
ENSG000001	ZFP37	0.91221974	0.97123107	0.02339009
ENSG000001	CARD11	0.76782305	0.91357827	0.02339542
ENSG000002	N/A	0.86847239	0.95343718	0.02339597
ENSG000001	OSBPL11	0.74319153	0.90601403	0.02340757

ENSG000001	PATZ1	0.68534087	0.87972633	0.02342753
ENSG000001	ARHGEF39	0.86662483	0.95264796	0.02349041
ENSG00000(	SEC61A1	0.56652123	0.8197023	0.0236126
ENSG000001	SPHK1	0.75651041	0.91082077	0.02368833
ENSG000001	HRH1	0.95715282	0.98578332	0.02369612
ENSG00000(	PTGS2	0.85232601	0.94731122	0.02370068
ENSG000002	UBAP1L	0.80168624	0.92746876	0.02370713
ENSG000001	HIC1	0.87834415	0.95778789	0.02371079
ENSG000001	SMPD4	0.67938972	0.87598276	0.02372235
ENSG000001	NSMF	0.78098819	0.9189743	0.02372251
ENSG000002	OR2L2	0.95862862	0.98620369	0.02374034
ENSG000001	PAXX	0.77152609	0.9150003	0.02378216
ENSG000001	TRAK1	0.65474926	0.86439228	0.02382542
ENSG00000(	SPATA7	0.85369605	0.94798679	0.0238435
ENSG000001	ATPSCKMT	0.84080698	0.94263513	0.02385484
ENSG000001	KRAS	0.72576901	0.89686038	0.02386602
ENSG00000(	CLPTM1L	0.78869388	0.92204735	0.02389815
ENSG000001	GALNT10	0.52813283	0.80062313	0.02396112
ENSG000001	INTS8	0.75225865	0.908573	0.02405209
ENSG000001	RAD51D	0.78491281	0.92041622	0.02405732
ENSG000001	ATP5F1B	0.59021689	0.83225833	0.02406249
ENSG000001	SEC23B	0.43641667	0.74496191	0.02408413
ENSG000002	IKBKE	0.67833323	0.87557173	0.02415035
ENSG00000(	ARSD	0.61122296	0.84339687	0.02415878
ENSG000001	PFKFB2	0.82062876	0.93518734	0.02417097
ENSG000001	EIF2D	0.80767519	0.92977176	0.02417912
ENSG00000(	NUP188	0.68888062	0.88059791	0.02427931
ENSG000001	PRPS1	0.77160949	0.91503238	0.02434747
ENSG000001	NSA2	0.8538973	0.94801844	0.02437106
ENSG000001	RAPH1	0.83848094	0.9416561	0.0243945
ENSG000001	NPR2	0.89902621	0.96576173	0.02440631
ENSG000001	SHMT2	0.72236596	0.89532737	0.02451224
ENSG000001	JAKMIP2	0.86213392	0.9512414	0.02454251
ENSG00000(	KDM5A	0.49883276	0.78354914	0.02455246
ENSG000001	PTRH2	0.80671829	0.92967903	0.02455474
ENSG00000(	NSMAF	0.65427285	0.86439091	0.0245743
ENSG00000(	CRAMP1	0.67771646	0.8751149	0.02457479
ENSG000001	AMD1	0.6964036	0.88266566	0.02457872
ENSG000001	RBSN	0.63332541	0.85444991	0.0245825
ENSG000001	OSBP	0.48162875	0.77466133	0.02464584
ENSG000001	OGG1	0.67745379	0.87510133	0.02465322
ENSG00000(	EXOSC7	0.81784809	0.9333228	0.02470258
ENSG000001	COMMD4	0.73628918	0.90186782	0.02471855

ENSG000001	HNRNPH2	0.63930903	0.85691647	0.02473078
ENSG000001	PLA2G15	0.73614315	0.90182256	0.02473341
ENSG000001	FBXL5	0.66371886	0.86832674	0.02479094
ENSG000001	GLE1	0.19590271	0.5634849	0.02480564
ENSG000001	ABL2	0.69598883	0.88245905	0.02481347
ENSG000001	BMP4	0.95775013	0.98585223	0.02483435
ENSG000002	ZNF8	0.75123482	0.908573	0.02483569
ENSG000001	STOX2	0.94835667	0.98373753	0.0248555
ENSG000000	PRKCQ	0.78390214	0.92000137	0.02487748
ENSG000001	WDR45B	0.56608083	0.8197023	0.0249021
ENSG000001	HSD17B1	0.8084879	0.92996727	0.02492708
ENSG000001	TUBA1B	0.66510813	0.86898991	0.02495954
ENSG000001	NPDC1	0.82513047	0.93591669	0.02496451
ENSG000001	ZBTB3	0.68857411	0.88057199	0.02506206
ENSG000001	PPM1D	0.58642057	0.82959975	0.02507677
ENSG000001	NR3C1	0.72588007	0.89689633	0.02508285
ENSG000001	GRIA4	0.95817769	0.98595586	0.0251099
ENSG000001	COPZ1	0.68587125	0.8799229	0.02511839
ENSG000001	PTPN12	0.75060581	0.90815046	0.02512716
ENSG000001	TMEM223	0.76520383	0.91260342	0.02514036
ENSG000001	ARHGAP21	0.70092132	0.88498263	0.02516035
ENSG000001	FBXL12	0.65194289	0.86356638	0.02519411
ENSG000001	MMADHC	0.64524675	0.8599145	0.02522392
ENSG000001	ALYREF	0.69251138	0.88158966	0.02522667
ENSG000001	PRKAG3	0.94936325	0.98403516	0.02522948
ENSG000001	CST6	0.95734725	0.98578332	0.0252608
ENSG000001	FAM204A	0.79999902	0.92678559	0.02527691
ENSG000001	PFDN2	0.74763869	0.90727819	0.02529207
ENSG000001	MRPL49	0.5579247	0.81587881	0.02530424
ENSG000001	SLC16A6	0.75668929	0.91082077	0.02530983
ENSG000001	MAPK7	0.6854356	0.87972633	0.02531316
ENSG000001	SUPT20H	0.64507875	0.85991395	0.02532245
ENSG000001	RBP5	0.82580056	0.93591669	0.02537331
ENSG000000	NCBP3	0.64769472	0.86142128	0.02540924
ENSG000001	SPRY4	0.94055831	0.98047286	0.02542327
ENSG000002	TMEM233	0.94598833	0.98315624	0.02543898
ENSG000002	GATC	0.70908076	0.88947345	0.02543907
ENSG000002	ZNF611	0.6605205	0.86700658	0.02548699
ENSG000000	CTNNA1	0.68749089	0.88048506	0.02550228
ENSG000000	ZNF671	0.72306977	0.89580985	0.02561269
ENSG000001	HMGCL	0.59749075	0.83640463	0.02563609
ENSG000001	TMEM131L	0.43016683	0.74091639	0.0256558
ENSG000001	CCDC97	0.67298406	0.87354948	0.02566175

ENSG000001	YBEY	0.78196713	0.91932519	0.02566947
ENSG000001	AKIRIN1	0.60924918	0.84246798	0.02567811
ENSG00000(	DNAJA2	0.65533956	0.8644689	0.02568533
ENSG000001	CDK20	0.80838096	0.92991014	0.02570593
ENSG000001	NBPF3	0.82418641	0.93589165	0.02571852
ENSG000001	RPTOR	0.71992399	0.8945116	0.0257426
ENSG00000(	PLPP1	0.82802122	0.93660278	0.02583502
ENSG000001	OXNAD1	0.8713326	0.95449478	0.02587139
ENSG00000(	ELP1	0.73634672	0.90186782	0.02590389
ENSG000001	SMARCAL1	0.63327296	0.85444991	0.02590484
ENSG000001	SELL	0.80868333	0.93001	0.02591285
ENSG000001	RAB2A	0.60401594	0.83883394	0.02591766
ENSG000001	C11orf96	0.95347426	0.9845509	0.02592413
ENSG000001	STIP1	0.67413364	0.87399456	0.02593063
ENSG000001	PUDP	0.62704433	0.85099522	0.02593119
ENSG000001	FAM189A1	0.92853016	0.97652064	0.02596387
ENSG000002	N/A	0.95140354	0.98403516	0.02597984
ENSG000001	SMYD4	0.81163724	0.93160392	0.02599671
ENSG000001	SLC27A4	0.73808036	0.90295968	0.02599917
ENSG000001	LHX4	0.86739827	0.9528412	0.02600176
ENSG000001	CSTA	0.86573797	0.95237042	0.02601143
ENSG000001	FKBP15	0.48067565	0.77396792	0.02608388
ENSG000001	RAB11A	0.50426969	0.78698836	0.02608933
ENSG00000(	ITCH	0.69406124	0.8821445	0.02609153
ENSG000001	ZNF287	0.85559981	0.9486844	0.0261143
ENSG000002	TMA7	0.84961794	0.94637076	0.02614113
ENSG000001	EXOSC1	0.769513	0.91386073	0.02614461
ENSG000001	CCDC86	0.71396902	0.89153258	0.02616039
ENSG000001	BBS9	0.72359768	0.89594062	0.02619283
ENSG000001	ITIH2	0.83117119	0.9379197	0.02620655
ENSG00000(	ZNF343	0.75238987	0.908573	0.02624851
ENSG000001	CARD6	0.62161622	0.84866582	0.02625348
ENSG000001	PDE6D	0.61804893	0.84664309	0.02626998
ENSG000001	CDS1	0.90045187	0.96608561	0.02630726
ENSG000001	MRPS15	0.76957222	0.91386073	0.02634968
ENSG00000(	ANAPC5	0.69903802	0.88418481	0.02637832
ENSG000001	MTFR1L	0.66380573	0.86837042	0.02638071
ENSG000001	C1QL3	0.92250272	0.9741258	0.02641368
ENSG000001	GPATCH4	0.72680214	0.89706038	0.02649665
ENSG00000(	TRAM2	0.72411766	0.89615079	0.02650225
ENSG000001	TAS2R60	0.95029422	0.98403516	0.02652627
ENSG000001	SSRP1	0.67503449	0.87446425	0.02655123
ENSG000001	MORC4	0.82730283	0.93654857	0.02658344

ENSG000001	TACO1	0.65585213	0.86467033	0.02659354
ENSG000001	FAM153B	0.92803868	0.97652064	0.02661068
ENSG000001	ARMC6	0.58974935	0.83191276	0.02662433
ENSG000001	ETF1	0.48981807	0.77995113	0.02663266
ENSG000002	ANKRD39	0.81544422	0.93228896	0.02665249
ENSG000001	CHCHD2	0.69826341	0.8835822	0.02665717
ENSG000001	POU2AF1	0.8018975	0.92757225	0.02668779
ENSG00000C	LRRFIP2	0.33279898	0.67523724	0.02672946
ENSG000001	CDCA7	0.86958039	0.95412119	0.02675442
ENSG000001	AMZ2	0.68032456	0.87644445	0.02675627
ENSG000001	ZNF587	0.69364573	0.8818985	0.02676715
ENSG00000C	POMT2	0.69450212	0.8821445	0.02681006
ENSG000001	USHBP1	0.87216726	0.95459934	0.02682457
ENSG000001	LRRC8B	0.78119488	0.9189743	0.02682911
ENSG000001	C1orf127	0.89005409	0.96183364	0.02684584
ENSG000001	SETD3	0.66684833	0.87009558	0.02688058
ENSG000001	PAXIP1	0.71392282	0.89153258	0.02692792
ENSG000001	SND1	0.5882052	0.83092812	0.02697662
ENSG000002	NDUFS3	0.79693688	0.92559829	0.02702418
ENSG000001	PREB	0.62006116	0.84761181	0.02703037
ENSG000001	TRIM73	0.86015379	0.95064852	0.02705719
ENSG000001	ZNF439	0.85740001	0.94961808	0.02706605
ENSG000001	WDR5	0.66843867	0.8710625	0.02715607
ENSG000001	CCNG1	0.80935896	0.93030173	0.02718039
ENSG000002	OTUD7B	0.73010461	0.89828814	0.02721696
ENSG000001	AAR2	0.61381645	0.8451435	0.02723962
ENSG000001	UBIAD1	0.74856441	0.90731063	0.02724402
ENSG000001	CD1D	0.7416899	0.90543757	0.02724764
ENSG000001	REPS1	0.637289	0.85602759	0.02727193
ENSG000001	OR10H1	0.94980693	0.98403516	0.02727591
ENSG000001	TYSND1	0.75323158	0.90916139	0.02728287
ENSG000001	ZNF286A	0.83667559	0.94106443	0.02730195
ENSG000001	URM1	0.67200921	0.87312263	0.02740816
ENSG000001	RXFP4	0.93712082	0.97920445	0.02745196
ENSG000001	ZNF696	0.78329704	0.91983554	0.02749956
ENSG00000C	PLEKHG2	0.753832	0.90944172	0.02750955
ENSG000001	HNRNPAB	0.5997478	0.83747033	0.02751047
ENSG000001	APH1A	0.39762851	0.72381553	0.02751173
ENSG000001	RAD21	0.63376397	0.85461553	0.02755785
ENSG000002	TRAF3IP1	0.73921449	0.90388024	0.02759187
ENSG000001	C15orf40	0.78011732	0.91848053	0.02760321
ENSG000001	SHISA3	0.95552282	0.98528818	0.02772254
ENSG000001	SERAC1	0.70483852	0.88716768	0.02772542

ENSG000001	AAK1	0.72733285	0.89708846	0.02773521
ENSG000001	MFSD9	0.59162464	0.83336595	0.02776118
ENSG000001	TRRAP	0.71168103	0.89073961	0.02780505
ENSG000001	C1orf159	0.73178453	0.89916258	0.0279013
ENSG000001	SNRK	0.75252324	0.90865769	0.02791715
ENSG000001	PPID	0.58850589	0.83116738	0.02791838
ENSG000001	TNFRSF10B	0.64765708	0.86142128	0.02793844
ENSG00000C	COX15	0.38060956	0.70987972	0.0279721
ENSG000001	PIGG	0.6856684	0.87981649	0.02798444
ENSG000002	TIMM23	0.70391045	0.88657108	0.02800263
ENSG000001	MBOAT1	0.62729645	0.85112497	0.02801295
ENSG000001	C2CD3	0.58857933	0.83116738	0.02802119
ENSG000001	ATP5PB	0.74962539	0.90788681	0.02811417
ENSG000001	MSL2	0.6528156	0.86379938	0.02813733
ENSG00000C	HSD17B10	0.7209883	0.89490971	0.02816691
ENSG000001	GAR1	0.73661183	0.90203016	0.02818449
ENSG000001	NAALADL1	0.77969712	0.91827559	0.02822638
ENSG00000C	BCKDHB	0.79727365	0.92579076	0.02823053
ENSG000001	JAKMIP3	0.84093667	0.942702	0.02823889
ENSG00000C	CS	0.53323531	0.80301815	0.02824309
ENSG000001	NDUFA3	0.90328749	0.96765052	0.02831918
ENSG000001	SUPV3L1	0.70212687	0.88553899	0.02832284
ENSG000001	EXOC6B	0.71508679	0.89175724	0.0283367
ENSG000001	DOK2	0.72766592	0.8971681	0.0283414
ENSG000001	PSMB4	0.53855189	0.80633537	0.02835032
ENSG000001	HADH	0.79523853	0.92462665	0.02835334
ENSG000001	ZNF740	0.56545486	0.81924606	0.0283751
ENSG000001	CCDC62	0.86157331	0.95081668	0.02837624
ENSG000001	RLF	0.7289563	0.89755543	0.02838044
ENSG000001	MCMBP	0.45337876	0.75477115	0.02840545
ENSG000001	GPER1	0.85352344	0.94785994	0.02848451
ENSG000002	STON1	0.69565929	0.88231682	0.02853313
ENSG000001	TMEM140	0.83078813	0.93764588	0.02854295
ENSG000001	LBHD1	0.80875894	0.93001	0.02854676
ENSG000001	SLC25A35	0.75712697	0.91082077	0.02859411
ENSG000001	RPP14	0.70989518	0.88993477	0.02859776
ENSG000001	NXF1	0.47414445	0.76912287	0.0286069
ENSG000001	CLCN7	0.61124874	0.84339687	0.02862298
ENSG000001	MPC2	0.58754611	0.83046863	0.0286295
ENSG000001	CDK5RAP2	0.61947604	0.84711589	0.02863239
ENSG000001	ZBED6CL	0.51034296	0.79143652	0.02864267
ENSG00000C	SPAG5	0.78833673	0.92204735	0.02866485
ENSG000001	WIPF1	0.58702102	0.83026658	0.02867367

ENSG000000	KDM1A	0.60924971	0.84246798	0.0286964
ENSG000001	SFXN1	0.76115306	0.91141904	0.02870711
ENSG000001	NMT2	0.78543568	0.92073635	0.02872092
ENSG000000	ELAC2	0.62649916	0.85068296	0.02876897
ENSG000001	R3HCC1L	0.49007365	0.77995936	0.02877702
ENSG000001	DDX54	0.61364093	0.8451435	0.02880182
ENSG000001	TLE4	0.63877357	0.85674595	0.02884508
ENSG000000	HSPB6	0.94371131	0.98221647	0.02887279
ENSG000001	ATPAF1	0.69642358	0.88266566	0.02887664
ENSG000001	ATP6V1F	0.68415184	0.87891236	0.02890448
ENSG000001	RPP38	0.69596778	0.88245905	0.02899479
ENSG000001	ZNF584	0.80130376	0.92735626	0.02900403
ENSG000001	ENTPD6	0.64772386	0.86142128	0.02900531
ENSG000000	ELAVL1	0.53871585	0.8064433	0.02902025
ENSG000001	TATDN2	0.62285733	0.84927918	0.02903455
ENSG000000	SLC46A1	0.80882187	0.93001	0.02911058
ENSG000001	CHCHD4	0.70803331	0.88904065	0.02912588
ENSG000001	ZNF7	0.72679529	0.89706038	0.02913475
ENSG000001	YWHAZ	0.33003797	0.67364255	0.02917101
ENSG000000	ABCF2	0.68445922	0.87902381	0.02917971
ENSG000001	TMEM50B	0.95088364	0.98403516	0.02918783
ENSG000000	SMARCA2	0.605596	0.83945694	0.02920558
ENSG000001	PPP1R14B	0.76259249	0.91167139	0.02922483
ENSG000001	ZNF317	0.40545239	0.72703815	0.0292682
ENSG000001	RBFA	0.7757351	0.91684457	0.02927616
ENSG000001	MMGT1	0.59854683	0.83673325	0.02931249
ENSG000001	MARCHF5	0.38106822	0.71037942	0.0293149
ENSG000002	GALT	0.63850179	0.85645217	0.02932535
ENSG000001	SERPINB8	0.62497618	0.85016376	0.02932551
ENSG000000	PDRG1	0.64464958	0.85986817	0.02938122
ENSG000001	COQ4	0.72941504	0.89805217	0.02938695
ENSG000001	HMGXB4	0.59694805	0.83614954	0.02944606
ENSG000001	MFSD1	0.58761434	0.83046863	0.02946294
ENSG000001	MAGOH	0.68034156	0.87644445	0.02946579
ENSG000001	CD247	0.77669946	0.9172058	0.02950912
ENSG000001	SBNO1	0.60486465	0.83913084	0.02951153
ENSG000001	SIAH1	0.68676771	0.88023892	0.0295199
ENSG000001	ACSBG2	0.93411125	0.97846361	0.02952096
ENSG000000	FNDC3B	0.72882535	0.89755543	0.02955483
ENSG000001	IPO4	0.896894	0.96506745	0.02956559
ENSG000001	PIAS3	0.49782261	0.78278241	0.02958178
ENSG000001	PYGB	0.61121863	0.84339687	0.02959499
ENSG000001	FAAH2	0.82642968	0.93615843	0.02960503

ENSG000001	SRPK3	0.76973062	0.91393902	0.02961365
ENSG00000(	BAZ1B	0.5714803	0.82164253	0.02962361
ENSG000001	CLEC11A	0.79372464	0.92392642	0.02962408
ENSG000001	CNOT10	0.49464051	0.78124152	0.02966233
ENSG000001	COA1	0.72658679	0.89706038	0.02969092
ENSG000001	CXCR5	0.76399797	0.91240328	0.02969967
ENSG000001	CASP6	0.78154503	0.91907937	0.02971253
ENSG00000(	WNT11	0.87734395	0.95703283	0.02971843
ENSG000001	KIF3B	0.48306334	0.77585394	0.02974264
ENSG000001	METTL17	0.71946609	0.89414244	0.02985902
ENSG000001	SATB1	0.77175253	0.91507664	0.02987163
ENSG000001	TMEM41B	0.7606761	0.91141904	0.02993376
ENSG00000(	ABCC2	0.7381277	0.90295968	0.02994631
ENSG000001	TIMM9	0.74224062	0.90555138	0.03003554
ENSG00000(	OGFR	0.72991894	0.89828814	0.0300486
ENSG000001	SLC4A2	0.61371842	0.8451435	0.03006225
ENSG00000(	FUS	0.48609419	0.77752459	0.0300648
ENSG000001	NOP56	0.66545291	0.86926459	0.03016413
ENSG000002	DNAJC9	0.76517147	0.91260342	0.0301829
ENSG000001	ATP5MC1	0.69414621	0.8821445	0.0301982
ENSG000001	ERLIN1	0.6533405	0.86393759	0.03020418
ENSG00000(	RDH11	0.65294012	0.86379938	0.03020558
ENSG00000(	RAD52	0.75692842	0.91082077	0.03025975
ENSG000001	NAXE	0.7086264	0.88938447	0.03026587
ENSG000002	KCTD7	0.78991867	0.92279369	0.03032491
ENSG000002	AMY2B	0.75673019	0.91082077	0.03034087
ENSG000001	IL1B	0.79373342	0.92392642	0.03037132
ENSG000001	NDUFA13	0.77625863	0.91692938	0.03040953
ENSG000001	TFCP2L1	0.88949573	0.96178325	0.03041236
ENSG000001	ARMCX6	0.71135491	0.8906831	0.03043355
ENSG000001	PLA2G12A	0.76880886	0.91372907	0.03045873
ENSG000001	GOLPH3L	0.63278873	0.85440485	0.03049114
ENSG000001	NHSL1	0.77910802	0.91822435	0.03049168
ENSG000001	SNRPD2	0.83605964	0.94067016	0.03051215
ENSG000001	AARS2	0.71742646	0.89309227	0.03052219
ENSG000001	NMT1	0.41989372	0.73466853	0.03053376
ENSG000001	KCTD5	0.43671177	0.74496191	0.03060317
ENSG000001	CCND2	0.70989952	0.88993477	0.03064629
ENSG000001	CD58	0.71512489	0.89175724	0.03065519
ENSG000001	RPAP1	0.63237302	0.85418109	0.03077552
ENSG000001	TIMM50	0.66508831	0.86898991	0.03088601
ENSG000001	AIMP2	0.64674269	0.86096261	0.03091008
ENSG00000(	RARB	0.90680024	0.96841405	0.03094882

ENSG000001	PRSS27	0.86531616	0.95237042	0.03095562
ENSG000001	PTPRK	0.95397354	0.98478405	0.03097693
ENSG00000(	IL5RA	0.81435748	0.93216232	0.03098224
ENSG000001	RSAD1	0.72825814	0.89755262	0.03115509
ENSG000002	C13orf46	0.83326827	0.93878551	0.03116485
ENSG000001	PNP	0.63444418	0.85503542	0.03117167
ENSG000001	SF3B3	0.57307294	0.82235115	0.03119437
ENSG000001	CCDC59	0.78880208	0.92204735	0.03121148
ENSG000001	S100A1	0.81420048	0.93216232	0.03122436
ENSG000001	DOCK1	0.89439875	0.96404358	0.03122765
ENSG000001	C1S	0.84050384	0.94249039	0.031283
ENSG00000(	ATG16L1	0.63719505	0.85602759	0.03135235
ENSG000001	TMEM30B	0.85812842	0.94972607	0.03141315
ENSG00000(	CLCN4	0.75174584	0.908573	0.03141695
ENSG000001	YTHDF2	0.41015999	0.72951778	0.03145843
ENSG000001	OR7D2	0.92727472	0.97634133	0.03152229
ENSG000001	NABP2	0.69687767	0.88285035	0.03164234
ENSG000001	FBH1	0.35829662	0.69382497	0.03164848
ENSG00000(	FYN	0.65845439	0.86550135	0.03169509
ENSG000001	STEAP3	0.75408813	0.90944172	0.03171405
ENSG000001	GTF3A	0.74561093	0.9064753	0.03171587
ENSG000001	VPS4B	0.62250289	0.84922092	0.03174438
ENSG00000(	SEMA4G	0.73506368	0.90147373	0.03177052
ENSG000001	RNF145	0.47846337	0.77232426	0.03180927
ENSG000001	MPRIP	0.75806993	0.91101927	0.03181016
ENSG000002	DAXX	0.91299239	0.97123107	0.0318234
ENSG00000(	PPP5C	0.71298589	0.89118991	0.03183727
ENSG000002	ZNF350	0.67317691	0.87354948	0.03188147
ENSG000001	ETFA	0.68675798	0.88023892	0.03189107
ENSG000001	NF2	0.63928151	0.85691647	0.03190204
ENSG000001	SERTAD3	0.60115122	0.83799671	0.03191188
ENSG000001	RRP8	0.64863274	0.86206513	0.03194891
ENSG000001	SNX22	0.68884767	0.88059791	0.03197489
ENSG00000(	NUP50	0.61424438	0.8451435	0.03204514
ENSG000001	EXO5	0.73276724	0.89967021	0.03207716
ENSG000001	PARK7	0.63958542	0.85691647	0.03208815
ENSG00000(	TAF11	0.70222698	0.88553899	0.03209304
ENSG000001	KIAA2026	0.45783434	0.7575144	0.03211864
ENSG000001	TLR1	0.70026681	0.88463644	0.03213859
ENSG000001	TERF2IP	0.28065191	0.63927558	0.03215786
ENSG00000(	RABL2B	0.67755478	0.8751149	0.03216868
ENSG000001	SEC24B	0.53764394	0.80547376	0.03217657
ENSG000001	ITFG2	0.67340462	0.87354948	0.03218162

ENSG000001	LCLAT1	0.70689589	0.88858625	0.03218521
ENSG000001	NDUFA10	0.68867805	0.88057199	0.03218718
ENSG000001	LDOC1	0.77729694	0.91753458	0.03219412
ENSG00000C	TMEM98	0.94404571	0.98221647	0.0322842
ENSG000001	TPM3	0.30459641	0.65635638	0.0323117
ENSG000001	CD96	0.78875953	0.92204735	0.03233853
ENSG000001	IL18R1	0.74418613	0.90628646	0.03241977
ENSG000001	DCTPP1	0.71389034	0.89153258	0.03245425
ENSG000002	C2CD4D	0.85258708	0.94733063	0.03245557
ENSG000001	MRPL52	0.66467734	0.86885195	0.03246795
ENSG000001	PLCB1	0.78335592	0.91983554	0.0324789
ENSG000001	GPN2	0.62293169	0.84927918	0.03248398
ENSG000002	ARFGAP3	0.39245361	0.72027458	0.03252832
ENSG000001	NSD3	0.66314211	0.86792185	0.03252892
ENSG00000C	KIAA0100	0.36420838	0.6973647	0.03254046
ENSG000001	RPL35A	0.85567041	0.9486844	0.03254572
ENSG000001	GNG5	0.68827223	0.88051877	0.03254944
ENSG000001	XRCC6	0.52789187	0.80062313	0.03255863
ENSG000001	SF3B6	0.75793026	0.91091885	0.03256374
ENSG000001	C18orf54	0.79359217	0.92392642	0.03257131
ENSG00000C	MRPS18A	0.69275128	0.88158966	0.03258429
ENSG000001	SNED1	0.79824797	0.92603117	0.03263125
ENSG000001	ZSCAN25	0.59794196	0.83653142	0.03263898
ENSG000001	PLAC8L1	0.92908958	0.97671555	0.03266844
ENSG000001	RUSC1	0.61788835	0.84662697	0.03267282
ENSG000002	CDRT4	0.82277683	0.93539963	0.0326843
ENSG000002	GTF2I	0.5497176	0.81217374	0.03268983
ENSG000001	PIEZO1	0.63358866	0.85459721	0.03269248
ENSG000001	ARHGAP20	0.95206344	0.98418884	0.03270198
ENSG000001	ZNF438	0.7737311	0.91586084	0.03272033
ENSG000001	HIGD1B	0.92353922	0.97449352	0.03272346
ENSG00000C	CSDE1	0.44028579	0.74716978	0.0327966
ENSG00000C	TULP3	0.68862978	0.88057199	0.03279994
ENSG00000C	SNAP29	0.30406413	0.65635638	0.03281856
ENSG000001	INTS9	0.52948754	0.80131319	0.03282403
ENSG000001	CEP152	0.68796741	0.88051877	0.03282763
ENSG00000C	TIMM21	0.75624596	0.91071465	0.03285813
ENSG000001	PCGF1	0.5878593	0.83058401	0.03294659
ENSG000001	PIGR	0.95535175	0.98521399	0.03295776
ENSG000001	SLC8A1	0.7121337	0.89093319	0.03296418
ENSG000001	ELF2	0.5672719	0.81990332	0.03307291
ENSG000001	EFTUD2	0.49566552	0.78167453	0.03309235
ENSG000001	PPIL1	0.74488752	0.90628646	0.03309886

ENSG000001	UBLCP1	0.59663472	0.83600051	0.03310212
ENSG00000(	GLT8D1	0.6790373	0.87594571	0.03312911
ENSG000001	RNASEH2C	0.61201036	0.84406797	0.03315367
ENSG00000(	ASAP3	0.8283015	0.93662954	0.03318536
ENSG00000(	ATP11A	0.71674728	0.89261078	0.03318582
ENSG00000(	CCNK	0.44138569	0.74775474	0.03323717
ENSG000001	ACOT2	0.71463803	0.89175724	0.03323772
ENSG000001	PPRC1	0.553139	0.81385767	0.03325445
ENSG000001	CRADD	0.5747243	0.82341424	0.03326982
ENSG000001	SRP68	0.53455617	0.80406779	0.03328028
ENSG000001	CD300A	0.60010181	0.83754165	0.03336091
ENSG000001	SWT1	0.60192552	0.83845247	0.0333791
ENSG000001	P2RX4	0.57288739	0.82223748	0.03341365
ENSG000002	PATL2	0.63974464	0.85691647	0.03343949
ENSG00000(	TKTL1	0.73178063	0.89916258	0.03344651
ENSG000001	CTSZ	0.49580607	0.78182025	0.03345622
ENSG000001	PEX11G	0.76793003	0.91357827	0.03346049
ENSG000002	ST20-MTHFS	0.93234889	0.97787927	0.03348015
ENSG000001	MAP3K19	0.94880245	0.98401286	0.03348208
ENSG000001	NDUFS4	0.8626771	0.951353	0.03349221
ENSG000002	RIMBP3	0.78247903	0.9193471	0.03350695
ENSG000001	UBE3B	0.37976171	0.70965485	0.03351288
ENSG000001	MEFV	0.70742605	0.88887192	0.03351577
ENSG00000(	MED24	0.58950375	0.831894	0.03353308
ENSG000001	KCTD17	0.68217669	0.87748577	0.03360669
ENSG000001	ACYP2	0.74964824	0.90788681	0.033645
ENSG000001	DNAJA4	0.53072081	0.80212192	0.033692
ENSG000001	SMAD2	0.51487706	0.79420539	0.03369637
ENSG000001	SNX3	0.57281641	0.82223748	0.03371555
ENSG000001	USP54	0.74491757	0.90628646	0.03371984
ENSG00000(	ATP6V1H	0.40887384	0.72866694	0.03372851
ENSG000001	ZNF44	0.6744509	0.87427601	0.03375589
ENSG000001	LIM2	0.92327734	0.97437154	0.03376649
ENSG000001	ICMT	0.62735427	0.85113229	0.03376781
ENSG000001	COX16	0.83472501	0.93977114	0.03377349
ENSG000001	PIK3R4	0.53165575	0.80232066	0.03377364
ENSG000001	HSD17B4	0.51316188	0.79293961	0.03387371
ENSG000001	MIER2	0.52668023	0.80019714	0.03391455
ENSG000001	EPS8L2	0.76396859	0.91240328	0.03398469
ENSG000001	NRSN2	0.82482188	0.93591669	0.0339868
ENSG000001	LMTK2	0.76139023	0.91150138	0.03403135
ENSG00000(	NDUFB4	0.68136796	0.87687561	0.03403395
ENSG000001	CNNM1	0.88585031	0.96095764	0.03405726

ENSG000001	PIK3CG	0.61340876	0.8451435	0.03406194
ENSG000001	SRSF4	0.13362515	0.49240713	0.03411055
ENSG00000(	CYB5R4	0.61518688	0.84533626	0.03423432
ENSG000001	DKC1	0.53265145	0.80280163	0.0342568
ENSG000001	SEC11A	0.66694646	0.87009558	0.03433372
ENSG000001	SIK2	0.62229812	0.84916838	0.03435487
ENSG000001	LONRF3	0.76013596	0.91136366	0.03436619
ENSG000001	SATB2	0.83744178	0.94120179	0.03437059
ENSG000001	PROCA1	0.64641677	0.86074045	0.03438153
ENSG000001	PYM1	0.65506008	0.86442301	0.03441592
ENSG000002	CCDC85C	0.5491721	0.81188267	0.03443532
ENSG000002	DPP3	0.55587613	0.81467287	0.03445291
ENSG000001	EIF1AD	0.4522127	0.75401289	0.0344589
ENSG000001	ALG5	0.75538617	0.91028686	0.03446955
ENSG000001	ITGA2	0.79016698	0.92280247	0.03449092
ENSG000001	CHD1L	0.69111836	0.88130753	0.03457005
ENSG00000(	WAC	0.37159164	0.70378624	0.03457042
ENSG000001	ODF2	0.50073845	0.7851753	0.03458231
ENSG000001	KIAA1191	0.46856377	0.76593878	0.03460583
ENSG000001	RRM1	0.69546987	0.88231682	0.03466227
ENSG000001	HVCN1	0.43988583	0.74656941	0.03467281
ENSG000001	HNRNPA1	0.68613068	0.8799229	0.03467348
ENSG000002	N/A	0.79862179	0.92603117	0.03468125
ENSG00000(	FH	0.63416663	0.8549455	0.03469777
ENSG000001	PCCB	0.6010919	0.83799671	0.03470412
ENSG000001	GAPVD1	0.48141565	0.77454874	0.0347733
ENSG000001	MCM3	0.60420185	0.83883394	0.03478983
ENSG000001	POP4	0.57998834	0.82610777	0.03482719
ENSG000001	TM9SF1	0.62245237	0.84922092	0.03491028
ENSG000001	CEMIP2	0.58771174	0.83052006	0.03491477
ENSG000001	TIMD4	0.86598649	0.95247761	0.03501577
ENSG000001	MAGEB17	0.93874253	0.97961749	0.03502625
ENSG000001	POLR2H	0.72388424	0.8959985	0.03503118
ENSG000001	SPDYE1	0.72992945	0.89828814	0.03503169
ENSG000001	SLC22A23	0.7437011	0.906159	0.03504532
ENSG000001	COQ3	0.75431244	0.90946544	0.03505018
ENSG00000(	FAM168A	0.40130601	0.72606468	0.03505643
ENSG000001	KLHL24	0.63359248	0.85459721	0.03506871
ENSG000001	NIPSNAP3B	0.84942427	0.94625949	0.0350996
ENSG000001	RAB3IP	0.70901532	0.88947345	0.03514135
ENSG000001	CCDC3	0.91711065	0.97245317	0.03515252
ENSG000001	CD81	0.66780978	0.87074767	0.03521974
ENSG000001	SPSB3	0.92833602	0.97652064	0.03524366

ENSG000001	STK17A	0.56267333	0.81828274	0.0352654
ENSG00000C	MRTO4	0.65846759	0.86550135	0.03533639
ENSG000001	GARNL3	0.84165404	0.94283868	0.0353704
ENSG000002	PIK3R6	0.75653553	0.91082077	0.03539522
ENSG00000C	ST3GAL6	0.71577365	0.89214837	0.03542106
ENSG000001	TXLNB	0.80944478	0.93030173	0.03544406
ENSG00000C	VPS35	0.30453025	0.65635638	0.03544492
ENSG00000C	TRAF4	0.71674936	0.89261078	0.03546103
ENSG000001	WWOX	0.67987472	0.87632975	0.03549083
ENSG000001	HPS4	0.63399286	0.85478221	0.03549382
ENSG000001	EIF5B	0.57718614	0.82460163	0.03550195
ENSG000001	PBLD	0.67253411	0.87337134	0.03551671
ENSG000001	NETO1	0.86638263	0.95264796	0.03553155
ENSG000001	CEP97	0.77449513	0.91617933	0.03553622
ENSG000001	DYNC1H1	0.53570888	0.80423066	0.03555062
ENSG000001	EPM2A	0.73912333	0.90383684	0.0355594
ENSG000001	GPRASP2	0.80968091	0.93041677	0.03556106
ENSG000001	NT5DC1	0.72845652	0.89755262	0.03558127
ENSG000001	FZD9	0.93753027	0.97932829	0.03561677
ENSG000001	COX8A	0.63278984	0.85440485	0.03561834
ENSG00000C	MAP2	0.92507859	0.97521605	0.03562704
ENSG000001	CRB2	0.82003774	0.93468999	0.03566587
ENSG000001	RBM25	0.52978973	0.8016768	0.03569019
ENSG000001	FAM161A	0.8903824	0.9619495	0.03569881
ENSG000001	TMEM176B	0.6895237	0.88074719	0.03574783
ENSG000001	SLC5A4	0.93031945	0.97723213	0.03584278
ENSG000001	HS3ST3B1	0.77026554	0.91423988	0.03586025
ENSG000001	MAPK11	0.7589429	0.91112428	0.0358636
ENSG000001	MYO1A	0.83808932	0.94146888	0.03587027
ENSG000001	A2ML1	0.92701	0.9761892	0.03588455
ENSG000001	CAMTA1	0.65081421	0.86333374	0.03588698
ENSG000001	GARS1	0.46734423	0.76533215	0.03591051
ENSG000001	CTTNBP2NL	0.67045125	0.87207538	0.03591115
ENSG000001	ZNF518B	0.6080583	0.84146691	0.03591198
ENSG000001	KIAA1522	0.70418432	0.88668793	0.03593948
ENSG000001	RNF135	0.42626643	0.73858585	0.03595027
ENSG000001	IMMT	0.49756896	0.78277562	0.035962
ENSG000001	C17orf58	0.72435118	0.89623486	0.03602602
ENSG000001	PEX11B	0.53965989	0.80698777	0.03602859
ENSG000001	GMPPB	0.62139206	0.84862344	0.03604085
ENSG000001	TTC36	0.92891432	0.9766111	0.03607148
ENSG000001	TMEM44	0.74177429	0.90543757	0.03610477
ENSG000001	MAPKAP1	0.38476821	0.71310141	0.03611368

ENSG00000C	PIGV	0.43835487	0.74584177	0.03613178
ENSG000002	N/A	0.65639359	0.86501605	0.03613506
ENSG000001	FAM222B	0.51038723	0.79143652	0.03613877
ENSG000001	BTG2	0.23582941	0.60330268	0.03615735
ENSG000001	AKIP1	0.69696832	0.88285035	0.03616999
ENSG000001	B3GNT3	0.90472388	0.96810345	0.03617482
ENSG000001	AMN1	0.76045019	0.91141904	0.03618545
ENSG000002	PDE7A	0.75903995	0.91117342	0.03619292
ENSG000001	EBI3	0.86304463	0.95166422	0.03620793
ENSG000001	RFC5	0.55941095	0.81696069	0.03622692
ENSG000001	LRP5	0.76735201	0.91352929	0.03627314
ENSG000001	MPV17	0.54206571	0.80808176	0.03629204
ENSG000001	PSMD7	0.48702209	0.77767904	0.03631768
ENSG000001	KCNQ5	0.82323661	0.93539963	0.03633658
ENSG000001	ZSCAN22	0.61746258	0.84653095	0.03637999
ENSG000001	SNX17	0.47156519	0.76694135	0.03642808
ENSG000001	THNSL1	0.7954739	0.92462665	0.03646436
ENSG000001	TOR1AIP2	0.26000225	0.62338681	0.036551
ENSG000001	EPAS1	0.76061528	0.91141904	0.03655197
ENSG000001	TMEM145	0.92543034	0.97525983	0.03658205
ENSG000001	ZPBP2	0.93642078	0.97918399	0.03659586
ENSG000001	C7orf50	0.63486172	0.85504577	0.03662707
ENSG00000C	NIPAL3	0.68792555	0.88051877	0.03663786
ENSG000002	TAS2R39	0.92850962	0.97652064	0.03667225
ENSG000001	TMEM254	0.61858297	0.84701734	0.03672374
ENSG000001	VDAC2	0.5311407	0.80232066	0.03675342
ENSG000001	ZNF689	0.45714768	0.75734179	0.03675459
ENSG000001	PSMD11	0.38475155	0.71310141	0.03679511
ENSG00000C	GNB5	0.66798725	0.87080466	0.03679913
ENSG000001	ZSCAN23	0.94386122	0.98221647	0.03682585
ENSG000001	POLR3A	0.48964802	0.77987726	0.0368285
ENSG000001	GDPD5	0.65524583	0.86446832	0.03683442
ENSG000001	FADS1	0.65446393	0.86439091	0.03683895
ENSG000001	ADAM17	0.57682303	0.8243883	0.03684865
ENSG000001	TVP23C	0.76096778	0.91141904	0.03686245
ENSG000001	ZEB1	0.74515907	0.90628646	0.03688594
ENSG00000C	NCDN	0.58373188	0.82875175	0.03688614
ENSG000001	SRRM1	0.24543831	0.61141177	0.03692129
ENSG000001	MRM3	0.6412948	0.8575293	0.03693663
ENSG000001	GADD45GIP1	0.68054726	0.87650083	0.03695692
ENSG000001	EIF4H	0.14163914	0.50055578	0.03700048
ENSG000001	NLRP3	0.39010831	0.71927554	0.03701729
ENSG00000C	PSME4	0.47516792	0.76999017	0.03703855

ENSG000002	NBPF14	0.52567748	0.79977109	0.03706303
ENSG000001	ZNF713	0.75562048	0.91043408	0.03707406
ENSG000001	FAF2	0.39180045	0.71972665	0.03708862
ENSG000001	FAM78A	0.34242082	0.68336859	0.03710524
ENSG000001	PJA1	0.64080914	0.85735151	0.03712969
ENSG000002	SCART1	0.76945077	0.91386073	0.03713338
ENSG000001	MYOZ1	0.91999352	0.97328217	0.03713741
ENSG000001	GYG1	0.39132373	0.71964297	0.03714455
ENSG000001	TMEM53	0.69700429	0.88285035	0.03715921
ENSG000001	ZNF296	0.58033917	0.82628613	0.03720655
ENSG000001	UQCRFS1	0.58026705	0.8262559	0.0372118
ENSG000001	CCL22	0.93832771	0.97954033	0.03723044
ENSG000001	LDLRAD3	0.77293866	0.91567233	0.03728976
ENSG000001	UBE2E3	0.41989237	0.73466853	0.03730606
ENSG000001	MAP3K10	0.62543377	0.85037415	0.03736412
ENSG000001	SASH3	0.49182844	0.7804495	0.03736805
ENSG000002	ZNF324B	0.66771649	0.87074767	0.03742022
ENSG000001	GALNT7	0.69822994	0.8835822	0.03743032
ENSG000002	PROB1	0.7492784	0.90770972	0.0374556
ENSG00000C	ITIH1	0.84868844	0.94591943	0.03746372
ENSG000001	NIN	0.59208561	0.83379828	0.03746789
ENSG000001	GMDS	0.56911392	0.82037292	0.03751792
ENSG000001	SLC38A7	0.5667252	0.8197023	0.03754203
ENSG000001	USF1	0.53119542	0.80232066	0.03761452
ENSG00000C	FAM120A	0.45199476	0.75395007	0.03761854
ENSG000001	SRD5A3	0.71141212	0.8906831	0.03762613
ENSG000001	PTDSS1	0.41907235	0.73426975	0.03762984
ENSG000001	SSUH2	0.93206905	0.97784797	0.0376632
ENSG000001	EIF3C	0.84711686	0.94527257	0.03768817
ENSG000001	FOXP4	0.63449663	0.85503542	0.03769809
ENSG000001	SMIM8	0.76183602	0.91151487	0.03769929
ENSG00000C	ITPR3	0.72609927	0.89703065	0.03776043
ENSG000001	CZIB	0.58966697	0.83191276	0.03776579
ENSG000001	KIAA0586	0.67260089	0.87337134	0.03776678
ENSG000001	SNIP1	0.23028261	0.59855451	0.03778178
ENSG000002	HAUS5	0.72204596	0.89515616	0.03787851
ENSG00000C	CEP68	0.70903177	0.88947345	0.0378837
ENSG000001	SRSF12	0.80637546	0.92964486	0.03791676
ENSG000002	N/A	0.93282681	0.97819097	0.03791911
ENSG00000C	MEOX1	0.75239715	0.908573	0.03795909
ENSG000001	CCT7	0.54225438	0.80815796	0.037975
ENSG00000C	VPS41	0.38315459	0.71206146	0.03799918
ENSG000001	BRWD3	0.65458848	0.86439091	0.03803812

ENSG000001	RPN2	0.40295038	0.72672584	0.03805076
ENSG000002	LEPROT	0.46641513	0.76486809	0.03806355
ENSG000001	NCOA5	0.41095919	0.72989984	0.03807309
ENSG000001	NEURL1	0.72035308	0.8946285	0.03807672
ENSG000001	EIF3E	0.81692597	0.93280078	0.03809758
ENSG000001	KLHL36	0.7059486	0.88780148	0.03812217
ENSG000001	MRPS27	0.67214101	0.87316437	0.03817378
ENSG000001	TELO2	0.61550904	0.84537475	0.03821133
ENSG000001	USP49	0.66493089	0.86898991	0.03822516
ENSG000001	NSD2	0.64398449	0.85940463	0.03822525
ENSG000001	ATOX1	0.51717784	0.79538014	0.03827198
ENSG000001	NDUFAF6	0.72888277	0.89755543	0.03832996
ENSG00000C	STK17B	0.59094438	0.83284123	0.03833497
ENSG000001	HARBI1	0.52705233	0.80038735	0.03834295
ENSG00000C	MAP3K4	0.60576662	0.83946052	0.03837525
ENSG000001	ILKAP	0.54812846	0.81141647	0.03838142
ENSG000001	PCGF6	0.8096127	0.93040424	0.038396
ENSG000001	WASHC2C	0.27043881	0.63178344	0.03846012
ENSG000001	POLR2D	0.56857123	0.82037292	0.03847507
ENSG000001	TNFRSF11A	0.76551448	0.91260342	0.03848932
ENSG000001	ANKRD36B	0.79881853	0.92603117	0.03849704
ENSG000001	DIDO1	0.46808504	0.76574496	0.03851785
ENSG000001	SLIRP	0.77595284	0.91686844	0.03855543
ENSG000001	PWWP2B	0.6449738	0.85991395	0.03855748
ENSG000001	SNX15	0.72204249	0.89515616	0.03858532
ENSG000001	NKAP	0.50382953	0.78675515	0.03863616
ENSG000001	SNRPD3	0.64555911	0.8600572	0.0386509
ENSG000001	EGFL6	0.89410071	0.96404358	0.03866705
ENSG000001	CIAO1	0.49810946	0.78286233	0.03874435
ENSG00000C	CSNK2A2	0.50005918	0.78433741	0.0388075
ENSG000001	CTR9	0.31096502	0.65949478	0.03885851
ENSG000001	ZDHHC16	0.62882578	0.85220301	0.03890025
ENSG000001	NEIL1	0.67661414	0.87490088	0.03890293
ENSG000002	SOCS7	0.71057145	0.89038035	0.03892245
ENSG000001	FGD4	0.70362924	0.8864702	0.03892551
ENSG000001	TMEM219	0.5208291	0.79735075	0.03892732
ENSG000001	MPLKIP	0.5541524	0.81405705	0.03894779
ENSG000001	YOD1	0.75064897	0.90815046	0.03900222
ENSG000001	CAT	0.66518911	0.86898991	0.03907881
ENSG000001	CCNG2	0.68956587	0.88074719	0.03910945
ENSG00000C	TRAF3IP3	0.62460909	0.84996462	0.0391529
ENSG000001	PRMT2	0.53045451	0.80199905	0.03916632
ENSG000001	UGP2	0.49046348	0.7800506	0.03916927

ENSG000001	MOAP1	0.89659786	0.96490939	0.03918519
ENSG000001	ANKRD36C	0.80707538	0.92967903	0.03920429
ENSG00000(	SEMA5B	0.92935114	0.97675413	0.03934559
ENSG000001	FAM118B	0.60851266	0.84186486	0.03936816
ENSG000001	PSPC1	0.45023913	0.75290713	0.03938865
ENSG000001	ZDHHC24	0.58212859	0.82746456	0.03939191
ENSG000001	GPR34	0.7187274	0.89370323	0.0393957
ENSG000001	COPB2	0.1945182	0.56193152	0.03939717
ENSG000001	DESI1	0.24135051	0.60721888	0.03948314
ENSG000001	KANSL2	0.68827642	0.88051877	0.03948466
ENSG000001	RRP1B	0.57501709	0.82344878	0.03950822
ENSG00000(	GCN1	0.51594344	0.79504352	0.03955885
ENSG000001	FLI1	0.39203756	0.71994497	0.03959829
ENSG000001	CCDC12	0.55288842	0.81383402	0.03960118
ENSG00000(	CCDC88C	0.58095364	0.82679852	0.03984555
ENSG000001	C1QBP	0.65827646	0.86546032	0.03984628
ENSG00000(	ALDH18A1	0.62149551	0.84864373	0.03985664
ENSG000002	N/A	0.82756828	0.93660278	0.03986434
ENSG000001	SPATC1	0.83554929	0.94026854	0.03987646
ENSG000001	FIBP	0.57150517	0.82164253	0.03988037
ENSG00000(	SLC7A8	0.7811304	0.9189743	0.039886
ENSG000001	GLIS2	0.9030123	0.96763453	0.03995649
ENSG000001	ATP6AP2	0.40252579	0.72669629	0.03997768
ENSG000002	TRIQK	0.6919063	0.88155074	0.04001683
ENSG00000(	PIAS1	0.38163386	0.7106995	0.04002709
ENSG00000(	SNX1	0.10191721	0.45128146	0.04003332
ENSG000002	N/A	0.92238718	0.97410521	0.04004955
ENSG000001	CEP164	0.43947529	0.74621708	0.0400626
ENSG000001	GOLPH3	0.37441135	0.70642398	0.0400802
ENSG000001	GPAT2	0.75846321	0.91108587	0.04011303
ENSG000001	SIRT5	0.61096802	0.84334674	0.04014406
ENSG000001	PDLIM3	0.91303018	0.97123107	0.04015074
ENSG000001	ZNF232	0.75698891	0.91082077	0.04015671
ENSG000001	HAX1	0.56671442	0.8197023	0.04016653
ENSG000001	ATP8B2	0.69695653	0.88285035	0.04020165
ENSG000001	PSIP1	0.70603938	0.88780148	0.04022446
ENSG000001	NDUFA8	0.66137143	0.86736018	0.04025709
ENSG000001	RPRD1A	0.62743724	0.8511716	0.04026696
ENSG000001	ANKRD37	0.7847274	0.92037119	0.04027671
ENSG000001	ATIC	0.58642049	0.82959975	0.04029883
ENSG000001	RRAGC	0.4094469	0.72902686	0.0403
ENSG000001	POP5	0.74518706	0.90628646	0.04032107
ENSG00000(	ARCN1	0.26867046	0.63079288	0.04032734

ENSG000001	ALCAM	0.67839781	0.87557173	0.04034297
ENSG000001	HKDC1	0.71554718	0.89204237	0.04034952
ENSG00000C	SNRNP40	0.56186661	0.81801588	0.04035392
ENSG000001	NONO	0.3482703	0.68768877	0.04036041
ENSG00000C	NUCB2	0.76906647	0.91375182	0.04049217
ENSG000001	KYAT3	0.53604211	0.80424764	0.04050032
ENSG00000C	FAM214A	0.73399206	0.90054625	0.0405585
ENSG000001	SNAPC2	0.50313683	0.78652767	0.04056064
ENSG00000C	EIF3I	0.58357756	0.82875175	0.04056863
ENSG000001	PDGFA	0.78118372	0.9189743	0.04057111
ENSG000001	FZD6	0.78411277	0.92011557	0.04058573
ENSG000001	LATS2	0.6290322	0.8524116	0.0405944
ENSG00000C	USP13	0.74580933	0.90651247	0.04059972
ENSG000001	CABLES2	0.38004112	0.70987972	0.04063392
ENSG00000C	MYLIP	0.39691196	0.72308765	0.04064249
ENSG000002	THTPA	0.64021265	0.85706063	0.04065747
ENSG000001	ADNP	0.54112534	0.80757124	0.04071149
ENSG000001	ADA	0.68677055	0.88023892	0.04071235
ENSG000001	PLEKHA7	0.76876345	0.91372907	0.04072129
ENSG000001	PRR14L	0.43668637	0.74496191	0.04076908
ENSG000001	ADAMTSL3	0.92044694	0.97357155	0.04077851
ENSG00000C	ST6GAL1	0.59412283	0.83456808	0.0408041
ENSG000002	ZNF134	0.56261111	0.81826556	0.04083525
ENSG000001	SERPINI2	0.86560323	0.95237042	0.04084652
ENSG000001	SUDS3	0.235083	0.60232491	0.04085643
ENSG000001	VMAC	0.66653214	0.87009558	0.04087185
ENSG00000C	BRD9	0.54296059	0.8084284	0.04093716
ENSG00000C	EDEM2	0.26391462	0.62728666	0.04098113
ENSG000001	TMEM33	0.4628353	0.76248547	0.04101758
ENSG00000C	SLC25A5	0.55502648	0.81433077	0.041065
ENSG000001	PARP4	0.3118842	0.66057537	0.0410656
ENSG000001	ACSF3	0.58166361	0.82722887	0.04107553
ENSG000001	NOMO2	0.57431473	0.82327493	0.04108404
ENSG000001	AZIN1	0.5786649	0.82549482	0.04115336
ENSG000002	SRSF8	0.68320006	0.87817601	0.04115796
ENSG00000C	TP53BP1	0.5339552	0.80362357	0.04117643
ENSG000001	NAT10	0.52421867	0.79877426	0.0411782
ENSG000001	DENND2D	0.67036444	0.87207538	0.04118124
ENSG000001	FTSJ3	0.49496155	0.78124152	0.04131481
ENSG000001	NUDT11	0.8381416	0.94146888	0.04134475
ENSG000002	CFAP44	0.80666046	0.92967903	0.0413696
ENSG000002	NPIPB5	0.45182144	0.75386982	0.04142759
ENSG000001	ZNF397	0.58696818	0.83026416	0.04143051

ENSG000001	MAP7D3	0.50898999	0.79132674	0.0415215
ENSG000001	MFSD4B	0.6896041	0.88074719	0.04155733
ENSG000001	SLC35G3	0.93139518	0.97763745	0.04157114
ENSG000001	SUN3	0.90236793	0.96736749	0.04158077
ENSG000002	ZNF2	0.54531017	0.80952117	0.0416238
ENSG000001	TIPARP	0.3793598	0.70965485	0.04166057
ENSG000001	ACY3	0.837156	0.94120179	0.04171602
ENSG000001	RPS6KA3	0.60544442	0.83945694	0.04174861
ENSG000001	CHAMP1	0.44701993	0.75099423	0.04178167
ENSG000001	ARHGEF19	0.65740702	0.865228	0.04179623
ENSG000001	SLC52A2	0.51141809	0.79175206	0.0418045
ENSG000001	IGFBP7	0.65010109	0.86293893	0.04182271
ENSG000001	TAMM41	0.68001023	0.87643484	0.04184329
ENSG000001	CNOT11	0.33058008	0.67415584	0.0418482
ENSG000001	ARHGEF7	0.44174296	0.74807889	0.04187788
ENSG000001	LURAP1	0.90370379	0.96790491	0.04188981
ENSG000001	STAMBP	0.31986593	0.66679459	0.04190906
ENSG000001	XPO5	0.40452531	0.72672584	0.0419255
ENSG000001	RCAN3	0.74218216	0.90555138	0.0419829
ENSG000001	EPC1	0.36557202	0.69837111	0.04199881
ENSG000001	RPS10	0.79539241	0.92462665	0.04200687
ENSG000001	TFCP2	0.29823858	0.65366489	0.04200817
ENSG000001	TCHP	0.43910257	0.74609757	0.04212815
ENSG00000(	TXLNA	0.2966869	0.65217594	0.04213624
ENSG000001	HBP1	0.49509874	0.78124152	0.04215478
ENSG000001	ECT2	0.72107543	0.89490971	0.04218388
ENSG000001	EXT1	0.477478	0.77191738	0.04219322
ENSG000002	RNPS1	0.4187259	0.7339003	0.04219624
ENSG000001	ALG14	0.71244313	0.89114391	0.04222516
ENSG000001	AKAP10	0.4396101	0.7462571	0.04222697
ENSG000001	KCTD15	0.59506693	0.83538836	0.04224492
ENSG000001	PRKAB1	0.23548081	0.60269577	0.04225589
ENSG000001	EIF2B2	0.56234917	0.81817772	0.04228925
ENSG000002	LIPJ	0.93400961	0.97846361	0.04229234
ENSG000001	MREG	0.74859118	0.90731063	0.04229632
ENSG000001	ADGB	0.91609543	0.9722662	0.04238926
ENSG00000(	SYNJ2	0.51922855	0.7962518	0.04239039
ENSG000001	UQCC1	0.59618701	0.83586476	0.04246636
ENSG000002	SMIM27	0.61199832	0.84406797	0.04246929
ENSG000001	ABCC10	0.52925983	0.8011926	0.04247714
ENSG000001	EIF2B5	0.49140578	0.78031845	0.04249668
ENSG000001	TSGA10	0.77049781	0.91431506	0.04249826
ENSG000001	TMEM186	0.53465642	0.80406779	0.04251723

ENSG000002	FAM185A	0.66515974	0.86898991	0.04253452
ENSG000001	LZIC	0.56395402	0.81864675	0.04254337
ENSG000001	OGA	0.58579133	0.82959975	0.042555
ENSG000001	UMPS	0.65239255	0.86379349	0.04258686
ENSG00000C	TUBG2	0.69970742	0.88452288	0.04263201
ENSG000001	BTNL9	0.87679805	0.9567976	0.04267891
ENSG000001	IGLON5	0.91177887	0.9710451	0.04269734
ENSG000001	EMC8	0.4259907	0.73856395	0.04271352
ENSG000001	ACAD9	0.50258005	0.78616496	0.04273869
ENSG000001	NFE2L2	0.31318622	0.6613866	0.04275337
ENSG00000C	RSF1	0.31984833	0.66679459	0.04280149
ENSG000001	LPAR5	0.60665996	0.84018575	0.04281309
ENSG000001	PPIA	0.68047104	0.87647218	0.04286999
ENSG000001	CREB3	0.44410621	0.7491529	0.04288729
ENSG00000C	TDRD3	0.63024579	0.85293934	0.04291467
ENSG00000C	MACROH2A2	0.69029267	0.88074719	0.04291506
ENSG000001	IGDCC4	0.88603006	0.96095764	0.04291853
ENSG000002	CHMP4A	0.62763838	0.8511716	0.04292583
ENSG000001	LY96	0.74517818	0.90628646	0.04295423
ENSG000001	RNF34	0.19579094	0.56346286	0.04303045
ENSG00000C	PPP2R3A	0.69941614	0.88434908	0.04305111
ENSG000001	CPEB4	0.71278789	0.89116324	0.0430579
ENSG000001	KCNN3	0.88754545	0.96125103	0.04308519
ENSG00000C	XRCC5	0.35499385	0.6919142	0.04308848
ENSG000001	TOMM7	0.83133011	0.938023	0.04311055
ENSG000001	CLEC7A	0.52193022	0.79776689	0.04319472
ENSG000001	HOXB2	0.69993047	0.88452288	0.04327022
ENSG000001	ANKRD16	0.61755741	0.84654112	0.04327142
ENSG000001	MRPL24	0.6160506	0.84569598	0.04328359
ENSG000001	CELF1	0.48565648	0.7774422	0.04329957
ENSG000001	IAH1	0.63615322	0.85549278	0.04330892
ENSG000001	LRRC1	0.6949853	0.88222003	0.04332651
ENSG000001	IL21R	0.6768769	0.87503585	0.04341311
ENSG00000C	NDC80	0.74616429	0.90653584	0.04341456
ENSG000002	TMEM272	0.76749148	0.91352929	0.04348255
ENSG000001	B9D1	0.81130044	0.93148672	0.04348917
ENSG000001	KANK1	0.65412295	0.86439091	0.04349105
ENSG000001	MTF1	0.49512216	0.78124152	0.0435155
ENSG000001	IKZF1	0.25190661	0.61748303	0.04354325
ENSG000001	FGD1	0.60689564	0.84023871	0.04354421
ENSG000001	NUDT17	0.64730706	0.86129024	0.04357506
ENSG000001	CD207	0.92549533	0.97525983	0.04357614
ENSG000001	SLC10A1	0.82389723	0.93569416	0.04359358

ENSG000001	TIMM44	0.6221731	0.84906914	0.04361667
ENSG000001	PTDSS2	0.67551268	0.87451032	0.04362421
ENSG00000(	OGFOD1	0.52986268	0.8016768	0.04363167
ENSG000001	ZNF503	0.68609892	0.8799229	0.04364252
ENSG000001	PTGIR	0.55940385	0.81696069	0.04365923
ENSG000002	CPT1B	0.71297625	0.89118991	0.04367664
ENSG000001	LEO1	0.52735229	0.80061891	0.0437163
ENSG00000(	TNK2	0.6130208	0.84505867	0.04372733
ENSG000001	USF3	0.53682449	0.80491451	0.04373143
ENSG000001	ZNF827	0.7601772	0.91136366	0.04376489
ENSG000001	SUMO1	0.54448112	0.80914698	0.04378103
ENSG000001	PDE6G	0.69393888	0.88206401	0.04379403
ENSG000002	CSNK2B	0.92371995	0.97462088	0.04379966
ENSG000001	ID2	0.56929423	0.82041807	0.04381786
ENSG000002	CRYZL1	0.64024079	0.85706063	0.04383966
ENSG000001	ADGRF3	0.77475443	0.91628579	0.04384004
ENSG000001	CDK12	0.27845137	0.63759905	0.04386867
ENSG000001	RASSF4	0.52411348	0.79870477	0.04391849
ENSG000001	CCDC93	0.54445157	0.80914698	0.04395621
ENSG000001	SUPT3H	0.73997543	0.90460632	0.04395632
ENSG000001	ABCC4	0.59308787	0.83444495	0.0439604
ENSG000002	SELENOH	0.623499	0.84952196	0.04399654
ENSG000001	UFSP2	0.63485173	0.85504577	0.04399928
ENSG000001	TINAGL1	0.91680381	0.97237856	0.04400049
ENSG000001	PAIP2	0.53002109	0.8016768	0.04400405
ENSG000001	LGALS2	0.65577013	0.86467033	0.04400739
ENSG000001	INTS4	0.25341671	0.61912837	0.04402483
ENSG00000(	TTC19	0.64657382	0.86080839	0.04402851
ENSG000001	CKAP2	0.63680922	0.85580792	0.04403453
ENSG000001	AHSA1	0.40741481	0.72768322	0.04406865
ENSG000001	STX16	0.38602415	0.715021	0.0441086
ENSG000001	TFDP2	0.58116576	0.82702791	0.04415306
ENSG000001	L3MBTL1	0.64420423	0.85955658	0.04418671
ENSG000001	POLR3F	0.68703974	0.88032389	0.04420703
ENSG000001	BSN	0.72568486	0.89686038	0.04422207
ENSG000001	ARNTL	0.42756425	0.73957182	0.04424924
ENSG000001	CTU2	0.67926037	0.87598276	0.04425185
ENSG000001	CCNH	0.63536764	0.85504577	0.04426606
ENSG000001	SIDT2	0.42421182	0.73732623	0.04426677
ENSG000001	SNW1	0.33910416	0.68041384	0.04428028
ENSG000001	PRKD2	0.69130898	0.88131945	0.04428047
ENSG000001	FRAS1	0.87902637	0.95817324	0.04428872
ENSG000001	TET3	0.52519827	0.79936321	0.04429245

ENSG000001	RNF111	0.52379062	0.79870477	0.04432751
ENSG000001	AFG1L	0.76456403	0.91243616	0.04439082
ENSG000001	SUN1	0.65181101	0.86353259	0.04443338
ENSG000001	ARMCX2	0.67553567	0.87451032	0.04443402
ENSG000001	TLNRD1	0.4908789	0.7800506	0.04446512
ENSG000002	BAHCC1	0.62476095	0.85010002	0.04446753
ENSG000001	CLEC12A	0.63026213	0.85293934	0.04446761
ENSG000002	ZDHHC11B	0.79078536	0.92297405	0.04446943
ENSG000001	NEK9	0.36517284	0.69805879	0.04447629
ENSG000001	CLTA	0.42014853	0.73468557	0.04448738
ENSG000001	C19orf44	0.73633317	0.90186782	0.04450079
ENSG000001	IKZF3	0.63137071	0.85334691	0.04450767
ENSG000001	LY86	0.59896101	0.83680347	0.04454457
ENSG00000C	SEL1L	0.58751918	0.83046863	0.04455436
ENSG00000C	ZFHX4	0.91387885	0.97135384	0.04457676
ENSG000001	PPP1R2	0.52368233	0.79870477	0.04464649
ENSG000001	VHL	0.3329567	0.67532519	0.04464957
ENSG000001	LSM7	0.66399735	0.86837979	0.04470381
ENSG000001	HNRNPD	0.25124233	0.61695463	0.04477756
ENSG000001	TAF3	0.4757635	0.7703596	0.04478413
ENSG00000C	ABL1	0.4917315	0.7804495	0.04478583
ENSG000001	NEMF	0.5668646	0.8197023	0.04479269
ENSG00000C	CYB561	0.65269953	0.86379349	0.04481465
ENSG000001	ITK	0.7090387	0.88947345	0.04485413
ENSG00000C	NUFIP1	0.64407535	0.85945525	0.04487886
ENSG000001	NDUFB5	0.60381638	0.83883394	0.0449096
ENSG000001	CFAP53	0.84295517	0.94328484	0.04492236
ENSG000002	GIMAP1	0.50206237	0.78558996	0.044947
ENSG00000C	ISOC1	0.43953482	0.74621708	0.04495321
ENSG000001	ZNF101	0.59555274	0.83572077	0.04498831
ENSG000001	ZBED4	0.59367573	0.83456808	0.04499644
ENSG00000C	NOS2	0.91541737	0.97217816	0.04504909
ENSG000001	SLC35C1	0.37726073	0.7086984	0.04506264
ENSG000001	ERCC5	0.58238066	0.82774114	0.04509429
ENSG000001	RUSF1	0.55341519	0.81385767	0.04511086
ENSG000001	WNT2B	0.73021721	0.89835861	0.04517904
ENSG00000C	SEC31B	0.61939725	0.84711589	0.04523722
ENSG000002	AARSD1	0.67476369	0.8743925	0.04525454
ENSG000001	NUDT2	0.69517239	0.88224216	0.0452622
ENSG000001	ADCY3	0.53593089	0.80423066	0.0452666
ENSG000001	ZNF672	0.35968709	0.69484359	0.04529297
ENSG000002	RUFY2	0.56538267	0.81924606	0.04531692
ENSG000001	MED13L	0.58707961	0.83027713	0.04535583

ENSG000001	MED9	0.48689302	0.77767904	0.0453667
ENSG000002	DDAH2	0.90667728	0.96840672	0.0453783
ENSG000002	SSTR3	0.72152945	0.89490971	0.0454
ENSG000001	NFU1	0.67746651	0.87510133	0.04546439
ENSG000001	MRFAP1L1	0.67573942	0.87451032	0.04548437
ENSG000001	ABCD1	0.67465909	0.8743925	0.04551946
ENSG000001	TRMT44	0.5158338	0.79496643	0.04555415
ENSG000001	ZNF691	0.37562796	0.70743725	0.04558661
ENSG000001	DPAGT1	0.4798947	0.77364477	0.04559118
ENSG00000C	OSBPL8	0.74637183	0.90662373	0.04568269
ENSG000001	GORASP2	0.34372557	0.68437426	0.04568564
ENSG000001	SGMS2	0.71072502	0.89043535	0.04570412
ENSG000001	EIF2S3	0.45862276	0.75816104	0.04574959
ENSG000001	ZDHHC9	0.62616656	0.85066154	0.04576233
ENSG000001	PPTC7	0.59853893	0.83673325	0.04576602
ENSG000001	MCM3AP	0.31445091	0.66265276	0.04578158
ENSG000001	ATRIP	0.56916308	0.82037292	0.04578602
ENSG000001	PITRM1	0.50499006	0.78773404	0.04579058
ENSG000001	AP3S1	0.46235987	0.76201149	0.04579691
ENSG000001	BCL2	0.68102077	0.87679749	0.04580185
ENSG000001	PROCR	0.6545983	0.86439091	0.04584555
ENSG000001	RTN1	0.4443431	0.74925205	0.04585031
ENSG000001	RAB34	0.51069058	0.79143652	0.04589548
ENSG000001	SFXN4	0.63759892	0.85616057	0.04592126
ENSG00000C	TXNL1	0.50783772	0.79065693	0.04592463
ENSG000001	VWA5A	0.37442167	0.70642398	0.04595891
ENSG000001	VPS53	0.19129609	0.55685897	0.04597858
ENSG000001	TTC3	0.64838188	0.86180228	0.04599348
ENSG000001	ZCCHC17	0.66413379	0.86837979	0.04599857
ENSG00000C	IARS2	0.3539904	0.69162813	0.04603979
ENSG000001	LYAR	0.56396527	0.81864675	0.04604046
ENSG000001	ENTPD4	0.37821225	0.70909881	0.0460629
ENSG000001	MARCHF6	0.54548076	0.80952117	0.04608151
ENSG000001	GPR25	0.79211886	0.92314956	0.04608576
ENSG000001	ZSCAN26	0.65324631	0.86393759	0.04609247
ENSG000001	MGME1	0.43859706	0.74601932	0.04612324
ENSG00000C	POLA2	0.49125682	0.78031845	0.04616031
ENSG000001	ZC3H12A	0.47302261	0.76859183	0.04622395
ENSG000001	ACTR2	0.46537416	0.76438119	0.04629543
ENSG000001	MRPL9	0.6226201	0.84922092	0.04634627
ENSG00000C	SARM1	0.57580692	0.82358853	0.04634774
ENSG000001	SREBF2	0.42357336	0.73732623	0.04635332
ENSG00000C	UBE2D4	0.54062656	0.80733448	0.0463721

ENSG000001	BPHL	0.7012944	0.88517701	0.04638287
ENSG000001	HOXC4	0.76448921	0.91243616	0.04644734
ENSG000001	BZW2	0.60313478	0.83873753	0.04646612
ENSG00000C	GEMIN5	0.43131701	0.74126857	0.04649547
ENSG000001	IL17RE	0.74769289	0.90727819	0.04652229
ENSG000001	GPR150	0.87951063	0.95817324	0.04655424
ENSG000001	SCHIP1	0.90574714	0.96827466	0.04655521
ENSG000001	LDHA	0.37799289	0.70909881	0.04658696
ENSG000001	EXOSC2	0.63547379	0.85504577	0.04661185
ENSG000001	NAP1L1	0.68975383	0.88074719	0.04662205
ENSG000001	CHN2	0.72700827	0.89708846	0.04664835
ENSG000001	ACTR5	0.55579946	0.81463404	0.0466813
ENSG000001	SLC25A42	0.67902351	0.87594571	0.04672153
ENSG000002	NBPF12	0.5238648	0.79870477	0.04673562
ENSG000001	RNF168	0.47155064	0.76694135	0.04678258
ENSG00000C	LAPTM4A	0.3483705	0.68768967	0.04682749
ENSG000001	PGRMC2	0.47796798	0.77199385	0.04684919
ENSG000001	FXYD6	0.63826823	0.8564206	0.04688464
ENSG000001	FBXW7	0.43278866	0.7425179	0.04689812
ENSG000001	CENPU	0.77882307	0.91810931	0.04694175
ENSG00000C	SLC39A9	0.20089342	0.56770499	0.04694711
ENSG000001	SMAP1	0.33031677	0.67392064	0.04697657
ENSG000001	RSL1D1	0.62554557	0.85038375	0.04698406
ENSG000001	SETDB1	0.03539221	0.32950881	0.04699039
ENSG000001	ANXA3	0.76524764	0.91260342	0.04700302
ENSG00000C	VPS13D	0.49015861	0.77995936	0.04701997
ENSG000001	TLE6	0.92519303	0.97521605	0.04702958
ENSG000001	DFFA	0.51774049	0.79566132	0.04707047
ENSG000001	GRIN2C	0.80436224	0.92881463	0.04712375
ENSG000001	NCL	0.3675162	0.69998364	0.0471273
ENSG000001	SLC35G1	0.73005219	0.89828814	0.04715682
ENSG000001	LPIN2	0.49485811	0.78124152	0.04718018
ENSG00000C	KDM7A	0.54171183	0.80806385	0.04718024
ENSG000001	CCDC112	0.67337871	0.87354948	0.04720042
ENSG000001	NCBP2L	0.91125464	0.97086887	0.04722178
ENSG00000C	AP2B1	0.1819385	0.54661139	0.04727753
ENSG000001	LRRC47	0.29255251	0.64955218	0.04729159
ENSG000001	NDUFB1	0.55366637	0.8139043	0.04731335
ENSG000001	EXOC4	0.30644551	0.65735608	0.04733434
ENSG000001	LRRK2	0.71221432	0.89093319	0.0473756
ENSG000001	PRPF38A	0.44331625	0.74894662	0.04737834
ENSG000001	ZNF418	0.72120154	0.89490971	0.04738668
ENSG000001	BSCL2	0.55991047	0.81699589	0.04740111

ENSG000001	S100PBP	0.545553	0.80955439	0.04742077
ENSG000001	PDCD1	0.9348609	0.97859224	0.04744168
ENSG000001	BCL7A	0.67445842	0.87427601	0.04745589
ENSG000001	SGF29	0.5640092	0.81864675	0.04748894
ENSG000001	SHC1	0.27514738	0.63499184	0.04749103
ENSG000001	FAM53A	0.75409818	0.90944172	0.04749388
ENSG000001	C1orf162	0.45253074	0.75423338	0.04749968
ENSG000001	UROC1	0.91871251	0.97272242	0.04751299
ENSG00000C	PDPR	0.3930651	0.72063539	0.04766023
ENSG000001	STK24	0.37233359	0.70436683	0.04770771
ENSG00000C	SCFD1	0.52477324	0.79901942	0.04770922
ENSG000001	C11orf21	0.43316564	0.74269383	0.04771708
ENSG000001	SKIDA1	0.91782338	0.97272242	0.04772065
ENSG000001	GALNT11	0.53700383	0.80503473	0.04774682
ENSG00000C	ADAT1	0.28219418	0.64012112	0.04775285
ENSG000001	MRPL54	0.56655354	0.8197023	0.04778755
ENSG000001	CTNNAL1	0.75858644	0.91110053	0.04782612
ENSG000001	CTNNBL1	0.41682773	0.73289419	0.04784089
ENSG000001	SAMSN1	0.60083175	0.83790304	0.04785668
ENSG000001	FCHSD2	0.24613161	0.6120929	0.04787679
ENSG000001	AAMP	0.31441885	0.66265276	0.04790077
ENSG000001	NCKAP1L	0.1654957	0.52885285	0.04790103
ENSG000001	RANBP3L	0.92524501	0.97521605	0.04790786
ENSG000002	N/A	0.81299409	0.93170624	0.04793541
ENSG000001	PMS2	0.47586477	0.77036688	0.04802736
ENSG000002	OR2AE1	0.88702017	0.96095764	0.04805039
ENSG000001	ZSWIM3	0.38341816	0.71225009	0.04807265
ENSG000002	INPP5B	0.42502761	0.73817459	0.048082
ENSG000001	AMOTL1	0.67072905	0.87216822	0.04809242
ENSG00000C	ING3	0.52747062	0.80062313	0.04814132
ENSG000001	NCAPD3	0.51095685	0.79164183	0.0481529
ENSG000001	CDKL1	0.52808633	0.80062313	0.04816398
ENSG000001	DIS3L2	0.48668114	0.77767904	0.04819517
ENSG000001	WDPCP	0.56363414	0.81863326	0.04819836
ENSG000001	RDH12	0.91955333	0.97307009	0.04824685
ENSG00000C	TINF2	0.20801243	0.57148862	0.0483038
ENSG000001	POF1B	0.88703441	0.96095764	0.04830654
ENSG000001	EWSR1	0.06609681	0.39345499	0.0483184
ENSG000001	SRSF5	0.45003169	0.75285025	0.04832377
ENSG000001	DENND6A	0.57451061	0.82332998	0.04834727
ENSG000001	ZSWIM6	0.52808762	0.80062313	0.04836394
ENSG000001	POLR3C	0.3239372	0.6699145	0.04840444
ENSG000001	SBF2	0.51851645	0.79577989	0.04841254

ENSG000001	DOLPP1	0.49060239	0.7800506	0.04845101
ENSG000001	AUH	0.55260642	0.81383402	0.04845712
ENSG000001	NME9	0.77198496	0.91507664	0.04847058
ENSG000001	MSTO1	0.52389951	0.79870477	0.04848283
ENSG000001	PXMP4	0.53249224	0.80280163	0.04851915
ENSG000001	SLC5A10	0.74613885	0.90653584	0.0485412
ENSG000002	KRTAP5-10	0.90625547	0.96827466	0.04857
ENSG000001	CAPN15	0.56648985	0.8197023	0.0485796
ENSG000001	NUP58	0.5227585	0.79827234	0.04857995
ENSG000001	ETV3	0.2807945	0.63927558	0.04862749
ENSG000001	ZNF623	0.87450351	0.95579655	0.04862912
ENSG000001	AFAP1L2	0.70351163	0.88639081	0.04866146
ENSG000002	HSBP1	0.49422358	0.78124152	0.0487106
ENSG000001	KHDRBS1	0.22879591	0.59716433	0.04874673
ENSG000001	WDR48	0.45344359	0.75477115	0.04880268
ENSG000001	PARP1	0.44942587	0.75245698	0.04882213
ENSG000002	PPME1	0.28760448	0.64495772	0.04882973
ENSG000001	OVOL1	0.90991074	0.97015546	0.04885116
ENSG000001	FBXO34	0.38332972	0.71225009	0.04889003
ENSG000001	TMPRSS6	0.81609843	0.93251163	0.04893767
ENSG00000(	SCMH1	0.56171579	0.81801019	0.04897103
ENSG00000(	CDV3	0.22418709	0.59160178	0.04898137
ENSG000001	INCA1	0.71112716	0.89059558	0.04899064
ENSG000001	BCL7B	0.35811931	0.69382497	0.0490144
ENSG000001	KLHL17	0.62337854	0.84950041	0.04908501
ENSG00000(	GPC1	0.75741658	0.91082077	0.04908591
ENSG00000(	RAD51	0.92507413	0.97521605	0.04909939
ENSG000001	C11orf80	0.62703017	0.85099522	0.04911447
ENSG000001	DOK7	0.78316855	0.91983554	0.04916938
ENSG00000(	ADCK1	0.54004614	0.80698777	0.04917364
ENSG000001	IRF2BP2	0.38385299	0.7123445	0.04918875
ENSG000001	ZNF304	0.6216011	0.84866582	0.04920371
ENSG000001	DAGLA	0.60334104	0.83883394	0.04921047
ENSG000001	STAT5A	0.48547028	0.77729819	0.04922606
ENSG000001	CEP104	0.42993709	0.74091639	0.04926764
ENSG000001	ZFYVE9	0.73068202	0.89859002	0.04930211
ENSG000002	LTB	0.89384855	0.9639421	0.04931842
ENSG000001	ATP5F1A	0.40055001	0.72594381	0.04933347
ENSG000001	COQ2	0.51048127	0.79143652	0.04937406
ENSG000001	H1-3	0.87608866	0.95676199	0.04940498
ENSG000001	DMWD	0.60045131	0.83766028	0.04941262
ENSG000001	GPRIN1	0.75031589	0.9080858	0.04941396
ENSG000001	MANBAL	0.23770477	0.60402516	0.04945666

ENSG000001	CISH	0.34220052	0.68310736	0.0494604
ENSG000001	SPIDR	0.4764813	0.77104115	0.04951252
ENSG000001	PDHB	0.38738914	0.71666498	0.04952739
ENSG000001	C11orf95	0.59340567	0.83456808	0.04953114
ENSG000001	PNCK	0.89320275	0.96361469	0.04955117
ENSG000001	TVP23A	0.63077642	0.85326916	0.04960242
ENSG00000(	PPIE	0.5718489	0.82170356	0.04960581
ENSG000001	PARPBP	0.82165275	0.93539963	0.04965425
ENSG000001	CHST2	0.54959593	0.81217374	0.04968306
ENSG000001	TTC9C	0.34482421	0.68522517	0.04975688
ENSG000001	ZKSCAN1	0.41453607	0.73184916	0.04976071
ENSG000002	ATXN1L	0.33042768	0.67401434	0.04981202
ENSG000001	GALNT1	0.54831987	0.81144134	0.04982692
ENSG000001	TRMT2B	0.41177824	0.73035548	0.04985516
ENSG000001	C7orf31	0.45689958	0.75721169	0.04988146
ENSG00000(	P2RY10	0.63383285	0.85463743	0.04989349
ENSG000001	CRK	0.53579039	0.80423066	0.04989736
ENSG000001	RAD54L2	0.44448756	0.74936605	0.04990474
ENSG000001	SON	0.12787799	0.48588488	0.04992969
ENSG000002	DDOST	0.31927828	0.66648568	0.04993125
ENSG000001	PAN2	0.48870421	0.77913481	0.04994564
ENSG000001	DYRK1A	0.33829619	0.67998455	0.04995674
ENSG000001	PAN3	0.53511145	0.80413676	0.04998403
ENSG000001	PLA2R1	0.90036731	0.96608561	0.05002394
ENSG00000(	CDC5L	0.33964009	0.68108387	0.05004502
ENSG000001	ZNF606	0.66146296	0.86736018	0.05012838
ENSG000001	MTOR	0.36499806	0.69795307	0.05014464
ENSG000001	NDUFAF2	0.77187207	0.91507664	0.0501465
ENSG000001	ARMC12	0.74524576	0.90628646	0.05015145
ENSG000001	DDB2	0.50382837	0.78675515	0.05018413
ENSG000001	SNRNP25	0.61954186	0.84711589	0.05019469
ENSG000001	RNF20	0.15575794	0.51796902	0.05020985
ENSG00000(	FAM107B	0.44693615	0.75099423	0.05029889
ENSG00000(	SEMA6A	0.64786466	0.86149962	0.05035522
ENSG000001	GPR176	0.92487202	0.97521605	0.05043218
ENSG000001	RAD50	0.63604881	0.85549278	0.05043231
ENSG00000(	NOP14	0.3617052	0.69588937	0.05044566
ENSG000001	HPGD	0.64794195	0.86149962	0.05048106
ENSG000001	SPDYE16	0.86474201	0.95224252	0.05051059
ENSG000001	TMEM14B	0.62711067	0.85099522	0.05053303
ENSG000001	EI24	0.48890894	0.77926911	0.05062763
ENSG000001	SLC17A9	0.63626692	0.85557482	0.05064595
ENSG000002	LYRM4	0.6932171	0.88183499	0.05068346

ENSG000001	CD19	0.56695078	0.8197023	0.05070927
ENSG000001	RMC1	0.0587085	0.38067583	0.05072374
ENSG00000(	REV3L	0.51063174	0.79143652	0.0507348
ENSG000001	KLF9	0.6619939	0.86736018	0.05074325
ENSG000002	CHKB-CPT1E	0.78866738	0.92204735	0.05076006
ENSG00000(	TOP2B	0.45021908	0.75290713	0.05076883
ENSG000001	BRME1	0.75214083	0.908573	0.05077842
ENSG000001	CAB39L	0.49932181	0.78408977	0.05081764
ENSG000001	L3MBTL2	0.40277759	0.72672584	0.05086379
ENSG000001	NDFIP1	0.47749804	0.77191738	0.05086691
ENSG000001	RRP7A	0.35331821	0.69126085	0.05093207
ENSG000001	TRNAU1AP	0.55291451	0.81383402	0.05094196
ENSG00000(	PLEKHA5	0.71498574	0.89175724	0.05099408
ENSG000001	MROH8	0.68421405	0.87892275	0.05099531
ENSG000001	IL18	0.5712276	0.82159192	0.05100776
ENSG000001	SRP14	0.55803533	0.81587881	0.05101015
ENSG000001	SYT11	0.45269578	0.75435359	0.05103571
ENSG000001	NOL4L	0.34950495	0.68878649	0.05105026
ENSG000001	PLAG1	0.77190335	0.91507664	0.05105411
ENSG000001	ZNF30	0.77218364	0.91511173	0.05110415
ENSG000002	PGAM4	0.7423782	0.90558693	0.05110652
ENSG000001	CLCF1	0.59584565	0.83576612	0.05110741
ENSG000002	TINCR	0.73000427	0.89828814	0.05110871
ENSG000001	NTNG2	0.75398556	0.90944172	0.05111468
ENSG000001	LRRC4C	0.89164073	0.96279647	0.05116127
ENSG000001	HSH2D	0.62438201	0.84979805	0.05116168
ENSG000001	SERPINB6	0.40612816	0.72748349	0.0511684
ENSG000001	RBM48	0.49586109	0.78183109	0.05121269
ENSG000001	FGFRL1	0.55819863	0.81594748	0.05123484
ENSG000001	XPNPEP2	0.67739633	0.87510133	0.05130878
ENSG000001	TBC1D9B	0.33584878	0.67749711	0.05133145
ENSG000001	MYL12A	0.40623349	0.72748349	0.051342
ENSG000001	DNAJC14	0.20270269	0.56815551	0.05140359
ENSG000001	MMP19	0.80469944	0.92889981	0.05151394
ENSG000001	PRKAR1A	0.56121739	0.81785019	0.05153335
ENSG000002	HEXA	0.41450502	0.73184916	0.0515413
ENSG00000(	TTC39A	0.62714841	0.85099522	0.05160332
ENSG00000(	MPC1	0.42465734	0.7376871	0.05160386
ENSG000001	GSTZ1	0.50167736	0.7853582	0.05164334
ENSG000001	GALNT6	0.41819771	0.73381176	0.05164371
ENSG000001	RAP2B	0.4694358	0.7662622	0.05165209
ENSG000001	NAAA	0.39732534	0.72359677	0.05165709
ENSG000002	NPIPB2	0.67624187	0.87463061	0.05166027

ENSG000001	DMAC1	0.72630675	0.89706038	0.05167443
ENSG000001	SLC25A16	0.50004033	0.78433741	0.05172142
ENSG000001	INTS14	0.46460243	0.76392983	0.05172458
ENSG000001	FAM81A	0.74531275	0.90628646	0.05174166
ENSG000001	SDHC	0.32428566	0.66992678	0.0517462
ENSG000001	DNAJC18	0.64130811	0.8575293	0.05179333
ENSG000002	RBMXL1	0.20684398	0.57036149	0.0518216
ENSG000001	KANSL3	0.28346483	0.64165879	0.05182182
ENSG000001	COQ6	0.60466359	0.83901251	0.05185584
ENSG000001	NPTN	0.3251491	0.6708073	0.05186009
ENSG000001	FAM117B	0.45639335	0.75682857	0.05186484
ENSG000001	ENSA	0.22484844	0.59200912	0.05189102
ENSG000001	TXNDC12	0.46686274	0.7650199	0.0519251
ENSG000001	TBP	0.40362522	0.72672584	0.05193041
ENSG000001	TBL1XR1	0.55991632	0.81699589	0.05194689
ENSG000001	RNMT	0.48624779	0.77752459	0.05196676
ENSG000001	CRLF3	0.39563876	0.72212079	0.05197064
ENSG000001	CSE1L	0.54246396	0.80815796	0.05203729
ENSG000001	GPBP1L1	0.15091743	0.51146865	0.05205023
ENSG000001	RIOK1	0.51681458	0.79538014	0.05209346
ENSG000001	TMEM218	0.58554019	0.82954508	0.05213217
ENSG000001	SNAPIN	0.31463033	0.66270362	0.05214699
ENSG000001	C3orf22	0.8979525	0.96556654	0.05220483
ENSG000001	SLC41A1	0.61314514	0.8450816	0.05221682
ENSG000001	SLC13A3	0.82599533	0.93591669	0.05222013
ENSG00000C	TMEM159	0.45940366	0.75906556	0.05222461
ENSG000001	PDE5A	0.56913306	0.82037292	0.05225408
ENSG000002	H2BC7	0.80742076	0.92973209	0.05225878
ENSG000001	WNT10A	0.72155013	0.89490971	0.05233172
ENSG000001	MAX	0.2109013	0.57523547	0.05234129
ENSG000001	CSGALNACT:	0.60186625	0.83845247	0.0523925
ENSG000001	SMIM19	0.67153756	0.87264949	0.05247464
ENSG000001	ANK3	0.67570969	0.87451032	0.05253685
ENSG000001	PIGH	0.59877361	0.83673325	0.05255488
ENSG000001	ELP6	0.6309743	0.85326916	0.05257304
ENSG000001	LTO1	0.48741906	0.77793335	0.05268732
ENSG000001	MED20	0.40091583	0.72606468	0.05270714
ENSG000001	MINDY2	0.59390214	0.83456808	0.05275446
ENSG000002	NOL7	0.38715895	0.7165258	0.05275838
ENSG000001	ZCRB1	0.6311484	0.8533351	0.05276031
ENSG000001	PNPO	0.44070522	0.74749092	0.05276427
ENSG000002	ERV3-1	0.51643907	0.79520352	0.05279993
ENSG00000C	SZRD1	0.14855046	0.50798213	0.05283785

ENSG000002	ZNF512	0.46898612	0.76616638	0.05288156
ENSG000001	RNF207	0.68763156	0.88051877	0.05290639
ENSG000001	AVPI1	0.66991273	0.87172407	0.05293908
ENSG000001	CCT3	0.428685	0.74045488	0.05295128
ENSG00000C	FTSJ1	0.39178763	0.71972665	0.05296637
ENSG000001	DGAT2L6	0.88735018	0.96110365	0.05297149
ENSG000001	ZNF750	0.88939353	0.96177707	0.05297384
ENSG000001	GPR183	0.71746601	0.89309227	0.05297656
ENSG000001	VANGL1	0.53048407	0.80199905	0.05297944
ENSG000001	DSE	0.49208386	0.7804495	0.05302361
ENSG000001	C1orf43	0.26692465	0.62929976	0.05302582
ENSG000001	GPR160	0.43388409	0.74337617	0.05305082
ENSG000002	SCYGR4	0.85176994	0.94720831	0.05306735
ENSG000001	CNBD2	0.91281671	0.97123107	0.05313288
ENSG000001	PXYLP1	0.67877115	0.87588072	0.05313334
ENSG000002	ZNF10	0.76275723	0.91167139	0.05317703
ENSG000001	EIF4E2	0.11893707	0.47566041	0.05318874
ENSG000001	EXOSC8	0.6450216	0.85991395	0.05320513
ENSG000001	LRRC59	0.29937126	0.6542516	0.05322858
ENSG000002	N/A	0.78935689	0.92253566	0.05324263
ENSG000002	LRRC73	0.85545367	0.9486844	0.05324583
ENSG000001	MIEF1	0.34793875	0.68740599	0.05326121
ENSG000001	CES3	0.89434229	0.96404358	0.05327129
ENSG000001	F2R	0.55995768	0.81699589	0.05332983
ENSG000001	TRIM33	0.18735553	0.55140326	0.05339584
ENSG000001	ATP5PF	0.51862178	0.79577989	0.0534195
ENSG000002	GET4	0.59236555	0.83392318	0.05344981
ENSG000001	MPP6	0.76214281	0.91151487	0.05346154
ENSG000001	ESYT2	0.42422255	0.73732623	0.05351594
ENSG000001	VIRMA	0.20211317	0.56777015	0.05354097
ENSG00000C	RPL6	0.69935062	0.88434908	0.05354913
ENSG000001	TXNDC11	0.31321189	0.6613866	0.05356009
ENSG000001	AKNAD1	0.89428976	0.96404358	0.0535677
ENSG000001	SNUPN	0.51484803	0.79420539	0.0535817
ENSG000001	METAP2	0.65545889	0.86448591	0.05363586
ENSG000001	SELENOW	0.52661101	0.80019714	0.05365492
ENSG000001	LUC7L3	0.61183495	0.84396943	0.05365535
ENSG000001	CITED2	0.21849343	0.58528507	0.05371827
ENSG000001	ZKSCAN5	0.27590892	0.63548653	0.05375227
ENSG000001	RGS9	0.71496152	0.89175724	0.05377438
ENSG000001	MRRF	0.51824385	0.79572786	0.05381657
ENSG000001	ERLIN2	0.38444997	0.71275568	0.053859
ENSG000001	VPS33A	0.25654135	0.62074989	0.05389116

ENSG000001	YEATS2	0.22022368	0.58773371	0.05390487
ENSG000002	ARID3C	0.89710853	0.96518862	0.05391572
ENSG000001	ZKSCAN7	0.62054568	0.84798858	0.05396611
ENSG000001	SPATA5L1	0.12067206	0.47724625	0.0539719
ENSG000001	HTT	0.35789334	0.69382497	0.0539934
ENSG000001	CFD	0.79147402	0.92308316	0.0540035
ENSG000001	CNOT8	0.3376085	0.67946062	0.05402254
ENSG000001	SOWAHC	0.69456342	0.8821445	0.05405211
ENSG000001	LSM4	0.44682252	0.75099423	0.05407639
ENSG00000(	DNAJC11	0.32897484	0.67287758	0.0540801
ENSG000001	EIF4G2	0.22348756	0.59081204	0.05415046
ENSG00000(	CBFA2T2	0.40804862	0.72783538	0.05416653
ENSG000001	CCDC107	0.60069344	0.83785409	0.0542095
ENSG000001	ALDH6A1	0.41417915	0.73145822	0.05422007
ENSG000002	HOMEZ	0.30354005	0.6561378	0.05430642
ENSG000001	COX5A	0.4964569	0.78193527	0.05432859
ENSG000001	TMED10	0.45242907	0.75419655	0.05432984
ENSG000001	MYD88	0.4654441	0.76438119	0.05434063
ENSG000001	FAF1	0.38090644	0.7101777	0.05435136
ENSG000001	TCP11	0.87942231	0.95817324	0.05438676
ENSG000001	CFAP157	0.70322283	0.88623325	0.05441005
ENSG000001	STX18	0.46144859	0.76122418	0.05442004
ENSG000001	TDRKH	0.5639686	0.81864675	0.0544345
ENSG00000(	NFYA	0.44525061	0.74940749	0.05444568
ENSG000002	C17orf49	0.65433436	0.86439091	0.05446968
ENSG00000(	CDK13	0.15122012	0.51206697	0.05447838
ENSG00000(	MTMR7	0.66605061	0.86976547	0.05450456
ENSG000001	ZNF823	0.6773865	0.87510133	0.05451444
ENSG00000(	TTC17	0.40876641	0.72855546	0.05453305
ENSG000001	MMRN1	0.64724589	0.86129024	0.05458044
ENSG000001	PAQR8	0.42119386	0.73521014	0.05458204
ENSG000001	UBQLN1	0.34355781	0.68429197	0.05461711
ENSG000001	MCC	0.49608729	0.78185081	0.05467506
ENSG000001	CPLANE2	0.65566621	0.86467033	0.05467746
ENSG000001	ACLY	0.2030754	0.56867381	0.05468361
ENSG000001	LUC7L2	0.47979911	0.77364477	0.05470326
ENSG000001	COL4A3	0.77860525	0.91810931	0.05470779
ENSG000001	CD33	0.42090541	0.7350227	0.05471113
ENSG000001	IMP3	0.43804032	0.74561911	0.05480406
ENSG000001	VPS35L	0.20528205	0.56904198	0.05481143
ENSG000001	EIF3M	0.56642074	0.8197023	0.05483302
ENSG000001	PSMC3	0.36108343	0.69535997	0.05486195
ENSG000001	CYC1	0.43587034	0.74462291	0.05487922

ENSG000001	MRPL58	0.61997609	0.84761181	0.0549028
ENSG000002	EBLN2	0.60031269	0.83755822	0.05493381
ENSG000001	PEX7	0.61638332	0.84579775	0.05500016
ENSG000001	CRELD2	0.39031452	0.71927554	0.05503275
ENSG000001	DHX57	0.28155342	0.63946601	0.05504952
ENSG000001	BBS2	0.52375829	0.79870477	0.05508005
ENSG000001	DDX19A	0.34063641	0.68148267	0.05510718
ENSG000001	UNK	0.32629718	0.67170284	0.05511848
ENSG000002	N/A	0.90532005	0.96827466	0.05513565
ENSG000001	DHX9	0.20337103	0.56874532	0.05517541
ENSG000001	SNX30	0.46513794	0.76426277	0.05521899
ENSG000001	TIAM1	0.5158209	0.79496643	0.05522067
ENSG000001	ZMYM2	0.57495923	0.82344878	0.0552252
ENSG000001	SEC16B	0.90548224	0.96827466	0.0552331
ENSG000001	CNOT1	0.26205511	0.62564164	0.05526089
ENSG000001	SLC46A3	0.43530585	0.74447662	0.05534299
ENSG000001	FBXO25	0.59449242	0.83494278	0.05538208
ENSG000002	RAB6C	0.89538058	0.96439765	0.05547126
ENSG000001	AKAP8	0.24867429	0.61459845	0.05551565
ENSG000001	FAM110B	0.5985994	0.83673325	0.0555295
ENSG000001	ARL4C	0.48760507	0.77796645	0.05554682
ENSG000001	OR4D1	0.66635364	0.86995128	0.05559242
ENSG000001	RPUSD4	0.52439896	0.79878989	0.05560415
ENSG000001	CSNK2A1	0.16924527	0.53349552	0.05562982
ENSG000002	ATP6V1FNB	0.62430195	0.84979805	0.05566338
ENSG000001	RAB39B	0.60403408	0.83883394	0.05566821
ENSG000001	EEF2K	0.49796118	0.78278591	0.05573348
ENSG000001	ABCC1	0.56478132	0.81898782	0.05575369
ENSG000001	UBAC2	0.09272898	0.43753271	0.05582038
ENSG000002	N/A	0.89448227	0.9640696	0.05587515
ENSG000001	TRIM68	0.3413118	0.68254277	0.05587537
ENSG000001	CEP70	0.76202814	0.91151487	0.05589795
ENSG000002	NUDT19	0.50069962	0.7851753	0.05591198
ENSG000001	CSNK1A1L	0.76415946	0.91243616	0.05594812
ENSG000001	SMCR8	0.35553331	0.69207883	0.05597244
ENSG000001	RAB5A	0.35072199	0.69032381	0.05601247
ENSG000001	CDCA7L	0.36492395	0.69791169	0.05605889
ENSG000001	DRG1	0.43598648	0.74463032	0.05609414
ENSG000001	RANBP2	0.43492212	0.74433605	0.05609607
ENSG000001	ZFP91	0.12061188	0.47724625	0.05611477
ENSG00000(	BCL2L13	0.1689772	0.53296205	0.05614061
ENSG000001	NDUFC2	0.55616939	0.81502909	0.05616594
ENSG000001	WDR20	0.26140926	0.6253871	0.05617346

ENSG000001	CIAO2B	0.43053898	0.74119757	0.05620586
ENSG000001	ACTR10	0.42436248	0.7374131	0.05621018
ENSG000001	ATP7A	0.54731522	0.81118038	0.05625583
ENSG000001	NOL6	0.31351206	0.66161883	0.05628039
ENSG000001	SLAIN2	0.32896053	0.67287758	0.05629476
ENSG000001	PAPSS1	0.27432977	0.63460874	0.05630416
ENSG000002	N/A	0.89534364	0.96439765	0.05631472
ENSG000001	SH2D5	0.88951771	0.96178325	0.05632691
ENSG000000	CDKL3	0.81201313	0.93168506	0.05635256
ENSG000001	FNDC11	0.69148317	0.88137846	0.05637884
ENSG000001	CSPG5	0.91153083	0.97090829	0.05638026
ENSG000001	LPCAT1	0.76439641	0.91243616	0.05643047
ENSG000001	NDUFV2	0.682765	0.87781049	0.05645114
ENSG000001	PPCS	0.31098699	0.65949478	0.0564675
ENSG000001	TMEM138	0.47546638	0.77011325	0.05647125
ENSG000001	ASNSD1	0.6503734	0.86310689	0.05647739
ENSG000002	N/A	0.90561858	0.96827466	0.05658194
ENSG000002	STRC	0.91201403	0.97118348	0.05658762
ENSG000001	BRIP1	0.80218587	0.92757225	0.0566196
ENSG000001	OR1K1	0.86931965	0.9539778	0.05662449
ENSG000001	GALNT15	0.89660207	0.96490939	0.05666761
ENSG000002	KIAA0040	0.5260377	0.80004441	0.0567155
ENSG000001	DEPDC4	0.87029813	0.95426978	0.05677388
ENSG000001	GSPT2	0.56159373	0.81801019	0.05678206
ENSG000001	ARL6IP1	0.43300874	0.74263121	0.05684894
ENSG000002	NPIPB11	0.60426562	0.83883394	0.05692738
ENSG000001	DDX39A	0.31131323	0.6598085	0.05692914
ENSG000001	PRORP	0.53877535	0.8064433	0.05694231
ENSG000001	AOAH	0.23382086	0.60167183	0.05697767
ENSG000000	EPB41L1	0.85642668	0.9490718	0.05704704
ENSG000001	PPIH	0.49443349	0.78124152	0.05706154
ENSG000000	MATK	0.60413841	0.83883394	0.05707758
ENSG000001	KIAA0930	0.31185955	0.66057537	0.0570825
ENSG000001	SEC22A	0.55114942	0.81317702	0.05709636
ENSG000001	GFI1	0.70072705	0.88498263	0.05711542
ENSG000001	TSPAN3	0.49452069	0.78124152	0.05712438
ENSG000001	ZDHHC2	0.48324618	0.77585394	0.05713021
ENSG000001	MAPRE1	0.03808886	0.33602196	0.05713668
ENSG000001	CDH13	0.59878856	0.83673325	0.05714538
ENSG000001	PDXDC1	0.78661205	0.92119044	0.05714685
ENSG000000	ZNF264	0.30787114	0.65762361	0.0571493
ENSG000001	MAP3K7CL	0.49371279	0.78107975	0.05717373
ENSG000000	PLEKHO1	0.40320787	0.72672584	0.05727514

ENSG000001	FAM114A1	0.48216728	0.77493168	0.05744078
ENSG00000C	SART3	0.1429372	0.5010464	0.05745252
ENSG000001	C14orf93	0.35223288	0.69082603	0.05745404
ENSG000001	IER5	0.34986386	0.68924835	0.05747224
ENSG00000C	INPP4A	0.42131916	0.7353498	0.05748685
ENSG00000C	DCUN1D1	0.56565191	0.81931223	0.05751919
ENSG000001	ALG8	0.47751551	0.77191738	0.05756154
ENSG000001	PTGER2	0.48417546	0.77655439	0.05763217
ENSG000001	ECD	0.15828766	0.52089921	0.05763272
ENSG000002	N/A	0.82805797	0.93660278	0.05763657
ENSG000001	RGS18	0.67499825	0.87446425	0.05764707
ENSG000001	ASPHD1	0.90587837	0.96827466	0.05767286
ENSG000001	RPA3	0.53267346	0.80280163	0.05768983
ENSG00000C	ICAM1	0.6397021	0.85691647	0.0576961
ENSG000001	APTX	0.48766044	0.77796645	0.05770447
ENSG000001	BLOC1S2	0.58409131	0.82875175	0.0577389
ENSG000001	LSM6	0.52839056	0.80062313	0.05774173
ENSG000001	PPA2	0.55737542	0.81587881	0.05774568
ENSG000001	PROX2	0.68177676	0.8771879	0.05777355
ENSG000001	TLK2	0.17625686	0.54121794	0.05777816
ENSG000001	ZNF382	0.71162747	0.89073961	0.05782455
ENSG000001	NARS1	0.4002881	0.72583241	0.05784636
ENSG000001	EMC7	0.30072382	0.65493729	0.05787326
ENSG000001	CENPV	0.6614336	0.86736018	0.05789406
ENSG000001	BMS1	0.37054386	0.70312117	0.05793539
ENSG000001	HMGXB3	0.30886573	0.65857555	0.058041
ENSG000002	CEBPZOS	0.53338729	0.80312903	0.05804574
ENSG000001	LSM5	0.65380802	0.86420392	0.05805442
ENSG000001	ANKRD36	0.71513032	0.89175724	0.05807765
ENSG000001	TCEAL1	0.7476466	0.90727819	0.05811288
ENSG000001	TOMM20	0.56700391	0.8197023	0.05812608
ENSG000002	CRYGS	0.56880006	0.82037292	0.05816562
ENSG000001	GABPB2	0.52426838	0.79877505	0.05817942
ENSG000001	SPRING1	0.30800013	0.65762361	0.05821302
ENSG000001	LEAP2	0.59439479	0.83487788	0.05823188
ENSG000001	CA5B	0.58886734	0.83142947	0.05824199
ENSG000001	ATG101	0.5094651	0.79136944	0.0582551
ENSG00000C	BAZ2A	0.45208215	0.75395007	0.05826194
ENSG000001	DISP2	0.69685176	0.88285035	0.05827173
ENSG000001	ISCA2	0.47163411	0.76694135	0.0582923
ENSG000001	DCTN4	0.19781418	0.56540555	0.05829485
ENSG000001	RESF1	0.57339901	0.82246288	0.05831563
ENSG000001	SMUG1	0.24095325	0.60721888	0.05832553

ENSG000001	SMU1	0.16352367	0.52709285	0.05833958
ENSG000001	ZBTB34	0.54673233	0.81063154	0.05834639
ENSG000001	RAB4A	0.49557274	0.78160414	0.05836333
ENSG000001	RAB24	0.52652571	0.80019714	0.05842083
ENSG00000C	GCLM	0.3908505	0.71964297	0.05845306
ENSG000002	VDAC1	0.30731488	0.65762361	0.05845501
ENSG00000C	PRKCH	0.52050259	0.79709497	0.05846557
ENSG000001	SMAD4	0.24359916	0.60937379	0.05848375
ENSG000001	SAMD15	0.8730512	0.95504496	0.05854104
ENSG000001	SVEP1	0.87641718	0.95679296	0.05857983
ENSG000001	SVBP	0.48013159	0.77383118	0.0585816
ENSG000001	TMEM31	0.90725765	0.96868131	0.05858408
ENSG000002	CYB5RL	0.62508334	0.85017942	0.0586225
ENSG00000C	ALG9	0.67317492	0.87354948	0.05862847
ENSG000001	ASH2L	0.06488553	0.3907813	0.05870318
ENSG00000C	ERGIC2	0.61374754	0.8451435	0.05879925
ENSG00000C	PTGER3	0.85838901	0.9497652	0.05881768
ENSG000001	RBMS1	0.11056423	0.46328552	0.05883808
ENSG000001	SLC7A1	0.50360957	0.78673932	0.05884662
ENSG000001	EPB41L4A	0.65086336	0.86333374	0.05885362
ENSG000001	UQCRC2	0.44317461	0.74887084	0.05890376
ENSG000001	LRIF1	0.43548909	0.74461931	0.05895628
ENSG000001	KLHL7	0.50411841	0.78690354	0.05900965
ENSG000001	PRPF38B	0.51721463	0.79538014	0.05900972
ENSG000001	RNF125	0.578523	0.8253649	0.05903311
ENSG000001	RIOK3	0.37719653	0.70869476	0.05909627
ENSG000001	MRPL14	0.55173636	0.81323014	0.05910913
ENSG000001	SMG8	0.13044234	0.48854664	0.05913051
ENSG000001	TMED8	0.42308949	0.73695551	0.05913093
ENSG000001	SLC39A1	0.66898528	0.87125589	0.05913801
ENSG000002	PRR29	0.45912252	0.75867823	0.05914898
ENSG000001	LETM1	0.31513358	0.6630587	0.0592369
ENSG000001	SSBP1	0.63302623	0.85440485	0.05926723
ENSG000001	QDPR	0.41668564	0.73285581	0.0593254
ENSG000001	RBM18	0.28442775	0.64253645	0.05934301
ENSG00000C	RUNX3	0.44437379	0.74925205	0.05935088
ENSG000002	N/A	0.53762743	0.80547376	0.0594531
ENSG000001	CCT5	0.15970935	0.52219103	0.0594645
ENSG000001	MARCHF7	0.49600918	0.78185081	0.05946915
ENSG00000C	COX10	0.37140062	0.70374977	0.05949002
ENSG000001	HECA	0.42070041	0.7349809	0.05953294
ENSG00000C	PIK3C3	0.32655409	0.67185873	0.05955308
ENSG000001	FOXK1	0.45232188	0.7541175	0.05955606

ENSG000001	HFM1	0.8595143	0.95048244	0.05955744
ENSG00000(	JKAMP	0.41700493	0.73294101	0.05956205
ENSG000001	MSH6	0.21451769	0.58023513	0.05961294
ENSG000001	BTRC	0.35462762	0.69190544	0.05962528
ENSG000001	AP1G1	0.10118489	0.44925869	0.05963834
ENSG000001	LYPLAL1	0.62383431	0.84972866	0.05968546
ENSG000001	GOT1	0.39821928	0.72429687	0.05972503
ENSG000001	SLAMF6	0.53744862	0.80547376	0.05972601
ENSG000001	ST3GAL3	0.57894814	0.82560872	0.05976376
ENSG000001	BCL2L2	0.30722103	0.65762361	0.05979605
ENSG000001	SUMO2	0.41841806	0.73381176	0.05979935
ENSG000001	PSMG1	0.63372061	0.85461553	0.05981736
ENSG000001	CPA5	0.83941057	0.94217303	0.05982302
ENSG000002	ZNF585B	0.51720256	0.79538014	0.05990344
ENSG00000(	ULK2	0.49216638	0.7804495	0.05991103
ENSG000002	C17orf100	0.64609239	0.86044964	0.05991287
ENSG00000(	ROCK1	0.51621135	0.79515933	0.05993513
ENSG000001	SLC2A8	0.58460846	0.82887597	0.05994599
ENSG00000(	PSMD5	0.34353089	0.68429197	0.05995849
ENSG00000(	MCUR1	0.39972106	0.72527731	0.06008131
ENSG000001	DHRS4	0.41410891	0.73141372	0.06009585
ENSG000001	WDR35	0.57591966	0.82367722	0.06010312
ENSG000001	TRIM32	0.52098231	0.79745258	0.06014327
ENSG000001	PPARA	0.39119539	0.71964297	0.06022889
ENSG000001	LANCL3	0.60120858	0.83799671	0.06024362
ENSG000001	UBASH3B	0.28084924	0.63927558	0.06025347
ENSG000001	S100Z	0.40445372	0.72672584	0.06025932
ENSG000002	RPL17	0.5718575	0.82170356	0.06032259
ENSG00000(	ITGA6	0.55414002	0.81405705	0.06032484
ENSG000002	GALNT4	0.82309691	0.93539963	0.06033206
ENSG000001	USP44	0.78955772	0.92261881	0.06035783
ENSG000001	LRR1	0.52050994	0.79709497	0.06038607
ENSG000001	PPFIA3	0.6924976	0.88158966	0.06043442
ENSG000001	BEND3	0.54899973	0.81177567	0.06043899
ENSG000001	ZNF202	0.42277961	0.73663877	0.06044685
ENSG000001	DPH2	0.42211351	0.73607851	0.06050291
ENSG00000(	ARFGEF1	0.32881052	0.67284741	0.06052456
ENSG000001	PPP2CA	0.15441528	0.51582229	0.06053157
ENSG000001	CMAS	0.42422066	0.73732623	0.06053521
ENSG00000(	MKS1	0.49995472	0.78433741	0.06054257
ENSG000001	PDCD2L	0.65937489	0.86611363	0.06060456
ENSG000001	RBBP7	0.33446163	0.67664065	0.06062268
ENSG000001	PPIP5K2	0.63288964	0.85440485	0.06064191

ENSG000001	SECISBP2	0.19455852	0.56193152	0.06068244
ENSG000001	EIF3A	0.29184397	0.64936748	0.06071532
ENSG000001	BAG5	0.2825679	0.64061085	0.06075184
ENSG000001	DTYMK	0.50923425	0.79136944	0.06082664
ENSG000001	PWWP3A	0.48579963	0.7774422	0.06085713
ENSG000001	MRPL17	0.54966498	0.81217374	0.06088264
ENSG000002	MILR1	0.41841765	0.73381176	0.06091195
ENSG000001	NAE1	0.66669997	0.87009558	0.06094666
ENSG000001	RBM19	0.38307044	0.71198657	0.06095522
ENSG000001	ARL4A	0.59820678	0.83653786	0.06096657
ENSG000001	EMG1	0.59071318	0.83266712	0.06099658
ENSG000002	TMEM141	0.4605766	0.76015269	0.06103296
ENSG000001	PMEPA1	0.47453914	0.76942167	0.06104412
ENSG000001	HK2	0.46749922	0.76548028	0.06107432
ENSG000001	RRS1	0.30463248	0.65635638	0.06107627
ENSG000001	MORN1	0.58412618	0.82875175	0.06108128
ENSG000001	LDAH	0.54767093	0.81121445	0.06110209
ENSG000001	TARS3	0.52382275	0.79870477	0.06113128
ENSG000001	RABGGTA	0.40522133	0.72686463	0.061156
ENSG000000	ATRX	0.21732912	0.58394389	0.06120559
ENSG000002	ARPIN	0.55499344	0.81433077	0.06121748
ENSG000001	CSF1	0.52936791	0.80120681	0.06122375
ENSG000001	NFATC2IP	0.32904855	0.67287758	0.06122967
ENSG000001	RBM28	0.39292171	0.72063539	0.06123529
ENSG000001	TCP11L1	0.52825396	0.80062313	0.06123777
ENSG000001	RPL15	0.60263892	0.8385858	0.06124661
ENSG000001	TNFRSF10D	0.48780183	0.77811557	0.06125211
ENSG000001	CWC22	0.4240656	0.73732623	0.0612904
ENSG000001	MED1	0.28159715	0.63946601	0.061291
ENSG000001	KLRG1	0.67077371	0.87216822	0.06130058
ENSG000001	DAAM1	0.430007	0.74091639	0.06130908
ENSG000001	MBTPS1	0.37087037	0.70329686	0.06130951
ENSG000001	NEK8	0.41251621	0.73063968	0.06133777
ENSG000001	HRK	0.74744851	0.90725285	0.06133831
ENSG000001	ANGPTL3	0.86181667	0.95102061	0.06135906
ENSG000001	ZBTB44	0.39806901	0.72426093	0.06139459
ENSG000001	ST8SIA4	0.46534728	0.76438119	0.06142017
ENSG000001	SLC7A6	0.56365109	0.81863326	0.06143228
ENSG000001	SIMC1	0.27464754	0.63464829	0.06143523
ENSG000001	AEN	0.40742256	0.72768322	0.06143888
ENSG000001	C16orf91	0.49074362	0.7800506	0.06144983
ENSG000000	CDON	0.84513612	0.94421922	0.06145131
ENSG000001	PDIA4	0.35839849	0.69382497	0.06145675

ENSG000001	TAPBPL	0.28720936	0.6446528	0.06145909
ENSG00000(	NDUFS1	0.1130098	0.46808189	0.06145925
ENSG000001	NLRC3	0.58004638	0.82610777	0.06146779
ENSG000001	CNOT6	0.49743129	0.78267582	0.06147756
ENSG000001	INO80	0.0971497	0.44321992	0.06147895
ENSG000001	GTF2H2	0.79236616	0.92314956	0.0614988
ENSG000001	TRAPPC2	0.36162981	0.69588937	0.06157843
ENSG000001	TNKS	0.24107138	0.60721888	0.06158697
ENSG000002	TMX2	0.34722734	0.68654778	0.06162373
ENSG000001	RGMA	0.87448847	0.95579655	0.0616472
ENSG00000(	EEF1AKNMT	0.20060365	0.56730451	0.06166916
ENSG000001	FOXJ3	0.2649188	0.6274921	0.06166945
ENSG000001	ARIH1	0.11605617	0.47160917	0.06169445
ENSG000001	TXNDC15	0.38839517	0.71712216	0.06174364
ENSG000001	RBM17	0.37487704	0.70718635	0.06176309
ENSG000001	DUT	0.55801527	0.81587881	0.0617638
ENSG000001	SERP1	0.37946079	0.70965485	0.0617708
ENSG00000(	DNTTIP2	0.58779952	0.83057183	0.06178616
ENSG000001	REC8	0.55820315	0.81594748	0.06179941
ENSG000001	COMMD3	0.61389175	0.8451435	0.06180116
ENSG000002	PEG10	0.77057928	0.91434491	0.06182513
ENSG000001	GGCX	0.24835688	0.61459845	0.06183936
ENSG000001	PIP5K1B	0.40416652	0.72672584	0.06191336
ENSG000001	MRAS	0.57874555	0.82553733	0.06191785
ENSG000001	SNX9	0.32205369	0.66822528	0.06193579
ENSG00000(	NUP133	0.2363188	0.60336718	0.06195495
ENSG000001	EIF2S2	0.40450103	0.72672584	0.06197412
ENSG000001	PEPD	0.3619698	0.69592458	0.06197641
ENSG000001	POLR3K	0.52475219	0.79901942	0.06201132
ENSG000001	TBL2	0.25625253	0.62056088	0.06207564
ENSG000002	CD300LD	0.87208543	0.95459934	0.06209799
ENSG000001	APOBEC3A	0.62202244	0.84900625	0.06214368
ENSG00000(	PSMB1	0.46124916	0.76110798	0.06217688
ENSG000001	ZC3H6	0.54403468	0.80885368	0.06218558
ENSG000001	EMSY	0.30855417	0.65826962	0.06220009
ENSG000001	ZNF607	0.66503137	0.86898991	0.06220827
ENSG000001	OCRL	0.43652572	0.74496191	0.06221741
ENSG000001	DYNC1I1	0.86136495	0.95080722	0.06222609
ENSG000001	TP53	0.27299923	0.63378205	0.06222775
ENSG00000(	PDE4A	0.48660209	0.77765336	0.0622325
ENSG000001	TSPYL1	0.31741409	0.66472853	0.06223883
ENSG000001	CIART	0.85411511	0.94814417	0.06235022
ENSG000001	CWF19L2	0.50933512	0.79136944	0.06236251

ENSG000002	CNEP1R1	0.47264037	0.76819436	0.06241616
ENSG000001	TBRG1	0.38821737	0.71704113	0.0624551
ENSG000002	GRID2IP	0.64043096	0.85706063	0.06249044
ENSG000001	SENP5	0.31293566	0.66126189	0.06253126
ENSG00000C	KMT2C	0.52124828	0.79758332	0.06255157
ENSG00000C	VPS9D1	0.63633838	0.85560005	0.06257772
ENSG00000C	ZW10	0.27334096	0.6339378	0.06258035
ENSG000001	GOLM2	0.43082982	0.74126857	0.06258711
ENSG000001	TMED9	0.2167214	0.58337291	0.06259028
ENSG000002	DYNLT2B	0.75515302	0.91013398	0.06259105
ENSG00000C	RAB18	0.5755273	0.82346952	0.06260728
ENSG000001	GORASP1	0.40840993	0.72824971	0.06261037
ENSG000001	LONP2	0.42962798	0.74091639	0.06261589
ENSG00000C	POLR2B	0.33775079	0.67946062	0.06267386
ENSG000002	NBPF1	0.66596445	0.86972289	0.06268565
ENSG000001	LIMS1	0.29099761	0.64869008	0.06269842
ENSG000001	USP8	0.11142383	0.46472866	0.06280827
ENSG000001	DGKE	0.65288047	0.86379938	0.06281067
ENSG000001	SLC39A8	0.57598598	0.82368641	0.06286826
ENSG000002	NBPF26	0.42820079	0.73996341	0.06288702
ENSG000001	MTERF4	0.59184881	0.8336094	0.06288832
ENSG000002	TMEM167B	0.34649642	0.68614821	0.06289487
ENSG00000C	RABGAP1	0.14184007	0.50055578	0.06296533
ENSG000001	TMEM106C	0.60163725	0.83830636	0.06307562
ENSG00000C	ZFYVE16	0.60288098	0.83860006	0.06313304
ENSG000001	SEPTIN6	0.38522691	0.71378858	0.06313461
ENSG000001	LNPK	0.50810049	0.79067965	0.06315657
ENSG000001	CFAP36	0.64744627	0.86140487	0.06322711
ENSG000001	DPH3	0.39104368	0.71964297	0.06322885
ENSG00000C	ADAM11	0.84448631	0.94402232	0.06329764
ENSG000001	NEK3	0.46585086	0.76466038	0.06333854
ENSG00000C	MFSD11	0.25848363	0.6223087	0.06335152
ENSG000001	MED6	0.2036182	0.56880022	0.06338899
ENSG000001	CRYBG1	0.06716098	0.39561184	0.06341776
ENSG000001	MRPS7	0.39535312	0.72209218	0.06345496
ENSG000001	DNAH2	0.87988748	0.95827424	0.06351068
ENSG00000C	FAM136A	0.56223777	0.81808896	0.06351644
ENSG000001	FAM151B	0.52171229	0.79764995	0.0635388
ENSG000002	C4orf46	0.54491206	0.80952117	0.06354928
ENSG00000C	CBX5	0.50330305	0.78656158	0.06360519
ENSG00000C	DNAJC25	0.55776933	0.81587881	0.06364438
ENSG000001	RABGAP1L	0.43576046	0.74462291	0.0636512
ENSG00000C	AARS1	0.35964839	0.69484359	0.06369706

ENSG000001	ATF7	0.05852932	0.3800398	0.06371685
ENSG000001	MYH3	0.36431223	0.69739047	0.06372784
ENSG000002	RPL36A	0.53143514	0.80232066	0.06373872
ENSG000001	RCAN1	0.41005961	0.72943277	0.06378521
ENSG00000C	GRIPAP1	0.21966804	0.58681553	0.0638104
ENSG000001	CRTAM	0.6682073	0.87090078	0.06383991
ENSG00000C	OSM	0.67610291	0.87463061	0.06385987
ENSG000001	PRPF3	0.24091807	0.60721888	0.0638607
ENSG000001	EEF1A1	0.66049567	0.86700658	0.0638985
ENSG000002	DMRTC1	0.88874551	0.96164016	0.06390118
ENSG00000C	HPF1	0.54741629	0.81118038	0.06390835
ENSG000001	ZNF410	0.57947562	0.82607074	0.0639651
ENSG000002	JRK	0.37819692	0.70909881	0.06396728
ENSG000001	IWS1	0.08759479	0.4299506	0.0640283
ENSG000001	HDAC2	0.37929585	0.70965485	0.06405114
ENSG000002	SMIM20	0.63140419	0.85334691	0.06416791
ENSG000001	CDIN1	0.57162987	0.82166733	0.06418493
ENSG000001	TMEM65	0.51267455	0.7923374	0.06424394
ENSG000001	NECAP2	0.11355973	0.46882009	0.06426013
ENSG000001	SOCS2	0.54023721	0.80712474	0.06428666
ENSG00000C	NUCKS1	0.48027061	0.77383118	0.06434606
ENSG000001	HCFC1	0.40570398	0.72730417	0.06435356
ENSG00000C	MPPED2	0.81678241	0.93280078	0.06437399
ENSG000001	PPP1R21	0.16806132	0.53240547	0.0643943
ENSG00000C	MCCC1	0.37678291	0.70849151	0.06440799
ENSG000001	TCEAL4	0.52265218	0.79818504	0.06444377
ENSG000002	MYZAP	0.77217098	0.91511173	0.06445149
ENSG000001	DOCK8	0.44799472	0.75114004	0.06446072
ENSG000001	KHDC4	0.34685159	0.68647359	0.06452928
ENSG00000C	PUM2	0.43875118	0.7460934	0.06457234
ENSG000001	PMFBP1	0.69272189	0.88158966	0.0646337
ENSG000001	C17orf80	0.37423761	0.70633918	0.06465337
ENSG000001	PCK2	0.30288968	0.65591444	0.06473255
ENSG000001	APLN	0.88970478	0.96183364	0.06474636
ENSG000001	CREB1	0.43583698	0.74462291	0.06475803
ENSG00000C	RAB11FIP3	0.68805171	0.88051877	0.06477043
ENSG000001	ROBO4	0.8975205	0.96522986	0.06477612
ENSG000002	N/A	0.35648005	0.69246335	0.06478677
ENSG000001	OGFOD3	0.39029576	0.71927554	0.06483179
ENSG000002	SCAF8	0.18439964	0.54968745	0.06484789
ENSG000001	NOL9	0.41608503	0.73235476	0.06486136
ENSG000001	PLEKHF1	0.51027964	0.79143652	0.0648947
ENSG000001	RAD17	0.77505624	0.916381	0.06491406

ENSG000001	MAPRE2	0.15260293	0.51346327	0.06495613
ENSG000001	UCN2	0.84891307	0.94594163	0.06500881
ENSG000001	CBR4	0.58636888	0.82959975	0.06501953
ENSG000001	SLC26A7	0.88132007	0.95917917	0.06502077
ENSG000001	UNC45B	0.75140897	0.908573	0.06502692
ENSG000001	DCAF10	0.11942076	0.47604098	0.06504876
ENSG000001	BAG2	0.72377826	0.89594966	0.06511933
ENSG000001	ELP3	0.20727555	0.57120384	0.06514067
ENSG000002	HCAR3	0.58979056	0.83191276	0.06516736
ENSG000002	TMEM273	0.4286799	0.74045488	0.06521149
ENSG000001	PSMD12	0.241208	0.60721888	0.06522037
ENSG000002	PDCD6	0.26911515	0.63099817	0.06528004
ENSG000001	AVL9	0.32470971	0.67035112	0.06533804
ENSG000001	EPHA10	0.88057245	0.95862252	0.06541096
ENSG000001	C11orf54	0.40345074	0.72672584	0.06542572
ENSG000001	LILRA5	0.69422032	0.8821445	0.0654431
ENSG000001	NAGS	0.44685227	0.75099423	0.06544535
ENSG000001	PFDN1	0.29467706	0.65098879	0.06558026
ENSG000001	ZNF773	0.46169463	0.76122418	0.06558678
ENSG000001	ZNF276	0.25708782	0.62086214	0.06561126
ENSG000001	YARS1	0.2928381	0.64967561	0.06561363
ENSG000001	VTI1A	0.15799096	0.52077869	0.06566755
ENSG000001	ENOSF1	0.61848806	0.84697252	0.06574127
ENSG00000C	PHF7	0.50439493	0.78710817	0.06577
ENSG000002	KRTAP10-11	0.76782527	0.91357827	0.06581539
ENSG000001	CEP128	0.54879381	0.81161901	0.06582067
ENSG000001	AKAP13	0.30177704	0.65561042	0.06582259
ENSG000001	ORMDL2	0.25109329	0.61695463	0.06582746
ENSG000001	LANCL2	0.27049715	0.63178344	0.06587327
ENSG000001	ZNF135	0.6085464	0.84186486	0.06594685
ENSG000001	CIPC	0.45357611	0.75489088	0.0659693
ENSG000001	LRFN4	0.53924698	0.80668447	0.06605191
ENSG000001	FBXO21	0.33431893	0.67664065	0.06611656
ENSG000001	PHB	0.45215624	0.75399619	0.0661469
ENSG000001	PITPNB	0.18213271	0.54662721	0.06617398
ENSG000001	TRARG1	0.84196797	0.94289495	0.06620311
ENSG000001	SPATA12	0.89845738	0.96566178	0.06621145
ENSG000001	ARPP19	0.27627571	0.6360577	0.06624015
ENSG000001	HMGN3	0.54650255	0.81061139	0.06624629
ENSG000001	ILRUN	0.0940411	0.43929464	0.0662541
ENSG000002	TMEM242	0.49702148	0.78237677	0.06626432
ENSG000001	ICAM5	0.74451772	0.90628646	0.06626567
ENSG000001	THBS2	0.63917492	0.85691647	0.06632616

ENSG000001	CPVL	0.27549033	0.63512113	0.06637324
ENSG000001	PLEKHB2	0.05297643	0.36981204	0.06638831
ENSG00000C	UBR7	0.84786908	0.94533065	0.06639196
ENSG000002	PRICKLE4	0.79030697	0.92280247	0.06641847
ENSG00000C	RWDD2A	0.58068958	0.82662376	0.06642924
ENSG000001	SDF2L1	0.48872871	0.77913481	0.06643084
ENSG000001	CNKSR1	0.62109902	0.8484591	0.0664332
ENSG000001	CCDC126	0.53055612	0.80203331	0.06646619
ENSG000002	C11orf98	0.8799415	0.95827424	0.06650912
ENSG000001	CEP350	0.34482239	0.68522517	0.06658332
ENSG000001	GALK2	0.37424203	0.70633918	0.0666035
ENSG000001	PIK3C2B	0.4250289	0.73817459	0.06660761
ENSG00000C	RB1CC1	0.5548761	0.81433077	0.06663508
ENSG000001	TRIM72	0.84766264	0.94527257	0.06672131
ENSG000001	NXPE3	0.39012263	0.71927554	0.06674016
ENSG000001	ILF2	0.37831251	0.70915034	0.06675824
ENSG000001	SAP18	0.04168156	0.34831883	0.06678545
ENSG000001	ABHD15	0.29623297	0.65199276	0.06679073
ENSG000001	MFAP1	0.1355327	0.49497175	0.06680736
ENSG000001	PIGO	0.28174504	0.63946601	0.06681078
ENSG000001	TP53BP2	0.1953159	0.56280385	0.06681731
ENSG000001	SLC22A5	0.55330395	0.81385767	0.06682415
ENSG000001	GCSAM	0.61527276	0.84533626	0.06686392
ENSG000001	CDCA4	0.46363013	0.7632528	0.06686705
ENSG000001	TBC1D30	0.41762172	0.73321581	0.06687427
ENSG000001	RBM33	0.49002093	0.77995936	0.06693187
ENSG000001	PRDM15	0.48488574	0.7769018	0.06697583
ENSG000001	U2AF1	0.78679315	0.92122734	0.06699403
ENSG000001	DISP1	0.48634714	0.77752459	0.06699722
ENSG00000C	FKBP4	0.39522182	0.72209218	0.06701653
ENSG00000C	PDCD7	0.4044135	0.72672584	0.06704511
ENSG000001	SLX4IP	0.39901211	0.72496091	0.06710159
ENSG000001	TTK	0.77533274	0.91656918	0.06720041
ENSG000001	TMEM217	0.8625479	0.951353	0.06721636
ENSG00000C	IPO5	0.41606406	0.73235476	0.06730367
ENSG000001	DNAJC16	0.40292543	0.72672584	0.06730706
ENSG000001	KLHDC10	0.23180604	0.599371	0.06733948
ENSG000001	UQCRB	0.67300873	0.87354948	0.067342
ENSG00000C	CPNE3	0.19159035	0.55704454	0.06734855
ENSG000001	MAD2L2	0.27303723	0.63378205	0.06736055
ENSG000001	WASHC5	0.06426573	0.39043354	0.06737352
ENSG000001	FBXO41	0.39261523	0.72047325	0.06740475
ENSG00000C	ARID1B	0.20354022	0.56880022	0.06741077

ENSG000002	N/A	0.67813435	0.87547646	0.06742094
ENSG000001	APC	0.4752845	0.77002538	0.06742885
ENSG000001	CANX	0.17026999	0.53437545	0.06744741
ENSG000001	CUL4A	0.20445271	0.5688754	0.06745068
ENSG000001	RBM15	0.36344975	0.69694698	0.06746959
ENSG000001	DDI2	0.24171446	0.60725942	0.06748961
ENSG000001	RTCA	0.40981179	0.72922889	0.0674909
ENSG000001	SELENOS	0.33928922	0.68063246	0.06752647
ENSG000001	HBEGF	0.62173187	0.84875234	0.06752855
ENSG000001	JAKMIP1	0.74673906	0.90679845	0.06754202
ENSG000001	NAT1	0.61046569	0.84315511	0.06756186
ENSG000001	SLC35B1	0.28146856	0.63945781	0.06758833
ENSG00000C	AIFM2	0.48049689	0.77383118	0.06759096
ENSG000002	ATXN7L3B	0.25460527	0.61970106	0.06761856
ENSG000001	LACTB2	0.5203898	0.79709497	0.06762941
ENSG000001	MRPL27	0.49946798	0.78410505	0.06764103
ENSG000001	SFMBT1	0.33704561	0.67898382	0.06765274
ENSG000001	TRIP12	0.2053313	0.56904198	0.06765869
ENSG000001	GNS	0.33501911	0.67700551	0.0676934
ENSG000001	FEZ2	0.20961149	0.5736945	0.06770318
ENSG000001	NUDT4	0.34061503	0.68148267	0.06774837
ENSG000001	ZNF791	0.37390295	0.70606132	0.0677739
ENSG00000C	FBXW11	0.13001963	0.48787839	0.06779428
ENSG000001	SECTM1	0.72553229	0.89686038	0.06780715
ENSG000001	MED19	0.41081025	0.72979497	0.06780735
ENSG000001	TRIM37	0.45066374	0.75341213	0.06780868
ENSG000001	NINL	0.66286695	0.86788287	0.06785887
ENSG000001	PLXNB2	0.39307886	0.72063539	0.06787515
ENSG000001	NEPRO	0.46052758	0.76015269	0.06788048
ENSG000001	JAG1	0.54911923	0.81187843	0.06789894
ENSG000001	H1-7	0.85683152	0.94924076	0.06791425
ENSG000001	MAP3K5	0.28440171	0.64253645	0.06800536
ENSG00000C	ZFP64	0.26719399	0.62930502	0.06801976
ENSG000001	TCP1	0.16455561	0.52786788	0.06802218
ENSG000001	CHSY1	0.49367028	0.78107975	0.06803535
ENSG000001	SAAL1	0.55454677	0.81426804	0.06804433
ENSG000001	UPF3A	0.46769737	0.76561035	0.0680537
ENSG000001	STXBP3	0.45458317	0.75583231	0.06806356
ENSG00000C	SEPHS1	0.42716288	0.73942744	0.06806931
ENSG000001	POLE4	0.4619439	0.76148052	0.06811057
ENSG000001	WRNIP1	0.3014112	0.65547284	0.06812178
ENSG000001	RBPMS	0.57638327	0.82397739	0.06814642
ENSG00000C	SH3YL1	0.64334135	0.85925257	0.06815159

ENSG000001	TBC1D19	0.6262359	0.85066154	0.06817164
ENSG000001	SSR1	0.20744126	0.57126603	0.06826212
ENSG000001	SH3PXD2A	0.44378403	0.74908353	0.06828351
ENSG000002	PCMTD2	0.65875954	0.86560481	0.06833031
ENSG000001	IQGAP2	0.37497633	0.70720386	0.06834598
ENSG000001	MITF	0.50109843	0.7853582	0.06836677
ENSG00000C	DDX20	0.32567861	0.67124189	0.06841672
ENSG000001	TLE1	0.35820574	0.69382497	0.06847328
ENSG000001	ASH1L	0.33871618	0.68032343	0.06847708
ENSG000001	SCPEP1	0.14209807	0.50068215	0.0684943
ENSG000001	INTS12	0.34524197	0.68522517	0.06849452
ENSG000001	SPN	0.36224365	0.69631231	0.06850225
ENSG000002	SPIB	0.32594539	0.67162122	0.0685192
ENSG000001	IFNLR1	0.47902881	0.77300644	0.06852034
ENSG000001	ACSS1	0.2039186	0.5688169	0.06853072
ENSG000001	AOPEP	0.34806075	0.6875026	0.0685534
ENSG000001	EVC2	0.71564909	0.89206159	0.06859177
ENSG000001	FANCI	0.44682993	0.75099423	0.06859381
ENSG00000C	DYNLL1	0.41006727	0.72943277	0.06860415
ENSG000001	ZFP90	0.49449443	0.78124152	0.06867572
ENSG000001	PBX3	0.33209071	0.67494767	0.0686882
ENSG000001	RRAS	0.44515773	0.74940749	0.0687069
ENSG000001	KNOP1	0.47805581	0.77199385	0.06871987
ENSG000002	TADA2A	0.76110849	0.91141904	0.06879029
ENSG000001	LRBA	0.39455766	0.72177025	0.06881329
ENSG00000C	CC2D2A	0.48488778	0.7769018	0.06881391
ENSG00000C	ZNF275	0.45018598	0.75290713	0.06883533
ENSG000001	ABHD12	0.34461909	0.68522517	0.0688426
ENSG000001	DCAF1	0.31090932	0.65949478	0.06885602
ENSG000001	TRPV2	0.25334247	0.61912837	0.06886222
ENSG000001	HOXB3	0.52910299	0.80117926	0.06887405
ENSG000001	AAGAB	0.2561534	0.62056088	0.06887896
ENSG000002	AGAP9	0.5098521	0.79143652	0.0688796
ENSG000001	MRM2	0.24261653	0.60820533	0.06891177
ENSG00000C	HEBP1	0.2048553	0.56891466	0.06895036
ENSG000001	PRKCG	0.89622557	0.9647318	0.06896511
ENSG00000C	CLASP1	0.10990113	0.46255597	0.06904761
ENSG00000C	CD79B	0.44252997	0.74855288	0.06910753
ENSG000001	PTPDC1	0.6191364	0.84711589	0.0691284
ENSG000001	TECTB	0.83397457	0.93938571	0.06913207
ENSG000001	FAM89A	0.61929888	0.84711589	0.06913737
ENSG00000C	MYDGF	0.41857838	0.73381176	0.06926793
ENSG000001	IARS1	0.39027796	0.71927554	0.06926999

ENSG000001	NREP	0.40852704	0.7282887	0.06934726
ENSG000001	SNRPC	0.40406734	0.72672584	0.06939746
ENSG000001	TIAL1	0.35457826	0.69189847	0.06940521
ENSG00000C	PCSK5	0.48350314	0.77585394	0.06943051
ENSG00000C	GPATCH2L	0.41286688	0.73073027	0.06944187
ENSG000001	ICA1L	0.63677809	0.85580792	0.06944461
ENSG000001	DCTN5	0.22337088	0.59081204	0.06944561
ENSG000001	PRDM4	0.1028954	0.45252237	0.06945425
ENSG00000C	SCD	0.39298988	0.72063539	0.06951743
ENSG00000C	KDM2B	0.28133882	0.63943639	0.06951793
ENSG000001	EMC1	0.32655719	0.67185873	0.06953684
ENSG000001	HEMK1	0.47164323	0.76694135	0.06954838
ENSG000001	SMAD7	0.31076188	0.65949478	0.0695646
ENSG00000C	SEL1L3	0.47395986	0.76910716	0.06960194
ENSG000001	INCENP	0.26036603	0.62385783	0.06960417
ENSG000001	CTPS1	0.3997851	0.72527731	0.06961115
ENSG000001	SLC11A2	0.29769135	0.65292644	0.06967136
ENSG000001	KLHL34	0.62871682	0.85212646	0.06973992
ENSG000002	ZNF850	0.59361838	0.83456808	0.06975324
ENSG000001	KPNA4	0.12950574	0.4871968	0.06977339
ENSG000001	FBXO30	0.48217865	0.77493168	0.06981587
ENSG000001	RGS12	0.35390947	0.69162813	0.06983555
ENSG000001	SDR39U1	0.46733815	0.76533215	0.06989103
ENSG000001	ENDOD1	0.26772797	0.63008279	0.06990338
ENSG000001	C12orf65	0.37247908	0.7044779	0.06990632
ENSG000001	FAM120AOS	0.1060813	0.4561473	0.06991973
ENSG000001	ELMOD3	0.18864988	0.55320717	0.06993236
ENSG000001	MRPL36	0.53491764	0.80413459	0.06997644
ENSG000001	COL4A6	0.88642405	0.96095764	0.07002053
ENSG00000C	POLR3E	0.44754659	0.75099423	0.07002354
ENSG00000C	PAK3	0.82589777	0.93591669	0.07008883
ENSG000001	TP53RK	0.4315048	0.74126857	0.07008907
ENSG000002	TRIM71	0.83483743	0.93977114	0.07009999
ENSG000001	C1QC	0.84560311	0.94449045	0.07015362
ENSG000001	RNF146	0.32740188	0.67217574	0.07017833
ENSG00000C	SEC22C	0.38771089	0.71668724	0.07022907
ENSG000001	QRFP	0.83413913	0.93944074	0.07023368
ENSG00000C	SUPT16H	0.11110618	0.46435807	0.07025225
ENSG000001	C9orf64	0.19160521	0.55704454	0.07027292
ENSG000001	NDUFA4L2	0.88164663	0.95932126	0.07027539
ENSG000001	SEC31A	0.12000683	0.47618546	0.07028876
ENSG000001	POLE3	0.33985734	0.68127111	0.0703327
ENSG000001	CTSW	0.49552512	0.78160414	0.07035764

ENSG000001	LILRB2	0.77192938	0.91507664	0.07035874
ENSG000001	RGS5	0.82917324	0.93682924	0.07036349
ENSG000001	STK33	0.85883438	0.95011881	0.07038162
ENSG00000C	SLC6A16	0.38159723	0.7106995	0.07039596
ENSG000001	MDM4	0.34986666	0.68924835	0.07039934
ENSG00000C	ME2	0.06553222	0.39248431	0.07040706
ENSG000002	N/A	0.87154787	0.95449478	0.07042705
ENSG000001	STK26	0.52837328	0.80062313	0.07044993
ENSG000001	CASP4	0.41318784	0.73077485	0.0704621
ENSG000001	SLC4A10	0.75697925	0.91082077	0.0704738
ENSG000002	FAM156A	0.7822644	0.9193471	0.07047457
ENSG000001	TSPYL4	0.48076864	0.77396792	0.07049596
ENSG000001	C11orf65	0.72119946	0.89490971	0.07050451
ENSG000001	CATSPER4	0.84367429	0.94370569	0.07053247
ENSG000001	STX8	0.48825691	0.778612	0.07056024
ENSG000001	BNIP1	0.56045241	0.81724883	0.07057113
ENSG00000C	BTK	0.04287872	0.35185871	0.07057521
ENSG000001	PARM1	0.59367387	0.83456808	0.07057571
ENSG000001	GRIN2D	0.67739233	0.87510133	0.0705789
ENSG000001	NUDT15	0.5650893	0.81916096	0.07060001
ENSG000001	RNF8	0.45179446	0.75386982	0.07061778
ENSG000001	MRPS5	0.37824135	0.70909881	0.07070947
ENSG000001	DIP2A	0.51458635	0.79420539	0.07074723
ENSG000001	CHTOP	0.14179275	0.50055578	0.07075421
ENSG000001	PTK2B	0.2929807	0.64970332	0.07077648
ENSG000001	TRAPPC8	0.35951714	0.69484359	0.07077762
ENSG000001	PKIA	0.60007503	0.83754165	0.07078024
ENSG000001	ITFG1	0.08363063	0.42260872	0.07082704
ENSG00000C	ARHGAP10	0.44378193	0.74908353	0.07086373
ENSG000001	RPL27	0.72579574	0.89686038	0.07086649
ENSG000001	ACKR3	0.60075081	0.83786213	0.07093172
ENSG000001	VPS13B	0.26994325	0.63142427	0.07097239
ENSG000001	ZNF331	0.55839023	0.81600055	0.07104499
ENSG00000C	ZCCHC8	0.24984579	0.61582699	0.0711597
ENSG000002	ZNF630	0.45431418	0.75565451	0.07124172
ENSG000001	CWC27	0.46025396	0.76008376	0.07126298
ENSG000001	PGBD1	0.65586499	0.86467033	0.07128385
ENSG000001	ZCCHC14	0.49695636	0.78237677	0.07130919
ENSG000001	MCM8	0.49350684	0.78107975	0.07131668
ENSG000001	CALM2	0.3313831	0.67444298	0.07133137
ENSG000001	DENND1A	0.50504567	0.78774511	0.07134227
ENSG000001	SEMA7A	0.49948831	0.78410505	0.07135218
ENSG00000C	ADSS2	0.14697698	0.5054701	0.07135913

ENSG000001	HEXD	0.34020958	0.68135285	0.07136605
ENSG000001	CBLB	0.44204479	0.74830001	0.07137578
ENSG000001	NDUFB6	0.28762388	0.64495772	0.07138104
ENSG000001	FKBP11	0.25714713	0.62086214	0.07138116
ENSG000001	TTC12	0.52933051	0.80120681	0.07139829
ENSG000001	SORBS2	0.87002604	0.95423131	0.07141086
ENSG000001	SETD4	0.27491106	0.6349546	0.07149962
ENSG000001	LSM12	0.22139994	0.58882965	0.07156163
ENSG000001	ZNF160	0.31520414	0.66312123	0.07156859
ENSG000001	TMCO4	0.29648974	0.65199276	0.07157087
ENSG000001	GAB1	0.31819832	0.66581089	0.07161925
ENSG000001	TSPO2	0.63511855	0.85504577	0.07165324
ENSG00000C	MDH1	0.52790405	0.80062313	0.07172523
ENSG000001	TBC1D12	0.52832583	0.80062313	0.07172898
ENSG000001	RGN	0.83741682	0.94120179	0.07178772
ENSG000001	DCAF5	0.07819596	0.41427772	0.07179579
ENSG000001	SYNM	0.57887277	0.82557375	0.07180198
ENSG000001	HERC3	0.12950493	0.4871968	0.07182381
ENSG000001	SPX	0.69031384	0.88074719	0.07184377
ENSG000002	TEX48	0.87615286	0.95676199	0.07188094
ENSG000001	UBE2D3	0.12910355	0.4871968	0.07190105
ENSG000001	NUDCD2	0.55040437	0.81252075	0.0719099
ENSG000001	REEP5	0.3538317	0.69160532	0.07191093
ENSG000001	DBR1	0.23704482	0.60384323	0.07191865
ENSG000001	GHSR	0.82225409	0.93539963	0.07191919
ENSG000001	ODAD1	0.64137329	0.85754101	0.07195807
ENSG000001	ZCCHC9	0.42407278	0.73732623	0.07196636
ENSG000001	SLC39A6	0.38129659	0.71047885	0.071991
ENSG000001	EIF2B1	0.19386321	0.56089752	0.07202644
ENSG00000C	PIAS2	0.49153679	0.78039335	0.07205973
ENSG000001	RGS9BP	0.88878343	0.96164016	0.07209156
ENSG000001	PM20D1	0.812324	0.93170624	0.07211635
ENSG000001	GRPEL1	0.21791119	0.58473377	0.07212124
ENSG000001	RREB1	0.11306321	0.46808189	0.07212276
ENSG000001	KIFBP	0.39697009	0.72308765	0.07212375
ENSG000001	GPATCH8	0.14967127	0.50933268	0.07217775
ENSG00000C	GSDMB	0.4201425	0.73468557	0.07220563
ENSG000001	IRF4	0.41205744	0.73041489	0.07223598
ENSG000002	SIGLEC12	0.76745984	0.91352929	0.07224361
ENSG00000C	PCM1	0.35162954	0.69054758	0.07225525
ENSG000001	NOLC1	0.1639982	0.52719041	0.07229615
ENSG000001	PTPRA	0.10232129	0.45171037	0.0723397
ENSG00000C	NFX1	0.27830401	0.63759905	0.07235233

ENSG000001	PCNX4	0.53078685	0.80212192	0.07238033
ENSG000001	OSR2	0.82929094	0.93682924	0.07245126
ENSG000001	TSC22D1	0.3328438	0.67523724	0.07249033
ENSG000001	FAM20C	0.48703188	0.77767904	0.07249805
ENSG000001	EPHB2	0.66370106	0.86832674	0.07250821
ENSG000001	ECHS1	0.1638217	0.52709285	0.07252325
ENSG00000(	ASTE1	0.39508619	0.72209218	0.07253502
ENSG000001	PTPRM	0.59690752	0.83614954	0.07255004
ENSG000001	ZNF669	0.40157893	0.72618744	0.07261491
ENSG000001	PPP1R8	0.2283262	0.59653313	0.07262315
ENSG00000(	IFT74	0.62664303	0.85080716	0.07264564
ENSG00000(	ZNF582	0.61429966	0.8451435	0.07266212
ENSG00000(	POLR1A	0.34943553	0.68873328	0.07266352
ENSG000001	CYB5D1	0.87327832	0.95510037	0.07268972
ENSG000001	DCP1B	0.39549402	0.72212079	0.07270833
ENSG000001	PSMD6	0.0722675	0.40373033	0.07275253
ENSG000001	YARS2	0.42616528	0.73856395	0.07277076
ENSG000001	PIP5K1A	0.04780927	0.35895177	0.07278593
ENSG000001	RPL39	0.78833957	0.92204735	0.07282855
ENSG000002	CSNK2A3	0.33189272	0.67488318	0.07283527
ENSG000001	WDR4	0.36378412	0.6970454	0.07283529
ENSG000001	BCL2L15	0.56050438	0.81725123	0.0728564
ENSG00000(	MBTD1	0.44821236	0.75119984	0.07286525
ENSG000002	ARMH2	0.56895183	0.82037292	0.07287561
ENSG000001	TNRC6B	0.18359835	0.54871392	0.07293276
ENSG000001	SPSB1	0.41350505	0.73118284	0.0729361
ENSG000001	LAMP5	0.57432008	0.82327493	0.07297336
ENSG000001	SLC41A2	0.67069297	0.87216822	0.07297737
ENSG000002	TXNIP	0.19024531	0.55527828	0.07298009
ENSG000001	ARL6IP5	0.33001097	0.67364255	0.07299878
ENSG000001	GDAP2	0.23900436	0.60520581	0.07301702
ENSG000001	ZNF615	0.46381683	0.7634274	0.0730489
ENSG000001	OMD	0.84518582	0.94421922	0.07306504
ENSG000001	ZSCAN16	0.32766317	0.67217574	0.07311184
ENSG00000(	ZMPSTE24	0.25376461	0.61921212	0.07311924
ENSG000001	COMMD9	0.23545877	0.60269577	0.07313392
ENSG000001	SMURF1	0.08256576	0.4220168	0.0731478
ENSG000001	RPUSD2	0.39119028	0.71964297	0.07324951
ENSG000001	NCALD	0.4805155	0.77383118	0.07326215
ENSG000001	CTNNB1	0.06794678	0.3972819	0.07333472
ENSG000001	HP1BP3	0.3251034	0.6708073	0.07334708
ENSG000001	ZMYM3	0.17169887	0.53584263	0.07336377
ENSG00000(	GEMIN8	0.43171054	0.74129485	0.07337358

ENSG000001	MSI2	0.47168412	0.76694135	0.07343803
ENSG000001	EEF2KMT	0.42810399	0.73995364	0.07346632
ENSG000001	ARL10	0.5712738	0.82159192	0.07350992
ENSG000001	CFDP1	0.42701402	0.7392501	0.07351134
ENSG000001	SELENOF	0.29822974	0.65366489	0.0735157
ENSG000001	XIRP2	0.88895889	0.96164016	0.07352097
ENSG000001	C2orf42	0.22683445	0.59438615	0.07355618
ENSG000001	SHOC2	0.38359899	0.7123445	0.07359119
ENSG000001	SLC20A2	0.30637394	0.65735608	0.07360312
ENSG000001	HNRNPA1L2	0.47506617	0.76999017	0.07360678
ENSG000001	MPP7	0.46190065	0.76148052	0.07364027
ENSG000001	NOL10	0.34713448	0.68653332	0.07371682
ENSG000001	NPAT	0.43802746	0.74561911	0.07374549
ENSG000002	RPL41	0.72422808	0.89621914	0.07376137
ENSG000001	SPCS2	0.22953547	0.59783342	0.07377625
ENSG000001	CA13	0.46544539	0.76438119	0.07377812
ENSG000001	TRAK2	0.27240363	0.63353982	0.07380232
ENSG000001	ADNP2	0.18798372	0.55211256	0.07380861
ENSG000001	PTPA	0.25134983	0.61695648	0.07381839
ENSG000001	FTO	0.18348976	0.54867831	0.07383669
ENSG000001	ZNF831	0.51113195	0.79171125	0.07387903
ENSG000001	C11orf71	0.48435129	0.77665533	0.07388074
ENSG000001	NEMP1	0.54311874	0.8084284	0.07389
ENSG000001	STAT4	0.53168506	0.80232066	0.07389369
ENSG000001	MMUT	0.2688697	0.63079288	0.07389644
ENSG000002	CCDC192	0.82661023	0.93615843	0.07391013
ENSG00000C	UBE2A	0.180883	0.54580882	0.07391625
ENSG000001	PANK1	0.67981871	0.87632711	0.07393537
ENSG000001	TRAF3	0.15771594	0.52077869	0.07396562
ENSG000002	TBC1D8	0.35056549	0.69014884	0.07399418
ENSG000001	IMP4	0.27276384	0.63378205	0.07401972
ENSG00000C	PDS5B	0.25064522	0.61651321	0.07403453
ENSG000001	ADD3	0.48704922	0.77767904	0.07405026
ENSG000001	PAFAH2	0.29744613	0.65274757	0.07407471
ENSG000001	IPP	0.40021015	0.72581393	0.07408192
ENSG000001	ETV6	0.3404786	0.68141884	0.07409698
ENSG000001	TRIM36	0.4261639	0.73856395	0.07410975
ENSG000001	ABHD1	0.6439356	0.85940463	0.07416771
ENSG000001	ACVR2B	0.65774335	0.86540022	0.074169
ENSG000001	MCMDC2	0.66157178	0.86736018	0.07417423
ENSG00000C	SAR1A	0.0464564	0.35774922	0.07424403
ENSG000001	METTL3	0.34263255	0.68347906	0.07425571
ENSG000001	LRRC56	0.84420516	0.94396799	0.07425969

ENSG00000(	MIPEP	0.43215959	0.74167374	0.07431997
ENSG000001	ZNF417	0.39059483	0.71962876	0.07434961
ENSG000001	PCBD2	0.49225398	0.7804495	0.07436065
ENSG000001	TCEANC2	0.20853823	0.57228774	0.07439858
ENSG000001	STRN	0.30462324	0.65635638	0.07441254
ENSG00000(	NDC1	0.49771939	0.78278241	0.07441292
ENSG000001	NOP9	0.16230747	0.52515336	0.0744252
ENSG000001	MT1X	0.50807687	0.79067965	0.07443337
ENSG000001	DROSHA	0.17258801	0.53689236	0.0744359
ENSG000001	SCN5A	0.8252999	0.93591669	0.07444121
ENSG000001	ZNF473	0.42965523	0.74091639	0.07444316
ENSG000001	RXYLT1	0.54495998	0.80952117	0.07447994
ENSG000001	POLR3GL	0.4455586	0.74969275	0.0744812
ENSG000001	AIFM3	0.42994244	0.74091639	0.07453284
ENSG00000(	PAPOLA	0.27441547	0.63464829	0.07456627
ENSG000001	RBM3	0.44096592	0.74750808	0.07457554
ENSG000001	ZNF660	0.63550485	0.85504577	0.07458825
ENSG000001	ASXL2	0.36667092	0.69886556	0.07461258
ENSG000002	UMAD1	0.26067999	0.62417543	0.07464081
ENSG000001	TNFRSF4	0.44281026	0.74863704	0.07469954
ENSG000001	ACSF2	0.34212018	0.68310736	0.07475877
ENSG000001	VEZF1	0.23085172	0.59855451	0.07475986
ENSG000001	NRL	0.41464676	0.73184916	0.0748155
ENSG000001	RGMB	0.55052803	0.81255555	0.07482546
ENSG000002	N/A	0.64222253	0.85825244	0.07485007
ENSG000001	DDX55	0.4231469	0.73695551	0.07488681
ENSG000001	ETFDH	0.28567371	0.64334037	0.07492125
ENSG000001	FLNC	0.87699448	0.9567976	0.07494299
ENSG000001	GLOD4	0.3828814	0.71198657	0.07494634
ENSG000002	FAM228B	0.36464968	0.69769659	0.07503738
ENSG000001	ORC4	0.43487974	0.74433605	0.07503811
ENSG000001	PRPF31	0.80518113	0.92897486	0.07504771
ENSG000002	PRR22	0.65142444	0.8634351	0.07508437
ENSG000001	ACAP2	0.36033955	0.69534637	0.07513005
ENSG000001	NPIPB3	0.36032714	0.69534637	0.07517513
ENSG000001	ARMC8	0.0735199	0.40498137	0.07520866
ENSG000001	TRIM45	0.69087997	0.88107274	0.07521144
ENSG000001	SLC22A15	0.46632718	0.76486809	0.07529476
ENSG000001	UBE2G2	0.35086389	0.69032381	0.07532885
ENSG000001	PTK2	0.23381323	0.60167183	0.07534931
ENSG000001	OAF	0.3468342	0.68647359	0.07535224
ENSG000001	PARD6B	0.61501057	0.84533626	0.0753587
ENSG000001	C16orf46	0.83231629	0.9384155	0.07537195

ENSG000001	MLH3	0.46156522	0.76122418	0.07537949
ENSG000001	COMMD7	0.33854455	0.68022544	0.07543512
ENSG000001	YWHAG	0.13470049	0.49415541	0.07544347
ENSG00000C	RASSF7	0.54153952	0.80788104	0.07545377
ENSG000001	AP3M1	0.19636059	0.5638811	0.07545782
ENSG000001	ZNF404	0.70114155	0.88513659	0.07546286
ENSG000001	TMEM17	0.85097453	0.94684256	0.0755264
ENSG000001	MAPK1IP1L	0.02796256	0.31159655	0.07552975
ENSG000001	STRN3	0.46406154	0.76349855	0.07553962
ENSG000001	TFF3	0.82947758	0.936855	0.07555625
ENSG000001	HDDC2	0.49088853	0.7800506	0.0755905
ENSG00000C	CACNA1S	0.83609976	0.94067016	0.07559964
ENSG000001	XBP1	0.30739803	0.65762361	0.07561096
ENSG000001	HAT1	0.61080729	0.84334674	0.07564281
ENSG000001	ALG2	0.33017464	0.67382451	0.07564473
ENSG000001	SNRPB2	0.37570632	0.70743725	0.07564925
ENSG000001	URB1	0.28091589	0.63927558	0.07565618
ENSG000001	RFC4	0.5579169	0.81587881	0.07571663
ENSG00000C	SLC44A1	0.23903337	0.60520581	0.07572163
ENSG000001	BCO1	0.83721941	0.94120179	0.0757525
ENSG000001	MYCL	0.31488086	0.662749	0.07575268
ENSG000001	SPEF2	0.68007372	0.87644445	0.07585464
ENSG000001	HSPE1	0.54872508	0.81161901	0.07588879
ENSG000001	RNASE3	0.55691782	0.81553703	0.07589249
ENSG000001	RNF214	0.19897593	0.56598606	0.07589675
ENSG000001	SAT2	0.34809133	0.6875026	0.0759313
ENSG000001	NFKBIZ	0.32903232	0.67287758	0.07593472
ENSG000001	CEP41	0.41979085	0.73466853	0.07594404
ENSG000001	DHFR2	0.44990332	0.75279291	0.07597183
ENSG000001	ATP13A1	0.36617628	0.69855054	0.07597637
ENSG000001	C1D	0.38598207	0.715021	0.07598902
ENSG000001	NBPF6	0.87191539	0.95457921	0.07599478
ENSG000001	ACAA2	0.35641196	0.69246335	0.07607538
ENSG000001	MFF	0.33893208	0.68041384	0.07609128
ENSG000001	DPPA4	0.75520325	0.91013398	0.07613392
ENSG000001	ADPRS	0.28117598	0.63941315	0.07638123
ENSG000001	MEAF6	0.31107592	0.65949478	0.07639894
ENSG000002	N/A	0.86138907	0.95080722	0.07642417
ENSG000001	ADAM10	0.33389203	0.67615426	0.07643395
ENSG000001	NRP2	0.75404252	0.90944172	0.07644497
ENSG000002	DNASE1	0.37735866	0.7086984	0.07644936
ENSG000001	MRPS25	0.52830979	0.80062313	0.0764572
ENSG000001	SMC2	0.51227384	0.79216083	0.07647511

ENSG00000(	REST	0.25796024	0.62198226	0.07653808
ENSG000001	EEF1B2	0.71521029	0.89178847	0.07653969
ENSG000001	HCAR2	0.55171561	0.81323014	0.07654014
ENSG000001	CEP112	0.80776756	0.92977176	0.07654131
ENSG000001	RELL2	0.34294966	0.68359637	0.07660126
ENSG000001	CLHC1	0.58608679	0.82959975	0.07665051
ENSG000002	CTAGE1	0.82381447	0.9356656	0.07665784
ENSG000001	SET	0.14472397	0.50329472	0.07674494
ENSG000001	ANKRD17	0.09419838	0.43939485	0.07674801
ENSG000001	MTCL1	0.60973176	0.84290158	0.0767598
ENSG000001	CYP3A5	0.65503916	0.86442301	0.07677644
ENSG000001	KBTBD2	0.2695858	0.6310094	0.07677701
ENSG000001	AP1S1	0.29635703	0.65199276	0.0768436
ENSG000001	CSNK1A1	0.09636052	0.44215472	0.07686327
ENSG000001	ACTA1	0.89478131	0.96412642	0.07690036
ENSG000001	AHR	0.3829696	0.71198657	0.07690982
ENSG000001	CNOT9	0.26846059	0.63073283	0.07695675
ENSG000001	STAM	0.22849873	0.59658045	0.07698169
ENSG00000(	SDHA	0.14691771	0.5054701	0.07698269
ENSG000002	ZNF469	0.47814237	0.77203636	0.07699725
ENSG000001	USP38	0.20125274	0.56773237	0.07704492
ENSG000001	EBNA1BP2	0.38036039	0.70987972	0.07705187
ENSG000001	ATG14	0.35571233	0.69207883	0.07708305
ENSG000001	REV1	0.39344908	0.72063539	0.07714595
ENSG000001	CCNYL1	0.15646529	0.51911584	0.07716834
ENSG000001	CRIM1	0.18459707	0.5498217	0.07725003
ENSG000001	PURB	0.39846168	0.72434196	0.07725419
ENSG00000(	FUT8	0.45102959	0.75358792	0.07727375
ENSG000001	POP1	0.224716	0.59200912	0.07727496
ENSG000001	NTPCR	0.43089081	0.74126857	0.07728021
ENSG000001	SLC7A7	0.16774113	0.53220434	0.07729057
ENSG000001	MEIOB	0.84269039	0.94324858	0.07729809
ENSG000001	LUZP1	0.1697938	0.53437545	0.07732697
ENSG000001	RHNO1	0.36678018	0.69892851	0.07734248
ENSG000001	WTAP	0.24454344	0.61049383	0.0773928
ENSG00000(	SLC25A43	0.29250622	0.64953823	0.0774086
ENSG00000(	RANBP9	0.14590836	0.50430399	0.07743234
ENSG000001	PLPBP	0.02755687	0.3113392	0.07744339
ENSG000001	OTULINL	0.46961416	0.7662622	0.07744393
ENSG000001	TMEM42	0.52044242	0.79709497	0.07750233
ENSG000001	ILK	0.52614584	0.80013393	0.07750955
ENSG000001	RPS27	0.7447824	0.90628646	0.07753584
ENSG00000(	ST7	0.30812289	0.65776904	0.07754396

ENSG000001	ZNF140	0.43488171	0.74433605	0.07754535
ENSG000002	FASTKD5	0.05379452	0.37194714	0.07757933
ENSG000001	PRMT9	0.41112418	0.72995333	0.07758099
ENSG00000C	CARMIL1	0.32457799	0.6701644	0.0775819
ENSG000001	HOXB4	0.43349182	0.7430177	0.07758997
ENSG000001	SLC1A4	0.37223794	0.70435002	0.0775955
ENSG000001	RAN	0.3904881	0.71951376	0.07760076
ENSG00000C	ACOT7	0.45129126	0.75378336	0.07761086
ENSG000001	CCDC77	0.2754735	0.63512113	0.0776329
ENSG000001	UVRAG	0.05058446	0.36286396	0.07763899
ENSG00000C	RNF13	0.32987474	0.67347871	0.07771652
ENSG000001	ZNF79	0.20832619	0.57196353	0.0777327
ENSG000001	COG7	0.14941259	0.50925123	0.07773902
ENSG00000C	PKD2L2	0.61790261	0.84662697	0.07774044
ENSG00000C	TUT7	0.41804248	0.7336528	0.07775587
ENSG000001	EHD4	0.20975911	0.57395664	0.0777689
ENSG000001	NOXO1	0.87729535	0.95703283	0.07777611
ENSG000001	CWC15	0.42523574	0.73817463	0.07780229
ENSG000001	ATP5MK	0.53635163	0.80444134	0.07787402
ENSG000001	ZNF17	0.56906738	0.82037292	0.07788084
ENSG000001	VAV3	0.20869336	0.57248761	0.07789987
ENSG000001	PHF3	0.16678516	0.5312065	0.07790523
ENSG000001	AUTS2	0.44690032	0.75099423	0.07791868
ENSG000001	CLUAP1	0.49469659	0.78124152	0.0779439
ENSG000001	MICALL1	0.42661342	0.73902934	0.07794453
ENSG000001	NCOA3	0.11042876	0.46307639	0.07796809
ENSG000001	BCAS2	0.36275084	0.69656832	0.077978
ENSG000001	RNF38	0.07235667	0.40373033	0.07797851
ENSG00000C	RBMS2	0.34411226	0.68472435	0.07803267
ENSG000001	ARV1	0.49274834	0.7804495	0.07803313
ENSG000001	COX11	0.49273617	0.7804495	0.07810764
ENSG000001	SFT2D1	0.15349147	0.51472848	0.07814482
ENSG000001	PLSCR3	0.62754443	0.8511716	0.07819759
ENSG000002	S1PR3	0.44105313	0.74750808	0.07821481
ENSG000001	VRK1	0.55721592	0.81587881	0.07821742
ENSG000001	TOMM22	0.32714973	0.67217574	0.07822433
ENSG000001	ZBTB40	0.38063338	0.70987972	0.07827584
ENSG000002	TIAF1	0.42913128	0.74078297	0.07828835
ENSG000001	GZMK	0.67511466	0.87449834	0.07832536
ENSG000002	CNPY2	0.45651347	0.75690894	0.07832797
ENSG000001	HSDL1	0.25554485	0.62030905	0.07833308
ENSG000002	EIF5AL1	0.23864005	0.60492348	0.07833493
ENSG00000C	LIMA1	0.56193565	0.81801588	0.07833993

ENSG000001	PWWP2A	0.27640339	0.6360577	0.07834526
ENSG000001	OR2L13	0.85612147	0.94899264	0.07836568
ENSG000001	SEPTIN7	0.3210785	0.66766831	0.07839183
ENSG000001	ZNF426	0.37392507	0.70606132	0.07840534
ENSG000001	ADH6	0.8021492	0.92757225	0.07841025
ENSG000001	EFCAB14	0.10705199	0.45778331	0.07842627
ENSG00000C	ELOVL5	0.29359265	0.65034978	0.07844005
ENSG000001	KCNK13	0.54812396	0.81141647	0.07849283
ENSG000001	ELOC	0.42750627	0.73955032	0.07849423
ENSG000001	GATD3A	0.40495161	0.72672584	0.07849638
ENSG000001	SBSN	0.86376359	0.95211331	0.07853404
ENSG000001	WDR19	0.44985932	0.75279291	0.07854971
ENSG000001	UTP6	0.27203858	0.63323084	0.07861856
ENSG000001	MRPL48	0.54240974	0.80815796	0.07862388
ENSG000001	DHX35	0.35646268	0.69246335	0.07865442
ENSG000001	SLC35A4	0.14127487	0.50055578	0.07870469
ENSG000001	TCEAL8	0.60934556	0.84246798	0.07872168
ENSG000002	ERCC6	0.31957151	0.66652314	0.07872315
ENSG000001	ARMH4	0.8754915	0.95649008	0.0787598
ENSG000001	PABIR1	0.16756738	0.53192406	0.07878356
ENSG000001	SAMD11	0.88903002	0.96164016	0.07880409
ENSG000001	MAMDC4	0.42950069	0.74091639	0.07886866
ENSG000001	UBR3	0.31304739	0.6613866	0.07889949
ENSG000001	CCDC32	0.27759893	0.63710347	0.07890008
ENSG000001	CHADL	0.72841579	0.89755262	0.07897799
ENSG000001	SGTB	0.29790554	0.65330798	0.07898804
ENSG000001	PPP1R15B	0.34365389	0.68437426	0.07900035
ENSG000001	BMT2	0.4427714	0.74863704	0.07904503
ENSG000001	NRTN	0.85779228	0.94972056	0.0790722
ENSG000001	MRPL21	0.36175785	0.69588937	0.0790881
ENSG000002	SDHD	0.20437294	0.5688169	0.0790893
ENSG000001	PGBD4	0.59566754	0.83572573	0.07913654
ENSG00000C	EXD2	0.37594007	0.70769082	0.07914262
ENSG000001	S1PR1	0.4946104	0.78124152	0.07915405
ENSG000002	STK38L	0.41923055	0.73438543	0.07918016
ENSG000002	TYW1B	0.3902297	0.71927554	0.07921042
ENSG000001	LIMK1	0.41135449	0.72999304	0.07932319
ENSG000002	N/A	0.80863784	0.93001	0.0794089
ENSG00000C	NRIP2	0.44521888	0.74940749	0.07943527
ENSG00000C	RRM2B	0.51929067	0.79627184	0.07952282
ENSG000002	ISY1-RAB43	0.82523799	0.93591669	0.079544
ENSG000001	KIN	0.30719284	0.65762361	0.07955205
ENSG000001	RDH16	0.57193545	0.82170634	0.07957774

ENSG00000(	MED17	0.24438606	0.6102886	0.07958931
ENSG000001	CDK5RAP1	0.25797946	0.62198226	0.07960097
ENSG000001	RNF121	0.21860346	0.58536368	0.07960506
ENSG000001	RTL6	0.32609263	0.67170284	0.07960613
ENSG000001	CD300C	0.27768927	0.63711187	0.07970137
ENSG000001	DPH5	0.62973898	0.85287112	0.07973568
ENSG00000(	EZR	0.11273117	0.46759554	0.07976428
ENSG00000(	MGAT4A	0.55773818	0.81587881	0.079786
ENSG000001	EHBP1	0.36280434	0.69656832	0.079786
ENSG000001	EMB	0.37972491	0.70965485	0.07980516
ENSG000001	VKORC1L1	0.192673	0.55854875	0.07984395
ENSG000001	ZSCAN20	0.49368895	0.78107975	0.07985522
ENSG00000(	POU1F1	0.85333856	0.94777027	0.07987945
ENSG000001	AP4S1	0.3646636	0.69769659	0.07988523
ENSG000001	CYP20A1	0.26865354	0.63079288	0.07991842
ENSG000001	PELI1	0.48986854	0.77995113	0.07993912
ENSG000001	TBC1D5	0.08165864	0.41943963	0.07994188
ENSG000001	SERTAD1	0.30246359	0.65591444	0.07997046
ENSG000001	INSIG1	0.20605274	0.56956674	0.07998117
ENSG00000(	CELSR1	0.51634169	0.79520352	0.07998489
ENSG000001	MBD2	0.05027341	0.36227834	0.07999351
ENSG000001	LCA5L	0.76580353	0.91274697	0.07999463
ENSG000001	C5orf46	0.82653794	0.93615843	0.08006518
ENSG000001	SAMD12	0.65593372	0.86469073	0.08008464
ENSG000001	LGALSL	0.31376301	0.66194513	0.08010772
ENSG000001	STAG1	0.27153114	0.63300088	0.08015429
ENSG000001	RIC8B	0.43640687	0.74496191	0.08016373
ENSG000001	SLAMF1	0.47017739	0.7662622	0.08022854
ENSG000002	PEX26	0.215466	0.58134288	0.08024992
ENSG000001	SNRPA1	0.3492742	0.68858669	0.08029424
ENSG000001	POGZ	0.04396163	0.35385593	0.08033141
ENSG00000(	CDC23	0.26147843	0.62545387	0.08033875
ENSG00000(	TXNDC16	0.48419269	0.77655439	0.08038089
ENSG000001	TMEM168	0.49251771	0.7804495	0.08038394
ENSG000001	AMHR2	0.82573332	0.93591669	0.08044661
ENSG000001	CTSH	0.20049291	0.56724227	0.08046436
ENSG000001	FAM183A	0.76718749	0.91352929	0.08049525
ENSG000001	PJA2	0.37846302	0.7092687	0.08049966
ENSG000001	GFRA2	0.62314206	0.84930015	0.08050336
ENSG000001	VPS33B	0.40254661	0.72669629	0.08052175
ENSG00000(	TXLNG	0.40248139	0.72669629	0.08053323
ENSG000001	TBCCD1	0.3944456	0.72171376	0.08054025
ENSG000001	VGLL4	0.18722475	0.55131813	0.08054951

ENSG000001	THOC7	0.34027778	0.68135285	0.08071145
ENSG000001	SLC25A25	0.19946172	0.56600138	0.08075974
ENSG000001	TGFBRAP1	0.1610828	0.52404357	0.08077191
ENSG000001	METTL21A	0.39587409	0.72222558	0.08079673
ENSG000002	CDRT15	0.77023912	0.91423988	0.08080304
ENSG000001	ZNF132	0.47626312	0.77076491	0.08085337
ENSG000001	NRARP	0.81285244	0.93170624	0.08088417
ENSG000001	PPP6R3	0.21636894	0.58303866	0.08098677
ENSG000001	MAGEE1	0.47597676	0.77037825	0.08103478
ENSG000002	OR2A7	0.75413091	0.90944172	0.08105932
ENSG000001	SLC38A9	0.30209587	0.65591444	0.08106335
ENSG000001	NLGN4Y	0.76497022	0.91260342	0.08108135
ENSG000001	PDCD6IP	0.05762902	0.37715532	0.08112203
ENSG000001	ZNF704	0.80669304	0.92967903	0.08113465
ENSG000001	ALKBH1	0.24891829	0.61478169	0.08113943
ENSG00000C	ACP3	0.18306254	0.5476216	0.08115368
ENSG000001	TMEM208	0.31253425	0.66090624	0.08118587
ENSG000001	TUT4	0.33778175	0.67946062	0.08122628
ENSG000001	ZNF180	0.44871783	0.75173663	0.08123507
ENSG000001	IQCG	0.45588393	0.75632853	0.08128437
ENSG000001	PLEKHG4B	0.86378919	0.95211331	0.08128733
ENSG000001	DYNC2LI1	0.63441845	0.85503542	0.08132841
ENSG000002	CLEC5A	0.45674503	0.75713824	0.08136506
ENSG000001	ZNF318	0.24309196	0.60864829	0.08144143
ENSG000001	PPP4R3A	0.16786945	0.53236254	0.08145177
ENSG000001	GRM6	0.85106293	0.94687603	0.08146082
ENSG00000C	TM9SF3	0.32825183	0.67253311	0.08146206
ENSG000001	RBBP4	0.18548277	0.55033096	0.08147796
ENSG000001	ANKRD27	0.04386754	0.35372007	0.08147821
ENSG000002	MPIG6B	0.70999662	0.88993477	0.08149418
ENSG000001	KREMEN2	0.74784557	0.90731063	0.08150159
ENSG000001	TRMT10B	0.34624503	0.68602569	0.08151444
ENSG000001	THG1L	0.39618427	0.72246676	0.08158587
ENSG000001	CDHR3	0.57469174	0.82341424	0.08159284
ENSG000001	RHOC	0.37972652	0.70965485	0.08159308
ENSG000001	ACADSB	0.53439562	0.80401157	0.08160686
ENSG00000C	IPCEF1	0.09117033	0.43488031	0.08160696
ENSG000001	RNF139	0.36468257	0.69769659	0.08170331
ENSG000001	SLC25A22	0.34974819	0.6891822	0.0817273
ENSG000001	CLEC18C	0.86572313	0.95237042	0.08173277
ENSG000001	TAS2R4	0.51826572	0.79572786	0.08179454
ENSG000001	C3orf20	0.70465571	0.88714389	0.08182941
ENSG000002	DCP1A	0.0229827	0.29616677	0.08183008

ENSG00000(	STRAP	0.15270765	0.51346327	0.08185711
ENSG000001	SMIM4	0.49140237	0.78031845	0.08185715
ENSG000001	SUGT1	0.36009257	0.69520391	0.08187918
ENSG000001	CHD6	0.31716053	0.66456776	0.0818997
ENSG00000(	ARHGAP44	0.52242509	0.7979767	0.08192655
ENSG000001	ZNF14	0.50822759	0.79067965	0.08194096
ENSG000001	COX7A2L	0.42395203	0.73732623	0.08194979
ENSG000001	TMBIM4	0.18695024	0.55108473	0.08198114
ENSG000002	ATP5PO	0.47777606	0.77199385	0.08199119
ENSG000001	SERINC1	0.34426232	0.68479845	0.08199701
ENSG000001	CALHM2	0.24099004	0.60721888	0.08200356
ENSG000001	RAB6A	0.14462063	0.50315089	0.08200541
ENSG000001	MRPL47	0.66470893	0.86885195	0.08207256
ENSG000001	BNIP2	0.27841405	0.63759905	0.0820974
ENSG000001	KIAA1143	0.33750451	0.67940251	0.08210032
ENSG000001	PRDX1	0.27905196	0.63787563	0.08213187
ENSG000001	DLK2	0.43151486	0.74126857	0.08219091
ENSG000001	MANF	0.32416815	0.6699145	0.08219968
ENSG000001	MAML2	0.28200629	0.63987369	0.08223503
ENSG000001	TEAD1	0.86013362	0.95064852	0.08225208
ENSG000001	LCOR	0.25041459	0.61633798	0.08226448
ENSG00000(	RBBP9	0.32563571	0.67124189	0.08228316
ENSG000001	NAGA	0.15337489	0.51472848	0.0822954
ENSG000001	CACYBP	0.35544129	0.69207883	0.08230649
ENSG000001	INIP	0.14571083	0.50430399	0.08231167
ENSG000001	ZBTB2	0.18683732	0.55108473	0.08233532
ENSG00000(	SCIN	0.79543031	0.92462665	0.08238015
ENSG000001	ZNF830	0.25710521	0.62086214	0.08239784
ENSG000001	SCML2	0.5579994	0.81587881	0.08242359
ENSG000001	RPL35	0.62364326	0.84957594	0.08243303
ENSG00000(	SPAST	0.30719241	0.65762361	0.08243887
ENSG000001	PAAF1	0.37426001	0.70633918	0.08256397
ENSG000001	RNF11	0.39156154	0.71964297	0.08256479
ENSG000001	IL22RA2	0.75554912	0.91041566	0.08256948
ENSG000001	HMGB1	0.5399697	0.80698777	0.08257888
ENSG000001	PAPOLG	0.12631356	0.48388732	0.08257921
ENSG000001	TSSK4	0.24725416	0.6133006	0.08263421
ENSG000001	LRCH3	0.07561472	0.41015711	0.08264429
ENSG000001	SLC16A8	0.83236189	0.9384155	0.08267228
ENSG000001	TIMMDC1	0.28444614	0.64253645	0.08268699
ENSG000001	CHRM5	0.62083355	0.84823918	0.08272158
ENSG000001	ZNF230	0.34917418	0.68858669	0.08273076
ENSG000001	TOGARAM2	0.51712823	0.79538014	0.08279188

ENSG000001	GAS6	0.44224394	0.74838091	0.08284435
ENSG000001	ANKAR	0.48967474	0.77987726	0.08296829
ENSG000001	SH3BGRL	0.09899386	0.4460792	0.08303798
ENSG00000C	ERC1	0.16972227	0.53437545	0.08305009
ENSG00000C	RFX3	0.48417772	0.77655439	0.08305021
ENSG000001	PIGN	0.23041507	0.59855451	0.08308067
ENSG000001	CDK2	0.24769591	0.61375236	0.08308262
ENSG000001	GSDMA	0.76090851	0.91141904	0.08311458
ENSG000001	CXorf38	0.05985561	0.38277429	0.08314023
ENSG000001	PFKFB1	0.69309348	0.88183499	0.08315148
ENSG000001	BCL10	0.07295823	0.40435477	0.08315414
ENSG000001	RGS20	0.8575745	0.94961808	0.08315823
ENSG000001	ZDHHC4	0.2897589	0.64777851	0.08319881
ENSG000001	RNF212	0.57396607	0.82291277	0.08325272
ENSG000001	CSRNP2	0.15598378	0.51836035	0.08330677
ENSG00000C	DHRS9	0.61086457	0.84334674	0.08331151
ENSG000001	METTL25	0.37968923	0.70965485	0.08332939
ENSG000001	TOPBP1	0.177434	0.54235614	0.08334027
ENSG000001	CCDC6	0.25169167	0.6172357	0.08334676
ENSG000001	DONSON	0.46396547	0.7634274	0.08334878
ENSG000001	OCLN	0.80771984	0.92977176	0.08338588
ENSG00000C	TF	0.80590082	0.92949365	0.0834021
ENSG000001	TBC1D8B	0.63658642	0.85572098	0.0834069
ENSG000001	AP3B1	0.04036708	0.34451913	0.08341669
ENSG000001	STEAP1B	0.73742003	0.90250162	0.08343974
ENSG000001	SLC16A10	0.68600336	0.8799229	0.08344653
ENSG000001	G3BP1	0.1964963	0.56389367	0.08344749
ENSG000001	RALGAPB	0.1544576	0.51582229	0.08345506
ENSG000001	SLC9A6	0.32183523	0.66822528	0.08351489
ENSG000001	KCNH7	0.51035158	0.79143652	0.08355369
ENSG000001	PDCL3	0.281252	0.63941315	0.08357756
ENSG000001	AMOTL2	0.87333716	0.95510039	0.08363184
ENSG00000C	SERPINB1	0.18995162	0.55472021	0.08364028
ENSG000001	ZNF485	0.56544459	0.81924606	0.08364354
ENSG000001	ABCB7	0.40589784	0.72742568	0.08366916
ENSG00000C	DAZAP1	0.19180875	0.55723719	0.08368318
ENSG000001	TRAPPC11	0.11213848	0.46614115	0.08370821
ENSG000001	EFL1	0.15012604	0.50988869	0.08371966
ENSG000001	CCDC138	0.66581081	0.86959219	0.08373344
ENSG000001	GPT2	0.31641321	0.66372668	0.08378409
ENSG000001	NPIPB12	0.3310601	0.67443203	0.08385718
ENSG000001	ANXA2	0.21554473	0.58145868	0.0838654
ENSG000002	ZCCHC3	0.19977377	0.56650131	0.08393941

ENSG000001	ANKRD18A	0.74182645	0.90543757	0.08399995
ENSG00000C	TMED2	0.22145093	0.58882965	0.08400267
ENSG000001	ORC5	0.40735795	0.72768322	0.08400439
ENSG000001	C14orf28	0.45767059	0.75749027	0.08401411
ENSG000001	BRS3	0.86508666	0.95229913	0.08403618
ENSG000001	DBH	0.58573413	0.82959975	0.08404436
ENSG000001	SNX12	0.03459964	0.32831054	0.08405522
ENSG000001	SMPD3	0.53640983	0.80444134	0.08406609
ENSG000001	MRPS16	0.17442062	0.53940156	0.08408765
ENSG000001	ERG28	0.31014496	0.65948451	0.08413962
ENSG000001	DCPS	0.15026402	0.5100887	0.08416898
ENSG000001	NODAL	0.77612463	0.91690146	0.08417141
ENSG000001	ACP1	0.31973887	0.66678654	0.08418795
ENSG000001	IL18BP	0.12099706	0.47724625	0.08425142
ENSG000001	PHC1	0.384409	0.71275568	0.08425638
ENSG000001	TPK1	0.18752836	0.55167498	0.08428561
ENSG000001	PHAX	0.35088024	0.69032381	0.08432973
ENSG000001	PPP1CC	0.25330244	0.61912837	0.08436369
ENSG000001	TMED3	0.27749712	0.63709819	0.08436932
ENSG000001	ITPRIPL1	0.39229067	0.72005693	0.08438129
ENSG000001	GPRC5B	0.65721307	0.86522797	0.08443063
ENSG000001	CERT1	0.26793109	0.63017463	0.08446017
ENSG000001	AGAP1	0.47593245	0.77037825	0.08447796
ENSG000001	ABHD6	0.25765569	0.62181264	0.08447859
ENSG000001	TSR2	0.14337891	0.50145476	0.08449011
ENSG000001	ARID2	0.21669013	0.58337291	0.08453898
ENSG000001	DHX37	0.17177498	0.53586381	0.08454067
ENSG00000C	RNF31	0.32782909	0.67234571	0.08454237
ENSG000002	GLYATL1B	0.88305437	0.96010136	0.08455362
ENSG000001	RAB1A	0.05739687	0.37700366	0.08456923
ENSG000001	URB2	0.29416677	0.65044158	0.08457335
ENSG000001	VPS26C	0.03757385	0.33471496	0.08460028
ENSG000001	SERPINB2	0.6200599	0.84761181	0.0846179
ENSG000001	CCDC141	0.59995177	0.83754165	0.08462287
ENSG000001	CILK1	0.40306756	0.72672584	0.08462364
ENSG000001	MCFD2	0.24491612	0.61095454	0.08463191
ENSG00000C	SPRTN	0.25576582	0.62038706	0.08463244
ENSG000001	IPO8	0.14193846	0.50068215	0.08467151
ENSG00000C	TNRC6A	0.17988165	0.54485895	0.08467361
ENSG000001	BYSL	0.23082513	0.59855451	0.08469144
ENSG000001	FFAR2	0.54752494	0.81118038	0.08469176
ENSG000001	CPSF3	0.24137565	0.60721888	0.08472354
ENSG000001	SHISA5	0.44235879	0.74849725	0.08473302

ENSG00000(	R3HDM1	0.13511719	0.49445522	0.08481437
ENSG000001	PWP1	0.30649251	0.65735608	0.08481871
ENSG000001	FBXL4	0.34179387	0.68304087	0.08490556
ENSG000001	TSR1	0.17876942	0.54395471	0.08494978
ENSG000001	ZNF419	0.43162752	0.74126857	0.08502496
ENSG000001	VPS37A	0.30130513	0.65546137	0.08505351
ENSG00000(	MRE11	0.34833146	0.68768967	0.08506942
ENSG00000(	ALDH3A2	0.19140419	0.55685897	0.08509194
ENSG000001	CAMK4	0.60235281	0.83852406	0.08514359
ENSG000001	FKBP14	0.46267954	0.7623062	0.08516154
ENSG000001	SLC4A1AP	0.01106959	0.24454004	0.08517461
ENSG000001	ATXN7L1	0.19135038	0.55685897	0.08520944
ENSG00000(	SYT7	0.81645236	0.93271912	0.08523369
ENSG000001	POC5	0.36422886	0.6973647	0.08531981
ENSG000001	RFWD3	0.09104286	0.43488031	0.0853205
ENSG000001	CA5A	0.80396614	0.92881463	0.0853219
ENSG000001	NABP1	0.35339489	0.69126085	0.08537422
ENSG000001	MRPS36	0.48255066	0.77529933	0.08538018
ENSG000001	MRPL40	0.45726908	0.75742878	0.08549172
ENSG000001	ATG4C	0.41549412	0.73221244	0.08554849
ENSG000001	C3orf80	0.83318618	0.93878551	0.08557833
ENSG000001	HACD4	0.22668082	0.59419473	0.08558502
ENSG000001	EAF1	0.06723156	0.39561184	0.08563861
ENSG000001	GLI3	0.83313721	0.93878551	0.08567008
ENSG000001	ZFP2	0.80543164	0.92915052	0.08567283
ENSG000001	UBQLN2	0.10762644	0.45866943	0.08569638
ENSG000001	UTP14A	0.2146393	0.58023513	0.08569944
ENSG000001	GPRIN3	0.53528315	0.80413676	0.08570623
ENSG000001	AK3	0.39585883	0.72222558	0.08572364
ENSG000001	FAM220A	0.30544139	0.65688103	0.08574667
ENSG000001	PCBD1	0.28134099	0.63943639	0.08574855
ENSG000001	CCDC180	0.52127847	0.79758332	0.08576337
ENSG000001	ATP5MC3	0.27527713	0.63499184	0.0857936
ENSG000001	UQCRQ	0.57555139	0.82346952	0.08588039
ENSG000001	LIAS	0.34710737	0.68653332	0.08589331
ENSG000001	SQOR	0.42735867	0.73948387	0.08589553
ENSG000001	MRPL39	0.51869982	0.79581679	0.08591555
ENSG000001	DNAJB7	0.86133667	0.95080722	0.08591605
ENSG00000(	UTP18	0.28677134	0.64421192	0.08596139
ENSG000001	SERBP1	0.136003	0.49590853	0.08596472
ENSG000001	RIOX1	0.1783868	0.54332516	0.08603659
ENSG000001	CDK8	0.30315439	0.65593778	0.08603884
ENSG000001	DDX50	0.39325696	0.72063539	0.08605558

ENSG00000(	TM7SF3	0.28050034	0.63927558	0.08614291
ENSG000001	UPF3B	0.39147246	0.71964297	0.08621304
ENSG00000(	ELOA	0.01924954	0.28088168	0.08624928
ENSG00000(	SMC1A	0.08367656	0.42260872	0.08627467
ENSG000001	IRAK4	0.11573466	0.47120954	0.08635277
ENSG000001	CCDC25	0.4408378	0.74750808	0.08637474
ENSG000001	SLC43A3	0.14099665	0.50051652	0.08638783
ENSG000001	DCAF7	0.01678794	0.2704224	0.08639266
ENSG000001	KATNAL1	0.48911445	0.77944365	0.08641417
ENSG000001	GINS1	0.51795076	0.79566132	0.08643963
ENSG000001	PIK3R1	0.36624708	0.69855054	0.08646554
ENSG000001	RIOX2	0.40793758	0.72771726	0.0864813
ENSG000001	F2RL2	0.73664593	0.90203016	0.08649562
ENSG000001	ZNF766	0.3664741	0.69873648	0.08655228
ENSG000001	ICOSLG	0.42737677	0.73948387	0.08655879
ENSG000001	PARP8	0.23847089	0.60472464	0.08661654
ENSG000001	ZNF559	0.38044634	0.70987972	0.08663614
ENSG000001	RAD51B	0.46881032	0.76611043	0.08666734
ENSG000001	MBIP	0.54763466	0.81121445	0.08666839
ENSG000001	GLRX3	0.33300227	0.67532519	0.08667105
ENSG000001	RUNX1	0.07129769	0.40241675	0.0866769
ENSG000001	NUCB1	0.50373307	0.78675515	0.08671895
ENSG000001	AJUBA	0.59996271	0.83754165	0.08672195
ENSG00000(	ZFAND6	0.3337113	0.67612558	0.0867902
ENSG000001	CDKN2AIP	0.37029755	0.70302695	0.08681532
ENSG000001	ASRGL1	0.30650673	0.65735608	0.08681741
ENSG000001	MMS22L	0.44212497	0.74830001	0.08682718
ENSG000001	ELMOD1	0.78359039	0.91983554	0.08683039
ENSG000001	PKIG	0.31366396	0.66185338	0.08690524
ENSG00000(	PPP2R5C	0.04522307	0.35545023	0.08700415
ENSG000001	COL21A1	0.84606364	0.94474488	0.08706579
ENSG000001	EPRS1	0.23008604	0.59855451	0.08712898
ENSG000001	EML2	0.14218506	0.50068215	0.08714368
ENSG00000(	KIF26A	0.79547266	0.92462665	0.08714505
ENSG000001	SLC25A51	0.3344693	0.67664065	0.08717999
ENSG00000(	FUNDC1	0.22953072	0.59783342	0.08726519
ENSG000001	ALKBH3	0.36114865	0.69535997	0.08728924
ENSG000001	ZCCHC7	0.31892502	0.66620166	0.08729357
ENSG000001	DARS1	0.29971192	0.6542516	0.08731274
ENSG000001	KDSR	0.12492003	0.48247538	0.08732296
ENSG000001	PTMA	0.20897534	0.57297071	0.0873295
ENSG000001	LYRM1	0.28636846	0.64387022	0.08735938
ENSG00000(	PDZD4	0.42082622	0.7350227	0.08741385

ENSG000001	ZNF665	0.54418828	0.80893391	0.08741961
ENSG000001	GMPS	0.30243278	0.65591444	0.08748724
ENSG000002	OLIG2	0.57209465	0.82175344	0.08748858
ENSG000001	SUMO3	0.13211456	0.49054292	0.08751393
ENSG00000C	DCT	0.80229259	0.92757225	0.08752988
ENSG000001	TOMM5	0.60549126	0.83945694	0.08758191
ENSG000001	LEPROTL1	0.25702075	0.62086214	0.08761335
ENSG000001	DNMT1	0.15853568	0.52129996	0.08765302
ENSG000001	TRPV3	0.78740972	0.92158665	0.08768351
ENSG000001	PMP22	0.58393869	0.82875175	0.0877198
ENSG000002	NBDY	0.27864027	0.63770725	0.0877378
ENSG000001	TNFRSF11B	0.80636884	0.92964486	0.08777072
ENSG000001	COA4	0.3014498	0.65547284	0.08782517
ENSG000001	PLAAT1	0.56840362	0.82037292	0.08782541
ENSG00000C	ISOC2	0.33202153	0.67489153	0.08783825
ENSG00000C	RRP15	0.48186593	0.77486788	0.08790376
ENSG00000C	DYRK4	0.39305147	0.72063539	0.0879116
ENSG000001	MTUS1	0.55998644	0.81699589	0.08793329
ENSG000001	AP4B1	0.17898777	0.54414332	0.08801165
ENSG000001	INKA1	0.35649126	0.69246335	0.08801722
ENSG000001	H4C11	0.86019142	0.95064852	0.08803174
ENSG000001	GRWD1	0.14249907	0.50068215	0.08804473
ENSG000002	DDX3X	0.34693677	0.68653332	0.08811158
ENSG000001	TANC1	0.74531049	0.90628646	0.08819386
ENSG000001	CDC16	0.21186748	0.57666273	0.08823123
ENSG000001	USP34	0.28895028	0.64680191	0.08823643
ENSG000001	WDR61	0.31476075	0.66270362	0.08824614
ENSG00000C	PREP	0.10170285	0.45081877	0.08825088
ENSG000001	STX1A	0.44808824	0.75114004	0.08825151
ENSG00000C	HSP90AB1	0.14953204	0.50925123	0.08825433
ENSG00000C	MAPKAPK5	0.42561993	0.73817463	0.08826011
ENSG00000C	AK2	0.14698699	0.5054701	0.08826756
ENSG000001	MPEG1	0.18479123	0.5498217	0.0883065
ENSG000001	OCIAD1	0.29535563	0.6512539	0.0883068
ENSG00000C	FAP	0.86692042	0.95276749	0.08831809
ENSG000001	ADAM33	0.85631847	0.9490718	0.08832973
ENSG000001	CALU	0.15863355	0.52140344	0.08837125
ENSG000001	LARP4B	0.04991173	0.36170272	0.08838309
ENSG000001	NGRN	0.23414674	0.60173104	0.08838353
ENSG000001	KATNA1	0.23746054	0.60402516	0.08841056
ENSG000001	C16orf71	0.63325967	0.85444991	0.08841867
ENSG000002	PIGBOS1	0.18463736	0.5498217	0.08842851
ENSG000002	P2RX5-TAX1E	0.49211403	0.7804495	0.08842999

ENSG000001	NUBP1	0.18626622	0.55069275	0.08843721
ENSG000001	MPZL2	0.29673309	0.65217594	0.08845349
ENSG000001	CRIPT	0.35526813	0.6919142	0.08848368
ENSG000001	HIPK3	0.42968959	0.74091639	0.08852243
ENSG000001	ARPC5L	0.23496205	0.60232491	0.08853636
ENSG000001	MGAT2	0.55805568	0.81587881	0.08855994
ENSG000001	HAVCR1	0.72864364	0.89755543	0.08856133
ENSG00000C	CATSPERG	0.23818435	0.60432883	0.08856664
ENSG000002	LCMT1	0.1225862	0.47922766	0.08857936
ENSG000001	DUSP11	0.16213647	0.52484247	0.0886142
ENSG000001	TIGD7	0.57316918	0.82235115	0.08862177
ENSG000001	ELP2	0.31282237	0.66112215	0.08862611
ENSG000001	PDE3A	0.67770102	0.8751149	0.08868356
ENSG000002	TATDN3	0.14796439	0.50706204	0.08870075
ENSG000001	PDHX	0.29906551	0.65423918	0.08870838
ENSG000001	SLU7	0.23781476	0.60402516	0.08870888
ENSG000001	KIF13B	0.12615629	0.48388732	0.08873934
ENSG000001	ORC2	0.34057014	0.68148267	0.08881
ENSG000001	HLCS	0.3604542	0.69534637	0.08884326
ENSG00000C	ZNF506	0.39839228	0.72429687	0.08885322
ENSG000001	C20orf173	0.78866479	0.92204735	0.08887439
ENSG000001	EXOSC10	0.11354582	0.46882009	0.08891917
ENSG000001	ZNF639	0.44270396	0.74861323	0.08894232
ENSG000001	FOSL1	0.55864492	0.81622582	0.08896461
ENSG000001	PPP1R16B	0.1436002	0.50185889	0.08897537
ENSG000001	CHEK2	0.43693022	0.74497757	0.08898368
ENSG000001	SP3	0.41997865	0.73466853	0.08907773
ENSG000001	SLC9C2	0.81683613	0.93280078	0.08909209
ENSG00000C	IL17RB	0.28781779	0.64495772	0.08911072
ENSG000001	CNR2	0.31409597	0.66224858	0.08913369
ENSG000001	RMDN2	0.42950319	0.74091639	0.08914334
ENSG000001	APOBEC3H	0.5732263	0.82236044	0.08917153
ENSG000001	SMAD6	0.74818934	0.90731063	0.08922053
ENSG000001	SPRYD4	0.29162754	0.64928401	0.08929201
ENSG000001	LDLRAD2	0.78493585	0.92041622	0.08930206
ENSG000001	PDGFRL	0.83959906	0.9422567	0.08933546
ENSG000001	ELL2	0.19410505	0.56139717	0.08935831
ENSG00000C	CDIP1	0.5157299	0.79496643	0.08935998
ENSG000002	RAMACL	0.77506068	0.916381	0.08936018
ENSG00000C	UBE2K	0.06069413	0.38451341	0.0894973
ENSG000001	BANF1	0.18478186	0.5498217	0.08952698
ENSG000001	TMEM245	0.31137423	0.65985165	0.08963086
ENSG000001	ZIK1	0.46656436	0.76486809	0.08968916

ENSG000001	SS18L1	0.37031013	0.70302695	0.08970479
ENSG000001	OARD1	0.35184798	0.6905543	0.08972626
ENSG000001	CCDC51	0.23731481	0.60402516	0.08972632
ENSG00000C	RAPGEF3	0.39533256	0.72209218	0.08974802
ENSG000001	RPS15A	0.66690375	0.87009558	0.08975349
ENSG000001	SLC25A17	0.31443644	0.66265276	0.08977637
ENSG000001	TMEM109	0.12785398	0.48588488	0.08986179
ENSG000001	SEPTIN2	0.10486667	0.45514301	0.08986927
ENSG000001	DRAM1	0.18219785	0.54662721	0.08992073
ENSG000001	UBR5	0.17315005	0.53759345	0.08993553
ENSG00000C	LCP2	0.14136266	0.50055578	0.08999874
ENSG00000C	RASAL2	0.58324638	0.82845829	0.09003188
ENSG000001	RARS1	0.33021012	0.67382451	0.09010597
ENSG000001	ZSCAN5A	0.36111817	0.69535997	0.09012228
ENSG000001	HEATR3	0.27003161	0.63142427	0.09012994
ENSG000001	EFCAB5	0.57156201	0.82164253	0.09019319
ENSG000001	FEN1	0.32445712	0.6700783	0.09025525
ENSG000001	TFPI2	0.8545707	0.94818753	0.0902577
ENSG000001	SLC25A26	0.39819993	0.72429687	0.0902969
ENSG000001	MESD	0.17134377	0.5354328	0.09037686
ENSG00000C	CLDN11	0.870929	0.95437279	0.09041652
ENSG000002	RBM12	0.10394519	0.45398919	0.09045026
ENSG00000C	FBXL3	0.35622573	0.69245905	0.09049479
ENSG000001	LONRF1	0.26709782	0.62929976	0.09050544
ENSG000001	BCDIN3D	0.37307381	0.70502787	0.09054813
ENSG00000C	MRPS33	0.56254301	0.8182398	0.09057513
ENSG000001	KIDINS220	0.16479241	0.52807074	0.09072058
ENSG000001	BTN2A1	0.03931667	0.34025028	0.09076645
ENSG000001	MYB	0.37635249	0.70798786	0.09083652
ENSG000001	SLC16A4	0.47769618	0.77199385	0.09093485
ENSG000001	ZC3H12D	0.31254247	0.66090624	0.09097835
ENSG000002	NBPF11	0.26775705	0.63008279	0.09099042
ENSG000001	LRRC34	0.58945652	0.831894	0.09104427
ENSG000001	NGDN	0.45587388	0.75632853	0.09112211
ENSG000001	ERCC3	0.14206123	0.50068215	0.09118588
ENSG000001	SVIP	0.56556724	0.81931223	0.09128435
ENSG000001	ZFAND5	0.20503516	0.56891466	0.09129894
ENSG000001	EFS	0.8107922	0.93129832	0.09131098
ENSG000001	CYP7B1	0.76455626	0.91243616	0.09131561
ENSG000001	NQO1	0.50537224	0.78810305	0.091321
ENSG000001	PTPN9	0.10323732	0.45287405	0.09133058
ENSG000001	SIPA1L3	0.10618781	0.4561473	0.09138697
ENSG00000C	M6PR	0.10712205	0.45794815	0.0914038

ENSG000001	CEP295	0.27963755	0.63854799	0.09150079
ENSG000001	CHCHD1	0.25676978	0.62078186	0.09151775
ENSG000001	IFNAR2	0.12268957	0.4792403	0.09152895
ENSG000001	COX7B	0.47478978	0.76968467	0.09157324
ENSG000001	BIRC6	0.18107649	0.54610144	0.09163387
ENSG000002	PSMB10	0.44742312	0.75099423	0.09164213
ENSG000001	ATP5MG	0.44680466	0.75099423	0.09164748
ENSG000001	GAS2	0.60690519	0.84023871	0.09165797
ENSG000002	POLG2	0.40786304	0.72771726	0.09170399
ENSG000001	STT3B	0.34161175	0.68284501	0.09174758
ENSG000001	ACTL6A	0.33555961	0.67708232	0.09174921
ENSG000001	ARL9	0.87154589	0.95449478	0.09175345
ENSG000001	BFSP1	0.55689616	0.81553703	0.09177364
ENSG000001	CD244	0.26217103	0.62575948	0.09178636
ENSG000001	CUL4B	0.15963861	0.52219103	0.09181025
ENSG00000C	STX7	0.26690441	0.62929976	0.09181851
ENSG000001	RNF169	0.35127508	0.69054758	0.09182226
ENSG000001	C4orf45	0.82464262	0.93591669	0.09182415
ENSG000002	ZNF717	0.40100578	0.72606468	0.09182449
ENSG000002	MPV17L2	0.37568101	0.70743725	0.09184141
ENSG000001	DTD2	0.35984642	0.69491274	0.09189632
ENSG00000C	RAB21	0.12025453	0.47672121	0.09189767
ENSG000001	GARRE1	0.40253168	0.72669629	0.09190338
ENSG000001	COQ10B	0.1656945	0.52885285	0.09192165
ENSG000001	ANKRD26	0.53086174	0.80212192	0.09197244
ENSG00000C	DHX32	0.23045458	0.59855451	0.09201077
ENSG000001	TFB2M	0.40551522	0.72707051	0.09201791
ENSG000001	CD28	0.51698551	0.79538014	0.09202765
ENSG000001	CD79A	0.29139196	0.64906931	0.09203535
ENSG00000C	IPO11	0.30078631	0.65493729	0.0920382
ENSG000001	POPDC2	0.28536885	0.64333814	0.09206107
ENSG000001	PRDX3	0.18435985	0.54968745	0.09209367
ENSG000001	TSPAN31	0.22181917	0.58912768	0.09212527
ENSG000001	N/A	0.72093354	0.89490971	0.09218894
ENSG000001	VPS26A	0.08277658	0.42228867	0.09222978
ENSG000001	SLC5A9	0.43618019	0.74479522	0.09227312
ENSG000001	FAM172A	0.27969165	0.63854799	0.09233654
ENSG000001	GTSF1	0.56189286	0.81801588	0.09233991
ENSG00000C	HECTD1	0.24536252	0.61141177	0.09235196
ENSG000001	SPCS3	0.14604778	0.50430399	0.09235731
ENSG00000C	HGF	0.42095479	0.73502988	0.09237815
ENSG000001	ZMYM5	0.35423318	0.69167785	0.09238736
ENSG000001	RECK	0.4254868	0.73817463	0.09241753

ENSG000000	PPP1R12A	0.18711154	0.55108473	0.09242783
ENSG000001	TM2D3	0.10161232	0.45054047	0.09242895
ENSG000001	IFNA21	0.75921365	0.91125749	0.09243061
ENSG000001	F8	0.31622344	0.66372668	0.09243523
ENSG000001	SREK1	0.36647286	0.69873648	0.0924377
ENSG000002	RNF115	0.07103742	0.40194888	0.09244512
ENSG000001	ACOT13	0.19844462	0.56598606	0.09245799
ENSG000001	CCT4	0.24640586	0.61214306	0.09246918
ENSG000001	TXNL4B	0.07887585	0.41446832	0.09248311
ENSG000001	TRIAP1	0.50103146	0.7853582	0.09248628
ENSG000001	MRPS23	0.33530643	0.67708232	0.09249613
ENSG000001	NR2C2	0.29557293	0.65140093	0.09250505
ENSG000001	MEP1B	0.8493059	0.94621791	0.09257586
ENSG000002	SPIRE2	0.35508963	0.6919142	0.09257727
ENSG000001	TOR1A	0.08133012	0.41882561	0.09259862
ENSG000001	TRPA1	0.79098667	0.92297405	0.09267356
ENSG000002	ANKRD28	0.37606673	0.70771854	0.09269735
ENSG000001	ACVR2A	0.41057378	0.72975628	0.09270205
ENSG000002	ZNF674	0.30329045	0.65599134	0.09270442
ENSG000001	FFAR1	0.83315759	0.93878551	0.09271486
ENSG000001	KAT14	0.28557679	0.64333814	0.09274048
ENSG000000	HEATR6	0.15356641	0.51472848	0.092742
ENSG000001	CCSER2	0.26951622	0.6310094	0.09274612
ENSG000001	FOXR1	0.87127878	0.95449478	0.09274721
ENSG000001	IGFLR1	0.1757787	0.54075763	0.09281907
ENSG000001	ELP4	0.33687323	0.67897352	0.09281916
ENSG000001	RET	0.63238966	0.85418109	0.09282991
ENSG000002	FBXO48	0.2906817	0.648324	0.09283434
ENSG000001	ZNF33B	0.5162621	0.79515933	0.09289436
ENSG000001	LSS	0.43759064	0.74532247	0.0929543
ENSG000001	MAP3K21	0.49013534	0.77995936	0.09297676
ENSG000001	SNAPC5	0.24389935	0.60954191	0.09298013
ENSG000001	TGS1	0.09448492	0.43981122	0.09300942
ENSG000001	GGA2	0.12338986	0.47994951	0.09309367
ENSG000001	PMVK	0.21541157	0.58129262	0.09309708
ENSG000000	TASP1	0.3241443	0.6699145	0.09312259
ENSG000001	SERPINH1	0.35165881	0.69054758	0.09316117
ENSG000000	CROT	0.36290835	0.69664198	0.09321509
ENSG000001	PEX13	0.28427401	0.64253645	0.09327257
ENSG000001	CAPRIN1	0.02511729	0.30075912	0.09327624
ENSG000001	SLMAP	0.26453158	0.6274921	0.09328102
ENSG000001	TANGO6	0.11634354	0.47160917	0.09330284
ENSG000001	NR2C2AP	0.24228812	0.60794812	0.09330586

ENSG000001	ALMS1	0.27289062	0.63378205	0.09343509
ENSG000001	ARL1	0.3415276	0.68283609	0.09349555
ENSG000001	C3orf52	0.56694445	0.8197023	0.09351331
ENSG000001	ZNF681	0.60244159	0.83852406	0.09354971
ENSG000001	LARP1	0.09163452	0.43611772	0.09362467
ENSG000001	PCGF3	0.31923191	0.66648568	0.09363385
ENSG000001	FUOM	0.3717367	0.70389374	0.09363674
ENSG000001	SLC45A2	0.7795163	0.91827559	0.09364406
ENSG000001	AVEN	0.23753643	0.60402516	0.09365337
ENSG000001	ENAH	0.74892417	0.90748372	0.09366408
ENSG000001	ADPGK	0.09024172	0.43395124	0.09366597
ENSG000001	NLRP4	0.82904298	0.93682924	0.09383588
ENSG00000C	RALA	0.31053382	0.65949478	0.0938477
ENSG000001	ZNF700	0.34635388	0.68614821	0.09391969
ENSG000002	SEC22B	0.07663732	0.41092575	0.09395034
ENSG000002	C12orf75	0.45873442	0.75823573	0.0939731
ENSG000001	RAB3GAP2	0.11625596	0.47160917	0.09399587
ENSG000001	ZNF547	0.52412388	0.79870477	0.09401557
ENSG000001	UGT2B15	0.80794396	0.92978846	0.09408907
ENSG000001	NIPA2	0.01124961	0.2451811	0.0940987
ENSG000001	THAP12	0.44147749	0.74778531	0.09416225
ENSG000001	CYREN	0.35400945	0.69162813	0.09419304
ENSG000001	NPC1	0.07635593	0.41079896	0.09420751
ENSG000001	BICD1	0.45809225	0.75759807	0.09420804
ENSG000001	HSPA9	0.07379635	0.40546039	0.09422011
ENSG000001	ZPR1	0.17749859	0.5423686	0.09428564
ENSG000001	UAP1	0.34264455	0.68347906	0.09429894
ENSG000001	KIRREL1	0.82535752	0.93591669	0.0943692
ENSG000001	RBMX	0.31091458	0.65949478	0.09437104
ENSG000001	PTPN1	0.03144361	0.32110055	0.09437901
ENSG000002	N/A	0.82326801	0.93539963	0.09443618
ENSG000001	KCTD18	0.18046869	0.54522392	0.09444361
ENSG000001	RELN	0.81858571	0.9337766	0.09452727
ENSG000001	ADGRE1	0.33377853	0.67615222	0.09454009
ENSG000001	AMER1	0.23754837	0.60402516	0.09454875
ENSG000001	DIABLO	0.26752196	0.6298034	0.09456335
ENSG000001	INTS10	0.17935485	0.54453715	0.09457213
ENSG000001	CPSF2	0.02584694	0.30449397	0.09457321
ENSG00000C	AQR	0.07817884	0.41427772	0.0946108
ENSG000001	LRIG1	0.31573602	0.66329484	0.09463231
ENSG000001	RHOQ	0.11415761	0.46962187	0.09463488
ENSG000001	ABI2	0.48204322	0.77486788	0.09463933
ENSG000001	TTC30B	0.44371655	0.74908353	0.09465834

ENSG000001	ACAT2	0.27127821	0.63286144	0.09468491
ENSG000001	MFSD14A	0.52893907	0.80100575	0.09468722
ENSG000001	LSM3	0.53469028	0.80406779	0.09473071
ENSG000001	DUSP4	0.57722622	0.82460163	0.0947389
ENSG000002	ZNF345	0.54124313	0.80757124	0.0947969
ENSG000001	CAMSAP2	0.47344621	0.76895276	0.09485549
ENSG00000C	MSH2	0.43190491	0.74139339	0.09489904
ENSG000001	N/A	0.78230197	0.9193471	0.09491798
ENSG000001	SNRNP48	0.37	0.70295438	0.09502625
ENSG000001	C1orf109	0.20923912	0.57321	0.09503997
ENSG000001	IL17C	0.80726225	0.92967903	0.09508932
ENSG00000C	HIVEP1	0.25243148	0.61827672	0.0950933
ENSG000001	TUBGCP4	0.32777106	0.67231161	0.09512182
ENSG000001	GRSF1	0.18123035	0.54622749	0.09513933
ENSG000001	ORMDL1	0.3939819	0.72120452	0.09517523
ENSG000001	ATAD5	0.28502019	0.64333814	0.09524029
ENSG000001	DNAJC8	0.19804149	0.56540555	0.09530737
ENSG000001	IRS1	0.35455582	0.69189847	0.09531037
ENSG000001	CSTF2	0.13899364	0.49952186	0.09534376
ENSG000001	SMURF2	0.27458509	0.63464829	0.09535835
ENSG000001	TRIM25	0.29521191	0.6512539	0.09536888
ENSG000001	C8orf76	0.32747806	0.67217574	0.09541465
ENSG000001	ACOT11	0.49262023	0.7804495	0.09545806
ENSG000001	IRF1	0.49068238	0.7800506	0.0954804
ENSG00000C	DHX33	0.17601936	0.54098549	0.09548216
ENSG000001	MYH10	0.37527058	0.70720386	0.09550717
ENSG000001	DOCK11	0.20739165	0.57126603	0.09551172
ENSG000001	TRIM35	0.17524023	0.54013466	0.09554814
ENSG000001	ATF7IP2	0.39733872	0.72359677	0.09556285
ENSG000001	CTNND1	0.12120696	0.47724625	0.0956317
ENSG000001	UCP3	0.23680891	0.60364584	0.0956322
ENSG000001	GTF2E2	0.13858209	0.49898885	0.09566816
ENSG000001	TMEM87A	0.03241087	0.32362351	0.09569304
ENSG000001	AMT	0.4948624	0.78124152	0.09573586
ENSG000001	FUT11	0.18735024	0.55140326	0.09574684
ENSG00000C	PIR	0.85762146	0.94961808	0.09577975
ENSG000001	PLAA	0.04389102	0.35372007	0.09585056
ENSG000001	CTSC	0.03247548	0.32362351	0.09585372
ENSG00000C	GATB	0.34406373	0.68472435	0.09585902
ENSG000001	IL10RA	0.14662153	0.50510677	0.09586763
ENSG000001	PPP2CB	0.07740548	0.41266462	0.09588517
ENSG00000C	GEMIN2	0.53175279	0.8023483	0.09599028
ENSG000001	HERC4	0.22150468	0.58882965	0.0960552

ENSG000001	CREG1	0.17711458	0.54199198	0.09605762
ENSG000001	PNISR	0.29369641	0.65039296	0.09606311
ENSG000001	TSHZ1	0.21987573	0.58702864	0.09614088
ENSG000002	PCDHGC5	0.78421354	0.92016731	0.09616939
ENSG000001	OSGEPL1	0.44517201	0.74940749	0.09617294
ENSG000001	NECAB1	0.57689398	0.82441714	0.09623507
ENSG000001	VASH2	0.49436703	0.78124152	0.09624998
ENSG000001	ICAM2	0.1568027	0.51959295	0.09627277
ENSG000001	MTHFR	0.06478469	0.39055819	0.09630058
ENSG000001	HYOU1	0.1388778	0.49941019	0.09632567
ENSG000001	FMNL3	0.23873986	0.60492348	0.09639256
ENSG000001	SLC16A2	0.74853322	0.90731063	0.09640027
ENSG000001	HSF5	0.63457987	0.85504577	0.09641859
ENSG000001	LGSN	0.75737164	0.91082077	0.09649963
ENSG000002	N/A	0.71008257	0.88997381	0.09654505
ENSG000001	RMI1	0.30084982	0.65498773	0.0965462
ENSG000001	SH2B3	0.04542515	0.35545023	0.09655393
ENSG000002	CEP95	0.38371012	0.7123445	0.09656169
ENSG000001	CLCN3	0.13498331	0.49418805	0.09662089
ENSG00000C	TMEM131	0.08293486	0.42228867	0.09664274
ENSG000001	RAB19	0.4416261	0.747959	0.09664279
ENSG000001	TBC1D15	0.37910578	0.70965485	0.09671321
ENSG000001	TMED6	0.4809999	0.77410996	0.09671783
ENSG000001	ARMH3	0.01280399	0.25583328	0.096755
ENSG000001	ZNF703	0.36104961	0.69535997	0.0967952
ENSG000001	CTSS	0.19922326	0.56600138	0.09680043
ENSG000001	NNT	0.12337067	0.47994951	0.09681191
ENSG000001	METTL7A	0.1476311	0.50621491	0.09683463
ENSG000001	EXOSC3	0.22774451	0.59585684	0.09683783
ENSG000001	SLC2A6	0.22576049	0.59319591	0.09684712
ENSG000001	DNAJC7	0.0812885	0.41882561	0.09695326
ENSG000001	CHAD	0.48236704	0.77508101	0.09696902
ENSG000002	MCTS1	0.33913752	0.68041384	0.09697618
ENSG000001	MLLT3	0.45980307	0.75954697	0.0970579
ENSG000001	C12orf43	0.1087996	0.46040635	0.09706728
ENSG000001	BACH1	0.35203335	0.69071745	0.09710629
ENSG000001	TYW1	0.16944547	0.5338157	0.09711827
ENSG000001	C15orf62	0.43511866	0.74433605	0.09711846
ENSG000001	UNC5CL	0.25565734	0.62030905	0.09711984
ENSG000001	ZNF566	0.39640216	0.72278291	0.09712648
ENSG000001	POFUT1	0.0904282	0.43397794	0.09713872
ENSG000001	CNKSR2	0.49188382	0.7804495	0.09719847
ENSG00000C	PTPRC	0.17021855	0.53437545	0.09720709

ENSG00000(	ALS2	0.13364773	0.49240713	0.09720971
ENSG000002	TEX52	0.28639422	0.64387022	0.09724081
ENSG000001	SNAP25	0.83149381	0.938023	0.09727874
ENSG000001	C11orf24	0.08993606	0.43377006	0.09728174
ENSG000001	AR	0.68919657	0.88074719	0.09728583
ENSG000001	SUGCT	0.68040299	0.87645406	0.09732042
ENSG00000(	IMPG2	0.69821371	0.8835822	0.09732273
ENSG000001	GNAI1	0.56015128	0.81702985	0.09734397
ENSG000002	AGAP6	0.28164919	0.63946601	0.0973608
ENSG000001	FGL1	0.6790071	0.87594571	0.09736694
ENSG000001	TMEM70	0.25961546	0.6232076	0.09737109
ENSG000001	TMEM41A	0.17278494	0.53714513	0.0973759
ENSG000001	MED14	0.06049985	0.38425153	0.09739715
ENSG00000(	ADAM28	0.30107703	0.65533646	0.0973982
ENSG000001	SLC38A1	0.42688209	0.7392501	0.097404
ENSG000002	MIA	0.69476042	0.88222003	0.09741578
ENSG000001	KIAA1328	0.32432119	0.66992678	0.09743441
ENSG000001	ABHD17B	0.13644385	0.49640125	0.0974806
ENSG000001	NGLY1	0.18899437	0.55371734	0.09748787
ENSG000001	EIF2AK3	0.26026632	0.62375562	0.09753523
ENSG000001	CASK	0.3655631	0.69837111	0.09755587
ENSG000001	MED27	0.16359207	0.52709285	0.09756417
ENSG000001	AHCYL2	0.07972004	0.4152849	0.09757899
ENSG000001	ATL1	0.36658593	0.69878561	0.09759344
ENSG000001	THAP1	0.29287276	0.64967561	0.0976689
ENSG000001	ZNF707	0.74528807	0.90628646	0.09767257
ENSG000001	DDX5	0.15298838	0.5140878	0.09768131
ENSG000001	NANP	0.40786074	0.72771726	0.09769543
ENSG000002	CHCHD10	0.71869384	0.89370323	0.09771153
ENSG000001	SMC3	0.25478111	0.6197628	0.09773143
ENSG000001	PTGR2	0.58292448	0.82836905	0.09774111
ENSG000001	SPATS2	0.26429795	0.6274921	0.09778011
ENSG00000(	AGA	0.25467409	0.61971887	0.09779549
ENSG000001	APOBEC3B	0.67396622	0.87391249	0.09783614
ENSG000001	DEGS1	0.01868148	0.27889987	0.09790808
ENSG000001	ZNF43	0.4385653	0.74601932	0.09791811
ENSG00000(	METTL24	0.83148022	0.938023	0.09794177
ENSG000001	GATAD1	0.11429534	0.46971445	0.09801937
ENSG00000(	SEC63	0.16586912	0.52885285	0.09804434
ENSG000001	SF3B1	0.35294939	0.69109369	0.09809136
ENSG000001	ZNF555	0.4475809	0.75099423	0.09818476
ENSG000001	C8orf33	0.10238237	0.45177448	0.09818589
ENSG000001	NXPH3	0.81543579	0.93228896	0.09819149

ENSG000001	KCTD16	0.79770225	0.9258334	0.0982066
ENSG000001	RNASEL	0.14491627	0.50363987	0.09822123
ENSG000001	LPP	0.0790591	0.41476011	0.09822278
ENSG000001	CLC	0.50234603	0.78587461	0.09825636
ENSG000001	LRPPRC	0.27484031	0.63488151	0.09827421
ENSG000001	CCDC92	0.40780183	0.72771726	0.09828664
ENSG000001	TAF7	0.35171697	0.69054758	0.09829916
ENSG000001	C10orf95	0.70511446	0.88728165	0.09834918
ENSG000001	RBM39	0.24616411	0.6120929	0.09836538
ENSG000002	WDR92	0.40893589	0.72869754	0.09837727
ENSG000001	ITGB7	0.18597174	0.55069275	0.09841876
ENSG000002	PLPP6	0.30706567	0.65762361	0.09841955
ENSG00000C	TSPAN15	0.59686136	0.83614954	0.09843902
ENSG000002	JMJD7	0.71791466	0.89332947	0.09845814
ENSG000001	FNBP1L	0.35893131	0.69428726	0.09846414
ENSG000001	ZNF502	0.53398378	0.80362357	0.09846722
ENSG000001	HERPUD2	0.06103776	0.38464604	0.09847512
ENSG000001	CSNK1G1	0.04431263	0.35453012	0.09848563
ENSG000002	PKHD1L1	0.49632583	0.78193527	0.09851848
ENSG000001	RSPRY1	0.06978556	0.4002892	0.09854194
ENSG000001	AMMECR1L	0.12993474	0.48787839	0.09856493
ENSG000001	YTHDF3	0.1817911	0.54661139	0.09860426
ENSG000001	VWDE	0.69459983	0.8821445	0.09862109
ENSG000001	DIS3L	0.23284098	0.60019671	0.09862161
ENSG000001	USP24	0.12069138	0.47724625	0.09862616
ENSG000001	C2orf50	0.79584866	0.9246397	0.09863131
ENSG000001	ZNF776	0.26646208	0.62929726	0.09866481
ENSG000001	SPG11	0.03855738	0.33749663	0.0987304
ENSG00000C	SLC4A4	0.62983259	0.85292675	0.09874573
ENSG00000C	WAPL	0.15085035	0.51146865	0.09875971
ENSG000001	SLC35B2	0.12100079	0.47724625	0.09876045
ENSG000001	HAUS2	0.25830961	0.62209627	0.09877909
ENSG000002	N/A	0.77497574	0.916381	0.09877998
ENSG000002	SLC12A8	0.76847495	0.91358163	0.09879909
ENSG000001	LRIG2	0.2316913	0.599371	0.09880918
ENSG000002	UBE2QL1	0.8389337	0.94195206	0.09881102
ENSG000001	NARS2	0.32885605	0.67284741	0.09883747
ENSG000001	FAM111B	0.62709512	0.85099522	0.09886488
ENSG000001	LAMTOR3	0.42875881	0.74045488	0.09886935
ENSG000001	ZRSR2	0.11929117	0.47602132	0.09889838
ENSG000001	QTRT2	0.13521212	0.49446444	0.09891755
ENSG00000C	WDR76	0.39616026	0.72246676	0.09892911
ENSG000001	MRPL15	0.41735216	0.73321581	0.09894647

ENSG000001	POGLUT1	0.35625277	0.69245905	0.09898877
ENSG000002	ELFN1	0.80708607	0.92967903	0.09904134
ENSG000001	TIMM10B	0.20750893	0.57126603	0.0990461
ENSG000002	PPP1CB	0.33768835	0.67946062	0.09908189
ENSG000001	PIGL	0.40918654	0.7289042	0.09911036
ENSG000001	CCDC88A	0.28541755	0.64333814	0.0991234
ENSG000001	KCTD6	0.20370296	0.56880022	0.09913529
ENSG000002	TEX53	0.83700951	0.94119112	0.09914278
ENSG000001	YPEL1	0.32124149	0.66766831	0.09916934
ENSG000001	GTF2H1	0.14744291	0.50603056	0.09919368
ENSG000001	AMBRA1	0.15472228	0.51606937	0.09922718
ENSG000001	LAMC1	0.4160163	0.73235476	0.09927312
ENSG000001	HSPA12A	0.68067368	0.87652458	0.09934461
ENSG00000(	SRI	0.07884131	0.41446832	0.09936016
ENSG000001	MVB12B	0.21364053	0.57911862	0.09938301
ENSG000001	ZNF749	0.49776288	0.78278241	0.09940448
ENSG000001	IPO9	0.1024471	0.45177448	0.09941354
ENSG000001	MPHOSPH6	0.36871248	0.70168596	0.09942909
ENSG000001	SORD	0.16888019	0.53296205	0.09943279
ENSG000001	ZBTB43	0.07210929	0.40373033	0.0994948
ENSG000001	KCNIP2	0.34713404	0.68653332	0.09950532
ENSG00000(	MNAT1	0.44426291	0.7492207	0.09950989
ENSG00000(	SPTLC1	0.09196767	0.43650367	0.09952163
ENSG00000(	HNRNPH3	0.06024784	0.38377569	0.09954315
ENSG000001	ZNF148	0.25124903	0.61695463	0.09965541
ENSG000001	CCT8	0.16520835	0.52867325	0.09966152
ENSG000001	CYP2R1	0.38718959	0.7165258	0.09973172
ENSG000001	HNRNPH1	0.29735714	0.65274757	0.09975164
ENSG000001	SLITRK5	0.75766277	0.91082077	0.09975214
ENSG000001	MYO1H	0.68983396	0.88074719	0.09978272
ENSG000001	PANK2	0.13116259	0.4894044	0.09979232
ENSG000001	GPD1L	0.28785462	0.64495772	0.09979377
ENSG000001	TNFAIP8	0.32229187	0.66824838	0.09992442
ENSG00000(	MPP5	0.34495519	0.68522517	0.09992633
ENSG000001	HSPD1	0.21348358	0.57911862	0.09994341
ENSG000001	PON2	0.45345769	0.75477115	0.09997863
ENSG000001	RTTN	0.31857383	0.66591607	0.09998439
ENSG000001	ANKRD42	0.26862772	0.63079288	0.10000889
ENSG000001	DECR1	0.03423257	0.32657713	0.10006584
ENSG000001	HILPDA	0.4481246	0.75114004	0.10006683
ENSG000001	RSRP1	0.23868428	0.60492348	0.10009673
ENSG000001	USP53	0.58644737	0.82959975	0.10010372
ENSG000001	MSH3	0.23524027	0.60245998	0.10010415

ENSG000001	NIPAL1	0.64310391	0.8590061	0.1001557
ENSG000001	ARFIP1	0.20500287	0.56891466	0.10018794
ENSG000001	AGAP4	0.29734536	0.65274757	0.10022052
ENSG000001	ELF4	0.21865066	0.58536368	0.10028528
ENSG000001	MCCC2	0.15784698	0.52077869	0.10028729
ENSG000001	PBDC1	0.31690033	0.66422665	0.1002934
ENSG000001	GPR141	0.4393207	0.74621708	0.10032611
ENSG000001	HPS3	0.03850238	0.3372649	0.10032947
ENSG000001	PNMA5	0.72295843	0.89574026	0.10033681
ENSG000001	CALHM5	0.74023152	0.90464694	0.10034967
ENSG000001	EIF4E	0.37365797	0.70572111	0.10036812
ENSG000001	MTMR4	0.08079443	0.41778956	0.10037342
ENSG000001	DARS2	0.07400017	0.40578886	0.10037864
ENSG000001	EIF5A	0.08290354	0.42228867	0.10042337
ENSG000001	METAP1	0.21443201	0.58023513	0.10046437
ENSG000001	DCP2	0.2919894	0.64936748	0.10048386
ENSG000001	SSR3	0.18775937	0.55189155	0.10049063
ENSG000001	PSMB2	0.04930242	0.360767	0.10053169
ENSG000001	FUT10	0.37066859	0.7031833	0.10054675
ENSG000001	CD1A	0.39206758	0.71994497	0.10054924
ENSG000001	SLC23A1	0.35905224	0.69428738	0.10055222
ENSG000001	UBE2W	0.28533999	0.64333814	0.10056384
ENSG000001	DHODH	0.2309354	0.59855451	0.10056888
ENSG000001	CCDC186	0.36591659	0.69846943	0.10058576
ENSG00000C	NCKAP1	0.3896763	0.7188269	0.10070786
ENSG000001	EYS	0.39831163	0.72429687	0.10070804
ENSG000001	PDS5A	0.18615046	0.55069275	0.1007104
ENSG000002	TRIM13	0.20001091	0.56676738	0.10073629
ENSG000001	NME7	0.42901985	0.74066935	0.10073669
ENSG000001	HNRNPDL	0.24681755	0.61277928	0.10078266
ENSG000001	BRCC3	0.22314814	0.59081204	0.10079037
ENSG000001	TPT1	0.61824405	0.8467902	0.10080542
ENSG000002	PPIAL4H	0.71337637	0.89148711	0.10081598
ENSG000001	MFSD6	0.21453757	0.58023513	0.1008304
ENSG000001	THSD1	0.59283413	0.83431304	0.10084283
ENSG000001	KLHL15	0.3340042	0.67622825	0.10094891
ENSG000001	CBLL1	0.06562681	0.39264465	0.10094978
ENSG00000C	TMEM260	0.12684626	0.48465716	0.10098612
ENSG000001	EZH2	0.29919943	0.65423918	0.10099032
ENSG000001	TMEM39A	0.15714051	0.5199629	0.10100676
ENSG000001	BICC1	0.71853052	0.89359529	0.10103201
ENSG000002	SHANK3	0.78749316	0.92161788	0.10104321
ENSG00000C	TNPO1	0.30971494	0.65921628	0.10106286

ENSG000001	TET2	0.40593731	0.72742568	0.10108475
ENSG000001	FYCO1	0.13132799	0.4894044	0.1010899
ENSG000001	COG2	0.07274882	0.40435477	0.10111763
ENSG000001	TOP1	0.17734728	0.54223298	0.10112357
ENSG000001	NMRK1	0.27301245	0.63378205	0.10114413
ENSG000001	ANAPC1	0.1005953	0.44848046	0.10116921
ENSG000001	RSRC1	0.16128611	0.52426078	0.10123952
ENSG000001	USP31	0.20323471	0.56867381	0.10130621
ENSG000001	FKBP3	0.38349097	0.71227898	0.10131355
ENSG000001	HIPK4	0.76903507	0.91375182	0.10131875
ENSG000001	YIPF5	0.07957106	0.4152849	0.10134496
ENSG000001	RASA2	0.31540452	0.66324235	0.10135981
ENSG000001	B3GALT2	0.64908432	0.86252724	0.10139658
ENSG000001	BCL2A1	0.51890994	0.79603449	0.10141052
ENSG000001	LRRC8D	0.10893009	0.4607184	0.10142881
ENSG000001	C12orf54	0.8198023	0.93468999	0.10144182
ENSG000001	ENOX2	0.08345473	0.42260872	0.10149205
ENSG000001	POLR1B	0.11138141	0.46472866	0.10150229
ENSG000001	RBM26	0.20189676	0.56775306	0.10152597
ENSG000001	RNF182	0.76957944	0.91386073	0.10160601
ENSG000001	TRIM2	0.5763674	0.82397739	0.10161795
ENSG000001	TAOK1	0.16231004	0.52515336	0.10167399
ENSG000001	POLA1	0.13804884	0.49898885	0.10167746
ENSG000001	MB21D2	0.32032664	0.66729534	0.10168291
ENSG000001	GDAP1L1	0.80176549	0.92749118	0.10169822
ENSG000001	MTPN	0.13276504	0.49139472	0.10171924
ENSG000001	NSUN4	0.06942413	0.39974794	0.10172815
ENSG000001	RPL7	0.69478696	0.88222003	0.10173032
ENSG000001	PDHA1	0.11846508	0.47516823	0.10173242
ENSG000001	TTN	0.34203711	0.68310736	0.10178224
ENSG000001	PIM2	0.20667331	0.57024809	0.10180911
ENSG000002	CLDN23	0.58829135	0.83097752	0.10181203
ENSG000001	SBSPON	0.4682103	0.76574644	0.10186546
ENSG000001	MEX3D	0.37697316	0.70855293	0.10190507
ENSG000002	RBM8A	0.09315809	0.43758758	0.10191872
ENSG000001	KDM4C	0.10316199	0.45287405	0.10194716
ENSG000001	CDC42BPB	0.15413174	0.51533385	0.10196565
ENSG000001	FMO5	0.36246648	0.69636577	0.10198505
ENSG000001	HSPA4	0.01908056	0.28068185	0.10202064
ENSG000001	FAM120C	0.35653099	0.69246335	0.10202147
ENSG000001	CAMK1	0.2183167	0.58528507	0.10203227
ENSG000001	C2orf76	0.54201354	0.80808176	0.10203888
ENSG000001	PSAT1	0.34787827	0.68740599	0.10205203

ENSG000001	METTL8	0.34524162	0.68522517	0.10210536
ENSG00000C	ECHDC1	0.31553679	0.66324235	0.10216063
ENSG000001	CCT6A	0.12265617	0.4792403	0.10223632
ENSG00000C	CEACAM21	0.78971973	0.92269405	0.10223809
ENSG000001	SELENOT	0.23452718	0.6022486	0.10224479
ENSG000001	RPF2	0.36344353	0.69694698	0.10224946
ENSG000001	PHF5A	0.47372264	0.76895276	0.10228319
ENSG000001	NR2C1	0.42030512	0.73468557	0.10231775
ENSG000001	DYNLT5	0.82090726	0.93538871	0.10238042
ENSG000002	TSN	0.14959385	0.50925123	0.10239363
ENSG000001	BCLAF3	0.18156677	0.54632234	0.10243976
ENSG00000C	DDX18	0.18199059	0.54661139	0.10246481
ENSG000002	DEFB131B	0.82793623	0.93660278	0.10251512
ENSG000001	ACOX2	0.81458794	0.93225797	0.10252798
ENSG00000C	PFN2	0.45566056	0.75632853	0.10260523
ENSG000001	SLC34A3	0.68681079	0.88023892	0.10265772
ENSG000001	WDR12	0.36492178	0.69791169	0.10265971
ENSG000001	LIN28A	0.82936625	0.93682924	0.1026613
ENSG000001	UBXN8	0.48579359	0.7774422	0.10269858
ENSG000001	DDX31	0.18803846	0.55211256	0.102711
ENSG000001	CALN1	0.86330642	0.95188825	0.10271741
ENSG000001	THUMPD3	0.30879344	0.65850788	0.10272033
ENSG00000C	DPP8	0.1321824	0.49068257	0.10277434
ENSG000001	FOXD2	0.48333826	0.77585394	0.10279781
ENSG000002	UPK3BL2	0.67531098	0.87451032	0.10280168
ENSG000001	SLC25A12	0.23163159	0.599371	0.10282072
ENSG00000C	ZC3HC1	0.23095055	0.59855451	0.10284748
ENSG000001	AGGF1	0.23625582	0.60336718	0.10285922
ENSG000001	CD46	0.332401	0.67523724	0.10290499
ENSG000002	USP27X	0.17672474	0.54166428	0.10295837
ENSG000001	KCTD3	0.43513197	0.74433605	0.1029846
ENSG000001	MRPS22	0.30703655	0.65762361	0.10298796
ENSG000001	SDR42E1	0.55531495	0.81451209	0.10303694
ENSG000001	ACVR1	0.1501806	0.50992939	0.10304984
ENSG000001	WNK4	0.78005011	0.918468	0.10307957
ENSG000001	NUP107	0.24435546	0.6102886	0.1030835
ENSG000001	PLK4	0.55989069	0.81699589	0.10310471
ENSG000001	RINT1	0.29524979	0.6512539	0.10312199
ENSG000001	CHCHD7	0.24072522	0.60721888	0.10314006
ENSG00000C	ESF1	0.37951204	0.70965485	0.10315
ENSG000001	ZNF790	0.48487897	0.7769018	0.10315271
ENSG000001	LSM11	0.34666114	0.68634763	0.10315978
ENSG000001	HNRNPF	0.00336579	0.2114129	0.10317479

ENSG000001	CELF4	0.79223238	0.92314956	0.10322817
ENSG000001	YIPF6	0.12588371	0.48382419	0.10323171
ENSG000001	PTGES3	0.10132077	0.44962352	0.10323449
ENSG000001	SLC39A14	0.29304902	0.64976607	0.10323979
ENSG000001	HSCB	0.39559666	0.72212079	0.10324994
ENSG000001	PHKB	0.03594011	0.33062862	0.10326157
ENSG000001	ZBTB49	0.12449076	0.48173414	0.10326985
ENSG000001	POGK	0.03827865	0.33650813	0.10329991
ENSG000002	UBE2V1	0.3636426	0.69694698	0.10330576
ENSG000001	ENTPD5	0.05501639	0.37354464	0.10331691
ENSG000001	CYP2C8	0.77556832	0.916756	0.10336271
ENSG000001	FSIP1	0.68343689	0.87841089	0.1033707
ENSG000001	H4C8	0.37780347	0.70909881	0.10337311
ENSG000001	TCEANC	0.10949366	0.46193514	0.10338312
ENSG000001	ZNF197	0.33954787	0.68098305	0.10340454
ENSG00000(	ZBTB25	0.49233769	0.7804495	0.10344618
ENSG000001	GOSR1	0.04000205	0.34274931	0.10346708
ENSG000001	TMEM147	0.20249957	0.56807486	0.10346817
ENSG000001	MINDY4	0.82483904	0.93591669	0.10362974
ENSG000001	ARHGAP12	0.48530332	0.77718414	0.10363373
ENSG00000(	SLC12A3	0.43891726	0.74609757	0.10363812
ENSG000001	FCF1	0.24176159	0.60728386	0.10365496
ENSG000001	RAB26	0.82240791	0.93539963	0.10366689
ENSG000001	DNAH6	0.58612326	0.82959975	0.10367565
ENSG000002	MS4A4E	0.35633638	0.69246335	0.10373918
ENSG000001	IMMP1L	0.45034261	0.75298249	0.10379478
ENSG00000(	ERMP1	0.24572616	0.61158271	0.10379558
ENSG000001	COA5	0.29712979	0.65264233	0.10379876
ENSG000001	GFER	0.28441039	0.64253645	0.10383227
ENSG00000(	HCCS	0.04537992	0.35545023	0.10384648
ENSG00000(	BAIAP2L1	0.53163763	0.80232066	0.10385723
ENSG00000(	TSPAN17	0.07531613	0.40943237	0.10386146
ENSG000001	NUP155	0.06477259	0.39055819	0.10386815
ENSG000001	PRKAA1	0.32765158	0.67217574	0.10387082
ENSG00000(	UBE3C	0.03628062	0.33244265	0.10389809
ENSG000001	ANO6	0.06862383	0.39796501	0.10396197
ENSG000001	RSKR	0.2856331	0.64333814	0.1039699
ENSG000001	PSMC2	0.30943555	0.65916845	0.10407918
ENSG000001	RPL7L1	0.17341312	0.53805368	0.10408583
ENSG000001	MPP3	0.75660479	0.91082077	0.104095
ENSG000001	ZNF443	0.60277738	0.83858603	0.10409695
ENSG000001	SEPTIN11	0.24158409	0.60721888	0.10409797
ENSG00000(	SYPL1	0.14594362	0.50430399	0.10410935

ENSG000001	CIAO2A	0.35133183	0.69054758	0.1042148
ENSG000001	SNX19	0.01355725	0.25910565	0.10423877
ENSG000001	MDM1	0.35531099	0.69191468	0.10430346
ENSG000001	MED23	0.20250536	0.56807486	0.10430872
ENSG000001	RNGTT	0.15819178	0.52089921	0.10430968
ENSG000001	TGFBR1	0.22083382	0.58818746	0.10437625
ENSG000001	PNN	0.29102477	0.64869008	0.10439631
ENSG000001	PLAGL2	0.06741065	0.39585774	0.10440037
ENSG000001	ZNF530	0.35329636	0.69126085	0.10440174
ENSG00000C	YAF2	0.21988718	0.58702864	0.10442052
ENSG000001	SOGA1	0.19138737	0.55685897	0.1044645
ENSG000002	EDDM13	0.54411716	0.80890224	0.10449186
ENSG000001	ENY2	0.27507757	0.63499184	0.10450616
ENSG000001	EIF2S3B	0.29625979	0.65199276	0.10450718
ENSG000001	FRMD4B	0.10262434	0.45201921	0.10466063
ENSG00000C	DBF4	0.4150393	0.73210136	0.10466921
ENSG000001	CCR4	0.3936923	0.72076675	0.10473567
ENSG000001	NIP7	0.1857503	0.55052683	0.10477423
ENSG000001	ZNF557	0.1658527	0.52885285	0.10482007
ENSG000001	NRROS	0.11079056	0.46375519	0.10482271
ENSG000001	FXR1	0.30044012	0.65493729	0.10482883
ENSG000001	LYRM7	0.37805296	0.70909881	0.1049681
ENSG000001	CNOT2	0.065242	0.39169239	0.1050083
ENSG000001	PTPN11	0.1803979	0.54515554	0.10501398
ENSG000001	C16orf89	0.75045451	0.90811642	0.10502726
ENSG00000C	STARD7	0.05753888	0.3770373	0.10513733
ENSG000001	SIL1	0.19456667	0.56193152	0.10515742
ENSG000002	RNF5	0.80411204	0.92881463	0.10516177
ENSG000001	ZNF57	0.35500164	0.6919142	0.10517637
ENSG00000C	PTCD2	0.44476656	0.74940749	0.10521761
ENSG000001	VMA21	0.22249259	0.5899614	0.10523873
ENSG000001	SMC4	0.15823465	0.52089921	0.10525382
ENSG000001	DENR	0.21966413	0.58681553	0.10526379
ENSG000001	LMO4	0.00796482	0.23002982	0.10529899
ENSG000001	ZNF664	0.23131759	0.5991557	0.10530946
ENSG000001	CNN3	0.52359153	0.79870477	0.1053184
ENSG000001	ACTR3	0.04224588	0.34997262	0.10537509
ENSG000001	PRMT3	0.2403021	0.60709277	0.10538943
ENSG000002	HSFX3	0.81303853	0.93170624	0.10539196
ENSG00000C	DLG1	0.23793465	0.60402516	0.1054752
ENSG000001	ZNF75A	0.28551159	0.64333814	0.10548956
ENSG000001	FZD1	0.12409797	0.4813614	0.10556357
ENSG000001	EPM2AIP1	0.36246106	0.69636577	0.10557015

ENSG000001	MCTP1	0.15930661	0.52203057	0.1055714
ENSG000001	PRKRA	0.18112704	0.54613924	0.10558166
ENSG000001	SETX	0.14820732	0.50736714	0.10560184
ENSG000001	CCNT1	0.14195829	0.50068215	0.1056276
ENSG000002	BBIP1	0.33508847	0.67705718	0.10563113
ENSG00000C	WASHC2A	0.01677313	0.2704224	0.10564812
ENSG000001	EID2B	0.5683823	0.82037292	0.10565224
ENSG000002	VARS1	0.82777736	0.93660278	0.10567056
ENSG000001	ZNF367	0.4330687	0.74263121	0.10571199
ENSG00000C	LSG1	0.05907851	0.38096018	0.10579359
ENSG000001	SYCP3	0.42310856	0.73695551	0.10583293
ENSG00000C	ZDHHC6	0.14169276	0.50055578	0.10586947
ENSG000001	TMEM200A	0.50324106	0.78656158	0.10587125
ENSG000002	N/A	0.08240439	0.42168296	0.10588481
ENSG000001	WEE1	0.29674668	0.65217594	0.10591338
ENSG000001	ATP8A2	0.45876134	0.75823573	0.10596385
ENSG000001	CNOT7	0.19308496	0.55944335	0.10599125
ENSG000001	CBLN3	0.3067959	0.65749363	0.10603182
ENSG000001	BROX	0.17771834	0.5423686	0.10603629
ENSG000001	ANKRD40CL	0.80205293	0.92757225	0.10603637
ENSG000001	ZBTB24	0.21088999	0.57523547	0.10609575
ENSG00000C	STAU2	0.22352782	0.59082227	0.10612085
ENSG000001	ZNF175	0.32449502	0.6700783	0.10615569
ENSG000001	TMEM165	0.18708381	0.55108473	0.10620978
ENSG00000C	MAP3K1	0.29246017	0.64952479	0.10624368
ENSG000001	LIMD1	0.08857722	0.43228984	0.1063512
ENSG000001	C1orf131	0.18359569	0.54871392	0.10635164
ENSG000001	PATJ	0.33541178	0.67708232	0.10641842
ENSG000001	GNA13	0.20794765	0.57145912	0.10641854
ENSG00000C	ITM2A	0.46488992	0.76408722	0.10642951
ENSG000001	ATP5MJ	0.39763716	0.72381553	0.10644093
ENSG000001	SKOR1	0.62560241	0.85038981	0.10645028
ENSG000002	SLC35B4	0.23511026	0.60232491	0.10646491
ENSG000001	GPR18	0.44757243	0.75099423	0.10648133
ENSG000001	RPGRIP1L	0.52203444	0.79776689	0.10650649
ENSG000001	STIM2	0.09000244	0.43377006	0.10653562
ENSG000001	NUDT9	0.19183212	0.55723719	0.10653641
ENSG000001	HNRNPA3	0.05157415	0.36531299	0.10660702
ENSG00000C	SLC24A1	0.18018217	0.54501079	0.10661832
ENSG000001	CLDN7	0.49395034	0.7812211	0.10664298
ENSG000001	HIF1A	0.2043512	0.5688169	0.10664329
ENSG000001	DSCAML1	0.82569667	0.93591669	0.1066674
ENSG000001	MRPL11	0.3633456	0.69694698	0.10667683

ENSG000001	ZC3H8	0.41513353	0.73210851	0.1067578
ENSG000001	ABCD2	0.56755277	0.82023626	0.10677408
ENSG000001	VN1R1	0.66701512	0.87011525	0.10679041
ENSG000001	LSMEM2	0.73077265	0.89863341	0.10684358
ENSG000001	JMJD1C	0.34455631	0.68522517	0.10691834
ENSG000001	AK9	0.30461741	0.65635638	0.10692395
ENSG000001	HBS1L	0.32871602	0.67280499	0.10698982
ENSG000001	CNKSR3	0.71146333	0.8906831	0.10700867
ENSG000001	KCTD10	0.01567625	0.26569439	0.10701637
ENSG000001	EIF4E3	0.09683813	0.44304329	0.10709747
ENSG000001	GAPT	0.24850877	0.61459845	0.10710409
ENSG000001	ZNF789	0.34943492	0.68873328	0.10713793
ENSG000001	TEX9	0.73383918	0.90042672	0.10717684
ENSG000001	ZC3H7A	0.06857732	0.39796501	0.10719987
ENSG000001	PIWIL4	0.27133163	0.63286144	0.10720237
ENSG000002	OOSP1	0.74437387	0.90628646	0.10722023
ENSG00000(	MRPL22	0.53209016	0.80270816	0.10722367
ENSG000001	ZNF337	0.41833374	0.73381176	0.10723068
ENSG000001	PTP4A2	0.0284441	0.31345545	0.10723942
ENSG000001	ZNF577	0.46052062	0.76015269	0.10725119
ENSG000001	SPDYA	0.44037736	0.74724707	0.10726373
ENSG000001	SLC25A33	0.24803463	0.61432011	0.10728341
ENSG000001	RAB8A	0.15713292	0.5199629	0.1073923
ENSG000001	TMEM178A	0.8441908	0.94396799	0.10740463
ENSG00000(	FAM13B	0.13310898	0.49200959	0.10744944
ENSG000001	MCM9	0.01477133	0.26260224	0.10745073
ENSG000001	CIP2A	0.47037018	0.76633358	0.10747891
ENSG000001	SYNJ1	0.22840727	0.59653313	0.1074917
ENSG000001	PTCD3	0.22498792	0.59201197	0.10759591
ENSG000001	GLUD2	0.6171244	0.84630481	0.10765148
ENSG00000(	EFCAB1	0.83147366	0.938023	0.10766023
ENSG000001	ELAC1	0.29214609	0.64944907	0.107668
ENSG000002	SARNP	0.60577691	0.83946052	0.10771143
ENSG000001	RPL23	0.661865	0.86736018	0.10772354
ENSG000001	OLA1	0.34509599	0.68522517	0.10776406
ENSG000001	RGPD2	0.30594491	0.65726747	0.10784545
ENSG000001	HMGA2	0.7678717	0.91357827	0.10787014
ENSG000001	ZNF470	0.4062832	0.72748349	0.10795863
ENSG000001	SIGLEC6	0.61477474	0.84533626	0.10806554
ENSG000001	PPP3CB	0.09043517	0.43397794	0.10807317
ENSG000001	APIP	0.43675977	0.74496191	0.10807583
ENSG000001	MRPL35	0.27827535	0.63759905	0.1080927
ENSG000001	SLC39A11	0.04667741	0.35791197	0.10823921

ENSG000001	DDX59	0.13532181	0.49446444	0.10827104
ENSG000001	MFSD4A	0.38617592	0.71513897	0.10829295
ENSG00000C	TMEM230	0.12854935	0.48678709	0.1082949
ENSG000001	MAK16	0.23531445	0.60255491	0.10832181
ENSG000001	RASAL1	0.77560369	0.916756	0.10838643
ENSG000001	MRPS14	0.33901316	0.68041384	0.10838711
ENSG000001	HPSE	0.1199411	0.47604098	0.10850173
ENSG000001	VPS29	0.25131108	0.61695463	0.10852642
ENSG000001	CDC42SE2	0.06851482	0.39796501	0.10853923
ENSG00000C	MTAP	0.26488625	0.6274921	0.10855135
ENSG000001	CENPP	0.26127673	0.62516213	0.10856175
ENSG000001	AFG3L2	0.09614465	0.44215472	0.10868659
ENSG000001	TMEM87B	0.2387089	0.60492348	0.10872764
ENSG000001	C12orf76	0.10839452	0.45963571	0.10873126
ENSG000001	EPC2	0.1855351	0.55033096	0.10873776
ENSG000002	N/A	0.11544533	0.4710215	0.10873926
ENSG00000C	CD84	0.18542252	0.55033096	0.10876677
ENSG000001	PLCH2	0.58386055	0.82875175	0.10877468
ENSG000002	CHUK	0.2543216	0.619382	0.10877714
ENSG00000C	ERP29	0.13635326	0.49629407	0.10880812
ENSG000001	SMNDC1	0.0515324	0.36531299	0.10884386
ENSG000002	TAS2R40	0.75568032	0.9104345	0.10894077
ENSG000002	ZBED8	0.56703186	0.8197023	0.1089432
ENSG000001	SERPINC1	0.69272236	0.88158966	0.10907169
ENSG000001	SLC17A5	0.0754539	0.40974508	0.1090816
ENSG000001	AKAP9	0.01576848	0.26642326	0.10911324
ENSG000001	TLK1	0.25215307	0.6179006	0.10915813
ENSG00000C	MAP4	0.06398178	0.39023108	0.10916719
ENSG000001	NBPF20	0.1798349	0.54485895	0.10921207
ENSG000001	FARSB	0.24924947	0.61514875	0.1092243
ENSG00000C	MLH1	0.05603918	0.37502119	0.10934228
ENSG000001	PRDM10	0.23218694	0.59955779	0.10937954
ENSG000001	DNAJC6	0.53153041	0.80232066	0.10938982
ENSG000002	C22orf42	0.78388691	0.92000137	0.10939327
ENSG000001	NF1	0.05710333	0.37665007	0.1093937
ENSG000001	LAIR1	0.37305445	0.70502787	0.10941429
ENSG000001	EIF5	0.12714887	0.48500467	0.10949258
ENSG000001	UGCG	0.20097113	0.56770499	0.10950353
ENSG000001	TMEM9B	0.06512288	0.39148468	0.10956901
ENSG000002	SMIM11B	0.81212493	0.93168506	0.10957529
ENSG000001	VSTM1	0.77708061	0.91743314	0.10964333
ENSG00000C	OFD1	0.2557659	0.62038706	0.10982545
ENSG000001	XPOT	0.23498183	0.60232491	0.10987829

ENSG000001	HELLS	0.38749306	0.71667898	0.10994289
ENSG000001	YRDC	0.10676201	0.45726583	0.10995524
ENSG000001	GPR62	0.79577343	0.9246397	0.11000103
ENSG00000C	TMSB10	0.19313335	0.55948371	0.11000705
ENSG000002	IPO7	0.25787901	0.62198226	0.1100443
ENSG000001	KCNT2	0.76604545	0.91290128	0.11009705
ENSG000001	EVL	0.184174	0.54950998	0.11009807
ENSG000001	HYKK	0.56310206	0.81853967	0.11016812
ENSG000001	URI1	0.30352119	0.6561378	0.11018003
ENSG000001	BAG4	0.20910851	0.57310021	0.11031325
ENSG000002	EML6	0.49783241	0.78278241	0.11032424
ENSG00000C	DPM1	0.39663206	0.72299866	0.11032762
ENSG000001	DIP2C	0.43032417	0.7410474	0.11039492
ENSG000001	HERC2	0.34279399	0.68359637	0.11042178
ENSG00000C	SLC25A13	0.07782294	0.41335649	0.11042365
ENSG000001	PDCL	0.05350544	0.37047669	0.11043802
ENSG000001	KIAA1671	0.35709798	0.69298434	0.11043899
ENSG000001	PLEKHA3	0.13959261	0.49975817	0.11044674
ENSG000001	PRKD3	0.19435348	0.56191549	0.11046062
ENSG000002	TEDDM1	0.6546948	0.86439091	0.11050455
ENSG000001	NEMP2	0.33137187	0.67444298	0.11055207
ENSG000001	RWDD1	0.42201848	0.73601655	0.11055691
ENSG000001	ZNF778	0.17959249	0.54474936	0.11058828
ENSG000001	PRIM2	0.19518732	0.56280385	0.11073039
ENSG00000C	GOLGA5	0.00221324	0.20535094	0.11074387
ENSG000001	ZC3H14	0.16839175	0.53253292	0.11074872
ENSG000001	SFPQ	0.06451297	0.39055819	0.11078995
ENSG000001	MRPL18	0.12878196	0.48697483	0.11083966
ENSG000001	YWHAQ	0.06520586	0.39169239	0.11084898
ENSG000001	CARD8	0.13134239	0.4894044	0.11086115
ENSG000001	HACL1	0.21116438	0.57528121	0.11086696
ENSG000001	ABRAXAS2	0.04307368	0.35215875	0.11087545
ENSG000001	TRIM4	0.08086661	0.41789729	0.11089774
ENSG000001	LMOD3	0.63486865	0.85504577	0.1109202
ENSG000001	YME1L1	0.00799086	0.23002982	0.11092401
ENSG000001	DR1	0.07884256	0.41446832	0.1109301
ENSG000001	PDE4B	0.21569056	0.58175542	0.11097012
ENSG000001	SRSF6	0.2405656	0.60721888	0.11099654
ENSG000001	BEX4	0.17942267	0.54453941	0.11100452
ENSG000001	ABI1	0.04461054	0.35527495	0.11100826
ENSG000001	PKDCC	0.79237204	0.92314956	0.11105473
ENSG000001	GLO1	0.24188918	0.60741643	0.11107096
ENSG000001	AGO3	0.26288703	0.6264304	0.11118405

ENSG000001	FAM227B	0.40150814	0.72614031	0.11123855
ENSG000001	GSTA4	0.49643354	0.78193527	0.11125424
ENSG000001	XRCC4	0.49264042	0.7804495	0.11138144
ENSG000002	PKD1L3	0.72132548	0.89490971	0.11141721
ENSG00000C	C5orf22	0.083102	0.42250308	0.11144792
ENSG00000C	STEEP1	0.14903936	0.50903493	0.11151978
ENSG000001	ZBTB8OS	0.15759736	0.52077869	0.11152015
ENSG000002	FAM200A	0.30011283	0.6545241	0.11152737
ENSG000001	DNA2	0.4714697	0.76694135	0.11156298
ENSG000001	ITSN2	0.17077753	0.53457173	0.11158997
ENSG000001	SCP2	0.15150274	0.51259637	0.11166693
ENSG00000C	DOCK9	0.33627863	0.67811486	0.11173999
ENSG00000C	ATP11B	0.2095213	0.5736945	0.11183631
ENSG000001	AASDH	0.27448897	0.63464829	0.11187287
ENSG000001	DCTD	0.09984683	0.447493	0.11188094
ENSG00000C	TBC1D23	0.26332836	0.62687307	0.11189362
ENSG00000C	C20orf194	0.13940699	0.49975817	0.11193833
ENSG000002	FDXACB1	0.41629692	0.73241014	0.1119449
ENSG000002	ANG	0.51072685	0.79143652	0.11196185
ENSG000002	TBC1D3I	0.79446428	0.92447045	0.11196218
ENSG000001	LINS1	0.205631	0.56937764	0.11197065
ENSG000001	BBS10	0.43812079	0.74567791	0.11209185
ENSG000001	PLEKHN1	0.34393504	0.68470736	0.11209766
ENSG000001	SPOPL	0.25597534	0.62056088	0.11213654
ENSG000001	INPP1	0.07753331	0.41275686	0.1121491
ENSG000001	CCZ1B	0.14343542	0.50147706	0.11217974
ENSG000001	ITGB1BP1	0.09799795	0.44463533	0.11218151
ENSG00000C	NDUFAB1	0.21705454	0.58371881	0.11218779
ENSG000001	PLRG1	0.29050587	0.648324	0.11223857
ENSG000001	TAF9B	0.33468663	0.67675278	0.11227309
ENSG00000C	GLRX2	0.27044354	0.63178344	0.11229123
ENSG000001	MTHFD1L	0.3312578	0.67444298	0.11229482
ENSG000001	ADPRM	0.43876791	0.7460934	0.11229748
ENSG000001	UBE2Z	0.0101949	0.24174018	0.11230072
ENSG000002	ZNF432	0.30502876	0.65643145	0.11232182
ENSG000001	EXT2	0.02220042	0.29306353	0.11239646
ENSG000001	PRPF18	0.31490476	0.662749	0.11240475
ENSG000001	GTF2F2	0.22433294	0.59169817	0.11242704
ENSG000001	IPMK	0.50367403	0.78673932	0.11244283
ENSG000001	FBXW8	0.17568434	0.54061531	0.11245438
ENSG000001	SRP54	0.03449501	0.32831054	0.11248869
ENSG000001	STS	0.18966191	0.55424774	0.11259254
ENSG000001	NEU3	0.10619634	0.4561473	0.11260591

ENSG000001	COX7C	0.55388486	0.81396882	0.11261459
ENSG000001	GOLGA8A	0.49087699	0.7800506	0.11264332
ENSG000001	ZNF714	0.38837756	0.71712216	0.11266142
ENSG000001	ATP6V0A2	0.10221445	0.45160695	0.11273201
ENSG000001	C1orf52	0.22186587	0.58912768	0.11277895
ENSG000001	RLIM	0.09602225	0.44204901	0.1128009
ENSG000001	ZSCAN9	0.20249795	0.56807486	0.11280102
ENSG00000(	LARS2	0.0222316	0.29312398	0.11291658
ENSG000001	SMAD1	0.24818369	0.6144738	0.11293149
ENSG00000(	CRY1	0.32348178	0.66951862	0.11295573
ENSG000001	UNC50	0.13609519	0.49591061	0.11296879
ENSG00000(	SIDT1	0.24203781	0.60760172	0.11311812
ENSG000001	EARS2	0.0796769	0.4152849	0.11311889
ENSG000001	TIMM17A	0.21094625	0.57523547	0.11312225
ENSG00000(	ZNF638	0.14418941	0.50250064	0.11314385
ENSG00000(	DUSP12	0.26885316	0.63079288	0.11314648
ENSG000001	ATXN10	0.16562186	0.52885285	0.11317265
ENSG000001	DDX17	0.25486289	0.6197628	0.11319277
ENSG000001	WASH6P	0.73109951	0.89889919	0.11320527
ENSG000001	ARIH2	0.07125398	0.40241675	0.11321333
ENSG00000(	DDHD2	0.30768031	0.65762361	0.1132753
ENSG000001	RNF7	0.2224313	0.58995895	0.11329211
ENSG000001	MAT2A	0.10029725	0.4480999	0.11330719
ENSG000001	MAPK8IP1	0.434032	0.74353384	0.11333507
ENSG000001	HDX	0.40499388	0.72672584	0.11335446
ENSG000001	MED4	0.10559759	0.4561473	0.11338832
ENSG000001	RDH10	0.32134918	0.66774337	0.11339294
ENSG000001	AFF3	0.25860976	0.6223087	0.113407
ENSG00000(	OXCT1	0.34025939	0.68135285	0.11340728
ENSG000001	ITGB3BP	0.55374767	0.8139043	0.11341508
ENSG000001	PLS1	0.40257641	0.72669629	0.11348933
ENSG000002	ZNF234	0.31510534	0.6630587	0.11350727
ENSG000001	TMA16	0.3812942	0.71047885	0.11350995
ENSG000001	FBXO8	0.20132018	0.56773237	0.11354402
ENSG000001	MAST1	0.4780685	0.77199385	0.11370479
ENSG00000(	IL20RA	0.75601094	0.91049915	0.11372366
ENSG000001	ZFP82	0.25820926	0.62203913	0.11372775
ENSG000001	TRAF6	0.01645682	0.27000801	0.11374389
ENSG000001	PPP2R1B	0.05232685	0.36780562	0.11375237
ENSG000001	SCLY	0.20781825	0.57145912	0.11375419
ENSG000001	LCMT2	0.17418737	0.53927128	0.11376807
ENSG000001	TMEM18	0.25848264	0.6223087	0.11376902
ENSG000001	LYSMD4	0.23675732	0.60364584	0.11377134

ENSG000001	CENPS	0.6001733	0.83754165	0.11377853
ENSG00000(	ABCB4	0.40382951	0.72672584	0.11379319
ENSG000002	SPRN	0.3044188	0.65635638	0.11388823
ENSG000001	LRRC32	0.62696731	0.85099522	0.11392544
ENSG000001	EML4	0.16394066	0.52712713	0.1139328
ENSG000001	RBBP6	0.03134287	0.32055029	0.11396935
ENSG000001	CASZ1	0.24366302	0.60937379	0.11401419
ENSG000001	RAB14	0.02471664	0.29927225	0.11402877
ENSG000001	SPATA24	0.45753308	0.75743527	0.11408883
ENSG000001	PHIP	0.06829981	0.39743754	0.11409381
ENSG000001	TENT2	0.10866626	0.46040635	0.11412341
ENSG00000(	ATP2C1	0.10914791	0.46127919	0.11412937
ENSG000001	EOMES	0.27289506	0.63378205	0.11417776
ENSG000001	C4orf3	0.0513227	0.36501389	0.11419848
ENSG000001	STPG2	0.82629741	0.93615843	0.1142353
ENSG000001	BUB3	0.25325841	0.61912837	0.11424246
ENSG000001	RHOH	0.22612466	0.59329927	0.1143344
ENSG000001	ARHGEF6	0.00078655	0.18856694	0.1144188
ENSG000001	ZNFX1	0.4493662	0.75245698	0.11442849
ENSG00000(	RNF14	0.14921524	0.5092072	0.11443757
ENSG000001	GNPAT	0.0783548	0.41427772	0.1144549
ENSG000001	PGM2L1	0.22901592	0.59754644	0.11447762
ENSG000001	PLA2G2C	0.75327636	0.90916139	0.11448248
ENSG000001	UBL3	0.19799924	0.56540555	0.11450012
ENSG000002	FAM133B	0.24148196	0.60721888	0.11455678
ENSG000001	SBDS	0.27881729	0.63783746	0.1145906
ENSG000001	TAPT1	0.16710332	0.53159417	0.11459927
ENSG000001	CAPS2	0.47339817	0.76895276	0.11459953
ENSG000001	KRR1	0.38023332	0.70987972	0.11462692
ENSG000001	SLF1	0.31858324	0.66591607	0.11466806
ENSG000001	PRNP	0.20582884	0.56956674	0.11475724
ENSG000002	EXOSC6	0.26807481	0.63028246	0.11480695
ENSG000001	DNAJC2	0.21704842	0.58371881	0.11485687
ENSG000001	DFFB	0.21469808	0.58023513	0.11485817
ENSG000001	TGM2	0.57961987	0.82610777	0.11486121
ENSG000001	WRN	0.32699696	0.67217574	0.11486419
ENSG000001	SGMS1	0.12260342	0.47922766	0.1148642
ENSG00000(	PRDM11	0.22708941	0.59462195	0.11487315
ENSG000001	CATSPER2	0.38056593	0.70987972	0.11490271
ENSG000001	KLHL3	0.41555322	0.73221244	0.11491216
ENSG000001	DENND2C	0.19911587	0.56600138	0.1149479
ENSG000001	DDX19B	0.0530055	0.36981204	0.11500643
ENSG00000(	FAR2	0.12810902	0.48600611	0.11501253

ENSG00000(	SLC9A7	0.16332986	0.52691971	0.1150156
ENSG00000(	NSUN2	0.04464505	0.35527495	0.11505534
ENSG000001	ZNF792	0.1223941	0.47887061	0.11506018
ENSG000001	GLDN	0.82118427	0.93539963	0.11506949
ENSG000001	QSOX2	0.23291355	0.60028846	0.11507827
ENSG000001	PAIP2B	0.40236311	0.72669629	0.115086
ENSG000001	POLR1F	0.31562519	0.66324235	0.1150877
ENSG000001	PPP2R5E	0.06431894	0.39043354	0.11512712
ENSG000002	PCDHGA11	0.76381886	0.91232358	0.11519144
ENSG000001	COPS4	0.3297871	0.67346913	0.11519534
ENSG000001	CDCP1	0.4120021	0.73041489	0.1152124
ENSG000001	CDC40	0.13498107	0.49418805	0.1152183
ENSG00000(	RSBN1	0.085328	0.42551314	0.11524153
ENSG000001	CCDC90B	0.27653961	0.6360577	0.11526351
ENSG000001	C12orf66	0.24937392	0.61525038	0.11531732
ENSG000001	STX12	0.0167602	0.2704224	0.11538184
ENSG000001	SLC35F2	0.32985617	0.67347871	0.11541972
ENSG000001	KIAA0319L	0.33439628	0.67664065	0.1154239
ENSG000001	SRSF11	0.31793157	0.66556648	0.11543135
ENSG000001	MRPL16	0.03293344	0.32362351	0.1154317
ENSG000001	LARP7	0.2864245	0.64387022	0.11548527
ENSG000001	LONRF2	0.7911871	0.92298177	0.11548934
ENSG000001	FAM20B	0.07013826	0.40059904	0.11551282
ENSG000001	INTS7	0.07607088	0.41035311	0.11556895
ENSG000001	GAPDHS	0.79037439	0.92280247	0.1156625
ENSG000001	SLC36A4	0.25560196	0.62030905	0.11570105
ENSG000001	GIN1	0.45657703	0.75693702	0.11572051
ENSG000001	DBI	0.39423943	0.72144863	0.11573142
ENSG000001	KLF4	0.11814086	0.4748744	0.11575641
ENSG000001	MPHOSPH10	0.28432646	0.64253645	0.11576461
ENSG000001	MED10	0.15658999	0.51931203	0.11583791
ENSG000001	ZC3H7B	0.05597726	0.37502119	0.1158783
ENSG000002	C1QTNF3-AM	0.80726118	0.92967903	0.11590008
ENSG000001	FABP2	0.77902571	0.91819399	0.11595093
ENSG000001	ASAP2	0.51906985	0.79608361	0.11595743
ENSG00000(	TFB1M	0.21268226	0.57808354	0.11599358
ENSG000001	PSMG2	0.06584046	0.3931782	0.11606308
ENSG000001	SYT4	0.80155068	0.92746876	0.11608283
ENSG00000(	WDR3	0.13150329	0.4894044	0.11612158
ENSG000001	RCC2	0.02207208	0.29231997	0.11618298
ENSG000001	KIF19	0.49702632	0.78237677	0.11618689
ENSG000001	FAM210A	0.20540463	0.56904198	0.11620925
ENSG000001	ACP2	0.21192275	0.57666997	0.11625405

ENSG000001	SPRYD7	0.15535042	0.51717788	0.11625934
ENSG000001	TULP4	0.1309535	0.4894044	0.1162637
ENSG00000C	MSL3	0.05981558	0.38277429	0.11626519
ENSG000001	BIVM	0.35557083	0.69207883	0.11627109
ENSG000001	PDK1	0.26360971	0.62687307	0.11629023
ENSG000001	ADH5	0.31013221	0.65948451	0.11637159
ENSG000001	TCEA1	0.13207921	0.49054292	0.11644273
ENSG00000C	RHOBTB2	0.02648005	0.30699826	0.11647105
ENSG000001	GTPBP4	0.12779492	0.48588488	0.11648162
ENSG000002	OOSP4B	0.69674174	0.88285035	0.11648856
ENSG000002	SLC25A53	0.31245108	0.66090624	0.11656341
ENSG000001	TOR3A	0.037558	0.33471496	0.11657902
ENSG000001	TAF1	0.21355548	0.57911862	0.11657993
ENSG000001	ZBTB39	0.26170031	0.62545731	0.11659185
ENSG000001	LAX1	0.30039362	0.65493729	0.11661015
ENSG000001	CYB5B	0.05075853	0.36306882	0.11666503
ENSG000001	RBCK1	0.38939202	0.71855418	0.1167185
ENSG00000C	CRYBG3	0.2941706	0.65044158	0.11673383
ENSG000001	MAP3K8	0.06094322	0.38464604	0.11676795
ENSG000001	ZNF605	0.42413506	0.73732623	0.11677955
ENSG000001	UBE3A	0.15724049	0.52008837	0.11679127
ENSG00000C	NDUFAF7	0.28027175	0.6389512	0.11686727
ENSG00000C	MYO9A	0.15060459	0.51094166	0.1169323
ENSG000001	NAGK	0.14550449	0.50417338	0.1169531
ENSG000001	ARF6	0.0011618	0.18856694	0.11704518
ENSG000001	GLUD1	0.03271206	0.32362351	0.11705221
ENSG000001	SNX20	0.06474867	0.39055819	0.11708234
ENSG000002	MBD5	0.02894461	0.31345545	0.11715699
ENSG000001	GNG3	0.73305543	0.89987306	0.11716782
ENSG000001	NIBAN3	0.15114187	0.51190876	0.11719581
ENSG00000C	RNH1	0.78358592	0.91983554	0.11722197
ENSG000001	CAST	0.01144874	0.24590889	0.11723696
ENSG000001	B3GNT7	0.19982671	0.56654204	0.11725069
ENSG00000C	THSD7A	0.81206148	0.93168506	0.11725152
ENSG000002	SHLD3	0.47756523	0.77191738	0.11725199
ENSG000001	GRAMD2B	0.45495818	0.7559601	0.11726367
ENSG000001	RPAIN	0.28743159	0.64495772	0.11726404
ENSG000001	ATG4A	0.20416337	0.5688169	0.11727377
ENSG000001	ACE	0.43438463	0.7438413	0.1172963
ENSG000001	TNNC2	0.68628533	0.87998223	0.11731278
ENSG000001	SPDYE2B	0.49251463	0.7804495	0.11733316
ENSG000001	LARP1B	0.29618738	0.65199276	0.11738274
ENSG000001	RARS2	0.2401108	0.60689277	0.1174142

ENSG000001	CXorf58	0.65065547	0.86327891	0.11746478
ENSG000001	GPLD1	0.47438627	0.76933778	0.11746597
ENSG000001	ACTR8	0.03469184	0.32831054	0.11750712
ENSG000002	ERVFRD-1	0.79088521	0.92297405	0.11752405
ENSG000001	EBAG9	0.29766041	0.65292644	0.11755315
ENSG000001	LLPH	0.12405616	0.4813142	0.11763363
ENSG000001	IL1RL1	0.55986511	0.81699589	0.11765014
ENSG000001	TSC22D2	0.13319368	0.49202523	0.1176675
ENSG00000(	CDC42	0.27007111	0.63142427	0.11767454
ENSG000001	CMC1	0.27564812	0.63512113	0.11768875
ENSG00000(	RFC1	0.04070018	0.34551949	0.1177044
ENSG000001	GTF3C6	0.1869193	0.55108473	0.11772077
ENSG000001	MTCH2	0.04389641	0.35372007	0.11774977
ENSG000001	SPTSSA	0.22454198	0.59186495	0.11776168
ENSG000001	CLEC4G	0.54976507	0.81217374	0.11780263
ENSG000001	MAGEH1	0.30772647	0.65762361	0.11784312
ENSG000001	PNMA8A	0.63245515	0.8541985	0.11786785
ENSG000001	CNIH4	0.05630253	0.37502119	0.1178818
ENSG000001	COPB1	0.02018545	0.28574648	0.11797359
ENSG000001	MTDH	0.06337827	0.38950527	0.11802258
ENSG000001	KPNB1	0.17008396	0.53437545	0.11802866
ENSG000001	TAX1BP1	0.30836509	0.65811303	0.11805748
ENSG000001	ZNF568	0.29195876	0.64936748	0.11805978
ENSG000001	MIS18A	0.32169252	0.66822528	0.11809339
ENSG000001	NDUFA12	0.19636007	0.5638811	0.11809692
ENSG000001	SRPRB	0.20587023	0.56956674	0.11819198
ENSG000001	TMEM67	0.57993873	0.82610777	0.11819604
ENSG00000(	PDCD2	0.18663986	0.55099482	0.11821599
ENSG000002	ZBED5	0.3618457	0.69588937	0.11823171
ENSG000001	PHF10	0.3015167	0.65547284	0.11828032
ENSG000001	TOR1AIP1	0.0324773	0.32362351	0.11828406
ENSG00000(	KIF16B	0.09248319	0.43746768	0.11831364
ENSG000001	SUPT7L	0.19086128	0.55647457	0.11831425
ENSG000001	PRMT6	0.21439339	0.58023513	0.11835217
ENSG000001	HTATSF1	0.15248679	0.5133091	0.11835358
ENSG000001	IL2RA	0.18204276	0.54662721	0.11836836
ENSG000001	HES7	0.79331805	0.92377404	0.11839476
ENSG000001	NEK4	0.23624947	0.60336718	0.11846354
ENSG00000(	REXO2	0.27868094	0.63770725	0.11850962
ENSG000001	RNF32	0.39554318	0.72212079	0.11852039
ENSG000001	TMEM167A	0.22049806	0.5880238	0.11856622
ENSG000002	N/A	0.78871622	0.92204735	0.11871213
ENSG000001	SYTL2	0.30263233	0.65591444	0.11877465

ENSG00000(	SNAPC1	0.43005713	0.74091639	0.11884397
ENSG00000(	ABCB1	0.3524119	0.69082603	0.11886577
ENSG00000(	SYT1	0.77409857	0.91603002	0.11904253
ENSG000001	NUP93	0.01991375	0.28363903	0.11904354
ENSG000002	FAM216A	0.32855928	0.67264721	0.11905554
ENSG000001	COPS8	0.29619138	0.65199276	0.11906769
ENSG000001	GLCCI1	0.2149414	0.58050623	0.11907169
ENSG000002	BAG6	0.53312893	0.80294166	0.11908192
ENSG000001	MTO1	0.11395647	0.46950297	0.11911211
ENSG00000(	TARBP1	0.36603291	0.69846943	0.11911756
ENSG000001	GPR89A	0.40788018	0.72771726	0.11913642
ENSG000001	ATL2	0.33271821	0.67523724	0.11931096
ENSG00000(	ATXN3	0.13258063	0.49124539	0.11934834
ENSG00000(	ALG6	0.24885797	0.61478169	0.11941653
ENSG000001	ARHGAP18	0.15929844	0.52203057	0.11942781
ENSG000001	WDR77	0.06889865	0.39825823	0.11948761
ENSG000001	NUP43	0.17070483	0.53457173	0.11949123
ENSG000001	TGIF2	0.03808585	0.33602196	0.11956698
ENSG000001	PSPH	0.33256123	0.67523724	0.11959431
ENSG000001	RASGEF1C	0.76182518	0.91151487	0.11959957
ENSG000002	ZNF880	0.24468156	0.61065086	0.11973207
ENSG000001	PCID2	0.28940662	0.64727721	0.11979327
ENSG000001	ZNF583	0.34997102	0.68928669	0.11979972
ENSG000001	NOL11	0.1429712	0.5010464	0.11982073
ENSG000001	RWDD2B	0.25952029	0.6232076	0.11984635
ENSG000001	TICAM1	0.16072207	0.52370945	0.11985354
ENSG000001	ZNF227	0.11387208	0.46939348	0.11986308
ENSG00000(	MYO3A	0.75579603	0.9104345	0.11986322
ENSG000001	GNE	0.02743389	0.3110643	0.11989366
ENSG000001	P2RY2	0.32616842	0.67170284	0.11990876
ENSG000001	RFXAP	0.25494886	0.61979408	0.11993899
ENSG000001	ZNF721	0.43936507	0.74621708	0.11995146
ENSG000001	SUCLG2	0.16807972	0.53240547	0.119992
ENSG00000(	ARID4B	0.04987891	0.36170272	0.11999477
ENSG000001	UBA2	0.21516079	0.58090552	0.12003092
ENSG000001	ABI3	0.16160913	0.52427832	0.12003466
ENSG000001	RFTN1	0.04666391	0.35791197	0.1200771
ENSG000001	NOD2	0.19967735	0.56647197	0.1200947
ENSG000001	SLC16A14	0.56425397	0.81882132	0.12009648
ENSG000001	ADGRG5	0.25961256	0.6232076	0.12010573
ENSG000001	SPART	0.04393549	0.35385593	0.12012173
ENSG000001	BCCIP	0.26795132	0.63017463	0.12024185
ENSG00000(	THOC1	0.25811437	0.62198226	0.1202663

ENSG000001	FAM124B	0.29650271	0.65199276	0.12027223
ENSG00000C	FSTL4	0.64390268	0.85940463	0.1202966
ENSG000001	ATL3	0.04620432	0.35738124	0.12031472
ENSG000001	GNL2	0.11538263	0.4710215	0.12031963
ENSG00000C	MLLT10	0.18011066	0.54501079	0.12033384
ENSG000001	TPP2	0.20602231	0.56956674	0.12034433
ENSG000001	CCIN	0.7277024	0.8971681	0.12034974
ENSG000001	ZDHHC23	0.306067	0.65735608	0.12039031
ENSG000001	RLN2	0.77342317	0.91571183	0.12041914
ENSG000001	MRPS31	0.35251996	0.69087104	0.12043004
ENSG000001	NSL1	0.08128354	0.41882561	0.12043729
ENSG000001	NCOA2	0.20105478	0.56773237	0.12050167
ENSG000001	CAPZA1	0.20046789	0.56724227	0.12052353
ENSG000001	C17orf75	0.28618397	0.64387022	0.12054144
ENSG000001	ZNF799	0.4309726	0.74126857	0.12054925
ENSG000001	DCLRE1A	0.34548466	0.68530904	0.12055357
ENSG000001	PPIG	0.36090464	0.69535997	0.12060039
ENSG000001	TMTC2	0.14603738	0.50430399	0.12063936
ENSG00000C	PDE8A	0.09141287	0.43578121	0.12066241
ENSG000001	CMPK1	0.3327019	0.67523724	0.12075809
ENSG000001	NUP88	0.16743091	0.53173243	0.120764
ENSG000002	H4C12	0.52709909	0.80038735	0.12077033
ENSG00000C	KIF2A	0.25760698	0.6217875	0.1207808
ENSG000001	SIGLEC8	0.51584445	0.79496643	0.12079194
ENSG000001	CDK6	0.1977889	0.56540555	0.12092816
ENSG000002	TUG1	0.23877284	0.60492348	0.12092884
ENSG000002	NEU1	0.75235551	0.908573	0.12096772
ENSG000001	FAM149B1	0.15995433	0.5226763	0.120993
ENSG000001	CDKAL1	0.08342004	0.42260872	0.12110654
ENSG00000C	APBA2	0.65296998	0.86379938	0.1211222
ENSG00000C	TRIO	0.14311352	0.50134504	0.12115683
ENSG000002	RPS27AP5	0.77768736	0.91771115	0.12117131
ENSG000001	ARHGAP32	0.18940702	0.55422631	0.12121995
ENSG00000C	CRLS1	0.2415851	0.60721888	0.12126468
ENSG000001	LRRC8C	0.17181316	0.53586381	0.12126484
ENSG000002	PARG	0.04264614	0.35185871	0.12126667
ENSG000001	FAM221A	0.31944595	0.66652314	0.12130359
ENSG000001	RNF43	0.47437783	0.76933778	0.12132313
ENSG00000C	TTC27	0.18959716	0.55424774	0.12141969
ENSG00000C	MTREX	0.23191006	0.599371	0.12143553
ENSG00000C	DPYSL2	0.06605966	0.39345499	0.1214392
ENSG000002	KANTR	0.44941398	0.75245698	0.12146827
ENSG000002	ZNF468	0.23754386	0.60402516	0.12148321

ENSG000002	ZNF285	0.48605404	0.77752459	0.12153197
ENSG00000(	DIP2B	0.16071216	0.52370945	0.12163316
ENSG000002	C4orf47	0.83198984	0.93831391	0.12164554
ENSG000001	CERCAM	0.3080082	0.65762361	0.1216607
ENSG000001	MBLAC2	0.54348701	0.8084284	0.12178663
ENSG000001	SASH1	0.31735035	0.66470815	0.12178724
ENSG000001	MAGEF1	0.35857564	0.69382497	0.12182125
ENSG000001	SYCP2L	0.62869893	0.85212646	0.12183099
ENSG000001	MLANA	0.34209774	0.68310736	0.12183122
ENSG00000(	SS18L2	0.285124	0.64333814	0.12187256
ENSG000001	CHMP4C	0.75593176	0.91047133	0.12187403
ENSG000001	ANKRD33B	0.08692699	0.42900716	0.12191966
ENSG000001	TARDBP	0.04244496	0.35072711	0.1219428
ENSG000001	GRM4	0.77871304	0.91810931	0.12194347
ENSG000001	APOA1	0.80905541	0.93015879	0.12194969
ENSG000001	PARS2	0.19744806	0.56521618	0.12199665
ENSG000001	NDN	0.51481351	0.79420539	0.1219998
ENSG000001	HUS1B	0.70813344	0.88904065	0.12206221
ENSG000001	CLDND2	0.37041298	0.70302695	0.12207361
ENSG00000(	FRYL	0.05040377	0.36244582	0.1221066
ENSG000001	ZBTB42	0.21283612	0.5782114	0.12212414
ENSG000001	WDR11	0.06311484	0.38891676	0.12214061
ENSG000001	AKR1A1	0.08570178	0.42602033	0.12218975
ENSG000002	MTCP1	0.47523718	0.77002538	0.12221409
ENSG000001	HHEX	0.02097806	0.28768647	0.12223317
ENSG000001	MRPL30	0.26218155	0.62575948	0.12223368
ENSG000001	EXOC2	0.03124401	0.32027086	0.12224677
ENSG000001	GALNT12	0.3347711	0.67675278	0.12225807
ENSG00000(	APPBP2	0.08954556	0.43356546	0.12227622
ENSG00000(	MAPK9	0.10303971	0.45287405	0.12230308
ENSG00000(	MRPS10	0.09745249	0.44410217	0.12237827
ENSG000001	GTF2B	0.1972219	0.56500135	0.12238397
ENSG000001	A4GALT	0.66436806	0.86854623	0.1223859
ENSG000001	FBXO45	0.14267563	0.50078343	0.12239812
ENSG000001	MRTFB	0.38184072	0.71079805	0.12241244
ENSG00000(	FYB1	0.11018203	0.46278943	0.12241522
ENSG000001	BATF	0.10981547	0.46255597	0.12241843
ENSG00000(	HACD3	0.23708545	0.603852	0.12243532
ENSG000001	CNST	0.21365817	0.57911862	0.12245052
ENSG000001	NAA35	0.10108671	0.44917064	0.12249856
ENSG000001	TMEM51	0.52787324	0.80062313	0.12254337
ENSG000001	IRF2	0.22141325	0.58882965	0.12261368
ENSG000001	ZNF738	0.44591252	0.75013279	0.12266833

ENSG000001	CLK4	0.3949248	0.72203513	0.1226861
ENSG000001	THOC2	0.04774267	0.35895177	0.12270624
ENSG000001	C1orf174	0.06967613	0.40004644	0.12276777
ENSG00000C	ELMO2	0.04934542	0.360767	0.12277569
ENSG000001	ZNF292	0.32261567	0.66849561	0.12279894
ENSG000001	UBXN2A	0.12477736	0.48220256	0.12280103
ENSG000001	TMEM30A	0.2164895	0.58303866	0.12281445
ENSG000001	NTM	0.7888255	0.92204735	0.12283193
ENSG00000C	MCUB	0.17161881	0.53577669	0.12284099
ENSG000001	NAPA	0.38911314	0.71812117	0.1229201
ENSG000001	UBE2F	0.0176613	0.27418797	0.12292176
ENSG000001	SARDH	0.48526744	0.77718414	0.12292496
ENSG000001	MPDU1	0.05535117	0.37400623	0.12295321
ENSG000001	SDAD1	0.05603768	0.37502119	0.12300473
ENSG000001	HINT1	0.47577546	0.7703596	0.12303788
ENSG000001	GCNT4	0.543433	0.8084284	0.12304596
ENSG000001	INVS	0.09153765	0.43611772	0.12306314
ENSG000001	RNF170	0.20796649	0.57145912	0.12307673
ENSG000001	XIRP1	0.69560364	0.88231682	0.12312263
ENSG000001	SAMD8	0.2808535	0.63927558	0.12323662
ENSG000001	ANKRD12	0.22451242	0.59186495	0.12326779
ENSG000001	SLC5A3	0.39118767	0.71964297	0.12327469
ENSG000001	THAP6	0.19665127	0.56403935	0.12335012
ENSG000001	SLC2A10	0.76352418	0.9121117	0.12343284
ENSG00000C	RBM41	0.27466118	0.63464829	0.12346146
ENSG000001	ATP5ME	0.40513017	0.72678142	0.12351232
ENSG000002	TVP23C-CDF	0.75475822	0.90980024	0.12357547
ENSG000001	COX6C	0.48647363	0.77752459	0.12358236
ENSG000001	ZNF493	0.3876863	0.71668724	0.1236423
ENSG000001	PCMT1	0.1082917	0.45963571	0.12364901
ENSG000001	PRKCI	0.27294561	0.63378205	0.12366173
ENSG00000C	DIMT1	0.1637426	0.52709285	0.1236938
ENSG000001	PBRM1	0.06001991	0.38277429	0.12373228
ENSG000002	ERVMER34-1	0.77783064	0.91778476	0.12373412
ENSG000002	COL6A6	0.70068088	0.88498263	0.12377323
ENSG000001	SHQ1	0.09008269	0.43377006	0.12377545
ENSG00000C	PDK4	0.51730383	0.79538919	0.12382928
ENSG000001	HNRNPA2B1	0.0122046	0.25275547	0.12385089
ENSG00000C	MAP3K13	0.11306382	0.46808189	0.1239149
ENSG000001	RNF2	0.19196351	0.5573876	0.1239256
ENSG00000C	GDI2	0.00777681	0.23002982	0.12393313
ENSG00000C	RAB10	0.01070025	0.24206404	0.12395673
ENSG000001	KLHL33	0.60794189	0.84138702	0.12406784

ENSG00000(	SLC25A40	0.25405226	0.61928338	0.1240688
ENSG00000(	OSTM1	0.19729985	0.56500135	0.12407172
ENSG00000(	SIRT1	0.17277475	0.53714513	0.12407672
ENSG000001	COMMD2	0.17531007	0.54013466	0.12409846
ENSG000001	FLVCR1	0.08462908	0.4247775	0.12411337
ENSG00000(	RABEP1	0.1690541	0.53299639	0.12414003
ENSG000002	SRRM5	0.44343326	0.74898844	0.12416662
ENSG000001	NKTR	0.24110066	0.60721888	0.12419323
ENSG000001	BMPR1A	0.382522	0.71170043	0.12426113
ENSG000001	NPAS1	0.70855088	0.88938447	0.12427184
ENSG000001	SCARA5	0.67396731	0.87391249	0.12431882
ENSG000001	MRPL19	0.12398642	0.48115857	0.12432201
ENSG000001	NKIRAS1	0.28141248	0.63945781	0.12433167
ENSG000001	ITGB1	0.14393895	0.50220003	0.12446941
ENSG00000(	HSPB11	0.23558751	0.60287382	0.12447593
ENSG000001	PUM1	0.05443492	0.37309407	0.12452292
ENSG000001	ZBTB38	0.28113265	0.63941315	0.1245586
ENSG000001	TNS3	0.11548446	0.4710215	0.12459183
ENSG000001	EHHADH	0.24821911	0.6144738	0.1246035
ENSG000001	ARL6IP6	0.03054243	0.3191802	0.12461016
ENSG000001	EXOG	0.29422655	0.65044158	0.12461913
ENSG00000(	CBFB	0.14542095	0.50417338	0.12461974
ENSG000002	RNF224	0.83809449	0.94146888	0.12462625
ENSG000001	SKP2	0.20199697	0.56775306	0.1246269
ENSG000001	NCK1	0.1753588	0.54018229	0.12467558
ENSG000001	ACSM1	0.4277028	0.73965389	0.124705
ENSG000001	ELK3	0.07063952	0.40156223	0.12471229
ENSG00000(	TSPOAP1	0.22572565	0.59319591	0.124716
ENSG000001	CKS1B	0.30480089	0.65635638	0.12483378
ENSG000001	ABR	0.56450552	0.81896677	0.12485399
ENSG00000(	FCGR2B	0.16147309	0.52426286	0.12486142
ENSG000001	ZNF461	0.3104907	0.65949478	0.12486555
ENSG000001	MCPH1	0.10595261	0.4561473	0.12488758
ENSG000002	ARHGAP8	0.79833648	0.92603117	0.12495308
ENSG000001	DCTN6	0.27994445	0.63885567	0.12495564
ENSG000001	KYNU	0.18940247	0.55422631	0.12497355
ENSG000001	ZEB2	0.04864593	0.360767	0.12503619
ENSG000001	LAMA2	0.42777047	0.73969214	0.12504937
ENSG000002	C2orf16	0.39343702	0.72063539	0.12507834
ENSG000001	RCOR3	0.1741171	0.5392217	0.12522867
ENSG000001	KRBOX4	0.29343565	0.6501794	0.12526469
ENSG00000(	NT5C2	0.17406767	0.5392217	0.12526681
ENSG000001	PRRC1	0.17436824	0.53940156	0.12532732

ENSG000001	DMAC2L	0.18623587	0.55069275	0.12540011
ENSG000001	GGACT	0.26436007	0.6274921	0.12540717
ENSG000001	ZNF32	0.40313526	0.72672584	0.12540744
ENSG000001	PDSS1	0.25256817	0.61835786	0.1254427
ENSG000001	ZNF558	0.35419956	0.69167785	0.1254925
ENSG000001	MAGI2	0.73948418	0.90407383	0.12550564
ENSG000001	SOX15	0.5217638	0.79765359	0.12553167
ENSG000001	CBX3	0.2687187	0.63079288	0.12555738
ENSG000001	NPIPB4	0.19664873	0.56403935	0.1255936
ENSG000001	SLAIN1	0.26632115	0.62912347	0.12563241
ENSG00000C	ANAPC4	0.14447157	0.50306345	0.1256489
ENSG000001	UTP25	0.10427757	0.45468989	0.12564924
ENSG000001	CAPRIN2	0.32407991	0.6699145	0.12567893
ENSG00000C	WDR70	0.14441885	0.50298774	0.12568426
ENSG00000C	WNT16	0.59397336	0.83456808	0.12568617
ENSG000001	ARHGAP29	0.53313506	0.80294166	0.12570319
ENSG000001	PLAT	0.56436783	0.81891336	0.12575202
ENSG000001	ZNF273	0.3896707	0.7188269	0.12582424
ENSG000002	N/A	0.71662886	0.89259764	0.12582983
ENSG000001	DNAL1	0.31162995	0.66022122	0.12586923
ENSG000001	LARS1	0.05520782	0.37354464	0.12595759
ENSG000001	CAPN7	0.3064521	0.65735608	0.12597616
ENSG000002	RNASE13	0.77685484	0.91723328	0.12599191
ENSG00000C	VAMP3	0.09452837	0.43981122	0.12603219
ENSG00000C	UPRT	0.18634448	0.55072357	0.12605369
ENSG00000C	NUAK1	0.74643337	0.90663066	0.12606408
ENSG000001	AHI1	0.41405438	0.73141372	0.12611368
ENSG000001	SLC30A5	0.11856511	0.47520101	0.12614179
ENSG000001	E2F6	0.17239966	0.53666665	0.12615377
ENSG000001	EDEM3	0.32107569	0.66766831	0.1261977
ENSG000001	MTPAP	0.12225471	0.47887061	0.12621808
ENSG000001	ZFAND1	0.41189477	0.73036315	0.12622356
ENSG000001	MYRF	0.31276725	0.66112215	0.12625077
ENSG00000C	TBPL1	0.03302606	0.32362351	0.12625885
ENSG000001	HPRT1	0.14706181	0.5054701	0.12628417
ENSG00000C	ARHGEF10L	0.09803376	0.44463533	0.12629354
ENSG000001	TTC13	0.25035199	0.61627732	0.12631348
ENSG000001	TMEM237	0.34026521	0.68135285	0.12633441
ENSG000001	SGO1	0.39900143	0.72496091	0.12635115
ENSG000002	ITSN1	0.0782971	0.41427772	0.12638043
ENSG000001	TMEM150B	0.33976556	0.68125135	0.12644441
ENSG000001	CALR	0.04657768	0.35774922	0.12645145
ENSG000002	SPDYE2	0.29715987	0.65264233	0.12648238

ENSG000001	CCZ1	0.0572214	0.37676557	0.12653098
ENSG000001	CEP85	0.0682171	0.39743754	0.12654097
ENSG000001	PEAK1	0.1454652	0.50417338	0.12654993
ENSG000001	TDG	0.05247215	0.36850788	0.12656206
ENSG000002	TERB1	0.81467071	0.93225797	0.12660195
ENSG000001	ECPAS	0.00269762	0.2093801	0.12660258
ENSG000001	TBC1D2B	0.01501214	0.26260224	0.12660886
ENSG000001	UBA3	0.1195834	0.47604098	0.12666652
ENSG000001	CEP170	0.6352132	0.85504577	0.1267319
ENSG00000C	DHX29	0.08363901	0.42260872	0.12677266
ENSG000001	PCF11	0.09067124	0.43454471	0.12681836
ENSG000001	OGT	0.30957526	0.65916845	0.12684442
ENSG000001	GIMAP7	0.30750473	0.65762361	0.12687214
ENSG000001	PPP1R26	0.12852141	0.48678709	0.12701814
ENSG000001	APBA1	0.78704165	0.9213551	0.12706836
ENSG000001	RPF1	0.14282222	0.5010464	0.12710387
ENSG000001	ABHD18	0.08566004	0.42602033	0.12711078
ENSG000001	TBX19	0.07495689	0.40825944	0.12711115
ENSG00000C	KLHL20	0.18168954	0.54641473	0.12711899
ENSG000001	ZNF34	0.07934095	0.4152849	0.12712426
ENSG000001	ZNF514	0.34794129	0.68740599	0.12716849
ENSG000001	EXTL2	0.39070572	0.71964297	0.12723906
ENSG00000C	ORC1	0.47082526	0.76685063	0.12725134
ENSG000002	ZNF891	0.53165674	0.80232066	0.12727892
ENSG000001	ZNF347	0.34006541	0.68135285	0.12727983
ENSG000001	ZNF223	0.57637101	0.82397739	0.12732215
ENSG000002	ZNF253	0.37273703	0.70471951	0.12745166
ENSG000001	KCNJ2	0.37777776	0.70909881	0.12749869
ENSG000001	GTPBP10	0.17130013	0.53539947	0.12755385
ENSG00000C	MTMR1	0.01661484	0.27031359	0.1275728
ENSG000001	TTC30A	0.41185556	0.73036315	0.12762262
ENSG000001	GNLY	0.35959267	0.69484359	0.12763543
ENSG000001	PDCD4	0.00522692	0.21833269	0.1276491
ENSG000001	PDZD11	0.14134052	0.50055578	0.12767079
ENSG000001	MTM1	0.07740297	0.41266462	0.12770492
ENSG000002	N/A	0.74100604	0.90518471	0.12772627
ENSG000001	LGR6	0.38821879	0.71704113	0.12780461
ENSG000001	TTC5	0.08983403	0.43377006	0.12783191
ENSG000001	SCN8A	0.64377516	0.85940463	0.12783305
ENSG000001	MYCBPAP	0.51779815	0.79566132	0.1278633
ENSG000001	LMO7	0.28786159	0.64495772	0.12788178
ENSG000001	PIGM	0.16045247	0.52341736	0.12788978
ENSG00000C	KLHL42	0.30717651	0.65762361	0.12790026

ENSG000001	GSE1	0.0282416	0.31279591	0.12795639
ENSG000001	CEBPG	0.14620569	0.50446567	0.12801969
ENSG000001	SLC33A1	0.21256446	0.57802866	0.12802255
ENSG000001	MKKS	0.24540882	0.61141177	0.12803854
ENSG000001	G3BP2	0.05903231	0.38096018	0.12806658
ENSG000001	DNAAF2	0.3195414	0.66652314	0.12810895
ENSG000001	ZKSCAN3	0.24910058	0.61496823	0.12811482
ENSG000001	SEC23A	0.13279793	0.49139472	0.12818977
ENSG000002	XKR9	0.80515886	0.92897486	0.12819965
ENSG000001	GGPS1	0.11429709	0.46971445	0.12822099
ENSG000002	SLC35G6	0.77414325	0.91603002	0.12825793
ENSG000001	ST18	0.78093507	0.9189743	0.12826873
ENSG000001	TC2N	0.52408576	0.79870477	0.12843187
ENSG000001	ANKRD40	0.09272206	0.43753271	0.12847535
ENSG000001	C3orf14	0.46968542	0.7662622	0.12852257
ENSG000001	KCTD1	0.27752636	0.63709819	0.12852679
ENSG000001	TMEM52B	0.7534406	0.90929203	0.12854062
ENSG000002	C2orf81	0.5434737	0.8084284	0.12855712
ENSG000001	TEX10	0.16550094	0.52885285	0.12858689
ENSG00000C	FGFR2	0.45544617	0.75622042	0.12859908
ENSG000001	BTF3L4	0.17470126	0.53975461	0.12860455
ENSG00000C	NECAP1	0.03473365	0.32831054	0.12862819
ENSG000001	ZG16B	0.53379262	0.80355932	0.12864433
ENSG000001	ZNF189	0.11025053	0.46278943	0.12867558
ENSG000001	GSTK1	0.03674788	0.33341755	0.12868454
ENSG000002	YAE1	0.5202218	0.79709497	0.12877034
ENSG000001	KBTBD6	0.37093685	0.70329686	0.12889309
ENSG000001	TRPS1	0.14508778	0.50391236	0.12893407
ENSG00000C	ARHGAP31	0.05733117	0.37687699	0.12893507
ENSG000001	METTL23	0.10499101	0.45514301	0.12895481
ENSG000001	OXCT2	0.65288305	0.86379938	0.12897637
ENSG00000C	DLG3	0.28461861	0.64283661	0.12898782
ENSG00000C	FAM76A	0.11653906	0.47188147	0.12899943
ENSG00000C	ANKRD10	0.18541449	0.55033096	0.1290004
ENSG000001	POLH	0.06792006	0.39726872	0.12900886
ENSG000002	C5orf51	0.03470869	0.32831054	0.12901261
ENSG000001	ZNF562	0.06820371	0.39743754	0.12912031
ENSG000002	HMGN1	0.20488	0.56891466	0.12912509
ENSG000001	UBE4A	0.15183691	0.51292686	0.12912692
ENSG000001	C18orf21	0.10108488	0.44917064	0.12916829
ENSG000001	ZNF138	0.49073681	0.7800506	0.12922993
ENSG00000C	JPH4	0.43909323	0.74609757	0.12924266
ENSG00000C	NFE2L3	0.08798623	0.43050213	0.12934379

ENSG000001	ATMIN	0.10007488	0.44762396	0.12935649
ENSG000001	PPWD1	0.28698607	0.64432975	0.12937659
ENSG000001	CEP76	0.24444536	0.61034282	0.12938257
ENSG000001	JCHAIN	0.64926999	0.86252724	0.12940347
ENSG000001	RNF17	0.60530255	0.83945694	0.12943903
ENSG000001	COQ5	0.27142347	0.63291553	0.12954041
ENSG000001	SRP72	0.18189245	0.54661139	0.12954089
ENSG000001	ERC2	0.78437911	0.92028573	0.12957467
ENSG000001	NOTUM	0.70809057	0.88904065	0.1296891
ENSG000001	KCNC1	0.8118819	0.93164748	0.12969759
ENSG000002	TMEM170B	0.34316634	0.68384779	0.12975614
ENSG000001	LRP12	0.26155274	0.62545387	0.12982862
ENSG000001	VPS45	0.09638969	0.44215472	0.12983498
ENSG000001	FIG4	0.02286247	0.29500624	0.12985481
ENSG00000(	CALCRL	0.52867417	0.80075396	0.12989238
ENSG000001	SLC30A6	0.08136292	0.41884321	0.12990178
ENSG000001	GOLM1	0.04634813	0.35767802	0.12992127
ENSG000001	CALCOCO2	0.11566314	0.47104419	0.12992242
ENSG00000(	MON2	0.12682434	0.48465716	0.12997584
ENSG000001	RAB40AL	0.73519304	0.90147373	0.1300808
ENSG000001	DEGS2	0.5355629	0.80413676	0.13011155
ENSG000001	DESI2	0.12866445	0.48686197	0.13019381
ENSG000001	COIL	0.15856996	0.52129996	0.1302193
ENSG00000(	GUCY1B1	0.22594471	0.59319591	0.1302233
ENSG000001	XPA	0.32085665	0.66766831	0.13022557
ENSG00000(	SNX5	0.08559321	0.42602033	0.1302737
ENSG000001	PUS3	0.05156319	0.36531299	0.13030645
ENSG000001	CCDC134	0.22394998	0.59136051	0.1303235
ENSG000001	PPHLN1	0.08939673	0.43342334	0.13044083
ENSG000001	CENPN	0.29227576	0.64947398	0.13047606
ENSG000001	CCL28	0.33218958	0.6750641	0.13053812
ENSG000001	SAYSD1	0.06371733	0.38981851	0.13055046
ENSG000001	WDR27	0.32215726	0.66822528	0.13055405
ENSG000002	CCER2	0.60422327	0.83883394	0.13056616
ENSG000001	FIGNL1	0.36930463	0.70215447	0.13060039
ENSG000001	COG3	0.09889495	0.4460792	0.13066675
ENSG000002	PPIL3	0.37574299	0.70743725	0.13067549
ENSG000001	ZSCAN30	0.19788929	0.56540555	0.13073045
ENSG000001	TMEM170A	0.04530191	0.35545023	0.13076773
ENSG000001	PDLIM5	0.00110991	0.18856694	0.13080132
ENSG000002	N/A	0.74962703	0.90788681	0.13080226
ENSG000001	OXSM	0.36323275	0.69694698	0.13085595
ENSG000001	USP14	0.13592826	0.49574736	0.13086605

ENSG000001	UBE2N	0.10243213	0.45177448	0.13088667
ENSG000001	GTF3C4	0.08686634	0.42885945	0.1309564
ENSG000001	RWDD4	0.13832413	0.49898885	0.13095902
ENSG000001	CCDC170	0.18048769	0.54522392	0.13096991
ENSG000001	ZNHIT6	0.13995242	0.4999841	0.13097699
ENSG000001	CCSAP	0.13868249	0.49917703	0.13100488
ENSG000001	C9orf85	0.20460875	0.5688754	0.13101366
ENSG000001	RIPK1	0.07865322	0.41446832	0.13103074
ENSG00000(	ADAM22	0.49440614	0.78124152	0.13119163
ENSG000001	VTN	0.423589	0.73732623	0.13120724
ENSG000001	GATAD2B	0.28551492	0.64333814	0.13123682
ENSG000001	ZKSCAN2	0.18555886	0.55033096	0.13135895
ENSG000001	RNASEH1	0.06010351	0.3831569	0.13139549
ENSG000001	PEX12	0.10735953	0.45831177	0.13140464
ENSG000001	DNAJB9	0.2457147	0.61158271	0.13146615
ENSG000001	PABIR2	0.14114851	0.50055066	0.13148543
ENSG00000(	TRAF5	0.30299851	0.65591444	0.13149171
ENSG000001	PTBP2	0.27730678	0.63709819	0.13150093
ENSG000001	SLC1A1	0.71130024	0.8906831	0.1315379
ENSG000001	SENP6	0.17344227	0.53805368	0.13155368
ENSG000001	UEVLD	0.06095348	0.38464604	0.13155533
ENSG000001	BTN3A2	0.04986145	0.36170272	0.13158253
ENSG000001	NFATC2	0.1288766	0.4871454	0.13161699
ENSG000001	DCLRE1C	0.05647966	0.3750908	0.1316503
ENSG000001	RMND1	0.24935936	0.61525038	0.13172874
ENSG000001	ZNF527	0.22336386	0.59081204	0.13173166
ENSG00000(	CLEC2D	0.34527594	0.68522517	0.13177377
ENSG000001	TES	0.00198194	0.19503458	0.13177925
ENSG000001	KIRREL3	0.43287209	0.74258259	0.13179033
ENSG000001	SH2D1A	0.47149536	0.76694135	0.13181934
ENSG000001	SLC10A4	0.76158762	0.91151487	0.13183747
ENSG000001	CAND1	0.13153666	0.4894044	0.13188402
ENSG000001	GFPT1	0.16206339	0.52481515	0.13188823
ENSG000001	LSM1	0.29459002	0.65098879	0.13192056
ENSG000001	WDHD1	0.38160065	0.7106995	0.13193797
ENSG000001	DDX10	0.10950227	0.46193514	0.13196415
ENSG000002	RTEL1	0.44738605	0.75099423	0.13204785
ENSG000001	HIGD1A	0.31081458	0.65949478	0.13206661
ENSG000001	UTP3	0.02236644	0.29338932	0.13216259
ENSG000001	UBTD2	0.12938313	0.4871968	0.13217387
ENSG000002	ZNF155	0.20020633	0.56699578	0.13223536
ENSG000001	TMEM184C	0.01915619	0.28080056	0.13231629
ENSG000001	ZNF614	0.29643818	0.65199276	0.13236859

ENSG000001	TRMT10C	0.33073476	0.6742308	0.13247201
ENSG000001	GTF2E1	0.09043999	0.43397794	0.13247937
ENSG000001	PEX11A	0.25378063	0.61921212	0.13254589
ENSG000002	NUP62	0.01321391	0.25805013	0.13257489
ENSG000001	MRPL42	0.2902858	0.648324	0.13261087
ENSG000001	HMBOX1	0.12488916	0.48247093	0.13262808
ENSG000001	RMDN3	0.03290191	0.32362351	0.13265418
ENSG000001	CETN3	0.62539349	0.85037415	0.13269198
ENSG000001	GTF2A1	0.07150165	0.40241675	0.13269508
ENSG000001	GLMN	0.20269776	0.56815551	0.13274336
ENSG000002	GOLGA6B	0.77643392	0.91706973	0.13275349
ENSG000001	TCERG1	0.13540783	0.49462699	0.13277
ENSG000001	MAPK8	0.20413167	0.5688169	0.13277192
ENSG00000C	CCAR1	0.1821351	0.54662721	0.13277838
ENSG00000C	RAD18	0.12236798	0.47887061	0.13289875
ENSG000001	RANGAP1	0.07925274	0.4152849	0.13292609
ENSG000001	COPS5	0.11974011	0.47604098	0.13299973
ENSG000001	MTRF1L	0.09290304	0.43758758	0.13304248
ENSG000001	CASKIN1	0.76134413	0.91150138	0.13304405
ENSG000001	WASHC3	0.18529462	0.55032535	0.13309024
ENSG000001	STN1	0.07176502	0.40264293	0.13310674
ENSG000001	SUSD1	0.01647301	0.27000801	0.13317518
ENSG000001	MT1E	0.51219992	0.7921414	0.13319619
ENSG000001	GPR135	0.55800331	0.81587881	0.13322449
ENSG00000C	USP28	0.08593025	0.42668178	0.13325356
ENSG000001	ZNF549	0.25670343	0.62074989	0.13331996
ENSG000001	NR1D2	0.29016593	0.648324	0.13333727
ENSG000001	PTPN13	0.44108363	0.74750808	0.13336802
ENSG00000C	ZFX	0.08116087	0.41848493	0.1334044
ENSG000001	PPAT	0.25166623	0.6172357	0.13341159
ENSG000001	ZFC3H1	0.09426928	0.43946321	0.13351877
ENSG00000C	FUCA2	0.04165488	0.34831883	0.13355047
ENSG000001	PLEK	0.1384851	0.49898885	0.13359569
ENSG000001	POLR3G	0.48700384	0.77767904	0.13364291
ENSG000001	ACBD3	0.04383812	0.35372007	0.13369454
ENSG000001	MTHFD1	0.0695059	0.40004644	0.13369557
ENSG000001	ATG10	0.18896022	0.55371723	0.13371183
ENSG000001	ANKEF1	0.23025327	0.59855451	0.13377094
ENSG000001	TRMT5	0.17355386	0.53809318	0.1337731
ENSG000001	CD180	0.18006971	0.54501079	0.13378623
ENSG000001	CEP192	0.12837602	0.48656428	0.13380247
ENSG000001	PIK3CA	0.1703577	0.53437545	0.13381559
ENSG000001	FNBP4	0.14828653	0.50753127	0.1338249

ENSG000001	TAF1D	0.17831624	0.54332516	0.13395461
ENSG00000C	MCF2L2	0.3774389	0.70874008	0.133957
ENSG000001	PSMA1	0.07900159	0.4147264	0.1340012
ENSG000001	RMDN1	0.1412558	0.50055578	0.13402267
ENSG000001	B3GALNT2	0.18037328	0.54515554	0.13402742
ENSG00000C	PHLPP2	0.25706288	0.62086214	0.13407263
ENSG000001	GSTO1	0.09459488	0.43984453	0.13415757
ENSG000002	IRF9	0.18968742	0.55424774	0.1342629
ENSG000001	CCDC102B	0.51788371	0.79566132	0.13426659
ENSG00000C	ZFR	0.09191287	0.43640386	0.13428243
ENSG000001	ATP2A2	0.00879947	0.23442641	0.13428935
ENSG000001	SLC35B3	0.12807301	0.48599936	0.13434929
ENSG00000C	LYZ	0.12601244	0.48388732	0.13437438
ENSG000001	PLAAT5	0.36730831	0.69974413	0.13439959
ENSG000001	ELOVL4	0.52686517	0.80032903	0.13442554
ENSG000001	MMP1	0.7361343	0.90182256	0.13443945
ENSG00000C	KIFAP3	0.12082259	0.47724625	0.13447265
ENSG000001	RAP1GDS1	0.06428885	0.39043354	0.13450623
ENSG000001	USP42	0.01768029	0.27418797	0.13452403
ENSG000001	GFM2	0.15827252	0.52089921	0.13454212
ENSG00000C	SCYL3	0.01242086	0.25464329	0.13465284
ENSG000001	FASTKD2	0.17940158	0.54453941	0.13468377
ENSG00000C	NEDD4	0.16393615	0.52712713	0.13470002
ENSG000001	HEATR1	0.04330737	0.35282577	0.13470006
ENSG000001	NIPA1	0.298954	0.65423918	0.13470662
ENSG000001	USP15	0.21639379	0.58303866	0.13474359
ENSG000001	BCL11A	0.0488139	0.360767	0.13475221
ENSG00000C	ATP8B1	0.12121125	0.47724625	0.13476488
ENSG000002	LRRC69	0.4140319	0.73141372	0.13487487
ENSG000002	CYFIP1	0.04517095	0.35545023	0.13487555
ENSG000001	MKLN1	0.108355	0.45963571	0.13488589
ENSG000001	LRRC58	0.11739205	0.47379934	0.13489643
ENSG00000C	RC3H2	0.05324995	0.36981204	0.13490768
ENSG000001	DHCR24	0.190171	0.55516118	0.13491848
ENSG000001	SECISBP2L	0.14721824	0.50568178	0.13497092
ENSG000001	RNASE7	0.80518806	0.92897486	0.13499068
ENSG000001	ZNF222	0.16537324	0.52878404	0.13499734
ENSG00000C	DERA	0.03960228	0.34093258	0.13502695
ENSG000002	METTL6	0.11720844	0.47331831	0.13504694
ENSG00000C	EPS15	0.00408627	0.21403539	0.13511162
ENSG000001	HTR6	0.57777324	0.82508213	0.13519889
ENSG000001	ZNF383	0.27558995	0.63512113	0.13526195
ENSG00000C	RBM27	0.10464139	0.45514301	0.13526992

ENSG000001	PIKFYVE	0.17246325	0.5367617	0.13535219
ENSG000001	CDKN3	0.68604064	0.8799229	0.13544668
ENSG00000(	OPN3	0.15799445	0.52077869	0.13551824
ENSG000001	TOMM70	0.14369565	0.50186843	0.13551858
ENSG000001	TMEM209	0.17129378	0.53539947	0.13555145
ENSG000001	SRP19	0.10013485	0.44765683	0.13558218
ENSG000002	RBM14-RBM	0.23208481	0.59952843	0.135584
ENSG000001	NCBP2	0.1416196	0.50055578	0.13563494
ENSG000001	POMP	0.11370447	0.46915085	0.13567251
ENSG000001	NUFIP2	0.15228251	0.51327042	0.13568284
ENSG000001	KMT2A	0.0929522	0.43758758	0.13572383
ENSG000002	TBCE	0.23627968	0.60336718	0.13573895
ENSG000001	ZFP69B	0.40507052	0.7267547	0.13580424
ENSG000001	ZNF780A	0.20289253	0.56858183	0.13583708
ENSG000001	DLX1	0.74279466	0.90586851	0.13585916
ENSG000001	ATM	0.33114292	0.67443203	0.13595833
ENSG000001	SEC61G	0.26491711	0.6274921	0.13598463
ENSG00000(	DIS3	0.12085986	0.47724625	0.13602258
ENSG000001	BBS12	0.23898746	0.60520581	0.13603795
ENSG000002	CD24	0.26448264	0.6274921	0.13605382
ENSG000001	C1orf216	0.07271206	0.40432385	0.13606097
ENSG000001	ZNF808	0.18465126	0.5498217	0.13607259
ENSG000001	ZNF83	0.36947522	0.70231435	0.13607399
ENSG000001	FUBP3	0.06101972	0.38464604	0.13613135
ENSG000001	KPNA2	0.07668319	0.41092575	0.13614561
ENSG000001	BET1	0.25726924	0.62106462	0.13614853
ENSG000001	SIRPD	0.32211729	0.66822528	0.13618755
ENSG000001	ZNF330	0.03366125	0.32398208	0.13622807
ENSG00000(	CPOX	0.19334504	0.55999703	0.13630332
ENSG000001	APOOL	0.09472135	0.43988973	0.13638075
ENSG000001	CEP120	0.27451004	0.63464829	0.13639475
ENSG00000(	MTFR1	0.3575144	0.69362664	0.13641518
ENSG00000(	SEH1L	0.06173651	0.38552656	0.13641736
ENSG000001	SASS6	0.24962684	0.61563048	0.13653231
ENSG000001	TULP2	0.72287598	0.89570645	0.13659458
ENSG000001	ZNF420	0.35124466	0.69054758	0.13660526
ENSG00000(	TXK	0.28750641	0.64495772	0.13663206
ENSG00000(	BZW1	0.03474496	0.32831054	0.13667032
ENSG000001	MINPP1	0.11173465	0.46538092	0.13667786
ENSG000002	PHACTR4	0.04850834	0.360767	0.13671786
ENSG000001	ABCA5	0.45133771	0.75378336	0.13678558
ENSG000001	LOXL1	0.38371217	0.7123445	0.13679543
ENSG000001	TTC39C	0.14253738	0.50068215	0.13687401

ENSG000001	PROSER1	0.01055018	0.24206404	0.13689911
ENSG000001	PTS	0.30963095	0.65916845	0.13696189
ENSG00000(	SLC12A2	0.25706106	0.62086214	0.13697086
ENSG000001	SLC19A2	0.40573512	0.72730417	0.13697729
ENSG000001	DNAH11	0.46863918	0.76598493	0.1370676
ENSG000001	ADGRA3	0.40697533	0.72768322	0.13710693
ENSG000001	PRKG2	0.58569149	0.82959975	0.13711315
ENSG000002	N/A	0.3751456	0.70720386	0.13713941
ENSG000001	SCML4	0.17504704	0.53975461	0.13724616
ENSG000001	DENND4C	0.14006051	0.50003573	0.1372657
ENSG000002	N/A	0.67842384	0.87557173	0.13727728
ENSG00000(	TRMT6	0.06429758	0.39043354	0.13728215
ENSG000001	GTPBP1	0.22479135	0.59200912	0.13734391
ENSG000001	WWP1	0.19536004	0.56282131	0.13734496
ENSG000001	PLA2G2D	0.64144067	0.85756048	0.13739552
ENSG000001	SS18	0.07978679	0.41535686	0.13740653
ENSG000001	IDH1	0.05521991	0.37354464	0.13743928
ENSG000001	MORF4L2	0.10579182	0.4561473	0.13758206
ENSG00000(	PGM3	0.29187352	0.64936748	0.13762499
ENSG000002	HLA-E	0.27320743	0.6338147	0.13763273
ENSG000001	TNKS2	0.03962184	0.34093258	0.13764462
ENSG000001	ZNF236	0.18580323	0.55052683	0.13773696
ENSG00000(	HEATR5B	0.03832049	0.33669362	0.13776235
ENSG00000(	CD22	0.09004402	0.43377006	0.13780609
ENSG00000(	PLEKHG6	0.27345571	0.6339378	0.13781084
ENSG000001	PPIL4	0.20912883	0.57310021	0.13784531
ENSG00000(	ZNF207	0.06058911	0.38444089	0.13785765
ENSG000001	METTL18	0.4558594	0.75632853	0.13785946
ENSG000002	TRIM59	0.30462517	0.65635638	0.1378597
ENSG000001	UBXN4	0.10330252	0.45287405	0.13797489
ENSG000001	LAMA5	0.24867344	0.61459845	0.138024
ENSG000001	FNTA	0.12987885	0.48787839	0.13808571
ENSG000001	NIFK	0.33449827	0.67664065	0.13813458
ENSG000001	SRSF2	0.05850799	0.3800398	0.13814536
ENSG000001	CALCA	0.74165818	0.90543757	0.13817807
ENSG000001	SGK3	0.09042432	0.43397794	0.13819076
ENSG000001	LARGE1	0.26046663	0.62395939	0.1382705
ENSG000001	FSCN3	0.60032657	0.83755822	0.1383076
ENSG000001	MDN1	0.20398412	0.5688169	0.13837701
ENSG000001	ITPR2	0.03822308	0.33650813	0.13840277
ENSG000001	NKAPD1	0.20347061	0.56880022	0.13844351
ENSG00000(	CD59	0.15782346	0.52077869	0.1385013
ENSG000001	SLC18B1	0.10076094	0.44884919	0.13859226

ENSG000001	CEP44	0.28525597	0.64333814	0.138659
ENSG000001	LENG1	0.67799394	0.87536479	0.13870536
ENSG000001	HIKESHI	0.16313888	0.52653704	0.13871965
ENSG000001	SERHL2	0.25787377	0.62198226	0.138725
ENSG000001	BCOR	0.05430562	0.37256839	0.1387758
ENSG000001	KIF23	0.29826235	0.65366489	0.13884568
ENSG000001	FAR1	0.25486134	0.6197628	0.13892493
ENSG000001	CDADC1	0.13710315	0.49766113	0.13899566
ENSG00000(	MRPS35	0.13004482	0.48787839	0.13902308
ENSG000001	FZD2	0.13747702	0.49827463	0.13902521
ENSG000001	MIX23	0.35151067	0.69054758	0.13902897
ENSG000001	TMEM60	0.10809516	0.45960368	0.1390629
ENSG000001	SPRY1	0.47105473	0.76694135	0.1391028
ENSG000001	MNX1	0.67563298	0.87451032	0.1391075
ENSG000001	ZNF701	0.13313205	0.49200959	0.13914061
ENSG000002	NUTM2D	0.13235319	0.49081838	0.13915253
ENSG000001	SUCLA2	0.19936613	0.56600138	0.13917036
ENSG000001	EIF3J	0.11602145	0.47160917	0.13929342
ENSG000001	PURA	0.14237129	0.50068215	0.13933465
ENSG000001	POMC	0.23838532	0.60460208	0.13938111
ENSG000001	MAP3K7	0.10634508	0.45644331	0.13942689
ENSG00000(	DLD	0.08973608	0.43377006	0.13949448
ENSG000002	APOBEC3C	0.073215	0.40493325	0.13949706
ENSG000001	NIF3L1	0.1263183	0.48388732	0.13953449
ENSG000001	SAV1	0.10577557	0.4561473	0.13960549
ENSG000001	ZNF501	0.32564585	0.67124189	0.13965894
ENSG000001	CNOT6L	0.04824075	0.360767	0.13970311
ENSG00000(	DYNC1I2	0.04909013	0.360767	0.1397049
ENSG000001	RYK	0.20034973	0.56713365	0.13977298
ENSG000001	ELMOD2	0.2875586	0.64495772	0.13979203
ENSG000001	SLC12A7	0.08620496	0.4272619	0.13979302
ENSG000001	ZNF706	0.07665622	0.41092575	0.13980357
ENSG00000(	GGCT	0.17677397	0.54166428	0.13991907
ENSG000002	ABCF1	0.74129511	0.90533613	0.13996826
ENSG000001	GUF1	0.12075375	0.47724625	0.14006618
ENSG00000(	TEKT2	0.46321854	0.76288461	0.14007004
ENSG000001	TRMT61B	0.25809568	0.62198226	0.1400748
ENSG000001	LOXL2	0.07195622	0.40315841	0.14008127
ENSG000002	H2BC18	0.34927634	0.68858669	0.1401584
ENSG000001	MRS2	0.1759536	0.54095022	0.14020106
ENSG000001	USP47	0.13921609	0.49975817	0.14029799
ENSG000001	TTL	0.02285857	0.29500624	0.14033625
ENSG000001	HSP90B1	0.0449047	0.35545023	0.14033824

ENSG000001	TMOD3	0.00050641	0.18856694	0.14035149
ENSG00000(	DTNBP1	0.05318595	0.36981204	0.14035636
ENSG000002	C11orf94	0.73006501	0.89828814	0.14043055
ENSG000002	SACM1L	0.17838522	0.54332516	0.14043182
ENSG000001	MTSS2	0.40669858	0.7275473	0.14046012
ENSG000001	FKTN	0.29490389	0.65121101	0.14056046
ENSG000002	ZNF260	0.31587774	0.66350671	0.1405617
ENSG000001	ALDH1L2	0.44250466	0.74855288	0.14056693
ENSG00000(	ACER3	0.02585929	0.30449397	0.14061336
ENSG000001	C4orf50	0.48484085	0.7769018	0.14071473
ENSG000001	ZNF569	0.42197765	0.73601655	0.14076135
ENSG000001	THBS4	0.23299276	0.60037143	0.14077957
ENSG000001	ZNF480	0.35518598	0.6919142	0.14078049
ENSG000002	RFPL4A	0.32726868	0.67217574	0.14079331
ENSG000001	NRXN2	0.4557089	0.75632853	0.14080526
ENSG00000(	BCLAF1	0.09837579	0.4448142	0.14082502
ENSG000001	SLC35A1	0.25671833	0.62074989	0.14090733
ENSG000001	C2CD5	0.19339258	0.56003484	0.14097001
ENSG000001	KAT2B	0.04501536	0.35545023	0.14097416
ENSG00000(	JADE2	0.02079592	0.28641584	0.14101443
ENSG00000(	PTPN4	0.27938718	0.63830383	0.14101572
ENSG000001	ABHD3	0.0940224	0.43929464	0.14102189
ENSG000001	MAN2A1	0.08570006	0.42602033	0.14107706
ENSG000001	RASGRP1	0.25468892	0.61971887	0.14108713
ENSG000001	WHAMM	0.02070167	0.28641584	0.14109589
ENSG00000(	UBE2T	0.48962955	0.77987726	0.14111805
ENSG000001	ABRACL	0.11652807	0.47188147	0.14115767
ENSG000002	TAF9	0.704737	0.88716768	0.14120713
ENSG000001	IGFBP4	0.26090983	0.6244683	0.14121887
ENSG000001	MTMR10	0.19156496	0.55704454	0.14123513
ENSG000001	STX2	0.25507509	0.61991532	0.14124018
ENSG00000(	RRAGB	0.27908287	0.63787563	0.14126651
ENSG000001	RAD51C	0.27176325	0.63323084	0.1413708
ENSG000001	CCDC43	0.12956559	0.4871968	0.14137462
ENSG000001	IFNG	0.64090845	0.85737789	0.14139675
ENSG000001	MCEE	0.34295597	0.68359637	0.14139898
ENSG000001	RPL9	0.56374922	0.81864675	0.14142659
ENSG000001	ATAD2	0.20773983	0.57145912	0.14146277
ENSG000001	ENAM	0.52862147	0.80074884	0.14149526
ENSG000001	FCRLA	0.14244251	0.50068215	0.14152637
ENSG000001	SPRY2	0.29278542	0.64967561	0.1415472
ENSG000001	AREG	0.74400788	0.90628646	0.14154963
ENSG000001	RSRC2	0.08927683	0.43342334	0.14164712

ENSG000001	TM2D2	0.02997067	0.31859418	0.14180206
ENSG000001	IDE	0.02982423	0.31849971	0.14180484
ENSG000001	IRAK1BP1	0.4238714	0.73732623	0.14181703
ENSG000001	ZNF627	0.19132158	0.55685897	0.1418862
ENSG000001	SLC10A7	0.10648166	0.4566397	0.14190835
ENSG000001	ACYP1	0.35856502	0.69382497	0.14198549
ENSG00000(	TRNT1	0.14623259	0.50446567	0.14201549
ENSG000001	TAF5L	0.04922259	0.360767	0.14203437
ENSG000002	DHRS11	0.79426334	0.92434446	0.14208841
ENSG000001	BRWD1	0.26441447	0.6274921	0.14214106
ENSG000001	TRIM56	0.06123258	0.38491421	0.1421448
ENSG000001	TRIP11	0.07865202	0.41446832	0.14222728
ENSG000001	FANCC	0.11052642	0.46324664	0.14225326
ENSG000001	SLC9B2	0.25301187	0.61912837	0.14228517
ENSG00000(	PHRF1	0.69528429	0.88225469	0.14231962
ENSG000001	MFN1	0.12284137	0.47958052	0.14233552
ENSG000001	NVL	0.13820474	0.49898885	0.14236619
ENSG00000(	COL4A4	0.30194897	0.65571024	0.14238463
ENSG000001	JADE3	0.20172077	0.56773237	0.14238939
ENSG000001	CAVIN1	0.39172869	0.71972665	0.1424267
ENSG000001	TERF1	0.10307398	0.45287405	0.14247689
ENSG000001	PGGT1B	0.09358913	0.4384324	0.14266619
ENSG000001	VAMP8	0.15466777	0.51606937	0.14269605
ENSG000001	ORC3	0.16039148	0.52341736	0.14269708
ENSG000002	ACSM4	0.79109626	0.92297405	0.14270437
ENSG000001	METTL15	0.20459934	0.5688754	0.14274317
ENSG000001	GPR171	0.29945682	0.6542516	0.14274761
ENSG000001	EIF2A	0.29371245	0.65039296	0.14275047
ENSG000001	MOCS2	0.26927304	0.63099817	0.14280418
ENSG000002	ZDBF2	0.49001534	0.77995936	0.14282022
ENSG00000(	CNOT4	0.00281898	0.20994948	0.14286561
ENSG00000(	MPHOSPH9	0.24636586	0.61214306	0.14302629
ENSG00000(	EPB41L2	0.0214878	0.2908707	0.14305277
ENSG000001	TMPRSS13	0.21783388	0.58471959	0.1431435
ENSG000001	PLAAT2	0.66486657	0.86898805	0.14318749
ENSG000001	FBXO11	0.07357856	0.40498137	0.1431908
ENSG000001	TFAM	0.13111169	0.4894044	0.14327295
ENSG000001	ZNF19	0.30984171	0.65931585	0.14329466
ENSG000001	DCAF16	0.16726007	0.53173243	0.14330289
ENSG00000(	PFKP	0.0460219	0.3573685	0.14332241
ENSG000001	KPNA3	0.0467797	0.35803149	0.14334282
ENSG000001	ZNF26	0.24462332	0.61059935	0.14336854
ENSG000001	TIGD2	0.25388915	0.61921212	0.14337544

ENSG000001	RASA1	0.11027482	0.46278943	0.14339331
ENSG000001	CLINT1	0.02847953	0.31345545	0.14350815
ENSG00000C	CD74	0.09909513	0.4460792	0.14351283
ENSG000001	NKG7	0.26125962	0.62516213	0.14370549
ENSG000001	SLC17A1	0.69334817	0.88183499	0.14372069
ENSG000001	BLM	0.07627374	0.41062891	0.14372282
ENSG000001	FBXL2	0.24747388	0.61356441	0.14373641
ENSG000001	CNGB3	0.7681327	0.91357827	0.14374648
ENSG00000C	NANS	0.02048846	0.28641584	0.14376764
ENSG000001	KCNMA1	0.56134995	0.81796995	0.14377078
ENSG000001	MGA	0.07969437	0.4152849	0.14379229
ENSG000001	ERH	0.28174785	0.63946601	0.143832
ENSG000001	ZC3H12B	0.31778985	0.66537122	0.14385705
ENSG000001	TTLL10	0.65168286	0.86352007	0.1438641
ENSG000002	INMT	0.68738383	0.88047649	0.14386801
ENSG000001	FAM160B1	0.1399252	0.4999841	0.14395972
ENSG000001	CCDC116	0.58387296	0.82875175	0.14406389
ENSG000001	NEK7	0.24631606	0.61214306	0.14418605
ENSG000001	TENT4A	0.03659063	0.33341755	0.14430384
ENSG000001	TMF1	0.16176112	0.52435922	0.14430445
ENSG00000C	NUP160	0.03328249	0.32398208	0.14431685
ENSG000002	PAGE2	0.71557874	0.89204237	0.14442775
ENSG000001	ZBTB37	0.26634198	0.62912347	0.14444816
ENSG000001	COX17	0.16378847	0.52709285	0.14451849
ENSG000001	TRIM23	0.31063274	0.65949478	0.14455724
ENSG000001	IPPK	0.09576552	0.44171811	0.14460938
ENSG000002	PPP4R3B	0.06098561	0.38464604	0.14461243
ENSG000001	CEP85L	0.40035781	0.72583241	0.14465256
ENSG00000C	SMARCE1	0.20203715	0.56775306	0.1446591
ENSG000001	PSMG4	0.21042936	0.57453195	0.1446856
ENSG000001	SGO2	0.35110587	0.69051672	0.14469703
ENSG000001	CD86	0.06162648	0.38545034	0.14482518
ENSG000001	L1CAM	0.46829183	0.76580263	0.14489028
ENSG000001	TMEM192	0.01880388	0.27889987	0.14490426
ENSG000001	CCDC110	0.76020875	0.91136366	0.14498696
ENSG000001	ACVRL1	0.27898918	0.63787563	0.14516602
ENSG000001	AKAP17A	0.6747176	0.8743925	0.14518708
ENSG000001	FASTKD3	0.21611924	0.58281482	0.1451904
ENSG000001	NIPSNAP2	0.12336153	0.47994951	0.14524029
ENSG000001	STK39	0.13843916	0.49898885	0.14529368
ENSG000001	ERCC6L2	0.20170178	0.56773237	0.14529881
ENSG000001	GOLIM4	0.05803948	0.37863925	0.14537545
ENSG000001	MS4A6A	0.15443309	0.51582229	0.14541622

ENSG00000(	FAT2	0.47165514	0.76694135	0.14543027
ENSG000001	MTMR6	0.10757186	0.45856726	0.14544651
ENSG000001	ICE1	0.05168881	0.36546885	0.14555407
ENSG00000(	STARD3NL	0.08510875	0.42526981	0.14556718
ENSG000001	TTBK2	0.12715803	0.48500467	0.14559489
ENSG000001	MTHFD2L	0.28944928	0.64727721	0.14564161
ENSG000001	SLC38A6	0.23358713	0.60135629	0.14565516
ENSG000001	KRT86	0.72994275	0.89828814	0.14568821
ENSG000001	SRSF3	0.09741641	0.44406238	0.1457113
ENSG000001	CCT2	0.23678152	0.60364584	0.14578308
ENSG000001	CAPN2	0.02202855	0.29231997	0.14589778
ENSG000001	SRC	0.17036233	0.53437545	0.14592867
ENSG000002	C17orf50	0.71645044	0.89251234	0.14594917
ENSG000001	STAG2	0.1815476	0.54632234	0.14594981
ENSG000001	PTK6	0.22815861	0.59645659	0.14598329
ENSG00000(	CLCN6	0.02469194	0.29927225	0.14601232
ENSG000001	AQP11	0.34788439	0.68740599	0.14608689
ENSG000001	NSMCE4A	0.1707556	0.53457173	0.14609849
ENSG000001	SETD7	0.05572355	0.37502119	0.14615135
ENSG000001	CHRNB2	0.44264831	0.74861323	0.14615512
ENSG00000(	POLB	0.14244344	0.50068215	0.14620066
ENSG000001	HNRNPR	0.00546766	0.21833269	0.14621219
ENSG000002	CERS1	0.75012369	0.9080858	0.14621346
ENSG000001	ALOX15	0.41143649	0.72999304	0.14628705
ENSG000001	MLF1	0.48880103	0.7791736	0.14633225
ENSG000001	PTRH1	0.72488114	0.89669145	0.14644887
ENSG000001	CREBL2	0.06728462	0.39569009	0.14645933
ENSG000001	RELCH	0.2040763	0.5688169	0.14653768
ENSG000001	DPH6	0.23303929	0.60037143	0.14655182
ENSG000001	ASF1A	0.2648095	0.6274921	0.14655897
ENSG000001	KALRN	0.15356487	0.51472848	0.14659673
ENSG000001	TTF2	0.07540795	0.40963494	0.14670658
ENSG000001	METTL5	0.28364505	0.64188775	0.14681584
ENSG000001	ADAMDEC1	0.56148938	0.81801019	0.14689025
ENSG000001	DUSP3	0.0506393	0.36298244	0.14690769
ENSG000002	ZNF551	0.18514659	0.550214	0.14693639
ENSG000001	KLRC1	0.51018016	0.79143652	0.14695626
ENSG000001	AMIGO2	0.17970273	0.54477842	0.14698012
ENSG00000(	AFF4	0.05393459	0.37208157	0.14700159
ENSG00000(	CDC14A	0.26926355	0.63099817	0.14700843
ENSG000002	EID1	0.21296495	0.57843959	0.14701486
ENSG00000(	COBLL1	0.28678025	0.64421192	0.14702113
ENSG000001	ZNF354B	0.32742239	0.67217574	0.14705075

ENSG000001	AMMECR1	0.1087195	0.46040635	0.14705361
ENSG000001	TMPO	0.10734649	0.45831177	0.1470637
ENSG000001	CYP4F2	0.72120861	0.89490971	0.14706874
ENSG000001	DPY19L4	0.40644368	0.7275473	0.14710195
ENSG000001	MTR	0.18460662	0.5498217	0.14710245
ENSG000001	CEP78	0.2249767	0.59201197	0.14711268
ENSG000001	PLEKHA2	7.64E-05	0.18011717	0.14715505
ENSG000001	GINS3	0.25390862	0.61921212	0.14728554
ENSG000001	SAMD3	0.21352936	0.57911862	0.14732425
ENSG000001	FBXO4	0.40357349	0.72672584	0.1474134
ENSG000001	TRIM21	0.26565336	0.62853695	0.14742941
ENSG00000C	CDC7	0.26923666	0.63099817	0.14746636
ENSG000001	ZNF430	0.19352522	0.56021911	0.14747603
ENSG000001	ZBTB6	0.22988141	0.59836237	0.14752419
ENSG000001	GPATCH11	0.31671289	0.66414403	0.14753819
ENSG000001	UTP20	0.09355194	0.4384324	0.14754084
ENSG000001	SEC11C	0.27059349	0.63181283	0.14756882
ENSG000001	LCTL	0.58616854	0.82959975	0.14758462
ENSG000001	SRSF1	0.01415814	0.25910565	0.14759608
ENSG000001	GPALPP1	0.20176817	0.56773237	0.14761555
ENSG000001	MOB1A	0.00622969	0.22079879	0.14763407
ENSG000001	TMEM68	0.2302735	0.59855451	0.14773205
ENSG000001	ACSL5	0.00577495	0.21912363	0.14773534
ENSG000001	SLF2	0.04829045	0.360767	0.1478782
ENSG000001	ACBD7	0.75162326	0.908573	0.14793314
ENSG000001	C6orf62	0.01116427	0.2451811	0.14794962
ENSG000001	SLC35A5	0.07886585	0.41446832	0.14799453
ENSG000001	LRP8	0.21782892	0.58471959	0.14802113
ENSG000001	KLHDC1	0.43714803	0.74497757	0.14809267
ENSG000001	XPO4	0.15678266	0.51959295	0.14814211
ENSG000001	UNC93B1	0.25200761	0.61763737	0.1481903
ENSG000002	IGFL2	0.69317481	0.88183499	0.14820309
ENSG000002	ZNF225	0.10757423	0.45856726	0.14828417
ENSG000001	ME3	0.42451202	0.73751355	0.14829032
ENSG000002	N/A	0.69348294	0.88183499	0.1482922
ENSG000001	UCHL5	0.13763988	0.49840739	0.14832382
ENSG000001	TNNT1	0.32844688	0.67259327	0.14836894
ENSG000001	CRACR2A	0.05668815	0.37516454	0.14842507
ENSG000001	FAM102B	0.11035881	0.46290261	0.14850505
ENSG00000C	SOX8	0.2782178	0.63759905	0.14851543
ENSG000001	HSPA14	0.29239014	0.64947398	0.14860922
ENSG000001	GON7	0.74521227	0.90628646	0.14861834
ENSG000001	PTPMT1	0.27176633	0.63323084	0.14862306

ENSG000001	HMG20A	0.09835204	0.4448142	0.14869551
ENSG000001	PRF1	0.22596026	0.59319591	0.1487415
ENSG000001	ABCG8	0.77108701	0.91463929	0.14874178
ENSG000001	AGBL3	0.38971689	0.7188269	0.14876904
ENSG000001	JMY	0.20495882	0.56891466	0.14876987
ENSG000001	ZNF655	0.09103404	0.43488031	0.14877193
ENSG00000(	EXOC1	0.03505089	0.32931219	0.14885086
ENSG000001	CREBRF	0.08949611	0.43356546	0.14886732
ENSG000001	TARS1	0.00972961	0.23800279	0.14887404
ENSG000001	CLCN5	0.02691975	0.30868361	0.14887804
ENSG000001	USP45	0.31981127	0.66679459	0.14887983
ENSG000001	FBXL17	0.06224688	0.38624952	0.14896573
ENSG000001	RAD1	0.12180074	0.47854428	0.14901939
ENSG000001	GAL3ST1	0.66906125	0.87125589	0.14902954
ENSG000001	NNMT	0.79139656	0.92305912	0.14903098
ENSG000001	LCT	0.76352912	0.9121117	0.14903651
ENSG000001	GCC2	0.0967296	0.44304329	0.14907329
ENSG000001	POLE2	0.39424855	0.72144863	0.14909582
ENSG000001	EDEM1	0.02354791	0.29875884	0.14921765
ENSG000001	ZNF436	0.01440818	0.2599395	0.14922071
ENSG000001	TFG	0.00195514	0.19438665	0.14923851
ENSG00000(	ABCC6	0.55155521	0.81323014	0.14925164
ENSG000001	ZNF280D	0.42233039	0.7362799	0.14927705
ENSG000001	LIPA	0.0186367	0.27889987	0.14930217
ENSG000001	MACF1	0.08725449	0.42940825	0.14931266
ENSG000001	OR52L1	0.63129878	0.85334691	0.14934315
ENSG000001	CENPO	0.10135256	0.4496268	0.14937173
ENSG000002	N/A	0.64151241	0.85758579	0.14939512
ENSG000001	RALGAPA1	0.22499882	0.59201197	0.14941944
ENSG000001	CPSF4L	0.76459112	0.91243616	0.14950605
ENSG000001	USP12	0.13882872	0.49941019	0.14960493
ENSG000001	MTRF1	0.20013279	0.56699578	0.14963529
ENSG00000(	BCAP29	0.04699997	0.35811287	0.14964487
ENSG00000(	ADA2	0.06723666	0.39561184	0.14988518
ENSG000001	PIGK	0.24694091	0.61292467	0.14994498
ENSG000001	GPHN	0.15890209	0.5217239	0.15001705
ENSG000001	MLKL	0.138409	0.49898885	0.15003607
ENSG000001	SDS	0.51407919	0.79390373	0.15005715
ENSG000001	PMM2	0.11609638	0.47160917	0.15011449
ENSG000001	ARID5B	0.10405123	0.45422994	0.15011755
ENSG00000(	CASR	0.77196426	0.91507664	0.15012091
ENSG000001	DPYD	0.06175738	0.38552656	0.15016438
ENSG000001	MIS18BP1	0.11375543	0.46915085	0.15018795

ENSG000001	CCDC66	0.33362719	0.67603951	0.15019442
ENSG000001	HMGN4	0.0396214	0.34093258	0.1502348
ENSG000001	ZXDB	0.15469488	0.51606937	0.15029876
ENSG000001	LANCL1	0.15961292	0.52219103	0.1503486
ENSG000001	CCDC117	0.05163343	0.36535566	0.15040521
ENSG00000C	TAF2	0.03265992	0.32362351	0.15041134
ENSG000001	NAF1	0.07321416	0.40493325	0.15041983
ENSG000001	MED13	0.09346961	0.4384324	0.15046065
ENSG000001	GPR151	0.71464428	0.89175724	0.15046844
ENSG000001	SHD	0.54281555	0.80839296	0.15050063
ENSG000001	CTLA4	0.27507123	0.63499184	0.15053616
ENSG000001	FABP3	0.23067418	0.59855451	0.15070349
ENSG000001	THNSL2	0.29658933	0.65209491	0.15073743
ENSG00000C	LYRM2	0.24027176	0.60709277	0.15080763
ENSG000001	DUSP10	0.0438209	0.35372007	0.15081114
ENSG000001	PPM1L	0.06300222	0.38851766	0.15081751
ENSG000001	NCEH1	0.06957808	0.40004644	0.15081897
ENSG00000C	ZNF37A	0.1230065	0.47983319	0.15090314
ENSG00000C	CREM	0.03533421	0.32934644	0.15090697
ENSG000001	C1QL1	0.70017957	0.88463644	0.15110615
ENSG000001	ZNF24	0.10691714	0.45756817	0.1511233
ENSG000001	WDR31	0.57570933	0.82352953	0.15114282
ENSG000001	WDSUB1	0.05668126	0.37516454	0.15115774
ENSG000001	STAM2	0.07833516	0.41427772	0.15118997
ENSG000001	NDUFAF5	0.24773868	0.61375236	0.15119694
ENSG000001	DUS4L	0.22823088	0.59645659	0.15122122
ENSG000001	PSMA6	0.09476698	0.43988973	0.15126176
ENSG000001	GPR155	0.13314591	0.49200959	0.15144891
ENSG000001	PIN4	0.12862165	0.48681347	0.15145336
ENSG000002	ADSL	0.107538	0.45856726	0.1515305
ENSG000001	SYCP2	0.44405602	0.7491529	0.15154189
ENSG000001	MAN1A2	0.14524056	0.5041195	0.15154707
ENSG000001	SLC37A1	0.04763261	0.35872481	0.15163711
ENSG000001	DHX36	0.03362513	0.32398208	0.15184312
ENSG000001	GTF2H3	0.15956484	0.52219103	0.15188147
ENSG000002	FAM90A26	0.41130381	0.72999304	0.1518835
ENSG000001	BPNT1	0.07456051	0.40721124	0.1518839
ENSG000001	ZNF440	0.07624175	0.41059278	0.15192594
ENSG00000C	FGFR3	0.75223907	0.908573	0.15195313
ENSG000001	WDR17	0.5993282	0.8371724	0.15196313
ENSG000001	KIAA1549L	0.69913939	0.8842289	0.15196872
ENSG000001	FGFR1OP2	0.21777237	0.58471959	0.15203197
ENSG000001	KLHL9	0.15394129	0.51533385	0.15204741

ENSG000001	NUGGC	0.38899062	0.71798902	0.1520921
ENSG000001	RAB3GAP1	0.0098655	0.2387274	0.15210183
ENSG000001	UBE2V2	0.1376331	0.49840739	0.15215367
ENSG000001	GOLGA4	0.11832464	0.47496594	0.15217113
ENSG000001	PCGF5	0.11217156	0.46614115	0.15221274
ENSG000001	KIF5B	0.04443599	0.35479539	0.15234466
ENSG000001	FRS2	0.12445472	0.48173414	0.15242697
ENSG000001	PDGFRB	0.3771565	0.70869476	0.15253513
ENSG000001	DCUN1D4	0.29193269	0.64936748	0.15253879
ENSG000001	N/A	0.65884919	0.86565256	0.1525389
ENSG00000C	HSF2	0.24142326	0.60721888	0.15255426
ENSG000001	ATP1B1	0.17071614	0.53457173	0.15262695
ENSG000002	DOK6	0.41071974	0.72975628	0.15263428
ENSG000001	UBFD1	0.02369747	0.29875884	0.15264201
ENSG000001	CD72	0.09829448	0.4448142	0.15270925
ENSG000001	COMMD1	0.12541581	0.48335545	0.15271467
ENSG000001	INTS2	0.16304674	0.52653704	0.15274854
ENSG00000C	POLR1H	0.7618893	0.91151487	0.15276274
ENSG000002	GAS2L2	0.75105682	0.90844082	0.1527787
ENSG000001	PLEKHA8	0.13805877	0.49898885	0.15285823
ENSG000001	ZWILCH	0.21863956	0.58536368	0.15290666
ENSG00000C	DDX43	0.30263254	0.65591444	0.15291776
ENSG000001	C8orf37	0.40674442	0.7275473	0.15299129
ENSG000001	PRIM1	0.22792887	0.59614707	0.15302986
ENSG000001	GRIP2	0.80295274	0.92807116	0.15309037
ENSG00000C	NOP58	0.26878325	0.63079288	0.15311088
ENSG000001	SNRPD1	0.17922907	0.54453451	0.1531508
ENSG000001	MED28	0.03431114	0.32696964	0.15315557
ENSG000001	TEC	0.06886249	0.39825823	0.15327008
ENSG00000C	IL12RB1	0.06826023	0.39743754	0.15328239
ENSG000001	TNS2	0.35976782	0.69484359	0.15329446
ENSG000001	RNF114	0.00934749	0.23551879	0.15336777
ENSG000001	ZFP62	0.35496008	0.6919142	0.15347944
ENSG000001	RNF6	0.06814689	0.39743754	0.15348269
ENSG000001	MS4A14	0.07605341	0.41035311	0.15349196
ENSG000001	SREK1IP1	0.15484676	0.51610961	0.15350911
ENSG000001	RPS24	0.3134794	0.66161883	0.15351737
ENSG000001	UBE2E2	0.03240166	0.32362351	0.15351836
ENSG000001	PRUNE2	0.26953964	0.6310094	0.15358067
ENSG000002	CHML	0.36617108	0.69855054	0.15362056
ENSG000001	KRT5	0.39771494	0.72387597	0.15367463
ENSG000001	CD36	0.14980201	0.50942953	0.1537039
ENSG000001	IMPACT	0.08709912	0.42907415	0.15371183

ENSG000001	ZXDA	0.23076853	0.59855451	0.15371607
ENSG000001	PEX1	0.152613	0.51346327	0.15376818
ENSG000002	POLR2M	0.23511334	0.60232491	0.15378532
ENSG000001	SNX14	0.15646689	0.51911584	0.15380107
ENSG000001	SNX6	0.02517221	0.30077106	0.15393898
ENSG000002	RAB7B	0.74007927	0.90464694	0.15397294
ENSG00000C	PAX2	0.79005856	0.92280247	0.15399915
ENSG00000C	SENP1	0.01861089	0.27889987	0.15429565
ENSG000001	ARHGAP39	0.47307218	0.76859183	0.15434472
ENSG000001	SSPN	0.25436627	0.61939789	0.15435016
ENSG000001	EID2	0.09259511	0.43753271	0.15438145
ENSG000001	CMC2	0.1703801	0.53437545	0.15440474
ENSG000001	SOCS5	0.13351932	0.49240713	0.15442395
ENSG00000C	PER3	0.22969764	0.59797988	0.15444438
ENSG000001	ANXA2R	0.13193621	0.49032921	0.15444592
ENSG000002	CBWD6	0.11335774	0.46854321	0.15448077
ENSG000001	SNRNP27	0.04121377	0.34708923	0.15450177
ENSG000002	RPA4	0.55999101	0.81699589	0.15450707
ENSG000001	GSKIP	0.1989688	0.56598606	0.15459342
ENSG000001	ZBTB14	0.19368615	0.56056904	0.15468171
ENSG000001	USP30	0.00392786	0.21403539	0.15471676
ENSG000002	PPIAL4C	0.65420708	0.86439091	0.15471828
ENSG000001	TAF13	0.1019184	0.45128146	0.15474305
ENSG000001	XIAP	0.00799445	0.23002982	0.1547851
ENSG000001	FN1	0.38815536	0.71704113	0.15482734
ENSG000001	TRANK1	0.34220578	0.68310736	0.15484044
ENSG000001	SMCO2	0.68564562	0.87981649	0.15486971
ENSG000001	ODAD3	0.60999375	0.84290213	0.15488705
ENSG000001	NOL8	0.11018521	0.46278943	0.15492831
ENSG00000C	IDI1	0.0940703	0.43929464	0.15493725
ENSG000001	THAP5	0.20332804	0.56872308	0.15494928
ENSG000001	FAM98B	0.10004386	0.44762396	0.15501727
ENSG000001	CYP2U1	0.10395589	0.45398919	0.15507804
ENSG000001	EXOC8	0.15537314	0.51717788	0.1551488
ENSG00000C	SNX13	0.16686472	0.53125145	0.15515032
ENSG000001	ZNF131	0.1258172	0.48382419	0.15519813
ENSG000001	CYSLTR2	0.05421628	0.37256839	0.15522212
ENSG00000C	ETV1	0.77610891	0.91690146	0.15522327
ENSG000001	ERAP1	0.0061125	0.22079879	0.15523213
ENSG000001	ZFP69	0.24158613	0.60721888	0.15524217
ENSG000001	CEP57	0.29562487	0.65140093	0.15524366
ENSG000001	RPS3A	0.59580748	0.83576612	0.15533634
ENSG000001	KTI12	0.10479747	0.45514301	0.15535568

ENSG000001	SCO1	0.0161567	0.26906292	0.15537044
ENSG000001	CUL2	0.02586052	0.30449397	0.15542373
ENSG00000C	AP5M1	0.11858807	0.47520101	0.15542775
ENSG000002	TRIM10	0.59139531	0.83318747	0.15543215
ENSG000001	IQCB1	0.16800519	0.53240547	0.15544312
ENSG000001	TMEM252	0.30661985	0.65735608	0.1555493
ENSG000001	C5orf63	0.32023502	0.66722134	0.15563135
ENSG000002	N/A	0.51109979	0.79171125	0.15576479
ENSG000001	DBT	0.0955341	0.4413663	0.15591498
ENSG000001	LCORL	0.17799629	0.54295053	0.15594065
ENSG000001	ANXA4	0.02058389	0.28641584	0.15601556
ENSG000001	CSTF2T	0.00037774	0.18856694	0.15604044
ENSG00000C	SLK	0.09255137	0.43753271	0.15605469
ENSG000002	ZNF658	0.43129792	0.74126857	0.1560643
ENSG00000C	PUS7	0.07502902	0.40825944	0.1560716
ENSG00000C	LAG3	0.27175859	0.63323084	0.1560802
ENSG000001	HAVCR2	0.02635323	0.30699826	0.15611729
ENSG000001	FBLN2	0.41553039	0.73221244	0.15611994
ENSG000001	MRGPRF	0.66164221	0.86736018	0.15617359
ENSG000001	RFX7	0.13456786	0.49389205	0.15625688
ENSG000001	REEP3	0.09796023	0.44463533	0.15626936
ENSG000001	SRD5A1	0.03484006	0.32855052	0.15627252
ENSG000001	LYPD6B	0.71363343	0.89153258	0.1563274
ENSG000002	ZNF670	0.41021425	0.72953436	0.15637732
ENSG00000C	RBL1	0.15807035	0.52089921	0.15650761
ENSG000001	IFT57	0.29486138	0.6512057	0.15652536
ENSG000001	PPM1B	0.09701837	0.44311879	0.15656644
ENSG000001	TADA1	0.15405027	0.51533385	0.15663388
ENSG000002	FAM243A	0.60247318	0.83852406	0.15666345
ENSG000001	ZNF248	0.31642898	0.66372668	0.1566819
ENSG000001	PLEKHA1	0.27958119	0.63854799	0.15670787
ENSG000002	HLA-DPA1	0.48351055	0.77585394	0.15671618
ENSG000001	KCTD12	0.10771945	0.4588904	0.1567194
ENSG000001	ERBB2	0.12045665	0.47724625	0.15677439
ENSG000001	NAA15	0.05430069	0.37256839	0.15679504
ENSG000001	CALHM1	0.72358971	0.89594062	0.1568342
ENSG000001	KLF12	0.31796509	0.66556648	0.15684906
ENSG000001	ACER2	0.36575059	0.69837111	0.15687148
ENSG000001	NKRF	0.07502149	0.40825944	0.15695335
ENSG000001	C12orf29	0.36086546	0.69535997	0.15695584
ENSG000001	RTN4IP1	0.23920995	0.60547587	0.15704288
ENSG000002	ZHX1-C8orf7	0.71473204	0.89175724	0.15713657
ENSG000001	NDUFAF4	0.30068543	0.65493729	0.15716627

ENSG00000(	EHD2	0.3689001	0.70186257	0.1572006
ENSG000001	SPRY3	0.56189206	0.81801588	0.15726766
ENSG000001	IL23R	0.6023435	0.83852406	0.1573811
ENSG000002	MAP10	0.21115137	0.57528121	0.1573848
ENSG000001	ZNF621	0.231789	0.599371	0.1574443
ENSG000001	BRIX1	0.24105415	0.60721888	0.1574506
ENSG000002	CHURC1	0.09395328	0.43929464	0.15745175
ENSG000001	CCDC121	0.23059644	0.59855451	0.15746219
ENSG000001	CRYBA4	0.68914342	0.88074719	0.15752067
ENSG000001	NAPG	0.09592129	0.44194893	0.15755606
ENSG000001	CISD2	0.12706071	0.48495433	0.15761764
ENSG000001	DEXI	0.02552272	0.30337654	0.15764149
ENSG000001	RNF144A	0.02954465	0.31711354	0.15765756
ENSG000001	TRPC6	0.32747021	0.67217574	0.15768598
ENSG000001	ZNF169	0.11136367	0.46472866	0.15769097
ENSG000001	SNRPG	0.32210586	0.66822528	0.15775144
ENSG000002	PLD5P1	0.36265222	0.69656832	0.15775393
ENSG00000(	QSER1	0.06479759	0.39055819	0.1577583
ENSG000001	ZFP14	0.22915507	0.59761256	0.15778792
ENSG000001	PSD3	0.31744195	0.66472853	0.15780829
ENSG000001	GSTCD	0.23666692	0.60364584	0.15782735
ENSG000001	SCAF11	0.01415192	0.25910565	0.15787791
ENSG000001	XPNPEP1	0.00464596	0.21772133	0.15794995
ENSG000001	ZNF136	0.08294181	0.42228867	0.15800199
ENSG000001	GFM1	0.00710671	0.22670251	0.15804023
ENSG000001	RCN2	0.32670852	0.67199978	0.15805259
ENSG000001	WDR44	0.02776493	0.3113392	0.15805435
ENSG000001	SDE2	0.03654236	0.33341755	0.15807074
ENSG000001	KIAA1549	0.74475854	0.90628646	0.15808062
ENSG000001	ANXA5	0.04628114	0.35738124	0.15808971
ENSG000001	STRIP2	0.33539474	0.67708232	0.1581019
ENSG000001	NR4A1	0.16831574	0.53253292	0.15811403
ENSG000001	KHDRBS3	0.44454017	0.74937694	0.15814875
ENSG000002	SPIN3	0.20625277	0.56956674	0.15818749
ENSG000001	ZBED2	0.503394	0.7866046	0.15822378
ENSG00000(	SNX10	0.17297158	0.53752663	0.15823604
ENSG000002	PPP2R2A	0.01248971	0.2548969	0.15834323
ENSG000001	ZBTB5	0.15788417	0.52077869	0.15834779
ENSG00000(	ORC6	0.3016679	0.65553823	0.15836248
ENSG000001	ZNF571	0.3616486	0.69588937	0.15839325
ENSG000001	DUSP6	0.04346811	0.35324909	0.15843898
ENSG00000(	MTMR11	0.06051187	0.38425153	0.158454
ENSG000001	ZMYM4	0.01511184	0.26260224	0.15845639

ENSG000001	OTUD3	0.17736031	0.54223298	0.15845978
ENSG000000	DDX1	0.06233086	0.38624952	0.1585404
ENSG000001	ZFAND4	0.02450829	0.29927225	0.15861101
ENSG000001	PRDM5	0.3186442	0.66595775	0.1586168
ENSG000001	EMC2	0.16897756	0.53296205	0.15867218
ENSG000001	ATP13A3	0.07138147	0.40241675	0.15873188
ENSG000002	LRCOL1	0.70945014	0.88968414	0.1588175
ENSG000001	SEPTIN8	0.16552245	0.52885285	0.15884466
ENSG000001	CLIC3	0.19501228	0.56261804	0.15885556
ENSG000000	DNAJC10	0.151587	0.5126678	0.15887821
ENSG000001	SLC4A5	0.1819512	0.54661139	0.1588794
ENSG000000	ERLEC1	0.12778322	0.48588488	0.15889108
ENSG000001	TOGARAM1	0.14895418	0.50895811	0.1589248
ENSG000001	TATDN1	0.31539745	0.66324235	0.15904018
ENSG000000	LGALS14	0.65243541	0.86379349	0.15917416
ENSG000001	LRRC27	0.09683051	0.44304329	0.15918435
ENSG000001	ADAR	0.21464164	0.58023513	0.15925676
ENSG000001	CCDC136	0.27673912	0.63628052	0.15930334
ENSG000001	DNAJC27	0.16140254	0.52426078	0.15931213
ENSG000001	FXYD2	0.68728197	0.88042583	0.15931676
ENSG000001	API5	0.03022091	0.3189525	0.15934582
ENSG000001	SOCS6	0.22337544	0.59081204	0.15935117
ENSG000001	LEXM	0.49482751	0.78124152	0.15941405
ENSG000001	CLASP2	0.0319048	0.3227302	0.15945704
ENSG000001	POMZP3	0.71257603	0.89116324	0.15946667
ENSG000000	ERCC8	0.33945706	0.68088502	0.15958646
ENSG000001	ZNF277	0.16154387	0.52427832	0.15970892
ENSG000000	CHMP2B	0.02147072	0.2908707	0.15985143
ENSG000001	TSEN15	0.13641245	0.49639823	0.15987468
ENSG000001	C2orf49	0.14258541	0.50068323	0.1598831
ENSG000001	SYNE1	0.03951454	0.34091267	0.15992929
ENSG000001	SCRN1	0.0080989	0.23111033	0.15996665
ENSG000001	NDRG4	0.27750844	0.63709819	0.16004395
ENSG000001	SYNCRIP	0.02173037	0.29183808	0.16016494
ENSG000001	TM4SF19	0.57810061	0.82523365	0.16016901
ENSG000001	PGBD2	0.15352268	0.51472848	0.16018493
ENSG000001	ESCO1	0.21973507	0.58681553	0.16024196
ENSG000002	ARHGEF35	0.48339293	0.77585394	0.16026787
ENSG000001	POT1	0.2225481	0.5899614	0.16029218
ENSG000001	PTGER1	0.71635829	0.89246602	0.16033883
ENSG000001	GZMH	0.33401186	0.67622825	0.16040008
ENSG000001	MRPL3	0.26154828	0.62545387	0.16064465
ENSG000001	RWDD3	0.39800715	0.72424544	0.16067415

ENSG000001	HRH3	0.75870618	0.91112183	0.16070025
ENSG000001	DIPK2B	0.60333062	0.83883394	0.16074279
ENSG000001	CERS6	0.12330495	0.47994951	0.1607779
ENSG00000C	INTS13	0.12082509	0.47724625	0.16081917
ENSG000001	SPTLC2	0.10465565	0.45514301	0.16091627
ENSG000001	STAP2	0.36276523	0.69656832	0.16092737
ENSG000001	PEX2	0.13116711	0.4894044	0.16099246
ENSG000001	PRELID3B	0.0775089	0.41275686	0.16107579
ENSG000001	DNPEP	0.03190378	0.3227302	0.1610945
ENSG000001	STK32B	0.19773854	0.56540555	0.1611948
ENSG000001	ARL5A	0.22182938	0.58912768	0.16121592
ENSG000001	JHY	0.38176297	0.7107769	0.16125286
ENSG000001	CEP290	0.32036016	0.66729534	0.16126604
ENSG00000C	HDAC9	0.17114717	0.53523048	0.16126642
ENSG000001	OTULIN	0.01872601	0.27889987	0.16134322
ENSG000001	BORA	0.18472001	0.5498217	0.1614375
ENSG000001	TLR10	0.10596879	0.4561473	0.16144581
ENSG00000C	FAM114A2	0.02686932	0.30854109	0.16145851
ENSG000001	TRMT1L	0.00592275	0.22079879	0.16146968
ENSG000002	NFKBIL1	0.61910197	0.84711589	0.16147967
ENSG000001	FLACC1	0.12011699	0.47648351	0.16148798
ENSG000001	UBP1	0.02049549	0.28641584	0.16150217
ENSG00000C	KDM5D	0.21522211	0.58097447	0.16155562
ENSG000001	IDH3A	0.03638311	0.33300599	0.16164193
ENSG000001	SLC35D1	0.12670958	0.48454628	0.16164564
ENSG000001	RAP2C	0.08296489	0.42228867	0.16165794
ENSG000001	TRA2A	0.03191077	0.3227302	0.16175477
ENSG000001	BTN1A1	0.46428418	0.76363273	0.16184969
ENSG000001	B4GALT6	0.35340003	0.69126085	0.16186117
ENSG000001	POLI	0.24804352	0.61432011	0.1618913
ENSG000001	RAP1B	0.10367081	0.45355618	0.1619207
ENSG000001	TSNAX	0.09248337	0.43746768	0.16194135
ENSG000002	C2orf15	0.39148341	0.71964297	0.16201386
ENSG000001	CARF	0.36297152	0.69664198	0.16202377
ENSG00000C	IKZF5	0.14140114	0.50055578	0.16207711
ENSG00000C	RASGRF1	0.6666435	0.87009558	0.16213466
ENSG000001	TIMELESS	0.03667493	0.33341755	0.16214551
ENSG000001	TRDMT1	0.16843927	0.53253292	0.16231395
ENSG000001	PSMD10	0.05279086	0.36946775	0.16239391
ENSG000001	CCR5	0.19274357	0.55865356	0.16240928
ENSG000001	WDR75	0.19937483	0.56600138	0.16243581
ENSG000001	ZUP1	0.16438601	0.52781009	0.16253792
ENSG000001	OR1L8	0.74174869	0.90543757	0.16258523

ENSG000001	ATF1	0.09789396	0.44463533	0.16261725
ENSG00000(	GPR137B	0.002708	0.2093801	0.16262823
ENSG000001	OPA1	0.04996247	0.36170272	0.16271542
ENSG000001	ARMC1	0.10416693	0.45454407	0.16275916
ENSG000001	GMFB	0.21094659	0.57523547	0.1627638
ENSG00000(	CLNS1A	0.14394525	0.50220003	0.16280153
ENSG000001	SLC30A8	0.66935173	0.87127337	0.16281104
ENSG000001	MIOS	0.03965065	0.34099737	0.16283992
ENSG000001	BRSK1	0.32585695	0.67152421	0.1630325
ENSG000001	PNPLA8	0.12111267	0.47724625	0.16313407
ENSG000001	PHEX	0.23546766	0.60269577	0.16316828
ENSG000001	ANXA1	0.23439249	0.6021681	0.16318019
ENSG000001	BBS4	0.0789745	0.4147264	0.16326557
ENSG000001	TMEM156	0.12694055	0.48480458	0.16330291
ENSG000001	TM2D1	0.06619387	0.39350613	0.16341212
ENSG000001	CPSF6	0.09803501	0.44463533	0.16341813
ENSG000001	TRMT10A	0.32099839	0.66766831	0.16359162
ENSG000002	HACD2	0.08030875	0.41684053	0.16359973
ENSG000001	GPR61	0.58281458	0.82828537	0.16363735
ENSG00000(	FOXC1	0.67099565	0.87218472	0.16367875
ENSG000001	TIMM8B	0.34580233	0.68548313	0.16370907
ENSG000001	CCP110	0.04780514	0.35895177	0.16378009
ENSG000001	CNFN	0.60251649	0.83852406	0.16378197
ENSG000001	PZP	0.42389437	0.73732623	0.16383635
ENSG000001	IREB2	0.04006712	0.34275311	0.16387929
ENSG000001	RBIS	0.17313949	0.53759345	0.1638858
ENSG000001	EMC9	0.07119425	0.40236027	0.16389108
ENSG000001	MRPS18C	0.15611786	0.51859362	0.16399069
ENSG000001	FRA10AC1	0.229633	0.59790748	0.1640193
ENSG000001	ZSCAN12	0.25282516	0.61880044	0.16405326
ENSG000001	BLOC1S5	0.08287467	0.42228867	0.16405693
ENSG000001	ZNF41	0.07814168	0.41427772	0.16408651
ENSG000001	LIN9	0.33471999	0.67675278	0.16412508
ENSG000001	FAM199X	0.06208261	0.38624952	0.16414279
ENSG00000(	SYNE2	0.00811725	0.23121033	0.16415596
ENSG00000(	ERP44	0.02178089	0.29183808	0.16422586
ENSG000001	HELZ	0.00866072	0.23436355	0.16425592
ENSG000001	KLHL29	0.18598879	0.55069275	0.16426139
ENSG000002	EFCAB9	0.66683592	0.87009558	0.16428491
ENSG000002	CLEC12B	0.19928802	0.56600138	0.16431694
ENSG000002	GANC	0.01145504	0.24590889	0.16436622
ENSG000001	RFC3	0.11113494	0.46435874	0.16440348
ENSG00000(	CACNG4	0.70578284	0.88780148	0.16441506

ENSG00000(	ACAT1	0.1775866	0.5423686	0.16442311
ENSG00000(	SEMA3F	0.72011736	0.89454101	0.16443118
ENSG000001	PDIA6	0.00696865	0.22577227	0.16448367
ENSG000001	RPS6KB1	0.08278807	0.42228867	0.16449623
ENSG000001	SLC35E4	0.17563757	0.54061531	0.16453914
ENSG000001	OSMR	0.36096296	0.69535997	0.16457786
ENSG000001	AIG1	0.00902377	0.23513527	0.1646437
ENSG00000(	LAMA3	0.76198582	0.91151487	0.16470903
ENSG000001	PTPN22	0.12479	0.48220256	0.16473427
ENSG000002	CCDC200	0.02798352	0.31159655	0.16478771
ENSG00000(	CHI3L2	0.11119715	0.46449914	0.16482855
ENSG000001	TMCO1	0.2790674	0.63787563	0.16496991
ENSG000001	RP2	0.15853041	0.52129996	0.16503322
ENSG000001	ZNF35	0.0505958	0.36286396	0.16506246
ENSG000002	GAN	0.14015276	0.5002427	0.16513021
ENSG000001	ARMCX5	0.15362285	0.51472848	0.16515616
ENSG00000(	HS3ST1	0.19871816	0.56598606	0.16518773
ENSG000001	NSMCE2	0.28781228	0.64495772	0.16519102
ENSG000001	ZNF141	0.24843655	0.61459845	0.16519612
ENSG000001	ARMCX3	0.02411434	0.29927225	0.16520849
ENSG000001	MFSD14B	0.02425597	0.29927225	0.16530278
ENSG000001	CACNB2	0.17214187	0.53630279	0.16530555
ENSG00000(	ZNF200	0.05145845	0.36502002	0.16548146
ENSG000001	THAP2	0.21265118	0.57808354	0.16550404
ENSG000001	TAF5	0.02653547	0.30699826	0.16559768
ENSG00000(	STYK1	0.31632562	0.66372668	0.16563214
ENSG000001	EIF1AX	0.15874207	0.52165454	0.16565903
ENSG000001	TMPRSS15	0.65138923	0.8634351	0.16565993
ENSG000001	MED7	0.15407456	0.51533385	0.16566087
ENSG00000(	MFAP3	0.10294	0.45259595	0.1657173
ENSG000001	ANKHD1	0.05492105	0.37354464	0.16572268
ENSG000002	LILRA2	0.40933089	0.72902686	0.16576898
ENSG000001	ALDH1A1	0.10611674	0.4561473	0.16578278
ENSG000001	MIS12	0.13814821	0.49898885	0.16581391
ENSG000001	CRBN	0.20546291	0.56910631	0.16581635
ENSG000001	ALG13	0.17045038	0.53440428	0.16589312
ENSG000001	TMEM263	0.23127685	0.5991557	0.16592245
ENSG000001	ZFP1	0.18132452	0.54622749	0.16592741
ENSG000001	FDX1	0.12183914	0.47854428	0.1659873
ENSG000002	TMEM244	0.70092238	0.88498263	0.16601272
ENSG000001	OSTC	0.17856629	0.54366789	0.16605087
ENSG000001	IL23A	0.16095706	0.52386068	0.16608916
ENSG000001	B3GAT1	0.30744916	0.65762361	0.16615477

ENSG000001	DUSP19	0.30349488	0.6561378	0.16622843
ENSG00000C	MSH4	0.55765561	0.81587881	0.16624478
ENSG000001	DLEU7	0.07851305	0.41446832	0.16632365
ENSG000001	DERL1	0.00020514	0.18011717	0.16632943
ENSG000002	COLQ	0.06831608	0.39743754	0.16633397
ENSG000001	PRKX	0.00434818	0.21723525	0.16647042
ENSG000001	RAD54B	0.63118523	0.8533351	0.16647688
ENSG00000C	MOV10L1	0.62024699	0.84779446	0.16652283
ENSG000001	GRK3	0.00203802	0.19934507	0.16656812
ENSG000001	BRD7	0.00791624	0.23002982	0.16657457
ENSG000001	GTPBP8	0.13890105	0.49941019	0.16659263
ENSG000001	KCTD9	0.10258966	0.45201921	0.16661659
ENSG000001	SLC7A6OS	0.02656762	0.30699826	0.16666831
ENSG000001	ZADH2	0.07838356	0.41429486	0.1667598
ENSG000001	BORCS7	0.10004695	0.44762396	0.16679785
ENSG000001	BACE2	0.11826238	0.47496594	0.16682447
ENSG000001	PDE12	0.01084306	0.24206404	0.16683707
ENSG000001	LRCH1	0.02876303	0.31345545	0.16683812
ENSG000001	TPD52L1	0.60822884	0.84156895	0.16684084
ENSG000001	ZNF619	0.07854417	0.41446832	0.16692448
ENSG000001	UTP23	0.04641425	0.35774922	0.16692615
ENSG000001	SCYL2	0.05751988	0.3770373	0.16692688
ENSG000001	ZKSCAN8	0.09983179	0.447493	0.16697299
ENSG000001	EEA1	0.16883076	0.53296205	0.16702455
ENSG000001	RHEB	0.01574811	0.26642326	0.16703705
ENSG000001	ZNF548	0.21968738	0.58681553	0.16708188
ENSG000002	OR3A2	0.63771565	0.85624651	0.16710702
ENSG000001	ZNF649	0.18421813	0.54954065	0.16723779
ENSG000001	ZNF624	0.29731153	0.65274757	0.16724347
ENSG00000C	GCFC2	0.17697794	0.54177807	0.16728996
ENSG000001	MRPL32	0.28670331	0.64421192	0.16732526
ENSG000001	GABPB1	0.013618	0.25910565	0.16740806
ENSG000002	SPON1	0.33117242	0.67443203	0.16742498
ENSG000001	STARD8	0.04651909	0.35774922	0.16748692
ENSG000002	B3GALT9	0.25553303	0.62030905	0.16750471
ENSG000001	NAPB	0.0532486	0.36981204	0.16757473
ENSG000001	VANGL2	0.73455107	0.9010812	0.16758455
ENSG000001	ERCC4	0.0448743	0.35545023	0.16759637
ENSG000002	NSUN6	0.22577534	0.59319591	0.16764708
ENSG000001	ATG12	0.0705965	0.40156223	0.1676628
ENSG000001	SMCHD1	0.1879782	0.55211256	0.16774358
ENSG000001	GIMAP4	0.04869336	0.360767	0.16774383
ENSG000001	ZNF765	0.17419954	0.53927128	0.1679185

ENSG000001	DYRK2	0.19508963	0.56274122	0.1679247
ENSG000001	ZNF596	0.33257457	0.67523724	0.16800813
ENSG000001	ABHD10	0.10805607	0.45960368	0.16807163
ENSG000001	HIVEP3	0.06811702	0.39743754	0.16817565
ENSG00000C	CLDND1	0.12723545	0.48507209	0.168219
ENSG000001	WSB2	0.01409664	0.25910565	0.16823713
ENSG000001	CLEC4D	0.22157556	0.5889073	0.16827942
ENSG000001	C2CD2	0.0418949	0.34903589	0.16831787
ENSG00000C	GCLC	0.0423033	0.35009105	0.16836444
ENSG000001	RNPC3	0.13138499	0.4894044	0.16840085
ENSG000001	ZMYM6	0.07066513	0.40156223	0.16840376
ENSG00000C	ALOX5	0.71217729	0.89093319	0.16852625
ENSG00000C	LAMB1	0.28612969	0.64387022	0.16862108
ENSG000001	GIMAP5	0.20668288	0.57024809	0.16864099
ENSG00000C	ASNS	0.2860839	0.64387022	0.16865379
ENSG000001	BPIFB4	0.54651493	0.81061139	0.16870874
ENSG000001	AVPR1A	0.40040791	0.72583241	0.16871028
ENSG000001	ENOPH1	0.13589855	0.49574736	0.16871575
ENSG000001	ZNF852	0.23958688	0.60622911	0.1687426
ENSG000001	IDH2	0.0928065	0.43753271	0.16875578
ENSG000001	NCBP1	0.0663598	0.39396126	0.16878232
ENSG000001	MAP2K6	0.15667307	0.51948144	0.16879609
ENSG000001	ZNF91	0.23184593	0.599371	0.16887
ENSG000001	TYW3	0.20436584	0.5688169	0.16889006
ENSG000001	ZNF226	0.22281391	0.59037688	0.16894016
ENSG000001	B4GALT4	0.06996756	0.4002892	0.16894437
ENSG00000C	VTA1	0.0219518	0.29231997	0.16896886
ENSG000001	ZNF239	0.37964514	0.70965485	0.1690426
ENSG000001	TNR	0.63114598	0.8533351	0.16906416
ENSG000001	SEC23IP	0.0006424	0.18856694	0.16907029
ENSG000001	CHAC2	0.36683308	0.69892851	0.16908704
ENSG000001	RFPL3	0.6420352	0.85807272	0.16917016
ENSG000001	TUSC1	0.36503156	0.69795307	0.16921125
ENSG000001	CACNA1D	0.34001259	0.68135285	0.16933285
ENSG000001	DTWD2	0.14607019	0.50430399	0.16945054
ENSG000001	FANCF	0.03890997	0.33839374	0.16946605
ENSG000002	H3C6	0.11889326	0.47560231	0.16947521
ENSG000001	NAA20	0.12091507	0.47724625	0.16947559
ENSG00000C	DNAJB11	0.01376186	0.25910565	0.16949485
ENSG00000C	MYOM2	0.67890539	0.87594571	0.16953111
ENSG000001	CD52	0.29535315	0.6512539	0.16953333
ENSG00000C	ST7L	0.06511284	0.39148468	0.16954345
ENSG000001	APLF	0.27356084	0.63403495	0.16955838

ENSG000001	SWAP70	0.00371145	0.21208655	0.16959718
ENSG000001	DIAPH2	0.0174673	0.27297069	0.16962998
ENSG000002	N/A	0.75584511	0.9104345	0.16964933
ENSG000001	FAM71D	0.6122443	0.8443189	0.16965489
ENSG00000C	BAK1	0.10569747	0.4561473	0.16971496
ENSG000001	AHNAK2	0.33273012	0.67523724	0.16972561
ENSG000001	ZNF626	0.14945182	0.50925123	0.16977203
ENSG000001	SLC66A3	0.03196096	0.3227302	0.16986509
ENSG00000C	PSME1	0.11466664	0.47028092	0.16988178
ENSG000001	KDM1B	0.02447335	0.29927225	0.16990151
ENSG000002	MRPS17	0.0845226	0.42469888	0.16993424
ENSG000001	COA7	0.07345179	0.40497004	0.16995625
ENSG000001	GRPEL2	0.17035354	0.53437545	0.16996697
ENSG000001	GLOD5	0.63007761	0.85293934	0.17006087
ENSG000001	OSBPL10	0.12807731	0.48599936	0.17008622
ENSG000001	USP16	0.2178909	0.58473377	0.1700902
ENSG00000C	MEF2C	0.01722986	0.27243096	0.17010325
ENSG00000C	RIOK2	0.18947837	0.55423532	0.17023554
ENSG000001	PPP4R2	0.1442171	0.50250064	0.17025959
ENSG000001	MTSS1	0.02152054	0.2909484	0.17032392
ENSG00000C	PALB2	0.0635936	0.38981851	0.17032911
ENSG00000C	XPO1	0.04285389	0.35185871	0.17042184
ENSG000001	GCSH	0.56871433	0.82037292	0.17046422
ENSG000001	CCR2	0.0549564	0.37354464	0.17065398
ENSG000001	PPEF2	0.49984822	0.78433741	0.17079572
ENSG000001	GPR63	0.64558584	0.8600572	0.17080356
ENSG000002	H2AB1	0.55662507	0.81537572	0.17082836
ENSG000002	CDKL4	0.68110161	0.87679749	0.17091477
ENSG000001	USO1	0.02442533	0.29927225	0.17092381
ENSG000001	RHBDD1	0.00069875	0.18856694	0.1709414
ENSG00000C	CD200	0.20157007	0.56773237	0.17094339
ENSG000001	SETBP1	0.04152034	0.34755298	0.17108674
ENSG000001	NPC2	0.12090724	0.47724625	0.17117241
ENSG000001	UTY	0.28563275	0.64333814	0.17129105
ENSG000001	VBP1	0.17456695	0.53968842	0.17134815
ENSG00000C	SLC4A8	0.00615812	0.22079879	0.17142458
ENSG000001	FFAR3	0.43756026	0.74532247	0.17142899
ENSG000001	TMEM45A	0.57089527	0.82133852	0.17144655
ENSG00000C	WDR7	0.01632288	0.27000801	0.17149311
ENSG000001	PANX1	0.04888734	0.360767	0.17163342
ENSG000001	HSD17B7	0.03076386	0.31953017	0.17169347
ENSG000001	QKI	0.06106947	0.38464604	0.17182754
ENSG000001	MTX3	0.24516536	0.61129452	0.17186749

ENSG000001	FBXO28	0.02609189	0.30552739	0.1719306
ENSG000001	PLEKHF2	0.09241484	0.43746768	0.17194189
ENSG000001	TOMM40L	0.02051532	0.28641584	0.171976
ENSG00000C	CLK1	0.24420549	0.61002532	0.17197724
ENSG000002	PPIAL4G	0.71811848	0.89332947	0.17205937
ENSG000001	NUTM2A	0.32054391	0.66735114	0.17206523
ENSG000001	SLC9A2	0.70386757	0.88657108	0.17206991
ENSG000001	MFSD8	0.06858656	0.39796501	0.17210735
ENSG000001	PTX3	0.08799122	0.43050213	0.17211835
ENSG000001	TTC37	0.12892237	0.4871454	0.17212518
ENSG000001	FAM91A1	0.06770331	0.39689346	0.17218138
ENSG000001	EIF4A2	0.16268005	0.52586821	0.17223994
ENSG000001	MYO6	0.21928283	0.58662135	0.17224048
ENSG000001	CSNK1G3	0.18108087	0.54610144	0.17236091
ENSG000001	S1PR5	0.14857468	0.50798213	0.17238449
ENSG000001	SPIN1	0.04673339	0.35792926	0.17239243
ENSG000002	OR9A4	0.65441192	0.86439091	0.1723931
ENSG000001	LDLR	0.13912738	0.49975817	0.17239352
ENSG000001	CYP17A1	0.71251394	0.89116324	0.17250396
ENSG000001	DNAJC13	0.0247157	0.29927225	0.17254338
ENSG000001	RPS4Y1	0.22452363	0.59186495	0.1725823
ENSG000001	UBN2	0.06136313	0.38542193	0.17259254
ENSG000002	SCGB2B2	0.62432959	0.84979805	0.17265321
ENSG000001	INPP4B	0.30469501	0.65635638	0.1727761
ENSG000001	ANGEL2	0.13685764	0.49714946	0.17280206
ENSG000001	CYP4V2	0.138586	0.49898885	0.17286091
ENSG000001	MAGT1	0.00875698	0.23436355	0.17293048
ENSG00000C	HHAT	0.11621043	0.47160917	0.17299958
ENSG000001	PRSS38	0.49813507	0.78286233	0.17303669
ENSG000001	ICE2	0.15486149	0.51610961	0.1730375
ENSG00000C	NDUFB2	0.11141853	0.46472866	0.1731243
ENSG000001	L3HYPDH	0.19558215	0.5632015	0.17314903
ENSG000001	ANP32E	0.11992242	0.47604098	0.17319204
ENSG000001	CFAP58	0.16774551	0.53220434	0.17322831
ENSG000001	EPS8	0.18260861	0.54695002	0.17327216
ENSG000001	GNPTAB	0.05263625	0.36895324	0.17331968
ENSG000001	ZNF804A	0.33261331	0.67523724	0.17336865
ENSG000001	ANKMY2	0.09357593	0.4384324	0.17337355
ENSG000001	ZNF585A	0.04117613	0.34701527	0.17344134
ENSG000001	N6AMT1	0.21366152	0.57911862	0.17344613
ENSG000001	CLCC1	0.0662693	0.3937119	0.17347278
ENSG000001	EGR4	0.62142838	0.84862344	0.17350763
ENSG00000C	USP33	0.0138401	0.25910565	0.17364404

ENSG000001	HPS5	0.00568009	0.21875144	0.1737735
ENSG00000(	PKN2	0.19460575	0.56194444	0.17377692
ENSG000001	SPDYE5	0.26253083	0.62594907	0.17383432
ENSG000001	IRF8	0.01858561	0.27889987	0.17388912
ENSG000002	ZNF578	0.50587349	0.78868674	0.17391272
ENSG00000(	PCNP	0.12281114	0.47957781	0.17391619
ENSG000001	HOOK1	0.39918913	0.72496091	0.17393939
ENSG000001	BOD1	0.12220254	0.47887061	0.17398088
ENSG000001	NLGN4X	0.73315544	0.89987515	0.17416123
ENSG000001	VAMP4	0.14076088	0.50051652	0.17421672
ENSG000001	ARL14EP	0.22764128	0.59568276	0.17436418
ENSG000001	SPSB4	0.69235791	0.88158966	0.17440574
ENSG000001	PIGF	0.21427151	0.58004777	0.17444469
ENSG000001	IFNE	0.59709813	0.83621548	0.17446423
ENSG000001	TENT4B	0.03184379	0.3227302	0.17447855
ENSG000001	PTRHD1	0.1608057	0.52384569	0.17448555
ENSG000001	FBXO3	0.16728321	0.53173243	0.17449929
ENSG000001	LURAP1L	0.69572704	0.88233383	0.17454548
ENSG000001	SUZ12	0.09378281	0.43895977	0.17465378
ENSG00000(	DPEP1	0.58314365	0.82845829	0.17466334
ENSG000001	NSUN3	0.02609423	0.30552739	0.17469963
ENSG000002	ETV3L	0.70810367	0.88904065	0.17473327
ENSG000001	ZNF720	0.08991905	0.43377006	0.17487337
ENSG000002	CCDC7	0.14106839	0.50055066	0.17490155
ENSG00000(	UBA5	0.12530945	0.48335545	0.1749976
ENSG000001	LPAR3	0.69622149	0.88256705	0.17505354
ENSG000001	CHRNB1	0.06523652	0.39169239	0.17506027
ENSG000001	IL6ST	0.13353475	0.49240713	0.17507887
ENSG000002	H2AC18	0.39573368	0.72221286	0.17509751
ENSG000001	SNX16	0.24150771	0.60721888	0.17510307
ENSG000001	SCAI	0.30959915	0.65916845	0.17515013
ENSG000001	IKZF4	0.00870915	0.23436355	0.17520213
ENSG000001	TOX	0.09081429	0.4347065	0.17522809
ENSG000001	STRBP	0.13659009	0.49671069	0.17524154
ENSG000001	C16orf87	0.13378784	0.4925946	0.17529389
ENSG000001	ITPR1	0.02772953	0.3113392	0.17550075
ENSG00000(	RNF19A	0.10321146	0.45287405	0.17552298
ENSG000001	DHRS2	0.69134482	0.88131945	0.17564563
ENSG000001	KCNA3	0.20147888	0.56773237	0.17565848
ENSG000001	GFOD1	0.0732791	0.40494581	0.17578472
ENSG000001	EXOC6	0.0304464	0.3191802	0.17578945
ENSG000002	PAM16	0.53482478	0.80406944	0.17590433
ENSG00000(	ANKRD44	0.05510399	0.37354464	0.17594042

ENSG000001	GZMB	0.19872572	0.56598606	0.17595527
ENSG000001	SORT1	0.13397037	0.4925946	0.17601672
ENSG000001	ZNF654	0.09861225	0.44547647	0.17607246
ENSG000001	FAM167A	0.34717169	0.68653332	0.17611919
ENSG00000C	MTMR2	0.05252308	0.36870613	0.17612438
ENSG000001	ENPP5	0.27308912	0.63381189	0.17612877
ENSG000001	SMC5	0.11317011	0.46821512	0.17617123
ENSG00000C	ZBTB11	0.18693072	0.55108473	0.17617714
ENSG000002	RAB6D	0.54349969	0.8084284	0.17620341
ENSG000001	TNFRSF10A	0.05648192	0.3750908	0.17621219
ENSG000001	HDAC8	0.06772169	0.39689346	0.17622273
ENSG000001	PATL1	0.05879996	0.38067583	0.17633416
ENSG000001	ATAD2B	0.06100094	0.38464604	0.17638768
ENSG000001	HIBCH	0.09610817	0.44215472	0.17641391
ENSG000001	ZNF146	0.16289342	0.52634836	0.17645171
ENSG000001	CYB5A	0.11987758	0.47604098	0.17650172
ENSG000001	GPR68	0.02316843	0.29724668	0.176531
ENSG00000C	NEBL	0.44719777	0.75099423	0.17665115
ENSG000001	TRAPPC10	0.04738284	0.35865951	0.17667739
ENSG000001	HLA-DQB1	0.03562819	0.33014896	0.17670339
ENSG000001	ADARB1	0.01927094	0.28088168	0.17673662
ENSG000001	SCLT1	0.03344681	0.32398208	0.17673767
ENSG000001	RPL26	0.27748787	0.63709819	0.17673944
ENSG00000C	BTN3A1	0.04743208	0.35865951	0.17674303
ENSG000001	COX18	0.04858796	0.360767	0.17676947
ENSG000001	TRAPPC6B	0.26711424	0.62929976	0.17677014
ENSG000001	LILRB1	0.57196065	0.82170634	0.17683181
ENSG000001	MDFIC	0.15250373	0.5133091	0.17687817
ENSG000001	GOSR2	0.02685718	0.30854109	0.17702596
ENSG000001	ANKDD1A	0.15976145	0.52225624	0.17705333
ENSG000001	CENPC	0.07701321	0.411743	0.17719451
ENSG000002	N/A	0.11699178	0.4728891	0.17727934
ENSG000001	TKTL2	0.7458336	0.90651247	0.17729393
ENSG000001	PDE4D	0.09089008	0.43482093	0.17736365
ENSG000001	ATP13A5	0.66183195	0.86736018	0.17746826
ENSG000001	PNO1	0.01534072	0.26409578	0.17750655
ENSG000001	FBXO5	0.19113312	0.55685897	0.17751344
ENSG000001	PM20D2	0.14001374	0.4999841	0.17751783
ENSG000001	ATE1	0.08893322	0.43309175	0.17751923
ENSG00000C	TYMP	0.27222979	0.63335652	0.17761372
ENSG000002	ZBTB10	0.22939658	0.59783342	0.1776406
ENSG000001	KIAA1586	0.24095352	0.60721888	0.17766523
ENSG000001	PLPP5	0.05616761	0.37502119	0.17769551

ENSG000001	CDYL2	0.02101696	0.28773382	0.17780401
ENSG000001	COL8A2	0.16857912	0.53253292	0.17783834
ENSG000002	ATP10A	0.06477746	0.39055819	0.17784928
ENSG00000C	RBM7	0.02231262	0.29312398	0.17797686
ENSG000001	CLEC3B	0.49448671	0.78124152	0.17801316
ENSG000001	CRB3	0.34206784	0.68310736	0.17811365
ENSG000001	GPX2	0.3124306	0.66090624	0.17831749
ENSG000001	B3GNT2	0.064519	0.39055819	0.17833273
ENSG000001	MICU2	0.04544649	0.35545023	0.17835344
ENSG000001	AEBP2	0.07871556	0.41446832	0.17836808
ENSG000001	PROM2	0.6690731	0.87125589	0.17839956
ENSG000001	DDX21	0.00016911	0.18011717	0.17842233
ENSG000001	GEN1	0.10265262	0.45201921	0.17846088
ENSG00000C	ARVCF	0.07213931	0.40373033	0.17846934
ENSG000001	KNTC1	0.17695805	0.54177807	0.17848569
ENSG000001	PLA2G7	0.09449248	0.43981122	0.17849995
ENSG000001	MPV17L	0.61730161	0.84647633	0.17852224
ENSG000001	TSPAN1	0.56461073	0.81898782	0.17855607
ENSG000001	C11orf42	0.36045639	0.69534637	0.17856514
ENSG000001	PLEKHG1	0.10048866	0.44829276	0.17867082
ENSG000001	KLHL5	0.0048375	0.21772133	0.1787142
ENSG000001	SLC20A1	0.00163666	0.19292382	0.17874718
ENSG000001	RHOT1	0.02464373	0.29927225	0.17876497
ENSG000001	FANCB	0.47093687	0.76688416	0.17879799
ENSG000001	WTIP	0.44518512	0.74940749	0.1788668
ENSG000001	OR3A1	0.66849576	0.87106699	0.17891046
ENSG000001	IER3IP1	0.17829325	0.54332516	0.17891243
ENSG000001	A2M	0.40447642	0.72672584	0.17892806
ENSG00000C	GADD45B	0.19105665	0.55674567	0.17895772
ENSG00000C	PAG1	0.01773708	0.27425066	0.17897315
ENSG000001	RAB11FIP2	0.20522694	0.56903516	0.17897324
ENSG000001	MTMR9	0.06699687	0.39524277	0.17898194
ENSG000001	KLRB1	0.31266782	0.66104002	0.17911766
ENSG000001	PNRC2	0.07289476	0.40435477	0.17922622
ENSG000001	FEM1C	0.03782685	0.33525901	0.17924215
ENSG000001	ZNF813	0.12588508	0.48382419	0.17928663
ENSG000001	TEP1	0.00334691	0.2114129	0.17929091
ENSG000001	CLOCK	0.08077615	0.41778956	0.17930117
ENSG000002	ARHGEF38	0.36894595	0.70186257	0.17934223
ENSG000001	KCNMB1	0.11617375	0.47160917	0.17934871
ENSG000001	MBNL2	0.18515547	0.550214	0.17956106
ENSG000001	CAPZA2	0.14095878	0.50051652	0.17957412
ENSG000001	RIMS2	0.47409184	0.76912287	0.17967966

ENSG000001	SCAPER	0.00642419	0.22290736	0.17969945
ENSG00000C	SLC30A9	0.09314791	0.43758758	0.17974022
ENSG000001	CKS2	0.35347037	0.69131515	0.17978597
ENSG000001	LRRC8E	0.6794547	0.87599698	0.17985548
ENSG00000C	LRRC40	0.24385974	0.60953672	0.18000692
ENSG000002	PSMB9	0.47683673	0.77130883	0.18006541
ENSG000002	TIMM23B	0.12360091	0.48039028	0.1801068
ENSG000001	ZBTB8A	0.57231976	0.82182269	0.18010879
ENSG000001	ABCB10	0.07218521	0.40373033	0.18012053
ENSG000002	ETFRF1	0.11534399	0.4710215	0.18012667
ENSG000001	KLF11	0.0533884	0.37010565	0.18014999
ENSG00000C	PDE6C	0.33886393	0.68041384	0.18018122
ENSG000001	DNAJC21	0.05390053	0.37208157	0.18019523
ENSG000001	PDIA3	0.00790865	0.23002982	0.18019921
ENSG000001	STAMBPL1	0.15360228	0.51472848	0.18022896
ENSG000001	FEM1B	0.04081982	0.34551949	0.18025473
ENSG000001	SUV39H2	0.13607381	0.49591061	0.18025605
ENSG000001	ERI2	0.14149199	0.50055578	0.180299
ENSG000001	AIMP1	0.11208879	0.46614115	0.18030892
ENSG000001	ATP6V1C2	0.16479005	0.52807074	0.18044066
ENSG00000C	RAB27B	0.1122703	0.46634252	0.18050748
ENSG000001	MAPK12	0.26565821	0.62853695	0.18054624
ENSG000001	STXBP4	0.35433052	0.69167785	0.1805571
ENSG000001	BOC	0.6917169	0.88144775	0.18055974
ENSG000001	MTERF3	0.17037134	0.53437545	0.18057171
ENSG000001	BDNF	0.65733434	0.86522797	0.18058643
ENSG00000C	METTL1	0.06774431	0.39689346	0.18059862
ENSG000001	EMP1	0.19798228	0.56540555	0.18066682
ENSG000001	CFAP97	0.18061875	0.54541688	0.18067503
ENSG000001	AVPR2	0.40461079	0.72672584	0.18068811
ENSG000001	FZD3	0.30540927	0.65688103	0.1807981
ENSG000001	TMTC4	0.11829114	0.47496594	0.18081422
ENSG00000C	GPATCH2	0.1630495	0.52653704	0.18083385
ENSG000001	RABGGTB	0.121124	0.47724625	0.18084392
ENSG000001	GALNT8	0.43699588	0.74497757	0.18088189
ENSG000001	GIMAP8	0.04213821	0.34997262	0.18090005
ENSG000001	SCRN3	0.03362686	0.32398208	0.18101166
ENSG000001	GBP2	0.2796788	0.63854799	0.18110652
ENSG000001	USP46	0.08833752	0.43150913	0.18114851
ENSG000001	ZNF12	0.13749775	0.49827463	0.18117177
ENSG000001	DZIP3	0.23677398	0.60364584	0.18123934
ENSG000001	CGRRF1	0.18556765	0.55033096	0.18126439
ENSG000001	E2F5	0.10685173	0.45740881	0.18127832

ENSG000001	SPR	0.20373974	0.56880022	0.18135956
ENSG00000C	HSD17B2	0.71392997	0.89153258	0.18137328
ENSG000001	ZNF431	0.1005333	0.44832716	0.18152702
ENSG000001	GTF3C3	0.1279664	0.48591919	0.18153584
ENSG00000C	BTBD1	0.02664042	0.30699826	0.18155967
ENSG000001	ZBTB1	0.20352419	0.56880022	0.18157348
ENSG000001	SLC25A28	0.05981943	0.38277429	0.18160127
ENSG000001	PSMA5	0.02047477	0.28641584	0.18165584
ENSG000001	VPS54	0.0493328	0.360767	0.18175747
ENSG000001	PREPL	0.18235856	0.54678908	0.18179815
ENSG000002	MICAL3	0.01397344	0.25910565	0.1818147
ENSG00000C	HLTF	0.178828	0.54395471	0.18184352
ENSG000002	ARHGEF25	0.60013046	0.83754165	0.18195808
ENSG00000C	LIMCH1	0.6634992	0.86831922	0.18198498
ENSG000001	TSTD2	0.09752168	0.44422866	0.18199232
ENSG000001	RCHY1	0.07234102	0.40373033	0.18202625
ENSG000001	MOB3B	0.02225571	0.29312398	0.18216705
ENSG000001	VPS36	0.08687065	0.42885945	0.18220431
ENSG000002	TBCE	0.04340161	0.3530621	0.18220773
ENSG000001	RRAS2	0.18499542	0.55004041	0.18230055
ENSG00000C	THUMPD1	0.03431399	0.32696964	0.1823663
ENSG000001	ANKRD46	0.19527146	0.56280385	0.18236941
ENSG000002	ZNF595	0.14846901	0.50794171	0.18248485
ENSG000001	SACS	0.31733677	0.66470815	0.18249704
ENSG000001	C11orf58	0.10283812	0.45239298	0.18252826
ENSG00000C	ZNF532	0.0928581	0.43753271	0.18259594
ENSG00000C	CYLD	0.0067832	0.22347774	0.18270524
ENSG000001	ABCD3	0.08206654	0.42066352	0.18271676
ENSG000001	NUP42	0.11606013	0.47160917	0.18276019
ENSG000002	CKLF	0.11874152	0.47554586	0.18277253
ENSG000001	DCLK2	0.61490682	0.84533626	0.18277591
ENSG000001	TMEM126A	0.15963843	0.52219103	0.18279713
ENSG000001	PDSS2	0.021928	0.29231997	0.18284886
ENSG00000C	GALC	0.0042883	0.21614597	0.18285991
ENSG000001	HINT3	0.01592643	0.26742239	0.18306413
ENSG000001	ROCK2	0.00154576	0.19292382	0.18310511
ENSG000001	YEATS4	0.22670668	0.59419473	0.18312199
ENSG00000C	AGK	0.05906376	0.38096018	0.18312329
ENSG000002	ETDC	0.6063074	0.8399124	0.18325774
ENSG000001	AKAP5	0.28471213	0.64286898	0.1833107
ENSG000001	PEA15	0.00302164	0.20994948	0.18331742
ENSG000001	AGPAT3	0.01083601	0.24206404	0.18349922
ENSG000001	DYNC1LI2	0.09633216	0.44215472	0.18351217

ENSG000001	ANKRD13C	0.13620553	0.49597876	0.18354191
ENSG000002	SELENOP	0.39331911	0.72063539	0.1837063
ENSG000001	CSTF3	0.01131071	0.24556914	0.18373695
ENSG000001	TUBA8	0.32649797	0.67185873	0.18374462
ENSG000001	P2RY12	0.13363089	0.49240713	0.18374918
ENSG00000(	PEX3	0.15411805	0.51533385	0.1837678
ENSG000001	GM2A	0.04984811	0.36170272	0.18383434
ENSG000001	NTAQ1	0.09498759	0.44053513	0.18394834
ENSG000001	DMTF1	0.07961647	0.4152849	0.18396518
ENSG000002	MTRNR2L8	0.38304124	0.71198657	0.18398024
ENSG000001	ZNF235	0.16473358	0.52807074	0.18398276
ENSG000001	KDM6A	0.01860856	0.27889987	0.18403177
ENSG000001	RTCB	0.03713112	0.3336458	0.18407355
ENSG000001	TMEM128	0.1337348	0.4925946	0.18409662
ENSG000001	PSMA2	0.09968672	0.44746644	0.18412091
ENSG000001	C12orf45	0.11413071	0.46962187	0.1841923
ENSG000001	PRIMPOL	0.10714695	0.45794815	0.18428859
ENSG000001	PSMD14	0.09921104	0.4464772	0.18432291
ENSG000002	ZNF737	0.17429973	0.53937579	0.18435928
ENSG000001	GSTT2B	0.53984187	0.80698777	0.18437929
ENSG000001	TAB3	0.08218662	0.4209666	0.18441187
ENSG000001	ZNF490	0.05313145	0.36981204	0.1844331
ENSG000001	ZNF782	0.18440554	0.54968745	0.18450291
ENSG000001	ZNF45	0.03523628	0.32931219	0.18453528
ENSG000001	ADCY7	0.06152824	0.38542193	0.18457508
ENSG000002	APOBEC3D	0.0846311	0.4247775	0.18467879
ENSG000001	BOLA2B	0.69329021	0.88183499	0.18468498
ENSG000001	TASL	0.07576675	0.41015711	0.18468732
ENSG00000(	KCNG1	0.11016626	0.46278943	0.18477882
ENSG000001	PRKAG2	0.03588177	0.33046641	0.18480447
ENSG000001	STYX	0.07240885	0.40374402	0.18489733
ENSG000001	COX7A2	0.136735	0.4969038	0.18490832
ENSG000001	WFDC12	0.6793876	0.87598276	0.18496244
ENSG000001	SAR1B	0.07140082	0.40241675	0.18505046
ENSG000001	TIPRL	0.01201912	0.25192551	0.18508889
ENSG00000(	AP4E1	0.02370664	0.29875884	0.18512793
ENSG00000(	DNM1L	0.04385732	0.35372007	0.18512829
ENSG000001	MSANTD2	0.12139551	0.47772636	0.18514262
ENSG00000(	CDC27	0.00544012	0.21833269	0.18518841
ENSG000001	INPP5F	0.0566211	0.37516454	0.18519616
ENSG000001	ZNF449	0.07974597	0.4152849	0.18549563
ENSG00000(	PIK3C2A	0.06782801	0.39701639	0.18551102
ENSG000001	MUC1	0.38291818	0.71198657	0.18551868

ENSG000001	CD200R1	0.13235748	0.49081838	0.18560684
ENSG00000(	ZNF800	0.02393824	0.29875884	0.18562581
ENSG000001	PRKAB2	0.08543687	0.42579452	0.18563034
ENSG000002	N/A	0.56118316	0.81785019	0.18564336
ENSG00000(	NNAT	0.26397056	0.62728666	0.18565947
ENSG000002	EEF1AKMT2	0.13208712	0.49054292	0.18570428
ENSG000002	IFI30	0.08098181	0.41809272	0.18588103
ENSG000001	ZSCAN29	0.01378442	0.25910565	0.18594012
ENSG000001	KTN1	0.1371273	0.49766113	0.18595746
ENSG000001	PRSS37	0.64160187	0.85763477	0.18600851
ENSG00000(	DOP1A	0.04733581	0.35865951	0.18603382
ENSG000001	STAC3	0.06910913	0.39896748	0.18607593
ENSG000001	ZNF529	0.15587243	0.51809634	0.18616563
ENSG000001	MDH1B	0.68883282	0.88059791	0.18624094
ENSG000001	FUT3	0.71735567	0.89309227	0.18626295
ENSG000001	ARHGAP5	0.1601343	0.5230958	0.18634506
ENSG000001	ZNF354C	0.32616136	0.67170284	0.18640481
ENSG000001	COG5	0.00930847	0.23551879	0.18641248
ENSG000001	GART	0.00930766	0.23551879	0.18645415
ENSG000001	ADAT2	0.20757958	0.57126603	0.18648061
ENSG000002	LIN52	0.08933726	0.43342334	0.18660292
ENSG000001	DSCC1	0.30131808	0.65546137	0.18661666
ENSG000001	GK	0.21746946	0.58412764	0.18662938
ENSG000001	SKA2	0.1458609	0.50430399	0.18667024
ENSG000001	RBM12B	0.17597457	0.54095022	0.18675147
ENSG000001	NLN	0.02214446	0.29303962	0.18676659
ENSG000001	SLC2A12	0.68932396	0.88074719	0.18679096
ENSG000001	STX11	0.2639804	0.62728666	0.18680385
ENSG000001	PRKD1	0.59633658	0.83586991	0.18682191
ENSG000001	ATAD1	0.17024509	0.53437545	0.18682449
ENSG000001	ADAL	0.08829178	0.43141549	0.1868495
ENSG00000(	TPD52	0.1680217	0.53240547	0.18692717
ENSG000001	CENPL	0.20361776	0.56880022	0.18698786
ENSG00000(	ZMYND11	0.11703043	0.47292758	0.18700233
ENSG000001	KIF3A	0.1753006	0.54013466	0.18701975
ENSG000001	KRCC1	0.15328506	0.5145523	0.18706158
ENSG000001	OLFML1	0.4023762	0.72669629	0.1871067
ENSG000001	ODR4	0.14752546	0.50620393	0.18716848
ENSG000001	UHMK1	0.04759254	0.35871889	0.187263
ENSG000001	OXTR	0.30268568	0.65591444	0.18727531
ENSG000001	TMEM220	0.29379722	0.65039296	0.18729003
ENSG000001	UTP15	0.06465285	0.39055819	0.18734583
ENSG000001	GNPNAT1	0.07108545	0.40194888	0.18737635

ENSG000001	TCAF1	0.02388704	0.29875884	0.18751995
ENSG000001	TWSG1	0.09565609	0.4413663	0.18752763
ENSG000001	KCNQ4	0.41762597	0.73321581	0.18754354
ENSG000001	TNFAIP3	0.01250069	0.2548969	0.18771875
ENSG000001	MRPS30	0.03291978	0.32362351	0.18772455
ENSG000002	TEX54	0.56525312	0.81917305	0.18777576
ENSG000001	VSX1	0.6039481	0.83883394	0.18778387
ENSG000001	EEF1E1	0.40543455	0.72703815	0.18797989
ENSG000001	TVP23B	0.14703514	0.5054701	0.18800841
ENSG000001	ACBD5	0.02550012	0.30334587	0.18803543
ENSG000001	ELL3	0.65810627	0.8654563	0.18807782
ENSG000001	LRRC37B	0.00625681	0.22079879	0.1880962
ENSG000001	MAD2L1BP	0.02592822	0.30450132	0.1880983
ENSG000002	FPGT-TNNI3I	0.53249443	0.80280163	0.18826926
ENSG000002	IGLL5	0.30147821	0.65547284	0.18828078
ENSG000001	NUDT21	0.1057276	0.4561473	0.18829015
ENSG000002	N/A	0.5683251	0.82037292	0.1883395
ENSG000001	C1QA	0.38616044	0.71513897	0.188392
ENSG000001	SFMBT2	0.03347145	0.32398208	0.1884319
ENSG000001	OTUD7A	0.51072396	0.79143652	0.1884762
ENSG000001	NSG2	0.69151146	0.88137846	0.18849134
ENSG000001	HSFX1	0.57954825	0.82610173	0.18865599
ENSG00000C	HSD17B6	0.49069113	0.7800506	0.18868648
ENSG000001	UBE2J1	0.0274224	0.3110643	0.1886953
ENSG000001	CDC37L1	0.16580494	0.52885285	0.18875162
ENSG000001	ZNF84	0.2383048	0.60449226	0.18875392
ENSG000001	NUP35	0.0665108	0.39445505	0.18878251
ENSG000001	RAB29	0.00518526	0.21833269	0.188786
ENSG00000C	ZZZ3	0.15430358	0.51573226	0.1888916
ENSG000001	PCDH1	0.21635764	0.58303866	0.18896003
ENSG000002	ZNF845	0.17827255	0.54332516	0.18897091
ENSG00000C	ATG5	0.13965939	0.49975817	0.18897393
ENSG000001	A1BG	0.3293937	0.67311302	0.18898121
ENSG000001	BAZ1A	0.06041122	0.38406301	0.18899466
ENSG000001	MIB1	0.15239201	0.5133091	0.18903808
ENSG000001	ZNF540	0.28894722	0.64680191	0.18906827
ENSG000001	U2SURP	0.09391958	0.43929464	0.18908428
ENSG00000C	EED	0.02038141	0.28641584	0.1891726
ENSG000001	NADK2	0.08937197	0.43342334	0.18926467
ENSG000001	IL2RB	0.03185012	0.3227302	0.18929362
ENSG000001	PTER	0.12444878	0.48173414	0.18930525
ENSG000001	GNL3	0.2017545	0.56773237	0.18935619
ENSG000001	DPY19L1	0.01996972	0.28393023	0.18937613

ENSG000001	GIMAP6	0.01541427	0.26455656	0.18944162
ENSG000001	NAB1	0.09798062	0.44463533	0.18944651
ENSG000001	TRA2B	0.0396014	0.34093258	0.18961027
ENSG000001	NDUFA4	0.32119332	0.66766831	0.18963024
ENSG000001	DDC	0.61304096	0.84505867	0.18963635
ENSG000002	HLA-C	0.24963424	0.61563048	0.18977545
ENSG00000C	UHRF1BP1	0.00602762	0.22079879	0.18978575
ENSG000002	C1QTNF9	0.57024005	0.82075948	0.18989752
ENSG000001	CCDC18	0.11354753	0.46882009	0.18989778
ENSG000001	C1GALT1C1	0.06069519	0.38451341	0.18993104
ENSG000001	CENPI	0.31690597	0.66422665	0.18996374
ENSG000001	PDIK1L	0.16726769	0.53173243	0.18997619
ENSG000001	SMG1	0.04625472	0.35738124	0.19014983
ENSG000001	GTPBP2	0.06433156	0.39043354	0.19028425
ENSG000001	LATS1	0.05849481	0.3800398	0.19028609
ENSG000001	OGN	0.64254122	0.85846637	0.19034974
ENSG000001	GPRC5D	0.68865175	0.88057199	0.19039802
ENSG000002	ZNF550	0.28216697	0.64012112	0.19039831
ENSG000001	ZBTB21	0.04628766	0.35738124	0.19040948
ENSG000001	ZNF391	0.18675656	0.55108473	0.19043012
ENSG000001	ARSK	0.1925677	0.55834317	0.19046336
ENSG000001	FOLR2	0.05639804	0.3750908	0.19051729
ENSG00000C	SIKE1	0.09584116	0.44171811	0.190549
ENSG000001	ZNF708	0.20683657	0.57036149	0.19077869
ENSG000001	GLCE	0.05773967	0.37757512	0.19080839
ENSG000001	PSMA3	0.12320423	0.47994951	0.19081707
ENSG000001	TTC21B	0.06967515	0.40004644	0.19083925
ENSG000001	ATP5F1C	0.14659456	0.50510677	0.19085003
ENSG000001	UBA7	0.05502172	0.37354464	0.1908583
ENSG00000C	IFT80	0.30217778	0.65591444	0.19086744
ENSG000001	FOXD4L3	0.698654	0.88383677	0.19100304
ENSG000001	PRPF40A	0.030962	0.31953017	0.19102931
ENSG000001	DHDH	0.22497932	0.59201197	0.19108053
ENSG000001	RPAP2	0.17891202	0.54410834	0.19122228
ENSG000002	RDH14	0.12552516	0.48354733	0.19125238
ENSG000001	ADAMTSL5	0.20187781	0.56775306	0.19126384
ENSG000002	ZNF254	0.27834254	0.63759905	0.19132736
ENSG000001	GXYLT1	0.06996962	0.4002892	0.19136573
ENSG000001	C9orf72	0.12115691	0.47724625	0.19145413
ENSG000001	ARMT1	0.06429784	0.39043354	0.19145763
ENSG000001	NTRK2	0.64125082	0.8575293	0.19155793
ENSG000001	TYW5	0.0992797	0.44666238	0.19157335
ENSG000001	DCAF17	0.12209318	0.47884711	0.19161049

ENSG000001	KLHL11	0.30778049	0.65762361	0.19165328
ENSG000002	AARD	0.71956388	0.89419553	0.19172528
ENSG000001	RAB30	0.10923978	0.46154732	0.19177368
ENSG000001	CAMKV	0.61886662	0.84711589	0.19178455
ENSG000001	LMBR1	0.01346002	0.25833181	0.19185887
ENSG000001	PPA1	0.10994673	0.46260819	0.19189234
ENSG000001	LPIN1	0.01757517	0.27412882	0.19191231
ENSG000001	SGCE	0.11444167	0.47002354	0.1919571
ENSG000002	EIF3CL	0.55495519	0.81433077	0.19208544
ENSG000001	NIPAL2	0.04699937	0.35811287	0.19209896
ENSG000001	STK3	0.14709283	0.5054701	0.19216718
ENSG000001	NAA30	0.0512774	0.3648515	0.19218439
ENSG000002	TRIM34	0.2350689	0.60232491	0.1923227
ENSG000001	PLCE1	0.15533189	0.51717788	0.19234958
ENSG000001	NOM1	0.01003564	0.24140535	0.19238627
ENSG000002	CRYBB2	0.51537885	0.79470146	0.19239083
ENSG000001	CHD9	0.04673227	0.35792926	0.19240342
ENSG000002	HAUS3	0.11981942	0.47604098	0.19240543
ENSG000001	PPP3CC	0.03053714	0.3191802	0.19243555
ENSG00000C	SYDE2	0.30641127	0.65735608	0.19247618
ENSG000001	SLC26A2	0.14032382	0.5002427	0.19251832
ENSG000001	ALKBH8	0.14560267	0.50423822	0.19268811
ENSG000002	SETSIP	0.44743016	0.75099423	0.19270149
ENSG000001	EDNRB	0.64511328	0.85991395	0.19276887
ENSG000001	QRSL1	0.02374178	0.29875884	0.19295608
ENSG000001	MYO5B	0.26909002	0.63099817	0.1929743
ENSG000001	PPP1R18	0.417658	0.73321581	0.19302128
ENSG000001	METTL4	0.03721889	0.33424953	0.19316518
ENSG000001	TJP2	0.19609665	0.56374315	0.19324966
ENSG000001	CEP135	0.07161325	0.4024868	0.19327833
ENSG000001	LRRC39	0.37642786	0.70798786	0.19328673
ENSG000001	ERRFI1	0.13751115	0.49827463	0.19332799
ENSG000001	MTRR	0.03916036	0.33942602	0.19332826
ENSG000001	N/A	0.61416103	0.8451435	0.19335727
ENSG000001	FNDC9	0.6756003	0.87451032	0.19360898
ENSG000001	AGBL2	0.21000183	0.57424655	0.19376396
ENSG000001	SLC30A1	0.09161576	0.43611772	0.19376491
ENSG000001	GMNN	0.15880195	0.52170151	0.19376694
ENSG000001	TMEM106A	0.00734464	0.22758586	0.19382581
ENSG00000C	TRAM1	0.03090209	0.31953017	0.19383819
ENSG000001	AGL	0.13782855	0.49881219	0.19387497
ENSG000001	MALT1	0.05644928	0.3750908	0.19395688
ENSG000001	LYPLA1	0.02773239	0.3113392	0.19410676

ENSG000001	PTPN2	0.05399496	0.37208157	0.1941174
ENSG00000(	HIVEP2	0.05819097	0.37930423	0.19415371
ENSG000001	TMED7	0.03524941	0.32931219	0.19418825
ENSG00000(	ABCC9	0.47732053	0.77186071	0.1942424
ENSG000001	PCMTD1	0.16018816	0.52312453	0.19447435
ENSG000001	DHRSX	0.66115427	0.86736018	0.19453657
ENSG000001	ARNT2	0.70170091	0.88523111	0.19456345
ENSG000001	ZNF75D	0.04136133	0.34725125	0.19457684
ENSG000001	NFAT5	0.05907263	0.38096018	0.19484673
ENSG000001	NUDT12	0.32631594	0.67170284	0.19488381
ENSG000001	TBATA	0.63425728	0.85499671	0.19509032
ENSG000001	ZNF326	0.09170137	0.43613216	0.19518806
ENSG000001	LRRIQ3	0.51157393	0.79176683	0.19522923
ENSG000001	ANKRA2	0.06630874	0.39380215	0.19532193
ENSG000001	RNFT1	0.10684587	0.45740881	0.19545105
ENSG000001	ASXL3	0.59716869	0.83624217	0.19559774
ENSG000001	CACNA1A	0.30261982	0.65591444	0.19563337
ENSG000001	PUS10	0.06798484	0.39736134	0.19569825
ENSG000001	TMEM19	0.02580992	0.30449397	0.19569999
ENSG000001	TAF1L	0.6099044	0.84290213	0.19572623
ENSG000001	C5orf34	0.38754745	0.71668724	0.19573893
ENSG000001	TASOR	0.03520016	0.32931219	0.19581217
ENSG000001	GZMA	0.26711297	0.62929976	0.19592062
ENSG000001	EOGT	0.09116189	0.43488031	0.19594619
ENSG000001	ACVR1C	0.36194749	0.69592458	0.1959478
ENSG000001	DOCK10	0.01835065	0.27758038	0.19616674
ENSG00000(	VEZT	0.139486	0.49975817	0.19618736
ENSG00000(	KARS1	0.02080357	0.28641584	0.19626093
ENSG000001	BPIFB3	0.59623356	0.83586476	0.19636197
ENSG000001	PRPF4B	0.03104481	0.31953017	0.19637851
ENSG00000(	FAM234B	0.03591337	0.33056998	0.19638823
ENSG000001	ZNF543	0.01611894	0.2689859	0.19644545
ENSG000001	SLC9A9	0.01492789	0.26260224	0.19645239
ENSG000001	WDR43	0.08521676	0.42526981	0.19660152
ENSG00000(	G2E3	0.14804654	0.50706204	0.19665582
ENSG000001	ZNF117	0.25996712	0.62338681	0.19669849
ENSG000002	C17orf114	0.73539551	0.90150345	0.19675538
ENSG000001	LNPEP	0.01087264	0.24206404	0.19678926
ENSG000001	CFL2	0.14159305	0.50055578	0.19681016
ENSG000001	MTERF1	0.08843187	0.43171026	0.19683196
ENSG000001	WDR86	0.1094212	0.46193514	0.19683547
ENSG00000(	EFR3B	0.24371949	0.60937379	0.19690427
ENSG000001	SMARCAD1	0.13174137	0.48982931	0.1969233

ENSG000001	NUS1	0.05549502	0.37471955	0.19698404
ENSG000001	NECTIN3	0.20768371	0.57145576	0.19707018
ENSG000001	ZBTB26	0.14033414	0.5002427	0.19711357
ENSG000002	SUPT4H1	0.16142597	0.52426078	0.19716886
ENSG000001	ERBIN	0.06334329	0.38950527	0.19719556
ENSG000001	CYCS	0.03014963	0.31859418	0.19728281
ENSG000001	TRIM52	0.23170417	0.599371	0.19731882
ENSG000001	ACP4	0.67634495	0.87463061	0.19733974
ENSG000001	MAGOHB	0.18245628	0.54678908	0.19734097
ENSG00000C	TUBE1	0.16877171	0.53293392	0.19734554
ENSG000001	E2F7	0.40747613	0.72768322	0.19737075
ENSG000001	NUP205	0.02640072	0.30699826	0.1974145
ENSG000001	SEC24A	0.00763373	0.22933195	0.19754761
ENSG000001	ITPRID2	0.04781149	0.35895177	0.19757761
ENSG00000C	PACC1	0.01024832	0.24174018	0.19757853
ENSG000001	HSPA8	0.00505793	0.21833269	0.19759172
ENSG000001	ASMT	0.61610453	0.84569598	0.19767688
ENSG000002	ZNF99	0.48684613	0.77767904	0.19769537
ENSG000001	FAM186B	0.37246861	0.7044779	0.19780326
ENSG00000C	HACE1	0.1721077	0.53630279	0.19784369
ENSG00000C	TBX21	0.08092435	0.41792898	0.19785777
ENSG000001	RFESD	0.33589036	0.67749711	0.1978623
ENSG000001	TIMM8A	0.12166754	0.47833312	0.19795568
ENSG000001	BTBD3	0.05702294	0.37665007	0.19798011
ENSG00000C	TGDS	0.1553641	0.51717788	0.19798091
ENSG00000C	LMAN1	0.05439445	0.37297411	0.1980692
ENSG000001	CRYZ	0.26200858	0.62564164	0.19811855
ENSG000001	JRKL	0.21034002	0.57453195	0.1982155
ENSG000001	RIC1	0.03202219	0.32294676	0.19822216
ENSG000001	TSPAN13	0.07344121	0.40497004	0.19824012
ENSG000001	GK5	0.11528214	0.47102068	0.19828431
ENSG000001	SRSF7	0.07173369	0.40260628	0.19829357
ENSG000001	GALNT3	0.07997077	0.41591462	0.19830477
ENSG000001	EPS8L1	0.14279725	0.5010464	0.19836316
ENSG00000C	WSCD2	0.65786467	0.86540022	0.19844727
ENSG000001	RASA4B	0.38231017	0.71146937	0.19847353
ENSG000001	WDR89	0.30558073	0.65695881	0.19850682
ENSG000001	NAA25	0.05050882	0.36271932	0.19853307
ENSG000001	RABL3	0.09777028	0.44463533	0.19860184
ENSG000001	UBE2E1	0.02722327	0.30967858	0.19861042
ENSG000001	RGPD1	0.42258757	0.73645533	0.19867501
ENSG000001	ZHX1	0.03796506	0.3358711	0.19869569
ENSG000001	INO80D	0.04004253	0.34274931	0.19871913

ENSG000001	EBF1	0.09601303	0.44204901	0.19871922
ENSG000001	SLC2A11	0.53977593	0.80698777	0.19874626
ENSG000001	ABHD13	0.11260248	0.46731123	0.19874877
ENSG000001	UBE2Q2	0.06226387	0.38624952	0.19875829
ENSG000001	TBC1D31	0.04269576	0.35185871	0.19877009
ENSG000001	KNL1	0.25130601	0.61695463	0.19888236
ENSG000001	TBC1D4	0.15136085	0.5123298	0.19902287
ENSG000001	FBXO22	0.13873921	0.49927052	0.19909413
ENSG000001	PYHIN1	0.09215957	0.43715892	0.19922797
ENSG000001	SPTSSB	0.27055016	0.63180252	0.19923946
ENSG000001	ERCC6L	0.21235358	0.57755193	0.19925459
ENSG000001	SPATA5	0.10024356	0.44801947	0.19926666
ENSG000001	PAX8	0.248589	0.61459845	0.19942157
ENSG000001	LIPT1	0.08370448	0.42260872	0.19943747
ENSG000001	ELF5	0.69981875	0.88452288	0.199452
ENSG00000(	MYCBP2	0.00477423	0.21772133	0.19951884
ENSG000001	ARL8B	0.00566888	0.21875144	0.19958854
ENSG000001	NUP54	0.05311039	0.36981204	0.19959821
ENSG000001	MMAA	0.04689198	0.35811287	0.19962159
ENSG00000(	PHKA1	0.57845407	0.82533908	0.19966151
ENSG000001	VLDLR	0.0733227	0.40494581	0.19968441
ENSG000001	SENP7	0.23094674	0.59855451	0.1997416
ENSG000001	SLC38A2	0.02967656	0.31805831	0.19988132
ENSG000001	EDRF1	0.02264081	0.29448836	0.19989708
ENSG000001	ZNF846	0.12695628	0.48480458	0.19994812
ENSG000001	CCNL1	0.03557703	0.33014896	0.20004345
ENSG000002	INSYN2B	0.42419074	0.73732623	0.20006847
ENSG000001	COMMD6	0.38487729	0.71322215	0.20020822
ENSG000002	BRD2	0.35205873	0.69071745	0.20021448
ENSG000001	SPPL2A	0.00120178	0.18856694	0.2002407
ENSG000001	DLAT	0.01116071	0.2451811	0.20024848
ENSG000001	MAF	0.0449746	0.35545023	0.20028549
ENSG000001	GRAPL	0.47371956	0.76895276	0.20035942
ENSG000001	USP1	0.06619055	0.39350613	0.20037428
ENSG000001	SAXO1	0.58757565	0.83046863	0.2004474
ENSG000002	C8orf89	0.61165417	0.84379173	0.20045555
ENSG00000(	LNX1	0.12628909	0.48388732	0.20047742
ENSG000001	TBCK	0.03937069	0.34025028	0.20048626
ENSG000001	TMIE	0.4720706	0.76742193	0.20054418
ENSG00000(	RPL26L1	0.26204434	0.62564164	0.20069271
ENSG00000(	RPL31	0.40751515	0.72768322	0.2007978
ENSG000001	BEND6	0.59879907	0.83673325	0.20080273
ENSG000001	WDR41	0.00478117	0.21772133	0.20082258

ENSG000001	FBN1	0.24027562	0.60709277	0.200901
ENSG000002	GNL1	0.72128585	0.89490971	0.20091181
ENSG000001	COL4A2	0.68997143	0.88074719	0.2009159
ENSG000001	POGLUT3	0.09191976	0.43640386	0.20099722
ENSG000001	CYSLTR1	0.03067846	0.31953017	0.20103231
ENSG000001	MRPL50	0.26269332	0.62624377	0.20115529
ENSG00000C	GPBP1	0.03882925	0.33829709	0.20121595
ENSG000001	HNMT	0.21713623	0.583769	0.20125344
ENSG000001	PDCD10	0.21683797	0.58352353	0.20136416
ENSG000001	SRP9	0.12448361	0.48173414	0.20148504
ENSG000001	CDKN2A	0.19184547	0.55723719	0.20150962
ENSG000001	ADO	0.00424936	0.21614597	0.20156473
ENSG000001	MTF2	0.03661206	0.33341755	0.20158109
ENSG000001	KCTD19	0.50521076	0.78792692	0.20164867
ENSG000002	TLCD4-RWDI	0.49745732	0.78267582	0.20166743
ENSG000001	MTMR8	0.14927916	0.50925123	0.20168602
ENSG00000C	ZNF268	0.13963855	0.49975817	0.20170788
ENSG000001	PRDX4	0.12462537	0.48202528	0.20173141
ENSG000001	CEBPZ	0.1540142	0.51533385	0.2018194
ENSG000002	ZNF805	0.16317714	0.52653704	0.20189777
ENSG000001	TMED5	0.04717518	0.35851546	0.20193669
ENSG00000C	DDX24	0.50606063	0.78874871	0.20196971
ENSG000001	KLF15	0.7150774	0.89175724	0.20198327
ENSG000001	STMN2	0.63167972	0.85364823	0.20211986
ENSG000001	SKA3	0.44466747	0.74940749	0.20216307
ENSG000001	FCRL6	0.13607912	0.49591061	0.20220819
ENSG000001	RAP2A	0.13712279	0.49766113	0.20237027
ENSG000001	ANAPC10	0.28507287	0.64333814	0.20242895
ENSG000001	TSPYL5	0.1348469	0.49418805	0.20250496
ENSG00000C	TBX15	0.60580825	0.83946052	0.20252159
ENSG000002	SPDYE21	0.27374952	0.63403633	0.20252264
ENSG000001	ELF1	0.02690112	0.30868361	0.20260873
ENSG000001	ZNF483	0.5275653	0.80062313	0.20262881
ENSG000001	IGIP	0.12340883	0.47994951	0.2026595
ENSG000002	SNX2	0.03254169	0.32362351	0.20266153
ENSG000001	PTPN14	0.40117613	0.72606468	0.20269488
ENSG000001	OMA1	0.0445534	0.35527495	0.20273349
ENSG000001	TPMT	0.03513735	0.32931219	0.20277174
ENSG000001	RPS7	0.42526821	0.73817463	0.20278935
ENSG000001	RPP30	0.02452183	0.29927225	0.20301357
ENSG000001	DIXDC1	0.10354261	0.45340382	0.20308912
ENSG00000C	AKAP11	0.08517833	0.42526981	0.20332751
ENSG000001	CCDC152	0.42240053	0.73628742	0.20341657

ENSG000001	TNIK	0.00948377	0.23577638	0.2034369
ENSG00000(	OSBPL3	0.05972287	0.38277429	0.20353505
ENSG000001	DRAM2	0.04207588	0.34981364	0.2036276
ENSG000001	INSIG2	0.03344048	0.32398208	0.20370795
ENSG000001	CISD1	0.1644842	0.52786788	0.20372574
ENSG000001	L3MBTL3	0.0009631	0.18856694	0.20375858
ENSG000001	CASD1	0.14658758	0.50510677	0.20379081
ENSG000001	COL27A1	0.29901644	0.65423918	0.20381255
ENSG000001	TRIM44	0.00360957	0.21208655	0.20387581
ENSG000001	ZNF841	0.03465948	0.32831054	0.20409167
ENSG000001	CTH	0.26588035	0.62868632	0.20419645
ENSG000001	TANK	0.04886054	0.360767	0.20426832
ENSG000001	KLHL6	5.40E-05	0.18011717	0.2042715
ENSG000001	ZNF528	0.14564269	0.50423822	0.20427327
ENSG000001	TCF12	0.0201028	0.28482469	0.20428115
ENSG000001	C2orf69	0.0707713	0.40156223	0.2042836
ENSG000001	ZNF780B	0.23480412	0.60232491	0.20429365
ENSG000001	ABRAXAS1	0.12824666	0.48630102	0.20430486
ENSG000001	NEUROD2	0.6436791	0.85940463	0.20431088
ENSG000001	KLF8	0.1393848	0.49975817	0.2043148
ENSG000001	THY1	0.56445091	0.81896072	0.20432433
ENSG000001	UHRF2	0.04492756	0.35545023	0.20438315
ENSG000002	YY2	0.22923909	0.59764855	0.20438902
ENSG00000(	FOXP3	0.13579962	0.49561216	0.20440295
ENSG00000(	MAPK6	0.12514332	0.48299311	0.2044671
ENSG000001	L3MBTL4	0.04342573	0.35308145	0.20463029
ENSG000001	LMNB1	0.07571193	0.41015711	0.20463684
ENSG00000(	NLK	0.00279696	0.20994948	0.20500503
ENSG000001	MEX3C	0.09682891	0.44304329	0.20511439
ENSG000001	ZNF770	0.17765373	0.5423686	0.20513663
ENSG00000(	CFH	0.28777072	0.64495772	0.2052175
ENSG000001	TRAT1	0.33911854	0.68041384	0.2052663
ENSG000001	DNAJC24	0.11175218	0.46538092	0.20551954
ENSG000001	ZNF283	0.09167148	0.43611772	0.20553611
ENSG000001	CHRNA6	0.70279746	0.88595232	0.20570046
ENSG000001	WNT9A	0.41109719	0.72995333	0.20579119
ENSG000001	SNX25	0.13718304	0.49775218	0.20580719
ENSG000001	TCF7L2	0.0241681	0.29927225	0.20581298
ENSG000002	OOEP	0.61667764	0.84604942	0.20584334
ENSG000001	MYEF2	0.22146903	0.58882965	0.20587376
ENSG000001	NUDT4B	0.47086905	0.76685063	0.20592627
ENSG000001	DHX15	0.01677019	0.2704224	0.20594454
ENSG000002	N/A	0.6749746	0.87446425	0.20598422

ENSG000001	SOS1	0.00353023	0.21208655	0.20606013
ENSG000001	SLC35G2	0.23623265	0.60336718	0.20609556
ENSG000001	BHLHA15	0.58991211	0.83196412	0.20625442
ENSG000001	CASP1	0.03688208	0.33341755	0.2063477
ENSG000002	IRGM	0.43126548	0.74126857	0.20639679
ENSG000001	MYSM1	0.14091124	0.50051652	0.20642608
ENSG000001	ULBP1	0.64717313	0.86129024	0.20652077
ENSG000001	CTNNA3	0.42373159	0.73732623	0.20664766
ENSG000001	KRBA2	0.10385941	0.45381195	0.20689216
ENSG000001	EMC6	0.45442563	0.75576246	0.20689586
ENSG000001	ZFP3	0.14028131	0.5002427	0.20698577
ENSG000001	ATG3	0.0171022	0.27224356	0.20707226
ENSG000002	LITAFD	0.63577845	0.85527214	0.20708001
ENSG000001	ZRANB2	0.14987651	0.50942953	0.20715642
ENSG000001	HAUS6	0.0751597	0.40883352	0.20727941
ENSG000001	RNLS	0.14628595	0.50451253	0.20731777
ENSG00000C	RORA	0.20110002	0.56773237	0.20750229
ENSG00000C	TRPM7	0.08456313	0.42469888	0.20759618
ENSG000001	CHL1	0.38091628	0.7101777	0.20760244
ENSG000001	TMPRSS7	0.53146608	0.80232066	0.20768508
ENSG000001	MOSMO	0.13223526	0.49076662	0.2077003
ENSG000002	TUBGCP5	0.15514067	0.51682788	0.20782572
ENSG000002	SFT2D2	0.0008453	0.18856694	0.20798652
ENSG000001	RSPO2	0.61749785	0.84653095	0.20806585
ENSG00000C	MYO16	0.37095698	0.70329686	0.2080811
ENSG000001	MIER1	0.04270216	0.35185871	0.20817521
ENSG000002	GOLGA6L10	0.68365341	0.87849752	0.20837745
ENSG00000C	TFRC	0.01177338	0.24956182	0.20841849
ENSG000002	HNRNPUL2-E	0.56600698	0.8197023	0.20850125
ENSG000001	DCAF4L1	0.26915354	0.63099817	0.20850787
ENSG000001	ZNF507	0.19857954	0.56598606	0.2085474
ENSG00000C	AASS	0.37095981	0.70329686	0.20855652
ENSG000001	EPG5	0.00447697	0.21750928	0.20863311
ENSG000001	TBK1	0.03060817	0.31953017	0.20864646
ENSG000001	C6orf120	0.06620337	0.39350613	0.20867774
ENSG000001	ZDHHC17	0.12832032	0.48646673	0.20879355
ENSG000001	THAP9	0.16517097	0.5286578	0.20887069
ENSG000001	PARD6G	0.1184921	0.47516823	0.2089131
ENSG000001	NDFIP2	0.10360617	0.45355618	0.20895213
ENSG000001	ABCE1	0.08933006	0.43342334	0.20910773
ENSG000001	HOPX	0.22604015	0.59329927	0.20911947
ENSG000001	MR1	0.00876676	0.23436355	0.20912136
ENSG000001	SNAPC3	0.047393	0.35865951	0.20927069

ENSG000001	FGF2	0.44504684	0.74940749	0.20933449
ENSG000001	B2M	0.08519592	0.42526981	0.20936257
ENSG000002	HSFX2	0.53510211	0.80413676	0.20939396
ENSG000001	CD83	0.02205397	0.29231997	0.20942362
ENSG000001	BCL6B	0.71302877	0.89118991	0.20942582
ENSG000001	TEX101	0.69559021	0.88231682	0.20942995
ENSG000002	UTP14C	0.00991024	0.23909751	0.20950919
ENSG00000C	SLC4A7	0.19618426	0.5638811	0.20957808
ENSG000001	HTR3A	0.46778304	0.76566463	0.20958619
ENSG000001	SMIM14	0.00336603	0.2114129	0.20961525
ENSG000001	ATP10D	0.01647098	0.27000801	0.20966648
ENSG000001	CGGBP1	0.03692373	0.33341755	0.20968968
ENSG000001	PPP2R2B	0.05713427	0.37665007	0.209694
ENSG000001	CLN5	0.05344396	0.37020885	0.20969924
ENSG000001	ZNF597	0.02174247	0.29183808	0.20972379
ENSG00000C	PRDM1	0.03135146	0.32055029	0.2097566
ENSG00000C	ACSL4	0.0656338	0.39264465	0.20976318
ENSG000001	POLR3D	0.00358317	0.21208655	0.20984514
ENSG000001	HSPH1	0.03136998	0.32055029	0.20989938
ENSG000001	TRIM38	0.0440744	0.35392484	0.20993821
ENSG000001	OR52K1	0.14087159	0.50051652	0.20996083
ENSG000002	GH1	0.64832574	0.8617982	0.20996812
ENSG00000C	KRIT1	0.05900556	0.38096018	0.20998864
ENSG000001	ZNF572	0.2741813	0.63454962	0.21030813
ENSG000001	APOL2	0.19805164	0.56540555	0.2103142
ENSG00000C	AGPS	0.01062403	0.24206404	0.2103346
ENSG000001	NFKBIE	0.02331901	0.29813446	0.21043871
ENSG000002	N/A	0.27019375	0.63142427	0.21045206
ENSG000002	ZC3H11B	0.06384127	0.38992693	0.2105384
ENSG000001	PLAAT3	0.0374725	0.33471496	0.21055433
ENSG000001	MLC1	0.03054789	0.3191802	0.21060476
ENSG000001	FCRL4	0.65664247	0.86503541	0.21063961
ENSG000001	FST	0.56030649	0.8171626	0.21066266
ENSG000001	ZNF451	0.06582413	0.3931782	0.2106878
ENSG000001	LNX2	0.03627493	0.33244265	0.21077207
ENSG000001	CTDSPL2	0.04536909	0.35545023	0.21077543
ENSG000001	KIF20B	0.13143045	0.4894044	0.21089344
ENSG000001	CEPT1	0.06340762	0.38953821	0.21090542
ENSG000002	ADGRG1	0.14098695	0.50051652	0.21100163
ENSG00000C	ZC3H15	0.09683487	0.44304329	0.21101841
ENSG000001	SMYD2	0.04749568	0.35865951	0.21103533
ENSG000001	GDPD1	0.07022837	0.40066693	0.21123514
ENSG000001	CPEB3	0.07949975	0.4152849	0.21127832

ENSG000001	PHF6	0.14183661	0.50055578	0.21135661
ENSG00000C	BLNK	0.04135868	0.34725125	0.21139246
ENSG000001	DENND5B	0.07342085	0.40497004	0.21147187
ENSG000001	KIF18A	0.22160643	0.5889073	0.21155031
ENSG000001	ARSB	0.01251312	0.2548969	0.21157651
ENSG000001	ZNF22	0.18510916	0.550214	0.21178618
ENSG000001	SPIRE1	0.05071819	0.36298244	0.21182969
ENSG000001	ELOVL6	0.11873318	0.47554586	0.21184669
ENSG000001	DENND4A	0.00136183	0.18856694	0.21187145
ENSG000002	N/A	0.62435357	0.84979805	0.21191552
ENSG000002	PNMA8B	0.50886796	0.79132674	0.21202274
ENSG000001	ZDHHC20	0.0502807	0.36227834	0.21207669
ENSG000001	ZNF121	0.10663339	0.45683544	0.21210349
ENSG000001	OPN1SW	0.26173464	0.62545731	0.21213286
ENSG00000C	PRR11	0.0608829	0.38464604	0.21215031
ENSG000001	CEP83	0.07583495	0.41015711	0.21216595
ENSG000001	GRK4	0.03530079	0.32934644	0.21220261
ENSG000002	N4BP2L2	0.03586634	0.33046641	0.21220997
ENSG000001	ESR2	0.00196338	0.19438665	0.21221185
ENSG000001	ZNF573	0.18485414	0.5498217	0.21226567
ENSG000001	WASL	0.06474377	0.39055819	0.21237321
ENSG000001	INSYN2A	0.61688971	0.84613614	0.21251506
ENSG00000C	TMEM38B	0.06378131	0.38991609	0.21253628
ENSG000001	CEP126	0.13416945	0.49298696	0.21260837
ENSG000001	DNAJB4	0.22103872	0.58845806	0.21279655
ENSG000002	N/A	0.12201375	0.47873532	0.21289662
ENSG000001	ADAM12	0.16381778	0.52709285	0.21290147
ENSG00000C	YTHDC2	0.04354344	0.35350745	0.21293901
ENSG000001	ZFP28	0.11822186	0.47496594	0.21305725
ENSG00000C	C1orf112	0.02082663	0.28641584	0.21307012
ENSG000001	SCD5	0.1471247	0.50547266	0.21328701
ENSG000001	SAT1	0.06095167	0.38464604	0.21332656
ENSG000001	KIF21A	0.09827786	0.4448142	0.21339391
ENSG000001	TCAF2	0.00547655	0.21833269	0.21340658
ENSG00000C	SRBD1	0.04380791	0.35372007	0.21343431
ENSG000001	NUTM2F	0.56003313	0.81699589	0.21344206
ENSG000001	RFX5	0.00241011	0.2093801	0.21346404
ENSG000001	MRPL44	0.02178523	0.29183808	0.21369542
ENSG000001	CRISP2	0.55572119	0.81461822	0.21371594
ENSG000001	CD48	0.07129365	0.40241675	0.21371964
ENSG000001	TBCEL	0.00641091	0.22290736	0.2138328
ENSG000001	ZNF594	0.38645183	0.71556829	0.21407433
ENSG000001	ATP8B4	0.06279053	0.38794893	0.21410142

ENSG000001	WWC1	0.34649123	0.68614821	0.21414684
ENSG000001	ADPRH	0.01827441	0.27758038	0.21416108
ENSG00000(	MAT2B	0.0015495	0.19292382	0.21418017
ENSG000001	EIF2S1	0.0327834	0.32362351	0.2144717
ENSG00000(	TGFBR3	0.08024521	0.41684053	0.21460205
ENSG00000(	MAP4K5	0.03397189	0.32562081	0.21461037
ENSG000001	TMOD2	0.01522594	0.26300382	0.21487732
ENSG000002	C6orf47	0.57012575	0.82066771	0.21489365
ENSG000001	SPRED3	0.19746288	0.56521618	0.21502037
ENSG00000(	FNIP2	0.007295	0.22727528	0.21503964
ENSG000001	ZNF793	0.18493257	0.54995424	0.21506894
ENSG000001	METTL14	0.03169938	0.32249556	0.21510887
ENSG000001	LYSMD3	0.09790779	0.44463533	0.21512842
ENSG00000(	BCAR1	0.70601359	0.88780148	0.21513161
ENSG000001	ZNF682	0.26919883	0.63099817	0.21524669
ENSG000001	HUS1	0.01157178	0.24690018	0.21527399
ENSG000001	FASTKD1	0.12135369	0.47767755	0.21530463
ENSG000001	KANSL1L	0.0251135	0.30075912	0.21537951
ENSG000001	SEC24D	0.05458001	0.37354464	0.21540667
ENSG000001	TPH1	0.32193783	0.66822528	0.21548709
ENSG000001	KIF24	0.27282293	0.63378205	0.21559942
ENSG000001	SERPINI1	0.23921462	0.60547587	0.21565627
ENSG000001	SMIM15	0.05680234	0.37568212	0.21576387
ENSG000001	DRAXIN	0.09548521	0.4413663	0.21607785
ENSG000002	H2BC17	0.41491335	0.73203826	0.21633437
ENSG000001	FUT4	0.01009625	0.24143268	0.21638483
ENSG000002	TRIM49D1	0.5222101	0.79796023	0.21646473
ENSG00000(	ACTR6	0.22071432	0.5880238	0.2164956
ENSG000001	CAAP1	0.05485509	0.37354464	0.21651143
ENSG000002	C12orf73	0.12466332	0.48205722	0.21656714
ENSG000001	LIN7C	0.03522786	0.32931219	0.2166111
ENSG000001	TMEM135	0.08914739	0.43342334	0.21667974
ENSG000001	ZNF620	0.0393749	0.34025028	0.21693771
ENSG000001	ICOS	0.14765251	0.50621491	0.21696462
ENSG000001	KBTBD3	0.3255369	0.67124189	0.2169687
ENSG000001	CSRNP1	0.16138176	0.52426078	0.21706435
ENSG000001	ZNF25	0.06670216	0.39496052	0.21713881
ENSG00000(	ZNF302	0.21186615	0.57666273	0.21721999
ENSG000001	GFAP	0.48443501	0.77671287	0.21734041
ENSG000001	RAB8B	0.02784216	0.31135087	0.21741514
ENSG000001	MIGA1	0.07223433	0.40373033	0.21749781
ENSG000001	ACADM	0.03302392	0.32362351	0.21759014
ENSG000001	B3GLCT	0.1048945	0.45514301	0.21760026

ENSG000001	PSEN2	0.02495736	0.30049829	0.21770477
ENSG000002	MRPL46	0.09440393	0.43981122	0.21776213
ENSG000001	SLC25A46	0.08331177	0.42260872	0.21777083
ENSG000001	SRSF10	0.07074788	0.40156223	0.21778284
ENSG000001	HELQ	0.043512	0.3534289	0.21788569
ENSG000002	PWP2	0.41635151	0.73242681	0.21794398
ENSG000001	UGDH	0.01436556	0.2599395	0.21794729
ENSG000001	UBQLNL	0.33339083	0.67581361	0.21798739
ENSG000001	RCBTB1	0.02156928	0.29113747	0.21806625
ENSG000001	XCL1	0.07687139	0.41125561	0.21808398
ENSG000001	PPARG	0.4649669	0.76413639	0.21810593
ENSG000001	DYNLT1	0.07663263	0.41092575	0.21814333
ENSG000001	CLDN12	0.42446519	0.73751111	0.21815234
ENSG000001	DOCK7	0.02231331	0.29312398	0.21820031
ENSG000001	DCUN1D5	0.04919839	0.360767	0.2182367
ENSG000002	EID3	0.22406576	0.59147387	0.21824154
ENSG000001	CDC73	0.00860095	0.23436355	0.2183582
ENSG000001	ISG20	0.16582324	0.52885285	0.21839875
ENSG000001	KLF10	0.02376597	0.29875884	0.21840355
ENSG000001	POU3F1	0.65097185	0.86340712	0.21858121
ENSG000001	ZDHHC13	0.01342714	0.25828987	0.21886896
ENSG000000	EXOC5	0.05340627	0.37010565	0.21888418
ENSG000001	CBWD5	0.06348378	0.38981851	0.21890208
ENSG000001	ABO	0.04929721	0.360767	0.2190468
ENSG000001	CCNC	0.11653662	0.47188147	0.21919687
ENSG000001	GAS1	0.41260619	0.73063968	0.21925037
ENSG000002	C9orf135	0.45197908	0.75395007	0.21944834
ENSG000001	CNTRL	0.00267853	0.2093801	0.21953827
ENSG000002	PRR3	0.26806774	0.63028246	0.21962735
ENSG000000	ZNF195	0.14338434	0.50145476	0.21976615
ENSG000001	LTV1	0.07439847	0.40673667	0.21981885
ENSG000001	PIF1	0.20511097	0.56900508	0.22009592
ENSG000001	USP6NL	0.01142192	0.24590889	0.2202464
ENSG000001	SORCS2	0.32245249	0.66841072	0.22025475
ENSG000001	IL17D	0.63973634	0.85691647	0.22029857
ENSG000001	IRF5	0.03050369	0.3191802	0.22048795
ENSG000001	ZNF678	0.1141394	0.46962187	0.22051521
ENSG000002	DOC2B	0.57250127	0.82197393	0.22061291
ENSG000002	KLRF2	0.56061415	0.81728196	0.22061712
ENSG000001	ADAP2	0.0331805	0.3238836	0.22066261
ENSG000000	DLX3	0.47548102	0.77011325	0.22094341
ENSG000002	ASB14	0.24613764	0.6120929	0.22096643
ENSG000001	TMEM144	0.05963853	0.38274736	0.22140534

ENSG00000C	TRIB2	0.08441019	0.42458743	0.22156899
ENSG000001	ZMYM1	0.09160712	0.43611772	0.22161189
ENSG000001	PIGA	0.10331006	0.45287405	0.22161245
ENSG000001	BMI1	0.10374761	0.45356756	0.22161599
ENSG000001	NCR3LG1	0.24535597	0.61141177	0.22166051
ENSG000002	FOXO3B	0.26566786	0.62853695	0.22168124
ENSG000002	EFCAB2	0.24894929	0.61478169	0.22172271
ENSG000001	ZNF613	0.05559299	0.37486021	0.22172368
ENSG00000C	ZNF510	0.10114116	0.44918737	0.22172789
ENSG000001	ARFGEF2	0.04369494	0.35372007	0.22173806
ENSG000001	CPNE8	0.07587928	0.41015711	0.22174518
ENSG000001	ACTA2	0.06501311	0.39140445	0.2218039
ENSG000001	ZNF556	0.57444233	0.82330481	0.22181557
ENSG000001	AASDHPPT	0.01873862	0.27889987	0.22187781
ENSG000001	FAM20A	0.12112539	0.47724625	0.22188506
ENSG000001	APPL1	0.02663707	0.30699826	0.22194313
ENSG000001	SETDB2	0.02809429	0.31180241	0.22198187
ENSG000001	RPS6KC1	0.00452697	0.21750928	0.22198962
ENSG000001	ASB2	0.59665366	0.83600051	0.22201224
ENSG000001	BTN3A3	0.00917287	0.23551879	0.22213559
ENSG00000C	FMO4	0.05424675	0.37256839	0.22218728
ENSG000001	EIF5A2	0.06221585	0.38624952	0.22221209
ENSG000001	SLC5A2	0.41667486	0.73285581	0.22221878
ENSG000001	CASP3	0.04592004	0.35708993	0.22230859
ENSG000002	TBC1D3K	0.68695541	0.88028723	0.2225522
ENSG000001	PAIP1	0.04750457	0.35865951	0.22258854
ENSG000001	SPAG17	0.59089463	0.83284123	0.22275682
ENSG00000C	IGSF9	0.52081692	0.79735075	0.222776
ENSG00000C	DNAH9	0.61793288	0.84662697	0.22281229
ENSG000001	TPRKB	0.07260629	0.40408609	0.22282933
ENSG000001	MINDY3	0.10092154	0.44917064	0.2228578
ENSG000001	GORAB	0.15855502	0.52129996	0.22288032
ENSG000001	NKD1	0.03850264	0.3372649	0.22291671
ENSG000001	PRKACB	0.18412485	0.54950998	0.22298218
ENSG000001	SNTG2	0.63187155	0.85383642	0.22302254
ENSG000001	CLIP4	0.01643281	0.27000801	0.22307757
ENSG000002	COL5A2	0.33175498	0.67477571	0.22316857
ENSG000001	MYO1E	0.01973219	0.28263225	0.22317955
ENSG000002	KLRK1	0.12856354	0.48678709	0.22319521
ENSG000001	FGD6	0.01638878	0.27000801	0.22320203
ENSG000001	TIGD5	0.34561014	0.68530904	0.22322094
ENSG000002	EFCAB7	0.18955292	0.55424774	0.22352204
ENSG000001	ZNF256	0.10564765	0.4561473	0.22364721

ENSG000001	RNASE2	0.06845225	0.39796501	0.22366612
ENSG000001	FCRL3	0.03484825	0.32855052	0.22368713
ENSG000001	ZNF677	0.14734592	0.5058046	0.22369555
ENSG000001	ZNF600	0.14672298	0.50526849	0.22379714
ENSG000001	GPN3	0.00829525	0.23282737	0.22384305
ENSG000001	UST	0.07227721	0.40373033	0.22390658
ENSG000002	ZNF525	0.23442166	0.6021681	0.22393087
ENSG000001	TRPV4	0.28770037	0.64495772	0.22400254
ENSG000001	ATR	0.00842333	0.23429041	0.22400795
ENSG000001	ZRANB3	0.03173275	0.32263285	0.2240507
ENSG000002	ZNF224	0.04062178	0.34551949	0.22412206
ENSG000001	SLC35F5	0.03206728	0.32311282	0.22415505
ENSG000002	SMIM13	0.08472717	0.42501928	0.22431581
ENSG000001	UBE2S	0.12428405	0.48162293	0.22431991
ENSG000001	TASOR2	0.01783544	0.27425066	0.22432855
ENSG000001	RAB28	0.02413693	0.29927225	0.22442262
ENSG000001	OXR1	0.08349929	0.42260872	0.22446489
ENSG000001	CRYGN	0.46675742	0.7650199	0.22446847
ENSG000001	CD226	0.07898419	0.4147264	0.22471544
ENSG000001	HORMAD1	0.05553181	0.37475895	0.2250206
ENSG000001	PRPS2	0.01199627	0.25192551	0.22506523
ENSG000001	EPX	0.35151173	0.69054758	0.22509399
ENSG000001	ERI1	0.00654187	0.22296216	0.225154
ENSG000001	UFM1	0.08942984	0.43345443	0.22520307
ENSG000001	KLHL28	0.12359156	0.48039028	0.22524635
ENSG000001	IL1RN	0.29063228	0.648324	0.22526491
ENSG000001	LACTB	0.01586979	0.26702367	0.2252963
ENSG000002	ITPRIPL2	0.07045013	0.40156223	0.2252963
ENSG000002	N/A	0.47938916	0.7733699	0.2253439
ENSG000001	PSMC6	0.15791658	0.52077869	0.22544585
ENSG000001	AKAP6	0.21390258	0.57962886	0.22559437
ENSG000001	C1orf100	0.40945164	0.72902686	0.22568949
ENSG000001	SIGLEC11	0.24732356	0.61337904	0.22579773
ENSG000002	N/A	0.05019273	0.36227834	0.22580715
ENSG000001	ZNF300	0.19858501	0.56598606	0.2259336
ENSG000001	HHIPL1	0.56417569	0.81878089	0.22595464
ENSG000001	EVI2A	0.25618834	0.62056088	0.22605187
ENSG000001	MS4A7	0.03885718	0.33829709	0.22605385
ENSG000001	GVQW3	0.24079714	0.60721888	0.22608012
ENSG000001	PHF1	0.42022771	0.73468557	0.22609751
ENSG000001	SLC6A12	0.17809629	0.54305154	0.22614692
ENSG000002	COL15A1	0.4291927	0.74081023	0.22619472
ENSG000001	GPBAR1	0.0475588	0.35866802	0.22627124

ENSG00000(	OTUB2	0.3086253	0.65826962	0.22644332
ENSG000001	LPAR1	0.06971245	0.40011351	0.22644797
ENSG000001	ZNF322	0.03105779	0.31953017	0.22646144
ENSG000001	SFTPD	0.25981214	0.62338681	0.22648872
ENSG000001	SLC1A2	0.6239647	0.84972866	0.22653539
ENSG00000(	MEF2A	0.0086346	0.23436355	0.22654869
ENSG000001	CD300E	0.01783815	0.27425066	0.22664601
ENSG000001	RALGPS2	0.04679084	0.35803149	0.22665016
ENSG000001	DMXL2	0.05323667	0.36981204	0.22668885
ENSG000001	ZNF365	0.20270523	0.56815551	0.22675095
ENSG00000(	ACSM3	0.02411582	0.29927225	0.22680289
ENSG000001	DCUN1D3	0.00757895	0.22933195	0.22681592
ENSG000001	AXL	0.30122352	0.65544978	0.2268575
ENSG00000(	IBTK	0.03053559	0.3191802	0.22686133
ENSG000002	PCDHGB4	0.65775898	0.86540022	0.22690754
ENSG000001	PYURF	0.03742596	0.33471496	0.22696054
ENSG000001	ZMAT4	0.49596947	0.78185081	0.22698282
ENSG000001	ENTPD7	0.01738331	0.27265457	0.22698501
ENSG000001	INTS6L	0.03993028	0.34274931	0.22712974
ENSG000001	SMARCA5	0.01303596	0.25656355	0.22721089
ENSG000001	FAM72A	0.39208466	0.71994497	0.22724645
ENSG000002	N/A	0.43177978	0.74133535	0.22731006
ENSG000002	ZNF888	0.03413827	0.32634691	0.22737367
ENSG000001	MX2	0.17560775	0.54061531	0.22754982
ENSG000001	C5orf15	0.00428399	0.21614597	0.22755454
ENSG000001	AKAP14	0.54341075	0.8084284	0.22761355
ENSG000001	ODF2L	0.13137163	0.4894044	0.22769386
ENSG000001	CNP	0.09301934	0.43758758	0.22772945
ENSG000001	LEMD3	0.03356409	0.32398208	0.22776531
ENSG000001	CSMD1	0.41764016	0.73321581	0.22776799
ENSG000001	PPP1R14C	0.51690208	0.79538014	0.22789305
ENSG00000(	LPCAT2	0.08034955	0.41684053	0.22789788
ENSG000001	SP140L	0.01608045	0.26872151	0.22790137
ENSG000002	N/A	0.43502002	0.74433605	0.22790252
ENSG00000(	ERO1B	0.03543079	0.32967891	0.22792682
ENSG000002	ZNF736	0.06809092	0.39743754	0.22794151
ENSG000001	ERFE	0.53730522	0.80541219	0.22796346
ENSG000001	ARHGEF15	0.64299975	0.85899548	0.2279806
ENSG000001	BNC2	0.25120628	0.61695463	0.22810981
ENSG000001	DLX4	0.03101996	0.31953017	0.22811267
ENSG000001	LGALS9	0.09476332	0.43988973	0.22840364
ENSG000001	ZNF154	0.09753496	0.44422866	0.22842504
ENSG000001	DCAF13	0.06961187	0.40004644	0.22843826

ENSG000001	ZNF772	0.15013721	0.50988869	0.22848213
ENSG000001	ST6GALNAC5	0.5495221	0.81217374	0.22848389
ENSG000001	PAXBP1	0.09653093	0.44251066	0.22877799
ENSG000001	DMXL1	0.0318463	0.3227302	0.22877803
ENSG000002	FPGT	0.11771847	0.47440925	0.22878085
ENSG000000	SCAMP1	0.03878966	0.33829709	0.22880925
ENSG000001	GRIK3	0.64583835	0.86025245	0.22890753
ENSG000001	CIITA	0.00256255	0.2093801	0.22907655
ENSG000001	TEX30	0.10471365	0.45514301	0.2291178
ENSG000000	PTPRU	0.44065282	0.74748014	0.22914536
ENSG000001	TXNDC9	0.12504284	0.48283488	0.22925782
ENSG000001	ZNF70	0.04903966	0.360767	0.22928287
ENSG000001	VWC2L	0.63912128	0.85691647	0.22929977
ENSG000001	DCK	0.02850952	0.31345545	0.22934353
ENSG000001	EFCAB13	0.14045189	0.50035294	0.22935131
ENSG000001	CCDC181	0.25646847	0.62074989	0.22938438
ENSG000001	TSGA10IP	0.43112388	0.74126857	0.22938921
ENSG000001	MRPL1	0.17907913	0.54420884	0.22945623
ENSG000000	IGFALS	0.45325408	0.75474172	0.2295288
ENSG000002	LCNL1	0.62295467	0.84927918	0.2295965
ENSG000000	DZANK1	0.09044659	0.43397794	0.22973412
ENSG000001	GABPA	0.06443882	0.39055819	0.22993128
ENSG000000	RPAP3	0.01704674	0.27224356	0.22995512
ENSG000001	DRAP1	0.07225488	0.40373033	0.23005289
ENSG000002	DBNDD2	0.24267596	0.60820533	0.23013543
ENSG000000	SPDL1	0.13443056	0.49361126	0.23014376
ENSG000001	SOCS4	0.08412275	0.42379805	0.23027633
ENSG000002	C8orf88	0.37932312	0.70965485	0.2304751
ENSG000001	SSX2IP	0.04991137	0.36170272	0.23058547
ENSG000001	KIF17	0.51983317	0.7967342	0.23083811
ENSG000001	BPNT2	0.00477647	0.21772133	0.23086199
ENSG000000	ZFYVE26	0.05470002	0.37354464	0.23089682
ENSG000001	C10orf88	0.05407417	0.37235045	0.2309338
ENSG000001	NOVA2	0.48990863	0.77995113	0.23093569
ENSG000001	MOB3C	0.01295007	0.25583328	0.23102395
ENSG000001	TCL1A	0.01735865	0.27265457	0.23103578
ENSG000001	LMO2	0.06534397	0.39169239	0.23108706
ENSG000000	KCNQ2	0.57969157	0.82610777	0.2311728
ENSG000002	TUNAR	0.53907528	0.80656378	0.23117997
ENSG000001	MATN1	0.26003565	0.62338681	0.23134555
ENSG000001	COA6	0.08807501	0.43074518	0.23138675
ENSG000001	LTN1	0.1083336	0.45963571	0.23141648
ENSG000001	ATP6V1G1	0.03585983	0.33046641	0.23153104

ENSG000001	MITD1	0.0033725	0.2114129	0.2315881
ENSG000001	SP110	0.06294752	0.38841248	0.23175347
ENSG000001	P2RY1	0.14333454	0.50145476	0.23187348
ENSG000001	CARNMT1	0.08263321	0.42205583	0.23191735
ENSG000001	N4BP2L1	0.00551667	0.21833269	0.23212928
ENSG000001	SCIMP	0.02268856	0.29463194	0.23213217
ENSG000001	BIRC2	0.0520368	0.36656028	0.23213249
ENSG000001	PHOSPHO2	0.40874408	0.72855546	0.23213726
ENSG000001	RPS27L	0.07705588	0.41179232	0.23214507
ENSG000001	HRH4	0.05726001	0.37686693	0.23219918
ENSG000001	MSANTD4	0.04644473	0.35774922	0.23220876
ENSG000001	GSG1L	0.62358593	0.84956912	0.23222658
ENSG000001	BTN2A2	0.00234065	0.2093801	0.23222721
ENSG000001	GRM2	0.19727974	0.56500135	0.23235328
ENSG000001	BLOC1S6	0.01885387	0.27889987	0.23247975
ENSG000001	NMI	0.0452496	0.35545023	0.23264328
ENSG000001	SLC15A2	0.00247667	0.2093801	0.23271655
ENSG000002	SRGAP2	0.12616656	0.48388732	0.23280121
ENSG000001	BTLA	0.15148798	0.51259637	0.23287405
ENSG000001	TMEM126B	0.05946048	0.38178501	0.23289654
ENSG000001	COG6	0.05491039	0.37354464	0.2329783
ENSG000001	PAWR	0.08422219	0.42403588	0.23308389
ENSG000001	NLRC5	0.03207845	0.32311282	0.23313841
ENSG000001	ADRB2	0.01504274	0.26260224	0.23315106
ENSG000001	REL	0.0072525	0.22727528	0.23315851
ENSG000001	ARGLU1	0.18198073	0.54661139	0.23324355
ENSG000002	NRAS	0.00657309	0.22296216	0.23330604
ENSG000001	MBNL1	0.05032196	0.36227834	0.23332541
ENSG00000(	OSBPL5	0.09523627	0.44084511	0.23339097
ENSG00000(	MBTPS2	0.04397865	0.35385593	0.23342708
ENSG000001	ZNF267	0.13186936	0.49019294	0.23345588
ENSG000002	GOLGA8B	0.17077624	0.53457173	0.23348014
ENSG000001	WDR5B	0.06862345	0.39796501	0.23354763
ENSG000001	SUB1	0.15209343	0.51296214	0.2338054
ENSG000001	PKD2	0.05623793	0.37502119	0.23385246
ENSG000001	RBM45	0.03150836	0.32149851	0.2340119
ENSG000001	MINAR1	0.09543624	0.4413663	0.23404387
ENSG000001	CHST12	0.050683	0.36298244	0.2340457
ENSG000001	WWC2	0.0692103	0.39920699	0.23407906
ENSG00000(	UFL1	0.09268757	0.43753271	0.23423319
ENSG000001	PACRGL	0.09931452	0.44669524	0.23424146
ENSG000001	MANEAL	0.12518719	0.48304764	0.23428892
ENSG000001	LGALS8	0.00385865	0.21383246	0.23431215

ENSG000001	NDUFA9	0.01426051	0.25987426	0.23443705
ENSG000001	COPS2	0.17656694	0.5414386	0.23445299
ENSG000001	ZNF761	0.09066711	0.43454471	0.23465143
ENSG000001	SPIN4	0.1441316	0.50241838	0.23466943
ENSG000001	HAPLN3	0.02002247	0.28415764	0.23467071
ENSG000001	CCR1	0.06235743	0.38624952	0.23474078
ENSG000002	KIFC1	0.5522682	0.81364474	0.23482156
ENSG000001	VPS13A	0.09757626	0.44429215	0.235001
ENSG000002	ZNF879	0.04540833	0.35545023	0.23512486
ENSG000001	ANKRD49	0.09476715	0.43988973	0.23521204
ENSG000001	GLRX	0.00829179	0.23282737	0.23528141
ENSG000001	FARP1	0.05760096	0.37712373	0.23531576
ENSG000001	SMAD5	0.06146168	0.38542193	0.23544645
ENSG000001	ATF2	0.0341104	0.32634691	0.23572958
ENSG000001	AIRE	0.06621043	0.39350613	0.23581003
ENSG000001	SMC6	0.0746495	0.40742317	0.23581779
ENSG000002	L1TD1	0.25931304	0.62303431	0.23600186
ENSG000001	ROBO1	0.27212525	0.63329478	0.23610428
ENSG00000(	HSP90AA1	0.10033059	0.4480999	0.23611766
ENSG000001	RYR3	0.11507563	0.47079584	0.23612568
ENSG000001	SRFBP1	0.05871978	0.38067583	0.23615722
ENSG000001	RO60	0.01042046	0.24174018	0.2362567
ENSG000001	RBAK	0.10875043	0.46040635	0.23652443
ENSG000001	DYNLT3	0.11961202	0.47604098	0.23673218
ENSG000001	NASP	0.00123592	0.18856694	0.23677653
ENSG000001	CREB3L2	0.00237386	0.2093801	0.23679678
ENSG000001	COBL	0.57602754	0.82368641	0.23691249
ENSG000001	GIPC2	0.65112867	0.8634351	0.23695071
ENSG000001	TAF1A	0.07412601	0.40606749	0.23696521
ENSG000001	PRXL2C	0.00088192	0.18856694	0.23699798
ENSG000001	CENPJ	0.03574564	0.33014896	0.23701254
ENSG000001	FGD2	0.0146581	0.26167713	0.237069
ENSG00000(	MSMO1	0.02162714	0.29166107	0.23708534
ENSG000001	PLA1A	0.61539316	0.84537475	0.23733436
ENSG000001	LMBRD2	0.05720509	0.37676557	0.23744389
ENSG000001	APOA2	0.18080354	0.54567047	0.23749731
ENSG000001	ACSL3	0.00321501	0.21134435	0.23765789
ENSG000001	PLAGL1	0.02304201	0.29669552	0.23776864
ENSG000001	PUS7L	0.12455952	0.48188538	0.23785634
ENSG000002	ARL17B	0.28540867	0.64333814	0.23792569
ENSG000001	COL10A1	0.5766226	0.8241744	0.23801206
ENSG000001	TMEM72	0.62041019	0.84787478	0.23809099
ENSG000001	SLC30A7	0.00400097	0.21403539	0.23810507

ENSG00000C	CASP10	0.01066975	0.24206404	0.2384402
ENSG000001	HMGCS1	0.01926493	0.28088168	0.23859064
ENSG000002	LIMS4	0.51350769	0.79316402	0.23884232
ENSG000001	TOMM20L	0.5069452	0.78972169	0.2388472
ENSG000001	MORC3	0.01312898	0.25731723	0.23893777
ENSG000001	ZNF334	0.53997192	0.80698777	0.23897646
ENSG00000C	FARP2	0.00124991	0.18856694	0.23899436
ENSG000001	ARHGAP17	0.00215873	0.20144452	0.23913281
ENSG000001	WSB1	0.0040584	0.21403539	0.23919095
ENSG000001	SYTL5	0.50922247	0.79136944	0.23924081
ENSG000001	RTN4RL2	0.42613826	0.73856395	0.23934661
ENSG000001	SUCNR1	0.25358439	0.61921212	0.23950505
ENSG000001	LSM8	0.04844562	0.360767	0.23956247
ENSG00000C	ZNF280C	0.01360765	0.25910565	0.23956679
ENSG000001	MTX2	0.0484546	0.360767	0.23958671
ENSG000001	DIPK2A	0.01542989	0.26455656	0.23959962
ENSG000002	UGT2B11	0.23820315	0.60432883	0.23967625
ENSG000001	MAP9	0.15029035	0.5100887	0.23985018
ENSG000001	ADGRG6	0.21737226	0.58396318	0.23987557
ENSG000001	CLIC2	0.08727273	0.42940825	0.2400751
ENSG000002	GPR142	0.57993156	0.82610777	0.24015573
ENSG00000C	C12orf4	0.04291092	0.35185871	0.24029221
ENSG000001	THRB	0.15398654	0.51533385	0.24063581
ENSG000001	H1-5	0.61511886	0.84533626	0.24086034
ENSG000001	FNDC3A	0.01958144	0.28172722	0.24094829
ENSG000001	PAK1IP1	0.04498199	0.35545023	0.24102038
ENSG000001	RHOU	0.02413863	0.29927225	0.24105884
ENSG000001	IQUB	0.56833017	0.82037292	0.24107054
ENSG000001	CASKIN2	0.16415392	0.52758655	0.24129324
ENSG00000C	ATP1B3	0.00450928	0.21750928	0.24130906
ENSG000001	ZNF518A	0.07951827	0.4152849	0.24136321
ENSG000001	HIF3A	0.5375657	0.80547376	0.24142265
ENSG000002	DNAJC19	0.0618821	0.38600832	0.24157743
ENSG00000C	PIBF1	0.05386257	0.37208157	0.24161796
ENSG000001	JUP	0.12989933	0.48787839	0.24162219
ENSG000001	ST8SIA1	0.09825646	0.4448142	0.24194326
ENSG000001	SQLE	0.0321893	0.32322672	0.24227239
ENSG000001	IFTAP	0.5149104	0.79420539	0.24229771
ENSG000001	CREBZF	0.11556445	0.4710215	0.24240026
ENSG000001	ZC3H12C	0.02118341	0.28959078	0.24248362
ENSG000001	CLEC2B	0.04950267	0.36128416	0.24250276
ENSG000001	MFAP4	0.35038422	0.6900168	0.24250358
ENSG000001	DNAJC1	0.04834929	0.360767	0.24252042

ENSG000001	ABCA1	0.03218318	0.32322672	0.24259944
ENSG000001	RDX	0.00529456	0.21833269	0.24261062
ENSG000001	TUBA3E	0.57512439	0.82344878	0.24275714
ENSG000001	CX3CR1	0.00400276	0.21403539	0.24277079
ENSG000001	MS4A1	0.06364548	0.38981851	0.24318971
ENSG000001	SESTD1	0.01810781	0.27631728	0.24324794
ENSG000001	LRRC43	0.40117308	0.72606468	0.24331445
ENSG000001	BBX	0.00011015	0.18011717	0.24333648
ENSG000001	KNCN	0.49501839	0.78124152	0.24342111
ENSG000001	PP2D1	0.17096515	0.53486727	0.24348591
ENSG000001	KIAA0895L	0.1408619	0.50051652	0.24380141
ENSG000001	CHODL	0.45526918	0.75608118	0.24380201
ENSG000001	ITGAV	0.1061703	0.4561473	0.24381573
ENSG000001	TMEM52	0.40624818	0.72748349	0.24387829
ENSG000001	SHLD2	0.1004399	0.44829276	0.2440488
ENSG000001	ADAM23	0.14555238	0.50423171	0.24447189
ENSG000002	SLC10A5	0.67214889	0.87316437	0.24450763
ENSG000001	TCTE1	0.57008793	0.82066771	0.24452394
ENSG000001	TIGIT	0.03037313	0.3191802	0.24457665
ENSG000001	DRD2	0.57068828	0.82112905	0.24459256
ENSG00000C	BTAF1	0.07148962	0.40241675	0.2446574
ENSG000001	SPRED1	0.19600129	0.56366859	0.24490647
ENSG000001	FER	0.02442143	0.29927225	0.24511778
ENSG000001	CD47	0.01463911	0.26167713	0.24522504
ENSG000001	B3GAT2	0.1891383	0.55383924	0.24530651
ENSG000001	MAIP1	0.01474931	0.26259267	0.2453825
ENSG000002	ZNF718	0.04682539	0.35811287	0.24538606
ENSG000001	FAM98A	0.00107217	0.18856694	0.24563614
ENSG000002	MARS2	0.02388201	0.29875884	0.24574143
ENSG000001	SP4	0.01381237	0.25910565	0.2458255
ENSG000001	DMD	0.37539712	0.70736023	0.24586143
ENSG000001	ASIC1	0.24479607	0.61084275	0.24609114
ENSG000001	TMEM130	0.65645653	0.86502879	0.24628501
ENSG000001	EML5	0.39482652	0.72193673	0.24632873
ENSG000001	PRKG1	0.35580805	0.69207883	0.2463637
ENSG000001	ATF5	0.1081815	0.45961253	0.2464073
ENSG000001	COX7A1	0.29903406	0.65423918	0.24643201
ENSG000002	SMIM34A	0.39914845	0.72496091	0.24668364
ENSG000001	CCDC91	0.02696423	0.30875752	0.24678172
ENSG000001	FAIM	0.10249984	0.45188429	0.24692094
ENSG00000C	RUFY3	0.04037814	0.34451913	0.24693516
ENSG000001	FSD1	0.08207563	0.42066352	0.24713917
ENSG000001	RUBCN	0.05243779	0.36842594	0.2471552

ENSG000001	HOXB8	0.53613207	0.80424764	0.24721462
ENSG00000(	MAP4K3	0.09412064	0.43939485	0.24745055
ENSG000001	RPL22L1	0.33821849	0.67998455	0.2474913
ENSG000002	RTL5	0.04717015	0.35851546	0.24770537
ENSG000001	UMODL1	0.39155123	0.71964297	0.24776989
ENSG000001	ZNF107	0.15452148	0.51592954	0.24787141
ENSG00000(	TRPM5	0.46801577	0.76574496	0.2479715
ENSG000001	TMEM64	0.01727257	0.27265457	0.24805765
ENSG000001	ERVK3-1	0.06155821	0.38542193	0.24806321
ENSG000001	SPATA13	0.01074955	0.24206404	0.24809974
ENSG000001	CCDC146	0.01996185	0.28393023	0.24813616
ENSG000001	NEK1	0.05007295	0.36227834	0.24825473
ENSG00000(	MYNN	0.06897279	0.39854492	0.24827753
ENSG000001	SLC2A13	0.04987905	0.36170272	0.24828906
ENSG000001	BDKRB2	0.35233185	0.69082603	0.24832943
ENSG000001	RAB23	0.27239305	0.63353982	0.24850468
ENSG000001	GNPDA2	0.07255513	0.40400469	0.24860592
ENSG000001	NPTX1	0.3509958	0.69046751	0.24863012
ENSG000002	N/A	0.55259469	0.81383402	0.24863687
ENSG000001	TRH	0.55358845	0.8139043	0.24870797
ENSG000001	ZNF496	0.05999248	0.38277429	0.24887535
ENSG000001	PLGRKT	0.02202802	0.29231997	0.24893436
ENSG000001	C3AR1	0.0137026	0.25910565	0.24905908
ENSG000002	N/A	0.27516535	0.63499184	0.24920757
ENSG00000(	PLEKHH1	0.2341178	0.60173104	0.24941306
ENSG000001	FAM8A1	0.05788514	0.37832043	0.24943914
ENSG000001	C5orf24	0.05940407	0.38178501	0.24944322
ENSG000001	SLFN11	0.00433312	0.21715097	0.24945793
ENSG000001	C19orf12	0.00014727	0.18011717	0.24955475
ENSG000001	MTBP	0.08175808	0.41959714	0.24958681
ENSG000001	CYP4F22	0.0693599	0.39974794	0.24961893
ENSG000001	RAB33B	0.03319401	0.3238836	0.2496399
ENSG00000(	TIE1	0.16117384	0.52412971	0.24976693
ENSG000001	ZNF711	0.30347604	0.6561378	0.2498306
ENSG000002	NLRP2B	0.47367554	0.76895276	0.24986772
ENSG000001	ASCC3	0.0022656	0.20901412	0.24990989
ENSG000001	GPAM	0.11415887	0.46962187	0.24996376
ENSG000001	PTGR1	0.32886106	0.67284741	0.24998608
ENSG000001	SLC16A7	0.13484605	0.49418805	0.24999081
ENSG000002	N/A	0.63063875	0.85323568	0.25002024
ENSG000001	FCRL5	0.04313986	0.35247453	0.25006628
ENSG000001	SLC1A7	0.35582152	0.69207883	0.25009248
ENSG000001	ZNF92	0.15325017	0.51454156	0.25017747

ENSG000001	OBI1	0.06678693	0.39505261	0.25028124
ENSG000001	DMC1	0.59942199	0.83721204	0.25038049
ENSG000001	SHFL	0.08536327	0.42555829	0.25039425
ENSG000001	TWF1	0.06168436	0.3855015	0.25061321
ENSG000001	TMEM181	0.02980127	0.31849971	0.25063458
ENSG000001	GEM	0.54188281	0.80808176	0.25071084
ENSG000001	TRIM69	0.04850433	0.360767	0.25075296
ENSG000001	LPAR4	0.43506648	0.74433605	0.25078052
ENSG000001	XKR6	0.07990133	0.41581982	0.25078773
ENSG000001	HSPA13	0.08648867	0.42833672	0.25120008
ENSG000001	PLA2G2F	0.51734343	0.79538919	0.25138676
ENSG000001	PANK3	0.05088815	0.36351554	0.25145295
ENSG00000C	DNAJA1	0.0159464	0.26748117	0.25152317
ENSG00000C	SELE	0.60442249	0.8388758	0.25164896
ENSG000001	SNX4	0.12685797	0.48465716	0.25169381
ENSG000001	CIBAR1	0.07779582	0.41335649	0.25171834
ENSG000002	STIMATE	0.02195675	0.29231997	0.2517367
ENSG000002	CHIC1	0.08259958	0.4220168	0.25175908
ENSG000001	ATP6V1B1	0.50837772	0.7908152	0.25176564
ENSG000001	OXGR1	0.67726578	0.87510133	0.2517831
ENSG000002	DDX52	0.11695358	0.4728891	0.25178543
ENSG000002	MZT1	0.05996713	0.38277429	0.25194266
ENSG000001	BANK1	0.00855098	0.23436355	0.25219384
ENSG000001	ACRV1	0.46937149	0.7662622	0.25221422
ENSG000001	RB1	0.00548332	0.21833269	0.25230432
ENSG000002	LGR4	0.14335269	0.50145476	0.25232452
ENSG000001	TIA1	0.03380639	0.32499367	0.25259495
ENSG000001	H4-16	0.24630945	0.61214306	0.25260615
ENSG000001	ARL13B	0.02317634	0.29724668	0.25270876
ENSG000002	COX20	0.06397518	0.39023108	0.25287664
ENSG000001	DNAH8	0.39162193	0.71964297	0.25292945
ENSG000001	EFCC1	0.56514387	0.81916096	0.25295055
ENSG000001	WARS2	0.02579125	0.30449397	0.25295937
ENSG000001	ZNF181	0.20178503	0.56773237	0.25298339
ENSG00000C	UBA6	0.00796973	0.23002982	0.25326061
ENSG000001	PDGFD	0.05927915	0.38150633	0.25338042
ENSG000001	CLIC5	0.01452607	0.26119577	0.25345472
ENSG000002	LORICRIN	0.51450611	0.79418535	0.2535331
ENSG000001	GLYATL1	0.58512786	0.82919554	0.25384393
ENSG000001	CEMIP	0.05768888	0.37739501	0.2539911
ENSG00000C	POLQ	0.23952837	0.60617553	0.25403363
ENSG000001	IL27	0.22912916	0.59761256	0.25404477
ENSG00000C	CENPQ	0.18282965	0.54726006	0.25421354

ENSG000001	SLC39A10	0.09001088	0.43377006	0.25447103
ENSG000001	TTYH1	0.53556048	0.80413676	0.25449342
ENSG000001	TRUB1	0.08321271	0.42260872	0.25451997
ENSG000001	DEPP1	0.15239993	0.5133091	0.25475711
ENSG000001	ZC3HAV1	0.02529972	0.30160909	0.25485849
ENSG00000C	ANGPT2	0.23331797	0.6008539	0.25490317
ENSG000001	TRAPPC13	0.04073983	0.34551949	0.25502947
ENSG000001	CASP8AP2	0.08889189	0.43309175	0.25504783
ENSG000001	RORB	0.55395701	0.81399095	0.25506321
ENSG00000C	FAM76B	0.08332971	0.42260872	0.25515726
ENSG000001	SHPRH	0.03878604	0.33829709	0.25518677
ENSG000001	PKD2L1	0.59320212	0.83450206	0.25535777
ENSG000001	WDR36	0.01240253	0.25464329	0.25557253
ENSG000002	ARPC4-TTLL3	0.29857817	0.65406944	0.25582483
ENSG000001	C10orf67	0.4438125	0.74908353	0.25585574
ENSG000001	ZNF644	0.11051802	0.46324664	0.25599068
ENSG000001	GPR182	0.58413293	0.82875175	0.25608575
ENSG00000C	PARP3	0.01898785	0.28000836	0.25617105
ENSG000001	CEP162	0.02137116	0.29086638	0.25619876
ENSG000001	BRMS1L	0.1168897	0.47282963	0.25638956
ENSG000001	WASHC4	0.07604419	0.41035311	0.25643304
ENSG000001	GOLT1B	0.05032393	0.36227834	0.25645688
ENSG000001	TIMP4	0.2738106	0.63403633	0.25646225
ENSG000001	FAM3C	0.03336972	0.32398208	0.25652945
ENSG000001	NETO2	0.00894638	0.23513527	0.25676045
ENSG000001	ACOT9	0.019573	0.28172722	0.25677454
ENSG000001	SCG3	0.56141749	0.81799496	0.25685835
ENSG000001	COL25A1	0.54809192	0.81141647	0.25693759
ENSG000001	PRTG	0.51278523	0.79243303	0.25705323
ENSG000001	H3-4	0.69763435	0.883235	0.25709382
ENSG000002	PLCXD2	0.08070016	0.41756804	0.2572107
ENSG000001	SLC25A36	0.07563537	0.41015711	0.25726521
ENSG000001	SEPSECS	0.04983614	0.36170272	0.25768715
ENSG000001	DSC1	0.44045072	0.74729345	0.25769439
ENSG00000C	CCNT2	0.07298314	0.40435477	0.25779283
ENSG000001	HLA-DQA1	0.01517684	0.26260224	0.25787136
ENSG000001	GPRC5C	0.30054837	0.65493729	0.25790417
ENSG000001	DENND2A	0.61913249	0.84711589	0.25811735
ENSG000001	MEIS2	0.35844134	0.69382497	0.25817251
ENSG000001	IMPA1	0.0301485	0.31859418	0.25839806
ENSG000001	PLA2G4C	0.08110834	0.41848493	0.25844732
ENSG000001	CBWD3	0.035232	0.32931219	0.25861558
ENSG000002	RPL17-C18oı	0.56790738	0.82037292	0.25866175

ENSG000002	C1QTNF9B	0.40101377	0.72606468	0.25869185
ENSG000001	ZNF618	0.0076411	0.22933195	0.25874233
ENSG000001	MCOLN2	0.02573691	0.30449397	0.25884408
ENSG000001	PHC3	0.05530521	0.37385126	0.25893986
ENSG000002	TRPC5OS	0.5355513	0.80413676	0.25899094
ENSG000001	RIPK2	0.01369518	0.25910565	0.25902617
ENSG000001	OTUD4	0.00865584	0.23436355	0.25904304
ENSG000001	DNAJC15	0.06033998	0.38403111	0.25921312
ENSG000001	SLC25A30	0.0050738	0.21833269	0.25928482
ENSG000001	CD276	0.60430026	0.83883394	0.25933161
ENSG000001	ZNF774	0.12771468	0.48588488	0.25939876
ENSG000002	HLA-A	0.13569796	0.49535247	0.25945103
ENSG000001	MCF2	0.34194931	0.68310736	0.25962657
ENSG000001	UBR1	0.00352274	0.21208655	0.2596316
ENSG000001	PYROXD1	0.0469621	0.35811287	0.26015759
ENSG000001	TERB2	0.2980268	0.65348564	0.26026306
ENSG000001	SCN3B	0.60021153	0.83754165	0.2602731
ENSG000001	CBR1	0.00814512	0.23121033	0.2602776
ENSG000002	CNTF	0.41238285	0.73063968	0.26028991
ENSG000001	TTC39B	0.05507261	0.37354464	0.26077247
ENSG000002	PPP1R10	0.55471351	0.81429195	0.26083896
ENSG00000C	PUM3	0.04898016	0.360767	0.26111388
ENSG000001	CCDC158	0.52599113	0.80004441	0.26126564
ENSG000001	DDIAS	0.00845564	0.23429041	0.26127568
ENSG000001	CDK15	0.28790153	0.64495821	0.26135263
ENSG000001	NAP1L5	0.05267595	0.36895324	0.2614176
ENSG000001	TP53AIP1	0.49794489	0.78278591	0.26146823
ENSG000001	MIA3	0.00515858	0.21833269	0.26149836
ENSG000001	HSPA2	0.36326465	0.69694698	0.2616061
ENSG000001	ZAN	0.45697588	0.75721169	0.2616066
ENSG000001	MED21	0.03809918	0.33602196	0.26186115
ENSG000001	RANBP6	0.09908059	0.4460792	0.26222262
ENSG000002	OR2A12	0.51893977	0.79603449	0.2622585
ENSG000001	FGFBP2	0.17215065	0.53630279	0.26228648
ENSG000001	GASK1B	0.03071724	0.31953017	0.26235032
ENSG00000C	ZNF112	0.4218083	0.73588712	0.2624197
ENSG000001	ZNF699	0.02869435	0.31345545	0.26243526
ENSG00000C	MARCO	0.05892146	0.38096018	0.26244512
ENSG000001	ZBTB33	0.01916688	0.28080056	0.26253164
ENSG000001	SLC35A3	0.07671513	0.41096123	0.26259524
ENSG000002	N/A	0.44506478	0.74940749	0.26265594
ENSG000001	MST1R	0.25430583	0.619382	0.26269796
ENSG000001	ZNF81	0.03344224	0.32398208	0.2627036

ENSG000002	FAM221B	0.50736382	0.79022218	0.26277408
ENSG00000C	P2RX7	0.01295611	0.25583328	0.26305366
ENSG00000C	GTF2IRD1	0.35829198	0.69382497	0.2632215
ENSG000001	FLVCR2	0.01175893	0.24956182	0.26325276
ENSG000001	MIER3	0.13278262	0.49139472	0.26333722
ENSG000001	AP1AR	0.06768509	0.39689346	0.2636497
ENSG000001	ZC3HAV1L	0.05226288	0.36780562	0.26377964
ENSG000001	IRAK2	0.00265761	0.2093801	0.2638467
ENSG000001	PRR4	0.34839781	0.68768967	0.26405215
ENSG00000C	COPZ2	0.06385513	0.38992693	0.26419395
ENSG00000C	TTC38	0.02227095	0.29312398	0.26425228
ENSG00000C	RECQL	0.02888727	0.31345545	0.26425838
ENSG00000C	EIF4G3	0.04444502	0.35479539	0.2642687
ENSG000001	CARMIL3	0.31549738	0.66324235	0.26450735
ENSG000001	TRAFD1	0.1312911	0.4894044	0.2645281
ENSG000001	GPR82	0.03696198	0.33341755	0.26461884
ENSG000001	CYP27B1	0.49383501	0.7811397	0.26462027
ENSG000001	ARHGEF3	0.00360436	0.21208655	0.26465565
ENSG000001	SRGAP2B	0.09285815	0.43753271	0.26468304
ENSG000001	CCN6	0.5250181	0.79924235	0.26475877
ENSG00000C	SPAG4	0.29055051	0.648324	0.26485214
ENSG00000C	ZNF184	0.04383933	0.35372007	0.26495692
ENSG00000C	GUCA1A	0.53549012	0.80413676	0.26496616
ENSG000001	C4orf33	0.01261936	0.25512421	0.26509842
ENSG000001	FAM189B	0.52795976	0.80062313	0.26529354
ENSG000001	THUMPD2	0.04646976	0.35774922	0.26531488
ENSG000001	AKAP3	0.26717348	0.62930502	0.26556206
ENSG000001	AP1S2	0.05603998	0.37502119	0.26572552
ENSG000001	ZSCAN1	0.48170594	0.77470873	0.26577502
ENSG000001	GNGT2	0.0041977	0.21614597	0.26583514
ENSG000002	ARHGEF28	0.21004904	0.57424655	0.26669237
ENSG000001	ZFP30	0.12057781	0.47724625	0.26669562
ENSG000001	RADX	0.07356293	0.40498137	0.26688987
ENSG000001	CUL5	0.02573269	0.30449397	0.26689979
ENSG000001	TCF19	0.45639902	0.75682857	0.26701447
ENSG000001	CHORDC1	0.05164077	0.36535566	0.26708964
ENSG000001	DTWD1	0.01418642	0.25910565	0.26721624
ENSG000001	TBC1D32	0.11832118	0.47496594	0.26732039
ENSG000001	ADGRB2	0.29826958	0.65366489	0.2673431
ENSG000001	HAUS1	0.0669093	0.39524277	0.26746827
ENSG000001	PCDH17	0.47415902	0.76912287	0.26759675
ENSG000001	ANGPTL4	0.22369668	0.59091342	0.26762885
ENSG000001	GABRR3	0.56034294	0.8171626	0.26775128

ENSG00000(	SORBS1	0.04994714	0.36170272	0.26782972
ENSG00000(	SUCO	0.0296205	0.31766715	0.26787147
ENSG000001	BBS7	0.03360262	0.32398208	0.26787612
ENSG000001	RHBDF2	0.11261864	0.46731123	0.26790473
ENSG000001	F3	0.6125795	0.84463768	0.26791376
ENSG000001	TMEM182	0.1262746	0.48388732	0.26798066
ENSG000001	SMO	0.43703756	0.74497757	0.26800156
ENSG000001	MS4A4A	0.07261952	0.40408609	0.26800641
ENSG000001	FAM166A	0.55005072	0.81222022	0.26809621
ENSG000002	THSD8	0.5628696	0.81833783	0.26814225
ENSG000001	ELK4	0.01652479	0.27013841	0.26815194
ENSG000001	ZNF816	0.05967995	0.38277429	0.26816919
ENSG000001	TAP1	0.35168028	0.69054758	0.26821834
ENSG000001	BTG3	0.09083939	0.4347065	0.2682237
ENSG000002	CXorf65	0.46804175	0.76574496	0.26832679
ENSG000001	IFI27L1	0.68061864	0.87652323	0.26843813
ENSG00000(	GOPC	0.04079083	0.34551949	0.26848859
ENSG000001	LEPR	0.05134639	0.36502002	0.26869896
ENSG000002	N/A	0.53251104	0.80280163	0.26871431
ENSG000001	FZD5	0.0501937	0.36227834	0.26874399
ENSG000001	USP37	0.03235202	0.32362351	0.26882017
ENSG000001	FUBP1	0.00127561	0.18856694	0.2688587
ENSG000001	CHST4	0.57832541	0.82533908	0.26889798
ENSG000001	CNDP2	0.02389339	0.29875884	0.26900995
ENSG000001	MAB21L3	0.36067884	0.69535997	0.26905319
ENSG000001	TDRD6	0.03296287	0.32362351	0.26930979
ENSG000001	ACOT4	0.11559982	0.4710215	0.26935473
ENSG000001	RPL34	0.35294798	0.69109369	0.26956016
ENSG000001	CCDC83	0.61465503	0.84533626	0.26957864
ENSG000001	SAMHD1	0.00495784	0.21833269	0.26960272
ENSG000001	C1orf105	0.52142362	0.79758937	0.26962
ENSG000001	CYBB	0.00767453	0.22991016	0.26977906
ENSG00000(	ADAMTS6	0.20302533	0.56867381	0.26980576
ENSG000002	PLEKHM1	0.33834904	0.6800066	0.2699427
ENSG000001	SLC35F1	0.46121949	0.76110798	0.27013317
ENSG000001	PRPF39	0.06997685	0.4002892	0.27013797
ENSG000002	N/A	0.02218838	0.29306353	0.27015603
ENSG000001	DTHD1	0.07137335	0.40241675	0.27016783
ENSG000001	PHLDB2	0.03065765	0.31953017	0.27018815
ENSG000001	GRHL1	0.04927313	0.360767	0.27020901
ENSG000001	CHM	0.03827526	0.33650813	0.27036798
ENSG000001	NFYB	0.0899191	0.43377006	0.27051234
ENSG000001	RAD51AP1	0.31469517	0.66270362	0.27066094

ENSG000001	KCNMB3	0.03630632	0.33249057	0.27083477
ENSG000002	SNURF	0.13172431	0.48982931	0.27096054
ENSG000002	H3-2	0.47458016	0.76942167	0.27104834
ENSG000001	DBF4B	0.04455959	0.35527495	0.27107349
ENSG000001	RFK	0.00297742	0.20994948	0.27116194
ENSG00000C	TIGAR	0.08092228	0.41792898	0.2712799
ENSG000001	ZNF124	0.03836471	0.33689984	0.27129853
ENSG000001	ZCCHC10	0.08404855	0.42368712	0.27135339
ENSG000001	AKT3	0.31846577	0.66591607	0.27157041
ENSG00000C	CNTLN	0.03555478	0.33014896	0.2716552
ENSG000001	LYSMD2	0.02452998	0.29927225	0.27175292
ENSG000001	TMEM229B	0.0111155	0.2451811	0.27183968
ENSG00000C	GPM6B	0.10503035	0.45514301	0.27187595
ENSG000001	ASPHD2	0.03107336	0.31953017	0.27202219
ENSG000001	RGPD6	0.55037606	0.81252075	0.2723445
ENSG000001	OR10A2	0.6037353	0.83883394	0.2724846
ENSG000001	ZNRF2	0.00194424	0.19438665	0.27268869
ENSG000001	MOB1B	0.02898308	0.31345545	0.27277234
ENSG000001	TRPC1	0.35161927	0.69054758	0.27297157
ENSG000001	FAM126A	0.00779521	0.23002982	0.27371424
ENSG000002	DDX47	0.08992928	0.43377006	0.27371654
ENSG000001	ANKRD20A2F	0.48092186	0.77410996	0.2738933
ENSG000001	AMZ1	0.35511133	0.6919142	0.27395314
ENSG000001	ZCCHC4	0.03051978	0.3191802	0.2740213
ENSG000001	FOXN2	0.01243897	0.25469306	0.27421233
ENSG000001	KLF5	0.1070031	0.45771995	0.27424651
ENSG000001	TAS2R5	0.28624407	0.64387022	0.27432349
ENSG000001	TEFM	0.03726048	0.33443803	0.27439341
ENSG000001	NBN	0.01566089	0.26569439	0.27440032
ENSG00000C	WNT8B	0.3870862	0.71649778	0.27451247
ENSG000001	TMEM171	0.54526456	0.80952117	0.27464595
ENSG000001	EAF2	0.13202666	0.49054292	0.27464694
ENSG000001	BMPR1B	0.56883353	0.82037292	0.27466342
ENSG000001	DDAH1	0.30487758	0.65635638	0.27484459
ENSG000001	HOXA6	0.40662553	0.7275473	0.27504006
ENSG00000C	PMS1	0.05184604	0.36585146	0.27526993
ENSG000001	CYP26C1	0.51781895	0.79566132	0.27528024
ENSG000001	LIG4	0.07014246	0.40059904	0.27547103
ENSG000001	COMMD10	0.03039431	0.3191802	0.27595249
ENSG000001	TMEM262	0.14658831	0.50510677	0.27622449
ENSG000001	CD164	0.0120273	0.25192551	0.27629718
ENSG000001	ARL5C	0.47073325	0.76678329	0.27636155
ENSG00000C	ANKIB1	0.01422219	0.25946702	0.27639726

ENSG000001	SMCO4	0.02766953	0.3113392	0.27650312
ENSG000002	SMIM30	0.12548287	0.48349915	0.27650428
ENSG00000(	SP100	0.02040608	0.28641584	0.27669373
ENSG000001	ASCL2	0.04366014	0.35372007	0.2767202
ENSG000001	DNAJB14	0.05849966	0.3800398	0.27680689
ENSG00000(	FAS	0.04749786	0.35865951	0.27708295
ENSG000001	PRR5L	0.00164191	0.19292382	0.27715334
ENSG000001	NEDD1	0.00854512	0.23436355	0.27727166
ENSG000001	PAQR3	0.05844263	0.3800398	0.27728125
ENSG000001	ZNF484	0.1061768	0.4561473	0.27730167
ENSG000001	CR2	0.03212797	0.32322672	0.27741227
ENSG000001	RRAD	0.49067345	0.7800506	0.27745527
ENSG000001	MYBL1	0.11724372	0.47331831	0.27759483
ENSG000001	GDA	0.5315019	0.80232066	0.27760997
ENSG000001	GABRR2	0.13146253	0.4894044	0.2777364
ENSG000001	AIDA	0.00127163	0.18856694	0.27791056
ENSG000001	CHD1	0.0061903	0.22079879	0.27803117
ENSG000001	LRATD2	0.0460497	0.35738124	0.27848095
ENSG000001	CC2D2B	0.00688817	0.22420199	0.27858013
ENSG000001	GSAP	0.00426201	0.21614597	0.27860391
ENSG000001	LOX	0.32392089	0.6699145	0.27876988
ENSG000001	CCDC144A	0.51328533	0.79305489	0.27905586
ENSG000001	CCDC14	0.06100203	0.38464604	0.2790615
ENSG000001	PDE8B	0.0165566	0.27018043	0.27907619
ENSG00000(	BTBD7	0.05447	0.37317697	0.27909264
ENSG000001	ATP8A1	0.07058015	0.40156223	0.2790961
ENSG000001	ZMAT1	0.17504149	0.53975461	0.27930009
ENSG00000(	STOML1	0.0365633	0.33341755	0.2793152
ENSG000001	LRRC9	0.52229669	0.7979767	0.27933469
ENSG000001	ZNF441	0.10989596	0.46255597	0.27953959
ENSG000002	ACAD11	0.5533738	0.81385767	0.27959603
ENSG000001	CYP19A1	0.48495746	0.77693678	0.27966058
ENSG000001	PTAR1	0.13584625	0.4956709	0.27972987
ENSG000002	N/A	0.10587447	0.4561473	0.27974179
ENSG00000(	CHRDL2	0.50612653	0.78874906	0.27979633
ENSG000001	CA3	0.31087494	0.65949478	0.27984133
ENSG000002	SYNJ2BP	0.04296753	0.35185871	0.27996725
ENSG000001	TET1	0.08881842	0.43307647	0.2800386
ENSG000001	TCF4	0.01784342	0.27425066	0.28008658
ENSG000001	TRIM14	0.01381759	0.25910565	0.28010345
ENSG000001	POLN	0.03603426	0.33117113	0.28034145
ENSG000001	CCDC50	0.00456065	0.21750928	0.28044096
ENSG000001	TTC26	0.15357345	0.51472848	0.28060716

ENSG000001	CPED1	0.0132926	0.25828987	0.28063089
ENSG00000(	VPS50	0.03807477	0.33602196	0.2808941
ENSG000001	SSB	0.04299076	0.35185871	0.28150731
ENSG000001	ZSWIM5	0.03902326	0.3391974	0.28185561
ENSG000001	CCNJ	0.0054586	0.21833269	0.28191027
ENSG000001	TTC32	0.07146237	0.40241675	0.28194326
ENSG000002	C17orf67	0.0326272	0.32362351	0.2819781
ENSG000001	MUC16	0.36987086	0.70295438	0.28198758
ENSG000001	NEK5	0.10803768	0.45960368	0.28244618
ENSG000001	ANKRD20A4F	0.36800327	0.70058597	0.28260487
ENSG000001	CLEC4F	0.22870571	0.59702486	0.28261492
ENSG000001	VCPIP1	0.01143561	0.24590889	0.2827791
ENSG000001	BLZF1	0.02283883	0.29500624	0.28290159
ENSG000002	DPF3	0.06717611	0.39561184	0.28292343
ENSG000001	EVA1A	0.57808991	0.82523365	0.28311425
ENSG00000(	NOX1	0.1777387	0.5423686	0.28327331
ENSG000001	PARP15	0.05329743	0.36981204	0.28328148
ENSG000001	KCNJ10	0.45959394	0.75930269	0.28356236
ENSG000001	XDH	0.37864929	0.7095359	0.28363067
ENSG000001	SLFN13	0.00556461	0.21875144	0.28370672
ENSG000001	PNOC	0.02992926	0.31859418	0.28377888
ENSG000001	CDK7	0.387905	0.71672142	0.28385568
ENSG000001	UGT3A2	0.55253807	0.81383402	0.28395289
ENSG000001	ABTB2	0.089649	0.43361062	0.28399175
ENSG000001	SSTR2	0.44741983	0.75099423	0.28402579
ENSG00000(	CAMK2B	0.56676638	0.8197023	0.28411031
ENSG000001	NOD1	0.00375154	0.21208655	0.28424391
ENSG000001	C18orf25	0.00561961	0.21875144	0.28439453
ENSG000001	TECTA	0.11960563	0.47604098	0.28439888
ENSG000001	HHLA2	0.16588333	0.52885285	0.28441712
ENSG000001	C1orf54	0.09249409	0.43746768	0.28442967
ENSG000001	KIAA1109	0.02180203	0.29183808	0.28450218
ENSG000001	AZI2	0.02378954	0.29875884	0.28453913
ENSG000002	C5orf58	0.01922839	0.28088168	0.2845515
ENSG000001	SHTN1	0.00283665	0.20994948	0.28456217
ENSG00000(	PSMA4	0.03907312	0.33920759	0.28465849
ENSG000001	BEND4	0.136514	0.4965452	0.28529203
ENSG000001	TTC14	0.05742475	0.37703424	0.28537622
ENSG000001	TAF1B	0.0080457	0.2308093	0.28545007
ENSG000001	FGL2	0.03285472	0.32362351	0.28563988
ENSG000001	PDP1	0.02283617	0.29500624	0.28582111
ENSG000001	ZNF519	0.18147238	0.54626754	0.28585157
ENSG000001	ADCY5	0.12623322	0.48388732	0.28585404

ENSG000001	GPR65	0.00324866	0.2114129	0.28593516
ENSG000001	TMEM106B	0.05400921	0.37208157	0.28599828
ENSG000001	HELZ2	0.20504333	0.56891466	0.28603911
ENSG000001	NXF3	0.50424893	0.78698836	0.2861039
ENSG000001	CLIC4	0.00097438	0.18856694	0.28618905
ENSG000001	NDUFA5	0.12182597	0.47854428	0.28630337
ENSG000001	GPR75	0.14346006	0.50147706	0.2863923
ENSG000001	ATP11C	0.01943098	0.28172722	0.28652316
ENSG000001	CA10	0.51579965	0.79496643	0.28661394
ENSG000001	ZNF675	0.12912832	0.4871968	0.2867254
ENSG000001	TMSB15B	0.33156324	0.67455111	0.28675973
ENSG000001	RNASEH2B	0.00533361	0.21833269	0.28681484
ENSG000001	TMEM267	0.0659869	0.39343668	0.28692293
ENSG000001	ANGPTL6	0.06939537	0.39974794	0.28712174
ENSG00000C	RIF1	0.01686632	0.27141566	0.28715482
ENSG000001	PAX5	0.00427457	0.21614597	0.28716674
ENSG000001	SKIL	0.01762196	0.27418797	0.28734148
ENSG000001	GCNT2	0.01397318	0.25910565	0.28735936
ENSG000002	NOTCH2NLA	0.13468254	0.49415541	0.2875157
ENSG000001	SPACA4	0.52686562	0.80032903	0.28752407
ENSG000002	CTXND2	0.51862349	0.79577989	0.28757277
ENSG000001	MARCHF1	0.01705305	0.27224356	0.28770992
ENSG000001	DPPA3	0.43903386	0.74609757	0.28793094
ENSG000001	C15orf61	0.01289916	0.25583328	0.28801784
ENSG000001	LIPH	0.21269145	0.57808354	0.28804913
ENSG000001	SMAD3	0.00380883	0.21242163	0.28806663
ENSG000001	TMEM241	0.01411764	0.25910565	0.28836054
ENSG000001	PLA2G4A	0.02118947	0.28959078	0.28890902
ENSG000001	C1orf21	0.02499312	0.30049829	0.28891549
ENSG000001	ANKRD29	0.53756328	0.80547376	0.28902224
ENSG000001	CD160	0.02806501	0.31169051	0.28928295
ENSG000001	NMD3	0.11193237	0.46578315	0.28932163
ENSG000001	FRRS1	0.00892467	0.23513527	0.28934526
ENSG000001	DCBLD1	0.01148975	0.24612008	0.28935581
ENSG000001	PIK3R3	0.00122114	0.18856694	0.28952539
ENSG000001	MOB4	0.0321646	0.32322672	0.28983441
ENSG000001	ZC2HC1A	0.09272567	0.43753271	0.2898829
ENSG000001	UCHL3	0.1445718	0.50315089	0.2899363
ENSG000002	SYCE3	0.57525983	0.82344878	0.29012123
ENSG000002	NANOGP8	0.40473737	0.72672584	0.29038635
ENSG000001	ANKFY1	0.01838844	0.27758038	0.2904796
ENSG000001	NAP1L2	0.08364432	0.42260872	0.29052278
ENSG00000C	SMC1B	0.17361525	0.53818075	0.29073372

ENSG000001	IL15RA	0.02262165	0.29448836	0.29082677
ENSG000002	MUSTN1	0.41599833	0.73235476	0.29090118
ENSG000001	ZNF66	0.13402596	0.4925946	0.29100683
ENSG00000C	SLC25A24	0.01031857	0.24174018	0.29113042
ENSG000001	RICTOR	0.02076791	0.28641584	0.29155171
ENSG000001	EREG	0.25799634	0.62198226	0.29173446
ENSG000001	CPEB2	0.08360923	0.42260872	0.29175022
ENSG000001	SLC26A5	0.51192624	0.79201014	0.29193771
ENSG000001	FANCM	0.04487957	0.35545023	0.29197955
ENSG000001	GAS2L3	0.17709772	0.54199198	0.29200318
ENSG000001	LUM	0.59762057	0.83641817	0.29203534
ENSG000002	PET117	0.35761396	0.6937369	0.29231924
ENSG000001	USP25	0.01080417	0.24206404	0.29304653
ENSG000001	CMTR2	0.02461829	0.29927225	0.29308406
ENSG000001	PHACTR2	0.01119729	0.2451811	0.29309469
ENSG000001	PRICKLE1	0.00246188	0.2093801	0.29309487
ENSG000001	P2RY6	0.1230243	0.47983319	0.29317361
ENSG00000C	CUL1	0.0011817	0.18856694	0.29323861
ENSG000001	MDGA1	0.17045512	0.53440428	0.29324749
ENSG000001	PLAAT4	0.04179557	0.34903589	0.29328889
ENSG000001	LHFPL3	0.22509188	0.59216086	0.29343534
ENSG000001	AMY1B	0.52336426	0.79870477	0.29365757
ENSG000001	NHLRC2	0.02146477	0.2908707	0.29378885
ENSG000001	ARHGAP11A	0.3926506	0.72047325	0.29428983
ENSG000001	CARD10	0.56961895	0.82045207	0.2942991
ENSG000001	TCN2	0.10406697	0.45422994	0.29441473
ENSG000001	HDHD2	0.03841209	0.33695147	0.29459575
ENSG000001	NEURL2	0.44095737	0.74750808	0.29471597
ENSG00000C	MATR3	0.10008263	0.44762396	0.29476708
ENSG000001	LIF	0.39749675	0.72378963	0.29483974
ENSG000002	DENND1B	0.01084529	0.24206404	0.29495713
ENSG000001	TMEM161B	0.07615315	0.41038789	0.29500031
ENSG000001	SRGAP2C	0.04930835	0.360767	0.29504853
ENSG000001	DEK	0.0127733	0.25573377	0.2954652
ENSG000001	TTC33	0.04828663	0.360767	0.29547429
ENSG000001	RARRES2	0.47015555	0.7662622	0.29552907
ENSG000001	NMUR1	0.06674704	0.39496052	0.29564983
ENSG000001	KLRD1	0.02878017	0.31345545	0.29573479
ENSG000001	RSL24D1	0.24648589	0.61214306	0.29581019
ENSG000001	IL12A	0.23355337	0.60135629	0.29599157
ENSG00000C	NAALAD2	0.11968238	0.47604098	0.29603764
ENSG000001	ZNF471	0.11287562	0.46777983	0.29640266
ENSG000001	CALB1	0.09985017	0.447493	0.29654464

ENSG000001	CD163L1	0.35234556	0.69082603	0.2966736
ENSG000001	KCNAB1	0.18634316	0.55072357	0.29681689
ENSG000001	COMMD8	0.14378921	0.50194033	0.29702857
ENSG000001	NEXN	0.06400107	0.39023108	0.29705501
ENSG000001	ZNF567	0.08888047	0.43309175	0.29709864
ENSG000001	ETNK1	0.03195564	0.3227302	0.29715627
ENSG000001	EXD1	0.42884941	0.74051893	0.29737011
ENSG000001	TRMT13	0.05973603	0.38277429	0.29743814
ENSG000001	BLVRA	0.01313765	0.25731723	0.29744956
ENSG000002	TIFAB	0.05746451	0.3770373	0.29745957
ENSG00000C	TNFRSF17	0.32225621	0.66824838	0.29787749
ENSG00000C	ENPP4	0.07665291	0.41092575	0.297878
ENSG000001	OTOA	0.36297291	0.69664198	0.29789656
ENSG000001	ZNF724	0.28554462	0.64333814	0.29793537
ENSG000001	IGF2	0.28469492	0.64286898	0.29807237
ENSG000001	FSD1L	0.05511378	0.37354464	0.29819342
ENSG00000C	XYLB	0.04640466	0.35774922	0.29839784
ENSG00000C	MYO3B	0.30543701	0.65688103	0.29848367
ENSG000001	RBBP8	0.01766737	0.27418797	0.29894194
ENSG000001	EDARADD	0.14001471	0.4999841	0.29923347
ENSG000001	MDK	0.15911663	0.5217239	0.29930106
ENSG000001	SGPP1	0.00730948	0.22727528	0.2993772
ENSG000001	IL9R	0.4960305	0.78185081	0.29938114
ENSG000001	EFR3A	0.00423232	0.21614597	0.29962003
ENSG000001	PEG3	0.51129421	0.79171125	0.29962057
ENSG000001	HELB	0.00094024	0.18856694	0.29965222
ENSG000002	DDO	0.10441481	0.45477021	0.29983032
ENSG000001	CERKL	0.00124201	0.18856694	0.30008006
ENSG000001	HEG1	0.00237395	0.2093801	0.30051875
ENSG000001	GUCY1A2	0.2092851	0.57323924	0.30059257
ENSG000002	SPECC1L-AD	0.57498084	0.82344878	0.30062973
ENSG000001	AKAP7	0.01554021	0.26471158	0.30097407
ENSG000001	BATF3	0.02851208	0.31345545	0.30164447
ENSG000002	N/A	0.09620544	0.44215472	0.30183604
ENSG000001	RPE	0.00915868	0.23551879	0.30195197
ENSG000002	TMEM256	0.18328128	0.54815586	0.30196112
ENSG000001	VSIG10L	0.08347539	0.42260872	0.30201203
ENSG000001	LARP4	0.00654062	0.22296216	0.30201647
ENSG000001	CDC42EP2	0.51019784	0.79143652	0.30209422
ENSG000001	RPRM	0.48744755	0.77793335	0.30250075
ENSG000001	TMEM62	0.0306812	0.31953017	0.30270329
ENSG000001	SCG2	0.50907661	0.79132674	0.30285379
ENSG000001	FAM177B	0.01212497	0.25234748	0.30289692

ENSG000002	HLA-B	0.0739608	0.4057296	0.3029156
ENSG000001	TMX3	0.03884941	0.33829709	0.30305928
ENSG000001	CCDC73	0.38171024	0.71076023	0.3030979
ENSG000001	PLSCR4	0.50805467	0.79067965	0.30333567
ENSG000001	DNAJA3	0.33443609	0.67664065	0.30334896
ENSG000001	PDLIM4	0.58568087	0.82959975	0.30341491
ENSG000001	GNRH2	0.32801688	0.67237631	0.30341721
ENSG000001	A3GALT2	0.32859751	0.67264721	0.30353948
ENSG00000C	N4BP2	0.0661049	0.39345499	0.30364776
ENSG000001	ARL6	0.163838	0.52709285	0.30369738
ENSG000002	GOLGA8O	0.17085764	0.53463395	0.30371076
ENSG000001	EXOSC9	0.0287422	0.31345545	0.30374166
ENSG000001	PSTPIP2	0.04625582	0.35738124	0.30394337
ENSG000001	FAM111A	0.00907143	0.23513527	0.30425885
ENSG000002	PIP4K2B	0.4120589	0.73041489	0.30434254
ENSG000001	TOR1B	0.05601899	0.37502119	0.30434796
ENSG000001	OR7C1	0.22517489	0.59228329	0.30454883
ENSG000001	PODN	0.27658539	0.6360577	0.30456308
ENSG00000C	BIRC3	0.02802583	0.31159655	0.30470157
ENSG000001	FCGR1B	0.26163044	0.62545731	0.30514678
ENSG000001	IL12B	0.52051475	0.79709497	0.30575594
ENSG000001	SHC4	0.60249536	0.83852406	0.3060033
ENSG000001	GDAP1	0.02380012	0.29875884	0.30606171
ENSG000001	CLDN20	0.08517548	0.42526981	0.30608973
ENSG000001	CCRL2	0.03792205	0.3358711	0.30625454
ENSG000001	AFF1	0.04223283	0.34997262	0.30627128
ENSG000001	MRPL13	0.14969117	0.50933268	0.30644464
ENSG000001	PPFIBP1	0.01836256	0.27758038	0.30670705
ENSG000001	APOE	0.53141262	0.80232066	0.30671281
ENSG000001	OTUD6B	0.08880203	0.43307647	0.30727005
ENSG000001	TPBG	0.30194345	0.65571024	0.30732563
ENSG000001	GOLGA8Q	0.62602194	0.85060404	0.30733591
ENSG000001	LBX2	0.13845355	0.49898885	0.30755463
ENSG000002	TMEM235	0.32831967	0.67254253	0.30761552
ENSG000001	RNF138	0.00451198	0.21750928	0.30780491
ENSG000001	CENPK	0.19886373	0.56598606	0.30783129
ENSG000001	BARD1	0.01044582	0.24174018	0.30792555
ENSG000001	ODF3B	0.14047565	0.50035294	0.30795616
ENSG000001	NUDCD1	0.10990583	0.46255597	0.30803755
ENSG000002	N/A	0.43794493	0.74561307	0.30805495
ENSG000001	HLA-DRB1	0.04201673	0.34950084	0.30807739
ENSG000001	SLITRK4	0.04924577	0.360767	0.30817044
ENSG000001	UGGT2	0.04996608	0.36170272	0.30820599

ENSG000001	SELENOI	0.01200488	0.25192551	0.30828562
ENSG000002	TEN1	0.52442571	0.79878989	0.3083216
ENSG000001	GPR89B	0.02093175	0.28753711	0.30846229
ENSG000001	DACT3	0.46246593	0.7620316	0.30850184
ENSG000001	C19orf33	0.46770272	0.76561035	0.30857578
ENSG00000C	RGPD5	0.20877786	0.57262264	0.30875692
ENSG000001	GLP1R	0.47653772	0.7710556	0.30882028
ENSG000001	ZNF20	0.31846822	0.66591607	0.3088809
ENSG000001	CELA2A	0.56485312	0.81898782	0.30896726
ENSG000001	CCDC82	0.11560145	0.4710215	0.30919066
ENSG000001	TMTC3	0.07187978	0.40314751	0.30922973
ENSG000001	ERAP2	0.00165156	0.19292382	0.30929959
ENSG000001	GPR180	0.04653821	0.35774922	0.30957809
ENSG000001	ANKRD7	0.43715203	0.74497757	0.30958715
ENSG000002	GK3P	0.23408177	0.60173104	0.31004456
ENSG000002	N/A	0.05815862	0.37924557	0.31022945
ENSG000001	HCFC2	0.01352064	0.25888521	0.31031618
ENSG000002	H2BS1	0.38963601	0.7188269	0.31032109
ENSG000001	GPR137C	0.52398017	0.79870477	0.31067529
ENSG000001	CHRM4	0.4579494	0.7575144	0.31077379
ENSG000001	DUSP5	0.01614293	0.26906292	0.31095618
ENSG000001	KHDRBS2	0.2762863	0.6360577	0.31106323
ENSG000001	KLHL14	0.02710121	0.30967087	0.31130177
ENSG000001	NCAM1	0.00779045	0.23002982	0.31130302
ENSG000001	FMR1	0.00887626	0.23472943	0.31142399
ENSG000001	GSDMC	0.54202691	0.80808176	0.31173038
ENSG00000C	TRMT11	0.05482255	0.37354464	0.31192324
ENSG000001	CUTA	0.48418179	0.77655439	0.31210726
ENSG000001	SLFN5	0.01406435	0.25910565	0.31290147
ENSG000001	SLAMF8	0.20395622	0.5688169	0.3129544
ENSG000002	ASAH2B	0.11506539	0.47079584	0.31318569
ENSG000001	IFI16	0.05974132	0.38277429	0.31351745
ENSG000001	PARP10	0.07485983	0.4080225	0.31355661
ENSG000001	AGPAT5	0.00239653	0.2093801	0.31356508
ENSG000001	MYOZ2	0.5152536	0.79458379	0.31364655
ENSG000001	C22orf15	0.49515036	0.78124152	0.31372812
ENSG000001	HINT2	0.25859786	0.6223087	0.31392545
ENSG000001	TMX1	0.02361931	0.29875884	0.31416285
ENSG000001	IFITM1	0.08221592	0.42098388	0.31439215
ENSG000001	RIMBP3C	0.34044937	0.68141884	0.31439412
ENSG00000C	RCN1	0.00185162	0.19438665	0.31445557
ENSG000001	PPP1R1B	0.37971634	0.70965485	0.31477529
ENSG000001	GJB5	0.28025888	0.6389512	0.31536597

ENSG00000(	PHTF2	0.02082839	0.28641584	0.31570995
ENSG000001	FMC1	0.05943101	0.38178501	0.31572512
ENSG000001	GPR85	0.35234979	0.69082603	0.31580812
ENSG000001	CGAS	0.00063218	0.18856694	0.31593001
ENSG000001	CMKLR1	0.00566249	0.21875144	0.31593287
ENSG000001	ACAN	0.60497349	0.83921013	0.31615502
ENSG000001	ATXN7	0.0059107	0.22079879	0.31626555
ENSG000001	NOC3L	0.05879056	0.38067583	0.31656718
ENSG000001	POGLUT2	0.22588074	0.59319591	0.31667697
ENSG000001	CPLANE1	0.00786603	0.23002982	0.3168079
ENSG000001	DPYS	0.56511209	0.81916096	0.31758666
ENSG000001	TM4SF1	0.45742379	0.75742878	0.31801024
ENSG000001	SCN2B	0.43164674	0.74126857	0.31813902
ENSG000001	PTH2R	0.27561462	0.63512113	0.31825501
ENSG000001	FEZ1	0.05656457	0.37516454	0.31826857
ENSG000001	HOXA1	0.03242349	0.32362351	0.31858301
ENSG000001	FZD4	0.36806369	0.70061549	0.31865105
ENSG000001	ZGRF1	0.0708219	0.40156223	0.31877288
ENSG000001	LRRC2	0.23416264	0.60173104	0.31899747
ENSG000002	PCDHGC4	0.52792996	0.80062313	0.31916428
ENSG000001	FAM241A	0.00035394	0.18856694	0.31930658
ENSG000001	CATSPERE	0.05326475	0.36981204	0.31947786
ENSG00000(	RGS17	0.11026595	0.46278943	0.31996548
ENSG000001	ZNF680	0.08643278	0.42826031	0.32031241
ENSG000002	CCDC179	0.22059383	0.5880238	0.32032502
ENSG000001	MANEA	0.05118501	0.36467355	0.32057044
ENSG00000(	EPB41L3	0.03335409	0.32398208	0.32096767
ENSG000001	CYP11B1	0.37629803	0.70798786	0.32114255
ENSG000001	HRNR	0.54279727	0.80839296	0.32149312
ENSG00000(	FAM135A	0.02058035	0.28641584	0.32157534
ENSG000001	SC5D	0.06365753	0.38981851	0.32202646
ENSG000001	ADAMTS12	0.42235087	0.7362799	0.32208148
ENSG000001	OR52B6	0.4816049	0.77466133	0.3224082
ENSG000001	FAM169A	0.14594865	0.50430399	0.32286143
ENSG000001	FYTTD1	0.00273636	0.20994948	0.32342708
ENSG00000(	SLC26A4	0.42934116	0.74090897	0.32344368
ENSG00000(	NUB1	0.06209846	0.38624952	0.32355386
ENSG000001	ANKRD61	0.05790024	0.37832043	0.32368597
ENSG000001	ANKRD30BL	0.39926581	0.72496091	0.32396489
ENSG00000(	RBFOX1	0.49318695	0.78087533	0.32445517
ENSG000001	C15orf48	0.38701594	0.71644941	0.32461238
ENSG000001	KRT36	0.46950759	0.7662622	0.32468349
ENSG000001	ILDR2	0.16106708	0.52404357	0.32515827

ENSG00000(	STAP1	0.04276137	0.35185871	0.32532629
ENSG000001	MACROD2	0.08199162	0.42050568	0.32544562
ENSG000001	IL15	0.00450304	0.21750928	0.32546598
ENSG000001	MED12L	0.00759298	0.22933195	0.32552832
ENSG000001	SCARB2	0.01167275	0.24859874	0.32553607
ENSG000001	RIN2	0.02903474	0.31345545	0.3256042
ENSG000001	NIPSNAP3A	0.0110162	0.24369207	0.3265028
ENSG000001	TFAP2A	0.35694923	0.69298434	0.32660645
ENSG000001	DMRTC1B	0.37491286	0.70718635	0.32671791
ENSG000001	RNASE1	0.33198532	0.67489153	0.32704026
ENSG000001	ITGA4	0.00393398	0.21403539	0.32723245
ENSG000001	DOCK4	0.14245206	0.50068215	0.32782596
ENSG00000(	SLC26A3	0.4513806	0.75378336	0.32787913
ENSG00000(	ESR1	0.03014068	0.31859418	0.32790047
ENSG000001	FAM72B	0.11967567	0.47604098	0.32796919
ENSG000001	TSSK3	0.08733739	0.42952109	0.32818905
ENSG000001	C21orf91	0.01028123	0.24174018	0.32825282
ENSG000001	RIMKLB	0.00611526	0.22079879	0.32835072
ENSG000001	LRRC63	0.27479748	0.63487288	0.32853207
ENSG00000(	MMP2	0.45795168	0.7575144	0.32858306
ENSG000001	NHLRC3	0.00127792	0.18856694	0.32865214
ENSG000001	GLS	0.00248492	0.2093801	0.32903525
ENSG000001	VPS13C	0.00092701	0.18856694	0.32963768
ENSG000001	DNAAF3	0.32711073	0.67217574	0.32964382
ENSG000001	KLK14	0.27268295	0.63378205	0.32978904
ENSG000001	DNAJC5B	0.41131485	0.72999304	0.32984495
ENSG000001	PRKCE	0.00900254	0.23513527	0.32989794
ENSG000001	YES1	0.03093448	0.31953017	0.33001456
ENSG000002	ZNF593OS	0.37758322	0.70892909	0.3301723
ENSG000001	ZNF28	0.02866001	0.31345545	0.33097994
ENSG000001	C3orf38	0.00177295	0.19438665	0.33108145
ENSG000002	SHISA9	0.5114727	0.79176111	0.33110067
ENSG000002	ALG11	0.01485863	0.26260224	0.3312147
ENSG000001	POLR2K	0.14908064	0.50906885	0.33128142
ENSG000001	CXCL13	0.43630326	0.74492703	0.33152714
ENSG000001	GOLGA8R	0.46642327	0.76486809	0.33191696
ENSG000001	PHF11	0.01193366	0.25192551	0.33209133
ENSG000001	ALG10	0.04211877	0.34997262	0.33217558
ENSG000001	PPARGC1B	0.00680271	0.22347774	0.33242056
ENSG000001	ZNF415	0.10178721	0.45106956	0.33263865
ENSG000001	GRIK1	0.0145435	0.26121998	0.33272961
ENSG000002	GPR33	0.48054073	0.77383118	0.33280644
ENSG000001	KBTBD8	0.05921639	0.38150633	0.33293006

ENSG000001	ACTR3C	0.0366083	0.33341755	0.33293948
ENSG000001	DUOXA1	0.46715132	0.76524777	0.33316731
ENSG000001	RNF217	0.05656753	0.37516454	0.33326727
ENSG000002	SYNRG	0.12201954	0.47873532	0.3332881
ENSG000001	PABIR3	0.00581145	0.21944321	0.33335042
ENSG000001	NUDT6	0.12361092	0.48039028	0.3346124
ENSG000001	CEND1	0.15135813	0.5123298	0.33467588
ENSG000001	STX19	0.47117259	0.76694135	0.33491907
ENSG000002	N/A	0.14587748	0.50430399	0.33531663
ENSG000001	ADAMTS1	0.04052004	0.34535266	0.33539906
ENSG000001	DDX60L	0.08314522	0.42254425	0.33543357
ENSG000001	ANK2	0.42830139	0.74005849	0.3358309
ENSG000001	CNTNAP3B	0.40750211	0.72768322	0.33599153
ENSG000002	COLCA2	0.36800503	0.70058597	0.33600935
ENSG000001	FLOT1	0.26449954	0.6274921	0.33624664
ENSG000001	KMO	0.00161445	0.19292382	0.33654433
ENSG000001	GPD2	0.00684037	0.22347774	0.33665076
ENSG000001	RBM43	0.01505209	0.26260224	0.33669651
ENSG000001	CBWD2	0.01983835	0.28330274	0.3367677
ENSG000001	NEB	0.02407551	0.29927225	0.3371636
ENSG000001	TPRG1	0.09673167	0.44304329	0.33732268
ENSG000002	SPTY2D1OS	0.14726984	0.50568178	0.33786005
ENSG000001	HMCN1	0.45510316	0.7559601	0.33821082
ENSG000001	ZNF462	0.42556925	0.73817463	0.33842617
ENSG000001	ALG10B	0.05287053	0.3697066	0.33855209
ENSG000001	SLC30A4	0.0410718	0.34663236	0.33862692
ENSG000001	RHOBTB3	0.0003763	0.18856694	0.33873475
ENSG000001	GCNT1	0.00086727	0.18856694	0.3387472
ENSG000002	RBM20	0.31069011	0.65949478	0.33894524
ENSG000001	ESYT3	0.40120545	0.72606468	0.33901238
ENSG000002	N/A	0.30318489	0.65593778	0.33901662
ENSG00000C	CLDN18	0.44215004	0.74830001	0.33931673
ENSG00000C	ADRB1	0.23134871	0.5991557	0.33935812
ENSG000001	KPNA5	0.0245346	0.29927225	0.33937755
ENSG000001	CCL20	0.51231637	0.79216083	0.33991557
ENSG000001	GYPA	0.49853741	0.78316099	0.34023023
ENSG000001	MND1	0.41483049	0.7319716	0.34043333
ENSG000001	POLK	0.02179638	0.29183808	0.34045321
ENSG000001	KATNAL2	0.01076461	0.24206404	0.34068134
ENSG000001	ZBTB41	0.0559856	0.37502119	0.34083188
ENSG000001	HS3ST3A1	0.53776441	0.80550839	0.34086883
ENSG000001	VAT1L	0.17170593	0.53584263	0.34093469
ENSG000001	GRIP1	0.01076672	0.24206404	0.34116048

ENSG000001	FANCA	0.05035796	0.36227834	0.3415249
ENSG000001	NT5C3A	0.02580232	0.30449397	0.34193993
ENSG000001	CHN1	0.02205998	0.29231997	0.34199491
ENSG000001	SH3RF1	0.02790147	0.31135087	0.34199783
ENSG000001	MGARP	0.56007768	0.81699589	0.34262913
ENSG000001	USPL1	0.00186291	0.19438665	0.34269723
ENSG000001	ZNF23	0.16463158	0.52786788	0.34280929
ENSG000001	LRRC66	0.38186188	0.71079805	0.34298155
ENSG000001	PTGDR	0.03312311	0.3238836	0.34326166
ENSG00000C	CDK17	0.02309248	0.29687456	0.34333983
ENSG000001	NBEAL1	0.04514625	0.35545023	0.34340674
ENSG000001	FASLG	0.01860122	0.27889987	0.34381431
ENSG000002	BMPR2	0.00409546	0.21403539	0.34392062
ENSG000001	GFUS	0.42167893	0.73574046	0.3441923
ENSG000001	APOF	0.46034318	0.76015269	0.34422147
ENSG000001	CLECL1	0.12908254	0.4871968	0.34474748
ENSG000001	ST3GAL5	0.00535737	0.21833269	0.34481263
ENSG000001	TAFA2	0.01220846	0.25275547	0.34500822
ENSG000002	N/A	0.38397542	0.7123445	0.34520355
ENSG000001	FCRL2	0.01738506	0.27265457	0.34538564
ENSG000001	FERMT1	0.27292787	0.63378205	0.34569222
ENSG000002	NPIPB8	0.46159355	0.76122418	0.34573796
ENSG000002	HLA-F	0.03038507	0.3191802	0.34582633
ENSG000001	YWHAE	0.19036502	0.55552783	0.34586406
ENSG000002	APOBEC3G	0.00758921	0.22933195	0.34613323
ENSG000001	SLC4A9	0.32238481	0.66835573	0.34616721
ENSG000001	PIK3AP1	0.02661076	0.30699826	0.3463501
ENSG000001	RSPH9	0.09561917	0.4413663	0.34662509
ENSG000001	RNASEH2A	0.10343736	0.45326416	0.34678358
ENSG000002	FAM205A	0.34169288	0.6829231	0.34698775
ENSG000001	GNB4	0.01408938	0.25910565	0.34707227
ENSG000001	ACO1	0.01608655	0.26872151	0.34714362
ENSG000001	STARD6	0.36794072	0.70058597	0.34752277
ENSG00000C	RAPGEF4	0.41850919	0.73381176	0.34764463
ENSG000001	TSNAXIP1	0.10475499	0.45514301	0.34788331
ENSG000002	MAFB	0.00988794	0.2389143	0.34813708
ENSG000001	NXT2	0.00649201	0.22296216	0.34882846
ENSG000001	SCRIB	0.04904223	0.360767	0.34883201
ENSG000001	GIMAP2	0.01073732	0.24206404	0.34891525
ENSG000002	ANKRD20A3F	0.43953951	0.74621708	0.34911573
ENSG000001	ALOX12B	0.38783728	0.71672142	0.34927642
ENSG000001	ZDHHC21	0.03851014	0.3372649	0.34929931
ENSG00000C	FKBP7	0.20518541	0.56901717	0.34960437

ENSG000001	ZNF781	0.25186712	0.61747944	0.34960788
ENSG000001	TEDC2	0.28091166	0.63927558	0.34967795
ENSG00000(	CHMP5	0.02364821	0.29875884	0.350182
ENSG000002	HOXB7	0.38400883	0.7123445	0.35035491
ENSG00000(	MTHFD2	0.00377428	0.21208655	0.3505221
ENSG000001	ANKDD1B	0.20312809	0.56867381	0.35083419
ENSG000001	RGL1	0.02323843	0.29780768	0.35099661
ENSG000001	C6orf52	0.48074726	0.77396792	0.35101053
ENSG000002	SMIM9	0.11447815	0.47002354	0.35101756
ENSG000001	GPR15	0.02498915	0.30049829	0.35116261
ENSG00000(	CBARP	0.37403353	0.706184	0.35152489
ENSG000001	DDHD1	0.00509699	0.21833269	0.35174233
ENSG000001	ZNF157	0.18370443	0.54871392	0.35178006
ENSG000002	COL28A1	0.32821398	0.67253311	0.35209817
ENSG000001	RTKN	0.09976253	0.44747076	0.35273629
ENSG000001	DGKH	0.05124134	0.3647548	0.35274264
ENSG000002	S1PR2	0.00627716	0.22079879	0.35280859
ENSG000001	TBX1	0.39838161	0.72429687	0.35287818
ENSG000001	NPIPB9	0.38791327	0.71672142	0.35307169
ENSG000001	PDZK1	0.11434003	0.46977203	0.35326567
ENSG000001	SOBP	0.1239137	0.48115857	0.35326675
ENSG000001	ABCC11	0.4358309	0.74462291	0.35354123
ENSG000001	LHFPL2	0.07435085	0.40673667	0.35365748
ENSG000001	TENT5A	0.01738216	0.27265457	0.35389221
ENSG000001	KCNJ3	0.35297407	0.69109369	0.35404021
ENSG000001	EBF3	0.35736747	0.69342443	0.35421687
ENSG000001	UTRN	0.00304658	0.20994948	0.35423446
ENSG000001	STOM	0.00826055	0.23282737	0.35431623
ENSG000001	SFTPB	0.28164075	0.63946601	0.35436672
ENSG000002	LINGO4	0.39959086	0.72527731	0.35462368
ENSG000001	IRF7	0.27070188	0.63197504	0.35481438
ENSG000002	N/A	0.33495145	0.67700551	0.35519585
ENSG000001	MACC1	0.11510098	0.47079584	0.35527477
ENSG000001	APOL1	0.14956781	0.50925123	0.35529777
ENSG000001	LGALS9B	0.01007226	0.24143268	0.35547963
ENSG000001	LRRCC1	0.03565075	0.33014896	0.35575917
ENSG000001	EFHB	0.27859513	0.63770725	0.35579455
ENSG000001	IGF2BP3	0.05109486	0.36427913	0.35589468
ENSG000001	AK4	0.07071549	0.40156223	0.35613334
ENSG000001	KPTN	0.04286174	0.35185871	0.35625732
ENSG000001	SLC38A11	0.12445937	0.48173414	0.35635298
ENSG000001	VSTM2B	0.41842762	0.73381176	0.35646749
ENSG000001	XCL2	0.08353417	0.42260872	0.35708711

ENSG000001	AP1S3	0.00760943	0.22933195	0.3571479
ENSG000001	DDI1	0.43308339	0.74263121	0.35717645
ENSG000001	SIK1	0.1699243	0.53437545	0.357245
ENSG000002	C21orf62	0.57839868	0.82533908	0.35771337
ENSG000000	CEACAM1	0.1275764	0.48588488	0.35793322
ENSG000002	ERICH4	0.43651798	0.74496191	0.35856405
ENSG000001	ST8SIA6	0.03384349	0.32504402	0.35867274
ENSG000001	ARHGAP42	0.13403233	0.4925946	0.3587409
ENSG000001	ANGPTL8	0.42017644	0.73468557	0.35890591
ENSG000001	GJC3	0.34877429	0.68818181	0.35891688
ENSG000000	DAPP1	0.00813818	0.23121033	0.35909858
ENSG000000	MOXD1	0.09211853	0.43709193	0.35932538
ENSG000001	TNFRSF14	0.16234921	0.52515336	0.35943207
ENSG000001	EEF1A2	0.09941191	0.44700947	0.36011118
ENSG000000	USH2A	0.28145257	0.63945781	0.36036888
ENSG000001	SPATA32	0.08895492	0.43309175	0.36058474
ENSG000001	SNTB1	0.00044923	0.18856694	0.36080942
ENSG000001	LVRN	0.42068515	0.7349809	0.36102578
ENSG000001	ETAA1	0.04668705	0.35791197	0.36110723
ENSG000001	SCUBE2	0.41384514	0.73141372	0.36122322
ENSG000001	BEX1	0.43342042	0.74297376	0.36129129
ENSG000001	OR52K2	0.46962214	0.7662622	0.36133108
ENSG000001	PRSS23	0.03385166	0.32504402	0.36139289
ENSG000001	SMOC2	0.37596483	0.70769082	0.36146098
ENSG000001	KCNA2	0.01710029	0.27224356	0.36150044
ENSG000001	CAMK2D	0.00174725	0.19438665	0.36154285
ENSG000001	C2CD6	0.14339191	0.50145476	0.36160785
ENSG000001	SLC9B1	0.20433407	0.5688169	0.3616997
ENSG000001	TNFRSF19	0.27002891	0.63142427	0.36224037
ENSG000001	RNF183	0.41511499	0.73210851	0.36237326
ENSG000001	PCDHB2	0.31010476	0.65948451	0.36248651
ENSG000001	RAG1	0.18906586	0.55377099	0.36283365
ENSG000000	PCDHGA2	0.40186259	0.72645768	0.36284602
ENSG000001	POMK	0.05928068	0.38150633	0.36294043
ENSG000001	RHEBL1	0.01132107	0.24556914	0.36302465
ENSG000001	RTKN2	0.07826489	0.41427772	0.36306468
ENSG000001	SERF1A	0.38014201	0.70987972	0.3634212
ENSG000001	ABCG4	0.42991705	0.74091639	0.36358109
ENSG000001	STAC	0.35425996	0.69167785	0.36370015
ENSG000001	CBWD1	0.00654665	0.22296216	0.36386743
ENSG000001	USP6	0.12098351	0.47724625	0.36389378
ENSG000001	FAM217A	0.47719021	0.77172684	0.36412787
ENSG000001	PPP2R3B	0.49980269	0.78433741	0.36416909

ENSG000001	ASPG	0.36384798	0.69706268	0.36431012
ENSG000001	HNRNPLL	0.0119324	0.25192551	0.36440468
ENSG000001	EGR3	0.10773482	0.4588904	0.36473653
ENSG000001	ARL5B	0.00816439	0.23135278	0.36501595
ENSG00000C	ARAP2	0.00326032	0.2114129	0.36511064
ENSG000001	AVP	0.39921057	0.72496091	0.36513343
ENSG000001	MASTL	0.01451074	0.26119577	0.36519164
ENSG000001	TMPRSS3	0.016728	0.2704224	0.36547738
ENSG000001	HOMER1	0.00968421	0.2378859	0.36560807
ENSG000002	RAB12	0.00078069	0.18856694	0.36589992
ENSG000001	PUF60	0.12659524	0.48433718	0.36629081
ENSG000001	DISC1	0.00518577	0.21833269	0.36634597
ENSG00000C	SNCAIP	0.32756516	0.67217574	0.3664138
ENSG000001	GREB1L	0.23260336	0.59995954	0.366458
ENSG000001	SYBU	0.01846535	0.2781279	0.36663365
ENSG000001	APOL3	0.00613847	0.22079879	0.36694771
ENSG000001	INAVA	0.21406555	0.5797802	0.36703774
ENSG000001	SPATC1L	0.51562737	0.79496643	0.3671076
ENSG000002	FBXO16	0.50824189	0.79067965	0.36728683
ENSG00000C	CD209	0.04808881	0.36048845	0.36746389
ENSG000001	TNFSF13B	0.06031774	0.38403111	0.36789742
ENSG000002	SLC22A18AS	0.18079	0.54567047	0.36798063
ENSG000001	SMIM23	0.40244086	0.72669629	0.36820263
ENSG000001	DTX3L	0.0415257	0.34755298	0.3684445
ENSG000001	LCN15	0.20308419	0.56867381	0.36857176
ENSG000001	CCDC70	0.28549176	0.64333814	0.36866271
ENSG000001	PMAIP1	0.02334455	0.29822618	0.3689526
ENSG000001	PSME2	0.02775078	0.3113392	0.36933303
ENSG000001	GPR3	0.25422692	0.619382	0.36947808
ENSG000001	KCNQ3	0.46054687	0.76015269	0.36968747
ENSG000002	FTCDNL1	0.05479717	0.37354464	0.36971113
ENSG000001	HYDIN	0.29855102	0.65406944	0.36976361
ENSG000002	HTD2	0.44758061	0.75099423	0.36988071
ENSG000001	SPON2	0.03606654	0.33117113	0.37008455
ENSG000002	N/A	0.50183911	0.78548326	0.37085766
ENSG000001	STX17	0.00437817	0.21740126	0.37090966
ENSG000001	SLC6A20	0.46530386	0.76438119	0.37103003
ENSG000001	KLHDC7B	0.0288422	0.31345545	0.3712388
ENSG000001	C9orf43	0.18443661	0.54968745	0.37132052
ENSG000001	LGALS3BP	0.05629242	0.37502119	0.37139252
ENSG000001	SH2D1B	0.00738495	0.22796473	0.37175781
ENSG000001	AKR1C3	0.018232	0.27755705	0.37181255
ENSG000001	ERMARD	0.34905469	0.6884911	0.37181386

ENSG000001	RBM11	0.11020514	0.46278943	0.3721703
ENSG000001	NES	0.44779312	0.7510408	0.37265697
ENSG00000(	IKZF2	0.00394826	0.21403539	0.37289768
ENSG000001	SHISAL1	0.3311099	0.67443203	0.37328687
ENSG000002	AIF1	0.34185043	0.68306984	0.37340003
ENSG000001	IL31RA	0.34037894	0.68141884	0.37365904
ENSG000002	DENND11	0.13854109	0.49898885	0.37383404
ENSG000001	CTSL	0.00705192	0.22661511	0.37459823
ENSG000001	POU3F2	0.37517252	0.70720386	0.37473416
ENSG000001	AGAP5	0.06232548	0.38624952	0.37496901
ENSG000001	SLC1A6	0.27883047	0.63783746	0.37560478
ENSG000001	RHOXF1	0.16394605	0.52712713	0.37612268
ENSG000001	SLFN12	0.01450076	0.26119577	0.37612453
ENSG000002	PSMB8	0.37163658	0.70378624	0.37732768
ENSG000001	IL4I1	0.09845766	0.4450604	0.37833121
ENSG000001	NR5A1	0.4476894	0.75102106	0.37858918
ENSG000001	FMNL2	0.02720099	0.30967858	0.37890524
ENSG000001	LILRB4	0.46898062	0.76616638	0.3789883
ENSG00000(	SLC18A1	0.00102446	0.18856694	0.37906492
ENSG000002	OR4E2	0.41684288	0.73289419	0.3791275
ENSG000001	C11orf16	0.34795774	0.68740599	0.37946424
ENSG000001	SLC47A1	0.02021578	0.28592654	0.37970385
ENSG000001	CDO1	0.31830718	0.66581089	0.37974164
ENSG000002	EMP2	0.30281697	0.65591444	0.37995875
ENSG000001	MRO	0.32152072	0.66801432	0.38013241
ENSG000002	N/A	0.17309317	0.53759345	0.38014942
ENSG000001	FAM222A	0.15709278	0.5199629	0.3802121
ENSG000001	PLAC8	0.00484482	0.21772133	0.38057461
ENSG000001	TCEAL9	0.08935174	0.43342334	0.38058344
ENSG000001	TNFSF10	0.08199304	0.42050568	0.38084257
ENSG000001	TNFSF15	0.03257187	0.32362351	0.38086304
ENSG000001	FOXD4	0.35057878	0.69014884	0.38087422
ENSG000001	GPR174	0.02582064	0.30449397	0.38160533
ENSG000001	DZIP1L	0.25409493	0.61929442	0.38173741
ENSG000002	N/A	0.25151881	0.6170914	0.38182083
ENSG000001	NRN1L	0.29427504	0.65044158	0.38198158
ENSG000001	WDR49	0.29907917	0.65423918	0.38203692
ENSG000001	INPP5J	0.28575717	0.64343907	0.38208246
ENSG000002	SYT15	0.00578	0.21912363	0.38220686
ENSG000001	MRPS28	0.05946507	0.38178501	0.38257715
ENSG000001	SCOC	0.08893306	0.43309175	0.3825773
ENSG000001	ADGRG2	0.09836517	0.4448142	0.38345686
ENSG000001	TOP3B	0.16199839	0.52470934	0.38358912

ENSG00000(	CETP	0.03764178	0.33471496	0.3837162
ENSG000001	HRAS	0.50996104	0.79143652	0.38373351
ENSG000001	ZNF860	0.14139221	0.50055578	0.38374552
ENSG000001	DNAI3	0.41224406	0.73053039	0.38459367
ENSG000001	ANTXR1	0.34892681	0.6883991	0.38484821
ENSG000001	TRIM5	0.02657543	0.30699826	0.38509592
ENSG000001	ZPLD1	0.10075064	0.44884919	0.38531014
ENSG000001	MARF1	0.1397151	0.49975817	0.38531557
ENSG000001	NYX	0.25175815	0.6173055	0.38551405
ENSG000001	CFAP73	0.29499877	0.65121791	0.38556889
ENSG000001	SMN1	0.32044606	0.66731855	0.38620183
ENSG000001	TMEM268	0.02894876	0.31345545	0.38636911
ENSG000002	ZNF705E	0.43143754	0.74126857	0.38654318
ENSG000001	LRRC71	0.49196634	0.7804495	0.38663092
ENSG000001	NHEJ1	0.41722479	0.73321077	0.38798049
ENSG000001	LACC1	0.00238019	0.2093801	0.38816559
ENSG00000(	SP140	0.04367494	0.35372007	0.38817441
ENSG000001	RHO	0.29381242	0.65039296	0.38823628
ENSG000001	GPR84	0.06901619	0.3986538	0.38835424
ENSG000001	ZSCAN32	0.00485003	0.21772133	0.38842511
ENSG000001	C1GALT1	0.00335126	0.2114129	0.38857147
ENSG000002	MDP1	0.33521671	0.67708232	0.38871256
ENSG000001	DRP2	0.43060397	0.74119757	0.38903636
ENSG000001	CASP5	0.07717479	0.41192866	0.38939458
ENSG000001	TPH2	0.19875948	0.56598606	0.38943574
ENSG00000(	MSR1	0.11566504	0.47104419	0.38973311
ENSG000001	RBM34	0.00975605	0.23800279	0.38975865
ENSG000001	SLC44A3	0.18393138	0.54918974	0.38995561
ENSG000001	PLEKHS1	0.44201653	0.74830001	0.39003208
ENSG000002	RPEL1	0.48204351	0.77486788	0.39044756
ENSG000001	PARP9	0.06664529	0.39496052	0.39047074
ENSG000001	COL24A1	0.01396163	0.25910565	0.39054224
ENSG000001	RASGRP3	0.00929992	0.23551879	0.39054522
ENSG000001	MRGPRX2	0.24163233	0.60724101	0.39116907
ENSG000001	GOLGA8M	0.25386776	0.61921212	0.39134783
ENSG000001	NRIP1	0.0083168	0.23282737	0.39151132
ENSG000001	APOBEC3F	0.01692921	0.27175434	0.39157915
ENSG000001	VSNL1	0.39311082	0.72063539	0.39196011
ENSG00000(	JAK2	0.0059911	0.22079879	0.39200422
ENSG000001	CRYM	0.32517856	0.6708073	0.39213553
ENSG000001	SUMO4	0.07079691	0.40156223	0.39241141
ENSG000001	PRSS35	0.2363377	0.60336718	0.39259619
ENSG000001	VEGFC	0.20263391	0.56815551	0.39286393

ENSG000001	RAB40A	0.43724197	0.74497757	0.39291632
ENSG000001	PTPRR	0.38428783	0.7126178	0.3932302
ENSG000001	PML	0.09766266	0.44443627	0.3934778
ENSG000001	CSRP2	0.29571913	0.65150496	0.39365086
ENSG000001	SERPINA12	0.25552793	0.62030905	0.39427906
ENSG000001	AANAT	0.09167119	0.43611772	0.39431858
ENSG000001	LEKR1	0.28030113	0.6389512	0.39449897
ENSG000001	CCNB3	0.27660303	0.6360577	0.39509434
ENSG000001	BST2	0.02759295	0.3113392	0.39519328
ENSG00000C	RUNX1T1	0.27015851	0.63142427	0.39533982
ENSG00000C	SCT	0.44740272	0.75099423	0.39560154
ENSG000002	RD3L	0.25325436	0.61912837	0.39579529
ENSG000001	FCRL1	0.00672525	0.22330866	0.39603108
ENSG000001	PPP1R1A	0.47403437	0.76912287	0.39611021
ENSG00000C	VRK2	0.00429331	0.21614597	0.3962293
ENSG000001	ADH4	0.28119217	0.63941315	0.39697573
ENSG000002	NOTCH4	0.15201669	0.51296214	0.3969813
ENSG000001	GDF9	0.15311393	0.5142759	0.39714193
ENSG000001	H3C13	0.46152398	0.76122418	0.39760739
ENSG000001	FAM180B	0.36359607	0.69694698	0.39842138
ENSG000001	TOM1L1	0.10107031	0.44917064	0.39846358
ENSG000002	GBP7	0.4198086	0.73466853	0.39908671
ENSG000001	DMRT2	0.35501378	0.6919142	0.39932442
ENSG000001	CEP57L1	0.00606464	0.22079879	0.39937949
ENSG000001	SDC3	0.07882349	0.41446832	0.40009663
ENSG000001	CAMK2N2	0.35697198	0.69298434	0.40011966
ENSG000002	NPHP3-ACAD	0.35087327	0.69032381	0.40028962
ENSG000001	ITGA11	0.12929963	0.4871968	0.40062615
ENSG000001	TIFA	0.02373494	0.29875884	0.40108156
ENSG000002	HMSD	0.35079962	0.69032381	0.40125853
ENSG000001	SLC34A1	0.15619909	0.51865126	0.40126136
ENSG000001	ZNF257	0.07623402	0.41059278	0.40127251
ENSG000001	TDRD7	0.01750943	0.27336597	0.4015677
ENSG000001	TSEN34	0.43545685	0.74461931	0.40169972
ENSG000002	TSNAX-DISC1	0.18765158	0.55167498	0.4017652
ENSG000001	LDB2	0.22918298	0.59761256	0.40198886
ENSG000001	GUCY1A1	0.00595849	0.22079879	0.40236062
ENSG000001	MAJIN	0.23757689	0.60402516	0.40278006
ENSG000001	IRX3	0.22292776	0.5903895	0.40390942
ENSG000001	SLC52A1	0.09107766	0.43488031	0.40411475
ENSG000002	H2AB3	0.07172186	0.40260628	0.40472269
ENSG000002	GOLGA8K	0.26968593	0.63105497	0.40535629
ENSG000002	LSM14A	0.20080182	0.56765893	0.40561488

ENSG000001	CD69	0.08369735	0.42260872	0.40565848
ENSG000001	F10	0.33778445	0.67946062	0.40605333
ENSG000001	TRPC3	0.23412972	0.60173104	0.40605825
ENSG000001	EDA2R	0.28029097	0.6389512	0.40638402
ENSG000001	DLGAP1	0.14680505	0.50533677	0.40648296
ENSG000001	CBLIF	0.15247295	0.5133091	0.40659928
ENSG000001	CDK18	0.00336406	0.2114129	0.40663278
ENSG000001	FBLL1	0.24183814	0.60738219	0.40733266
ENSG000002	MRPL45	0.37911433	0.70965485	0.40781897
ENSG000001	DHH	0.25814735	0.62198226	0.40876189
ENSG000002	ARPIN-AP3S2	0.37871302	0.70957343	0.4089376
ENSG000001	CACNG1	0.46890801	0.76616638	0.40913177
ENSG000001	ADAM29	0.42560405	0.73817463	0.40959078
ENSG000001	PFDN4	0.11448809	0.47002354	0.41051951
ENSG000001	C5	0.01038804	0.24174018	0.41064578
ENSG00000C	RIMBP2	0.16726486	0.53173243	0.41068381
ENSG000001	TNFSF11	0.2863622	0.64387022	0.41068766
ENSG000001	NKX2-3	0.14497959	0.50375209	0.4113408
ENSG00000C	RRN3	0.37029345	0.70302695	0.41144818
ENSG000001	CCM2L	0.18127477	0.54622749	0.41186699
ENSG000001	FCGR1A	0.20599255	0.56956674	0.41198584
ENSG000001	PTPRO	0.00276349	0.20994948	0.41220778
ENSG000001	EXPH5	0.07905502	0.41476011	0.4126859
ENSG000002	UBE2L5	0.40289134	0.72672584	0.41317809
ENSG000001	ASS1	0.35141536	0.69054758	0.41415906
ENSG00000C	SEMA3G	0.00476854	0.21772133	0.41442031
ENSG000001	CXCL17	0.27553569	0.63512113	0.41509914
ENSG000001	FBXW10	0.34651816	0.68614821	0.41537386
ENSG000001	UBE2L6	0.09898794	0.4460792	0.41568437
ENSG000001	GTPBP6	0.10700899	0.45771995	0.41568524
ENSG000001	IFNK	0.29230278	0.64947398	0.41593066
ENSG000001	GPR161	0.0418578	0.34903589	0.41593766
ENSG000001	CCR6	0.01344098	0.25828987	0.41689816
ENSG000001	PARP11	0.00331729	0.2114129	0.41871589
ENSG000001	GNG5P2	0.28677045	0.64421192	0.41907123
ENSG000001	FAM110C	0.23175368	0.599371	0.41958436
ENSG00000C	SLC1A3	0.03232869	0.32362351	0.41980103
ENSG000002	SMIM10L1	0.3227011	0.66858463	0.42024282
ENSG000001	PPM1K	0.01483053	0.26260224	0.42041218
ENSG000001	IL36A	0.17444094	0.53940156	0.42047262
ENSG000001	JPH3	0.27825975	0.63759905	0.42048398
ENSG000002	PCDHGA4	0.14045874	0.50035294	0.42052615
ENSG000001	GOLGA6L4	0.40657219	0.7275473	0.42058021

ENSG000001	ASAH2	0.40988716	0.72927217	0.42064685
ENSG000001	KCTD14	0.42746324	0.73955032	0.4212586
ENSG000002	C15orf65	0.27956225	0.63854799	0.42145556
ENSG000001	MAP1LC3C	0.28243164	0.64048081	0.42149946
ENSG000001	RAB41	0.05731165	0.37687699	0.42160946
ENSG000001	GPR22	0.29945864	0.6542516	0.42194012
ENSG000002	ACCSL	0.35237905	0.69082603	0.42222825
ENSG000002	ZCWPW2	0.14226111	0.50068215	0.42234293
ENSG000001	TLR7	0.01489796	0.26260224	0.4223696
ENSG000001	CMTR1	0.01528309	0.2637104	0.42296985
ENSG000001	DNTT	0.25922863	0.62303431	0.42303154
ENSG000001	MAG	0.40362651	0.72672584	0.42306083
ENSG000001	GRID1	0.27587757	0.63548653	0.42328084
ENSG000001	HCRTR1	0.11764365	0.47422542	0.42385331
ENSG000001	CFAP46	0.1466449	0.50510677	0.4241454
ENSG000001	TAT	0.40970119	0.72918099	0.42467144
ENSG000001	C9orf152	0.41583585	0.73235476	0.42513278
ENSG000001	RAPGEF5	0.12057324	0.47724625	0.42619863
ENSG000001	PARVA	0.13340412	0.49240713	0.42687711
ENSG000001	HHIPL2	0.20426481	0.5688169	0.4272894
ENSG000002	PCDHGB6	0.27981455	0.63864899	0.42743239
ENSG000001	TTC21A	0.09231431	0.43743098	0.42755648
ENSG000001	FRZB	0.3274506	0.67217574	0.42759731
ENSG000001	ZNF732	0.06701382	0.39524277	0.42811871
ENSG000001	GDF7	0.17483737	0.53975461	0.42885333
ENSG000002	IQCA1L	0.17845512	0.54343132	0.42958668
ENSG000002	HSPA1A	0.23630301	0.60336718	0.42971745
ENSG00000C	HSD17B14	0.22750138	0.59541263	0.42990756
ENSG000001	SERPINB9	0.0024991	0.2093801	0.43058553
ENSG000002	N/A	0.37526752	0.70720386	0.43107352
ENSG000001	AKR1C2	0.35995541	0.69504054	0.43222713
ENSG000001	ZCCHC2	0.02902884	0.31345545	0.43238136
ENSG000001	ZNF684	0.05123089	0.3647548	0.43268019
ENSG000001	KLB	0.39095701	0.71964297	0.4343169
ENSG000001	ZBP1	0.07933836	0.4152849	0.43449974
ENSG000001	SLC36A3	0.24873661	0.61463039	0.434596
ENSG000001	SSC4D	0.05202422	0.36656028	0.43466446
ENSG000001	EPB41L5	0.00369072	0.21208655	0.43485451
ENSG00000C	COL19A1	0.03771052	0.33514272	0.43496338
ENSG000002	ANKRD65	0.29045614	0.648324	0.43497062
ENSG000002	N/A	0.35848609	0.69382497	0.43518153
ENSG000001	SSC5D	0.07396438	0.4057296	0.43558234
ENSG000001	OR3A3	0.31112459	0.65949478	0.43598577

ENSG000001	CRHR2	0.33737019	0.67926926	0.43651953
ENSG000002	SIK1B	0.00564519	0.21875144	0.43669307
ENSG000001	TMEM132C	0.29634512	0.65199276	0.43670899
ENSG000002	TCL1B	0.17560224	0.54061531	0.4372648
ENSG000001	GALM	0.06160828	0.38545034	0.43802401
ENSG000001	H3C12	0.24060279	0.60721888	0.43880185
ENSG000001	FAM228A	0.16014715	0.5230958	0.4389846
ENSG000001	LRRN4CL	0.30937621	0.65916845	0.43913737
ENSG000002	TWIST2	0.32201583	0.66822528	0.43962947
ENSG000001	MOGAT1	0.34219179	0.68310736	0.44035336
ENSG000001	CASP7	0.00024405	0.18011717	0.441358
ENSG000001	RNF213	0.02632275	0.30699826	0.44161913
ENSG000001	DIRAS2	0.2617353	0.62545731	0.44171357
ENSG000002	LCN8	0.26247063	0.62589744	0.44197679
ENSG000001	IL7	0.04760973	0.35871889	0.44285369
ENSG000002	N/A	0.4039356	0.72672584	0.44309586
ENSG00000C	CYP46A1	0.12878425	0.48697483	0.4436323
ENSG000002	C1orf53	0.35822956	0.69382497	0.44364139
ENSG00000C	ARNTL2	0.03881381	0.33829709	0.44364432
ENSG000001	KLRG2	0.29045128	0.648324	0.44371349
ENSG000001	FAM160A1	0.27126145	0.63286144	0.44373116
ENSG00000C	SERPIND1	0.15572611	0.51796902	0.44436803
ENSG000001	TIMM10	0.02648964	0.30699826	0.44443855
ENSG000002	DNAAF4	0.26788799	0.63017463	0.4444477
ENSG000001	C9orf116	0.23461695	0.6022886	0.44446403
ENSG000001	LPAR6	0.02270029	0.29463194	0.44453284
ENSG000001	PLS3	0.04515532	0.35545023	0.44527391
ENSG000001	NECTIN4	0.25482069	0.6197628	0.44557416
ENSG000002	SEBOX	0.15577032	0.51796902	0.44572896
ENSG000002	PERCC1	0.23790217	0.60402516	0.44581351
ENSG000001	RNF165	0.006489	0.22296216	0.44597521
ENSG000001	CLDN3	0.32203157	0.66822528	0.44600013
ENSG000001	NPIPA1	0.25668605	0.62074989	0.44609773
ENSG000001	LYPD5	0.13533288	0.49446444	0.44643244
ENSG000001	GRIN3A	0.04406517	0.35392484	0.44676533
ENSG000001	NPIPB6	0.07354032	0.40498137	0.44689357
ENSG000002	PGA4	0.18238178	0.54678908	0.44733757
ENSG000001	CEP20	0.0177348	0.27425066	0.44855648
ENSG000001	TLR3	0.01121881	0.2451811	0.44904274
ENSG00000C	SCML1	0.03064118	0.31953017	0.44924137
ENSG000001	SP7	0.35205099	0.69071745	0.44932717
ENSG000002	VWA7	0.23069615	0.59855451	0.44954795
ENSG000001	CCN1	0.26583109	0.62868632	0.4495596

ENSG000001	CXCL9	0.30269358	0.65591444	0.44958904
ENSG000001	C4BPB	0.1642591	0.52769983	0.44986576
ENSG000001	UGT8	0.05685095	0.37585055	0.45015123
ENSG00000C	SNCB	0.17978604	0.54485895	0.45064664
ENSG000001	KANSL1	0.04885038	0.360767	0.45072025
ENSG000001	KCNIP4	0.00704934	0.22661511	0.45078459
ENSG000002	OR2A1	0.13983825	0.4999841	0.45080137
ENSG000001	PRICKLE2	0.31082033	0.65949478	0.45082167
ENSG000002	KLRC4-KLRK:	0.03013212	0.31859418	0.45123574
ENSG000001	MICU3	0.13300671	0.49183101	0.45129275
ENSG000001	CD40	0.00079831	0.18856694	0.45160407
ENSG000001	NSMCE3	0.13532569	0.49446444	0.45184866
ENSG000001	ZNF80	0.02066518	0.28641584	0.4521772
ENSG000001	CCDC74B	0.38157944	0.7106995	0.45258757
ENSG000001	ZNF474	0.27931128	0.63830383	0.45317274
ENSG000002	SPINK9	0.36365507	0.69694698	0.45327803
ENSG000001	SOX5	0.07822627	0.41427772	0.45329842
ENSG000001	SYNPO2	0.02236965	0.29338932	0.45356283
ENSG000001	ALDOB	0.33277121	0.67523724	0.45469692
ENSG000001	CLRN1	0.29044535	0.648324	0.45480633
ENSG000001	SPARCL1	0.44882425	0.75183734	0.45497173
ENSG00000C	WDFY1	0.00628251	0.22079879	0.45526591
ENSG00000C	LAMB4	0.21413385	0.57986845	0.45538315
ENSG000001	SLC27A3	0.25358295	0.61921212	0.45540753
ENSG000001	CPNE6	0.30375948	0.65635638	0.45555311
ENSG000001	NR1I2	0.2995172	0.6542516	0.45602295
ENSG000001	CD164L2	0.24535683	0.61141177	0.45614868
ENSG000001	BOLL	0.2533286	0.61912837	0.45642254
ENSG000002	N/A	0.17260443	0.53689236	0.45781928
ENSG000001	N/A	0.14984382	0.50942953	0.45797494
ENSG000001	FOXA3	0.05516951	0.37354464	0.45850003
ENSG000001	SLC14A2	0.07285761	0.40435477	0.45856783
ENSG000002	HLA-DPB1	0.10512801	0.45525417	0.45898554
ENSG000001	SLC10A6	0.30299735	0.65591444	0.45932874
ENSG000001	SUSD5	0.25862777	0.6223087	0.45941469
ENSG000001	GNRHR	0.20619953	0.56956674	0.45941919
ENSG000001	RMI2	0.06882656	0.39825823	0.45945439
ENSG00000C	GRAMD1B	0.0208157	0.28641584	0.45947808
ENSG000001	CATSPER3	0.08245895	0.42182925	0.45950735
ENSG000001	OR5B21	0.28563005	0.64333814	0.45983964
ENSG000001	ABI3BP	0.10579023	0.4561473	0.46024716
ENSG000001	IRF6	0.05330961	0.36981204	0.46027467
ENSG000001	PTPRG	0.33828659	0.67998455	0.46088061

ENSG000002	HSPA1B	0.10550709	0.45598045	0.46130833
ENSG000002	N/A	0.21160033	0.57620716	0.46156985
ENSG000002	LILRA4	0.2170656	0.58371881	0.46176946
ENSG000001	FBXO6	0.06734041	0.39585774	0.4619728
ENSG000002	H3C8	0.28637089	0.64387022	0.46269578
ENSG00000C	TLL2	0.30475572	0.65635638	0.46357214
ENSG000001	ZNF670-ZNF	0.38787635	0.71672142	0.46365705
ENSG000002	N/A	0.31228295	0.66090624	0.46379006
ENSG000001	GCHFR	0.1326063	0.49124539	0.46392065
ENSG000001	GABRB3	0.19187739	0.55723719	0.46400136
ENSG000002	TIGD1	0.03677931	0.33341755	0.46400279
ENSG000001	DNAJC12	0.24410149	0.6099532	0.46409008
ENSG000001	GLT8D2	0.14579754	0.50430399	0.46409661
ENSG000002	ARL14EPL	0.23074168	0.59855451	0.46417835
ENSG000001	HPCA	0.26571267	0.62855137	0.46436685
ENSG000001	COL20A1	0.24566688	0.61158271	0.46443811
ENSG000001	PLCB4	0.36652874	0.69875861	0.46540293
ENSG000001	RAX2	0.20739875	0.57126603	0.46571633
ENSG000002	GOLGA8N	0.19806779	0.56540555	0.46590334
ENSG000001	TLDC2	0.00103741	0.18856694	0.46687877
ENSG000001	RUFY4	0.0879988	0.43050213	0.46689275
ENSG000001	SPATA22	0.02645419	0.30699826	0.46713313
ENSG000001	UCP1	0.24994148	0.61582699	0.46790186
ENSG000001	LDHC	0.14461079	0.50315089	0.46806929
ENSG000001	TNFAIP6	0.10256186	0.45201921	0.46817452
ENSG000001	OR2T10	0.24170921	0.60725942	0.46831581
ENSG000001	KIF7	0.22569143	0.59319591	0.46871793
ENSG000001	IL17RD	0.37356432	0.70563635	0.46896803
ENSG000001	EFHD1	0.13485232	0.49418805	0.46897471
ENSG000001	KIRREL2	0.15962025	0.52219103	0.46909821
ENSG000001	SYN3	0.30180889	0.65561042	0.46988145
ENSG000002	N/A	0.35414409	0.69167785	0.46988353
ENSG000001	HTR1F	0.26349459	0.62687307	0.47043943
ENSG000001	PLCH1	0.09447089	0.43981122	0.47130775
ENSG000002	TARM1	0.20193844	0.56775306	0.47139445
ENSG000001	GABRB2	0.12626294	0.48388732	0.4716576
ENSG000001	STARD4	0.005506	0.21833269	0.47191012
ENSG000001	FAM156B	0.14929757	0.50925123	0.47269884
ENSG000001	TTLL2	0.21040143	0.57453195	0.47286656
ENSG000001	STAT2	0.03690083	0.33341755	0.4733561
ENSG000001	AGBL4	0.42871775	0.74045488	0.47412097
ENSG000001	FCN3	0.14066868	0.50051652	0.47413951
ENSG000001	XRN1	0.0121319	0.25234748	0.47431856

ENSG000002	N/A	0.02279952	0.29500624	0.47545397
ENSG000001	SLC22A3	0.30373042	0.65635638	0.47569758
ENSG000002	FAM24B	0.0193911	0.28170518	0.4763245
ENSG000001	PRLR	0.01945097	0.28172722	0.47651955
ENSG000001	LINGO2	0.27777352	0.6371251	0.4765203
ENSG000002	N/A	0.26087626	0.6244683	0.47690594
ENSG000001	STAC2	0.2067872	0.57036149	0.4773736
ENSG000001	OR2C1	0.29343428	0.6501794	0.47747892
ENSG00000C	CD38	0.00667152	0.22330866	0.47809452
ENSG000001	CCL1	0.33105098	0.67443203	0.47831482
ENSG000001	B3GNT4	0.03735929	0.33471496	0.47961521
ENSG000001	OR1F1	0.17718206	0.5420963	0.47980252
ENSG000001	NCOA7	0.01363489	0.25910565	0.47997126
ENSG000002	FANK1	0.05618837	0.37502119	0.48003161
ENSG000001	ENTPD8	0.32089305	0.66766831	0.48031956
ENSG00000C	TGFB2	0.11885182	0.47555373	0.48090568
ENSG000001	PKD1L1	0.0687943	0.39825823	0.48090661
ENSG000001	KIAA1958	0.01776896	0.27425066	0.48122159
ENSG000001	FREM3	0.11554059	0.4710215	0.4814347
ENSG000002	OR2A42	0.03580635	0.33046641	0.48231682
ENSG000002	PCDHGA7	0.30507063	0.65643145	0.48271137
ENSG000002	N/A	0.21111966	0.57528121	0.48299578
ENSG000002	TNFSF12-TNI	0.31854727	0.66591607	0.4836112
ENSG000002	BTBD17	0.29561624	0.65140093	0.48383045
ENSG000001	KLRF1	0.00486412	0.21772133	0.48397094
ENSG000001	CABYR	0.07590824	0.41015711	0.48423168
ENSG000001	SFRP4	0.41352787	0.73118284	0.48520261
ENSG000001	FLG	0.2564688	0.62074989	0.48547775
ENSG000001	PPP1R9A	0.02665989	0.30700471	0.485625
ENSG000001	CCDC38	0.3291659	0.67287758	0.48585665
ENSG000002	IFITM5	0.25545875	0.62030905	0.48644328
ENSG00000C	IL12RB2	0.00030463	0.18856694	0.48660073
ENSG000002	ANTXRL	0.28697022	0.64432975	0.4874796
ENSG000001	COL9A1	0.38242882	0.71160862	0.48803866
ENSG000001	CHRNA5	0.27145555	0.63291553	0.48818481
ENSG000001	GPM6A	0.00310187	0.20994948	0.48884657
ENSG000001	LRRN4	0.30229337	0.65591444	0.48957267
ENSG000001	WNT7B	0.30842408	0.65815242	0.48960418
ENSG000001	PANX3	0.13755981	0.49833972	0.49002969
ENSG000001	FHAD1	0.00447274	0.21750928	0.49011934
ENSG000001	ZNF251	0.27788526	0.63729138	0.49026918
ENSG000001	SLC35F4	0.25593478	0.62056088	0.49048874
ENSG000001	CPNE4	0.32833926	0.67254253	0.49060348

ENSG000001	SGCD	0.323421	0.66947817	0.49070284
ENSG000002	N/A	0.26891349	0.63079288	0.49073839
ENSG000002	N/A	0.26447795	0.6274921	0.49077228
ENSG000001	SMIM10	0.19729376	0.56500135	0.49101126
ENSG000001	STAT1	0.03711707	0.3336458	0.49105641
ENSG000001	COL22A1	0.17010507	0.53437545	0.49196377
ENSG000002	CFAP97D1	0.38267637	0.71174298	0.49285695
ENSG000001	CCN2	0.26364442	0.62687307	0.49287328
ENSG000001	TEKT5	0.35285404	0.69109369	0.49302
ENSG000001	RARRES1	0.22401318	0.59143122	0.49310002
ENSG000001	MPDZ	0.23117309	0.59903565	0.49331599
ENSG000001	MOV10	0.03181762	0.3227302	0.4934302
ENSG000001	CKM	0.15415264	0.51533385	0.49534753
ENSG000001	IFITM3	0.12942032	0.4871968	0.4968417
ENSG000001	LYPD1	0.20132364	0.56773237	0.49708059
ENSG000001	CDHR4	0.17949837	0.54466737	0.4972423
ENSG000001	SPINK7	0.19806407	0.56540555	0.49769488
ENSG000001	GJD3	0.25792945	0.62198226	0.49813321
ENSG000001	TIGD4	0.3385971	0.68022544	0.49816453
ENSG000001	IQCA1	0.12708509	0.48495433	0.49823081
ENSG000001	TMEM232	0.31256384	0.66090624	0.49828982
ENSG000001	CPA6	0.27274294	0.63378205	0.49835125
ENSG000001	TMEM123	0.00664751	0.22330866	0.49848858
ENSG000001	AGRN	0.06698247	0.39524277	0.49853462
ENSG000001	ITPK1	0.07729854	0.41245361	0.4991338
ENSG000002	NPIPA3	0.28002013	0.63893857	0.49936375
ENSG000001	AIM2	0.03475801	0.32831054	0.49979177
ENSG000001	AIF1L	0.06050524	0.38425153	0.50021434
ENSG000001	LHFPL5	0.18129993	0.54622749	0.50041217
ENSG000001	CLDN4	0.300771	0.65493729	0.50068545
ENSG000001	RPL39L	0.03162909	0.32198214	0.50091869
ENSG00000C	CD5L	0.24315206	0.6087049	0.5015617
ENSG000001	TENT5B	0.08935218	0.43342334	0.50192827
ENSG000001	TAL2	0.30166754	0.65553823	0.50206244
ENSG000001	ANO5	0.00357397	0.21208655	0.50240022
ENSG000002	ZYG11A	0.25587472	0.62055829	0.50336939
ENSG000002	SMIM11A	0.08778996	0.43050213	0.503847
ENSG000001	TIMM23B-AG	0.01499522	0.26260224	0.50450569
ENSG00000C	PRDM6	0.19543122	0.56292651	0.50463951
ENSG000001	MRAP2	0.13888402	0.49941019	0.50468248
ENSG000001	MYOF	0.01400969	0.25910565	0.50472754
ENSG000002	N/A	0.17462902	0.53975461	0.50536501
ENSG000002	OR2AG1	0.18798614	0.55211256	0.50612724

ENSG000002	N/A	0.02434722	0.29927225	0.50643182
ENSG00000C	PI4K2B	0.00869682	0.23436355	0.50650412
ENSG000002	N/A	0.11720336	0.47331831	0.50708681
ENSG000001	PLSCR1	0.02241527	0.29351345	0.50779229
ENSG000001	GPR83	0.19087462	0.55647457	0.50825727
ENSG000001	SOCS1	0.05998783	0.38277429	0.50843894
ENSG000002	TAF15	0.10621845	0.4561473	0.50851464
ENSG000001	SULT1E1	0.17880835	0.54395471	0.50891167
ENSG000001	EGR2	0.01071311	0.24206404	0.5089193
ENSG000001	SLC8A2	0.35579942	0.69207883	0.50964928
ENSG000001	GPR31	0.09890903	0.4460792	0.50965345
ENSG000002	KRTAP5-8	0.21839159	0.58528507	0.50985479
ENSG00000C	CFAP61	0.28267257	0.64066925	0.51103806
ENSG000002	MYOCOS	0.23161649	0.599371	0.51140064
ENSG000001	WARS1	0.06956744	0.40004644	0.51152557
ENSG000001	GNG12	0.22086463	0.58818746	0.51192069
ENSG00000C	EIF2AK2	0.01761033	0.27418797	0.51212573
ENSG000001	SCN3A	0.00067921	0.18856694	0.51216382
ENSG000002	ZSCAN5C	0.17498199	0.53975461	0.51264876
ENSG000001	ADAMTS16	0.35184255	0.6905543	0.5126887
ENSG000002	CCDC194	0.09961668	0.44746644	0.51297039
ENSG000002	ZBED6	0.31887887	0.66619096	0.51309896
ENSG00000C	SLAMF7	0.01123836	0.2451811	0.51318586
ENSG000001	ADAM21	0.30991981	0.65935115	0.51326483
ENSG000001	RPS17	0.23180494	0.599371	0.51369113
ENSG000001	BRCA2	0.00731671	0.22727528	0.51499486
ENSG000001	IFNW1	0.06670566	0.39496052	0.51557279
ENSG000001	CD2AP	0.00237806	0.2093801	0.5165987
ENSG000001	INTS3	0.14248566	0.50068215	0.51850861
ENSG000001	BHMT2	0.12705377	0.48495433	0.5193069
ENSG000002	OR5K2	0.24993489	0.61582699	0.52089059
ENSG000002	ATP5MF-PTCI	0.30791501	0.65762361	0.52121007
ENSG000001	CCDC148	0.19448633	0.56193152	0.52123553
ENSG000001	PRR19	0.26731663	0.62949703	0.52155674
ENSG000001	SLC39A2	0.25999728	0.62338681	0.5217762
ENSG000001	PTP4A1	0.26361184	0.62687307	0.52192795
ENSG000001	CELF5	0.27366416	0.63403495	0.52258128
ENSG000001	SPDYE17	0.19132658	0.55685897	0.52275449
ENSG000001	RGS13	0.33737253	0.67926926	0.52292603
ENSG000001	BATF2	0.26709709	0.62929976	0.52318341
ENSG000002	N/A	0.23495022	0.60232491	0.52359557
ENSG00000C	TREM2	0.29644513	0.65199276	0.52403152
ENSG000001	GBP6	0.25055877	0.61641253	0.52430272

ENSG000002	CTAGE8	0.16507683	0.5286578	0.52446145
ENSG000001	FANCL	0.00014324	0.18011717	0.52464628
ENSG00000C	POU4F3	0.30072916	0.65493729	0.5248962
ENSG000001	KCNH8	0.0040528	0.21403539	0.52566731
ENSG000002	N/A	0.28019297	0.6389512	0.52637726
ENSG000001	NYAP2	0.2619277	0.62561343	0.5264985
ENSG000001	FAM124A	0.29966542	0.6542516	0.52680647
ENSG000002	N/A	0.3080011	0.65762361	0.52683586
ENSG000001	TEKT3	0.22589463	0.59319591	0.52687007
ENSG000001	MESP2	0.21112067	0.57528121	0.5271861
ENSG000001	CLK2	0.13447847	0.49367555	0.52732313
ENSG000001	DHX58	0.03748001	0.33471496	0.52750385
ENSG000001	HES4	0.08132268	0.41882561	0.52779615
ENSG000001	KRT7	0.24053937	0.60721888	0.52857518
ENSG000001	PCDH9	0.0054976	0.21833269	0.52862889
ENSG000001	PFN4	0.17064676	0.53457173	0.52866781
ENSG00000C	ZC3H11A	0.08455821	0.42469888	0.52915734
ENSG000001	USP41	0.29228934	0.64947398	0.5297091
ENSG000001	P2RY14	0.02998558	0.31859418	0.53086799
ENSG000001	NRN1	0.0978524	0.44463533	0.53132021
ENSG000001	SYT6	0.24011018	0.60689277	0.53146845
ENSG000001	OR2B2	0.19638773	0.5638811	0.53185147
ENSG000002	FSBP	0.24302151	0.60856573	0.53269714
ENSG000001	GLIPR1L2	0.02695957	0.30875752	0.53332996
ENSG000002	ABCF2-H2BE	0.20173678	0.56773237	0.53482002
ENSG000001	CNTNAP2	0.16704031	0.53159417	0.53482819
ENSG000001	MT2A	0.07168022	0.40258447	0.53517795
ENSG00000C	IFI35	0.05523685	0.37354464	0.53641748
ENSG000002	N/A	0.20374097	0.56880022	0.5379892
ENSG00000C	SCN4A	0.0571008	0.37665007	0.53813547
ENSG000001	VAMP5	0.0248184	0.29983359	0.54011065
ENSG000002	CLEC6A	0.00289866	0.20994948	0.54053599
ENSG000002	SAMD9	0.01088297	0.24206404	0.54118121
ENSG000001	CYP2F1	0.17219266	0.53633075	0.54194498
ENSG000002	VSTM5	0.17901544	0.54414332	0.54252177
ENSG000001	GJA3	0.07366771	0.40523875	0.54270792
ENSG000001	MAP1B	0.08869743	0.43274646	0.54447746
ENSG000001	CLDN6	0.16734845	0.53173243	0.54570776
ENSG000001	RASL11B	0.19942749	0.56600138	0.54606104
ENSG00000C	RGS1	0.02840757	0.31345545	0.5462479
ENSG000001	KIAA0408	0.13954395	0.49975817	0.54653335
ENSG000001	RASGEF1B	0.00064914	0.18856694	0.5471487
ENSG000002	SCRT1	0.20824176	0.5719087	0.54742283

ENSG00000(	ABCB5	0.14089451	0.50051652	0.54882586
ENSG000001	IL10	0.07299155	0.40435477	0.54920495
ENSG000001	PRELID3A	0.11811678	0.4748744	0.54923677
ENSG000001	DDX58	0.04194222	0.34923892	0.55036187
ENSG000001	MIPOL1	0.23061363	0.59855451	0.55054057
ENSG000001	ACSM5	0.22822205	0.59645659	0.55081606
ENSG000001	SYNC	0.15088644	0.51146865	0.55093336
ENSG000001	ARHGAP27	0.07230722	0.40373033	0.55124146
ENSG000001	BCL2L14	0.31064975	0.65949478	0.55137378
ENSG00000(	PARP12	0.01214008	0.25234748	0.55151987
ENSG000001	RBP2	0.32789687	0.67237631	0.55155474
ENSG000001	VWA5B2	0.25542544	0.62030905	0.55267686
ENSG000001	SGCG	0.1881079	0.5521164	0.55270046
ENSG000002	N/A	0.25125833	0.61695463	0.55441213
ENSG000001	ABCA12	0.16599017	0.5290896	0.55447561
ENSG000001	GTF2H2C	0.00399506	0.21403539	0.55487984
ENSG000001	IL21	0.24058473	0.60721888	0.55675459
ENSG000001	OASL	0.11193483	0.46578315	0.55698699
ENSG000001	CCDC196	0.34251095	0.68338046	0.55761129
ENSG000001	MKRN3	0.08613352	0.42703813	0.55846459
ENSG000001	DNAAF1	0.17769836	0.5423686	0.55963075
ENSG000001	RAB39A	0.01192431	0.25192551	0.56082314
ENSG000001	RASSF6	0.1303036	0.48839785	0.56131095
ENSG000001	ENTHD1	0.2248519	0.59200912	0.5613572
ENSG000001	FAN1	0.0760076	0.41035311	0.56206518
ENSG000001	GBP3	0.0021553	0.20144452	0.56346804
ENSG000002	FAM72D	0.04970686	0.36170272	0.56365037
ENSG000001	CADM2	0.16460255	0.52786788	0.56399477
ENSG000001	PARP14	0.02942145	0.31615886	0.56869048
ENSG000001	CELA1	0.12213114	0.47887061	0.56940603
ENSG000001	DCDC1	0.19784789	0.56540555	0.56950235
ENSG000001	NCMAP	0.27704001	0.63661176	0.56968641
ENSG000001	VXN	0.23592949	0.60336718	0.56987351
ENSG000001	PDIA2	0.22068723	0.5880238	0.5701853
ENSG000001	INHBA	0.18243196	0.54678908	0.5704515
ENSG000001	CHURC1-FN	0.27293355	0.63378205	0.57103497
ENSG000001	PGAP4	0.16931724	0.53356366	0.57201886
ENSG000002	N/A	0.07926243	0.4152849	0.57211208
ENSG000001	SH2D7	0.24289671	0.60853999	0.57300967
ENSG000001	PRPF8	0.13347091	0.49240713	0.57331568
ENSG000001	GRHL3	0.25234869	0.61819338	0.57353646
ENSG000001	IFIT2	0.07007209	0.40047888	0.57364237
ENSG000002	N/A	0.210083	0.57424655	0.5738191

ENSG000001	TACR1	0.17193584	0.53604496	0.57482815
ENSG000001	ZDHHC15	0.0588483	0.38083692	0.57483459
ENSG000001	SPOCK3	0.23792244	0.60402516	0.57491642
ENSG000001	PRR16	0.16030311	0.52339464	0.57571959
ENSG000001	FBXO39	0.16933261	0.53356366	0.57599753
ENSG000001	PNPT1	0.01910165	0.28068185	0.5779174
ENSG000001	KLHL31	0.14210533	0.50068215	0.57854301
ENSG000002	SERPINE3	0.0941927	0.43939485	0.57880682
ENSG000001	MNS1	0.30029797	0.65483993	0.5789957
ENSG000001	ELAVL4	0.32126044	0.66766831	0.58105648
ENSG000001	NT5C1B	0.23196695	0.599371	0.58169442
ENSG000002	CCDC187	0.18360778	0.54871392	0.58285254
ENSG000002	SMIM18	0.25447479	0.61954604	0.58334053
ENSG000001	CALCB	0.22694843	0.59448256	0.58409703
ENSG000001	UNC79	0.01646905	0.27000801	0.58505756
ENSG000002	ZIM2	0.11782399	0.4744792	0.58545667
ENSG000001	EXOC3L1	0.10006437	0.44762396	0.58563495
ENSG00000(	CHGB	0.1896753	0.55424774	0.58703715
ENSG000001	AXDND1	0.15090864	0.51146865	0.58752137
ENSG00000(	NGFR	0.04619092	0.35738124	0.58761311
ENSG000001	NRG2	0.18893739	0.55371723	0.58810514
ENSG000001	SPATA45	0.15550753	0.51741305	0.58847612
ENSG000001	CYP3A7	0.20021436	0.56699578	0.5890004
ENSG000001	ZNF100	0.03618998	0.33198688	0.58905452
ENSG00000(	FERMT2	0.20249082	0.56807486	0.5913865
ENSG000001	TRIM22	0.0072956	0.22727528	0.59271139
ENSG000001	NEURL3	0.15908456	0.5217239	0.59326947
ENSG000001	FKBP10	0.20822759	0.5719087	0.59365058
ENSG000001	TRIM6	0.02368436	0.29875884	0.59386849
ENSG000001	FGF7	0.14955664	0.50925123	0.59433603
ENSG000001	CYP2J2	0.04030813	0.34428359	0.5952059
ENSG000001	VWA3B	0.27050308	0.63178344	0.59726387
ENSG000001	LAD1	0.13987975	0.4999841	0.59741879
ENSG000001	ESM1	0.14366864	0.50186843	0.59839368
ENSG00000(	FBLN1	0.10454461	0.45514301	0.59884965
ENSG000001	PAX9	0.24136551	0.60721888	0.59937322
ENSG000001	SLCO5A1	0.10329665	0.45287405	0.60020083
ENSG000001	HERC6	0.01491092	0.26260224	0.60048264
ENSG000001	GASK1A	0.15650347	0.51913113	0.60054022
ENSG000001	NAPRT	0.25898326	0.62261049	0.60107803
ENSG000001	GALNTL6	0.08023945	0.41684053	0.60125917
ENSG000001	PGAP1	0.0096262	0.2371784	0.60166532
ENSG000002	N/A	0.01782491	0.27425066	0.60228872

ENSG000001	CFAP20DC	0.18159153	0.54632234	0.60255516
ENSG000001	USP50	0.17762623	0.5423686	0.60255927
ENSG000001	GRB7	0.1509654	0.51152445	0.60356987
ENSG000001	MT1G	0.1661846	0.52950145	0.60402554
ENSG000002	APOL6	0.01515103	0.26260224	0.6041714
ENSG000001	PKP3	0.10898845	0.46084517	0.60463333
ENSG000001	CCL17	0.07791902	0.41349453	0.60470796
ENSG000001	HAPLN2	0.18908089	0.55377099	0.60571885
ENSG000001	CHRNA7	0.19488036	0.56243733	0.60661983
ENSG000001	FRMD3	0.04745559	0.35865951	0.60804904
ENSG000001	IFIT5	0.01239522	0.25464329	0.608751
ENSG000001	ANKFN1	0.09863201	0.44547647	0.61032116
ENSG000001	FAM133A	0.19880138	0.56598606	0.61041051
ENSG000001	ANXA10	0.25625797	0.62056088	0.61384904
ENSG000002	DNLZ	0.02999172	0.31859418	0.61629432
ENSG000001	MYO1B	0.04944104	0.36112199	0.61683607
ENSG000002	GNG10	0.06942718	0.39974794	0.61696899
ENSG000001	TRIM67	0.24381009	0.60950645	0.61769724
ENSG000002	AMY2A	0.10266963	0.45201921	0.61781251
ENSG000001	KRT23	0.09524448	0.44084511	0.61817372
ENSG000001	TRIM64B	0.21089206	0.57523547	0.61903375
ENSG000001	KLHL4	0.20203394	0.56775306	0.6191119
ENSG000001	GRIA3	0.17582494	0.54079741	0.61933529
ENSG000002	SPESP1	0.07313041	0.40493325	0.6197893
ENSG000001	PYCR3	0.19068629	0.55616548	0.62012833
ENSG000001	CFAP52	0.05581536	0.37502119	0.62287045
ENSG000001	RGPD3	0.03523397	0.32931219	0.62325686
ENSG000001	CORO2B	0.0185987	0.27889987	0.62364888
ENSG000001	IFIT3	0.08905236	0.4331765	0.62635636
ENSG00000(	MRPS24	0.00671654	0.22330866	0.62762195
ENSG00000(	FMO2	0.18008822	0.54501079	0.62802249
ENSG000001	SHOX2	0.17956785	0.54474936	0.62951231
ENSG000002	MTRNR2L4	0.19673891	0.56409142	0.62975323
ENSG000001	CSPG4	0.04714078	0.35851546	0.63033265
ENSG000001	IL26	0.1617062	0.52428584	0.63051242
ENSG00000(	ALX4	0.14020192	0.5002427	0.63095582
ENSG000002	PCDHGA12	0.31378898	0.66194513	0.63175327
ENSG000001	GJA9	0.17725854	0.54222813	0.63179833
ENSG000001	RDH13	0.09016173	0.43395124	0.63294941
ENSG000001	HSF2BP	0.148672	0.50820783	0.6350866
ENSG000001	NPFFR1	0.11884248	0.47555373	0.63554239
ENSG000002	SMN2	0.20091717	0.56770499	0.63605076
ENSG000002	PCDHGA6	0.14547769	0.50417338	0.63722724

ENSG000002	C19orf84	0.01057296	0.24206404	0.638358
ENSG000002	VIT	0.06268427	0.38773505	0.63944572
ENSG000001	INHBC	0.11970398	0.47604098	0.63945985
ENSG000001	ANGPTL7	0.1769574	0.54177807	0.63983625
ENSG000001	PAH	0.07694735	0.41152638	0.64055765
ENSG000001	CFAP77	0.23785681	0.60402516	0.64253157
ENSG000001	KCNK12	0.14901025	0.50903493	0.64260489
ENSG000001	IFIH1	0.0141232	0.25910565	0.64395446
ENSG000001	PPP1R36	0.17003026	0.53437545	0.64408106
ENSG000001	TTC9B	0.14805575	0.50706204	0.64465892
ENSG000001	NANOS3	0.13618934	0.49597876	0.64534843
ENSG000002	TMEM178B	0.12025929	0.47672121	0.64543903
ENSG000001	LHFPL1	0.17934636	0.54453715	0.64838952
ENSG000001	DNAI1	0.17145794	0.53568647	0.64862555
ENSG000002	GABBR1	0.13851325	0.49898885	0.64915234
ENSG000001	DDR2	0.00892282	0.23513527	0.64933467
ENSG000001	H1-6	0.08785179	0.43050213	0.65015578
ENSG000001	C3orf70	0.10737316	0.45831177	0.65094078
ENSG000001	GCH1	0.00170487	0.19438665	0.65101814
ENSG000002	DHX16	0.19691559	0.5644983	0.65166068
ENSG000001	KCNJ1	0.19478155	0.56225208	0.65169317
ENSG000001	GDPD2	0.09176401	0.43630228	0.65261114
ENSG000002	SMTNL1	0.08720173	0.42932594	0.6527968
ENSG000001	PIFO	0.13562547	0.49519918	0.6529395
ENSG000001	ZACN	0.09859643	0.44547647	0.65350574
ENSG000001	CRISPLD1	0.12762271	0.48588488	0.65431142
ENSG000002	H4C14	0.153467	0.51472848	0.65481991
ENSG000002	TTLL13P	0.28591099	0.6436707	0.65745574
ENSG000001	ANKRD22	0.1140359	0.46962187	0.65759677
ENSG000001	SLC25A32	0.00885862	0.23464493	0.65795457
ENSG000001	GBP5	0.07741788	0.41266462	0.65865481
ENSG000001	MKX	0.17494437	0.53975461	0.66010231
ENSG000001	ZNF320	0.00727044	0.22727528	0.66168851
ENSG000001	KLRC4	0.00910883	0.23513527	0.66268218
ENSG000001	CLEC20A	0.18657833	0.55095275	0.66281688
ENSG000001	SEPTIN4	0.10206046	0.45141805	0.66595358
ENSG000001	ELOVL2	0.11627825	0.47160917	0.66611475
ENSG000001	FAM83H	0.06296124	0.38841248	0.66622341
ENSG000001	RBP4	0.11331133	0.4685093	0.66643691
ENSG000001	SLC34A2	0.17805168	0.54301749	0.66730881
ENSG000001	TMPRSS2	0.12397499	0.48115857	0.66892076
ENSG000001	ISM2	0.044265	0.35453012	0.66965932
ENSG00000C	SPA17	0.05467475	0.37354464	0.67118901

ENSG000001	CD80	0.06414461	0.39043354	0.67135811
ENSG000001	RTP4	0.02199704	0.29231997	0.67193228
ENSG000001	TEKT1	0.13614407	0.49597739	0.67224789
ENSG000001	VWA5B1	0.06238303	0.38624952	0.67248166
ENSG000002	N/A	0.05401221	0.37208157	0.67363662
ENSG000001	OTOF	0.10841934	0.45963571	0.67373723
ENSG000001	NAPEPLD	0.03394338	0.32553969	0.67379653
ENSG000001	IMPG1	0.20143337	0.56773237	0.67440891
ENSG000001	CLTRN	0.21537839	0.58129262	0.67491573
ENSG000001	SERPING1	0.17063579	0.53457173	0.67606968
ENSG000001	GBP1	0.0541981	0.37256839	0.67772633
ENSG000001	LENG8	0.07204539	0.40351881	0.67903948
ENSG000001	TSLP	0.23675867	0.60364584	0.67978734
ENSG000001	VIPR2	0.07578771	0.41015711	0.68006408
ENSG00000(	OAS1	0.02431719	0.29927225	0.68028474
ENSG000001	BAALC	0.07786769	0.4134525	0.68068469
ENSG000001	HTR4	0.08602578	0.42681511	0.68255675
ENSG000002	AKR1C8P	0.14119123	0.50055066	0.68261427
ENSG000001	EPHX4	0.14643577	0.50492197	0.68282646
ENSG00000(	F11	0.01772427	0.27425066	0.68460494
ENSG000001	DBX1	0.04847332	0.360767	0.68465811
ENSG000001	AQP8	0.08367942	0.42260872	0.68508542
ENSG000001	PDCD1LG2	0.11876199	0.47554586	0.68564188
ENSG00000(	LAP3	0.01635687	0.27000801	0.68580157
ENSG000001	NUPR1	0.21188458	0.57666273	0.68670803
ENSG000002	KLRC3	0.01973917	0.28263225	0.68727076
ENSG000001	RASSF8	0.09733858	0.44383219	0.6875369
ENSG000002	N/A	0.09836883	0.4448142	0.68854058
ENSG000001	GSC	0.04433489	0.35453012	0.68948841
ENSG000002	KRTAP5-9	0.16581124	0.52885285	0.68955684
ENSG000001	SMOC1	0.06480038	0.39055819	0.69102484
ENSG000002	DGKK	0.01396207	0.25910565	0.69137639
ENSG000002	KLF14	0.12897937	0.4871454	0.69219553
ENSG000001	PLIN1	0.04379895	0.35372007	0.69343078
ENSG000001	GBP4	0.0262021	0.30624	0.69600016
ENSG000001	APOBEC4	0.02067985	0.28641584	0.69661826
ENSG000001	PRAP1	0.06567857	0.39264465	0.69667923
ENSG000002	OR4E1	0.19222905	0.55805885	0.69906539
ENSG000001	LIPM	0.1253933	0.48335545	0.69918001
ENSG000001	TNP2	0.09891686	0.4460792	0.69971281
ENSG000002	N/A	0.18921689	0.55396947	0.70005035
ENSG000001	MDC1	0.12583046	0.48382419	0.70169026
ENSG000001	ZP1	0.04769374	0.35895177	0.70281076

ENSG00000(	CRTAC1	0.11637301	0.47160917	0.70671266
ENSG000001	ZNF114	0.06533532	0.39169239	0.70820534
ENSG00000(	DCN	0.12928012	0.4871968	0.70870662
ENSG000002	ACACA	0.08086674	0.41789729	0.71139634
ENSG000001	OAS2	0.01871045	0.27889987	0.71192342
ENSG000001	SAMD9L	0.01518105	0.26260224	0.71228623
ENSG000001	MORN2	0.05459665	0.37354464	0.71282142
ENSG000001	TFEC	0.00999934	0.2408892	0.71513522
ENSG000001	KRTAP4-6	0.07974739	0.4152849	0.71524507
ENSG000001	GJD4	0.0758425	0.41015711	0.71533966
ENSG000001	LDHAL6B	0.15968756	0.52219103	0.71534288
ENSG000002	CCDC39	0.00329165	0.2114129	0.71549225
ENSG000001	TMEM132B	0.16534375	0.52878404	0.71615148
ENSG000001	RAB3B	0.06724695	0.39561184	0.71747905
ENSG000001	HJV	0.17989708	0.54485895	0.71828538
ENSG000001	SEZ6L2	0.13665049	0.49674408	0.71981221
ENSG000002	HIGD1C	0.20292737	0.56858183	0.72109938
ENSG000001	CALHM6	0.03076382	0.31953017	0.72270495
ENSG000001	LRRC49	0.13818334	0.49898885	0.72295147
ENSG000001	ENPEP	0.09466758	0.43988973	0.7234565
ENSG000001	IL5	0.12998706	0.48787839	0.72529215
ENSG000001	MIP	0.00937533	0.23551879	0.72651494
ENSG00000(	SOX30	0.11795002	0.47451798	0.72746807
ENSG000001	DDX60	0.00904022	0.23513527	0.72782624
ENSG00000(	KITLG	0.15827909	0.52089921	0.72838449
ENSG000001	ZNF843	0.05566231	0.37502119	0.72893345
ENSG000001	A1CF	0.11003004	0.46261866	0.72980428
ENSG000001	EPSTI1	0.03922696	0.33969504	0.73075867
ENSG000002	N/A	0.14678655	0.50533677	0.73094833
ENSG000001	C2orf66	0.12058863	0.47724625	0.73294014
ENSG000002	N/A	0.10600603	0.4561473	0.7334094
ENSG000001	CNTN4	0.03559512	0.33014896	0.73403096
ENSG000001	IFI6	0.04430506	0.35453012	0.73467469
ENSG000001	HESX1	0.13135121	0.4894044	0.73504001
ENSG00000(	ETV7	0.12569312	0.4837353	0.73632103
ENSG000001	HSD11B1	0.11463294	0.47026153	0.73689884
ENSG000001	LRRC26	0.07843676	0.41430635	0.73716694
ENSG000001	BAAT	0.02803736	0.31159655	0.73807567
ENSG000002	EXOC3L4	0.19874122	0.56598606	0.73858261
ENSG000001	FGF8	0.11106537	0.46430702	0.7393254
ENSG000001	CHRNB3	0.09372006	0.43879258	0.73932689
ENSG000001	FOXB1	0.05938221	0.38178501	0.73964548
ENSG000002	C4B	0.22147966	0.58882965	0.74033797

ENSG000001	EDN3	0.14595205	0.50430399	0.74163485
ENSG00000(	YTHDC1	0.06317066	0.38911305	0.74196315
ENSG000001	CES1	0.05451443	0.37332384	0.74330962
ENSG000001	XAF1	0.006223	0.22079879	0.74407423
ENSG000001	SINHCAF	0.04884228	0.360767	0.74433482
ENSG000002	CDC14C	0.14465725	0.50317048	0.74472523
ENSG000002	TAP2	0.00422232	0.21614597	0.74548352
ENSG000002	HLA-DMA	0.02392515	0.29875884	0.74548823
ENSG000002	PRSS2	0.06590793	0.39329183	0.74587452
ENSG000002	N/A	0.02971672	0.31817936	0.74713236
ENSG000002	TRIM26	0.12579688	0.48382419	0.74774438
ENSG000001	C1QB	0.07957501	0.4152849	0.74821436
ENSG000001	KLHL10	0.04753558	0.35865951	0.75231493
ENSG000001	INTU	0.16040945	0.52341736	0.75260532
ENSG000001	IDO1	0.0309499	0.31953017	0.75265175
ENSG00000(	USP2	0.10528854	0.45552092	0.75301546
ENSG000001	WFDC2	0.16047125	0.52341736	0.75309294
ENSG000001	SMCO3	0.13258825	0.49124539	0.75340241
ENSG000001	MX1	0.04053964	0.34535266	0.75457263
ENSG000001	DYNLT2	0.09604908	0.44204901	0.75522476
ENSG000001	GLIPR1L1	0.07792654	0.41349453	0.75962001
ENSG000001	IL6	0.16312452	0.52653704	0.76030606
ENSG000002	TPBGL	0.0258856	0.30450132	0.76059824
ENSG000001	SPIN2A	0.12121473	0.47724625	0.76063639
ENSG000002	PCDHGA9	0.03415093	0.32634691	0.76118362
ENSG000001	KIAA1217	0.10832157	0.45963571	0.76332641
ENSG000001	APOL4	0.08286524	0.42228867	0.76494014
ENSG000001	CAVIN4	0.11066789	0.46348065	0.76513588
ENSG000001	CD274	0.03763227	0.33471496	0.76667738
ENSG000001	ISG15	0.07195501	0.40315841	0.76696599
ENSG000001	TBX10	0.13104761	0.4894044	0.7677139
ENSG000001	PLET1	0.20641341	0.56989193	0.76838422
ENSG000001	SEMA3D	0.08427765	0.42405214	0.76885599
ENSG000001	DCHS2	0.10108861	0.44917064	0.76956218
ENSG000001	PAPPA2	0.09319319	0.43758758	0.77103806
ENSG000002	KRTAP10-2	0.13504097	0.49428772	0.77117378
ENSG000001	PENK	0.0879949	0.43050213	0.77164912
ENSG00000(	HECW1	0.0265316	0.30699826	0.77242523
ENSG000001	SV2B	0.06141529	0.38542193	0.77262861
ENSG000001	TNFSF18	0.0353717	0.32950679	0.77375329
ENSG00000(	CHRNA3	0.12162484	0.47828106	0.77431536
ENSG000001	CMYA5	0.02055678	0.28641584	0.77473754
ENSG000001	IL1A	0.0895516	0.43356546	0.78074026

ENSG000001	CPO	0.11316577	0.46821512	0.7854417
ENSG000001	RGSL1	0.05523214	0.37354464	0.79099911
ENSG000001	VIP	0.12251323	0.47910582	0.79238176
ENSG000002	N/A	0.16838975	0.53253292	0.79274452
ENSG000002	PSMB11	0.06602913	0.39343668	0.79469532
ENSG000001	DNALI1	0.13969276	0.49975817	0.79636419
ENSG000001	KCNV2	0.05380944	0.37194714	0.79881561
ENSG000002	CAPNS2	0.01294366	0.25583328	0.80067158
ENSG000001	GPC5	0.09582765	0.44171811	0.800937
ENSG000001	FILIP1	0.07943752	0.4152849	0.80352908
ENSG000001	NAT8	0.12197226	0.47873532	0.80667013
ENSG00000C	SAMD4A	0.00635797	0.22284683	0.80978769
ENSG000001	PPP4R4	0.04618262	0.35738124	0.80985558
ENSG000001	CCDC42	0.02042735	0.28641584	0.81365186
ENSG000002	PNMA2	0.11077295	0.46375519	0.81532544
ENSG000002	C1orf146	0.11613251	0.47160917	0.81624562
ENSG000002	GOLGA8S	0.03391195	0.3254305	0.81694537
ENSG000001	VSTM4	0.10226564	0.45160695	0.81785095
ENSG000001	GALNT16	0.04985123	0.36170272	0.81931835
ENSG000001	ADAMTS3	0.02723539	0.30967858	0.82027829
ENSG000001	INSL6	0.00484103	0.21772133	0.82180101
ENSG000001	SYCE1	0.09272688	0.43753271	0.8220194
ENSG000002	VPS52	0.09697476	0.44311879	0.8241502
ENSG000001	TMEM255A	0.01740485	0.27265457	0.82555741
ENSG000001	SLC6A11	0.01741349	0.27265457	0.82711345
ENSG000001	GPR156	0.07774118	0.41335649	0.8282758
ENSG000001	ADAMTS8	0.00773832	0.23002982	0.82995039
ENSG000001	ATF3	0.07436044	0.40673667	0.83431762
ENSG000001	CREB3L1	0.08149534	0.41887936	0.83509982
ENSG000001	OR56B1	0.11927768	0.47602132	0.83645099
ENSG000001	SPATS2L	0.01535422	0.26409578	0.83720815
ENSG000001	HERC5	0.04150163	0.34755298	0.83782244
ENSG000001	CXCL11	0.1374234	0.49827463	0.84035891
ENSG000002	MICA	0.03587346	0.33046641	0.84381193
ENSG000002	ARHGAP23	0.04897139	0.360767	0.8440271
ENSG000002	LY75-CD302	0.04189634	0.34903589	0.84418082
ENSG000001	CCSER1	0.01263287	0.25512421	0.84652054
ENSG000001	SPATA21	0.02703606	0.3093296	0.84870327
ENSG000001	NANOS1	0.08793991	0.43050213	0.84999758
ENSG00000C	C8B	0.00505713	0.21833269	0.85166043
ENSG000001	IFIT1	0.04132971	0.34725125	0.85172886
ENSG000001	NKX6-3	0.00653394	0.22296216	0.85179621
ENSG000001	PLSCR2	0.01779294	0.27425066	0.85355747

ENSG000001	A4GNT	0.11912591	0.47578591	0.8564229
ENSG000001	C6orf58	0.0664051	0.3940861	0.85652745
ENSG000001	SLC25A47	0.09633182	0.44215472	0.85832703
ENSG000001	TEX38	0.08410802	0.42379805	0.85984975
ENSG000001	PDE6A	0.0174486	0.27294123	0.86287576
ENSG000001	GRM7	0.07589362	0.41015711	0.86430079
ENSG000001	IDO2	0.04569912	0.35605405	0.86615324
ENSG000001	ACKR4	0.08168184	0.41943963	0.86829919
ENSG000001	SLIT3	0.04326099	0.35262482	0.87403643
ENSG000001	KCNJ8	0.11146374	0.46477576	0.8746022
ENSG000001	NCAM2	0.0230868	0.29687456	0.87569684
ENSG000002	HLA-DOA	0.0677794	0.39689346	0.87595403
ENSG000001	ROPN1B	0.16245312	0.52523922	0.87709069
ENSG00000(	SCARF1	0.04432421	0.35453012	0.87730461
ENSG000001	ID1	0.08255667	0.4220168	0.87910259
ENSG000002	OR52I1	0.02200721	0.29231997	0.88224262
ENSG000002	GPANK1	0.01372307	0.25910565	0.8986612
ENSG000001	DGKI	0.10514277	0.45525417	0.90065813
ENSG000001	CNTN2	0.06421338	0.39043354	0.90146112
ENSG000001	OAS3	0.02504712	0.30062982	0.90545988
ENSG000001	SHBG	0.08374926	0.42270336	0.91014743
ENSG000001	PCDHB9	0.02723373	0.30967858	0.91092795
ENSG000001	S100G	0.07054375	0.40156223	0.91191083
ENSG000001	DDN	0.02644934	0.30699826	0.91346097
ENSG000001	MAGED4	0.04974383	0.36170272	0.91759098
ENSG000002	HLA-DRA	0.01146474	0.24590889	0.91908175
ENSG000002	CTAGE6	0.03608066	0.33117113	0.92325532
ENSG000001	SH3BGR	0.05023822	0.36227834	0.93368447
ENSG00000(	SIGLEC1	0.02142291	0.2908707	0.9431408
ENSG000002	BCL2L2-PABI	0.01461596	0.26165299	0.94928956
ENSG000001	ULBP2	0.0643706	0.39043354	0.95100731
ENSG000001	ZNF695	0.03797865	0.3358711	0.95110673
ENSG000001	PARD3B	0.0205982	0.28641584	0.95140964
ENSG000002	N/A	0.07826023	0.41427772	0.95274162
ENSG000001	ACOD1	0.12199105	0.47873532	0.95274627
ENSG000001	GPR45	0.04550331	0.3556589	0.9608041
ENSG000001	CMPK2	0.01582213	0.26687746	0.96176857
ENSG000002	CTXND1	0.05393985	0.37208157	0.96198595
ENSG000001	FAT4	0.00899389	0.23513527	0.96244305
ENSG000002	ERVW-1	0.01712269	0.27230282	0.96617568
ENSG000001	PROX1	0.03740362	0.33471496	0.96803594
ENSG000002	TAS2R14	0.00256518	0.2093801	0.97581625
ENSG000001	GUCY2F	0.00264353	0.2093801	0.97861701

ENSG000001	PPIC	0.00607496	0.22079879	0.99253863
ENSG000002	TRIM75P	0.00875285	0.23436355	0.99757898
ENSG000001	CCDC81	0.01047447	0.24174018	0.99897778
ENSG000001	TRIM61	0.04133945	0.34725125	0.99942851
ENSG000001	CPE	0.1049488	0.45514301	1.00573218
ENSG000001	SCN2A	0.03117685	0.32005877	1.00696409
ENSG000001	RP1L1	0.06143653	0.38542193	1.01202476
ENSG000001	CCL2	0.13052624	0.48861254	1.01681149
ENSG000001	MSTN	0.02071083	0.28641584	1.02363038
ENSG000001	ERICH3	0.01468177	0.26167713	1.02437258
ENSG00000C	LAMP3	0.03875537	0.33829709	1.03021753
ENSG000001	ZNF177	0.04290001	0.35185871	1.03073096
ENSG000001	IFI44	0.00869581	0.23436355	1.03592801
ENSG000001	IFI27	0.1862376	0.55069275	1.0411756
ENSG000001	UNC80	0.04317107	0.35247453	1.04452431
ENSG000001	KCNH1	0.01950412	0.28172722	1.0492667
ENSG00000C	NSF	0.07830898	0.41427772	1.05306947
ENSG000002	KDM4E	0.00214366	0.20144452	1.05404043
ENSG000001	RLN1	0.05595857	0.37502119	1.06376147
ENSG000001	HCAR1	0.03745263	0.33471496	1.06780529
ENSG000001	TM4SF20	0.01791803	0.27425066	1.07639754
ENSG000002	N/A	0.01252742	0.2548969	1.08341658
ENSG000002	N/A	0.01736831	0.27265457	1.08354914
ENSG00000C	SEMA3A	0.01059131	0.24206404	1.08459449
ENSG000001	RSAD2	0.04708356	0.35851546	1.09738982
ENSG000002	GAB4	0.00574148	0.21912363	1.10860074
ENSG000001	STXBP6	0.03502779	0.32931219	1.10927188
ENSG000001	USP18	0.00742653	0.22881323	1.12114668
ENSG000001	GSDMD	0.01270511	0.25521652	1.1325562
ENSG000001	WDR64	0.0169261	0.27175434	1.14403677
ENSG000001	ABCA4	0.01063943	0.24206404	1.15166855
ENSG00000C	RAI14	0.00699412	0.22577227	1.15370095
ENSG000002	LMLN2	0.03712222	0.3336458	1.15522277
ENSG000001	IFI44L	0.00937547	0.23551879	1.16401553
ENSG000001	APCDD1L	0.02073517	0.28641584	1.16467738
ENSG000001	RHBDL3	0.03155229	0.32160363	1.17087746
ENSG00000C	PALMD	0.02621627	0.30624	1.17257953
ENSG000001	CCL25	0.02602929	0.30537396	1.17929673
ENSG00000C	DAZL	0.01663318	0.27031359	1.18287063
ENSG000001	BNIP5	0.02487408	0.30012694	1.1942054
ENSG000001	THRSP	0.00755311	0.22933195	1.19726944
ENSG000001	RNF151	0.01584095	0.26687746	1.20368779
ENSG000001	TAS1R1	0.00793367	0.23002982	1.21942222

ENSG000001	CCL8	0.04186718	0.34903589	1.30999303
ENSG000001	LY6E	0.00382011	0.21242163	1.31089675
ENSG000002	SKIV2L	0.02519238	0.30077106	1.31828928
ENSG000001	ANGPTL1	0.00457631	0.21750928	1.35507441
ENSG000001	TAS2R3	0.01794533	0.27436759	1.37948537
ENSG000001	SLIT2	0.0129916	0.2560008	1.38176084
ENSG000002	TCAF2C	0.00040016	0.18856694	1.39185202
ENSG000001	BMP3	0.00193278	0.19438665	1.60632205
ENSG000001	CXCL10	0.01246775	0.2548969	1.62218249
ENSG000001	CCDC168	6.10E-05	0.18011717	1.80141807
ENSG00000C	CD99	---	---	---
ENSG000001	VAMP7	---	---	---
ENSG000001	ASMTL	---	---	---
ENSG000001	SLC25A6	---	---	---
ENSG000001	P2RY8	---	---	---
ENSG000001	PLCXD1	---	---	---
ENSG000001	IL3RA	---	---	---
ENSG000001	CSF2RA	---	---	---
ENSG000002	ZBED1	---	---	---

V3 vs V1 Dupilumab Olink

Identifier	p-value	q-value	Difference
CCL17	2.83E-07	3.48E-05	-1.9454035
CCL26	3.88E-06	0.00028532	-1.593004
CCL13	1.97E-06	0.00018108	-1.0644459
PSIP1	0.00011308	0.00693551	-0.7476
TIMP3	0.04192812	0.30273248	-0.6579959
PDLIM7	0.03020433	0.28573449	-0.6439884
ATP5IF1	0.03308191	0.28573449	-0.6334846
CCL22	0.00386361	0.12047742	-0.6000383
NCK2	0.0370295	0.28573449	-0.5208884
SERPINB8	0.00321849	0.12047742	-0.5059769
CXCL3	0.06585214	0.34619411	-0.4950769
SPRY2	0.0335379	0.28573449	-0.47855
CD40	0.01714853	0.24271769	-0.4749269
VASH1	0.13109162	0.41232235	-0.4544268
FIS1	0.04266788	0.30273248	-0.4527269
IL32	0.00337671	0.12047742	-0.4526845
CLIP2	0.09899483	0.36384457	-0.4372308
LY6D	0.00383999	0.12047742	-0.4369462
SKAP2	0.05815029	0.32448666	-0.4344
MANF	0.09985951	0.36384457	-0.4264691
PPP1R9B	0.03802012	0.28573449	-0.4255385
LAT	0.01618457	0.24006939	-0.4246307
PDGFB	0.06303407	0.34217953	-0.4201037
DFFA	0.00664829	0.14391601	-0.4141231
DAPP1	0.1479929	0.43114849	-0.4105153
DECR1	0.03166813	0.28573449	-0.4104384
FXYD5	0.0226646	0.26905073	-0.4095691
MGMT	0.03804617	0.28573449	-0.4071884
MAP2K6	0.05100384	0.30273248	-0.394446
PLA2G4A	0.03627368	0.28573449	-0.3910653
NBN	0.03364432	0.28573449	-0.3878154
EGF	0.09899204	0.36384457	-0.3876499
SH2D1A	0.00392861	0.12047742	-0.3866268
HSPA1A	0.01203647	0.22091122	-0.3865191
IL16	0.00016901	0.00888523	-0.3819384
EIF4G1	0.0889835	0.35037213	-0.3649077
SRPK2	0.04708352	0.30273248	-0.3648961
METAP1D	0.05819598	0.32448666	-0.3632615
ENAH	0.01260635	0.22091122	-0.3628923
CCL24	0.01395151	0.23337074	-0.3598076
NCF2	0.1284886	0.41232235	-0.35895

IL18	0.00579617	0.13331199	-0.3573538
PADI2	0.0043914	0.1211768	-0.3572768
SHMT1	0.0465057	0.30273248	-0.3570538
YTHDF3	0.07845907	0.35037213	-0.3569883
IL6	0.24379057	0.51560304	-0.3513691
IRAK4	0.10947328	0.38005817	-0.3479538
CXCL1	0.04666241	0.30273248	-0.3361999
GOPC	0.16263928	0.43114849	-0.3337538
ANGPT1	0.10843157	0.38002685	-0.3283075
IL1B	0.00573019	0.13331199	-0.3273845
IL2	0.17869198	0.44740717	-0.327023
DBNL	0.09354021	0.3548742	-0.3228423
AXIN1	0.08093413	0.35037213	-0.3224846
CRKL	0.08667952	0.35037213	-0.3212808
IL20	0.01205822	0.22091122	-0.317573
TBC1D5	0.15378191	0.43114849	-0.3154614
CDSN	0.01070022	0.2187601	-0.3081423
IRAK1	0.02193031	0.26905073	-0.3023884
HEXIM1	0.07413938	0.35037213	-0.3008268
CD40LG	0.06322883	0.34217953	-0.2989577
SAMD9L	0.08450162	0.35037213	-0.2973038
TNFSF11	0.0288924	0.28573449	-0.2967115
FKBP1B	0.22177499	0.50087876	-0.2966307
GBP2	0.16102995	0.43114849	-0.2960268
PROK1	0.07296687	0.35037213	-0.292973
DNAJA2	0.08581373	0.35037213	-0.2919884
NUDC	0.01935279	0.26377139	-0.2905422
CCL7	0.14218741	0.43114849	-0.2898192
NUB1	0.10196507	0.36787399	-0.2885845
CCL28	0.03492301	0.28573449	-0.2877691
SCRN1	0.03089563	0.28573449	-0.2836576
PARP1	0.02863347	0.28573449	-0.2747615
CXCL6	0.08830731	0.35037213	-0.2745423
CRELD2	0.05051084	0.30273248	-0.259523
CCN2	0.06828259	0.35037213	-0.2587692
IL7	0.07807168	0.35037213	-0.2579346
CXCL14	0.04747944	0.30273248	-0.2578692
STX8	0.05477823	0.31497479	-0.2552462
ICA1	0.08617618	0.35037213	-0.2542654
RAB6A	0.00460999	0.1211768	-0.2526423
IL5	0.24615421	0.51762714	-0.2499576
TRIM21	0.07629403	0.35037213	-0.2475308
CLEC4A	0.01588554	0.24006939	-0.2471499

MVK	0.18349865	0.45018336	-0.2449268
F2R	0.05048957	0.30273248	-0.2441961
DAG1	0.03713342	0.28573449	-0.2436307
IL1A	0.1546591	0.43114849	-0.2409077
NT5C3A	0.23155636	0.50763644	-0.2409076
LSP1	0.0811456	0.35037213	-0.2379769
HCLS1	0.04909978	0.30273248	-0.2339038
EGLN1	0.03467037	0.28573449	-0.2335615
PRDX5	0.08683131	0.35037213	-0.2334038
EPCAM	0.02215806	0.26905073	-0.2327384
KYNU	0.04872947	0.30273248	-0.2269576
FABP1	0.35529983	0.61666566	-0.2261423
TRIM5	0.16241853	0.43114849	-0.2258807
FOXO1	0.16567201	0.43239219	-0.2255807
ITGB6	0.01630906	0.24006939	-0.2239038
SCGN	0.11677929	0.39067981	-0.2221423
NFATC1	0.16363984	0.43114849	-0.2183962
JUN	0.16860044	0.43626718	-0.2158922
IL17RB	0.0519471	0.30343703	-0.2157692
FABP9	0.03353225	0.28573449	-0.213573
HPCAL1	0.16952774	0.43626718	-0.2118807
BANK1	0.27800961	0.5654919	-0.2095307
IL17F	0.06566788	0.34619411	-0.2070653
MPIG6B	0.15206394	0.43114849	-0.2042038
PLXNA4	0.29662493	0.57150772	-0.2034423
PTPN6	0.15183656	0.43114849	-0.2031884
BCR	0.28608916	0.5654919	-0.1969423
KRT19	0.11272398	0.38333284	-0.1944269
PRDX3	0.28669463	0.5654919	-0.1944115
SCGB3A2	0.08157552	0.35037213	-0.1940653
EDAR	0.16218787	0.43114849	-0.1936461
MYO9B	0.39760927	0.65031205	-0.1930538
GALNT3	0.19798786	0.47489844	-0.1926269
IL15	0.11354152	0.38333284	-0.1916961
ARHGEF12	0.35241986	0.61666566	-0.18975
CKMT1A_CKI	0.44109262	0.68332342	-0.1875499
TNF	0.13415273	0.41837463	-0.1818538
NFATC3	0.15451129	0.43114849	-0.18055
CD70	0.09290466	0.3548742	-0.1772653
CASP2	0.30550492	0.58251715	-0.1766654
FGF2	0.14568333	0.43114849	-0.175823
DGKZ	0.16204954	0.43114849	-0.1742692
SPINT2	0.0749678	0.35037213	-0.1738961

TPP1	0.08519107	0.35037213	-0.1729653
EPO	0.10701355	0.37866334	-0.1720461
PRKAB1	0.17567706	0.44585626	-0.166473
TPT1	0.17274901	0.44146969	-0.1663461
MICB_MICA	0.02689651	0.28573449	-0.1631422
ANGPTL4	0.10375098	0.37068312	-0.1618769
CXCL8	0.25813794	0.53429145	-0.1604538
NPPC	0.15975765	0.43114849	-0.1580999
IL17A	0.41634121	0.66905486	-0.1571077
CCL11	0.2430307	0.51560304	-0.1561923
WAS	0.62734397	0.79607786	-0.1554423
HSD11B1	0.04729257	0.30273248	-0.1526692
TNFAIP8	0.57996295	0.76223702	-0.1509731
MAPK9	0.04382897	0.30273248	-0.1509423
ROBO1	0.14800937	0.43114849	-0.1490231
TGFB1	0.07432652	0.35037213	-0.1487807
NTF3	0.38566195	0.63976155	-0.1472154
LTO1	0.25843445	0.53429145	-0.1470308
SPON1	0.20131564	0.47489844	-0.14505
FGF19	0.59595539	0.77222389	-0.1404884
TNFRSF14	0.15069213	0.43114849	-0.1401769
IL5RA	0.12138925	0.39532074	-0.1396076
TFF2	0.2888926	0.5654919	-0.1390961
CCL21	0.11915041	0.3914942	-0.1387922
CXADR	0.34185677	0.6136746	-0.1387038
MERTK	0.1114231	0.38321216	-0.1386538
CEP164	0.12927631	0.41232235	-0.1366346
RABGAP1L	0.41022401	0.66211594	-0.1359846
TREM2	0.22321771	0.50087876	-0.1354423
DNER	0.08373195	0.35037213	-0.1307769
AGRP	0.30049188	0.57594277	-0.1300115
MZB1	0.16402388	0.43114849	-0.1298
TNFSF13	0.21936388	0.50087876	-0.1297038
AOC1	0.5075902	0.73252233	-0.1287577
PTPRM	0.08791795	0.35037213	-0.12795
FST	0.20486786	0.47716058	-0.1273077
CTSC	0.15134562	0.43114849	-0.1261269
MMP1	0.45286548	0.68607932	-0.1256
IL24	0.24306051	0.51560304	-0.1238115
LY75	0.15887411	0.43114849	-0.1234616
GLOD4	0.21693756	0.49895638	-0.1225154
IL33	0.36515901	0.62501635	-0.1210615
EIF5A	0.08735775	0.35037213	-0.1203807

CD84	0.03698491	0.28573449	-0.120023
WFIKKN2	0.21254107	0.49191896	-0.1191807
CD200R1	0.18115127	0.44740717	-0.1162846
NME3	0.20302631	0.47588332	-0.1149
FLT3LG	0.28472051	0.5654919	-0.1135346
TNFSF12	0.29644183	0.57150772	-0.1134423
LILRB4	0.33485875	0.6100397	-0.11275
CRHBP	0.19979641	0.47489844	-0.1108923
CCL23	0.35458769	0.61666566	-0.1104961
NCLN	0.5734081	0.75904382	-0.1102692
CD200	0.15589021	0.43114849	-0.1100654
IKBKG	0.52021839	0.74141681	-0.1100154
ENPP5	0.11825948	0.3914942	-0.10995
CNTNAP2	0.2875236	0.5654919	-0.1090769
CRIM1	0.23228884	0.50763644	-0.1090462
CXCL17	0.25841353	0.53429145	-0.1057769
PNPT1	0.441932	0.68332342	-0.1056
CXCL12	0.30916884	0.58487071	-0.1050962
CLSTN2	0.23312652	0.50763644	-0.1047461
AMN	0.37050808	0.63123598	-0.1041577
CLEC4G	0.22198619	0.50087876	-0.1041461
IL1R2	0.02077976	0.26905073	-0.1038423
VEGFD	0.09915146	0.36384457	-0.1036038
ACTN4	0.20042488	0.47489844	-0.1034461
AGRN	0.34597576	0.61666566	-0.1033154
PREB	0.18702889	0.45280678	-0.1019885
IL10RB	0.23067183	0.50763644	-0.1004385
SPINK4	0.38889142	0.63976155	-0.1002808
IL1RL2	0.44642937	0.68452503	-0.0998653
ADA	0.18063143	0.44740717	-0.0993654
CD4	0.13909526	0.43014334	-0.0978923
IL2RB	0.45698624	0.68607932	-0.0972461
RAB37	0.56677677	0.75706739	-0.0970769
LAMA4	0.31385206	0.58628202	-0.0959538
MATN2	0.32414022	0.60244244	-0.09595
LGMN	0.29483562	0.57150772	-0.0952192
CLEC7A	0.38302359	0.63976155	-0.0940731
DNPH1	0.53766817	0.74664863	-0.0935807
ANXA11	0.07817148	0.35037213	-0.0917346
MEGF10	0.33925098	0.61198215	-0.09
VEGFA	0.40100916	0.65297067	-0.0892231
PTH1R	0.24266307	0.51560304	-0.0874192
LAP3	0.38128459	0.63976155	-0.0871961

IL20RA	0.38014235	0.63976155	-0.0865923
ITGA6	0.18002166	0.44740717	-0.0864154
LIFR	0.3365919	0.61017645	-0.0862769
SMPDL3A	0.44041383	0.68332342	-0.0848692
ANGPTL2	0.5332149	0.74326926	-0.08475
LAMP3	0.44457035	0.68452503	-0.0802885
IL22RA1	0.47404069	0.69778789	-0.0799385
PRKCQ	0.31072713	0.58487071	-0.0796423
SULT2A1	0.66827032	0.81431615	-0.07885
ENPP7	0.42218782	0.67349325	-0.0787538
CKAP4	0.45389594	0.68607932	-0.0783192
FCAR	0.35320376	0.61666566	-0.0777231
MILR1	0.58949755	0.7692734	-0.0773307
DPP10	0.28218076	0.5654919	-0.0765038
GMPR	0.58445787	0.76541102	-0.0764769
PRSS8	0.4656821	0.688237	-0.0758461
BTN3A2	0.34906186	0.61666566	-0.0746192
BSG	0.27262792	0.56048645	-0.07435
LGALS4	0.54359014	0.74931043	-0.0728769
NELL2	0.28804295	0.5654919	-0.0728308
LTBR	0.44890157	0.68545965	-0.0718308
FGF5	0.52946294	0.74326926	-0.0705961
ICAM4	0.38942007	0.63976155	-0.0702077
PLAUR	0.52294658	0.74141681	-0.0686769
ERBB3	0.35692876	0.61666566	-0.0681346
CST7	0.61167339	0.78158267	-0.0677923
CD83	0.50623753	0.73252233	-0.0675846
SLAMF1	0.54770983	0.74931043	-0.0659384
SCG3	0.54783577	0.74931043	-0.0649731
TLR3	0.42276342	0.67349325	-0.0637654
ARNT	0.55420218	0.74931043	-0.0629039
BTN2A1	0.43345511	0.6810977	-0.0623346
LY9	0.50703391	0.73252233	-0.0605615
CD6	0.55587432	0.74931043	-0.0603654
B4GALT1	0.46146476	0.68752645	-0.0598115
CCL25	0.57082405	0.75835108	-0.0586692
CEACAM21	0.5304578	0.74326926	-0.0567231
CD244	0.43494011	0.6810977	-0.0560923
IL18R1	0.45862911	0.68607932	-0.0549231
TGFA	0.56618934	0.75706739	-0.0547577
LHPP	0.75564362	0.87999004	-0.054223
SLAMF7	0.55437677	0.74931043	-0.0536308
PSPN	0.64530726	0.80844121	-0.0531923

LGALS9	0.59262545	0.7706225	-0.0531654
CD58	0.42670753	0.67684643	-0.0516077
OSCAR	0.57631529	0.76015781	-0.0493692
TNFRSF4	0.65401181	0.80844121	-0.0484269
AGER	0.6061577	0.77994138	-0.0462231
IL10RA	0.73344115	0.86613291	-0.0455808
CRLF1	0.55410494	0.74931043	-0.0455154
WNT9A	0.5536362	0.74931043	-0.0451462
AMBN	0.46471618	0.688237	-0.0446231
IL3RA	0.64262864	0.80844121	-0.0435615
HLA-E	0.522267	0.74141681	-0.0426269
ADAM23	0.51729655	0.74141681	-0.0425346
RGS8	0.6198003	0.78922668	-0.0420423
NFASC	0.332652	0.6090345	-0.0406346
TPSAB1	0.35933848	0.61792786	-0.0392192
SIRPB1	0.73433008	0.86613291	-0.0386885
TNFRSF11A	0.72599971	0.86183192	-0.0374038
LRRN1	0.68800935	0.82809933	-0.0359961
CHRDL1	0.77113199	0.89237916	-0.0354692
GZMB	0.85614631	0.94613166	-0.0344961
SELPLG	0.33220163	0.6090345	-0.0341731
IFNGR1	0.63761269	0.80632808	-0.0339769
PAPPA	0.79687257	0.91354861	-0.0326423
CSF1	0.7686363	0.89229704	-0.0320462
PON3	0.45559496	0.68607932	-0.0319654
PRELP	0.67261824	0.81431637	-0.0306192
SIGLEC10	0.65593637	0.80844121	-0.0297731
IL15RA	0.81164596	0.92215481	-0.0276615
ARTN	0.81521907	0.92215481	-0.0266423
JCHAIN	0.74820664	0.87687912	-0.0265346
ISM1	0.77897102	0.89862488	-0.0257192
ESM1	0.82592156	0.92947747	-0.0247654
SIGLEC1	0.78465025	0.90234778	-0.0245423
IL12RB1	0.8422514	0.93672796	-0.0236961
EPHA1	0.75351645	0.87999004	-0.0233731
ITGA11	0.74307674	0.87364933	-0.0221923
TNFSF10	0.81690888	0.92215481	-0.0219308
TNFRSF13B	0.81376585	0.92215481	-0.0213885
TANK	0.87487042	0.95977273	-0.0196538
IL17C	0.92729217	0.98761962	-0.0187577
KLRB1	0.87892231	0.95977273	-0.0181577
MLN	0.91427373	0.97895324	-0.0165731
LAIR1	0.91510847	0.97895324	-0.0102962

PIK3AP1	0.95551104	0.99938502	-0.0093385
FSTL3	0.96557337	1	-0.0053885
CCL3	0.97489651	1	-0.0043615
PGF	0.97319142	1	-0.0034077
CD79B	0.97795898	1	-0.0028769
HLA-DRA	0.99790113	1	-0.0002615
OMD	0.99963224	1	-3.46E-05
BCL2L11	1	1	0
BID	1	1	0
MGLL	1	1	0
MMP10	0.99635875	1	0.00073462
ADGRE2	0.99225644	1	0.00093847
C1QA	0.98319373	1	0.00096538
TNFRSF11B	0.99222629	1	0.00097307
CCL4	0.99347977	1	0.00118848
SMOC2	0.96712459	1	0.00372691
MEPE	0.91403168	0.97895324	0.00485
IL17D	0.92857714	0.98761962	0.007
LTA	0.89039563	0.96656517	0.00708077
CCL20	0.97466535	1	0.00762694
REG4	0.9538942	0.99938502	0.00766536
CD22	0.87818185	0.95977273	0.00941154
PCDH1	0.65927757	0.80844121	0.00977692
PNLIPRP2	0.93681106	0.99328173	0.00991149
NRTN	0.94956507	0.99938502	0.01018462
ALDH3A1	0.9559335	0.99938502	0.01100002
BACH1	0.93929903	0.99328173	0.01130001
COL9A1	0.88390429	0.96235733	0.01153076
TNFRSF13C	0.90539661	0.97732236	0.01181538
SLC39A5	0.90561664	0.97732236	0.01346153
CD48	0.85277379	0.94524323	0.01686154
CTRC	0.83164438	0.93034367	0.02312692
IDS	0.09121058	0.35332097	0.02637691
HGF	0.83174747	0.93034367	0.02696153
IL11	0.87409144	0.95977273	0.02816923
IFNLR1	0.81253038	0.92215481	0.03254614
COLEC12	0.67269614	0.81431637	0.03281538
SCGB1A1	0.66012187	0.80844121	0.03435385
CLEC4C	0.68858259	0.82809933	0.03467306
FCRL3	0.65020122	0.80844121	0.03791923
PSMG3	0.84254607	0.93672796	0.03798462
FCRL2	0.52382709	0.74141681	0.04216923
SIT1	0.64967699	0.80844121	0.04649999

CDON	0.66125218	0.80844121	0.04735768
CLEC4D	0.72418461	0.86183192	0.05185769
GZMA	0.70956438	0.85055274	0.05391539
FCRL6	0.59972046	0.77437589	0.0551923
CTSO	0.32694886	0.60460895	0.06412691
IL12B	0.4764739	0.69857528	0.06470001
IL13	0.40850239	0.66211594	0.0792615
CD276	0.35594297	0.61666566	0.08020768
KLRD1	0.53166908	0.74326926	0.08517307
PKLR	0.71271172	0.85155167	0.09035768
IL10	0.6082695	0.77994138	0.09601536
CSF3	0.43272677	0.6810977	0.10444614
TRAF2	0.56780054	0.75706739	0.10510767
CXCL9	0.6549696	0.80844121	0.11180384
CD160	0.38278999	0.63976155	0.11837687
GAL	0.24334726	0.51560304	0.14669614
OSM	0.48818761	0.71290889	0.16748841
FASLG	0.31150722	0.58487071	0.17454612
NCR1	0.22880989	0.50763644	0.18385381
IL1RN	0.38800425	0.63976155	0.18758073
PTX3	0.08949723	0.35037213	0.20428459
ITM2A	0.13068663	0.41232235	0.23472691
CXCL10	0.18615775	0.45280678	0.36400765
IFNG	0.28655927	0.5654919	0.57329601
IL4R	4.48E-16	8.24E-14	2.17566872
IL4	6.78E-19	2.50E-16	5.96267176

RNA-seq QC

Sample Number	PatientID	Visit	Treatment	Entry QC	Sequenced reads	Sequenced bases	Assigned featureCounts	Detected protein-coding genes
1	2	1	Dupilumab	passed	66,253,400	19,876,020,000	36,390,576	15,666
2	2	2	Dupilumab	passed	43,247,900	12,974,370,000	20,133,808	14,905
3	2	3	Dupilumab	passed	75,336,200	22,600,860,000	38,268,359	15,566
4	4	1	Dupilumab	passed	57,590,200	17,277,060,000	33,184,475	15,605
5	4	2	Dupilumab	passed	32,477,500	9,743,250,000	18,122,189	15,033
6	4	3	Dupilumab	passed	54,564,100	16,369,230,000	32,629,390	15,682
7	5	1	Dupilumab	passed	60,883,400	18,265,020,000	28,556,162	14,923
8	5	2	Dupilumab	passed	57,291,900	17,187,570,000	26,357,012	14,802
9	6	1	Dupilumab	passed	65,080,400	19,524,120,000	29,751,733	15,029
10	6	2	Dupilumab	passed	55,270,600	16,581,180,000	29,294,344	15,280
11	6	3	Dupilumab	passed	51,267,800	15,380,340,000	28,260,352	15,109
12	7	1	Dupilumab	passed	56,230,800	16,869,240,000	30,299,020	15,410
13	7	2	Dupilumab	passed	50,974,200	15,292,260,000	22,952,861	14,884
14	8	1	Baricitinib	passed	55,965,200	16,789,560,000	30,644,203	15,273
15	8	2	Baricitinib	passed	56,918,900	17,075,670,000	32,098,434	15,526
16	8	3	Baricitinib	passed	66,627,400	19,988,220,000	33,859,853	15,339
17	9	1	Dupilumab	passed	63,974,800	19,192,440,000	31,344,215	15,159
18	9	2	Dupilumab	passed	56,920,100	17,076,030,000	25,646,893	14,889
19	9	3	Dupilumab	passed	54,841,000	16,452,300,000	30,987,130	15,522
20	14	1	Dupilumab	passed	55,743,900	16,723,170,000	28,372,256	15,161
21	14	2	Dupilumab	passed	61,976,600	18,592,980,000	30,491,198	15,284
22	14	3	Dupilumab	passed	64,625,700	19,387,710,000	36,969,462	15,746
23	15	1	Baricitinib	passed	56,722,500	17,016,750,000	31,533,079	15,581
24	15	3	Baricitinib	passed	36,507,300	10,952,190,000	22,697,002	15,210
25	16	1	Dupilumab	passed	69,328,200	20,798,460,000	33,937,866	15,340
26	16	2	Dupilumab	passed	36,315,300	10,894,590,000	18,331,435	14,896

27	16	3	Dupilumab	passed	54,730,800	16,419,240,000	25,358,971	14,950
28	17	1	Dupilumab	passed	42,556,600	12,766,980,000	23,782,742	15,045
29	17	2	Dupilumab	passed	35,788,100	10,736,430,000	18,924,170	15,080
30	17	3	Dupilumab	passed	65,739,200	19,721,760,000	34,613,315	15,569
31	18	1	Dupilumab	passed	37,153,100	11,145,930,000	16,846,433	14,606
32	18	2	Dupilumab	passed	65,555,300	19,666,590,000	33,004,587	15,265
33	18	3	Dupilumab	passed	54,850,500	16,455,150,000	28,298,425	14,744
34	19	1	Dupilumab	passed	36,342,700	10,902,810,000	19,380,500	15,135
35	19	2	Dupilumab	passed	62,307,200	18,692,160,000	34,938,088	15,463
36	19	3	Dupilumab	passed	32,350,500	9,705,150,000	17,959,769	15,276
37	20	1	Dupilumab	passed	56,400,100	16,920,030,000	31,940,906	15,441
38	20	2	Dupilumab	passed	56,851,700	17,055,510,000	32,141,641	15,458
39	20	3	Dupilumab	passed	34,899,200	10,469,760,000	18,862,852	15,125
40	21	1	Dupilumab	passed	49,344,700	14,803,410,000	28,731,807	15,419
41	21	2	Dupilumab	passed	31,994,200	9,598,260,000	16,839,820	14,916
42	21	3	Dupilumab	passed	41,850,500	12,555,150,000	26,087,846	15,514
43	23	1	Dupilumab	passed	65,861,000	19,758,300,000	37,959,393	15,536
44	23	3	Dupilumab	passed	35,188,600	10,556,580,000	21,535,295	15,271
45	26	1	Dupilumab	passed	61,792,100	18,537,630,000	29,452,580	15,500
46	26	2	Dupilumab	passed	54,373,100	16,311,930,000	27,438,229	15,504
47	27	1	Baricitinib	passed	52,907,500	15,872,250,000	33,589,490	15,501
48	27	3	Baricitinib	passed	31,743,800	9,523,140,000	19,414,617	15,348
49	28	1	Dupilumab	passed	65,576,400	19,672,920,000	35,559,306	15,554
50	28	3	Dupilumab	passed	37,793,400	11,338,020,000	21,538,784	15,148
51	29	1	Dupilumab	passed	77,150,400	23,145,120,000	46,745,383	15,558
52	29	2	Dupilumab	passed	75,652,200	22,695,660,000	42,929,722	15,503
53	29	3	Dupilumab	passed	51,799,700	15,539,910,000	27,885,991	15,107
54	31	1	Dupilumab	passed	54,053,200	16,215,960,000	31,075,398	15,546
55	31	2	Dupilumab	passed	47,566,100	14,269,830,000	26,356,095	15,410
56	31	3	Dupilumab	passed	67,948,200	20,384,460,000	38,555,580	15,374
57	32	1	Dupilumab	passed	53,944,100	16,183,230,000	32,654,873	15,559
58	32	2	Dupilumab	passed	59,040,300	17,712,090,000	35,822,368	15,714
59	32	3	Dupilumab	passed	53,416,900	16,025,070,000	32,404,805	15,538
60	33	1	Dupilumab	passed	47,040,000	14,112,000,000	27,302,993	15,491
61	33	2	Dupilumab	passed	58,307,900	17,492,370,000	34,112,125	15,662

62	33	3	Dupilumab	passed	49,336,900	14,801,070,000	28,812,627	15,547
63	34	1	Dupilumab	passed	41,774,800	12,532,440,000	22,622,030	15,164
64	34	3	Dupilumab	passed	51,703,200	15,510,960,000	31,551,456	15,798
65	35	1	Dupilumab	passed	44,836,100	13,450,830,000	25,880,094	15,749
66	35	3	Dupilumab	passed	51,965,600	15,589,680,000	28,111,525	15,159
67	36	1	Dupilumab	passed	61,261,400	18,378,420,000	33,094,036	15,456
68	36	2	Dupilumab	passed	52,603,700	15,781,110,000	28,232,951	15,266
70	37	1	Dupilumab	passed	50,793,400	15,238,020,000	25,823,436	15,109
71	37	2	Dupilumab	passed	51,802,800	15,540,840,000	30,283,376	15,409
72	37	3	Dupilumab	passed	49,442,400	14,832,720,000	29,538,940	15,132
73	38	1	Dupilumab	passed	53,902,300	16,170,690,000	30,305,624	15,604
74	38	2	Dupilumab	passed	48,135,100	14,440,530,000	24,893,192	15,313
75	38	3	Dupilumab	passed	56,187,500	16,856,250,000	31,516,592	15,715
76	39	1	Dupilumab	passed	44,057,500	13,217,250,000	24,634,807	15,019
77	39	3	Dupilumab	passed	60,758,400	18,227,520,000	33,734,913	15,417
78	40	1	Dupilumab	passed	49,302,300	14,790,690,000	25,261,025	15,148
79	40	2	Dupilumab	passed	45,662,400	13,698,720,000	24,273,774	15,120
80	40	3	Dupilumab	passed	45,405,200	13,621,560,000	25,632,545	15,247
81	41	1	Dupilumab	passed	60,468,800	18,140,640,000	30,129,172	15,174
82	41	3	Dupilumab	passed	57,581,100	17,274,330,000	30,565,212	15,488

Olink ID mapping and sample-level QC

SampleNumber	PatientID	Visit	Treatment	Manifest BXP ID	NPX SampleID	Sample QC	Below LOD (analyzable)
1	2	1	Dupilumab	BX0604_28	BX0604_28	PASS	13
2	2	2	Dupilumab	BX0604_15	BX0604_15	PASS	7
3	2	3	Dupilumab	BX0604_27	BX0604_27	PASS	12
4	4	1	Dupilumab	BX0604_51	BX0604_51	PASS	10
5	4	2	Dupilumab	BX0604_34	BX0604_34	PASS	5
6	4	3	Dupilumab	BX0604_39	BX0604_39	PASS	8
7	5	1	Dupilumab	BX0604_53	BX0604_53	PASS	13
8	5	2	Dupilumab	BX0604_19	BX0604_19	PASS	11
9	6	1	Dupilumab	BX0604_17	BX0604_17	PASS	4
10	6	2	Dupilumab	BX0604_55	BX0604_55	PASS	6
11	6	3	Dupilumab	BX0604_03	BX0604_3	PASS	10
12	7	1	Dupilumab	BX0604_13	BX0604_13	PASS	6
13	7	2	Dupilumab	BX0604_50	BX0604_50	PASS	8
14	8	1	Baricitinib	BX0604_72	BX0604_72	PASS	9
15	8	2	Baricitinib	BX0604_78	BX0604_78	PASS	9
16	8	3	Baricitinib	BX0604_56	BX0604_56	PASS	9
17	9	1	Dupilumab	BX0604_33	BX0604_33	PASS	16
18	9	2	Dupilumab	BX0604_84	BX0604_84	PASS	8
19	9	3	Dupilumab	BX0604_07	BX0604_7	PASS	9
20	14	1	Dupilumab	BX0604_25	BX0604_25	PASS	9
21	14	2	Dupilumab	BX0604_70	BX0604_70	PASS	15
22	14	3	Dupilumab	BX0604_30	BX0604_30	PASS	16
23	15	1	Baricitinib	BX0604_23	BX0604_23	PASS	4
24	15	3	Baricitinib	BX0604_06	BX0604_6	PASS	5
25	16	1	Dupilumab	BX0604_09	BX0604_9	PASS	10
26	16	2	Dupilumab	BX0604_40	BX0604_40	PASS	13
27	16	3	Dupilumab	BX0604_24	BX0604_24	PASS	14

28	17	1	Dupilumab	BX0604_54	BX0604_54	PASS	14
29	17	2	Dupilumab	BX0604_26	BX0604_26	PASS	13
30	17	3	Dupilumab	BX0604_74	BX0604_74	PASS	13
31	18	1	Dupilumab	BX0604_04	BX0604_4	PASS	7
32	18	2	Dupilumab	BX0604_21	BX0604_21	PASS	6
33	18	3	Dupilumab	BX0604_79	BX0604_79	PASS	13
34	19	1	Dupilumab	BX0604_63	BX0604_63	PASS	11
35	19	2	Dupilumab	BX0604_75	BX0604_75	PASS	9
36	19	3	Dupilumab	BX0604_68	BX0604_68	PASS	9
37	20	1	Dupilumab	BX0604_37	BX0604_37	PASS	5
38	20	2	Dupilumab	BX0604_67	BX0604_67	PASS	8
39	20	3	Dupilumab	BX0604_88	BX0604_88	PASS	9
40	21	1	Dupilumab	BX0604_32	BX0604_32	PASS	15
41	21	2	Dupilumab	BX0604_43	BX0604_43	PASS	12
42	21	3	Dupilumab	BX0604_73	BX0604_73	PASS	11
43	23	1	Dupilumab	BX0604_86	BX0604_86	PASS	14
44	23	3	Dupilumab	BX0604_35	BX0604_35	PASS	7
45	26	1	Dupilumab	BX0604_71	BX0604_71	PASS	18
46	26	2	Dupilumab	BX0604_46	BX0604_46	PASS	11
47	27	1	Baricitinib	BX0604_01	BX0604_1	PASS	9
48	27	3	Baricitinib	BX0604_76	BX0604_76	PASS	10
49	28	1	Dupilumab	BX0604_31	BX0604_31	PASS	6
50	28	3	Dupilumab	BX0604_83	BX0604_83	PASS	9
51	29	1	Dupilumab	BX0604_49	BX0604_49	PASS	10
52	29	2	Dupilumab	BX0604_82	BX0604_82	PASS	9
53	29	3	Dupilumab	BX0604_10	BX0604_10	PASS	13
54	31	1	Dupilumab	BX0604_85	BX0604_85	PASS	9
55	31	2	Dupilumab	BX0604_12	BX0604_12	PASS	9
56	31	3	Dupilumab	BX0604_65	BX0604_65	PASS	8
57	32	1	Dupilumab	BX0604_11	BX0604_11	PASS	8
58	32	2	Dupilumab	BX0604_38	BX0604_38	PASS	8
59	32	3	Dupilumab	BX0604_41	BX0604_41	PASS	8
60	33	1	Dupilumab	BX0604_45	BX0604_45	PASS	5
61	33	2	Dupilumab	BX0604_22	BX0604_22	PASS	7
62	33	3	Dupilumab	BX0604_36	BX0604_36	PASS	5

63	34	1	Dupilumab	BX0604_58	BX0604_58	PASS	7
64	34	3	Dupilumab	BX0604_05	BX0604_5	PASS	7
65	35	1	Dupilumab	BX0604_18	BX0604_18	PASS	5
66	35	3	Dupilumab	BX0604_44	BX0604_44	PASS	11
67	36	1	Dupilumab	BX0604_60	BX0604_60	PASS	16
68	36	2	Dupilumab	BX0604_20	BX0604_20	PASS	7
70	37	1	Dupilumab	BX0604_47	BX0604_47	PASS	16
71	37	2	Dupilumab	BX0604_29	BX0604_29	PASS	11
72	37	3	Dupilumab	BX0604_81	BX0604_81	PASS	9
73	38	1	Dupilumab	BX0604_80	BX0604_80	PASS	11
74	38	2	Dupilumab	BX0604_52	BX0604_52	PASS	13
75	38	3	Dupilumab	BX0604_14	BX0604_14	PASS	10
76	39	1	Dupilumab	BX0604_66	BX0604_66	PASS	10
77	39	3	Dupilumab	BX0604_62	BX0604_62	PASS	11
78	40	1	Dupilumab	BX0604_48	BX0604_48	PASS	13
79	40	2	Dupilumab	BX0604_64	BX0604_64	PASS	11
80	40	3	Dupilumab	BX0604_61	BX0604_61	PASS	7
81	41	1	Dupilumab	BX0604_02	BX0604_2	PASS	7
82	41	3	Dupilumab	BX0604_59	BX0604_59	PASS	7
83	1	1	Dupilumab	BX0604_57	BX0604_57	PASS	7
84	3	1	Baricitinib	BX0604_08	BX0604_8	PASS	11
85	3	2	Baricitinib	BX0604_77	BX0604_77	PASS	11
86	7	3	Dupilumab	BX0604_42	BX0604_42	PASS	18
87	10	1	Dupilumab	BX0604_87	BX0604_87	PASS	11
88	11	1	Dupilumab	BX0604_69	BX0604_69	PASS	11

Cohort data

Pt ID	Age	Sex	Comorbidities
2	37	m	
4	39	f	Conjuctivitis, urticaria, depression, anxiety, anorexia nervosa
5	84	m	Hypertension, hypercholesterolemia
6	61	f	Urticaria, hypertension, osteroporosis, food allergy
7	25	m	Conjuctivitis, food allergy, vitiligo, depression
8	22	m	
9	27	f	Urticaria, hypertension, migraine, depression
14	46	f	Conjuctivitis, food allergy, urticaria, alopecia, vitiligo
15	20	m	
16	65	f	Urticaria, hypertension, hypercholesterolemia, osteoporosis
17	27	m	Urticaria, depression
18	42	m	Conjuctivitis, food allergy
19	63	m	Food allergy, hypertension
20	22	m	
21	37	m	
23	39	m	
26	32	m	Food allergy, depression
27	26	m	Sleep apnea
28	29	f	Hypercholesterolemia
29	81	m	Hypertension
31	48	m	Urticaria
32	27	f	Food allergy, urticaria
33	NA	NA	NA
34	19	m	
35	48	m	Food allergy, prior MI
36	23	m	Conjuctivitis, urticaria, hypercholesterolemia, depression, anxiety, stress
37	25	f	
38	55	f	
39	66	f	Hypertension, hypercholesterolemia, depression, anxiety
40	37	m	
41	74	f	Urticaria, pancreatitis, hypertension, hypercholesterolemia, prior apoplexia cerebri
